# Microbiome modelling for post-mortem interval estimation across species and climates: swine analogues in a British summer and external validation in human donors from the USA

**DOI:** 10.1038/s41598-026-65366-y

**Published:** 2026-08-19

**Authors:** Andrea Bonicelli, Peter A. Cross, Lavinia Iancu, M. Eric Benbow, Noemi Procopio

**Affiliations:** 1 Research Centre for Field Archaeology and Forensic Taphonomy, School of Law and Policing, University of Lancashire, Fylde Road, Preston, PR1 2HE UK; 2 Department of Criminal Justice, University of North Dakota, Centennial Drive Stop, Grand Forks, 8050 ND US; 3 Department of Entomology and Department of Osteopathic Medical Specialties, Michigan State University, Fee Road, East Lansing, 48824 MI US

**Keywords:** Microbiome, Post-mortem interval (PMI), Forensic microbiology, Machine learning, Ecology, Ecology, Microbiology

## Abstract

Background Microbial clocks have shown high accuracy in estimating time since death, or post-mortem interval (PMI), across diverse contexts, including controlled laboratory settings, outdoor environments, and both human cadavers and animal models. However, the limited number of studies performed under different environmental and climatic conditions, particularly in Europe, restricts the broader applicability of these models, as data from distinct climates may not reliably translate across geographic regions. This study investigates the nasal thanatomicrobiome of pig carcasses exposed to outdoor summer conditions in the United Kingdom to assess the potential of microbial succession as a PMI estimator in a temperate climate. Model generalisation was further evaluated through an independent external validation on a published human thanatomicrobiome dataset spanning three geographically distinct states of the USA and all four seasons. Results Three pig carcasses were exposed for 10 days in June 2023, with duplicate swabs collected from both internal and external nares across 26 time points, yielding 312 samples in total. Microbial communities were profiled by 16S rRNA gene amplicon sequencing on an Illumina MiSeq platform; reads were processed in QIIME2 and taxonomy was assigned against the SILVA v138 database. A Random Forest regression model trained on combined internal and external nasal data achieved the highest predictive accuracy for PMI estimation (RÂ² = 0.96, MAE = 8.83 h), outperforming models built on external or internal swabs alone. Ambient temperature, which remained overall stable throughout the study period, was not a significant predictor under these conditions. Dominant taxa associated with decomposition succession included *Moraxella*, *Myroides*, *Ignatzschineria*, *Acinetobacter*, *Vagococcus*, *Pasteurella*, *Clostridium*, and *Savagea*; the prominence of *Ignatzschineria* and *Myroides* in particular is consistent with insect-associated microbial transfer, suggesting that carrion fly activity significantly shaped the microbial community succession at this site. External validation on a published human facial skin thanatomicrobiome dataset, sampled across all four seasons, confirmed robust model performance with an RÂ² = 0.594 and MAE = 30.3 ADD. Conclusions This study demonstrates that thanatomicrobiome-based PMI estimation can achieve high accuracy under temperate European conditions, and that results can be extended beyond the original experimental conditions, with model performance remaining robust across host species, geographic locations, anatomical location and inter-laboratory variation. However, predictive accuracy was optimised when models were trained on locally derived data, underscoring the importance of generating climate-specific models to improve the robustness and forensic applicability of microbial clocks in casework. By expanding reference datasets across diverse climatic environments and performing external validations, thanatomicrobiome-based microbial clocks have the potential to become a reliable and court-admissible tool for PMI estimation in forensic investigations.

**Supplementary Information:**

The online version contains supplementary material available at 10.1038/s41598-026-65366-y.

## Introduction

Understanding the dynamic processes that take place after death, particularly through the study of microbial communities associated with decomposing bodies, known as the thanatomicrobiota, is gaining significant interest in forensic sciences. One major application is the estimation of the post-mortem interval (PMI), or the time since death^1,2^. Traditionally, PMI is estimated through physiological indicators like algor, livor, and rigor mortis, supplemented by biochemical analyses such as the analysis of the concentration of potassium in vitreous humour^3^. Despite the scientific progresses towards the identification of new strategies for estimating PMI in objective and reliable ways, the only method used in the regular forensic practice for early PMIs remains the Henssge’s Nomogram method^4^. For longer PMIs, instead, practitioners rely on decomposition scoring (total body scores) and insect colonisation^5,6^. Total body score, however, often lacks of precision and its reliability is subject to operator interpretation^7^, whereas entomology is based on temperature and therefore it cannot be applied, for example, when the environmental temperature is unknown (*e.g.*, in cases where the temperature was not recorded when the body was discovered) or in cases where there is a suspicion that a body has been moved from its original site to a secondary location after death.

To improve the accuracy of PMI estimation, researchers are now focusing more onto microbial succession patterns. Specific microbial taxa dominate at distinct decomposition stages, offering a potential biological clock for PMI estimation^8^. In the early stages, endogenous anaerobes such as *Clostridium* and *Bacteroides* proliferate due to the anoxic conditions characterising the cadaver. As decomposition progresses, gas production alters the cadaveric internal chemistry, encouraging fermentative and facultative anaerobes^9–12^. In advanced stages, ruptured tissues increase oxygenation, favouring aerobic taxa like *Pseudomonas* and *Enterobacter*, which contribute to the degradation of complex biomolecules^10,13^. Overall, this microbial succession forms a predictable “microbial clock”^8^, enabling increasingly accurate PMI predictions using high-throughput sequencing and machine learning methods^2,14^. These models often outperform traditional approaches, especially at intermediate and late PMIs.

Environmental conditions, particularly temperature and insect accessibility, strongly affect decomposition rates and microbial activity. Higher temperatures accelerate microbial metabolism and succession; colder climates slow these processes^2,15–17^. Adjusting for temperature by using accumulated degree days (ADDs) or accumulated degree hours (ADHs), as entomologists do for insect development, helps to standardise microbial clock models across climates^14^. Regarding insects accessibility, recently it became more evident that insects can play a key role, and influence, the postmortem microbial composition and successions^2,18–20^, despite data available in literature is still limited. Soil composition also matters, especially in burial contexts, despite studies show that microbial succession in surface remains is relatively consistent across soil types^9,14,15^.

PMI estimation via microbial analyses can be achieved by sampling different anatomical locations, being skin, nose, ears and oral cavity the easiest to access. Due to the non-invasive nature of sample collection from these sites, in fact, sampling from these areas can be easily performed on the crime scene, maximizing the potential of such approach for improved PMI estimations. While several studies have been focused on the oral cavity^11,12,21–24^ and on the skin^8,11,25–28^, both from humans and from animal models, less have been focused on other anatomical areas, such as ears and nasal cavities^23,29,30^.

This study builds on a previous collaboration with the University of North Dakota, where the microbial succession of internal and external pig nostrils was investigated in an extreme cold environment over 23 weeks^17^. In that context, three pigs used as human proxies due to their anatomical and physiological similarities with humans (including *∼*80*%* gut microbiome similarity^31^) were covered with thick layers of snow for almost the full duration of the study, resulting in limited decomposition progression and restricted insect accessibility. Microbial communities shifted predictably with time, with Firmicutes and Proteobacteria being the most dominant taxa identified. Random Forest models achieved high accuracy (MAE = 1.36 weeks, RÂ² = 0.91) when both microbial and environmental data (ambient temperature and snow precipitation) were included. Cold-adapted taxa such as *Psychrobacter sp.* and *Pseudomonas sp.* were important predictors in such models. In the current work, the same study setting was applied to the temperate British summer climate. Decomposition was notably faster than in the previous study, with complete nasal skeletonisation occurring within 10 days of animal deposition, and insect colonisation observed almost immediately, with egg deposition recorded from 42 hours onward. By applying identical sampling protocols, sequencing technology (Illumina MiSeq), and statistical approaches (Random Forest, diversity analyses), we wanted to ensure direct comparability between the two studies.

The primary goal of this study was to develop a non-invasive, microbiome-based model for predicting PMI under European temperate conditions, in an outdoor setting with insect activity, to produce more regionally applicable models for real-world forensic use. A secondary goal was to compare microbial succession across the two contrasting climatic contexts, identifying both consistent and climate-specific taxa crucial for PMI prediction, and evaluating the contribution of insect microbiomes to post-mortem microbial succession.

An independent external validation of the developed model was performed using a published human facial thanatomicrobiome dataset^2^, allowing assessment of model transferability across host species, geographic regions, anatomical locations (nose versus facial skin) and inter-laboratory conditions.

## Results

**Fig. 1:**
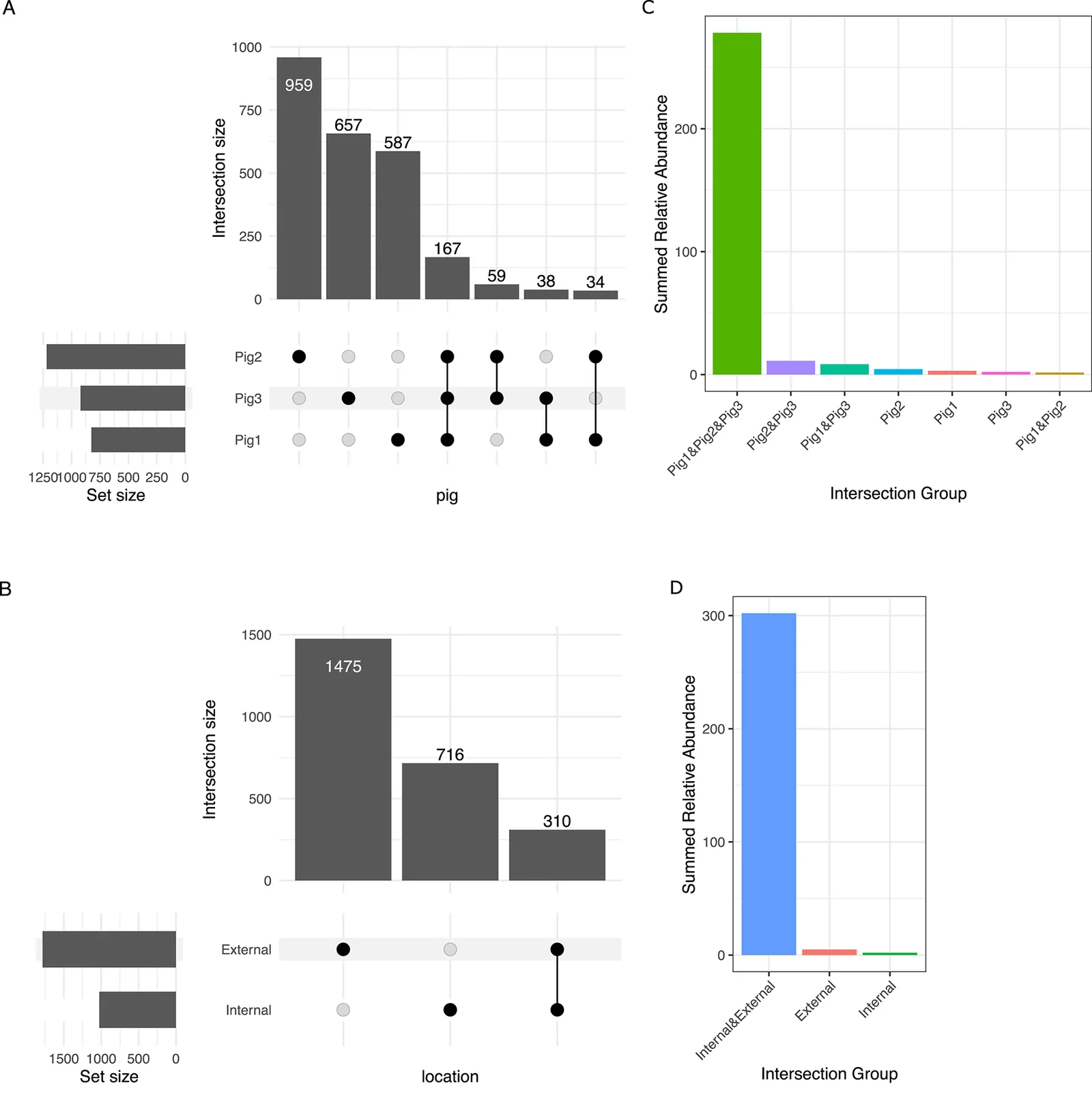
UpSet plots showing the distribution and overlap of features across individual pigs (A, C) and sample locations (B, D). (A, B) Intersection sizes and set sizes are displayed for Pig1, Pig2, and Pig3, and for internal vs. external locations, respectively. (C, D) Summed relative abundances are shown for each intersection group.

A total of 312 samples yielded 6,410,838 raw sequences, with an average of 20,547.56 reads per sample. After denoising, 3,569,328 high-quality sequences were retained. Initially, 2,651 Amplicon Sequence Variants (ASVs) were identified. ASVs identified as mitochondrial or chloroplast sequences were excluded, leaving a final count of 2,501 taxa. Before conducting formal analyses and generating figures, pre-processing steps were further applied, including the removal of samples where all ASV values were zero (total sum of intensities), reducing sample size to 309. The abundances were then normalized to the median sequencing depth, following^32^.

Temperature measurements across the experiments are reported in Supplementary Figure 1.

The comparison among the three pigs’ microbiomes returned a large number of unique ASVs for each pig, and also identified some shared taxa among them (Fig. 1A). The greatest number of intersections was found between the three pigs (167). Pig2 was the one characterized by the greatest number of ASVs (959), followed by Pig3 (657) and Pig1 (587). The set sizes for each pig are shown on the bottom left of the figure. The intersections of ASVs between internal and external nose samples are represented in Fig. 1B. The greatest number of ASVs was found in the external swabs (1475), whereas internal swabs had overall less taxa (716). The intersection between the two resulted in 310 shared ASVs. Panels 1C and 1D show the summed relative abundances for each intersection group. Shared taxa, particularly those common to all three pigs, accounted for the greatest proportion of total abundance, suggesting that unique ASVs, while numerous, largely represent rare taxa with low read counts rather than dominant community members.

### Microbial alpha and beta diversity analyses

**Fig. 2:**
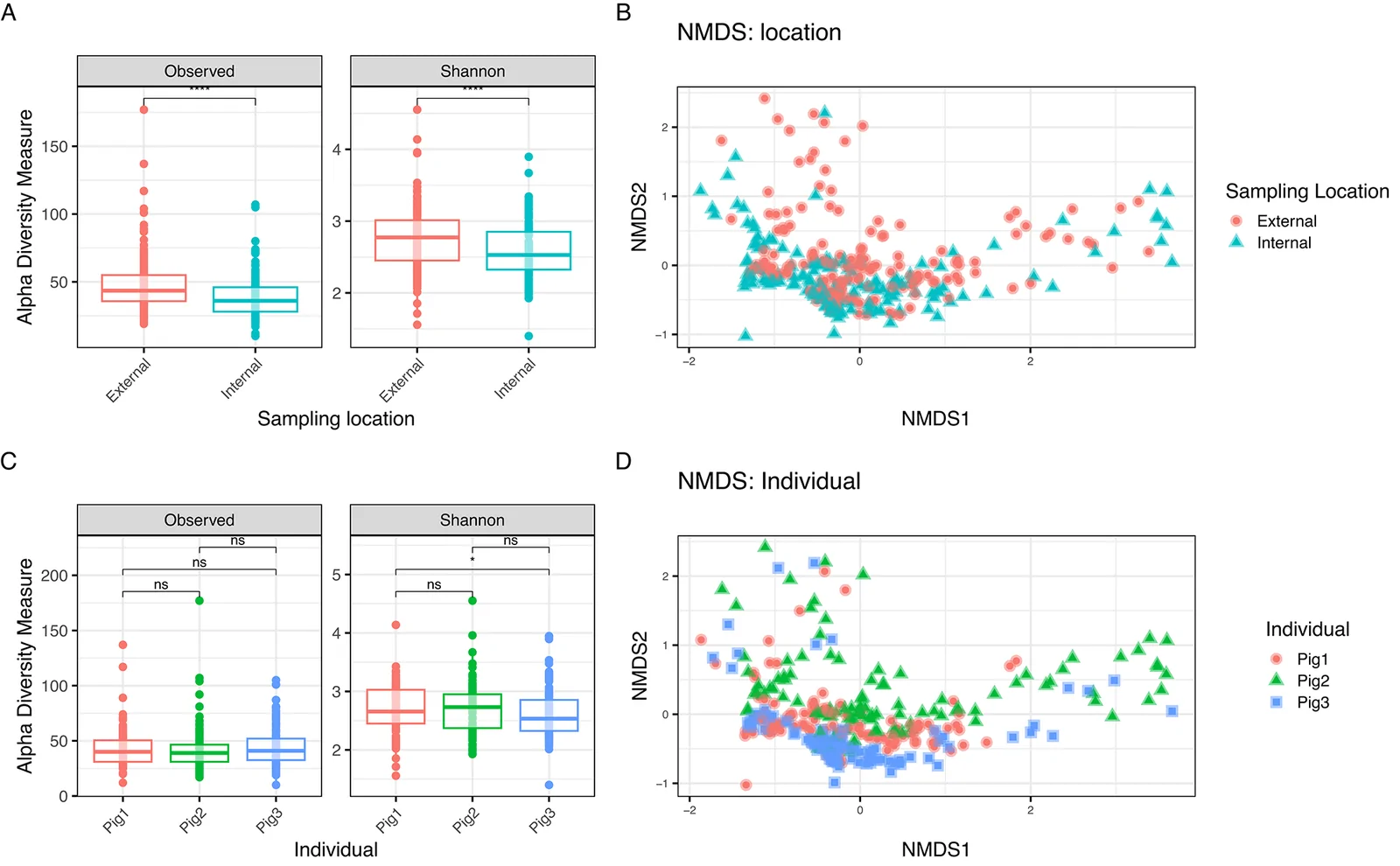
Alpha and beta diversity of microbial communities by sampling location and individual pig. (A) Alpha diversity (Observed richness and Shannon index) compared between external and internal sampling locations. (B) NMDS ordination of beta diversity by sampling location. (C) Alpha diversity metrics compared across individual pigs by sampling location, with significance indicated. (D) NMDS ordination of beta diversity by individual pig. Statistical significance is based on Wilcoxon rank-sum test (ns: p > 0.05; *: p *łe* 0.05; ****: p *łe* 0.0001).

Alpha diversity (observed richness and Shannon diversity indices) analyses of microbial communities considering both locations (exterior vs. interior) and the three individual pigs are shown in Fig. 2A and C, respectively. The observed richness and Shannon diversity indices show a significantly higher diversity in exterior samples compared to interior ones, but non-significant difference between the three pigs, with the exception of a moderate difference between Pig1 and Pig3 for Shannon index only. Non-metric Multidimensional Scaling (NMDS) plots based on beta diversity (Bray-Curtis dissimilarities) show overlaps between samples collected in different locations (internal or external, Fig. 2B), and on different animals (Fig. 2D), despite beta diversity differs significantly both by sampling location (PERMANOVA: R$^{2}$ = 0.022, F = 6.98, p = 0.001) and by pig (PERMANOVA: R$^{2}$ = 0.127, F = 22.21, p = 0.001). Overall this indicates that, despite the differences, there is a shared microbial community structure both inside and outside the nostrils, and between the three animals.

**Fig. 3:**
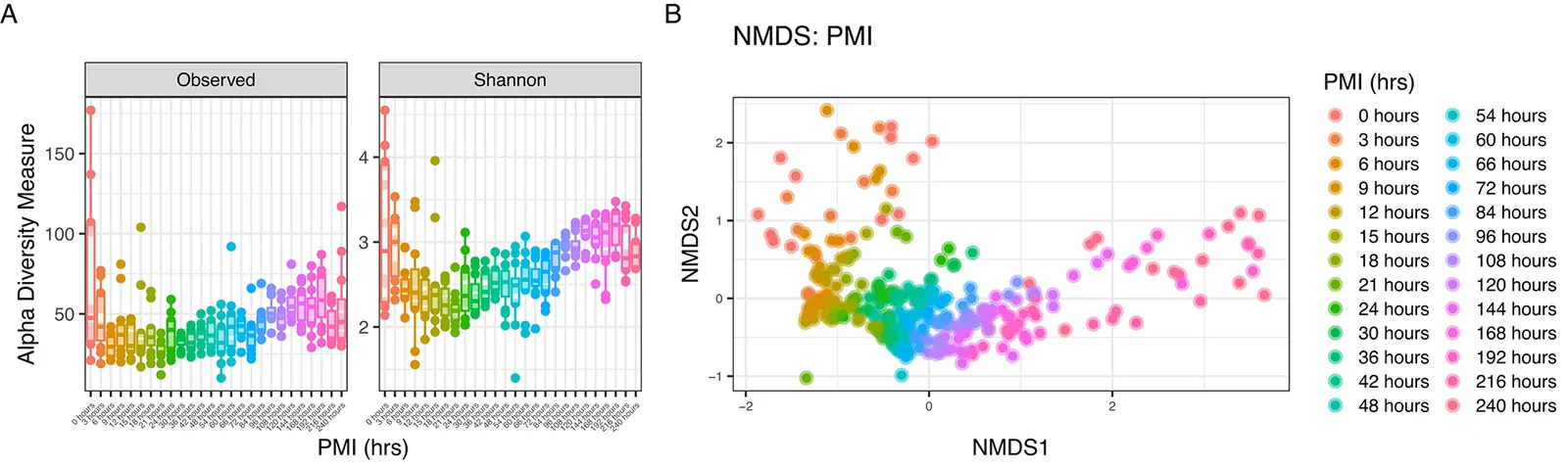
Alpha and beta diversity of microbial communities across postmortem interval (PMI). (A) Alpha diversity (Observed richness and Shannon index) at each PMI time point, ranging from 0 to 240 hours. (B) NMDS ordination of beta diversity across PMI time points.

Alpha diversity (observed richness and Shannon diversity indices) plotted against PMI (in hours) shows a non-linear trend, with relatively high diversity at early PMIs (0–6 hours), a decrease in diversity reaching its minimum at around 20 hours, an increase until around 150 hours, and a final decrease from around 168 hours to the end of the experiment, suggesting an initial drop in diversity from the death event, and an increased diversity as decomposition progressed (Fig. 3A). The NMDS plot with the microbial composition over time shows a clear temporal trajectory, with samples grouped by PMI in a roughly continuous gradient, forming a curved progression along both axes (combined variability of the data is 44.3*%*) and showing that time since death is a major driver of the observed microbial shifts (Fig. 3B). A PERMANOVA analysis based on Bray-Curtis dissimilarity revealed a significant effect of PMI group on microbial community composition (F = 10.282, R$^{2}$ = 0.476, p = 0.001). The model explained approximately 47.6*%* of the total variance in the dataset.

**Fig. 4:**
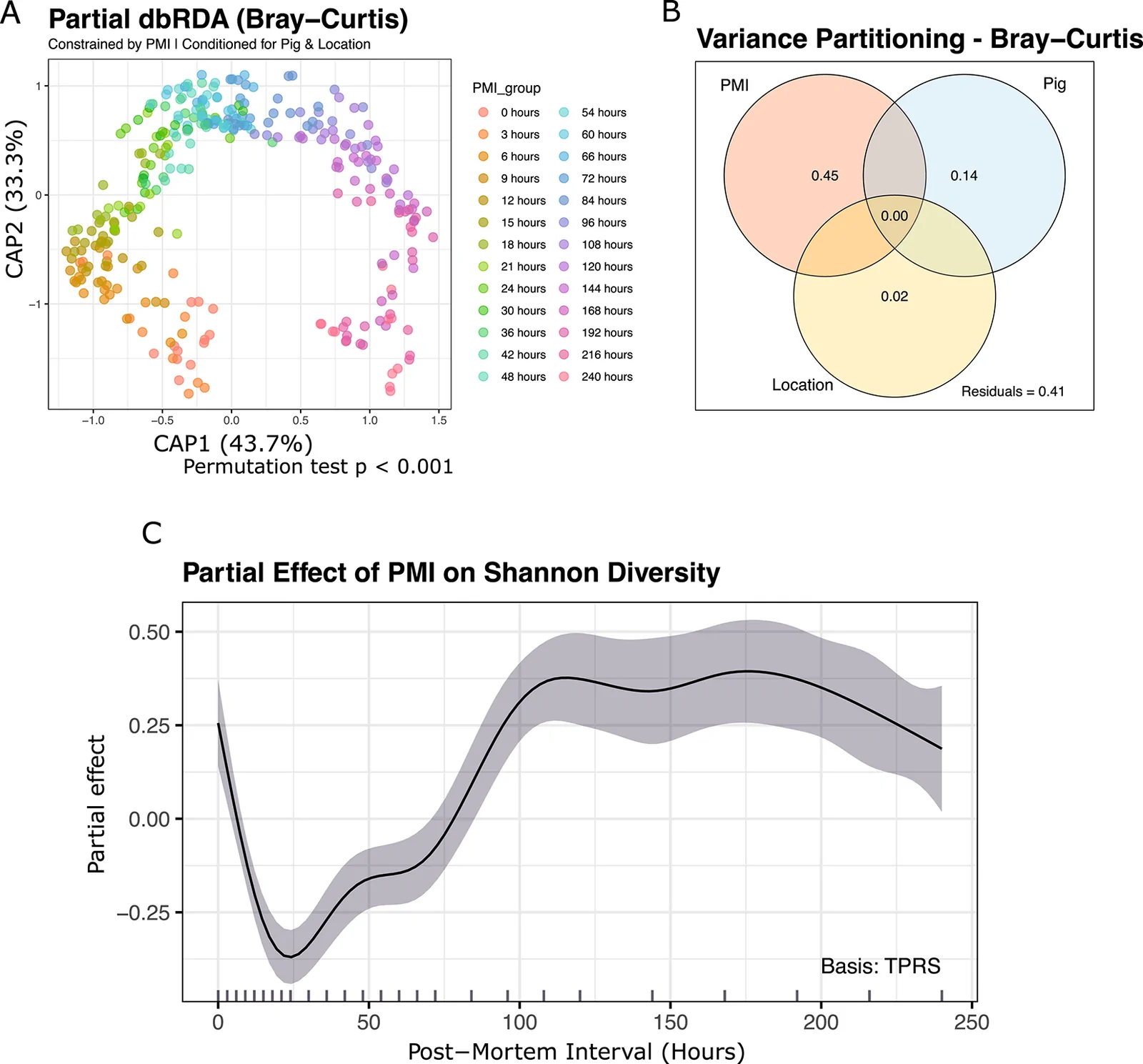
Multivariate and diversity analyses of microbial community composition in relation to PMI, pig individual, and sampling location. (A) Partial distance-based redundancy analysis (dbRDA) ordination (Bray–Curtis dissimilarity) constrained by PMI and conditioned for pig and location, colored by PMI group (0–240 hours); permutation test p *łe* 0.001. (B) Variance partitioning Venn diagram showing the proportion of Bray–Curtis dissimilarity explained by PMI (0.45), pig (0.14), and location (0.02), with residuals of 0.41. (C) Partial effect of PMI on Shannon diversity estimated by a generalized additive model (thin plate regression spline basis), with 95*%* confidence interval shaded.

To isolate the contribution of PMI from inter-individual and spatial variation, partial dbRDA was performed on Bray–Curtis dissimilarities constrained by PMI and conditioned for pig individual and sampling location. The ordination revealed a structured trajectory across PMI time points along CAP1 (43.7*%* of variance) and CAP2 (33.3*%* of variance), with a permutation test confirming a significant effect of PMI on community composition (p *łe* 0.001; Fig. 4A). Variance partitioning further quantified the relative contributions of each factor to microbial community turnover. PMI accounted for the largest proportion of explained variance (0.45), followed by pig individual (0.14) and sampling location (0.02), with no detectable shared variance among the three factors (0.00). Residual variance was 0.41 (4B). The partial effect of PMI on Shannon diversity, modelled using a generalized additive model with a thin plate regression spline basis, revealed a non-linear relationship over the 240-hour postmortem period. Shannon diversity declined in the early postmortem period, reaching a minimum at approximately 20–30 hours, before increasing and stabilising between approximately 100 and 200 hours PMI, followed by a subsequent decline. The 95*%* confidence interval widened at later time points, reflecting increased variability at extended PMIs (4C).

### Microbial taxonomic diversity and abundances

**Fig. 5:**
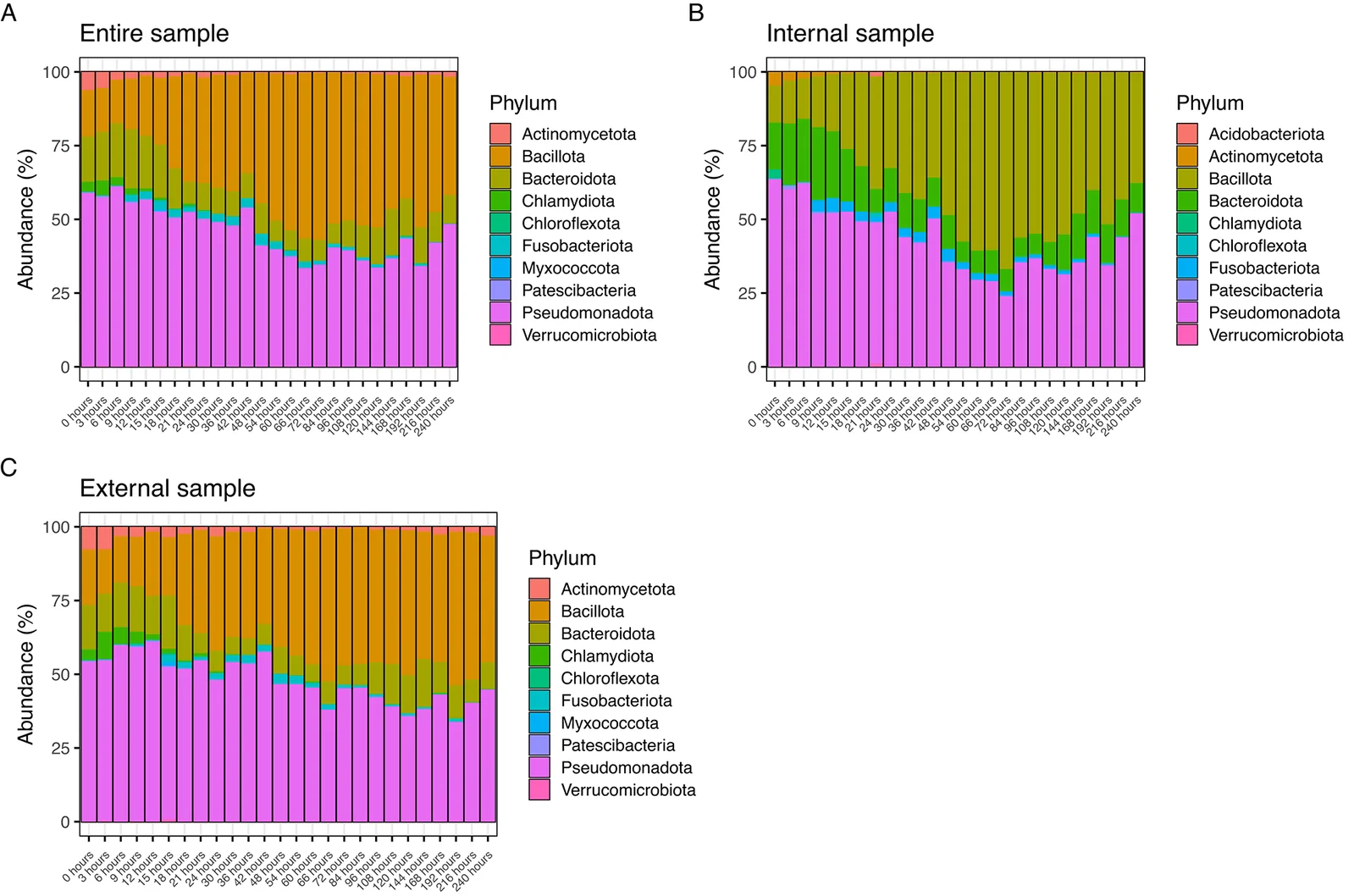
Bacterial community relative abundances (*%*) at phylum level (top 10 phyla are represented) for (A) entire, (B) internal, and (C) external sample across the various PMIs.

Bacterial communities at phylum level were represented, over time, for both the “entire sample” (internal and external swabs, Fig. 5A), and for internal and external swabs only (Fig. 5B and C). Additionally, bar plots at class level are reported in Supplementary Figure 2. In the “entire sample”, at early PMIs, Pseudomonadota is the predominant phylum in the bacterial community (*∼*60*%*) for the first 48 hours. Pseudomonadota relative abundance decreases consistently over increasing time points, to reach its minimum around 66 hours (33.50*%*) to then increase again for the remaining time points. The other abundant phylum in early PMIs is Bacteroidota (with a maximum of 20.08*%* at 9 hours), that gradually decreases at 18 hours and increases again at 108 hours. In contrast, Bacillota, the third most abundant phylum at the beginning of the experiment, becomes the dominant one between 54 and 192 hours and persists at high abundances until the end of the experiment (*∼*240 hours). The samples taken from the internal nasal cavity are characterised by a very similar trend, however Pseudomonadota phylum shows, in this case, a more marked decrease up to 72 hours (24*%*), before starting to increase again. At the same time, Bacillota becomes the most abundant phylum from 48 hours onward. The other abundant phylum is Bacteroidota, that follows a similar pattern of Pseudomonadota. More linear seems to be the behavior of the community samples collected from the external nasal cavity, with Bacillota increasing consistently throughout the increasing PMIs and Pseudomonadota starting to decrease from 48 hours onward.

### PMI modelling with random forest and best predictors

**Fig. 6:**
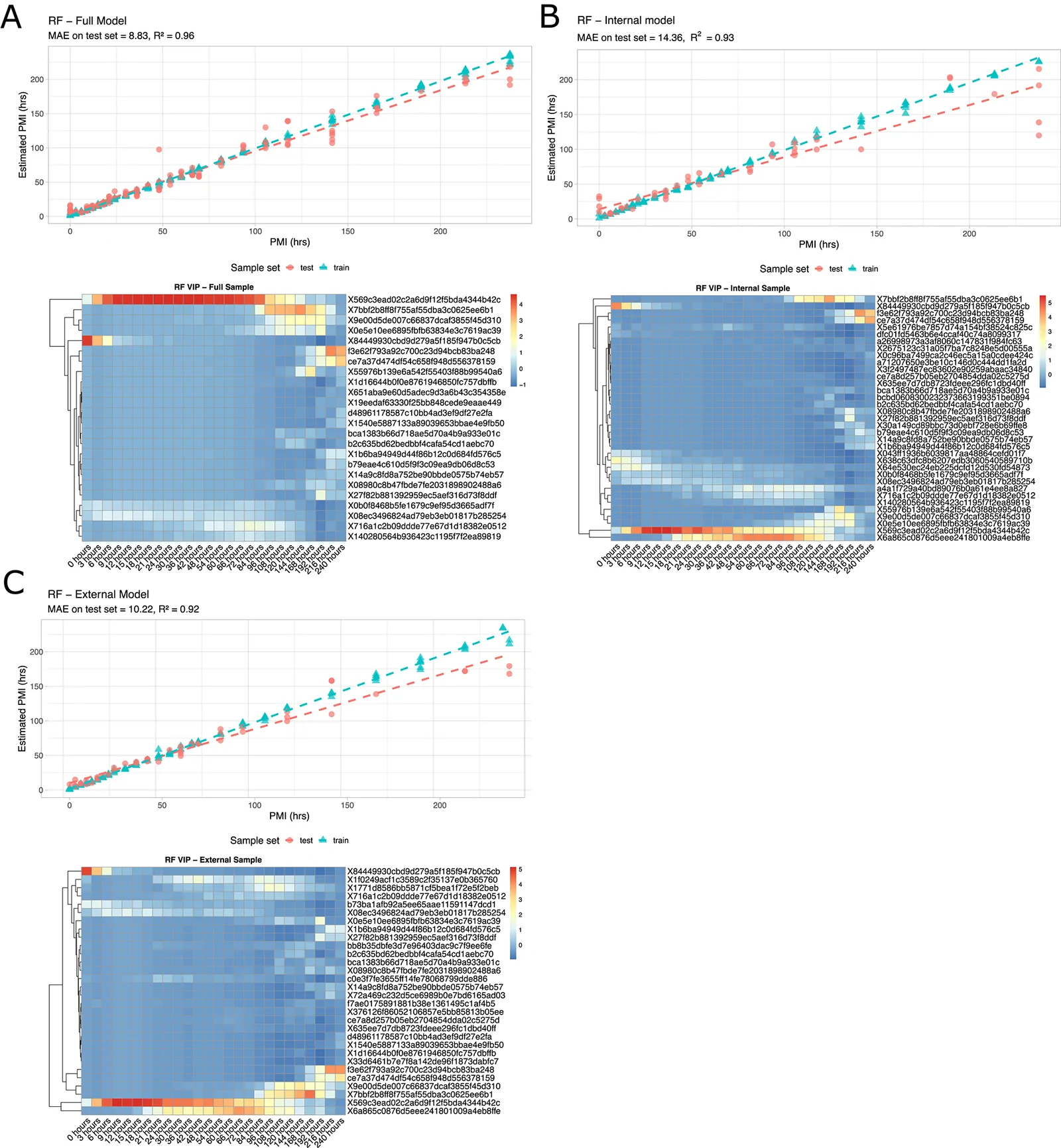
Random forest (RF) models for PMI estimation based on microbial community composition at ASV level. Scatter plots of actual vs. estimated PMI (hours) for train and test sample sets, with mean absolute error (MAE) and coefficient of determination (R$^{2}$), developed on (A) samples pooled from both external and internal nose (MAE = 8.83, R$^{2}$ = 0.96), (B) samples obtained only from the internal nose (MAE = 14.36, R$^{2}$ = 0.93), and (C) samples obtained only from the external nose (MAE = 10.22, R$^{2}$ = 0.92). Heatmaps showing the top variable importance in projection (VIP) features and their normalized abundance across PMI time points are reported for samples pooled from both external and internal nose, samples obtained only from the internal nose, and samples obtained only from the external nose, respectively, below their models.

Random Forest was applied to the entire dataset to estimate PMI using the nasal microbial data. The model developed by pooling together samples collected from both the internal and external nose was the best performing one, providing an error of 8.83 hours with R$^{2}$ of 0.96, whereas the one developed using only the internal samples provided an error of 14.36 hours (R$^{2}$ of 0.92) and the one using the external samples gave an error of 10.22 hours (R$^{2}$ of 0.92) (Fig. 6A). This indicates slightly better accuracy of the external model compared to the internal model when only one sample type is used. It is worth noticing the considerable reduction in estimation accuracy after the first 125 hours, with a slight underestimation towards more prolonged PMIs. Nonetheless, both the training and test datasets showed close alignment with the best fit, reflecting the models’ robustness. To promote inter-study comparisons, we also converted our data into ADDs and ADHs (instead of using PMI in hours) and recalculated the models. The study duration was 3453.3 ADH (143.89 ADD). The model combining internal and external swabs obtained an R$^{2}$ of 0.96, RMSE of 198.05 ADH (corresponding to 8.25 ADD) and MAE of 117.87 ADH (corresponding to 4.91 ADD). Similarly, the internal model yielded an R$^{2}$ of 0.93, RMSE of 204.06 (corresponding to 8.50 ADD) and MAE of 142.66 (corresponding to 5.94 ADD). Lastly, the external model obtained an R$^{2}$ of 0.92, RMSE of 227.35 (corresponding to 9.47 ADD), and MAE of 151.52 (corresponding to 6.31 ADD) (Fig. 6).

**Fig. 7:**
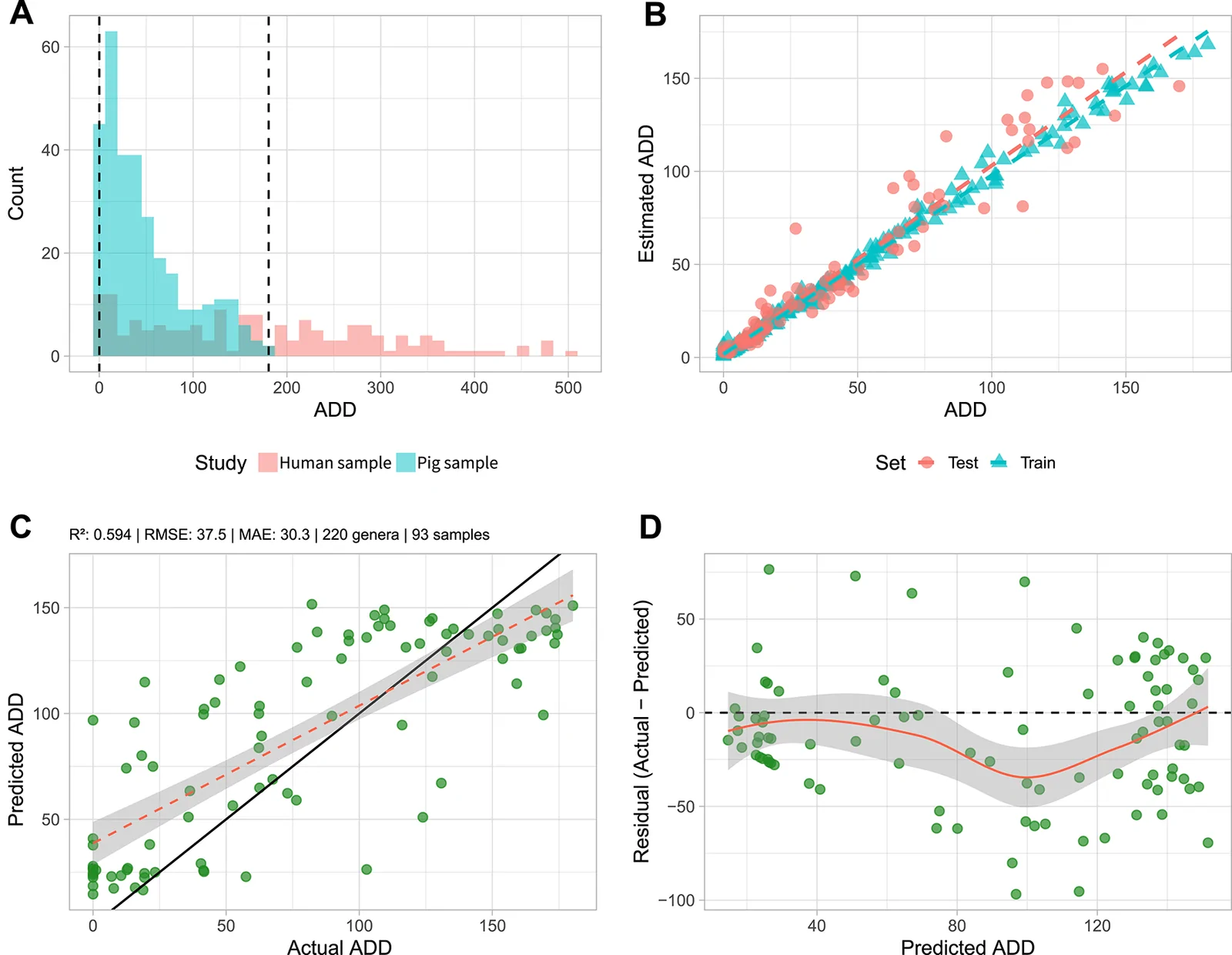
Genus-level random forest model trained on current pig dataset and externally validated on the Burcham et al. 2024 dataset (human skin face samples, ADD-matched). (A) Distribution of accumulated degree days (ADD) in the pig training and test dataset at genus-level. (B) Internal genus-level model performance on the pig dataset (train/test split, 70/30). (C) External validation of the genus-level pig random forest model on ADD-matched human external dataset. (D) Residual plot for the external validation set.

Among the best ASVs predictors for PMI estimation in the developed models are ASV1172 and ASV1064, two Gammaproteobacteria identified as *Ignatzschineria* and as *Pasteurella*, respectively. *Ignatzschineria* appears at 144 hours and increases consistently until the end of the experiment (Fig. 6). In contrast, *Pasteurella* appears and increases steadily from the beginning of the experiment, reaching its peak at 15 hours, after which it gradually decreases across the remaining time intervals (Fig. 6). Similarly to ASV1172, ASV1173 and ASV1176 (belonging to the same *Ignatzschineria* genus) has a similar trend of being low in relative abundance until late decomposition (Fig. 6). Instead ASV1075, a Pseudomonadales of the genus *Moraxella*, has a relatively high abundance at the very early PMI stages, but the abundance drops from 6 hours and decreases consistently over the course of decomposition (Fig. 6). ASV1770, Bacillota of the genus *Clostridium*, shows similar trends to ASV1705 (Planococcaceae family, *Kurthia* genus) and ASV557 (Bacteroidota belonging to the *Myroides* genus), with constant increases from around 66–84 hours until approximately 192–216 hours (Fig. 6). Among the other markers selected in all the models, it is important to mention ASV1692, a Bacillota belonging to the Planococcaceae family, and ASV1719 and ASV1720, two Bacillota of the same family and of *Savagea* genus. They both increase in abundance at PMIs over 108 hours and 168 hours, respectively, suggesting their potential for estimation of PMIs also for longer time periods than those covered in this study (Fig. 6). Other potential markers for longer PMIs include ASV1327 and ASV1330 (both Bacillota identified as *Erysipelothrix* genus), ASV1541 (Bacillota identified as *Helcococcus* genus), ASV1461 (Clostridia of the *Tissierella* genus) and ASV562 (another Bacteroidota of the genus *Myroides*) (Fig. 6). Abundance changes over 240 hours are reported for each ASV in the Supplementary Figure 3.

To assess the generalizability of the genus-level random forest model, we performed an external validation using skin face microbiome samples from the^2^ dataset (Fig. 7). The Random Forest model developed at the genus-level instead of at ASV level, showed a robust internal performance using the current pig data as a training data set, with an out-of-bag R$^{2}$ of 0.938 and a RMSE of 11.6 ADDs, indicating that the model explained more than 93*%* of the ADD variance during training. On the held-out current pig data used as test set (30*%* of samples), the model maintained a consistent accuracy with an RMSE of 11.8 ADDs and MAE of 7.87 ADDs, confirming the absence of substantial overfitting. (Fig 7B. 6B). When applied to the human independent cohort, the model developed on pigs at genus level achieved an R$^{2}$ of 0.594, RMSE of 37.5 ADDs, and MAE of 30.3 ADDs (Fig. 7C6C), indicating that approximately 59*%* of the variance in the PMI could be explained by the microbial composition at genus-level, despite the differences in geographic location, host species, anatomical location (nose versus facial skin) for the sampling and sequencing runs between datasets. Residual analysis revealed non-uniform prediction error across the post-mortem interval, with the greatest deviation from zero observed at intermediate ADD values (Fig. 7D 6D), suggesting that the early and mid decomposition dynamics, potentially influenced by the composition of the ante-mortem microbiome or site-specific environmental factors, are not fully captured by this current model.

## Discussion

This study successfully developed a non-invasive, microbiome-based model for PMI estimation under European temperate conditions, achieving high predictive accuracy and confirming the utility of this approach for regionally applicable forensic casework. Comparison of microbial succession across the two climatically distinct settings enabled identification of both climate-consistent and climate-specific taxa, and shed light on the contribution of insect activity to post-mortem microbial succession.

Although cross-climatic validation against the North Dakota dataset was precluded by the limited number of samples available at comparable accumulated degree hours (ADHs), external validation on the^2^ human thanatomicrobiome dataset demonstrated robust model transferability across host species, geographic regions, and interlaboratory conditions, while highlighting that predictive accuracy was optimised when models were trained on locally derived data.

### Postmortem microbiome composition and diversity

#### Community composition over decomposition

The swine carcass post-mortem microbiomes were highly diverse, varied among carcasses, and had relatively few shared ASVs among them, despite their general overlap on the NMDSs. The exterior microbiomes were also considerably more diverse at the ASV level compared to interior communities, potentially reflecting the higher variability of the skin microbiome versus the internal nasal one^33^, likely augmented by environmental sources such as flies. This level of ASV diversity is comparable to other studies of swine carcass microbiomes and consistent with the findings of^17^.

Decomposition, the process by which organic matter is broken down into smaller components, is driven by a community of microbial decomposers whose dynamics and functional roles are becoming increasingly well understood^2,34^. Microbial communities have been reported to shift significantly after approximately 48 hours post-mortem^23^, evolving into a more consistent profile referred to as the thanatomicrobiome or epinecrotic microbiome^12,35^, which differs markedly from the microbiome associated with the *in vivo* condition^23^. This post-mortem profile becomes highly reproducible across individuals, and even across species regardless of their initial microbiomes, thereby supporting the use of microbiome analyses for PMI estimation^8,23,27^.

Inter-individual differences are nevertheless widely documented in swine microbiome studies, with biological variation in the gut microbiome associated with age, sex, stress levels, antibiotic intake, and shifts in dietary composition^36,37^. Comparable inter-individual variability has been reported in human microbial studies, reflecting variables such as ante-mortem health conditions, sex, diet, drug intake, and geographic factors^23,38^. At the nasal level specifically, inter-individual differences in humans have been linked to lifestyle factors such as smoking and the presence of sinus infections^39^, suggesting that the nasal microbiome may be particularly sensitive to host-specific conditions both ante- and post-mortem. These inter-individual differences also underpin the use of microbiome analyses in human identification and cause-of-death investigations during early post-mortem stages^23,40–43^. Despite this variability, the composition of nasal bacterial communities at the phylum level identified in this study is consistent with that reported by^29^ on human cadavers, highlighting inter-species similarities in the thanatomicrobiome and supporting the use of animal models as proxies in forensic microbiome research.

In outdoor settings, it has become increasingly evident that post-mortem microbial succession is further shaped by the activity of necrophagous insects, whose colonisation introduces additional microbial communities that interact with and modify the host thanatomicrobiome^2,18–20^; the contribution of insects to post-mortem microbial succession is therefore given particular emphasis in this study.

#### Alpha and beta diversity

Within this study, alpha diversity (both observed richness and Shannon diversity indices) exhibited a non-monotonic pattern over time, with an initial decline, a subsequent increase peaking around the mid-phase of decomposition, and a slight decrease or stabilization at later timepoints (Fig. 3). An increase in the nasal microbiome diversity with increasing PMIs was also reported by^30^, but different from^17^ where diversity decreased constantly with increasing PMIs. The increasing abundance of Bacillota (Firmicutes) observed with the progression of decomposition, from around 15 hours onward, is in line with previous publications; specifically, this is associated with a decrease in Actinomycetota (Actinobacteria) and Bacteroidota (Bacteroidetes). Pseudomonadota (Proteobacteria) showed an initial stability up to around 48 hours, after which they decreased, before increasing again from approximately 84 hours. Comparing with the North Dakota study, the main differences concern Bacillota, which decreased over increasing PMIs in^17^, and Pseudomonadota, which increased consistently in^17^.

It is important to highlight that different phylum-level trends with increasing PMIs were reported by^29^. Pseudomonadota were reported to increase up to 5 days and then decrease from approximately 5 to 10 days, whereas in the present study the trend is almost opposite. Bacteroidota decreased with increasing PMIs in both studies. Bacillota, however, which^29^ reported as decreasing for approximately 10 days, showed a notable increase with prolonged PMIs here. The study of^29^ differs substantially from ours in several respects: some cadavers (n=23) had been previously frozen, deaths occurred across different seasons and with various causes, samples were not collected longitudinally, and the geographic location differed from the UK. These discrepancies likely reflect the methodological and contextual differences outlined above, and highlight the value of longitudinal datasets for cross-study comparisons—as explored in the external validation component of this study^2^.

### Random forest model performance

This study provides the first minimally invasive, microbiome-based PMI prediction model—via nasal swabs—applicable to northwestern and central European climatic conditions, addressing a gap in regionally specific decomposition data under temperate environments. Among studies focused on accessible and non-invasive sampling sites^30^, sampled bacterial populations from both ear and nasal canals of 21 human cadavers (144 samples total), applying machine learning models for PMI estimation. Their model predicted PMI with an accuracy of 55 ADDs up to approximately 450 ADDs, and found that nasal samples alone performed poorly, whereas combining nasal and ear samples yielded successful models^2^. collected non-invasive skin swabs and soil samples from 36 human cadavers across three human taphonomy facilities (HTFs) over 2016–2017, spanning all four seasons. Their best model, based on facial skin 16S rRNA data, predicted PMI with an accuracy of 26.47 ADDs for up to 558 ADDs. More recently^29^, sampled the nose and oral cavity of 34 cadavers and reported that combining both anatomical locations yielded the best Random Forest model, with a MAE of 2.26 days at genus level.

In the present study, both internal and external nasal samples yielded robust Random Forest models, consistent with^17^. As reported by^30^ and^29^, combining distinct anatomical locations outperformed models built on either site alone—a finding replicated here and in^17^. We believe this improvement reflects not merely the increased sample size in combined models, but also the broader range of taxa captured across body sites, potentially including exogenous microbial contributions from necrophagous activity^2,44,45^. These diverse microbial communities provide stronger correlations with PMI, reduce noise, and enhance predictive power. Including environmental temperature did not improve model performance, in contrast with^17^. Given the minimal temperature fluctuations recorded during this study, we attribute this to the thermal homogeneity of the decomposition period rather than a general irrelevance of temperature as a predictor. This has practical implications for real casework, where environmental records are not always available.

Our model achieved a MAE of 8.83 hours over a 240-hour decomposition period (RÂ² = 0.96), representing a significant advancement over existing PMI estimation methods. The approach does not require specialised training, does not interfere with the cadaver, and can be conducted directly at the scene upon discovery. Once collected, samples must be frozen immediately or preserved using stabilisation devices such as the Copan eNAT^®^ System (Copan Italia S.P.A.), which maintain both human and microbial nucleic acids at room temperature for up to one month.

The high accuracy achieved here, relative to human cadaver studies, likely reflects the reduced inter-individual variability of pigs compared with humans—as documented in the diversity section above—combined with the environmental homogeneity of the setting^30^. The earlier North Dakota study produced similarly robust models, though with higher error values commensurate with its longer decomposition period (MAE = 9.52 days over 161 days). Multi-site studies remain essential to evaluate the reproducibility of such models^30^, and the present experiment was designed with this in mind—replicating the North Dakota protocol under UK conditions to advance towards multi-centric, regionally tailored investigations, particularly in Europe where regulatory constraints have historically limited human decomposition research.

The external validation on^2^ —the largest multi-centric human thanatomicrobiome study published to date—demonstrated that key decomposer taxa involved in soft tissue breakdown are largely shared across diverse locations, climates, and seasons, regardless of host species, and that our model retains transferability beyond its original training conditions. For this validation, only samples with ADH values comparable to our study were included, as the model was built for PMIs of up to 10 days and cannot reliably extrapolate beyond this range. Validation was performed at genus rather than ASV level, as identical ASVs cannot be expected across independently conducted studies; relevant genera were nonetheless shared across both datasets, making genus-level comparison the more robust and appropriate basis for cross-study evaluation.

The reduction in predictive accuracy when external data were included confirms that locally derived training data remains important for optimising model performance, reinforcing the case for climate- and region-specific model development. In the absence of a locally tailored model, however, cross-validated models such as the one presented here may provide a reliable baseline for forensic PMI estimation. This is consistent with^2^, who identified temperature and insect accessibility as the key drivers of post-mortem microbial succession—both of which were accounted for in the present study. The convergence of temperature-adjusted decomposition metrics across datasets, alongside the presence of shared decomposer taxa, may partly explain why cross-dataset transferability was achieved, despite considerable differences in insect accessibility between settings.

From a statistical standpoint, integrating internal and external nasal microbiome data for PMI estimation improved accuracy, as each source represents a partially independent indicator of the decomposition process, as previously noted by^30^. The internal nasal microbiome, being relatively shielded from environmental disturbances, provides a stable signal likely linked to endogenous physiological changes, whereas the external nasal microbiome may capture colonisation by environmental and insect-associated taxa, which exhibit distinct yet complementary temporal dynamics. Within a multivariate modelling framework, each dataset contributes unique explanatory variance for PMI, thereby reducing residual error and increasing the proportion of variance explained. This represents a form of data fusion, in which predictor error terms are not perfectly correlated, allowing their combination to lower overall prediction error. As a result, the model gains improved generalisability across cases, integrating the stability of internal signals with the environmental sensitivity of external measures to produce more robust and precise PMI estimates.

### PMI indicator taxa

Given the natural environmental conditions under which carcass (or cadaver) decomposition frequently occurs, the role of necrophagous and necrophilous arthropods, as well as vertebrate scavengers, in shaping postmortem microbiome dynamics is critical to consider when deriving microbial-based PMI estimates^2,18,19,34^. It has been reported that postmortem microbiome succession is significantly different when carcass access by scavengers is eliminated or limited^44^, suggesting that these necrophagous organisms contribute exogenous microbes to the overall community^46^. Interestingly, the delayed access by insects to a carcass also affects invertebrate community succession^45^, suggesting that postmortem microbiomes and attending insects interact in ways that alter the overall decomposition process, thus likely affecting PMI estimates derived from either microbes, insects, or both^47^. Some of the more common bacterial taxa known to be associated with environmental sources such as blow flies (Diptera: Calliphoridae) and contribute to the postmortem microbiomes of cadavers are those of *Acinetobacter*, *Ignatzschineria*, *Dysgonomonas*, *Vagococcus*, and *Wohlfahrtiimonas*^48,49^. Differences in insect accessibility between the present UK study and the North Dakota experiment^17^ were considerable: in the UK, necrophagous insects colonised carcasses from the earliest post-mortem stages throughout the exposure period, whereas in North Dakota, persistent snow cover prevented insect access for several months. This fundamental difference in insect activity likely introduced distinct exogenous microbial contributions to the respective postmortem microbiomes, and may partly account for the divergent microbial succession patterns observed between the two datasets, as well as the reduction in predictive accuracy when models trained under one set of conditions were applied to the other.

The early PMI indicator features identified in this study included two members of the Pasteurellaceae family, *Glaesserella* and *Pasteurella* species respectively (ASV1062 and ASV1064), a member of *Moraxella* genus (ASV1075), two members of the *Bergeyella* genus (ASV495 and ASV501), and a *Streptococcus* species (ASV1635). Of these, the strongest indicators appeared to be the *Pasteurella* (Enterobacterales order) and *Moraxella* (Pseudomonadales order). *Pasteurella* exhibited an initial increase in abundance during early postmortem intervals (up to 24 hours), followed by a progressive decline through to 240 hours, as observed also in the oral cavity of mouse carcasses under controlled indoor laboratory conditions^22^. Both *Glaesserella* and *Pasteurella* are well-established members of the porcine nasopharyngeal microbiome with significant clinical relevance. *Glaesserella parasuis* is a commensal organism of the upper respiratory tract of healthy pigs that can act as an opportunistic pathogen, particularly in younger or immunologically naive animals, causing GlÃ¤sser’s disease, characterised by polyserositis, meningitis, and arthritis^50^. Similarly, *Pasteurella multocida* is a common commensal of the porcine nasopharynx that becomes pathogenic under conditions of stress or immunosuppression, contributing to atrophic rhinitis and enzootic pneumonia, with particular severity in previously unexposed young stock^51,52^. Instead, *Moraxella* is a common commensal genus found in the upper respiratory tract of both humans^53^ and swine^54^. Its high abundance at the onset of decomposition (time 0) is expected, and it is rapidly outcompeted by decomposer taxa.

Key indicators for late PMI were found instead to be several ASVs (ASV1156, ASV1172, ASV1173, ASV1176) of the *Ignatzschineria* genus, belonging to the Wohlfahrtiimonadaceae family, which were often very low before 144 hours, but then increased until the end of decomposition. It has been reported that members of *Ignatzschineria* occurred on the outside of adult *Phormia regina* (Meigen, 1826) (Diptera: Calliphoridae), but with less internal colonization^48^, it was reported as a shared microbiome genus of house flies and blow flies^55^ and also as a genus isolated from flesh flies (Diptera: Sarcophagidae) larvae^56^. This genus was also reported in internal larval samples of *Lucilia sericata* (Meigen, 1826) (Diptera: Calliphoridae)^49^. In addition to *Ignatzschineria*, also other members of the Wohlfahrtiimonadaceae family associated with prolonged PMIs, such as *Koukoulia* species (ASV1163, ASV1167) and *Wohlfahrtiimonas* (ASV1158), were reported to be associated with flies^2,13^, including the external body of *P. regina* (Meigen, 1826)^48^. Other species found in prolonged PMIs include *Myroides* genus (ASV557 and ASV562), *Acinetobacter* genus (ASV1041, ASV1049) and *Vagococcus* genus (ASV1653), which were also reported on the external body of *Phormia regina* (Meigen, 1826) (Diptera: Calliphoridae)^48^, in adult blowflies^57^ and in both *Lucilia sericata* (Diptera: Calliphoridae) and * Lucilia cuprina* (Wiedemann, 1830) species^55^.

Thus, *Ignatzschineria*, *Myroides*, *Koukoulia*, *Acinetobacter*, *Vagococcus* and *Wohlfahrtiimonas* may represent insect-associated markers of later decomposition PMIs when insect activity has been substantial on carcasses. In this study, larval blow flies were most obvious and active from about 120–240 h, corresponding to the increases in the above-mentioned taxa abundances and their importance in the models. Interestingly, none of these genera were reported in^17^, likely because the pig carcasses in their study were covered by snow, resulting in minimal or no insect activity, and consequently, the absence of insect-associated bacterial taxa from their microbial profiles. Other key indicators for late PMI included *Savagea* species (ASV1719, ASV1720), *Kurthia* species (ASV1705), *Clostridium* species (ASV1736 and ASV1770) and lastly, genus of the family Planococcaceae (ASV1692). Many of these, and of those mentioned in the above paragraph, such as *Savagea*, *Clostridium*, *Vagococcus*, Planococcaceae, and the abovementioned *Myroides* and *Ignatzschineria*, have been reported in association with prolonged (post-rupture) PMIs in both gravesoil and carcasses in either summer or winter conditions in China^58^, are well-known members of the decomposers network and were identified among the most important contributors for PMI estimation in the American multi-centric study mentioned before^2^, and were found to be associated with late PMIs also in other works^13,35,59^.

In contrast with^58^ where certain taxa (such as *Vagococcus*, *Myroides*, *Carnobacterium*, *Psychrobacter*, *Clostridium*, and *Campylobacter*) were reported as significant for PMI estimation under winter conditions, our study demonstrated that some of these taxa can also serve as reliable predictors in British summer conditions. Notably, similar to^17,58^ also identified *Psychrobacter*, *Carnobacterium* and *Clostridium* as key taxa for PMI prediction in cold environments, findings further corroborated by^60^, where these taxa were found associated with human cadavers that had been frozen and subsequently thawed. These recurring observations suggest that *Psychrobacter* and *Carnobacterium* may indeed be taxa with a strong affinity for cold or post-freezing conditions. Conversely, other taxa such as *Vagococcus*, *Myroides* and *Clostridium*, which we also detected in this study under temperate summer conditions, do not appear to be exclusively associated with cold environments. Instead, their presence might indicate a broader ecological adaptability, making them potentially valuable indicators for PMI estimation across a wider range of climatic contexts.

## Conclusions

The results of this study demonstrate the potential utility of the post-mortem microbiome in death investigations, particularly through the application of machine learning models capable of providing quantifiable error rates and confidence intervals. The high accuracy achieved under temperate European outdoor conditions, combined with robust external validation against a human thanatomicrobiome dataset, confirms that microbiome-based PMI estimation can generalise across host species, geographic regions, anatomical locations (nose versus facial skin) and laboratory settings. Nevertheless, locally derived training data optimises predictive accuracy, underscoring the importance of regionally specific model development—particularly for climatic contexts historically underrepresented in decomposition research, such as Europe.

The consistency of key taxa across animal proxy and human cadaver studies underscores the translational value of animal models, whose microbiome data can be effectively integrated into predictive frameworks to enhance robustness and scalability. The role of necrophagous insects in shaping post-mortem microbial succession, given particular emphasis here, further highlights the importance of accounting for entomological activity in outdoor forensic contexts.

Future directions should prioritise bringing global research groups together to combine regionally specific models and achieve the standardisation necessary to bring post-mortem microbiome science into courts of law. This requires harmonisation of experimental protocols, sequencing methodologies, and analytical pipelines across diverse climatic regions to ensure the credibility and admissibility of microbiological evidence in forensic investigations.

## Materials and methods

### Experimental design

The study was ethically approved by the University of Lancashire Ethics Committee (ref. SCIENCE 0223), and all experiments were performed in accordance with relevant guidelines and regulations. Experimental planning and reporting of the results were performed following ARRIVE guidelines.

Three pigs (*Sus scrofa domesticus*) (approx. 50 kg each, same breed, healthy, similar age) were purchased from a local pig farm and used as human analogs^25^. In accordance with ARRIVE guidelines, the study did not include control groups due to the non-invasiveness of the samplings and longitudinal nature of the study. The sample size was decided based on similar experiments (such as^17^) where the sample size and sampling frequency was shown to be adequate to achieve the intended aims. Randomisation and blinding were not required for this specific study, as it did not involve the delivery of any treatment on the animals. Pigs were humanely euthanized via captive bolt by a competent individual licensed under the Welfare of Animals at Time of Killing Regulations (England) (2015). They were placed on the ground, rough grassland, at the Taphonomic Research in Anthropology: Centre for Experimental Studies (TRACES) at the University of Lancashire, located in Burnley (UK). A tent was placed on the top of the area to minimize the rainfalls, but animals were accessible to insects throughout the study. Scavenging in TRACES is rare, and therefore extra precautions to avoid scavenging actions were not taken. The average temperature for the duration of the experiment (10 days) was +15.8$^{\circ }$C (standard deviation 3.03$^{\circ }$C), with a minimum of +10$^{\circ }$C and maximum of +25.5$^{\circ }$C. External temperatures were recorded at the beginning of each sampling time and are shown in Supplementary Figure 1.

### Sample collection

Samples were collected over a period of 240 hours according to the schedule shown in the metadata. Two samples were collected using sterile cotton swabs from the outer surface of the nose (circular swabbing for 30 seconds), and two were collected from the inner portion of both nostrils (circular swabbing for 15 seconds per nostril). The first sample was collected immediately after euthanization (time 0), and following samples were collected at the time intervals shown in metadata. The swabs were then stored in separate Eppendorf tubes, immediately placed in dry ice for quenching and transportation, and subsequently stored at −80$^{\circ }$C until further processing. 45 minutes before extraction, samples were moved to +4$^{\circ }$C before DNA extraction.

### Nucleic acid extraction

A modified version of the QIAGEN Blood and Tissue protocol was implemented for DNA extraction, as previously done in^17^. The protocol was adjusted to use twice the standard amounts of lysis buffers, specifically 360*μ*L of buffer ATL per sample and 400*μ*L of buffer AL per sample. This adjustment was necessary because the swabs were not stored in a preservation buffer, making them more prone to absorbing the lysis buffers and drying out during incubation. DNA was eluted in 50*μ*L. The samples and a positive control, in the form of *E. coli* TOP10 PLO3 D55N HOX A., were amplified using Platinum^TM^ Hot Start PCR Master Mix from Invitrogen^TM^ (Waltham, MA, USA).

### Sequencing

Library preparation and sequencing of the 16S rRNA gene’s V4 region were conducted using the Illumina MiSeq System. The V4 region of the 16S rRNA gene was amplified using the primers 515 F (5’-GTGYCAGCMGCCGCGGTAA-3’) and 806R (5’-GGACTACNVGGGTWTCTAAT-3’)^61,62^, following the Earth Microbiome Project protocol as implemented in the MiSeq SOP. This work was performed at the NU-OMICS facility of Northumbria University, Newcastle, UK. PCR amplification used the KAPA2G Robust HotStart^®^ PCR Kit (Sigma-Aldrich, St. Louis, MO, USA). The resulting PCR products were then normalized with Quant-iT^TM^ PicoGreen^TM^ dsDNA Assay Kit (Invitrogen^TM^, Waltham, MA, USA) according to manufacturer guidelines.

### Data processing and statistical analysis

Bacterial identification, by targeting and sequencing the V4 region of 16S rRNA, was performed according to the gold standards established by the Human Microbiome Project. The.FASTq raw sequencing files obtained were processed using QIIME2 software version 2024.05^63^. Quality filtering was performed with DADA2 (QIIME2 DADA2 denoise-paired)^64^, trimming reads at 250 bp for forward and 200 bp for reverse sequences, with a trim of 20 bp applied at the 5’ end of each read, as shown in ForensOMICS GitHub. Taxonomic classification was carried out with the latest release of Silva-138-99 available at Silva. The cleaned data were further analysed in R (version 4.2.3) using the ’Phyloseq’ (v1.48.0) package^65^. Contaminants such as archaea, fungi, and both positive and negative controls were excluded. Relative abundance of bacterial classes was examined across PMI time points (0–240 hours) for the entire pooled sample, internal nose samples, and external nose samples separately (Supplementary Figure 2). Data were finally processed using median normalisation. Environmental temperature was recorded throughout the decomposition period and monitored across all PMI time points (Supplementary Figure 1). Alpha diversity was measured to assess the variations between individuals and locations in the samples. To examine beta diversity and differences between these same groups, Non-metric Multidimensional Scaling (NMDS) was applied. Beta diversity differences across all conditions were assessed using PERMANOVA (adonis2, Bray–Curtis distances, 999 permutations) in the ’Vegan’ library^66^. The independent effects of PMI, pig individual, and sampling location on microbial community composition were further quantified using partial distance-based redundancy analysis (partial dbRDA, Bray–Curtis dissimilarity) and variance partitioning. The partial effect of PMI on Shannon diversity was modelled using a generalized additive model (GAM) with a regression spline basis. Model assumptions were verified through standard diagnostic plots, including Q-Q plots of deviance residuals, residuals vs. linear predictor, histogram of residuals, and response vs. fitted values. For univariate approaches, statistical significance was calculated using Wilcoxon rank-sum test with significance set at *α* *łe* 0.05. The random forest (RF) regression algorithm, as implemented in the ’ranger’ (v0.15.1) package^67^, was used to model PMI using three sample sets: pooled external and internal nose samples, internal nose samples only, and external nose samples only. The primary RF models were trained on ASV-level data. The dataset was split into a training set (70%) and a testing set (30%), ensuring balanced PMI distributions across the two sets. Hyperparameter tuning was conducted using combinations resulting from a search grid aimed to reduce residual squared mean error of the estimations. The generated models were subsequently applied to estimate the post-mortem interval (PMI) on the validation dataset. To evaluate and compare the performance of the models, root mean squared error (RMSE), mean absolute error (MAE), and coefficient of determination (R$^{2}$) were used. Variable importance scores were calculated using a permutation-based approach as implemented in the ’ranger’ package to identify the ASVs with the greatest predictive ability (VIPs). For each variable, the out-of-bag (OOB) prediction error is first recorded, then the values of that variable are randomly permuted across all OOB samples and the increase in prediction error (RMSE) is measured. Variables causing the greatest increase in error upon permutation are ranked as the most important predictors of PMI. This permutation is repeated for each tree in the forest and the scores are averaged across all trees to produce a stable importance ranking. Data visualisation was achieved using ’ggplot2’ (v3.5.1)^68^, ’UpSetR’ (v1.4.0)^69^, and ’pheatmap’ (v1.0.12)^70^.

As a proof of concept, to asses generalisability of models based on microbial succession for PMI estimation, a random forest regression model was trained on the current pig dataset to predict accumulated degree days (ADD) from genus-level microbial composition. Raw ASV counts were agglomerated to genus level using taxonomic classification against the SILVA database, and both datasets were normalized by median-ratio scaling using the pig dataset’s median as reference. Only genera detected in both datasets were retained as features (n = 220). The model was trained on 70*%* of the pig samples (stratified by ADD) and internally validated on the remaining 30*%*, using 500 trees, mtry = 33*%*of features, minimum node size of 3, and a sampling fraction of 0.7 without replacement. For external validation^2^, dataset samples were restricted to facial skin sites and filtered to the ADD range observed in the current pig study to ensure thermal comparability across datasets. A genus-level model was developed exclusively for the purpose of external validation. The trained model was applied directly to the aligned external validation feature matrix without retraining. Model performance was assessed using R$^{2}$, root mean squared error (RMSE), and mean absolute error (MAE). Residual analysis was performed to evaluate systematic prediction bias across the ADD range.

## Supplementary Information

Below is the link to the electronic supplementary material.

Supplementary material 1

Supplementary material 2

Supplementary material 3

Supplementary material 4

Supplementary material 5

## Data Availability

The datasets presented in this study can be found in the NCBI SRA database under BioProject accession number PRJNA1294906. Link to access the data is: PRJNA1294906. Details on data analysis, scripts and full taxonomy table are available at ForensOMICS GitHub.
