# Supplementary material for: Microbiome modelling for post-mortem interval estimation across species and climates: swine analogues in a British summer and external validation in human donors from the USA
Source: Sci Rep. 2026 Aug 19;16:24916. doi: 10.1038/s41598-026-65366-y (PMC13490378; doi:10.1038/s41598-026-65366-y)

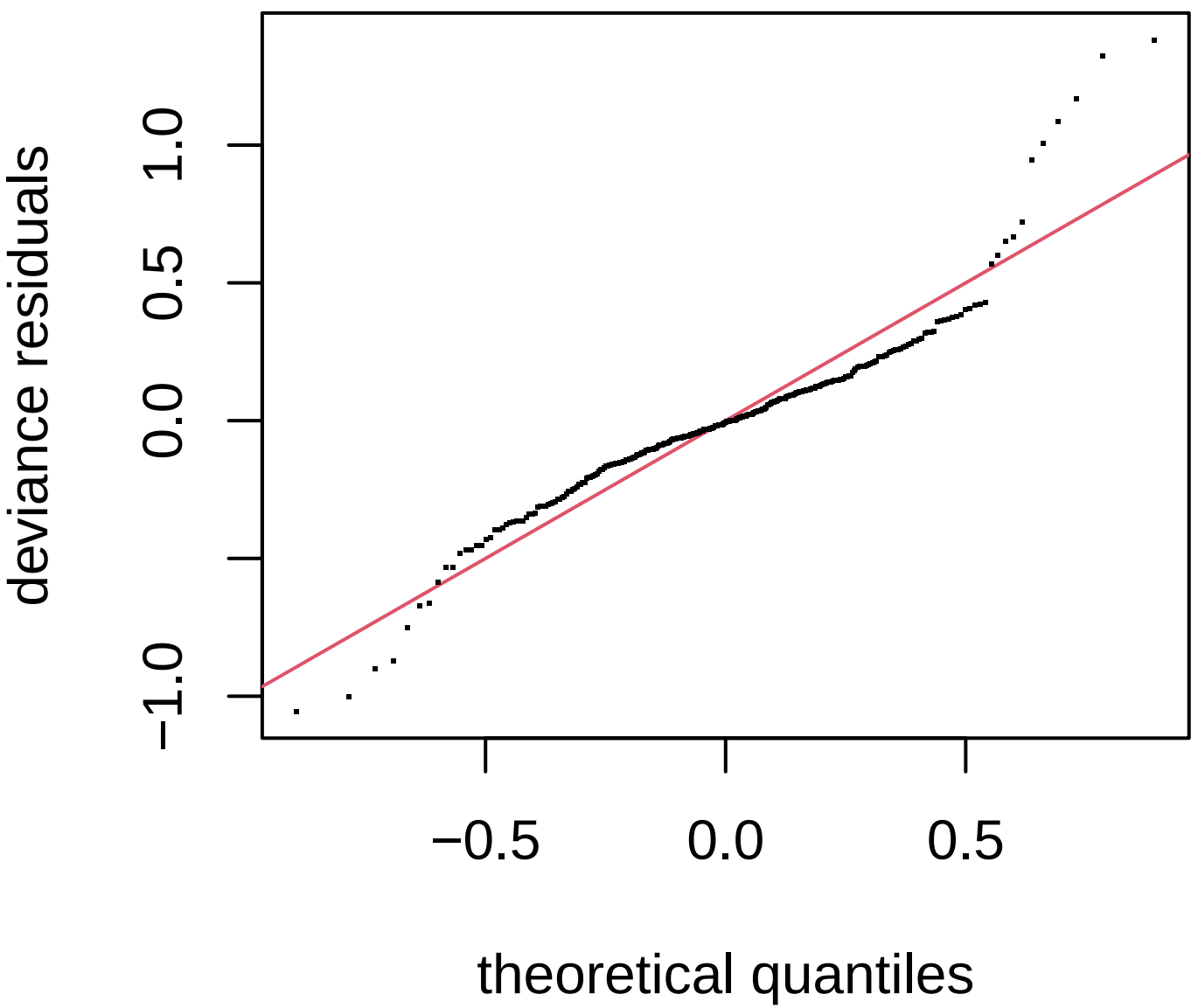

deviance residuals
−1.0
0.0
0.5
1.0
−0.5
0.0
0.5
theoretical quantiles

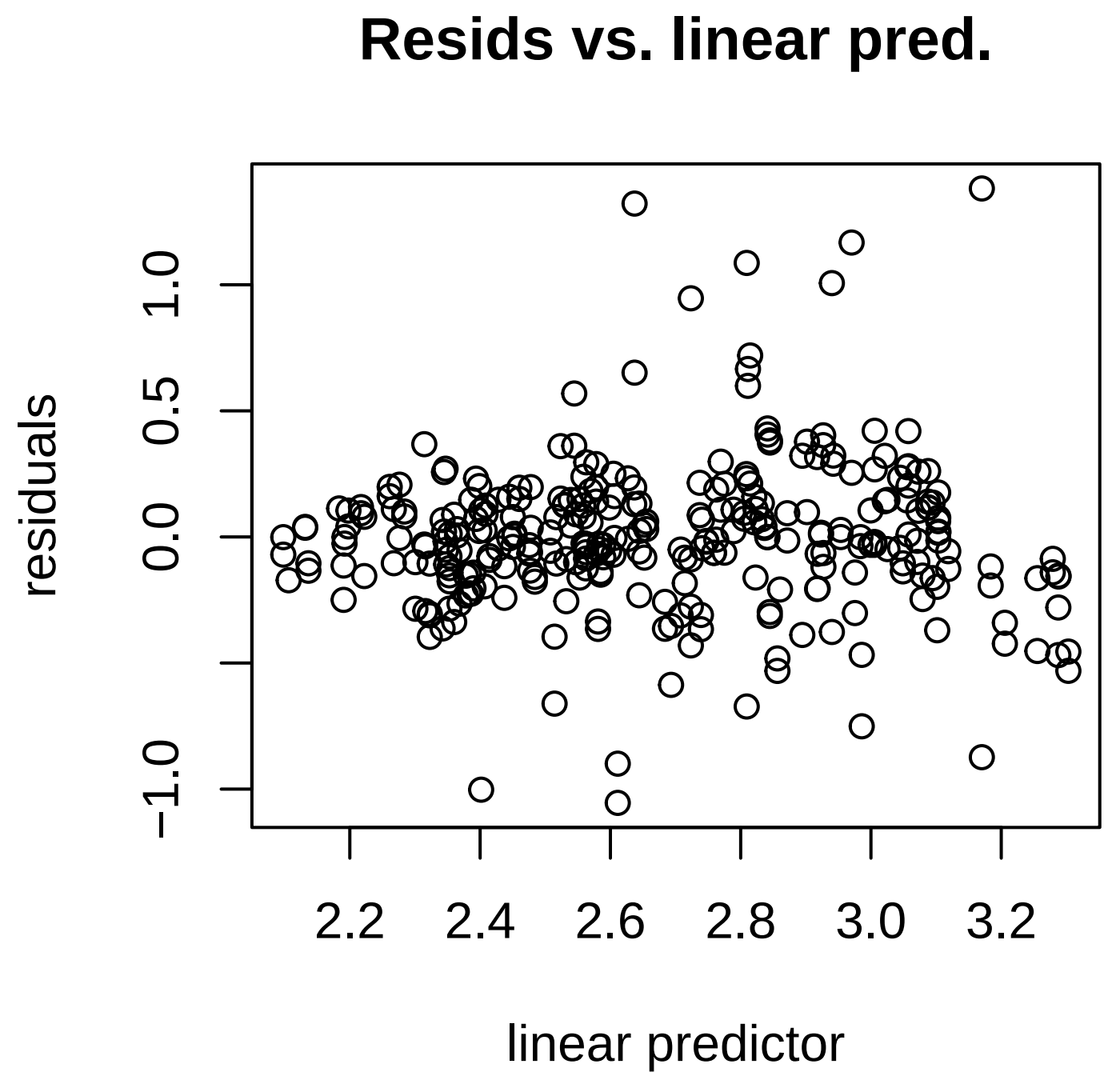
Resids vs. linear pred.
residuals
−1.0
0.0
0.5
1.0
2.2
2.4
2.6
2.8
3.0
3.2
linear predictor

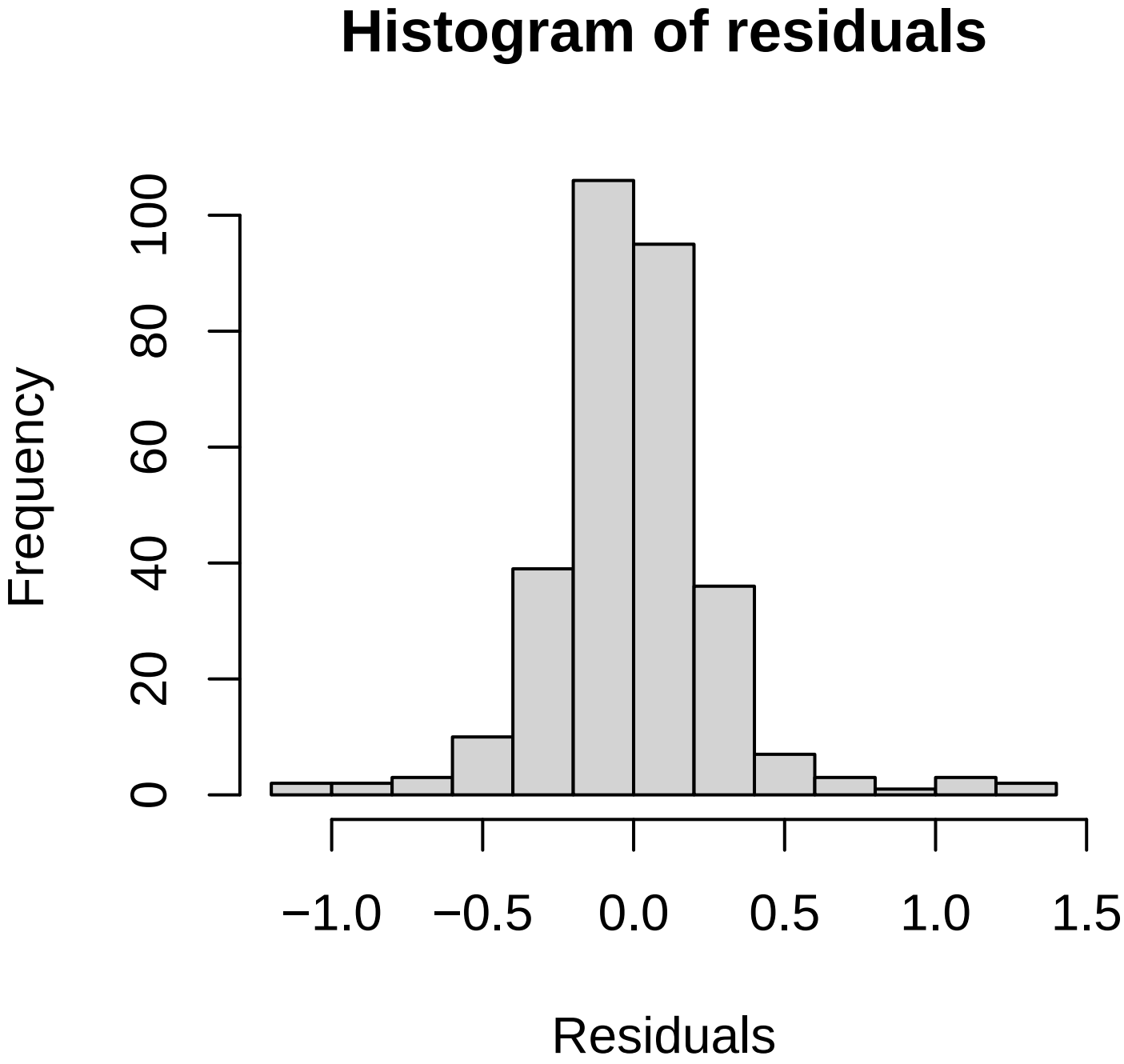
Histogram of residuals
Frequency
0
20
40
60
80
100
−1.0
−0.5
0.0
0.5
1.0
1.5
Residuals

Response vs. Fitted Values

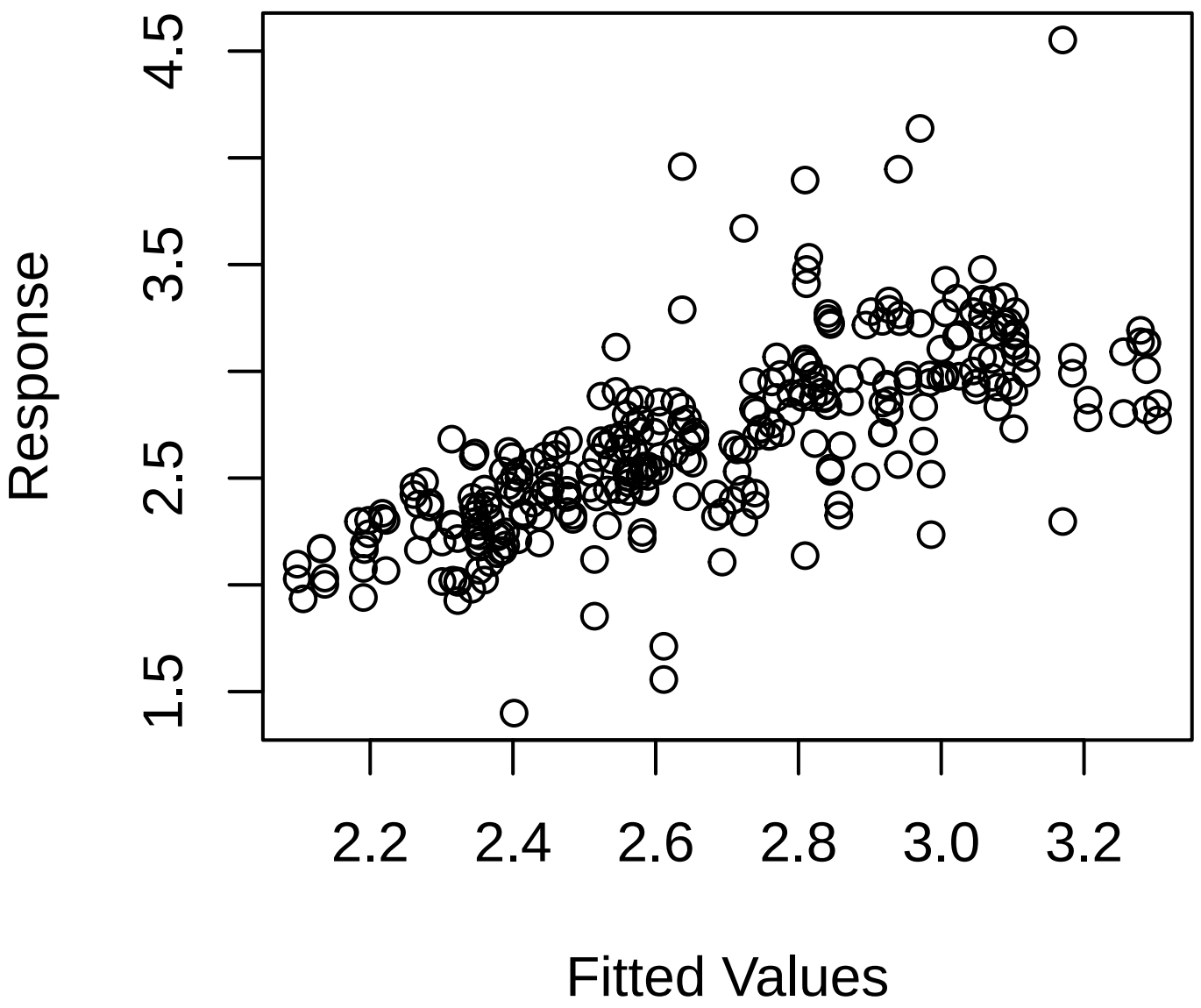

Supplement: Supplementary file 1 [file 41598_2026_65366_MOESM2_ESM.pdf]
