# Supplementary material for: Microbiome modelling for post-mortem interval estimation across species and climates: swine analogues in a British summer and external validation in human donors from the USA
Source: Sci Rep. 2026 Aug 19;16:24916. doi: 10.1038/s41598-026-65366-y (PMC13490378; doi:10.1038/s41598-026-65366-y)
Supplement: Supplementary file 2 [file 41598_2026_65366_MOESM3_ESM.pdf]

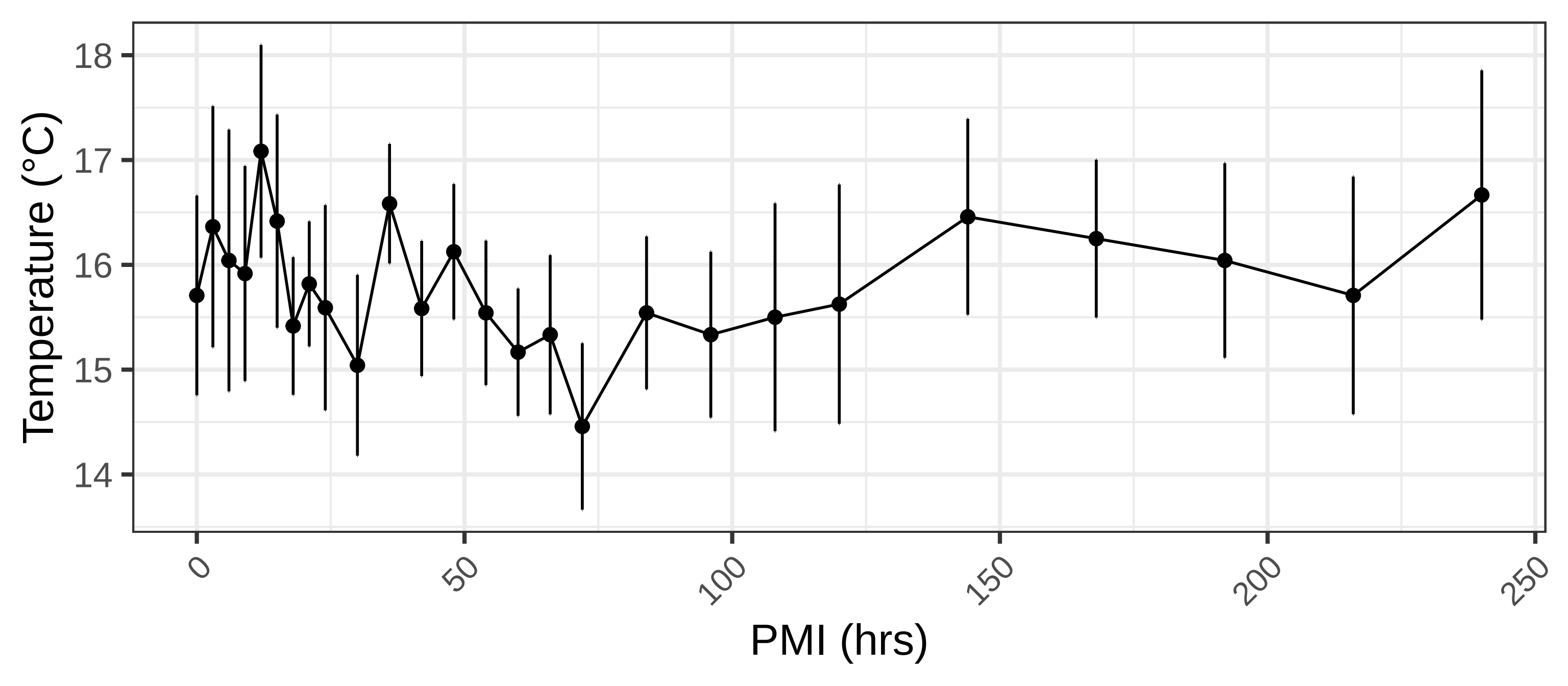

Temperature (°C)
18
17
16
15
14
0
50
100
150
200
250
PMI (hrs)
