# Supplementary material for: Microbiome modelling for post-mortem interval estimation across species and climates: swine analogues in a British summer and external validation in human donors from the USA
Source: Sci Rep. 2026 Aug 19;16:24916. doi: 10.1038/s41598-026-65366-y (PMC13490378; doi:10.1038/s41598-026-65366-y)
Supplement: Supplementary file 3 [file 41598_2026_65366_MOESM4_ESM.pdf]

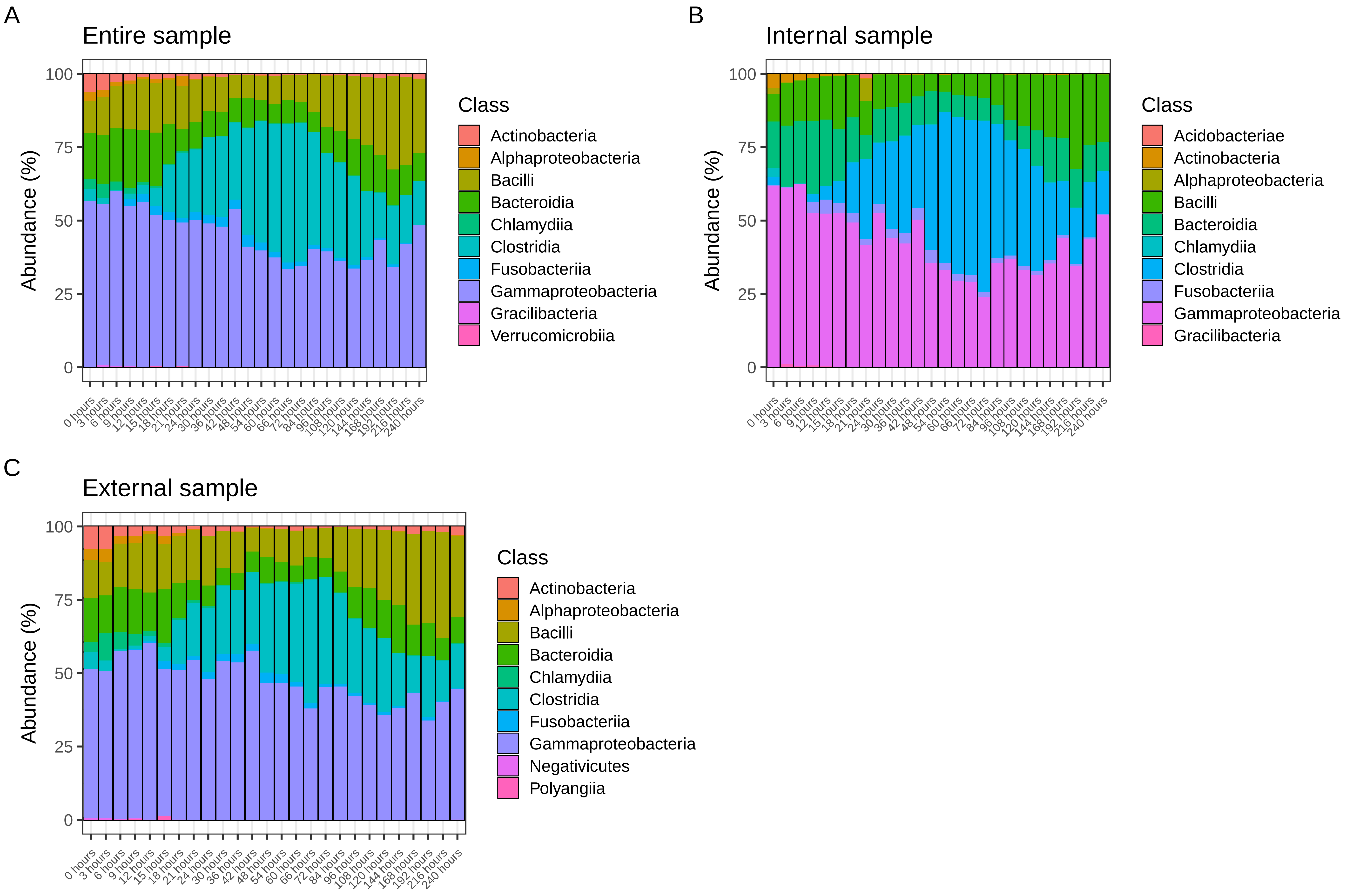
A
Entire sample
Abundance (%)
0
25
50
75
100
0 hours
3 hours
6 hours
9 hours
12 hours
15 hours
18 hours
21 hours
24 hours
30 hours
36 hours
42 hours
48 hours
54 hours
60 hours
66 hours
72 hours
84 hours
96 hours
108 hours
120 hours
144 hours
168 hours
192 hours
216 hours
240 hours
Class
Actinobacteria
Alphaproteobacteria
Bacilli
Bacteroidia
Chlamydiia
Clostridia
Fusobacteriia
Gammaproteobacteria
Gracilibacteria
Verrucomicrobiia
B
Internal sample
Abundance (%)
Class
Acidobacteriae
Actinobacteria
Alphaproteobacteria
Bacilli
Bacteroidia
Chlamydiia
Clostridia
Fusobacteriia
Gammaproteobacteria
Gracilibacteria
C
External sample
Abundance (%)
Class
Actinobacteria
Alphaproteobacteria
Bacilli
Bacteroidia
Chlamydiia
Clostridia
Fusobacteriia
Gammaproteobacteria
Negativicutes
Polyangiia
