# Supplementary material for: Microbiome modelling for post-mortem interval estimation across species and climates: swine analogues in a British summer and external validation in human donors from the USA
Source: Sci Rep. 2026 Aug 19;16:24916. doi: 10.1038/s41598-026-65366-y (PMC13490378; doi:10.1038/s41598-026-65366-y)
Supplement: Supplementary file 4 [file 41598_2026_65366_MOESM5_ESM.pdf]

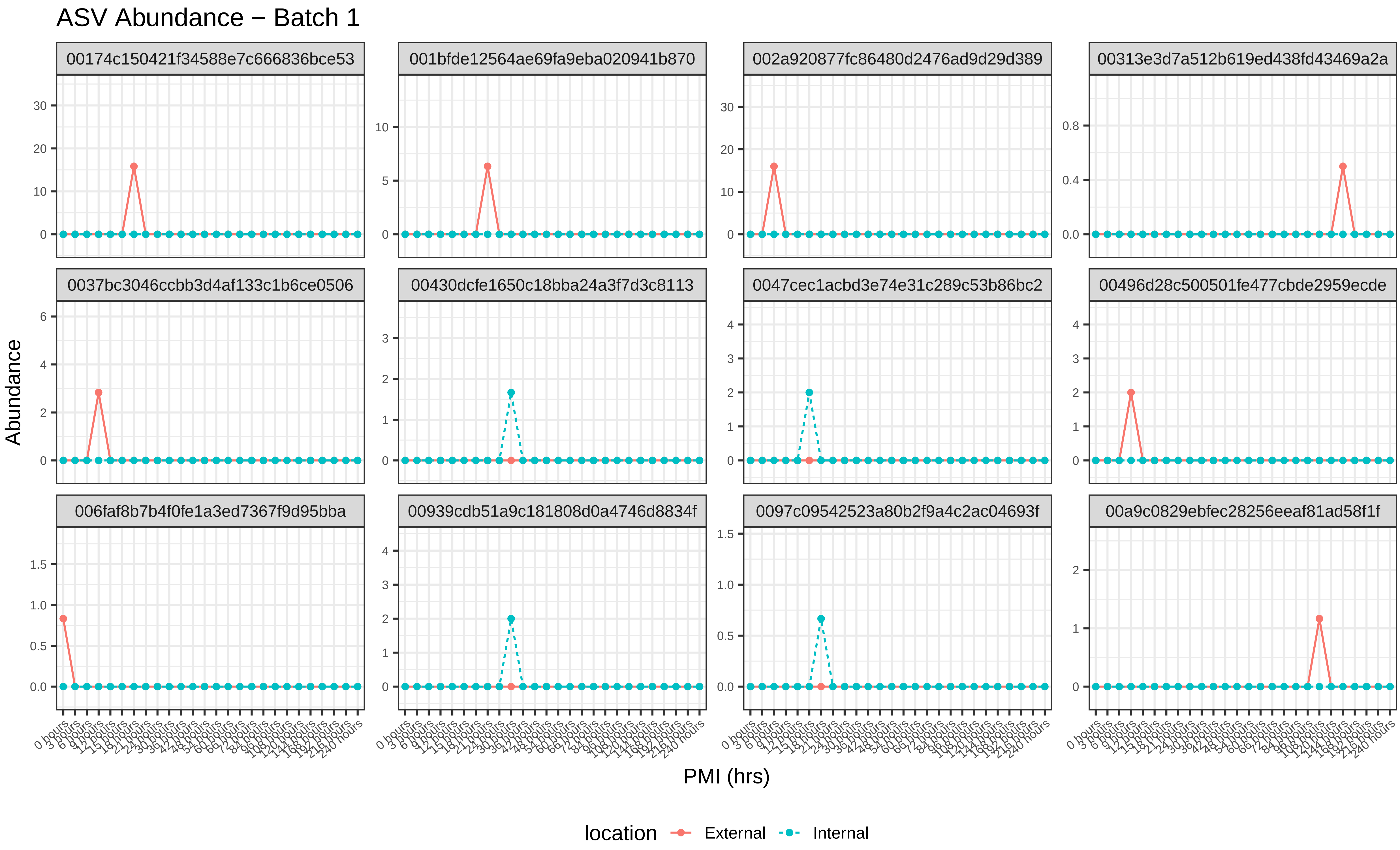
ASV Abundance − Batch 1
00174c150421f34588e7c666836bce53
001bfde12564ae69fa9eba020941b870
002a920877fc86480d2476ad9d29d389
00313e3d7a512b619ed438fd43469a2a
0037bc3046ccbb3d4af133c1b6ce0506
00430dcfe1650c18bba24a3f7d3c8113
0047cec1acbd3e74e31c289c53b86bc2
00496d28c500501fe477cbde2959ecde
006faf8b7b4f0fe1a3ed7367f9d95bba
00939cdb51a9c181808d0a4746d8834f
0097c09542523a80b2f9a4c2ac04693f
00a9c0829ebfec28256eeaf81ad58f1f
Abundance
PMI (hrs)
location
External
Internal

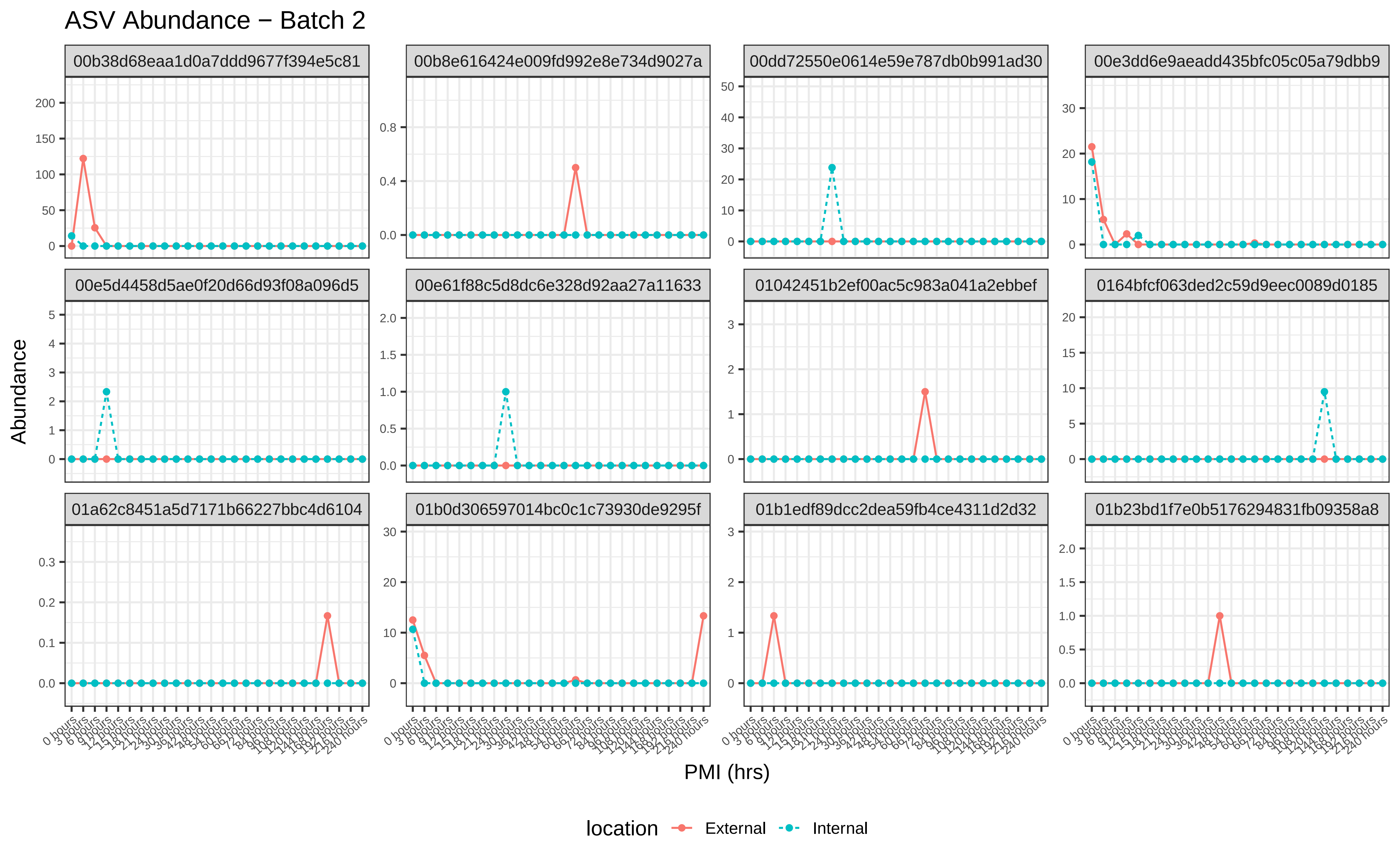
ASV Abundance − Batch 2
00b38d68eaa1d0a7ddd9677f394e5c81
00b8e616424e009fd992e8e734d9027a
00dd72550e0614e59e787db0b991ad30
00e3dd6e9aeadd435bfc05c05a79dbb9
00e5d4458d5ae0f20d66d93f08a096d5
00e61f88c5d8dc6e328d92aa27a11633
01042451b2ef00ac5c983a041a2ebbef
0164bfcf063ded2c59d9eec0089d0185
01a62c8451a5d7171b66227bbc4d6104
01b0d306597014bc0c1c73930de9295f
01b1edf89dcc2dea59fb4ce4311d2d32
01b23bd1f7e0b5176294831fb09358a8
Abundance
0 hours
3 hours
6 hours
9 hours
12 hours
15 hours
18 hours
21 hours
24 hours
30 hours
36 hours
42 hours
48 hours
54 hours
60 hours
66 hours
72 hours
84 hours
96 hours
108 hours
120 hours
144 hours
168 hours
192 hours
216 hours
240 hours
PMI (hrs)
location
External
Internal

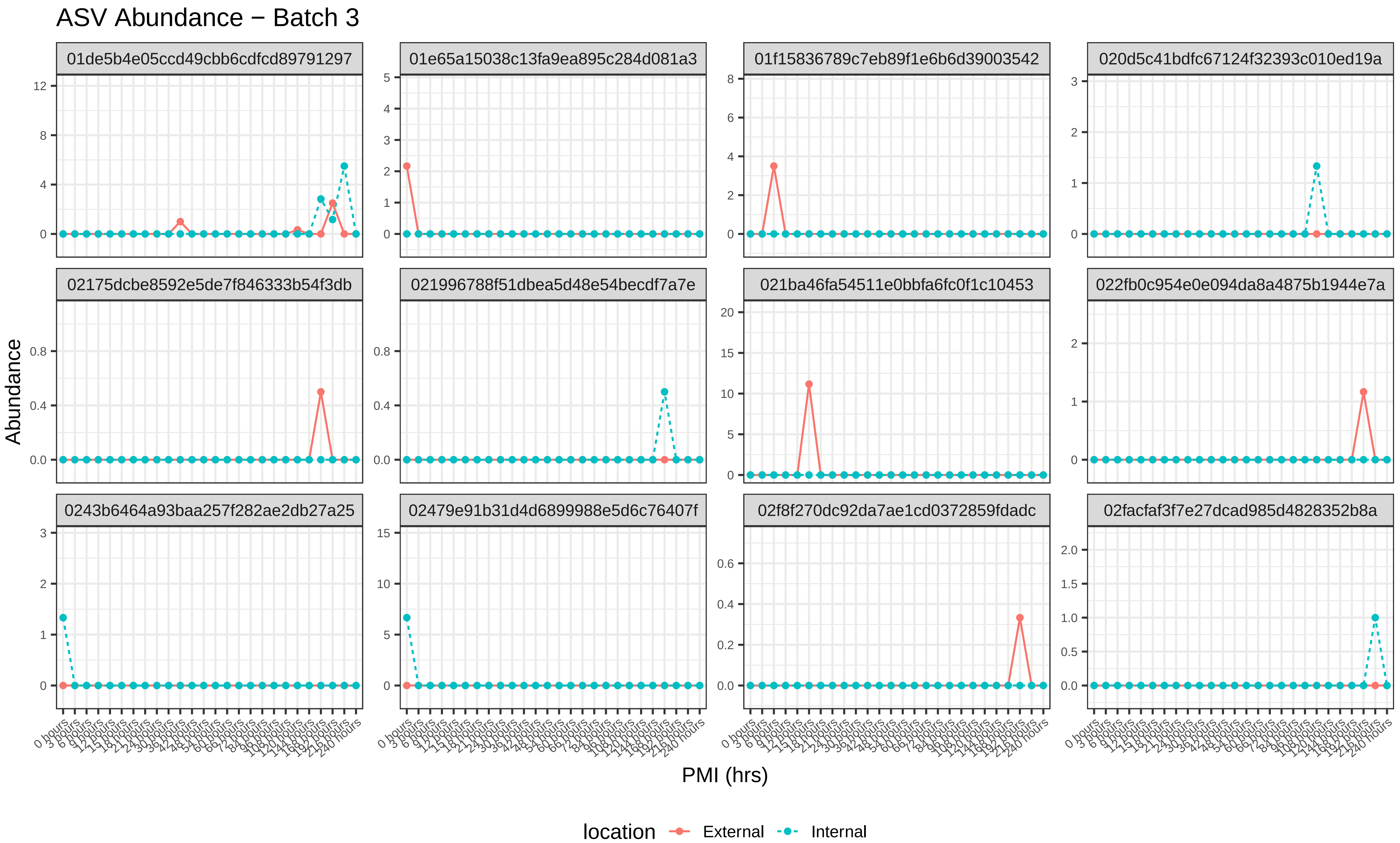
ASV Abundance − Batch 3
01de5b4e05ccd49cbb6cdfcd89791297
01e65a15038c13fa9ea895c284d081a3
01f15836789c7eb89f1e6b6d39003542
020d5c41bdfc67124f32393c010ed19a
02175dcbe8592e5de7f846333b54f3db
021996788f51dbea5d48e54becdf7a7e
021ba46fa54511e0bbfa6fc0f1c10453
022fb0c954e0e094da8a4875b1944e7a
0243b6464a93baa257f282ae2db27a25
02479e91b31d4d6899988e5d6c76407f
02f8f270dc92da7ae1cd0372859fdadc
02facfaf3f7e27dcad985d4828352b8a
Abundance
PMI (hrs)
location
External
Internal

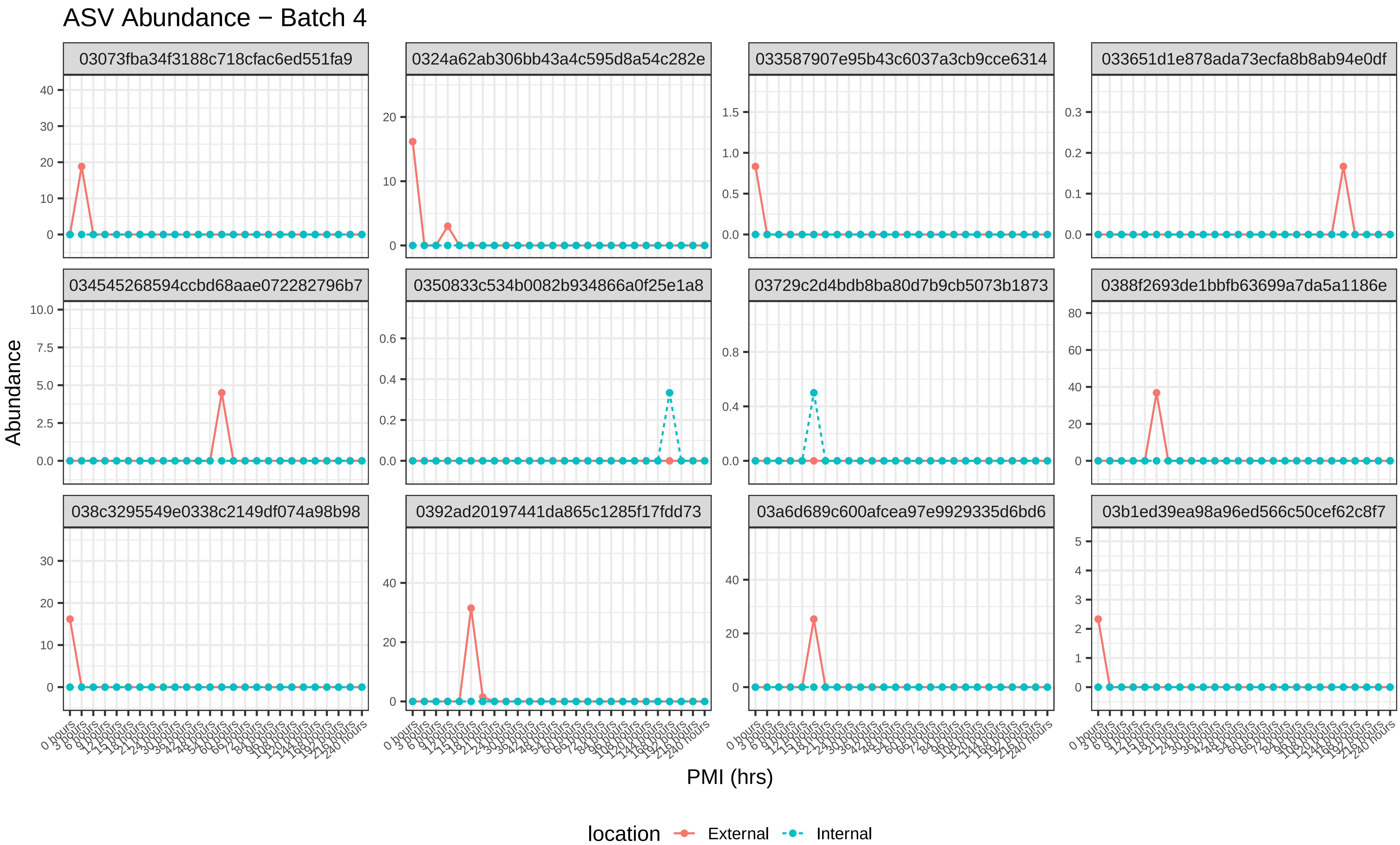
ASV Abundance − Batch 4
03073fba34f3188c718cfac6ed551fa9
0324a62ab306bb43a4c595d8a54c282e
033587907e95b43c6037a3cb9cce6314
033651d1e878ada73ecfa8b8ab94e0df
034545268594ccbd68aae072282796b7
0350833c534b0082b934866a0f25e1a8
03729c2d4bdb8ba80d7b9cb5073b1873
0388f2693de1bbfb63699a7da5a1186e
038c3295549e0338c2149df074a98b98
0392ad20197441da865c1285f17fdd73
03a6d689c600afcea97e9929335d6bd6
03b1ed39ea98a96ed566c50cef62c8f7
Abundance
PMI (hrs)
location
External
Internal

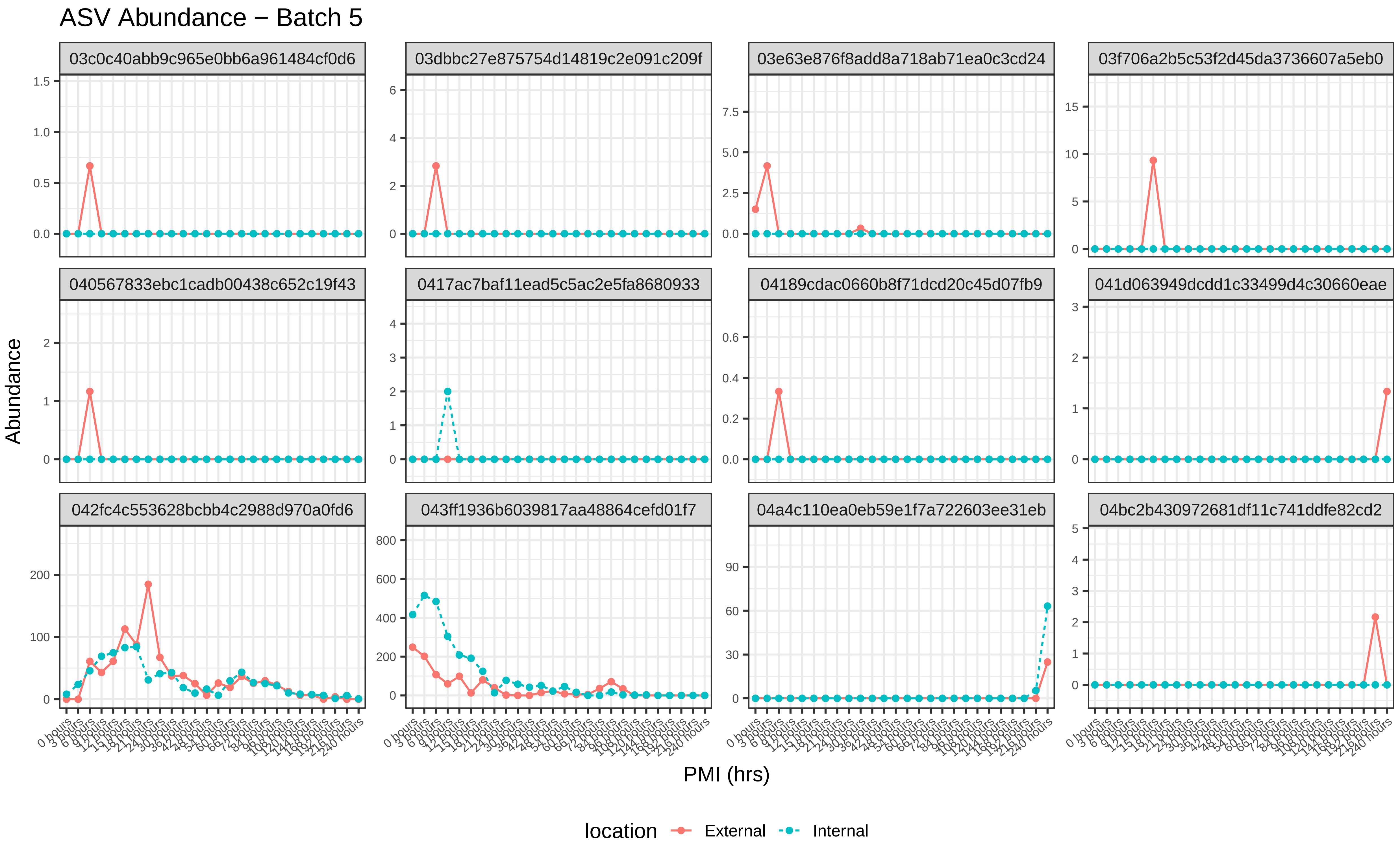
ASV Abundance − Batch 5
03c0c40abb9c965e0bb6a961484cf0d6
03dbbc27e875754d14819c2e091c209f
03e63e876f8add8a718ab71ea0c3cd24
03f706a2b5c53f2d45da3736607a5eb0
040567833ebc1cadb00438c652c19f43
0417ac7baf11ead5c5ac2e5fa8680933
04189cdac0660b8f71dcd20c45d07fb9
041d063949dcdd1c33499d4c30660eae
042fc4c553628bcbb4c2988d970a0fd6
043ff1936b6039817aa48864cefd01f7
04a4c110ea0eb59e1f7a722603ee31eb
04bc2b430972681df11c741ddfe82cd2
Abundance
PMI (hrs)
location
External
Internal

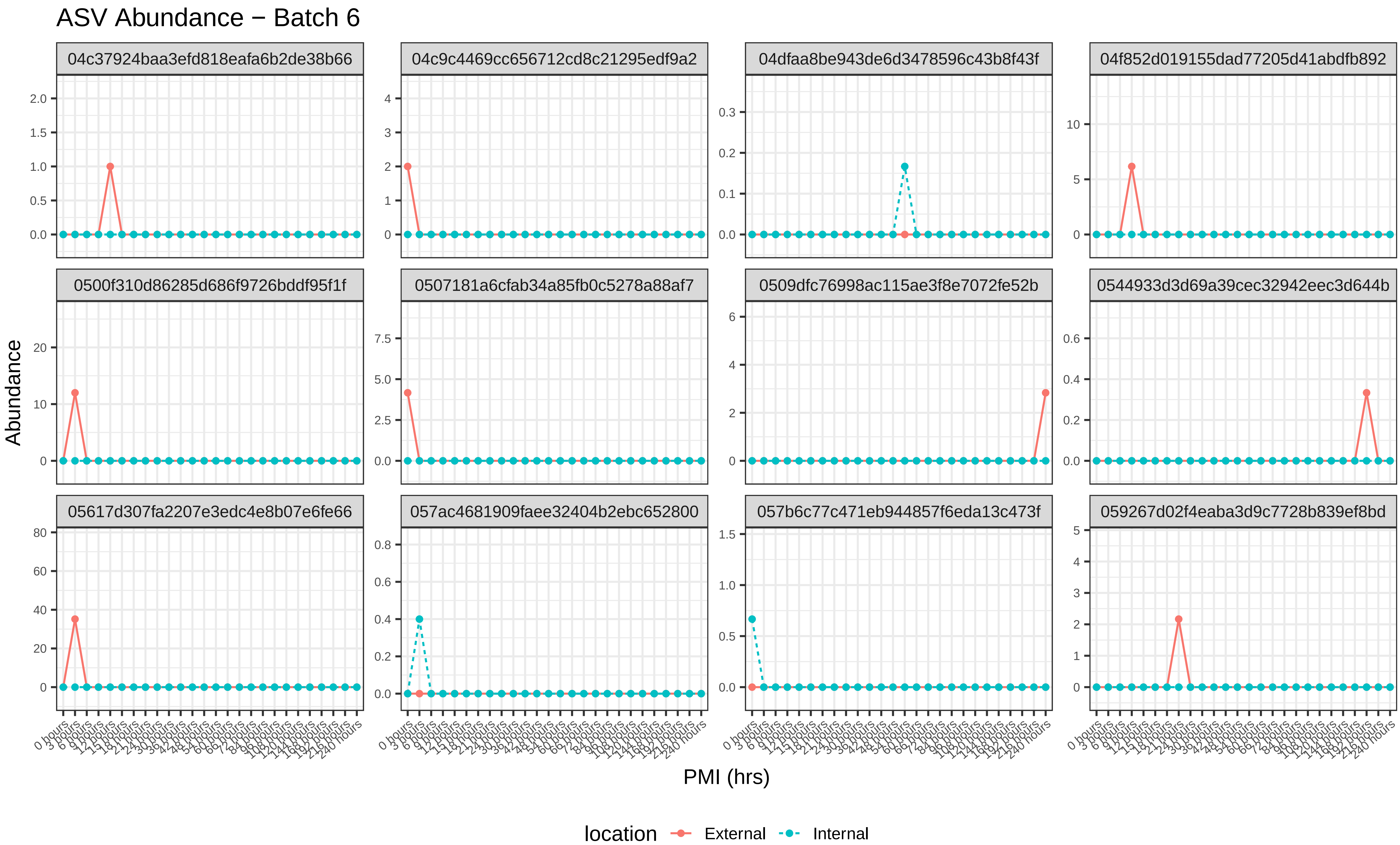
ASV Abundance − Batch 6
04c37924baa3efd818eafa6b2de38b66
04c9c4469cc656712cd8c21295edf9a2
04dfaa8be943de6d3478596c43b8f43f
04f852d019155dad77205d41abdfb892
0500f310d86285d686f9726bddf95f1f
0507181a6cfab34a85fb0c5278a88af7
0509dfc76998ac115ae3f8e7072fe52b
0544933d3d69a39cec32942eec3d644b
05617d307fa2207e3edc4e8b07e6fe66
057ac4681909faee32404b2ebc652800
057b6c77c471eb944857f6eda13c473f
059267d02f4eaba3d9c7728b839ef8bd
Abundance
PMI (hrs)
location
External
Internal

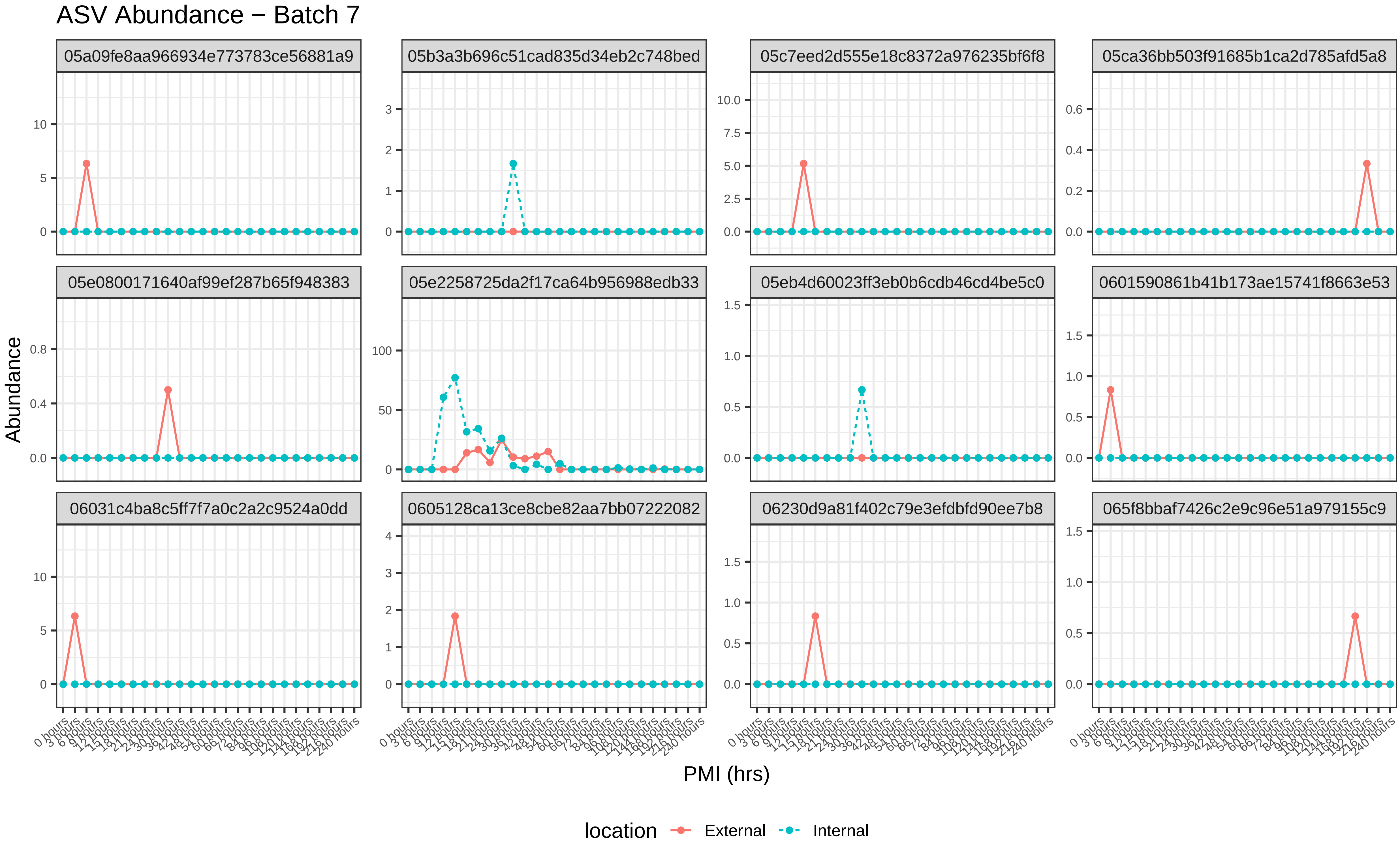

ASV Abundance − Batch 7
05a09fe8aa966934e773783ce56881a9
05b3a3b696c51cad835d34eb2c748bed
05c7eed2d555e18c8372a976235bf6f8
05ca36bb503f91685b1ca2d785afd5a8
05e0800171640af99ef287b65f948383
05e2258725da2f17ca64b956988edb33
05eb4d60023ff3eb0b6cdb46cd4be5c0
0601590861b41b173ae15741f8663e53
06031c4ba8c5ff7f7a0c2a2c9524a0dd
0605128ca13ce8cbe82aa7bb07222082
06230d9a81f402c79e3efdbfd90ee7b8
065f8bbaf7426c2e9c96e51a979155c9
Abundance
PMI (hrs)
location
External
Internal

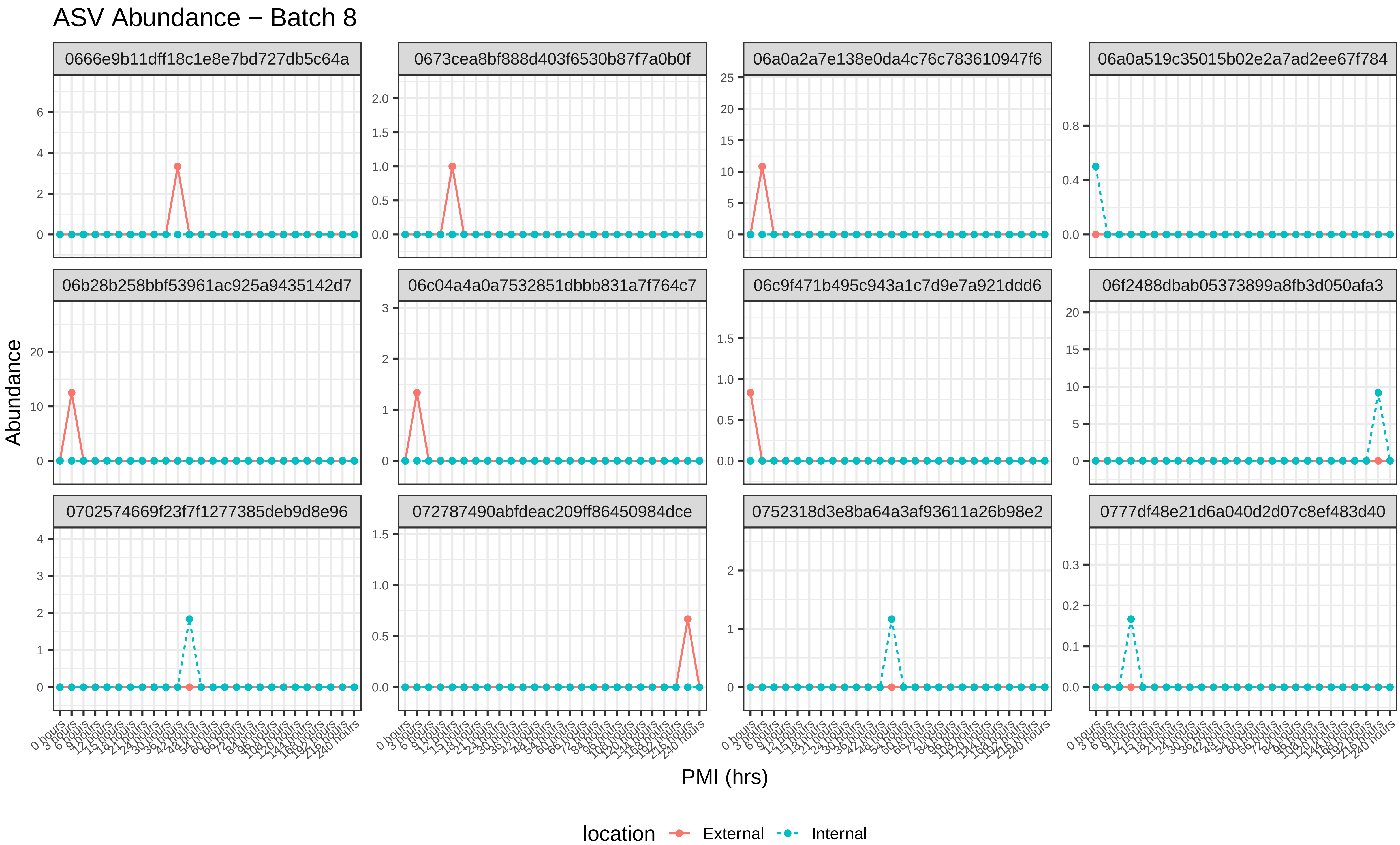
ASV Abundance − Batch 8
0666e9b11dff18c1e8e7bd727db5c64a
0673cea8bf888d403f6530b87f7a0b0f
06a0a2a7e138e0da4c76c783610947f6
06a0a519c35015b02e2a7ad2ee67f784
06b28b258bbf53961ac925a9435142d7
06c04a4a0a7532851dbbb831a7f764c7
06c9f471b495c943a1c7d9e7a921ddd6
06f2488dbab05373899a8fb3d050afa3
0702574669f23f7f1277385deb9d8e96
072787490abfdeac209ff86450984dce
0752318d3e8ba64a3af93611a26b98e2
0777df48e21d6a040d2d07c8ef483d40
Abundance
PMI (hrs)
location
External
Internal

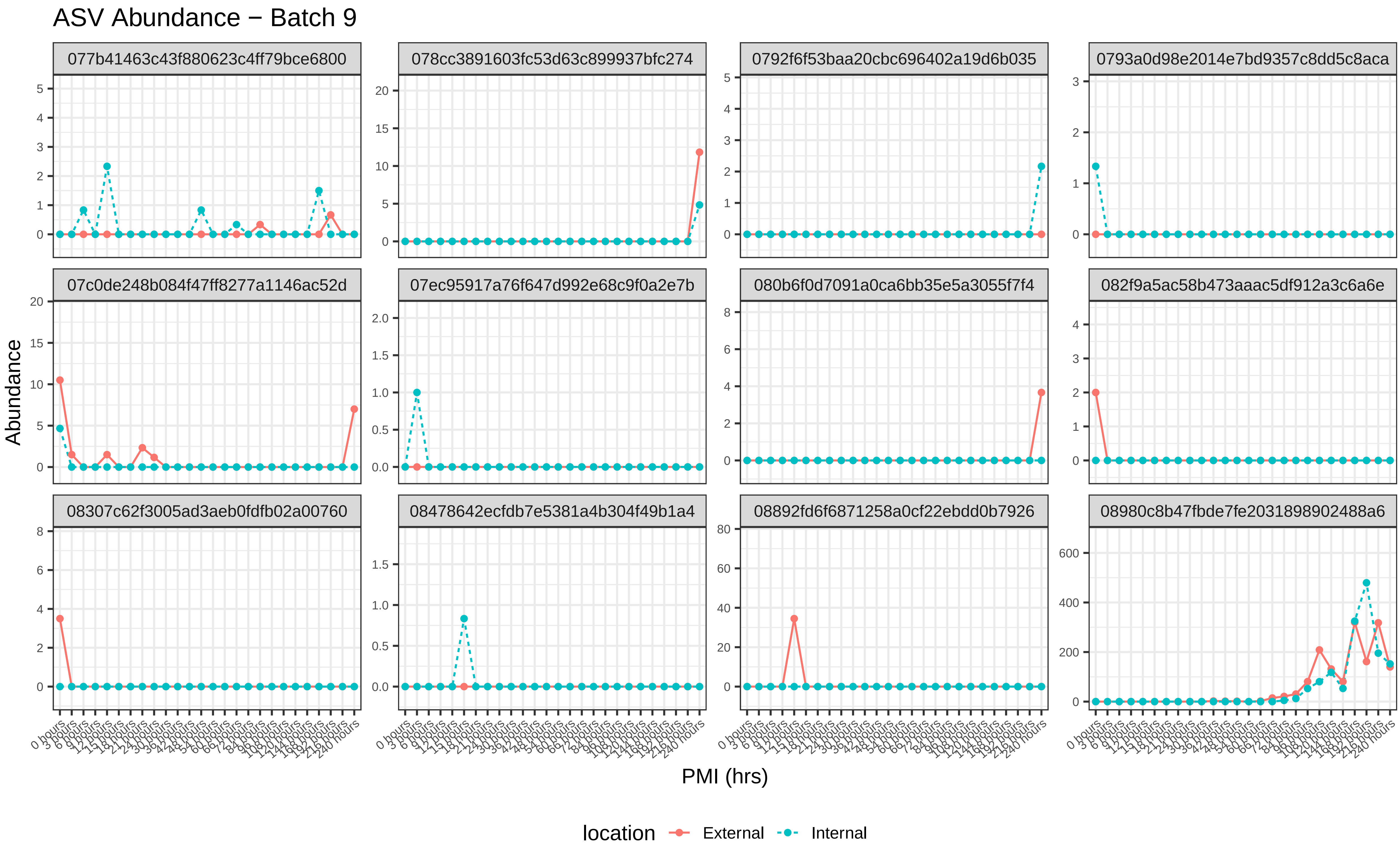

ASV Abundance − Batch 9
077b41463c43f880623c4ff79bce6800
078cc3891603fc53d63c899937bfc274
0792f6f53baa20cbc696402a19d6b035
0793a0d98e2014e7bd9357c8dd5c8aca
07c0de248b084f47ff8277a1146ac52d
07ec95917a76f647d992e68c9f0a2e7b
080b6f0d7091a0ca6bb35e5a3055f7f4
082f9a5ac58b473aaac5df912a3c6a6e
08307c62f3005ad3aeb0fdfb02a00760
08478642ecfdb7e5381a4b304f49b1a4
08892fd6f6871258a0cf22ebdd0b7926
08980c8b47fbde7fe2031898902488a6
Abundance
PMI (hrs)
location
External
Internal

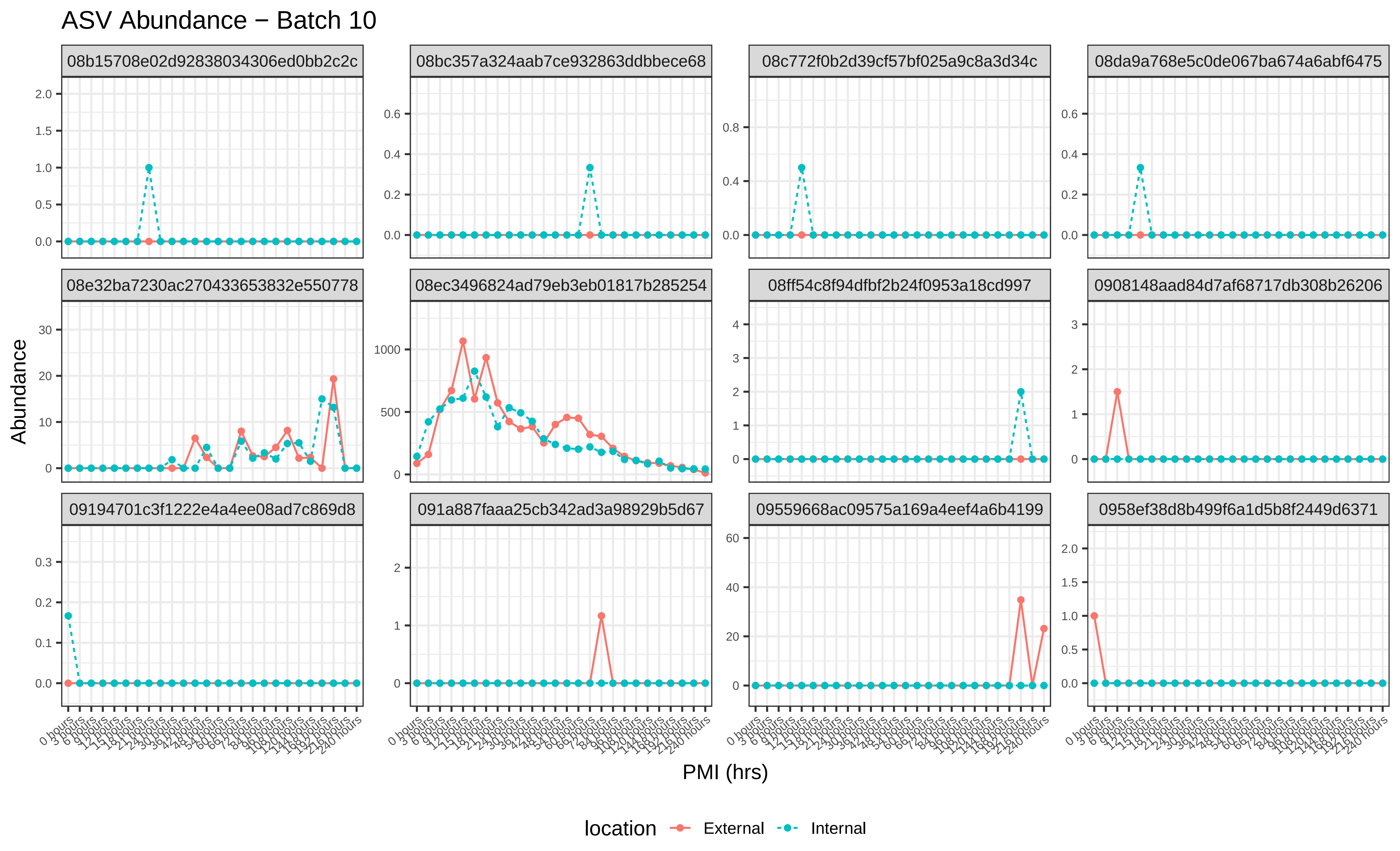
ASV Abundance − Batch 10
08b15708e02d92838034306ed0bb2c2c
08bc357a324aab7ce932863ddbbece68
08c772f0b2d39cf57bf025a9c8a3d34c
08da9a768e5c0de067ba674a6abf6475
08e32ba7230ac270433653832e550778
08ec3496824ad79eb3eb01817b285254
08ff54c8f94dfbf2b24f0953a18cd997
0908148aad84d7af68717db308b26206
09194701c3f1222e4a4ee08ad7c869d8
091a887faaa25cb342ad3a98929b5d67
09559668ac09575a169a4eef4a6b4199
0958ef38d8b499f6a1d5b8f2449d6371
Abundance
PMI (hrs)
location
External
Internal

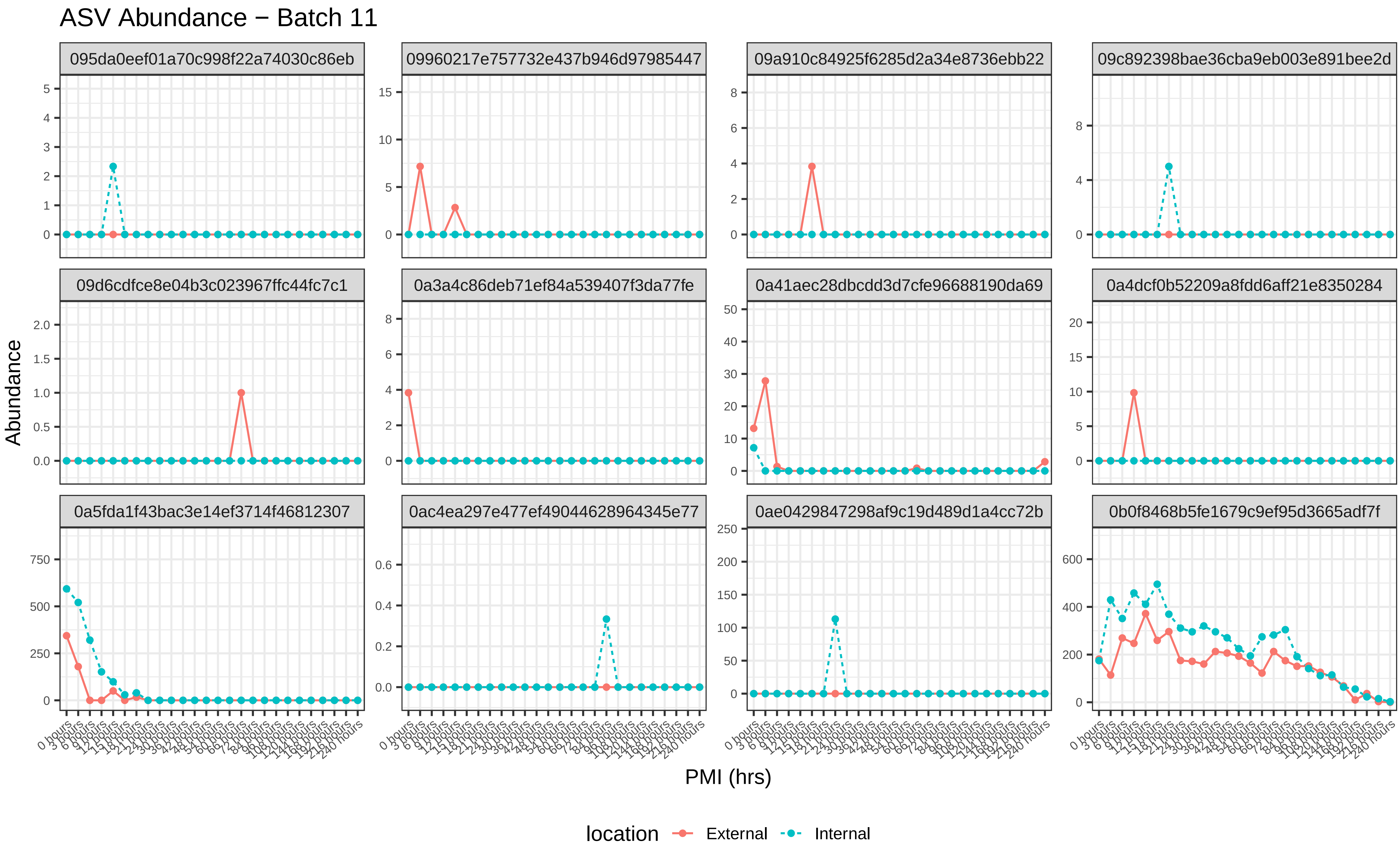

ASV Abundance − Batch 11
095da0eef01a70c998f22a74030c86eb
09960217e757732e437b946d97985447
09a910c84925f6285d2a34e8736ebb22
09c892398bae36cba9eb003e891bee2d
09d6cdfce8e04b3c023967ffc44fc7c1
0a3a4c86deb71ef84a539407f3da77fe
0a41aec28dbcdd3d7cfe96688190da69
0a4dcf0b52209a8fdd6aff21e8350284
0a5fda1f43bac3e14ef3714f46812307
0ac4ea297e477ef49044628964345e77
0ae0429847298af9c19d489d1a4cc72b
0b0f8468b5fe1679c9ef95d3665adf7f
Abundance
PMI (hrs)
location
External
Internal

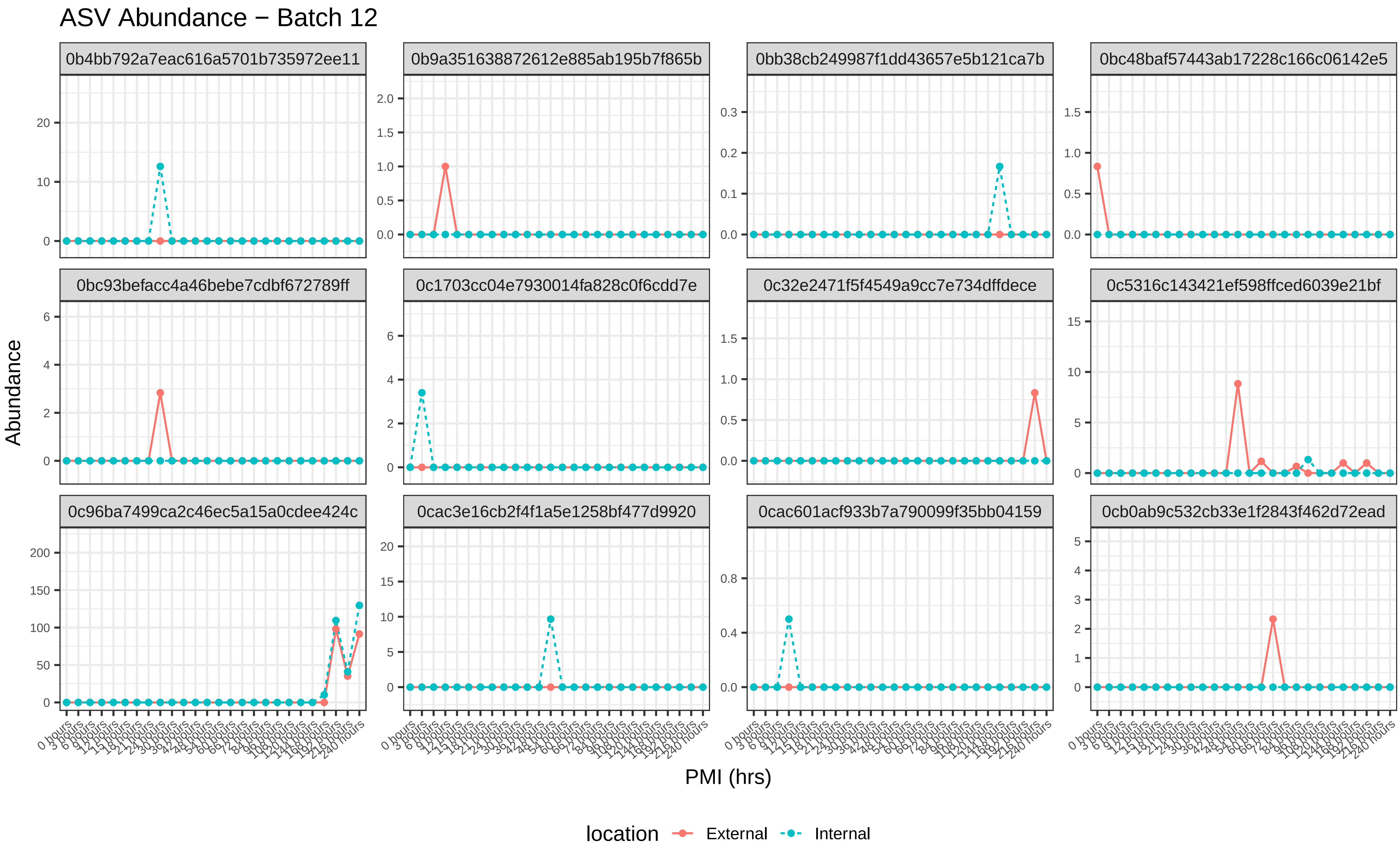

ASV Abundance − Batch 12
0b4bb792a7eac616a5701b735972ee11
0b9a351638872612e885ab195b7f865b
0bb38cb249987f1dd43657e5b121ca7b
0bc48baf57443ab17228c166c06142e5
0bc93befacc4a46bebe7cdbf672789ff
0c1703cc04e7930014fa828c0f6cdd7e
0c32e2471f5f4549a9cc7e734dffdece
0c5316c143421ef598ffced6039e21bf
0c96ba7499ca2c46ec5a15a0cdee424c
0cac3e16cb2f4f1a5e1258bf477d9920
0cac601acf933b7a790099f35bb04159
0cb0ab9c532cb33e1f2843f462d72ead
Abundance
PMI (hrs)
location
External
Internal

ASV Abundance − Batch 13

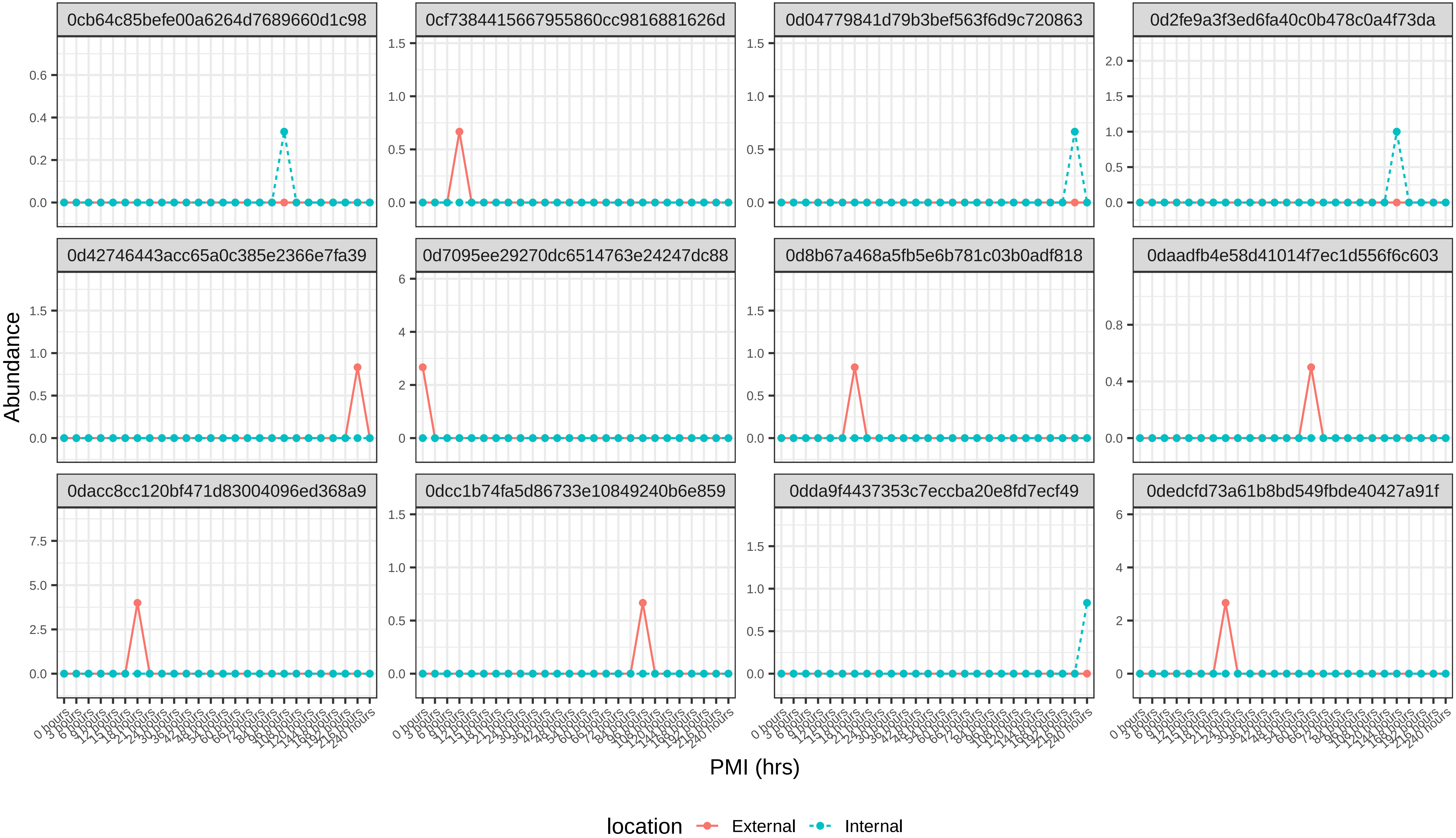

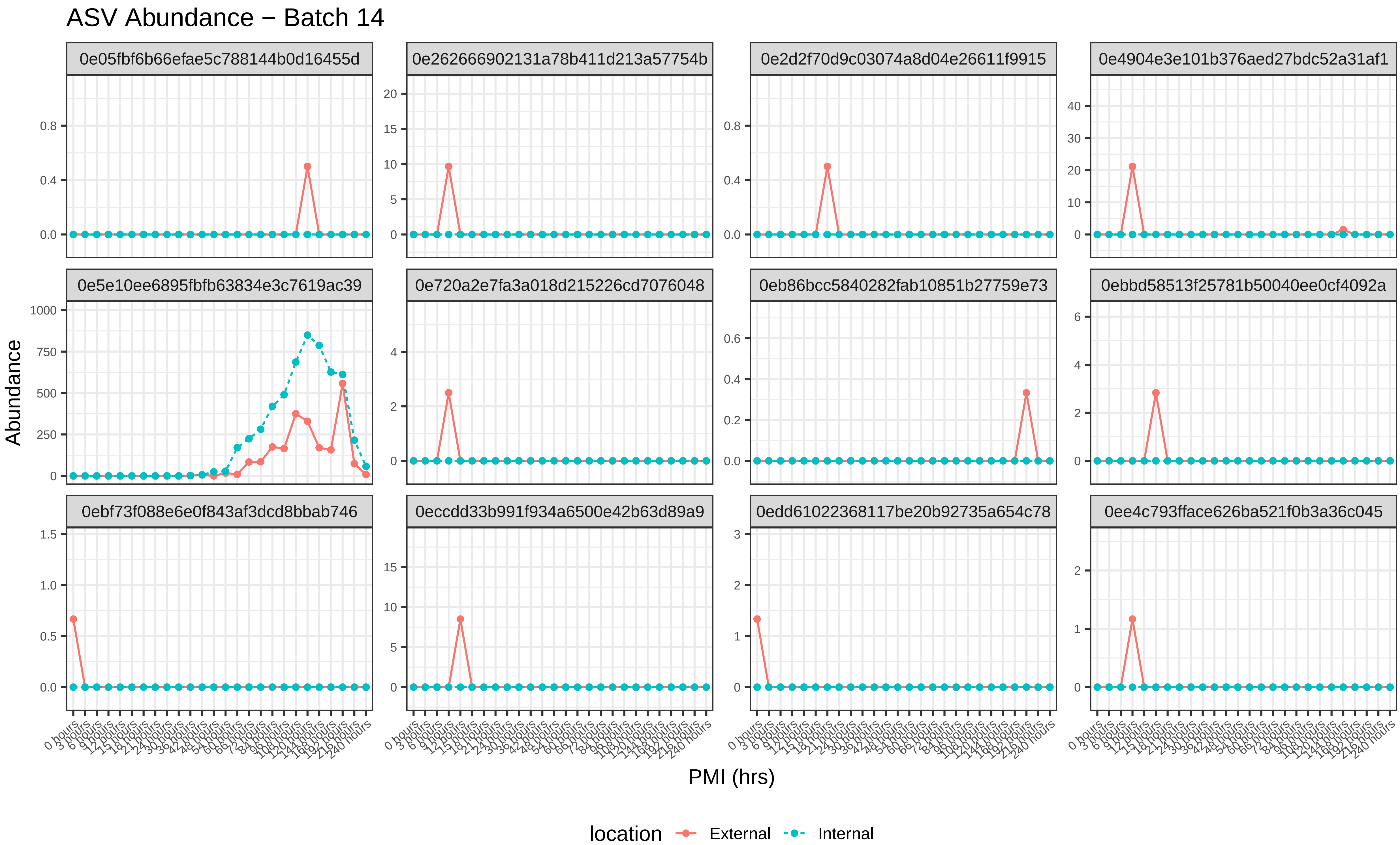
ASV Abundance − Batch 14
0e05fbf6b66efae5c788144b0d16455d
0e26266902131a78b411d213a57754b
0e2d2f70d9c03074a8d04e26611f9915
0e4904e3e101b376aed27bdc52a31af1
0e5e10ee6895fbfb63834e3c7619ac39
0e720a2e7fa3a018d215226cd7076048
0eb86bcc5840282fab10851b27759e73
0ebbd58513f25781b50040ee0cf4092a
0ebf73f088e6e0f843af3dcd8bbab746
0eccdd33b991f934a6500e42b63d89a9
0edd61022368117be20b92735a654c78
0ee4c793fface626ba521f0b3a36c045
Abundance
PMI (hrs)
location
External
Internal

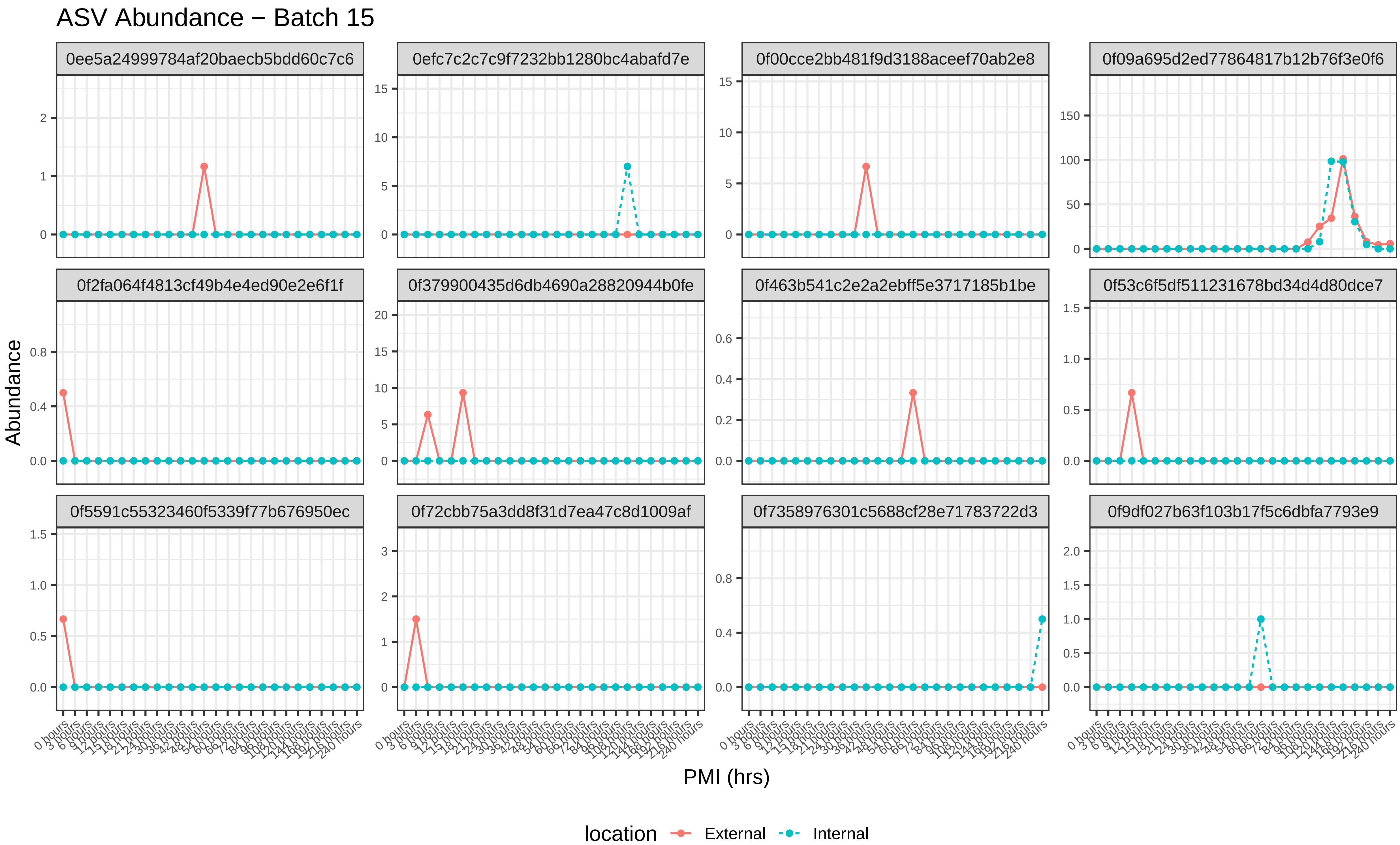

ASV Abundance − Batch 15
0ee5a24999784af20baecb5bdd60c7c6
0efc7c2c7c9f7232bb1280bc4abafd7e
0f00cce2bb481f9d3188aceef70ab2e8
0f09a695d2ed77864817b12b76f3e0f6
0f2fa064f4813cf49b4e4ed90e2e6f1f
0f379900435d6db4690a28820944b0fe
0f463b541c2e2a2ebff5e3717185b1be
0f53c6f5df511231678bd34d4d80dce7
0f5591c55323460f5339f77b676950ec
0f72cbb75a3dd8f31d7ea47c8d1009af
0f7358976301c5688cf28e71783722d3
0f9df027b63f103b17f5c6dbfa7793e9
Abundance
PMI (hrs)
location
External
Internal

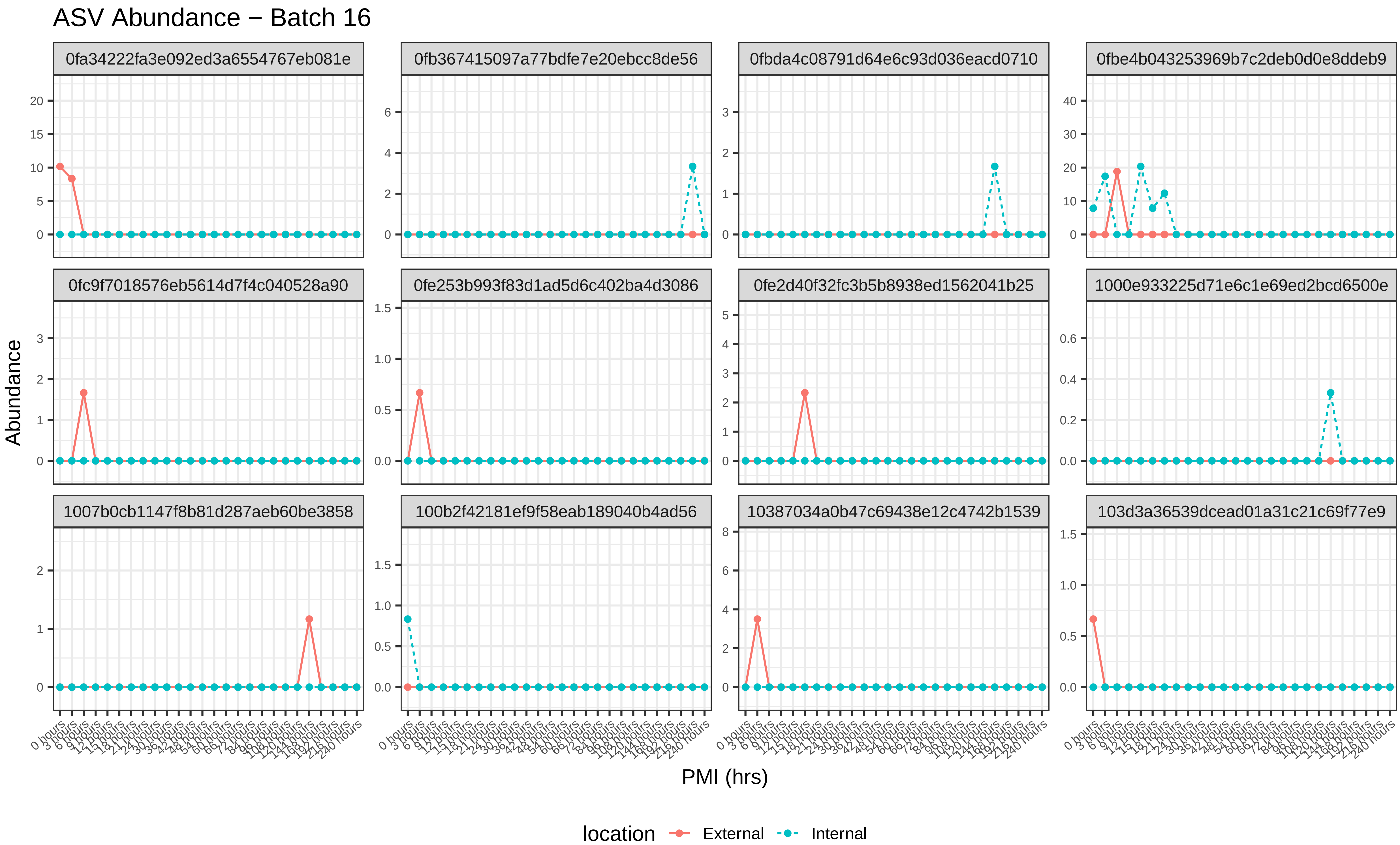
ASV Abundance − Batch 16
0fa34222fa3e092ed3a6554767eb081e
0fb367415097a77bdfe7e20ebcc8de56
0fbda4c08791d64e6c93d036eacd0710
0fbe4b043253969b7c2deb0d0e8ddeb9
0fc9f7018576eb5614d7f4c040528a90
0fe253b993f83d1ad5d6c402ba4d3086
0fe2d40f32fc3b5b8938ed1562041b25
1000e933225d71e6c1e69ed2bcd6500e
1007b0cb1147f8b81d287aeb60be3858
100b2f42181ef9f58eab189040b4ad56
10387034a0b47c69438e12c4742b1539
103d3a36539dcead01a31c21c69f77e9
Abundance
PMI (hrs)
location
External
Internal

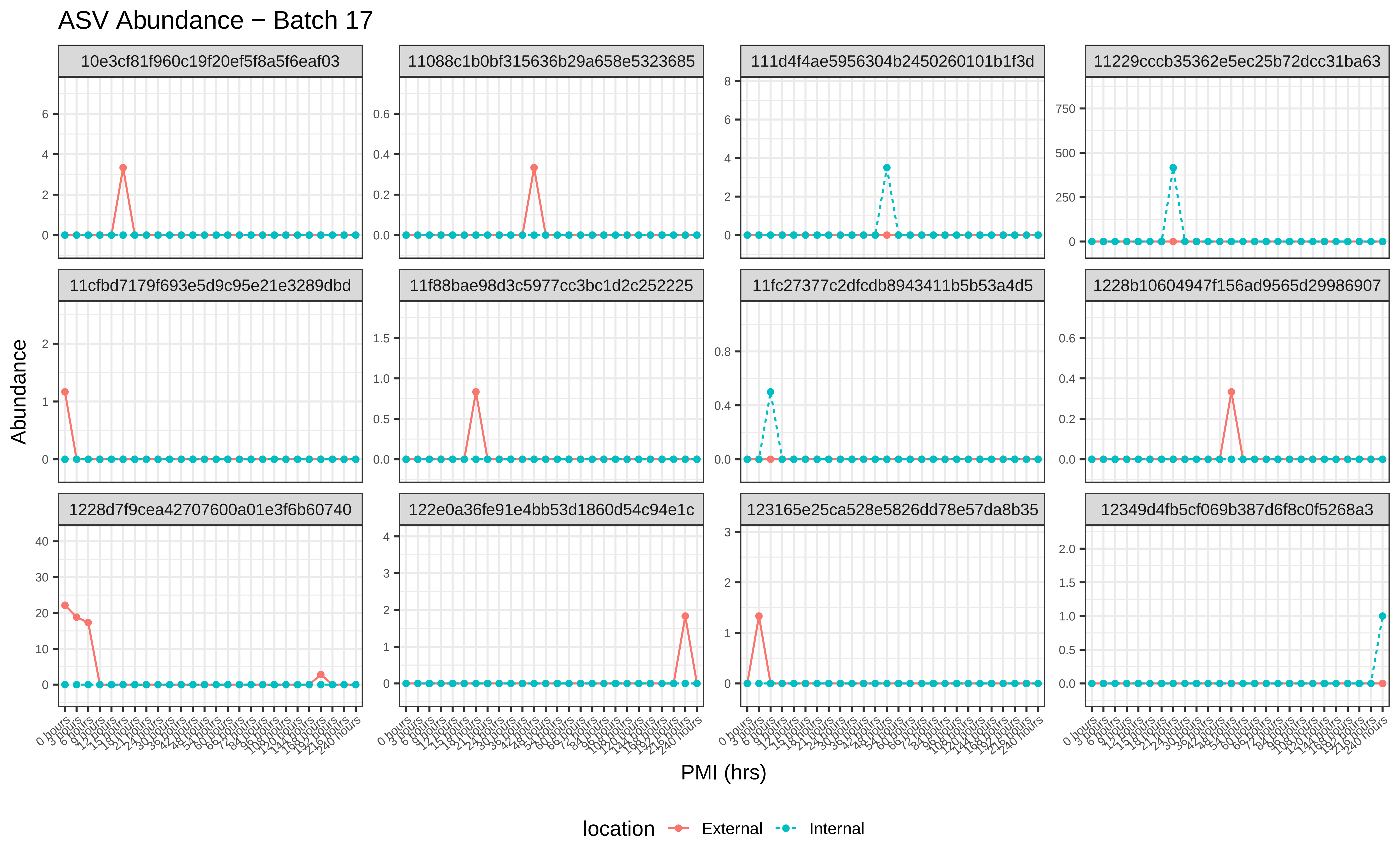

ASV Abundance − Batch 17
10e3cf81f960c19f20ef5f8a5f6eaf03
11088c1b0bf315636b29a658e5323685
111d4f4ae5956304b2450260101b1f3d
11229cccb35362e5ec25b72dcc31ba63
11cfbd7179f693e5d9c95e21e3289dbd
11f88bae98d3c5977cc3bc1d2c252225
11fc27377c2dfcdb8943411b5b53a4d5
1228b10604947f156ad9565d29986907
1228d7f9cea42707600a01e3f6b60740
122e0a36fe91e4bb53d1860d54c94e1c
123165e25ca528e5826dd78e57da8b35
12349d4fb5cf069b387d6f8c0f5268a3
Abundance
PMI (hrs)
location
External
Internal

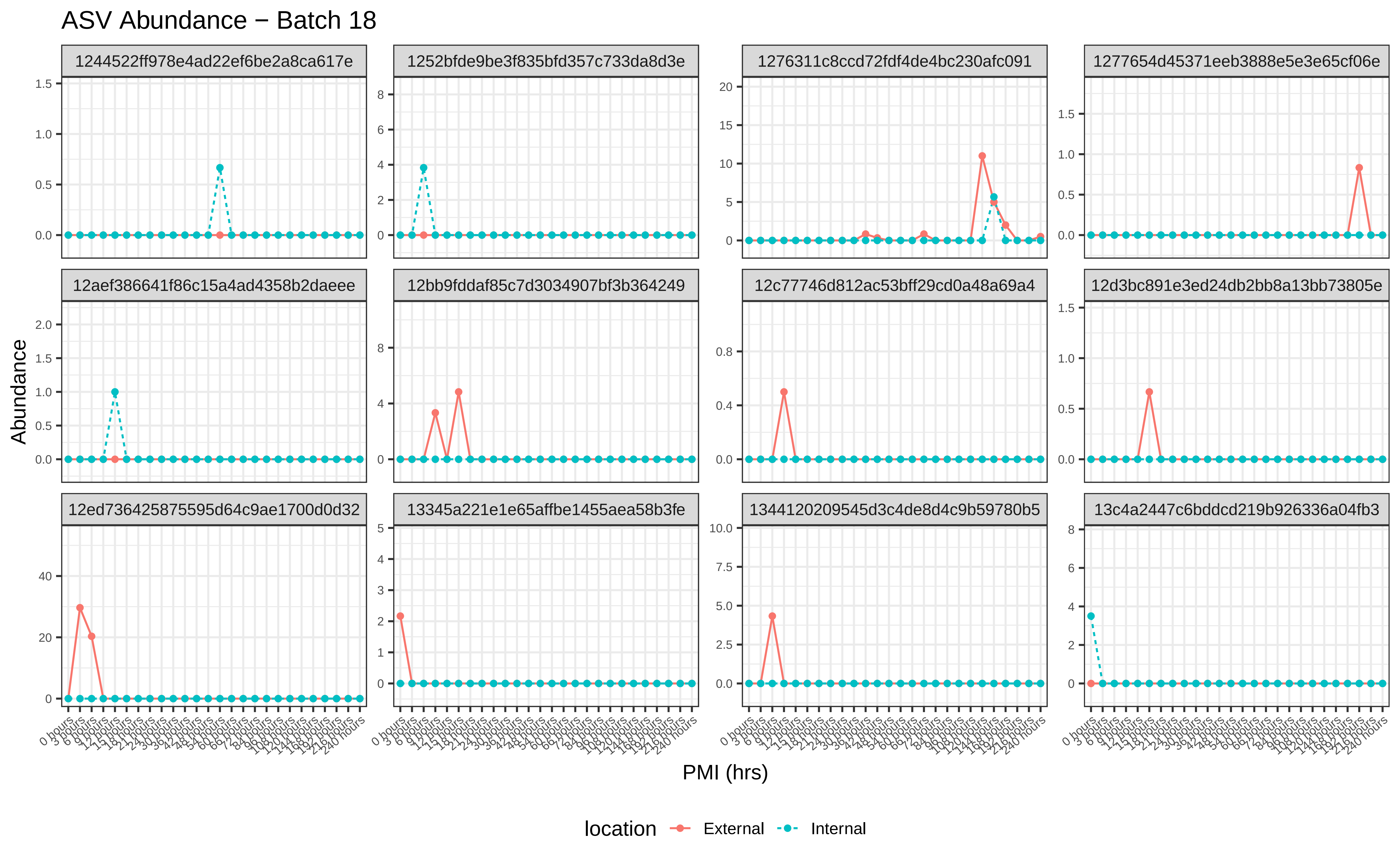

ASV Abundance − Batch 18
1244522ff978e4ad22ef6be2a8ca617e
1252bfde9be3f835bfd357c733da8d3e
1276311c8ccd72fdf4de4bc230afc091
1277654d45371eeb3888e5e3e65cf06e
12aef386641f86c15a4ad4358b2daeee
12bb9fddaf85c7d3034907bf3b364249
12c77746d812ac53bff29cd0a48a69a4
12d3bc891e3ed24db2bb8a13bb73805e
12ed736425875595d64c9ae1700d0d32
13345a221e1e65affbe1455aea58b3fe
1344120209545d3c4de8d4c9b59780b5
13c4a2447c6bddcd219b926336a04fb3
Abundance
PMI (hrs)
location
External
Internal

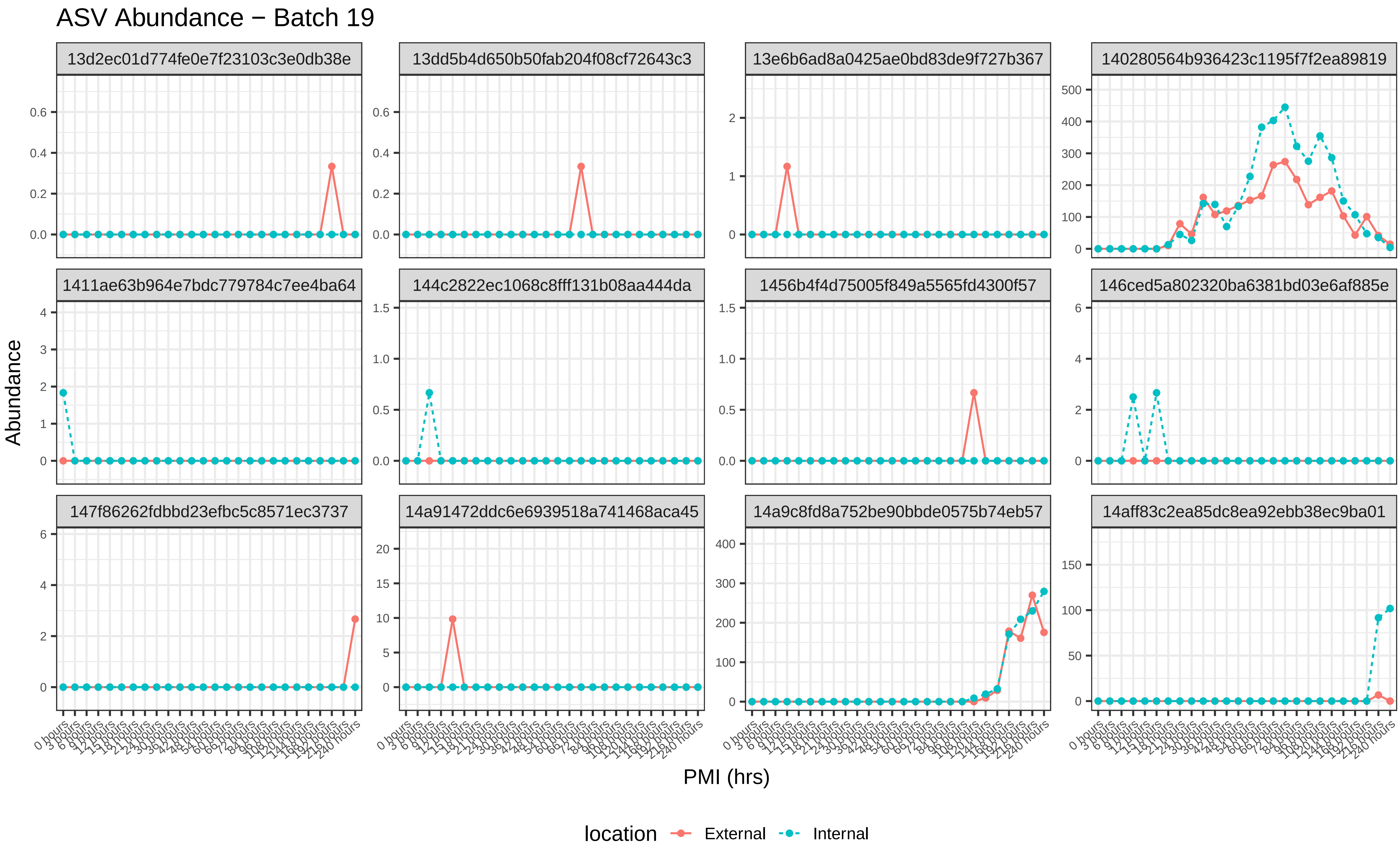
ASV Abundance − Batch 19
13d2ec01d774fe0e7f23103c3e0db38e
13dd5b4d650b50fab204f08cf72643c3
13e6b6ad8a0425ae0bd83de9f727b367
140280564b936423c1195f7f2ea89819
1411ae63b964e7bdc779784c7ee4ba64
144c2822ec1068c8fff131b08aa444da
1456b4f4d75005f849a5565fd4300f57
146ced5a802320ba6381bd03e6af885e
147f86262fdbbd23efbc5c8571ec3737
14a91472ddc6e6939518a741468aca45
14a9c8fd8a752be90bbde0575b74eb57
14aff83c2ea85dc8ea92ebb38ec9ba01
Abundance
PMI (hrs)
location
External
Internal

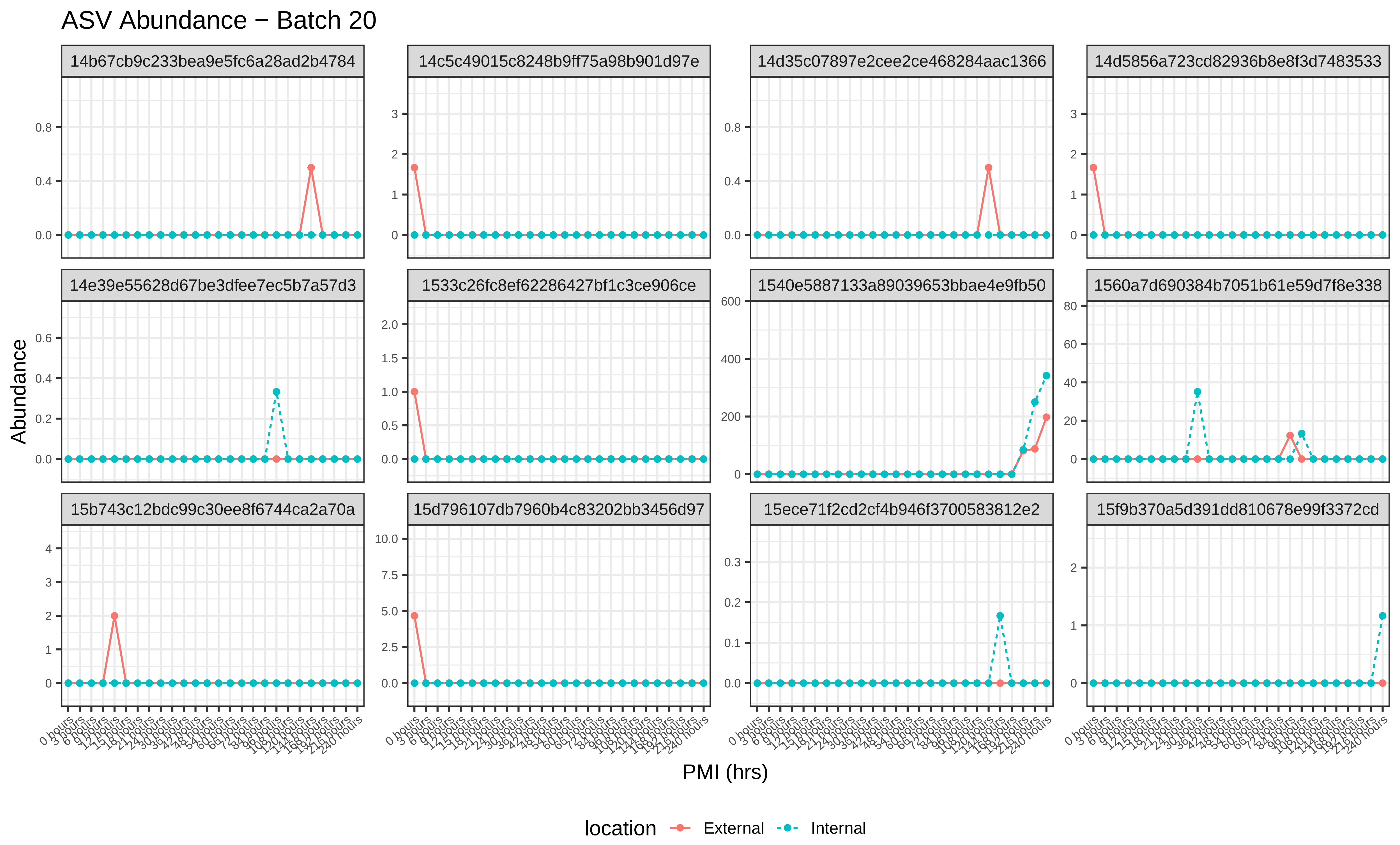
ASV Abundance − Batch 20
14b67cb9c233bea9e5fc6a28ad2b4784
14c5c49015c8248b9ff75a98b901d97e
14d35c07897e2cee2ce468284aac1366
14d5856a723cd82936b8e8f3d7483533
14e39e55628d67be3dfee7ec5b7a57d3
1533c26fc8ef62286427bf1c3ce906ce
1540e5887133a89039653bbae4e9fb50
1560a7d690384b7051b61e59d7f8e338
15b743c12bdc99c30ee8f6744ca2a70a
15d796107db7960b4c83202bb3456d97
15ece71f2cd2cf4b946f3700583812e2
15f9b370a5d391dd810678e99f3372cd
Abundance
PMI (hrs)
location
External
Internal

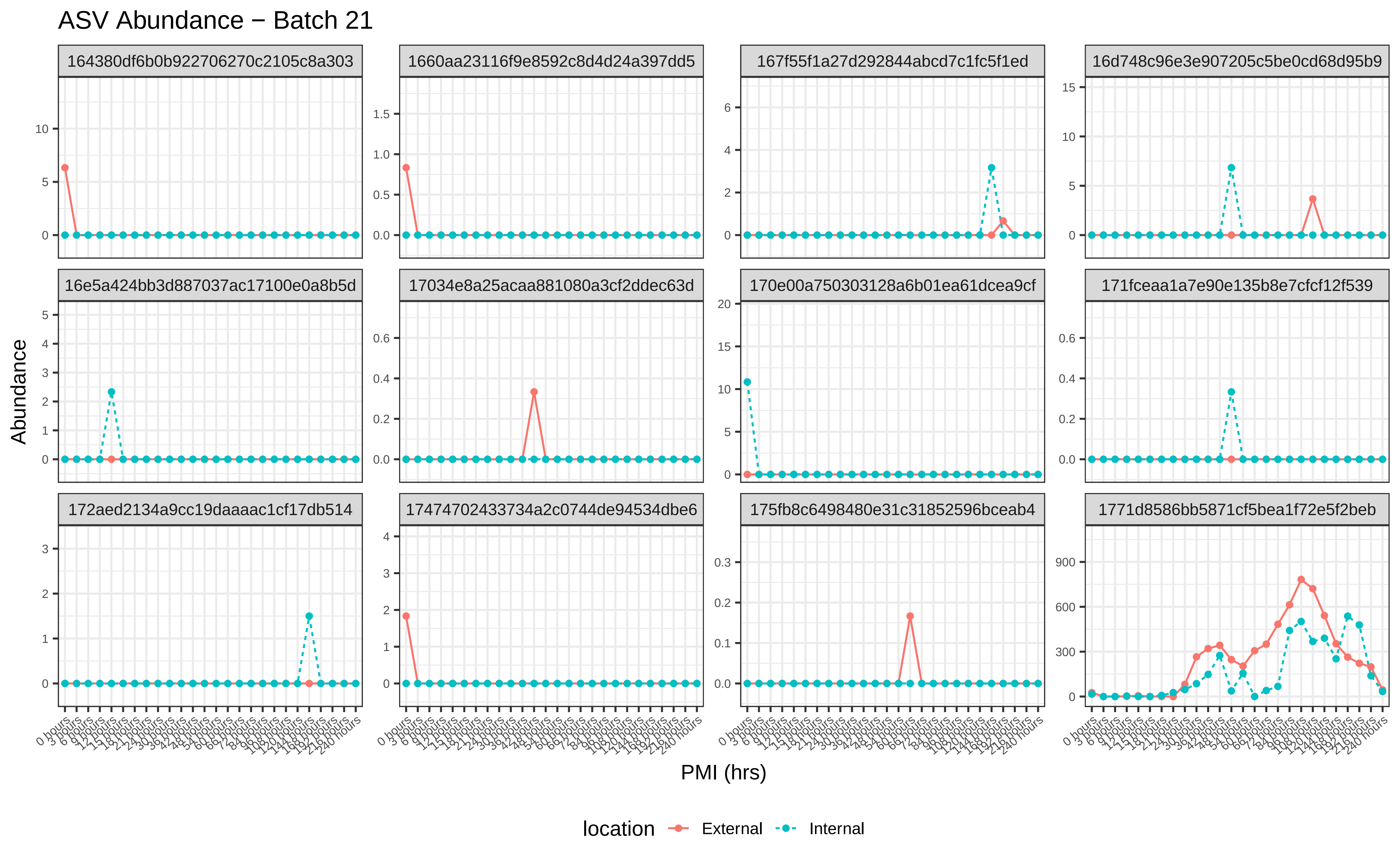

ASV Abundance − Batch 21
164380df6b0b922706270c2105c8a303
1660aa23116f9e8592c8d4d24a397dd5
167f55f1a27d292844abcd7c1fc5f1ed
16d748c96e3e907205c5be0cd68d95b9
16e5a424bb3d887037ac17100e0a8b5d
17034e8a25acaa881080a3cf2ddec63d
170e00a750303128a6b01ea61dcea9cf
171fceaa1a7e90e135b8e7cfcf12f539
172aed2134a9cc19daaaac1cf17db514
17474702433734a2c0744de94534dbe6
175fb8c6498480e31c31852596bceab4
1771d8586bb5871cf5bea1f72e5f2beb
Abundance
PMI (hrs)
location
External
Internal

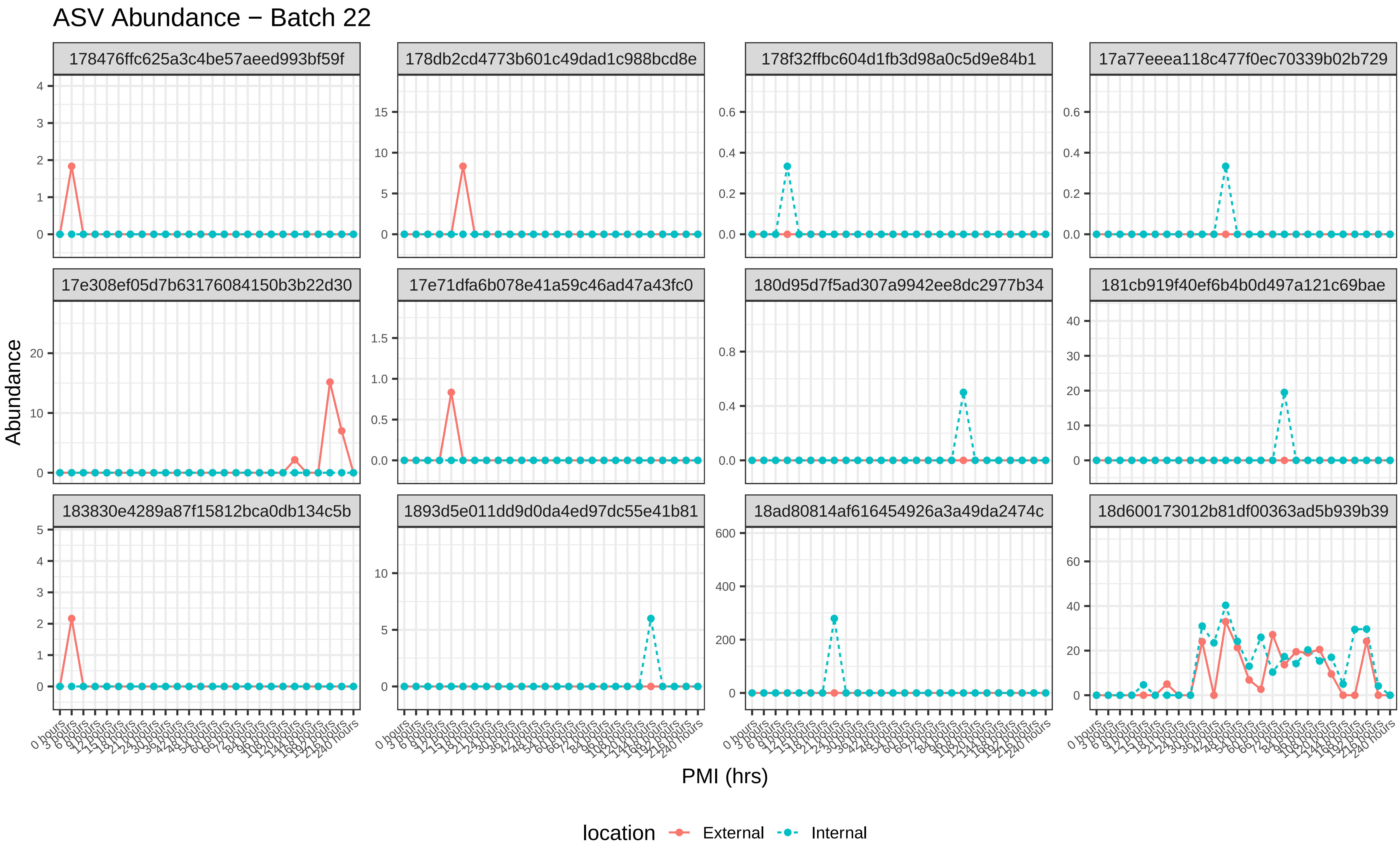
ASV Abundance − Batch 22
178476ffc625a3c4be57aeed993bf59f
178db2cd4773b601c49dad1c988bcd8e
178f32ffbc604d1fb3d98a0c5d9e84b1
17a77eeea118c477f0ec70339b02b729
17e308ef05d7b63176084150b3b22d30
17e71dfa6b078e41a59c46ad47a43fc0
180d95d7f5ad307a9942ee8dc2977b34
181cb919f40ef6b4b0d497a121c69bae
183830e4289a87f15812bca0db134c5b
1893d5e011dd9d0da4ed97dc55e41b81
18ad80814af616454926a3a49da2474c
18d600173012b81df00363ad5b939b39
Abundance
PMI (hrs)
location
External
Internal

ASV Abundance − Batch 23

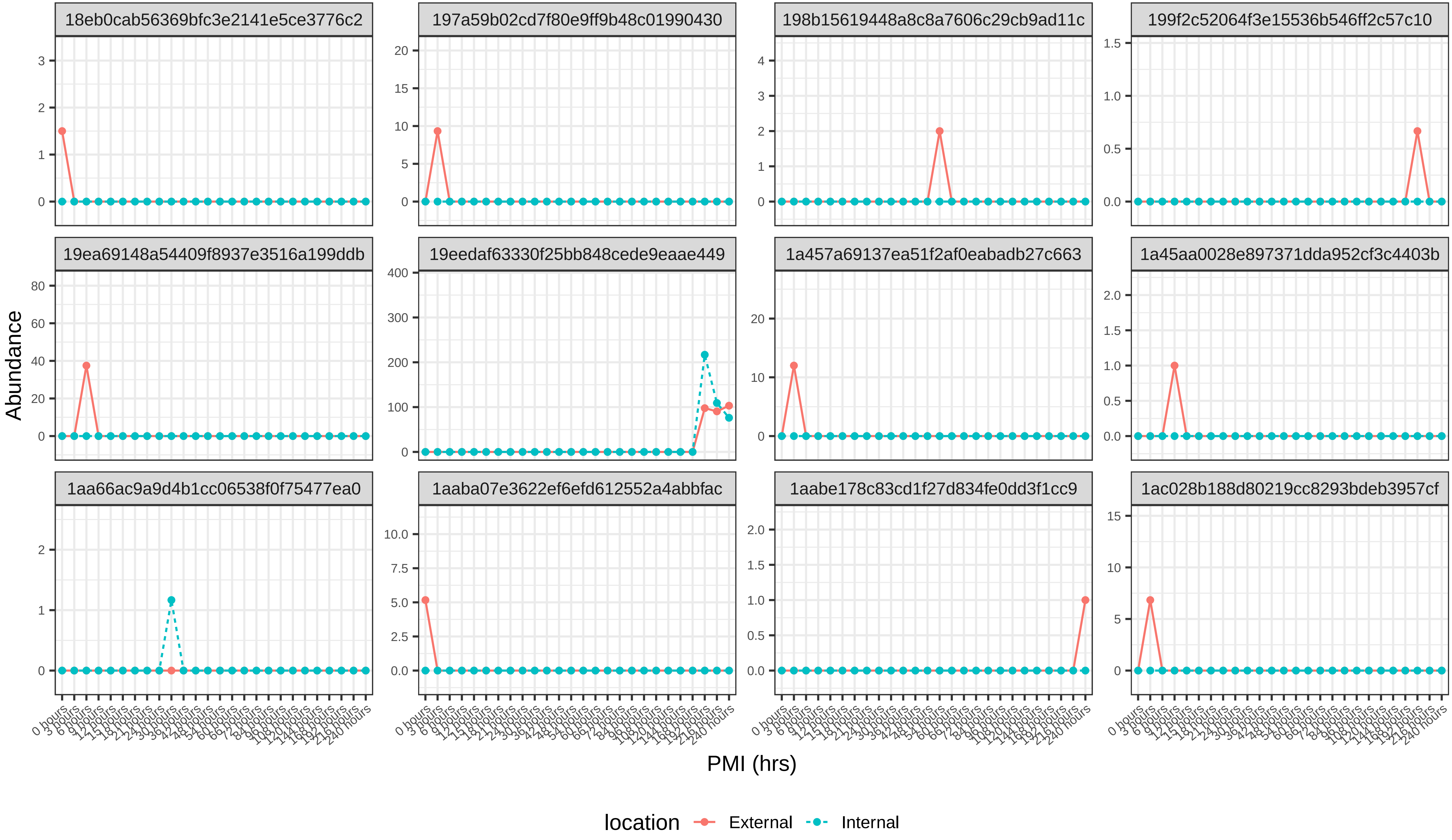

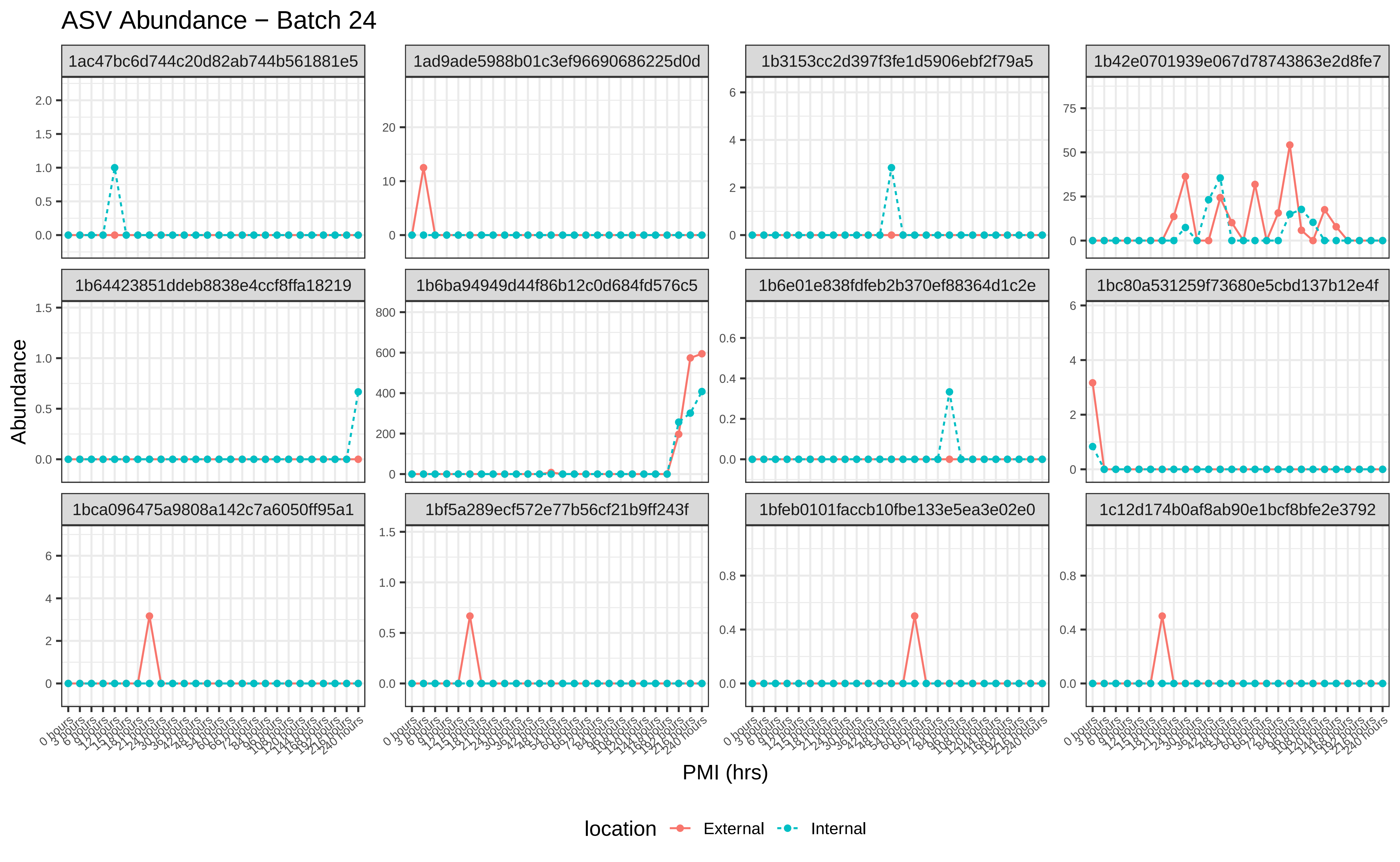
ASV Abundance − Batch 24
1ac47bc6d744c20d82ab744b561881e5
1ad9ade5988b01c3ef96690686225d0d
1b3153cc2d397f3fe1d5906ebf2f79a5
1b42e0701939e067d78743863e2d8fe7
1b64423851ddeb8838e4ccf8ffa18219
1b6ba94949d44f86b12c0d684fd576c5
1b6e01e838fdfeb2b370ef88364d1c2e
1bc80a531259f73680e5cbd137b12e4f
1bca096475a9808a142c7a6050ff95a1
1bf5a289ecf572e77b56cf21b9ff243f
1bfeb0101faccb10fbe133e5ea3e02e0
1c12d174b0af8ab90e1bcf8bfe2e3792
Abundance
PMI (hrs)
location
External
Internal

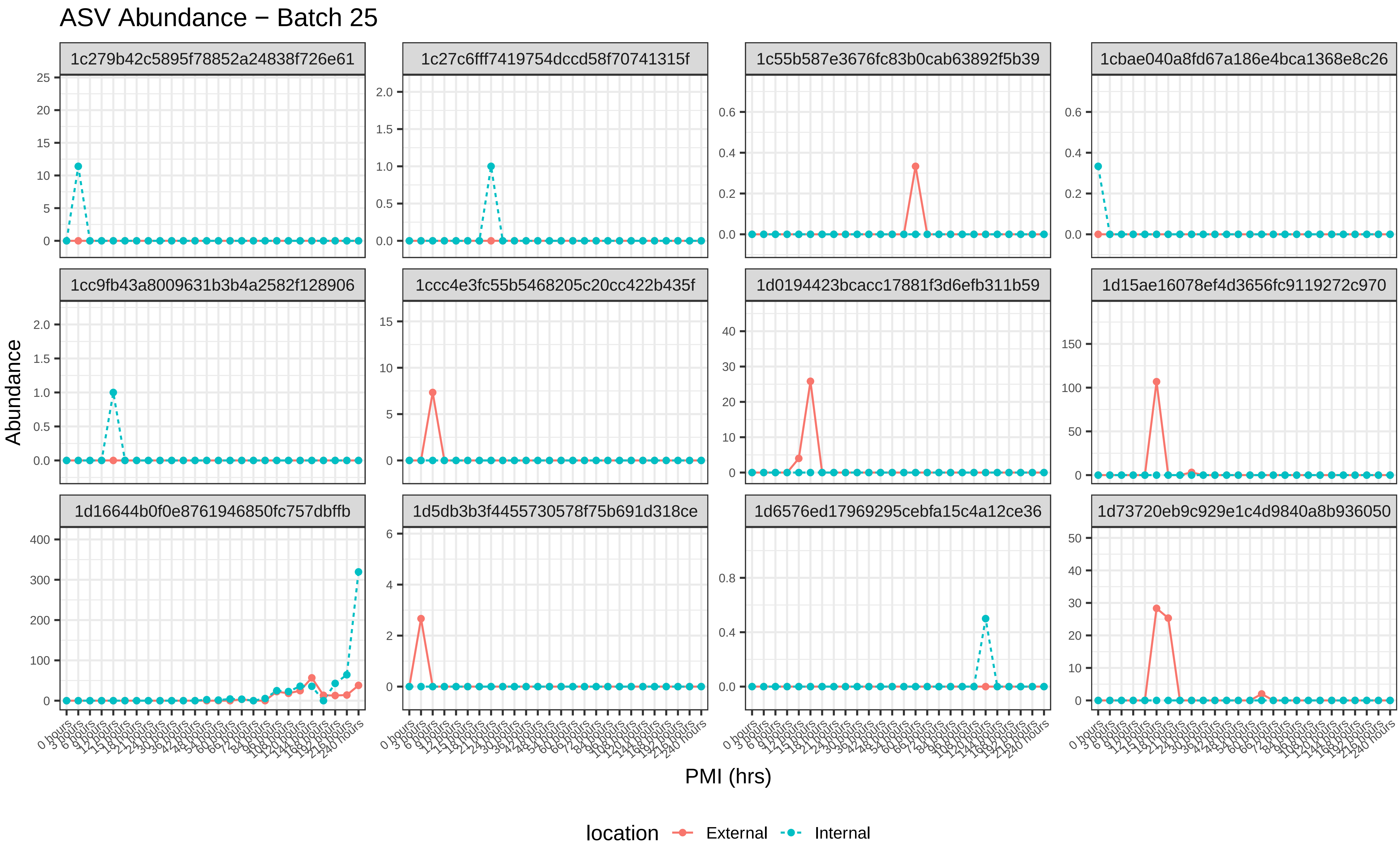
ASV Abundance − Batch 25
1c279b42c5895f78852a24838f726e61
1c27c6fff7419754dccd58f70741315f
1c55b587e3676fc83b0cab63892f5b39
1cbae040a8fd67a186e4bca1368e8c26
1cc9fb43a8009631b3b4a2582f128906
1ccc4e3fc55b5468205c20cc422b435f
1d0194423bcacc17881f3d6efb311b59
1d15ae16078ef4d3656fc9119272c970
1d16644b0f0e8761946850fc757dbffb
1d5db3b3f4455730578f75b691d318ce
1d6576ed17969295cebfa15c4a12ce36
1d73720eb9c929e1c4d9840a8b936050
Abundance
PMI (hrs)
0 hours
3 hours
6 hours
9 hours
12 hours
15 hours
18 hours
21 hours
24 hours
30 hours
36 hours
42 hours
48 hours
54 hours
60 hours
66 hours
72 hours
84 hours
96 hours
108 hours
120 hours
144 hours
168 hours
192 hours
216 hours
240 hours
location
External
Internal

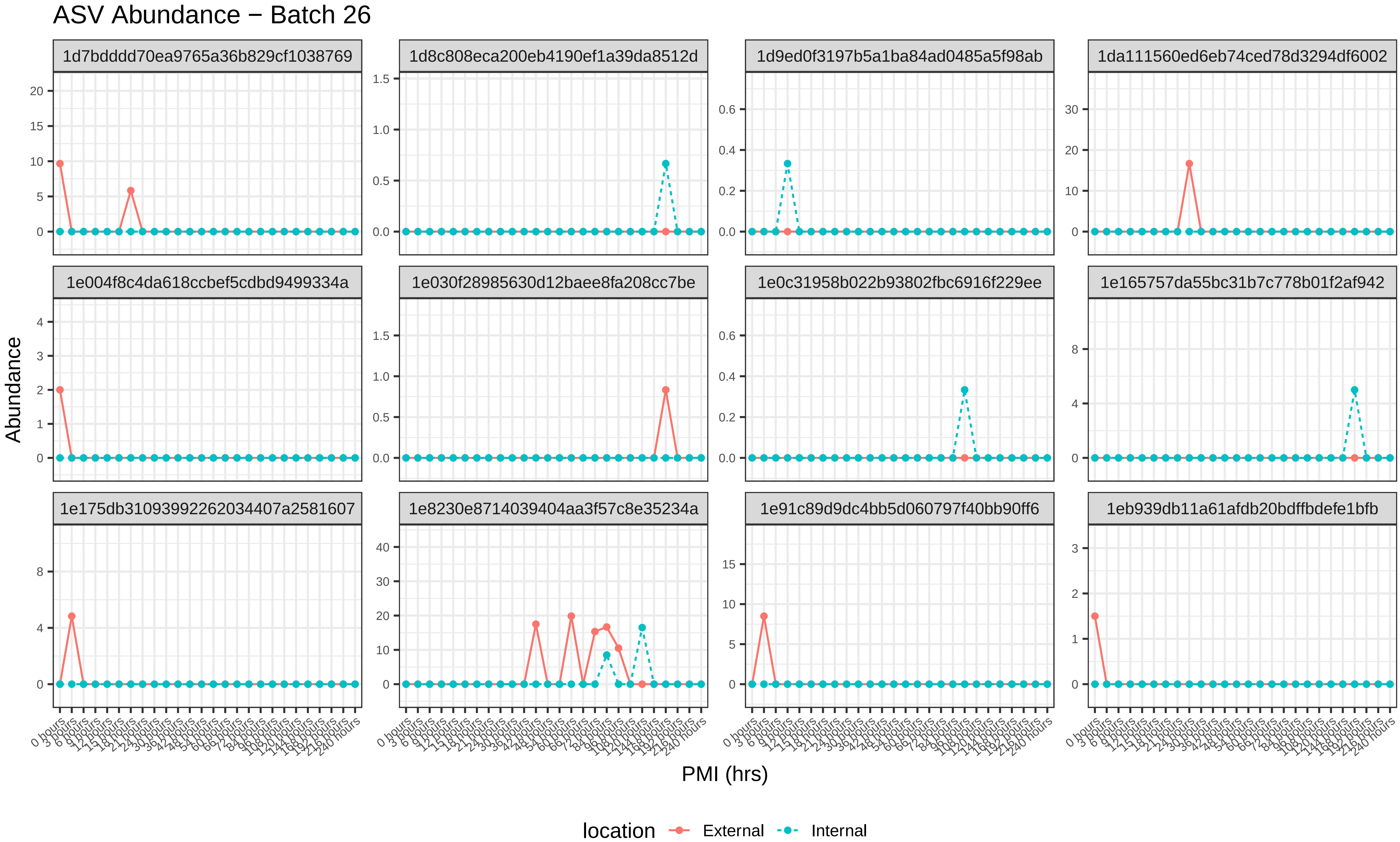
ASV Abundance − Batch 26
1d7bdddd70ea9765a36b829cf1038769
1d8c808eca200eb4190ef1a39da8512d
1d9ed0f3197b5a1ba84ad0485a5f98ab
1da111560ed6eb74ced78d3294df6002
1e004f8c4da618ccbef5cdbd9499334a
1e030f28985630d12baee8fa208cc7be
1e0c31958b022b93802fbc6916f229ee
1e165757da55bc31b7c778b01f2af942
1e175db31093992262034407a2581607
1e8230e8714039404aa3f57c8e35234a
1e91c89d9dc4bb5d060797f40bb90ff6
1eb939db11a61afdb20bdffbdefe1bfb
Abundance
PMI (hrs)
location
External
Internal

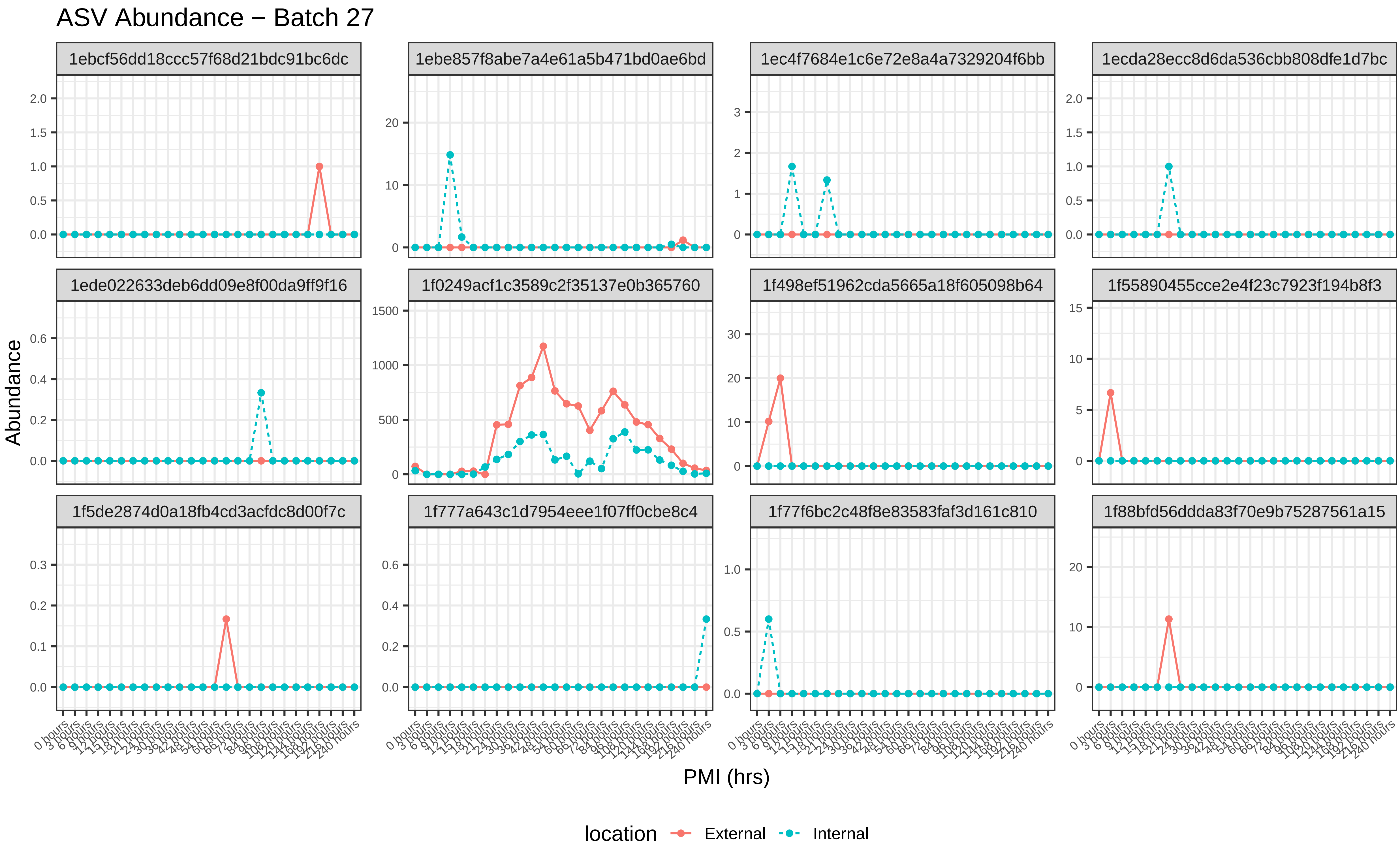
ASV Abundance − Batch 27
1ebcf56dd18ccc57f68d21bdc91bc6dc
1ebe857f8abe7a4e61a5b471bd0ae6bd
1ec4f7684e1c6e72e8a4a7329204f6bb
1ecda28ecc8d6da536cbb808dfe1d7bc
1ede022633deb6dd09e8f00da9ff9f16
1f0249acf1c3589c2f35137e0b365760
1f498ef51962cda5665a18f605098b64
1f55890455cce2e4f23c7923f194b8f3
1f5de2874d0a18fb4cd3acfdc8d00f7c
1f777a643c1d7954eee1f07ff0cbe8c4
1f77f6bc2c48f8e83583faf3d161c810
1f88bfd56ddda83f70e9b75287561a15
Abundance
PMI (hrs)
location
External
Internal

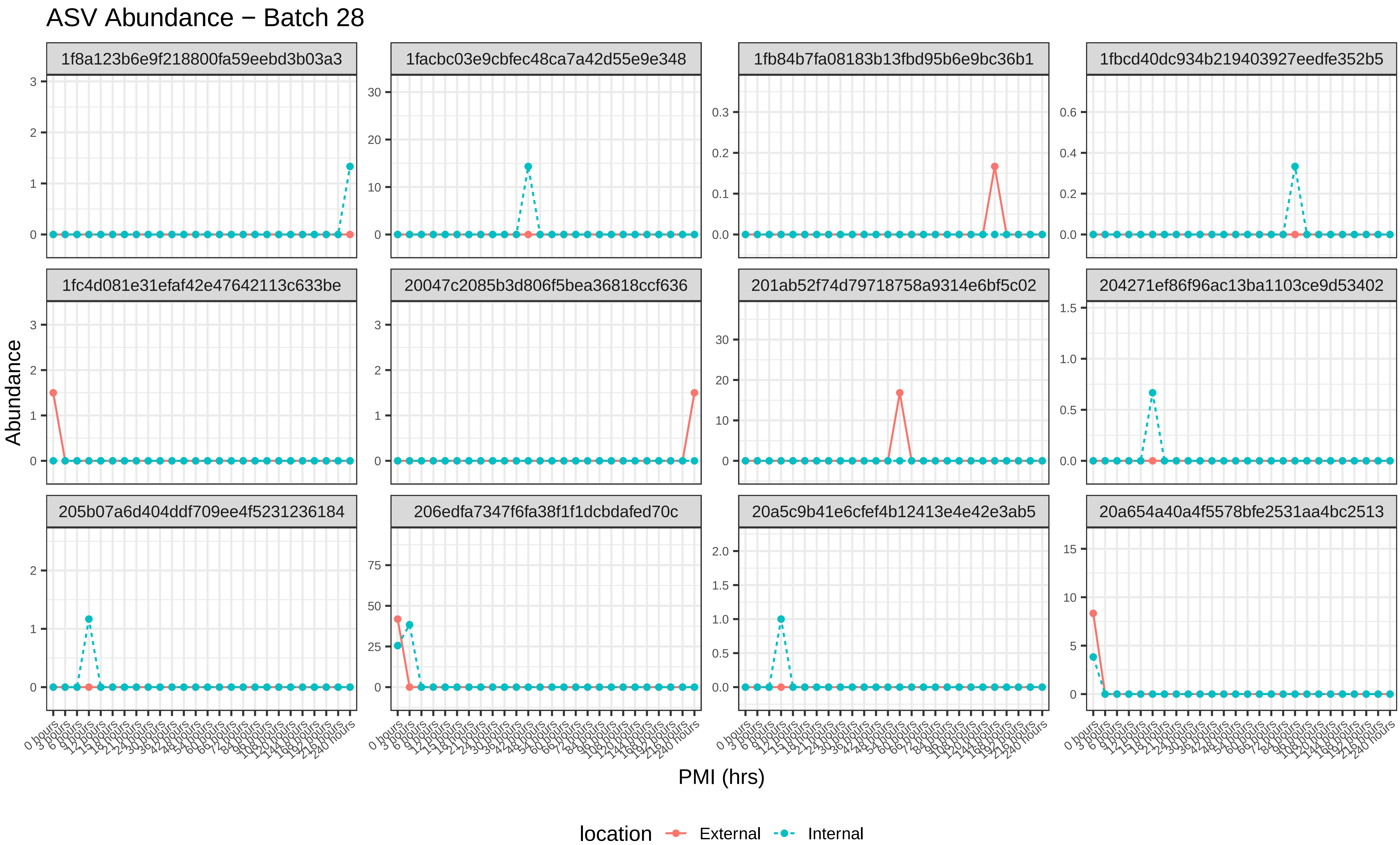
ASV Abundance − Batch 28
1f8a123b6e9f218800fa59eebd3b03a3
1facbc03e9cbfec48ca7a42d55e9e348
1fb84b7fa08183b13fbd95b6e9bc36b1
1fbcd40dc934b219403927eedfe352b5
1fc4d081e31efaf42e47642113c633be
20047c2085b3d806f5bea36818ccf636
201ab52f74d79718758a9314e6bf5c02
204271ef86f96ac13ba1103ce9d53402
205b07a6d404ddf709ee4f5231236184
206edfa7347f6fa38f1f1dcbdafed70c
20a5c9b41e6cfef4b12413e4e42e3ab5
20a654a40a4f5578bfe2531aa4bc2513
Abundance
PMI (hrs)
location
External
Internal

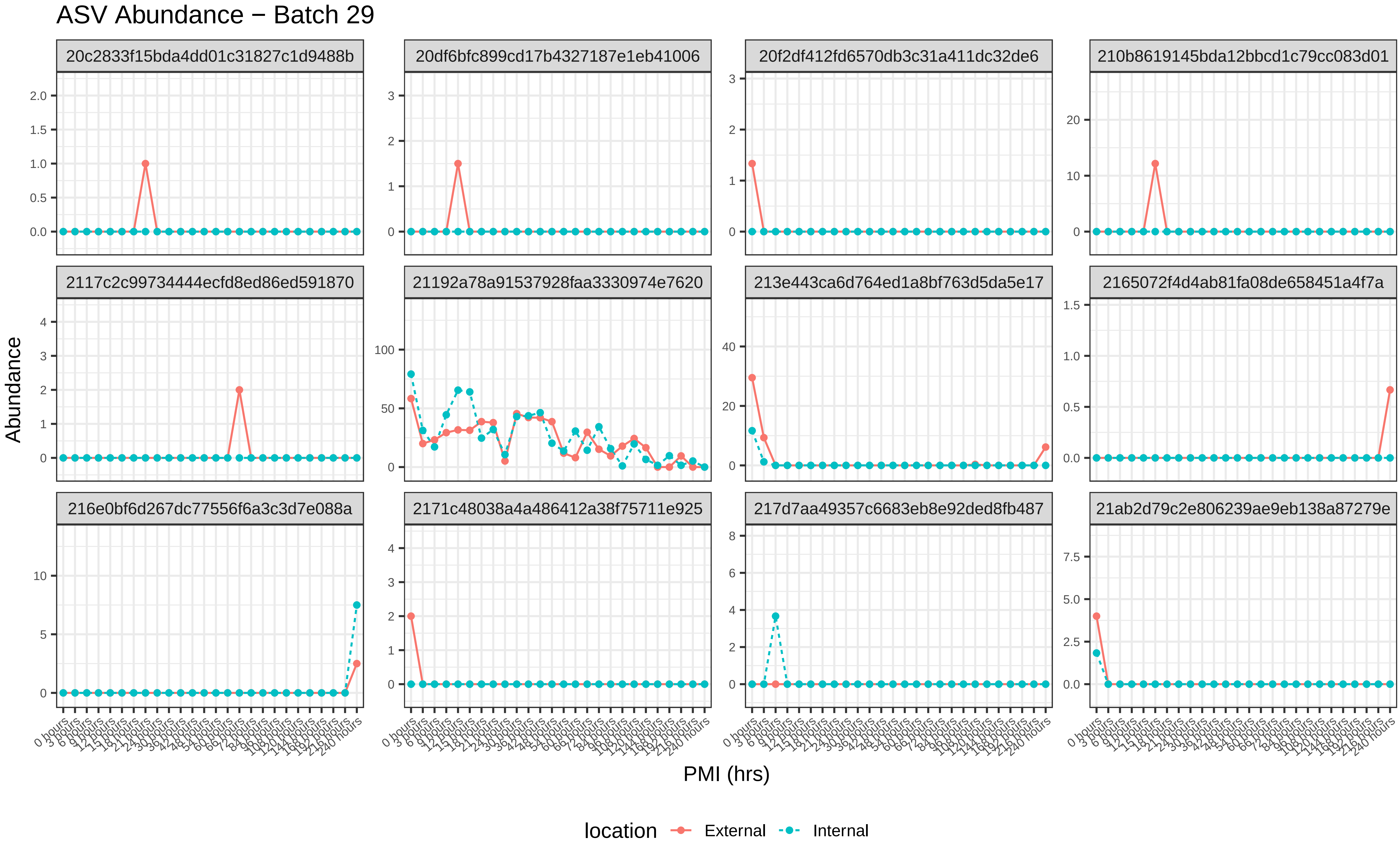

ASV Abundance − Batch 29
20c2833f15bda4dd01c31827c1d9488b
20df6bfc899cd17b4327187e1eb41006
20f2df412fd6570db3c31a411dc32de6
210b8619145bda12bbcd1c79cc083d01
2117c2c99734444ecfd8ed86ed591870
21192a78a91537928faa3330974e7620
213e443ca6d764ed1a8bf763d5da5e17
2165072f4d4ab81fa08de658451a4f7a
216e0bf6d267dc77556f6a3c3d7e088a
2171c48038a4a486412a38f75711e925
217d7aa49357c6683eb8e92ded8fb487
21ab2d79c2e806239ae9eb138a87279e
Abundance
PMI (hrs)
location
External
Internal

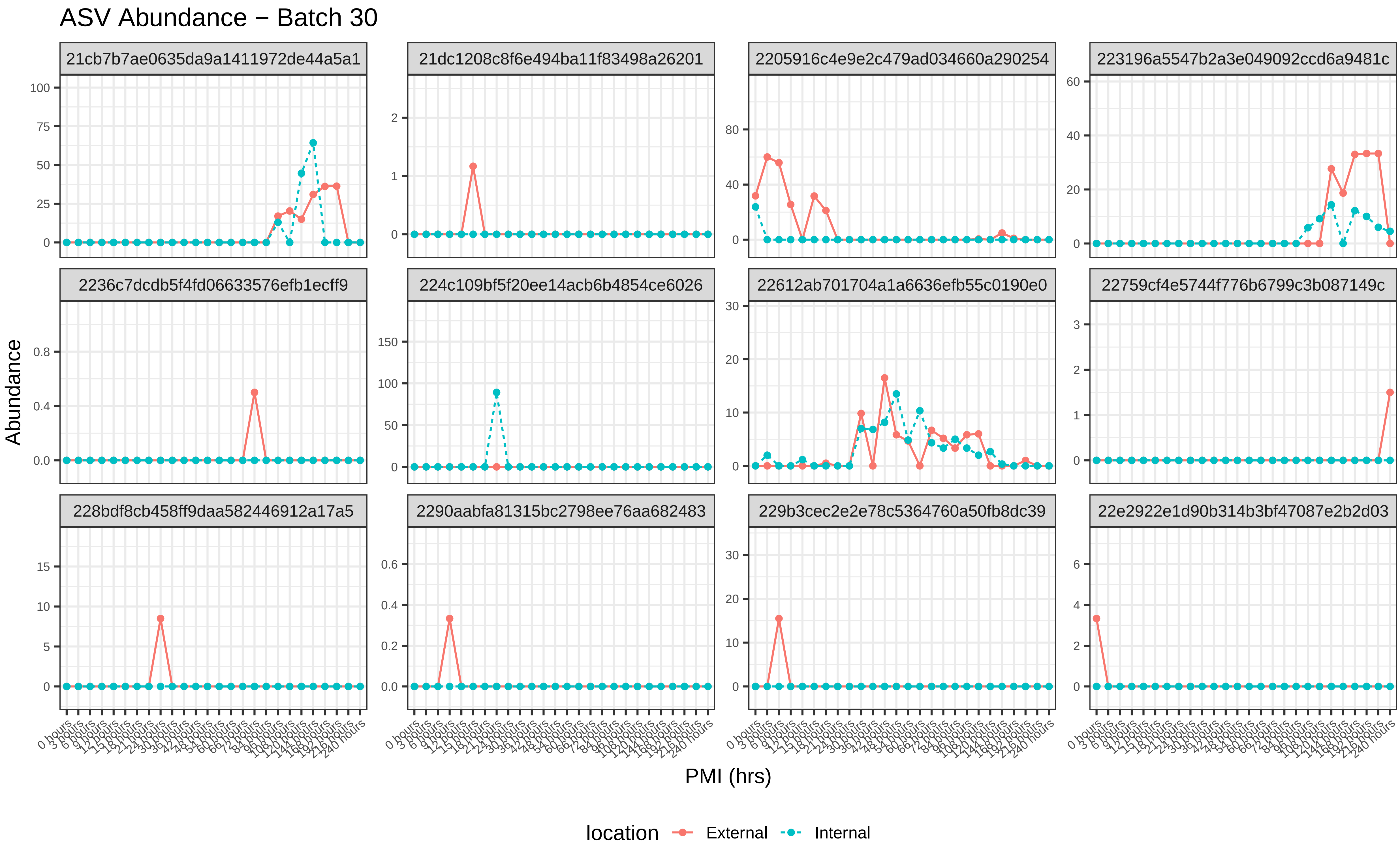
ASV Abundance − Batch 30
21cb7b7ae0635da9a1411972de44a5a1
21dc1208c8f6e494ba11f83498a26201
2205916c4e9e2c479ad034660a290254
223196a5547b2a3e049092ccd6a9481c
2236c7dcdb5f4fd06633576efb1ecff9
224c109bf5f20ee14acb6b4854ce6026
22612ab701704a1a6636efb55c0190e0
22759cf4e5744f776b6799c3b087149c
228bdf8cb458ff9daa582446912a17a5
2290aabfa81315bc2798ee76aa682483
229b3cec2e2e78c5364760a50fb8dc39
22e2922e1d90b314b3bf47087e2b2d03
Abundance
PMI (hrs)
location
External
Internal

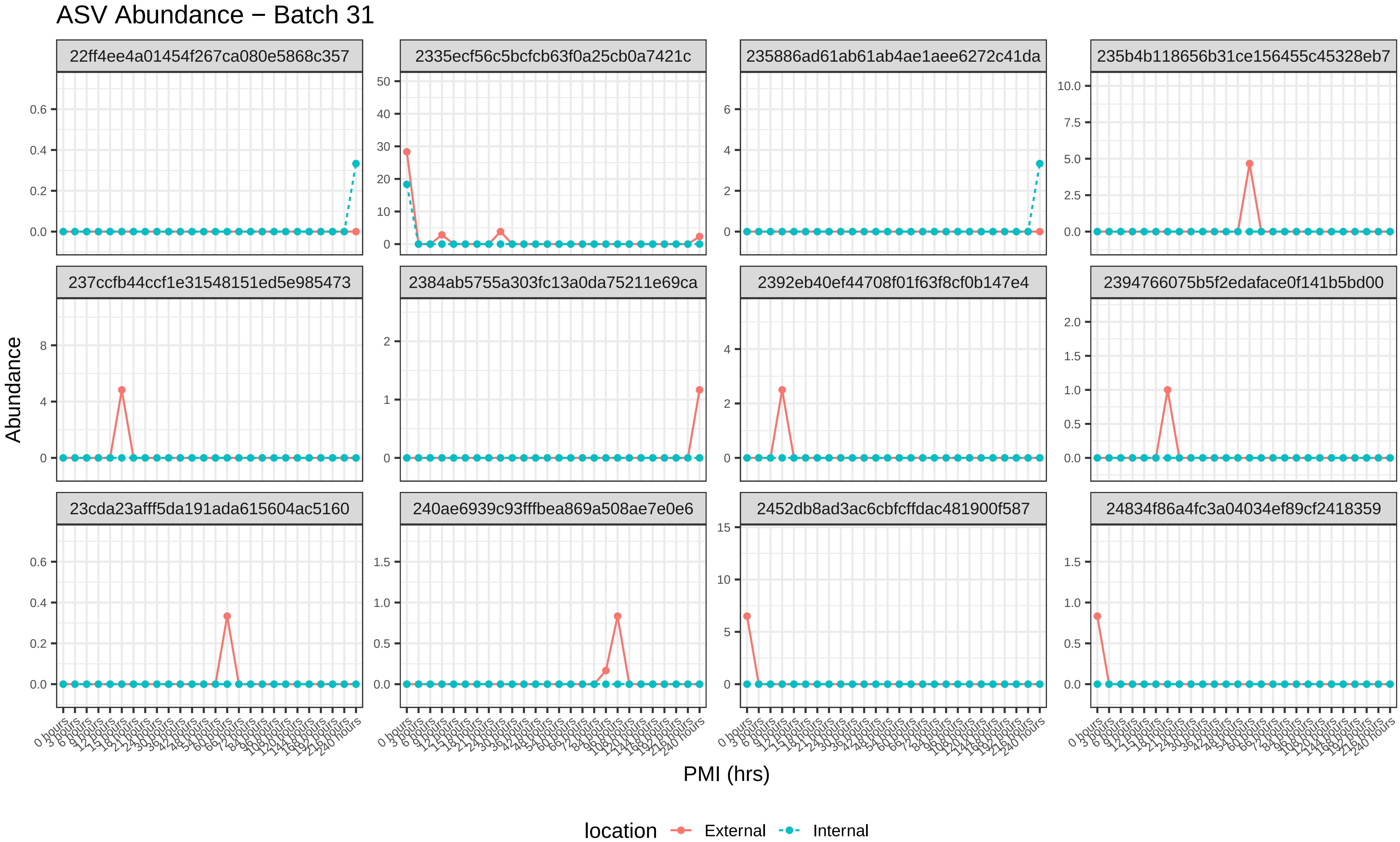

ASV Abundance − Batch 31
22ff4ee4a01454f267ca080e5868c357
2335ecf56c5bcfcb63f0a25cb0a7421c
235886ad61ab61ab4ae1aee6272c41da
235b4b118656b31ce156455c45328eb7
237ccfb44ccf1e31548151ed5e985473
2384ab5755a303fc13a0da75211e69ca
2392eb40ef44708f01f63f8cf0b147e4
2394766075b5f2edaface0f141b5bd00
23cda23afff5da191ada615604ac5160
240ae6939c93fffbea869a508ae7e0e6
2452db8ad3ac6cbfcffdac481900f587
24834f86a4fc3a04034ef89cf2418359
Abundance
PMI (hrs)
location
External
Internal

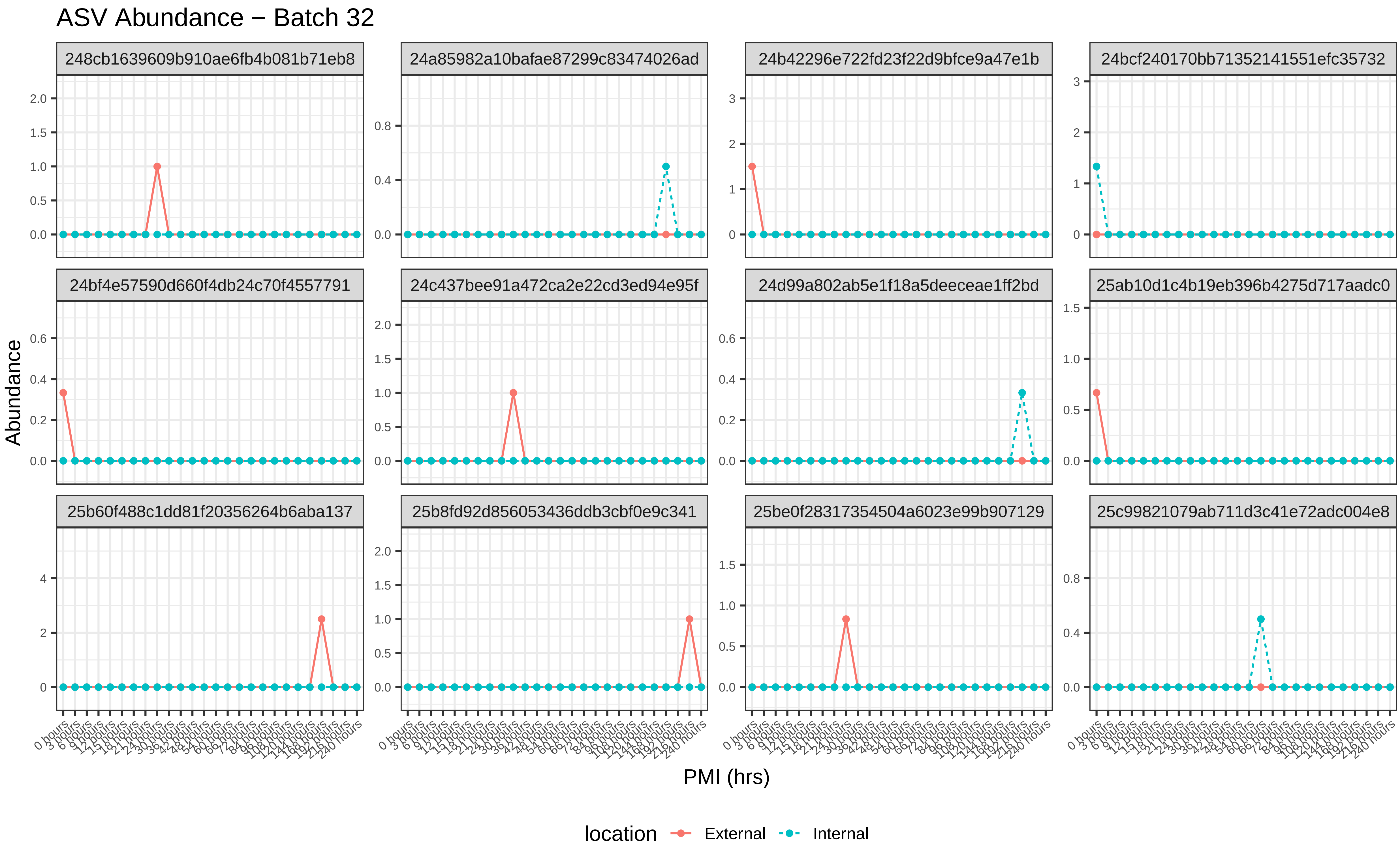
ASV Abundance − Batch 32
248cb1639609b910ae6fb4b081b71eb8
24a85982a10bafae87299c83474026ad
24b42296e722fd23f22d9bfce9a47e1b
24bcf240170bb71352141551efc35732
24bf4e57590d660f4db24c70f4557791
24c437bee91a472ca2e22cd3ed94e95f
24d99a802ab5e1f18a5deeceae1ff2bd
25ab10d1c4b19eb396b4275d717aadc0
25b60f488c1dd81f20356264b6aba137
25b8fd92d856053436ddb3cbf0e9c341
25be0f28317354504a6023e99b907129
25c99821079ab711d3c41e72adc004e8
Abundance
PMI (hrs)
location
External
Internal

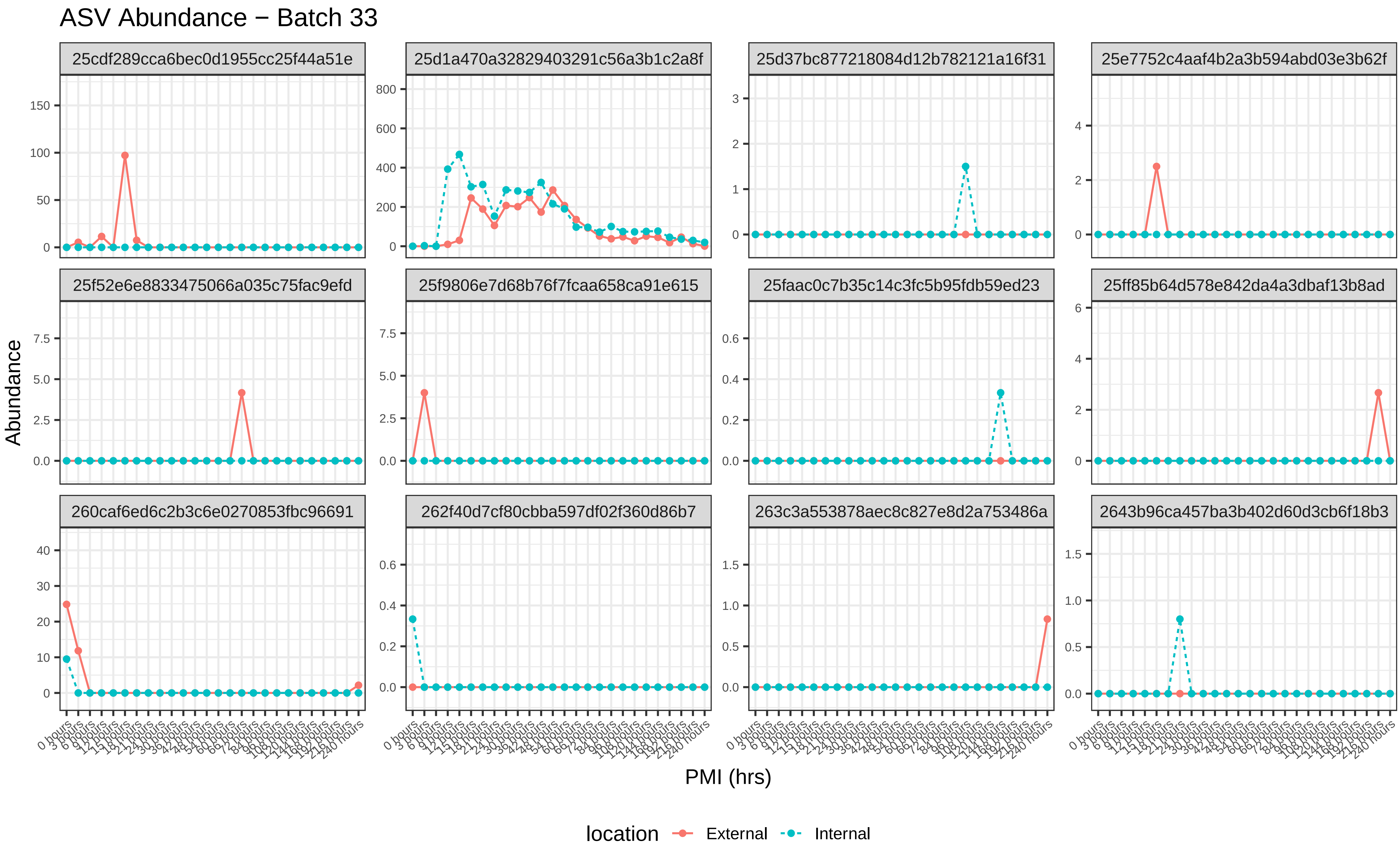

ASV Abundance − Batch 33
25cdf289cca6bec0d1955cc25f44a51e
25d1a470a32829403291c56a3b1c2a8f
25d37bc877218084d12b782121a16f31
25e7752c4aaf4b2a3b594abd03e3b62f
25f52e6e8833475066a035c75fac9efd
25f9806e7d68b76f7fcaa658ca91e615
25faac0c7b35c14c3fc5b95fdb59ed23
25ff85b64d578e842da4a3dbaf13b8ad
260caf6ed6c2b3c6e0270853fbc96691
262f40d7cf80cbba597df02f360d86b7
263c3a553878aec8c827e8d2a753486a
2643b96ca457ba3b402d60d3cb6f18b3
Abundance
PMI (hrs)
location
External
Internal

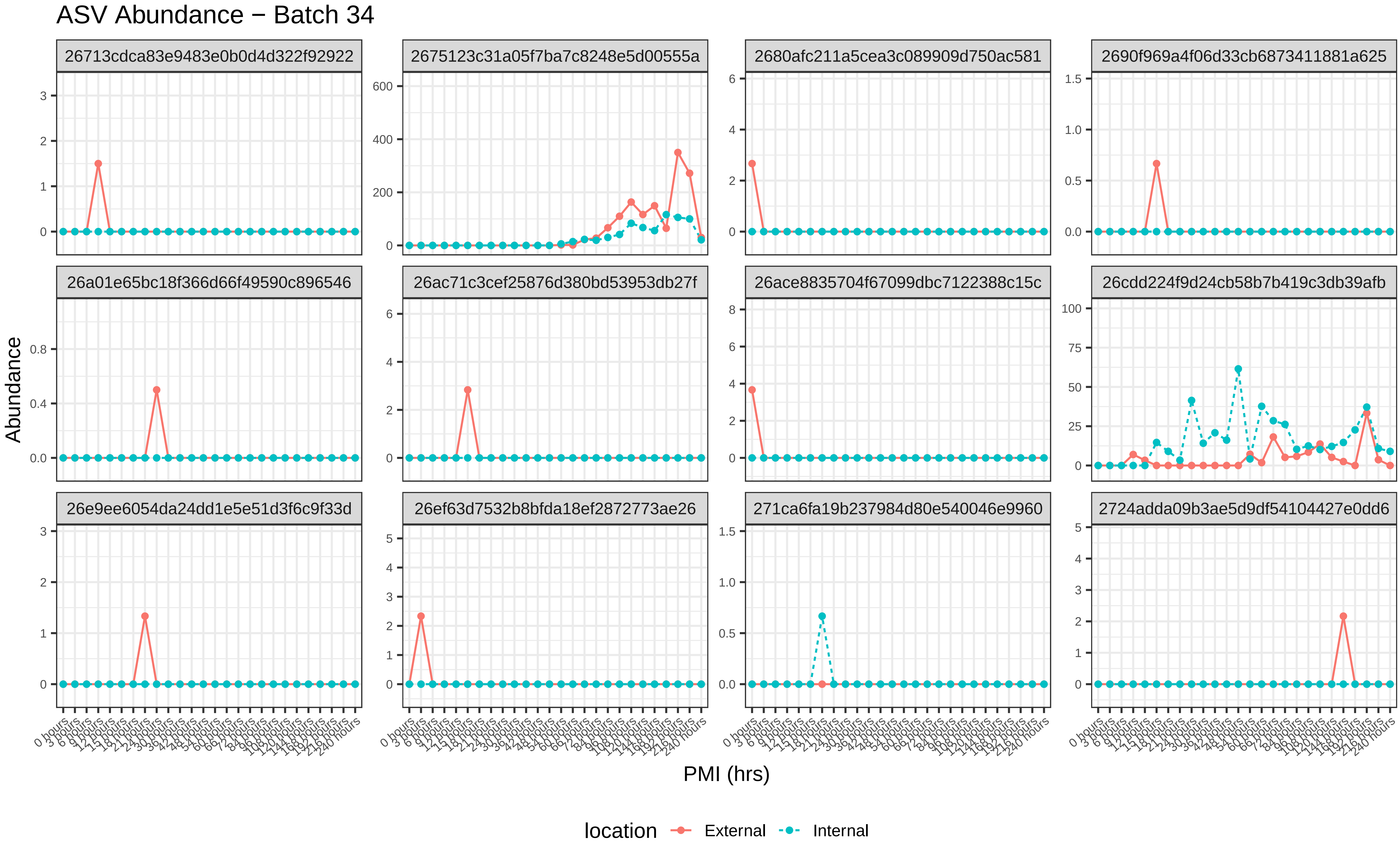
ASV Abundance − Batch 34
26713cdca83e9483e0b0d4d322f92922
2675123c31a05f7ba7c8248e5d00555a
2680afc211a5cea3c089909d750ac581
2690f969a4f06d33cb6873411881a625
26a01e65bc18f366d66f49590c896546
26ac71c3cef25876d380bd53953db27f
26ace8835704f67099dbc7122388c15c
26cdd224f9d24cb58b7b419c3db39afb
26e9ee6054da24dd1e5e51d3f6c9f33d
26ef63d7532b8bfda18ef2872773ae26
271ca6fa19b237984d80e540046e9960
2724adda09b3ae5d9df54104427e0dd6
Abundance
PMI (hrs)
location
External
Internal

ASV Abundance − Batch 35

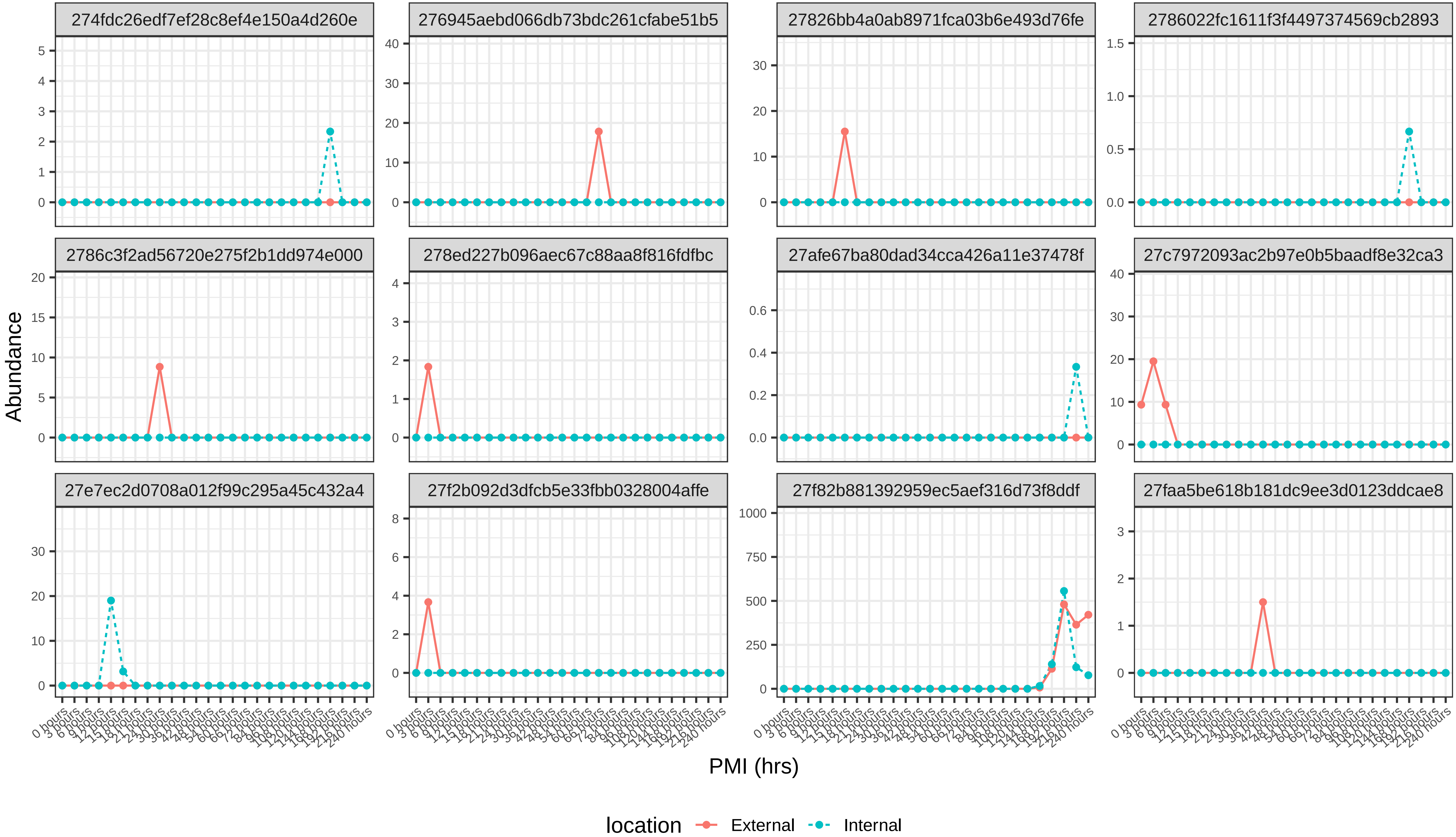

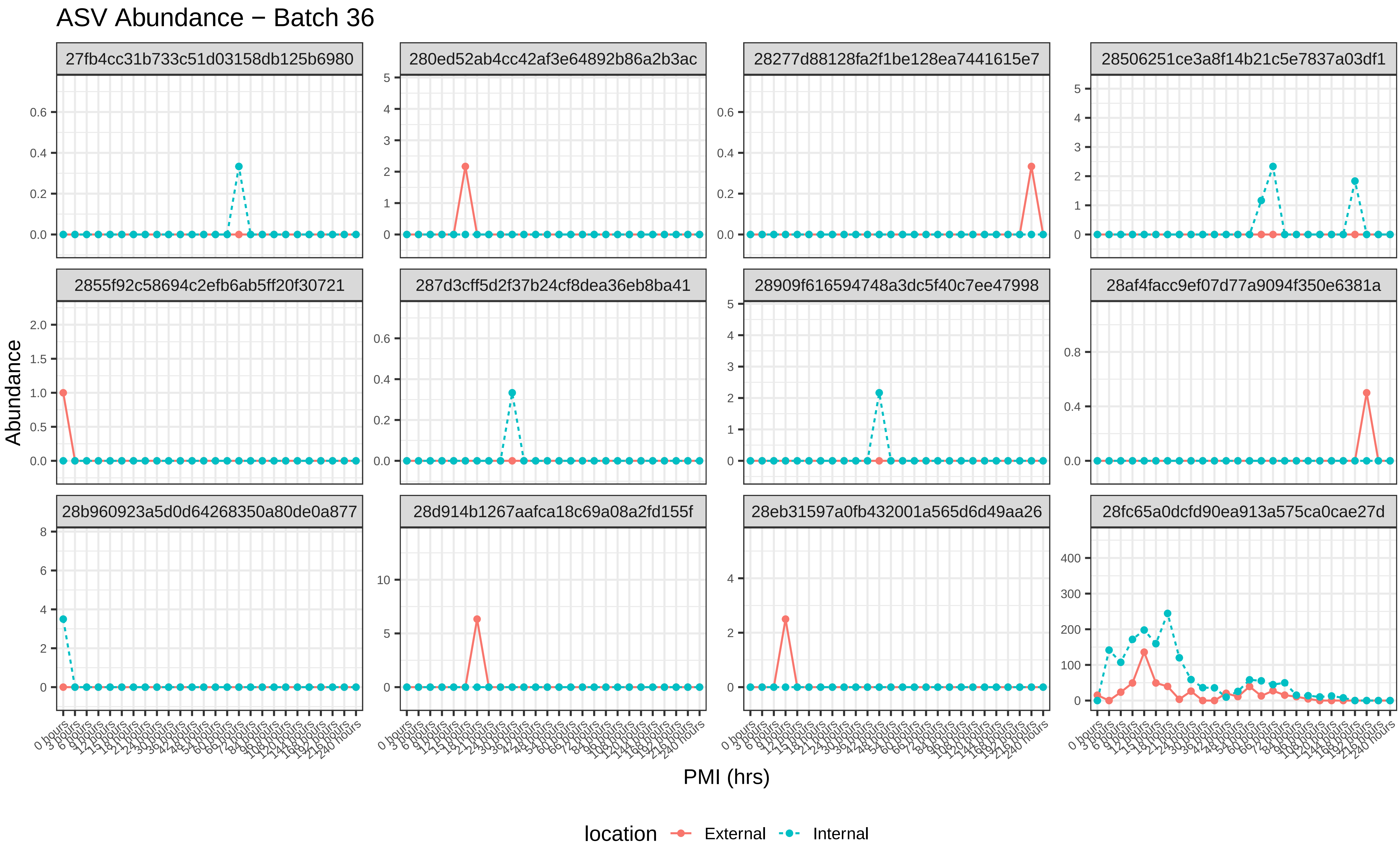

ASV Abundance − Batch 36
27fb4cc31b733c51d03158db125b6980
280ed52ab4cc42af3e64892b86a2b3ac
28277d88128fa2f1be128ea7441615e7
28506251ce3a8f14b21c5e7837a03df1
2855f92c58694c2efb6ab5ff20f30721
287d3cff5d2f37b24cf8dea36eb8ba41
28909f616594748a3dc5f40c7ee47998
28af4facc9ef07d77a9094f350e6381a
28b960923a5d0d64268350a80de0a877
28d914b1267aafca18c69a08a2fd155f
28eb31597a0fb432001a565d6d49aa26
28fc65a0dcfd90ea913a575ca0cae27d
Abundance
PMI (hrs)
location
External
Internal

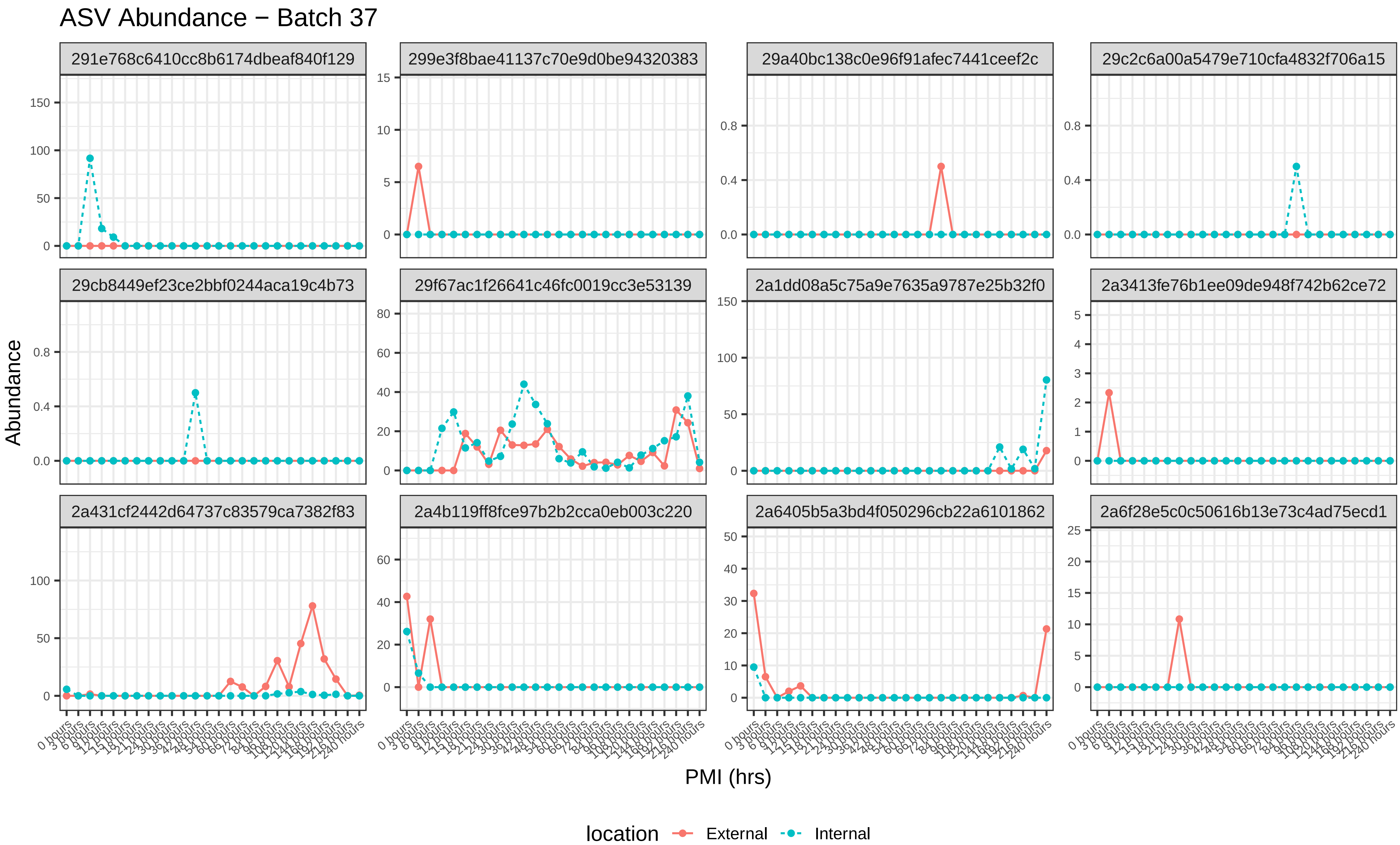

ASV Abundance − Batch 37
291e768c6410cc8b6174dbeaf840f129
299e3f8bae41137c70e9d0be94320383
29a40bc138c0e96f91afec7441ceef2c
29c2c6a00a5479e710cfa4832f706a15
29cb8449ef23ce2bbf0244aca19c4b73
29f67ac1f26641c46fc0019cc3e53139
2a1dd08a5c75a9e7635a9787e25b32f0
2a3413fe76b1ee09de948f742b62ce72
2a431cf2442d64737c83579ca7382f83
2a4b119ff8fce97b2b2cca0eb003c220
2a6405b5a3bd4f050296cb22a6101862
2a6f28e5c0c50616b13e73c4ad75ecd1
Abundance
PMI (hrs)
location
External
Internal

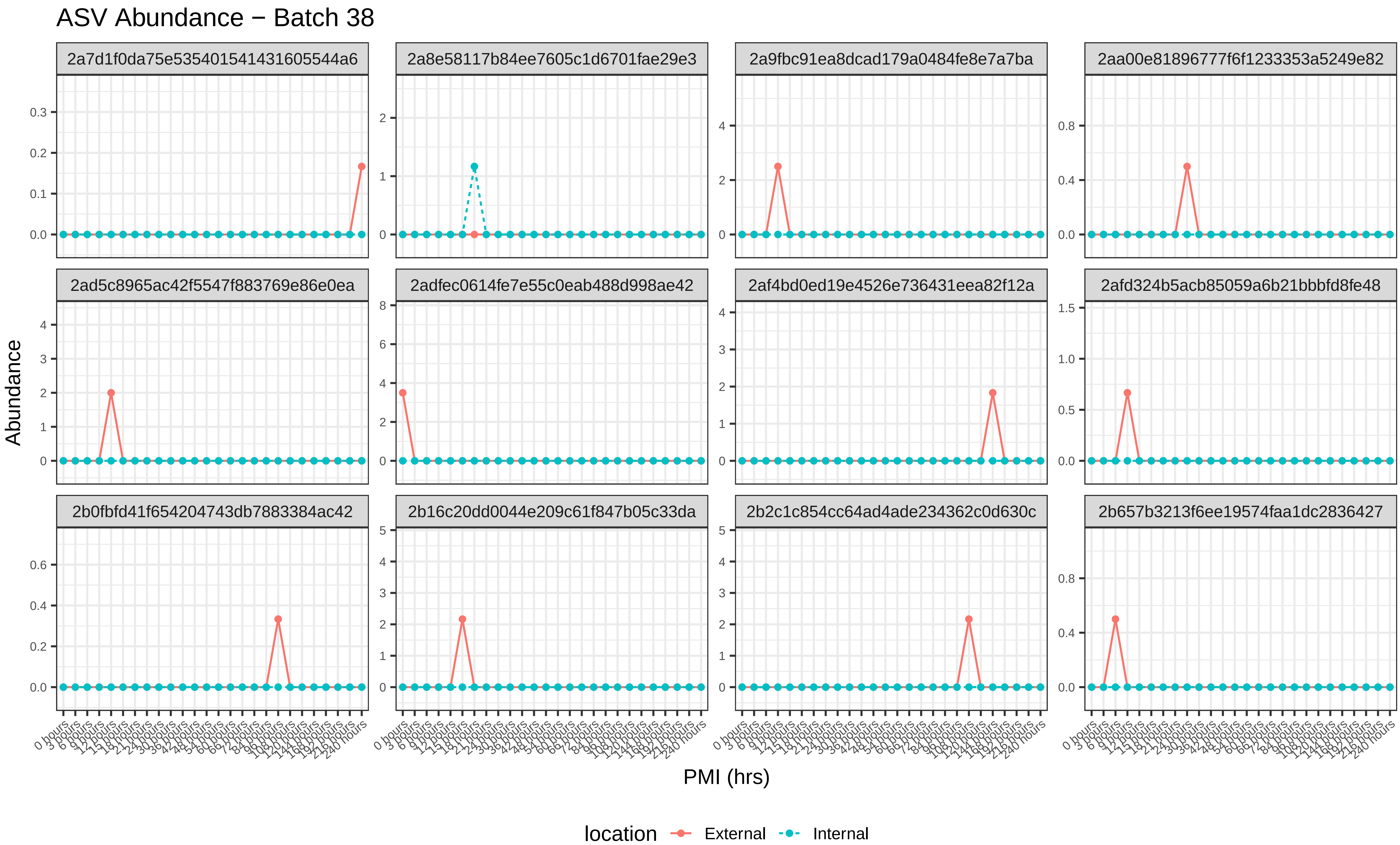
ASV Abundance − Batch 38
2a7d1f0da75e535401541431605544a6
2a8e58117b84ee7605c1d6701fae29e3
2a9fbc91ea8dcad179a0484fe8e7a7ba
2aa00e81896777f6f1233353a5249e82
2ad5c8965ac42f5547f883769e86e0ea
2adfec0614fe7e55c0eab488d998ae42
2af4bd0ed19e4526e736431eea82f12a
2afd324b5acb85059a6b21bbbfd8fe48
2b0fbfd41f654204743db7883384ac42
2b16c20dd0044e209c61f847b05c33da
2b2c1c854cc64ad4ade234362c0d630c
2b657b3213f6ee19574faa1dc2836427
Abundance
PMI (hrs)
location
External
Internal

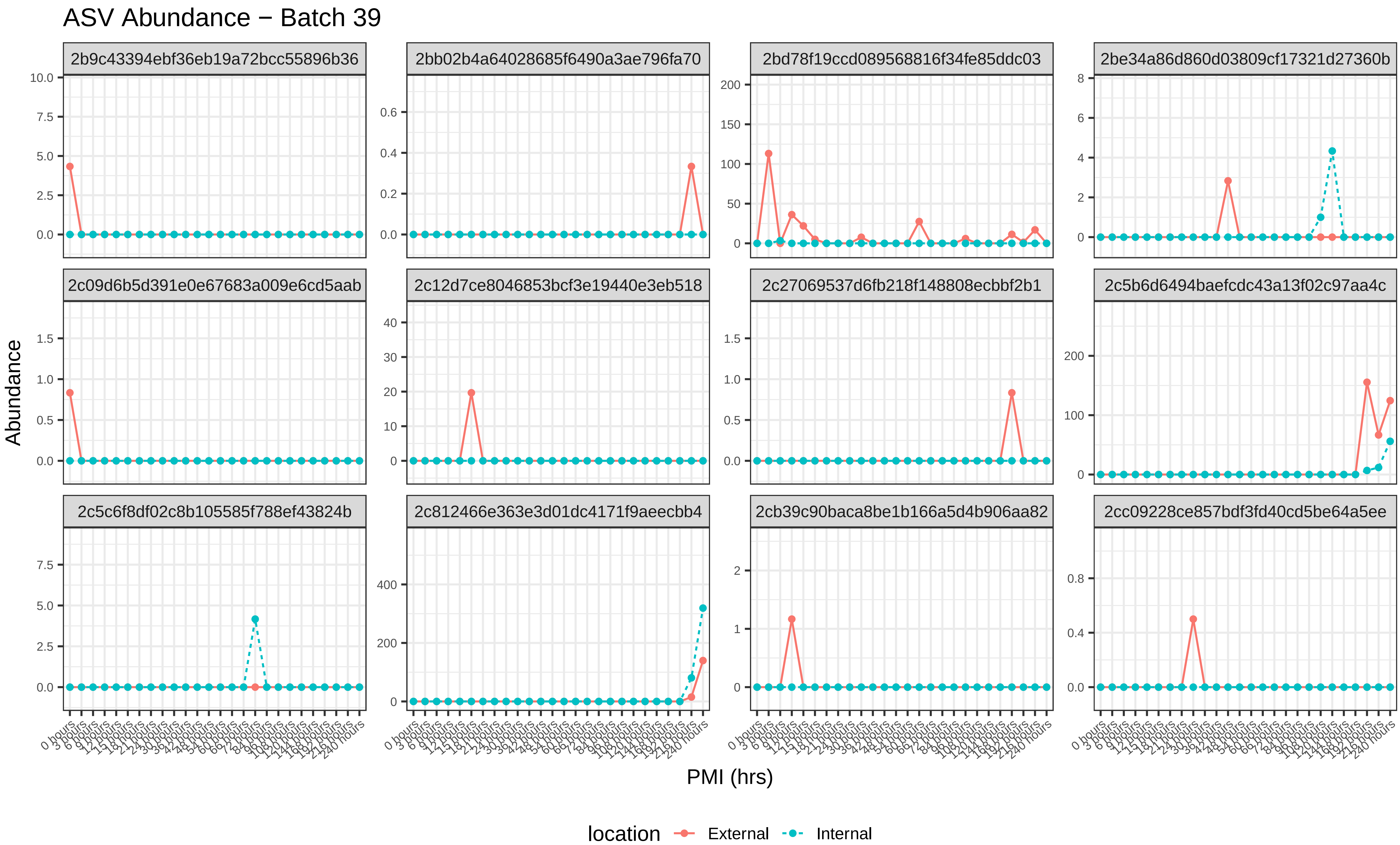

ASV Abundance − Batch 39
2b9c43394ebf36eb19a72bcc55896b36
2bb02b4a64028685f6490a3ae796fa70
2bd78f19ccd089568816f34fe85ddc03
2be34a86d860d03809cf17321d27360b
2c09d6b5d391e0e67683a009e6cd5aab
2c12d7ce8046853bcf3e19440e3eb518
2c27069537d6fb218f148808ecbbf2b1
2c5b6d6494baefcdc43a13f02c97aa4c
2c5c6f8df02c8b105585f788ef43824b
2c812466e363e3d01dc4171f9aeecbb4
2cb39c90baca8be1b166a5d4b906aa82
2cc09228ce857bdf3fd40cd5be64a5ee
Abundance
PMI (hrs)
location
External
Internal

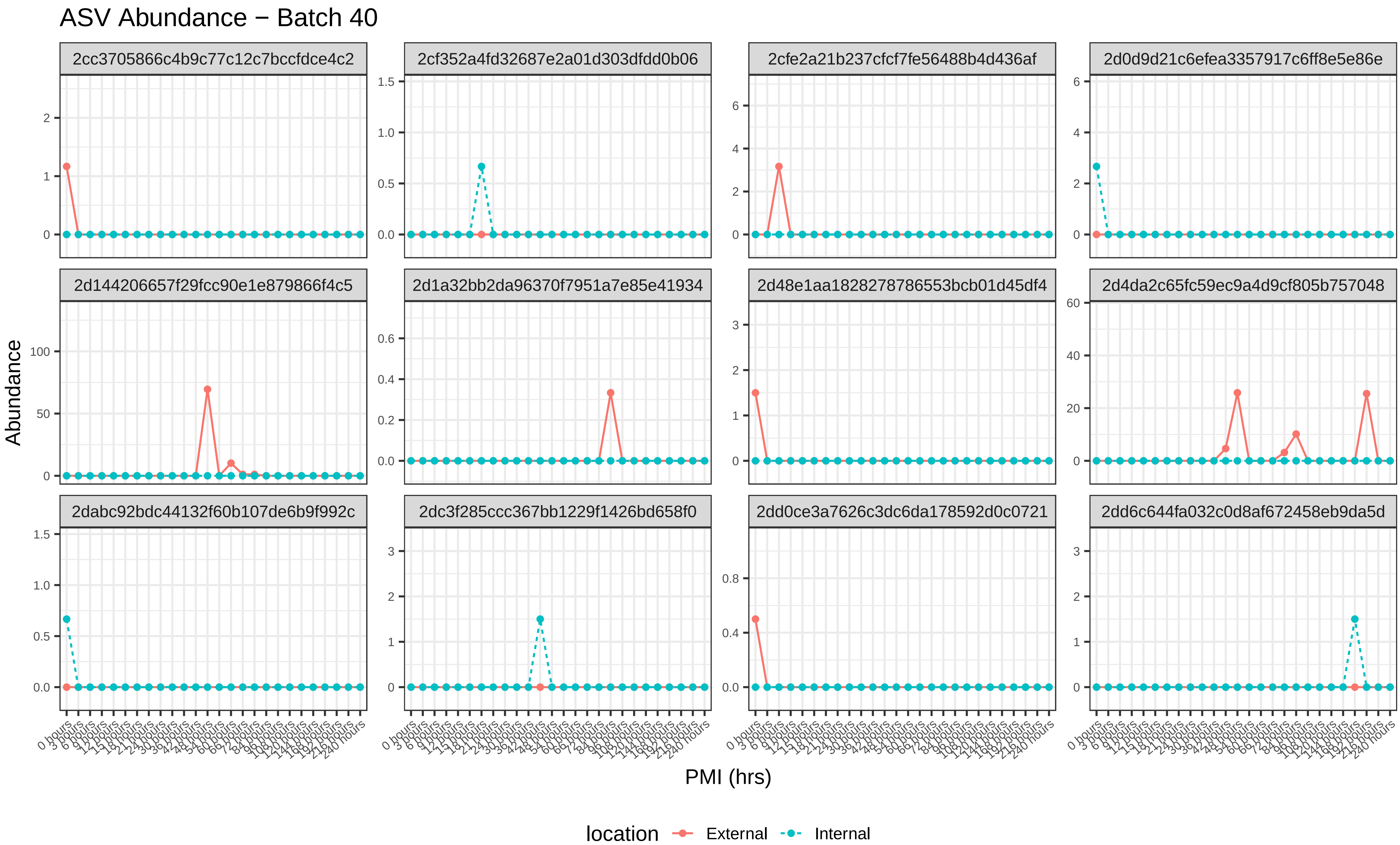

ASV Abundance − Batch 40
2cc3705866c4b9c77c12c7bccfdce4c2
2cf352a4fd32687e2a01d303dfdd0b06
2cfe2a21b237cfcf7fe56488b4d436af
2d0d9d21c6efea3357917c6ff8e5e86e
2d144206657f29fcc90e1e879866f4c5
2d1a32bb2da96370f7951a7e85e41934
2d48e1aa1828278786553bcb01d45df4
2d4da2c65fc59ec9a4d9cf805b757048
2dabc92bdc44132f60b107de6b9f992c
2dc3f285ccc367bb1229f1426bd658f0
2dd0ce3a7626c3dc6da178592d0c0721
2dd6c644fa032c0d8af672458eb9da5d
Abundance
PMI (hrs)
location
External
Internal

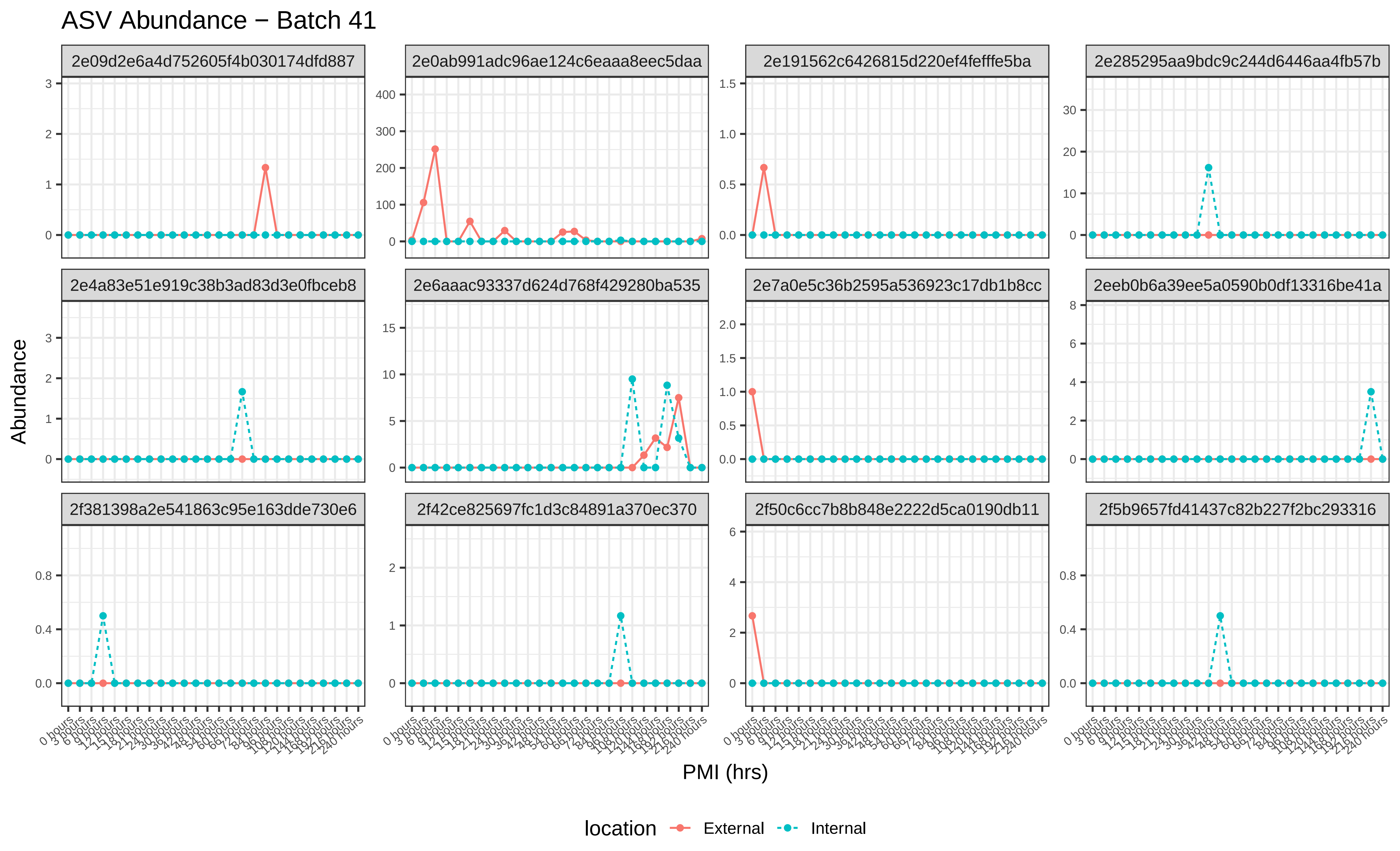
ASV Abundance − Batch 41
2e09d2e6a4d752605f4b030174dfd887
2e0ab991adc96ae124c6eaaa8eec5daa
2e191562c6426815d220ef4fefffe5ba
2e285295aa9bdc9c244d6446aa4fb57b
2e4a83e51e919c38b3ad83d3e0fbceb8
2e6aaac93337d624d768f429280ba535
2e7a0e5c36b2595a536923c17db1b8cc
2eeb0b6a39ee5a0590b0df13316be41a
2f381398a2e541863c95e163dde730e6
2f42ce825697fc1d3c84891a370ec370
2f50c6cc7b8b848e2222d5ca0190db11
2f5b9657fd41437c82b227f2bc293316
Abundance
PMI (hrs)
location
External
Internal

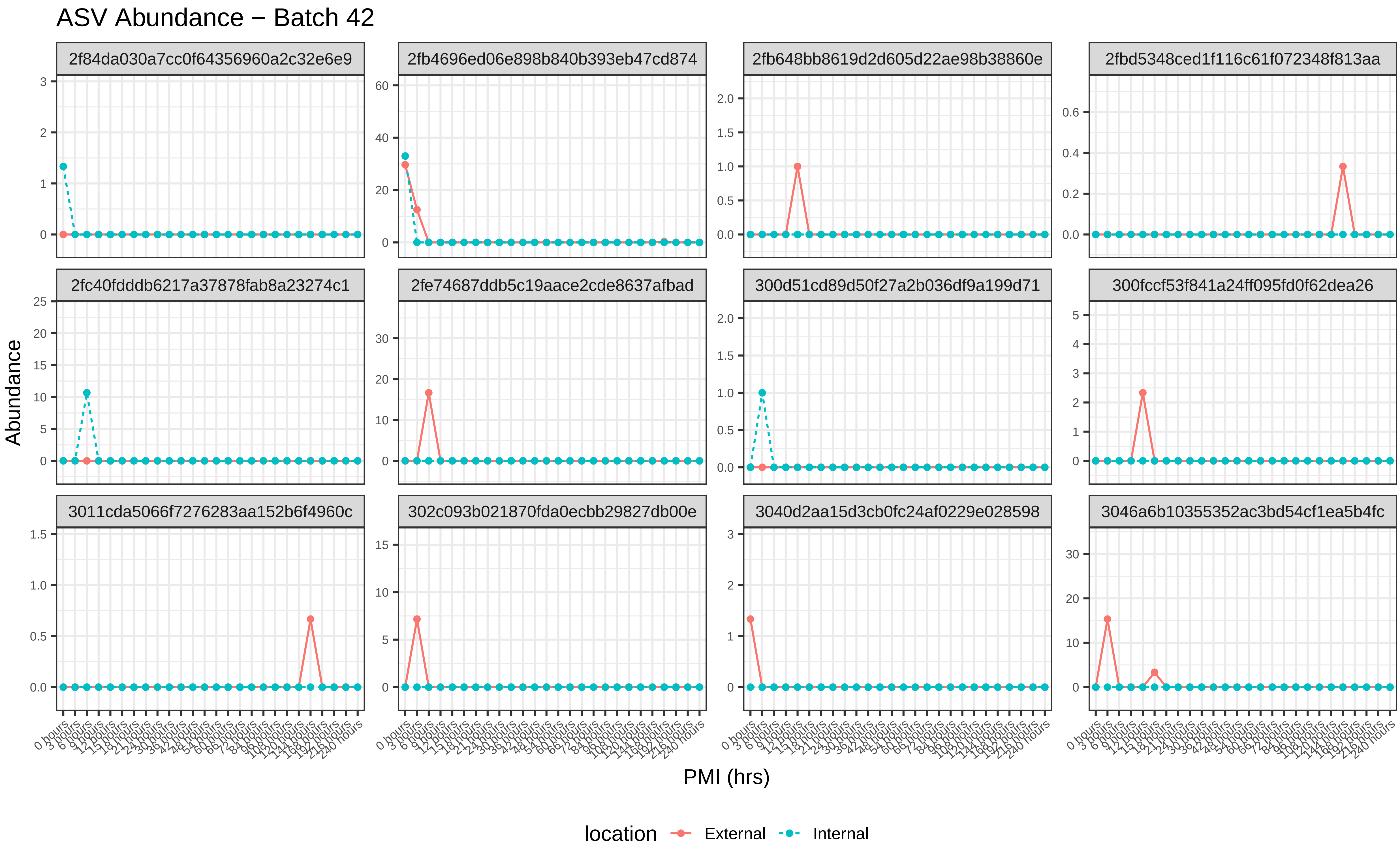
ASV Abundance − Batch 42
2f84da030a7cc0f64356960a2c32e6e9
2fb4696ed06e898b840b393eb47cd874
2fb648bb8619d2d605d22ae98b38860e
2fbd5348ced1f116c61f072348f813aa
2fc40fdddb6217a37878fab8a23274c1
2fe74687ddb5c19aace2cde8637afbad
300d51cd89d50f27a2b036df9a199d71
300fccf53f841a24ff095fd0f62dea26
3011cda5066f7276283aa152b6f4960c
302c093b021870fda0ecbb29827db00e
3040d2aa15d3cb0fc24af0229e028598
3046a6b10355352ac3bd54cf1ea5b4fc
Abundance
PMI (hrs)
location
External
Internal

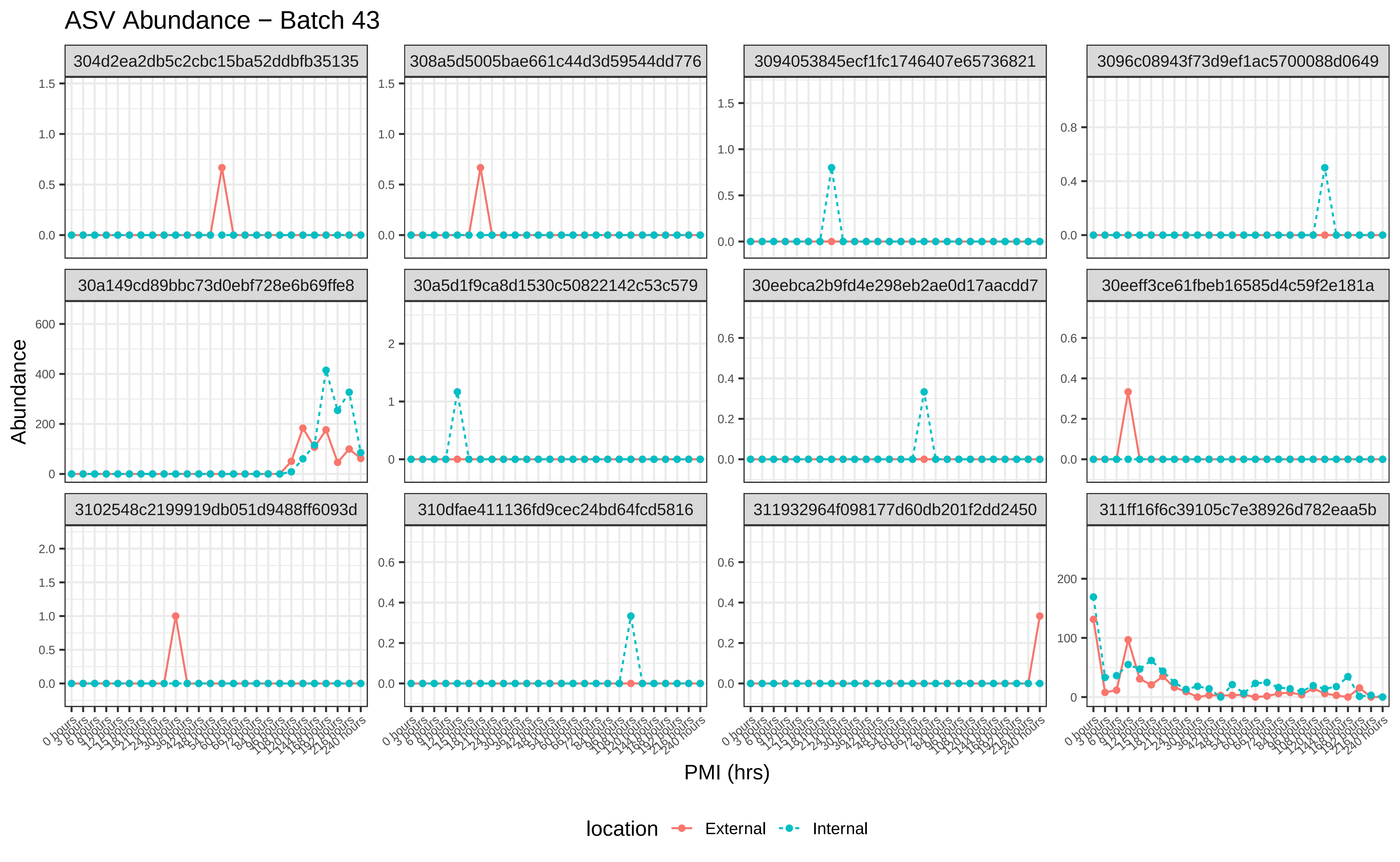

ASV Abundance − Batch 43
304d2ea2db5c2cbc15ba52ddbfb35135
308a5d5005bae661c44d3d59544dd776
3094053845ecf1fc1746407e65736821
3096c08943f73d9ef1ac5700088d0649
30a149cd89bbc73d0ebf728e6b69ffe8
30a5d1f9ca8d1530c50822142c53c579
30eebca2b9fd4e298eb2ae0d17aacdd7
30eeff3ce61fbeb16585d4c59f2e181a
3102548c2199919db051d9488ff6093d
310dfae411136fd9cec24bd64fcd5816
311932964f098177d60db201f2dd2450
311ff16f6c39105c7e38926d782eaa5b
Abundance
PMI (hrs)
location
External
Internal

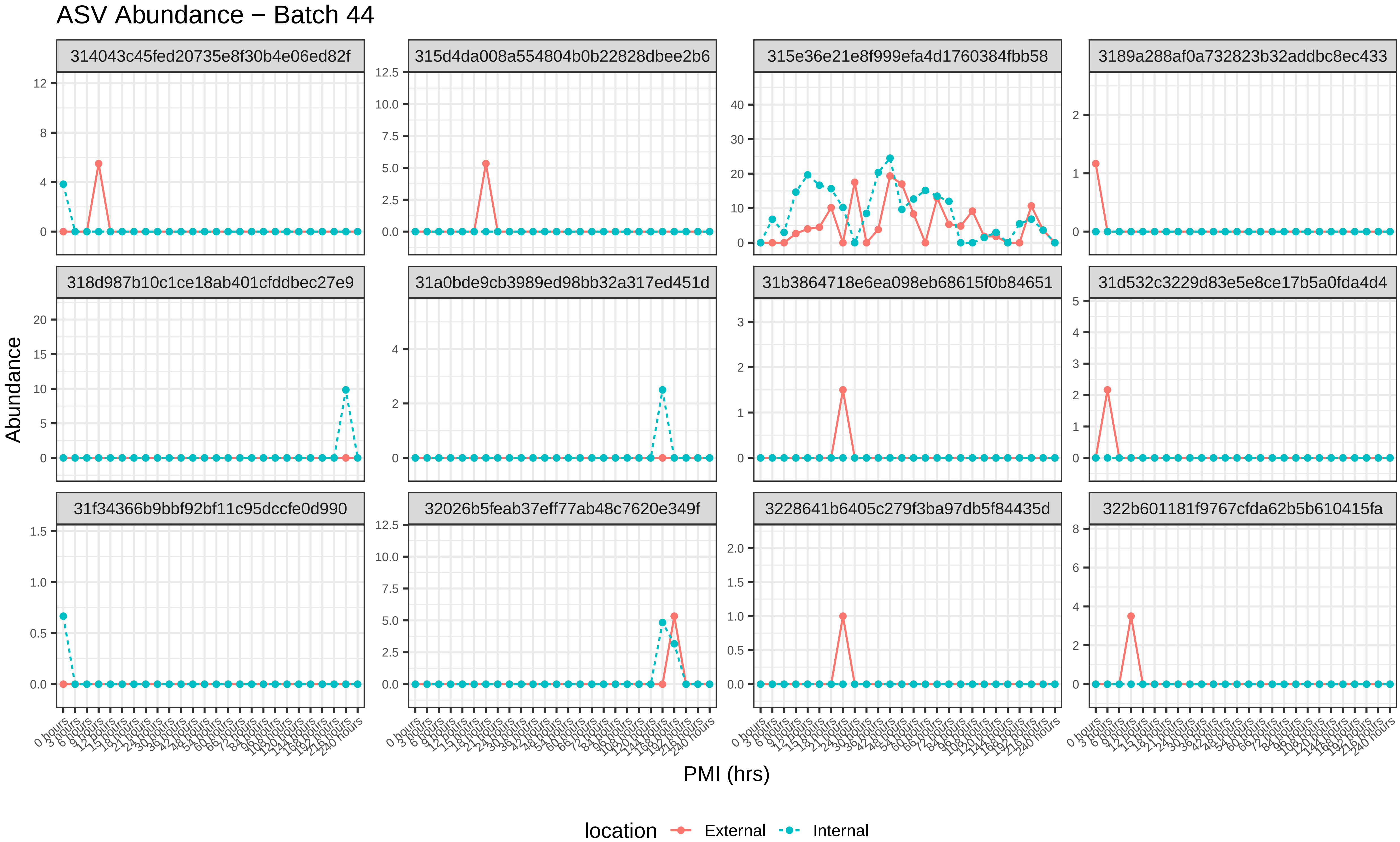

ASV Abundance − Batch 44
314043c45fed20735e8f30b4e06ed82f
315d4da008a554804b0b22828dbee2b6
315e36e21e8f999efa4d1760384fbb58
3189a288af0a732823b32addbc8ec433
318d987b10c1ce18ab401cfddbec27e9
31a0bde9cb3989ed98bb32a317ed451d
31b3864718e6ea098eb68615f0b84651
31d532c3229d83e5e8ce17b5a0fda4d4
31f34366b9bbf92bf11c95dccfe0d990
32026b5feab37eff77ab48c7620e349f
3228641b6405c279f3ba97db5f84435d
322b601181f9767cfda62b5b610415fa
Abundance
PMI (hrs)
location
External
Internal

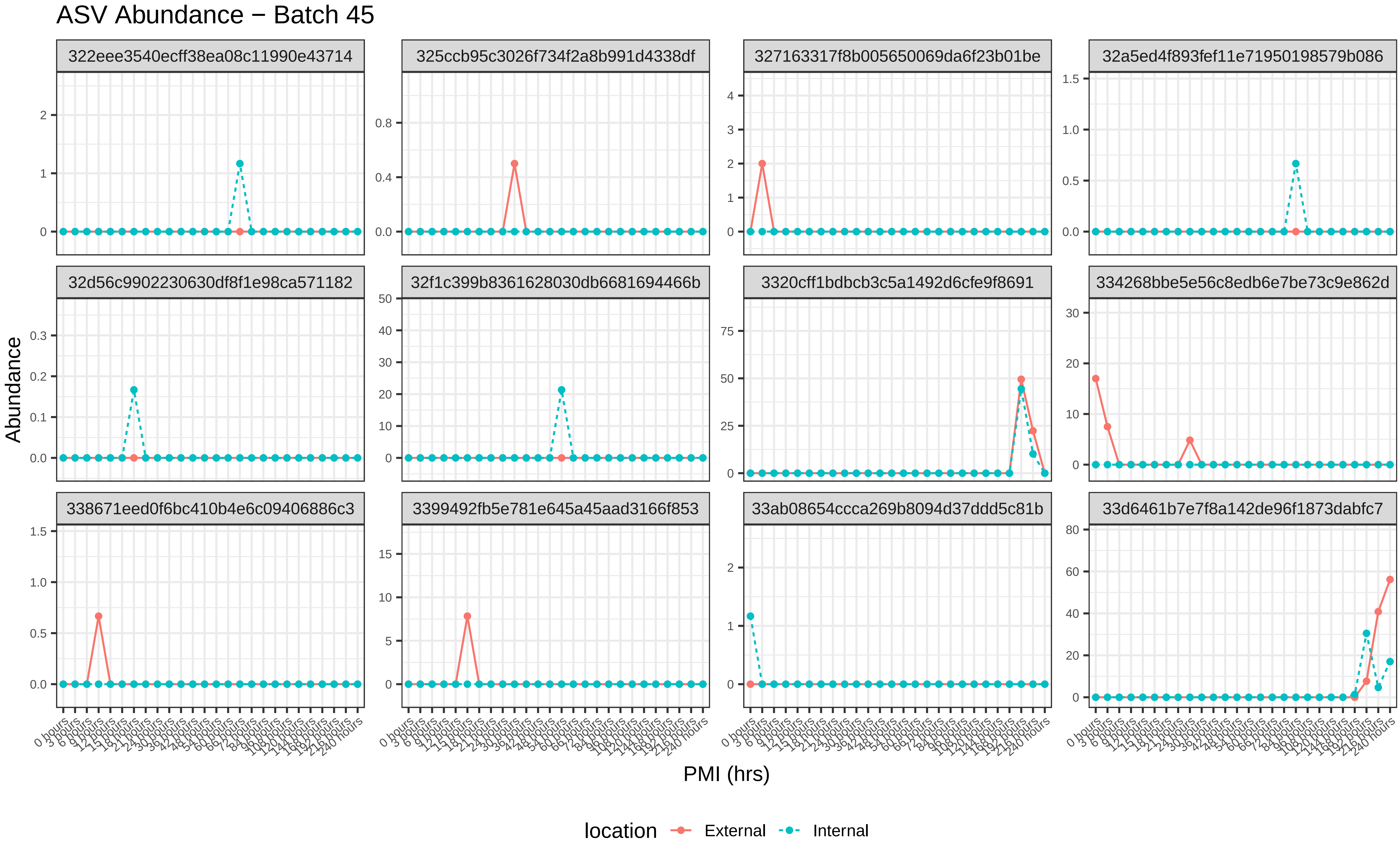

ASV Abundance − Batch 45
322eee3540ecff38ea08c11990e43714
325ccb95c3026f734f2a8b991d4338df
327163317f8b005650069da6f23b01be
32a5ed4f893fef11e71950198579b086
32d56c9902230630df8f1e98ca571182
32f1c399b8361628030db6681694466b
3320cff1bdbcb3c5a1492d6cfe9f8691
334268bbe5e56c8edb6e7be73c9e862d
338671eed0f6bc410b4e6c09406886c3
3399492fb5e781e645a45aad3166f853
33ab08654ccca269b8094d37ddd5c81b
33d6461b7e7f8a142de96f1873dabfc7
Abundance
PMI (hrs)
location
External
Internal

ASV Abundance − Batch 46

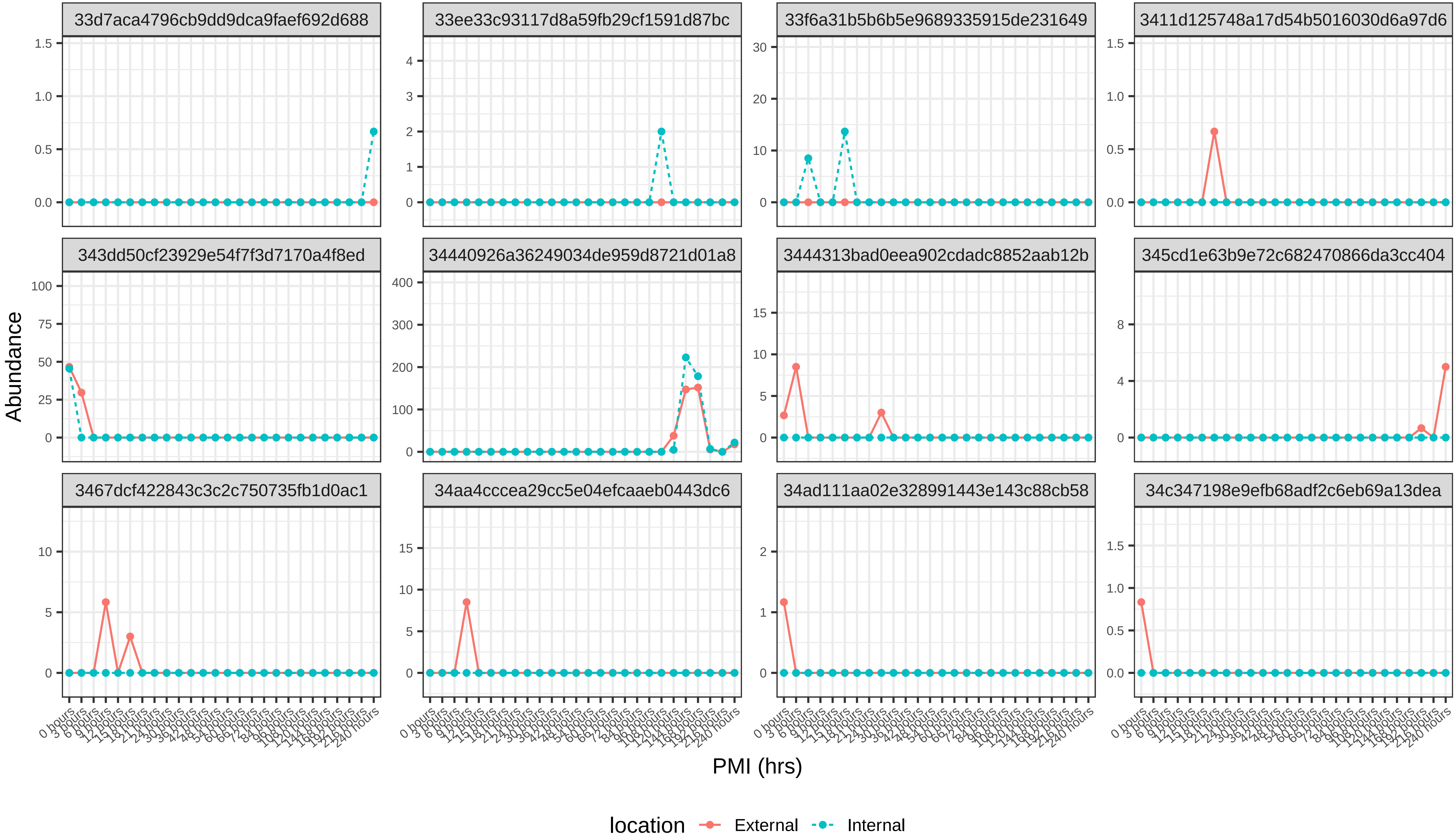

ASV Abundance − Batch 47

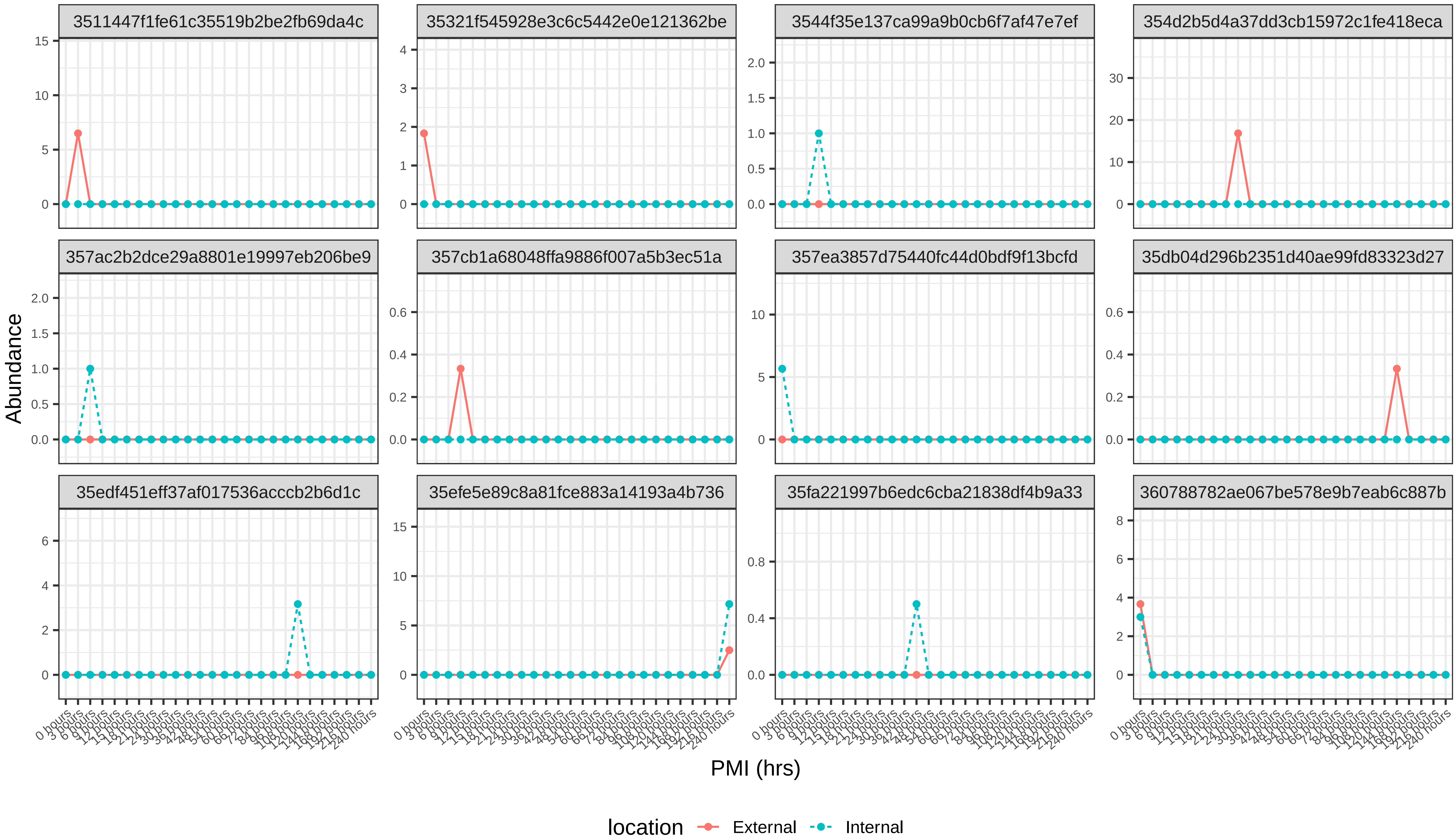

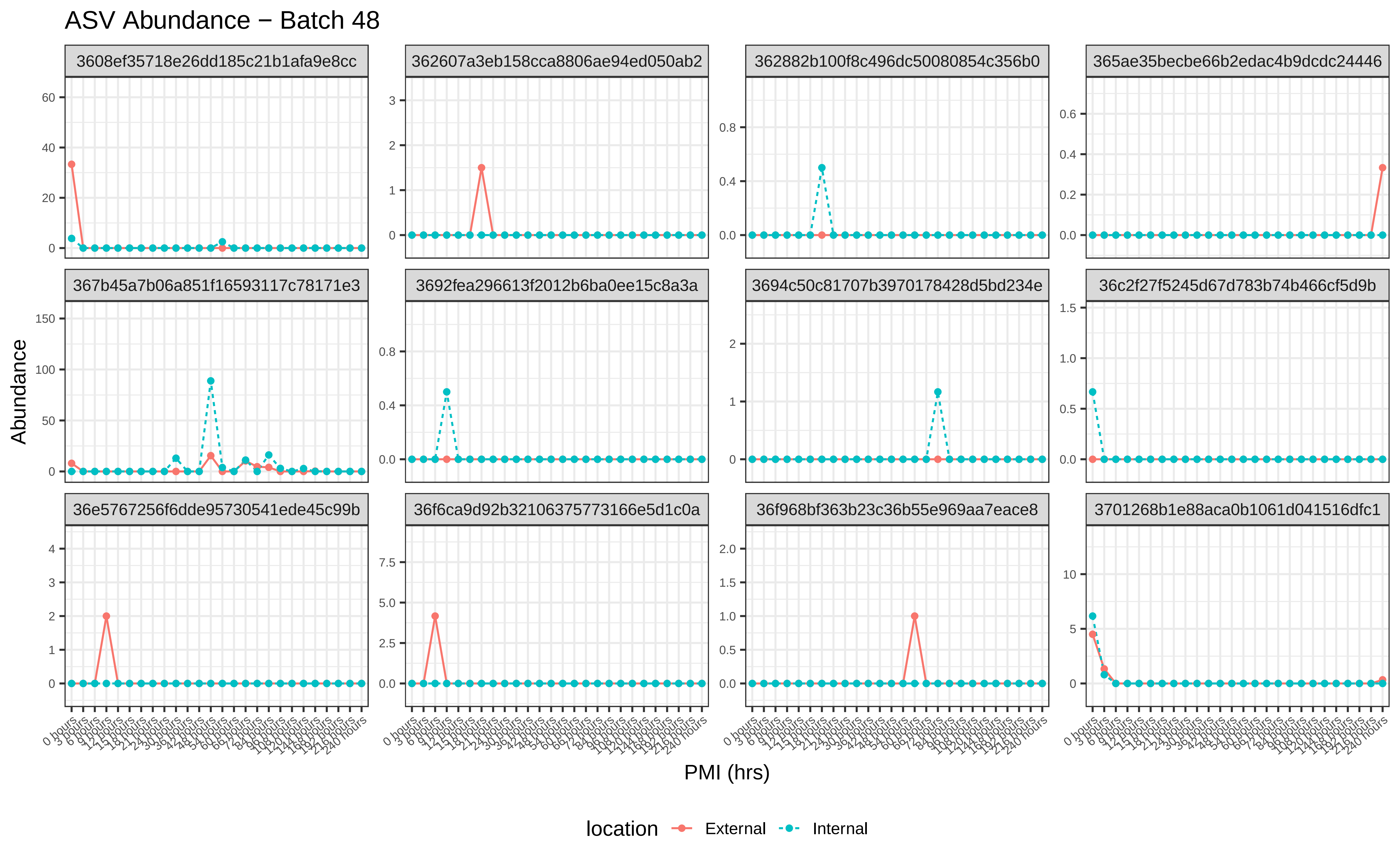

ASV Abundance − Batch 48
3608ef35718e26dd185c21b1afa9e8cc
362607a3eb158cca8806ae94ed050ab2
362882b100f8c496dc50080854c356b0
365ae35becbe66b2edac4b9dcdc24446
367b45a7b06a851f16593117c78171e3
3692fea296613f2012b6ba0ee15c8a3a
3694c50c81707b3970178428d5bd234e
36c2f27f5245d67d783b74b466cf5d9b
36e5767256f6dde95730541ede45c99b
36f6ca9d92b32106375773166e5d1c0a
36f968bf363b23c36b55e969aa7eace8
3701268b1e88aca0b1061d041516dfc1
Abundance
PMI (hrs)
location
External
Internal

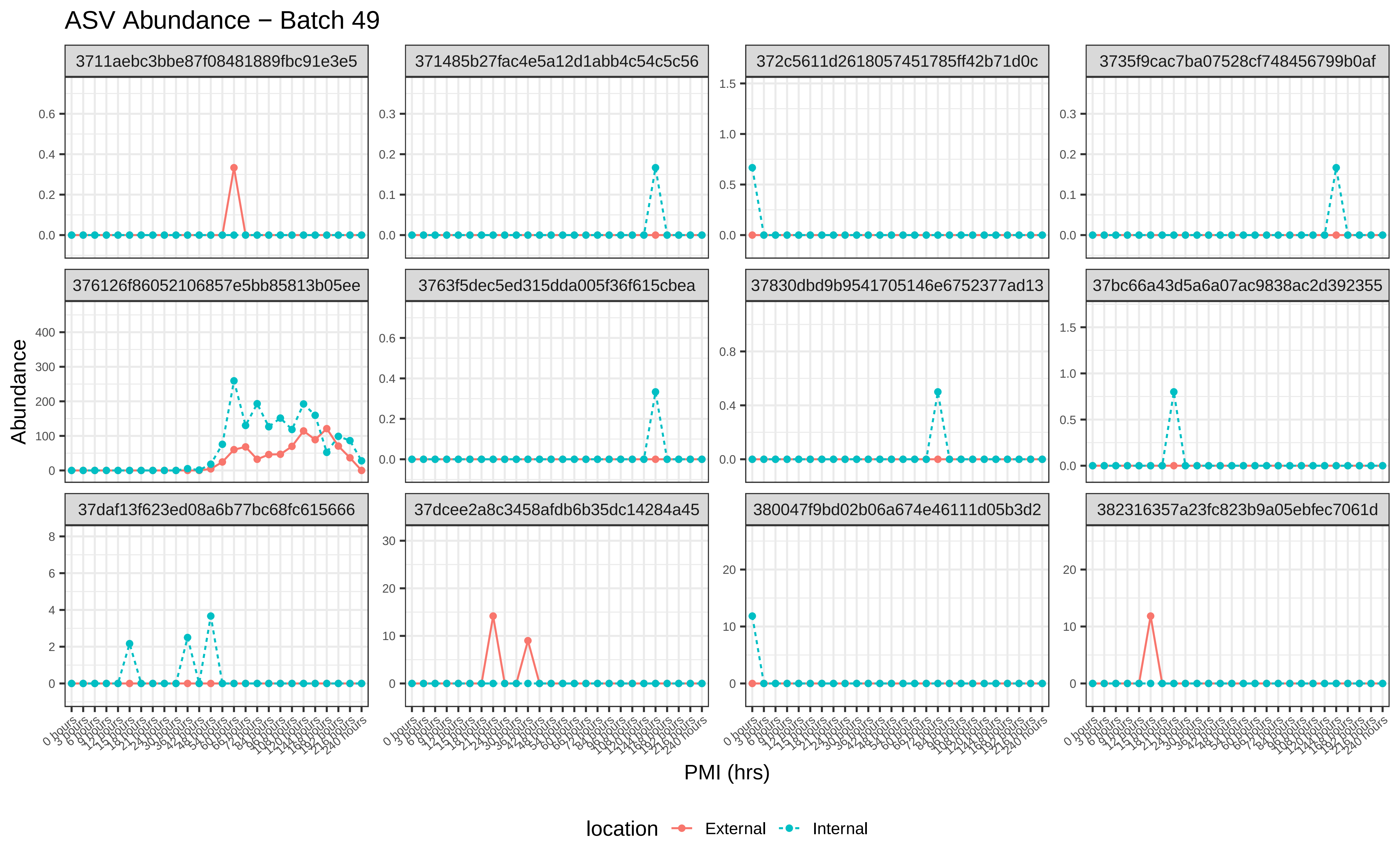

ASV Abundance − Batch 49
3711aebc3bbe87f08481889fbc91e3e5
371485b27fac4e5a12d1abb4c54c5c56
372c5611d2618057451785ff42b71d0c
3735f9cac7ba07528cf748456799b0af
376126f86052106857e5bb85813b05ee
3763f5dec5ed315dda005f36f615cbea
37830dbd9b9541705146e6752377ad13
37bc66a43d5a6a07ac9838ac2d392355
37daf13f623ed08a6b77bc68fc615666
37dcee2a8c3458afdb6b35dc14284a45
380047f9bd02b06a674e46111d05b3d2
382316357a23fc823b9a05ebfec7061d
Abundance
PMI (hrs)
location
External
Internal

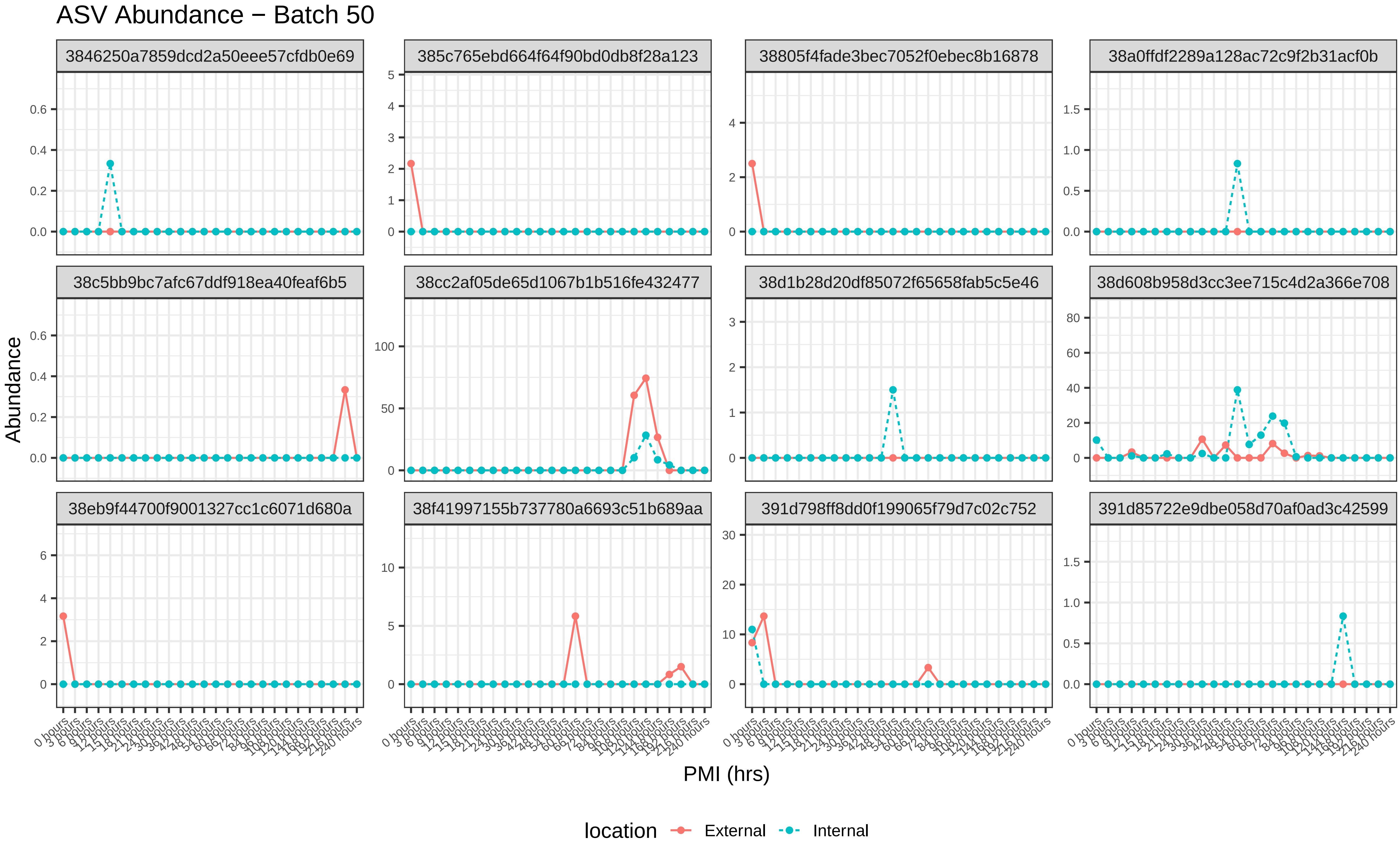
ASV Abundance − Batch 50
3846250a7859dcd2a50eee57cfdb0e69
385c765ebd664f64f90bd0db8f28a123
38805f4fade3bec7052f0ebec8b16878
38a0ffdf2289a128ac72c9f2b31acf0b
38c5bb9bc7afc67ddf918ea40feaf6b5
38cc2af05de65d1067b1b516fe432477
38d1b28d20df85072f65658fab5c5e46
38d608b958d3cc3ee715c4d2a366e708
38eb9f44700f9001327cc1c6071d680a
38f41997155b737780a6693c51b689aa
391d798ff8dd0f199065f79d7c02c752
391d85722e9dbe058d70af0ad3c42599
Abundance
PMI (hrs)
location
External
Internal

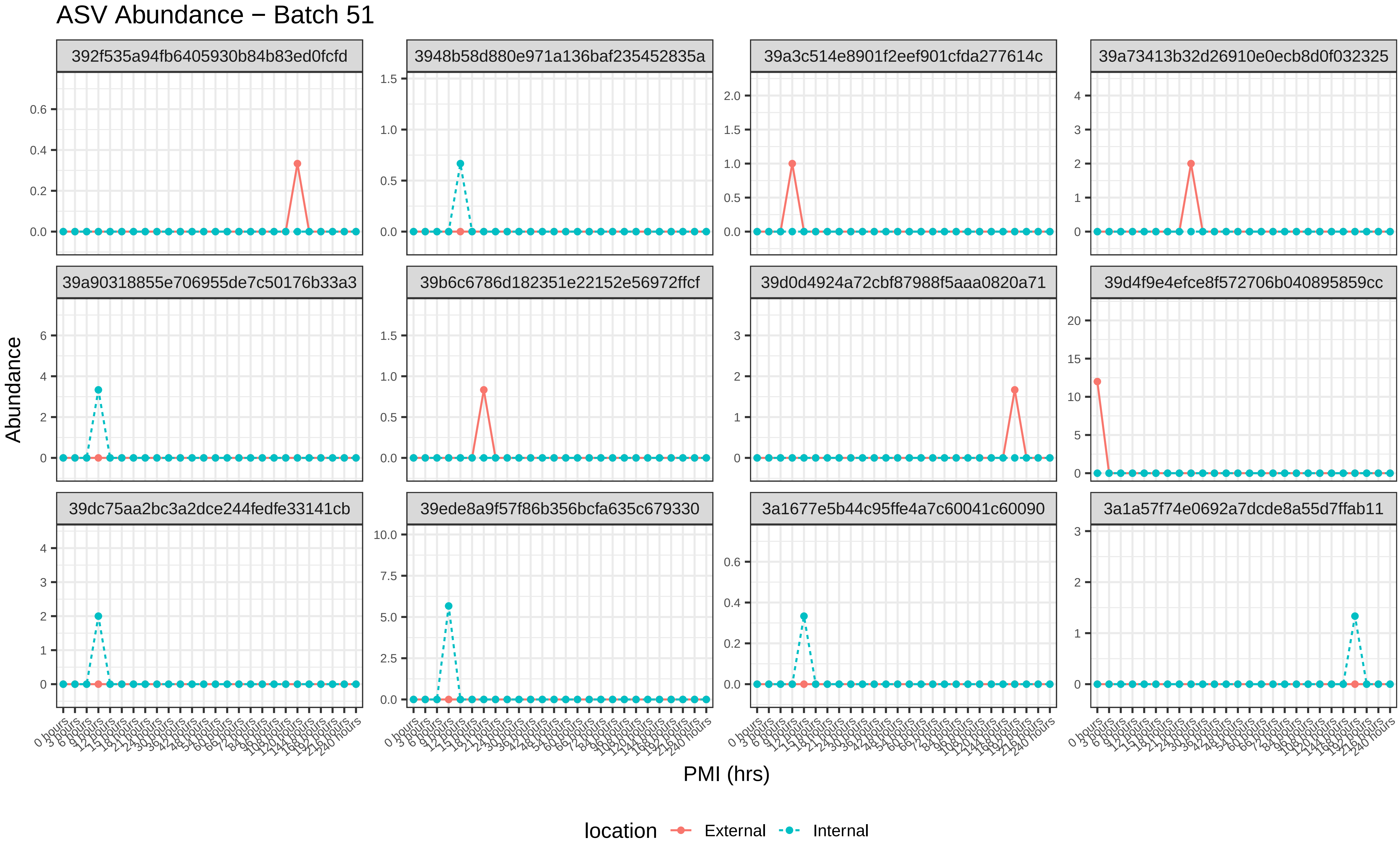
ASV Abundance − Batch 51
392f535a94fb6405930b84b83ed0fcfd
3948b58d880e971a136baf235452835a
39a3c514e8901f2eef901cfda277614c
39a73413b32d26910e0ecb8d0f032325
39a90318855e706955de7c50176b33a3
39b6c6786d182351e22152e56972ffcf
39d0d4924a72cbf87988f5aaa0820a71
39d4f9e4efce8f572706b040895859cc
39dc75aa2bc3a2dce244fedfe33141cb
39ede8a9f57f86b356bcfa635c679330
3a1677e5b44c95ffe4a7c60041c60090
3a1a57f74e0692a7dcde8a55d7ffab11
Abundance
PMI (hrs)
0 hours
3 hours
6 hours
9 hours
12 hours
15 hours
18 hours
21 hours
24 hours
30 hours
36 hours
42 hours
48 hours
54 hours
60 hours
66 hours
72 hours
84 hours
96 hours
108 hours
120 hours
144 hours
168 hours
192 hours
216 hours
240 hours
location
External
Internal

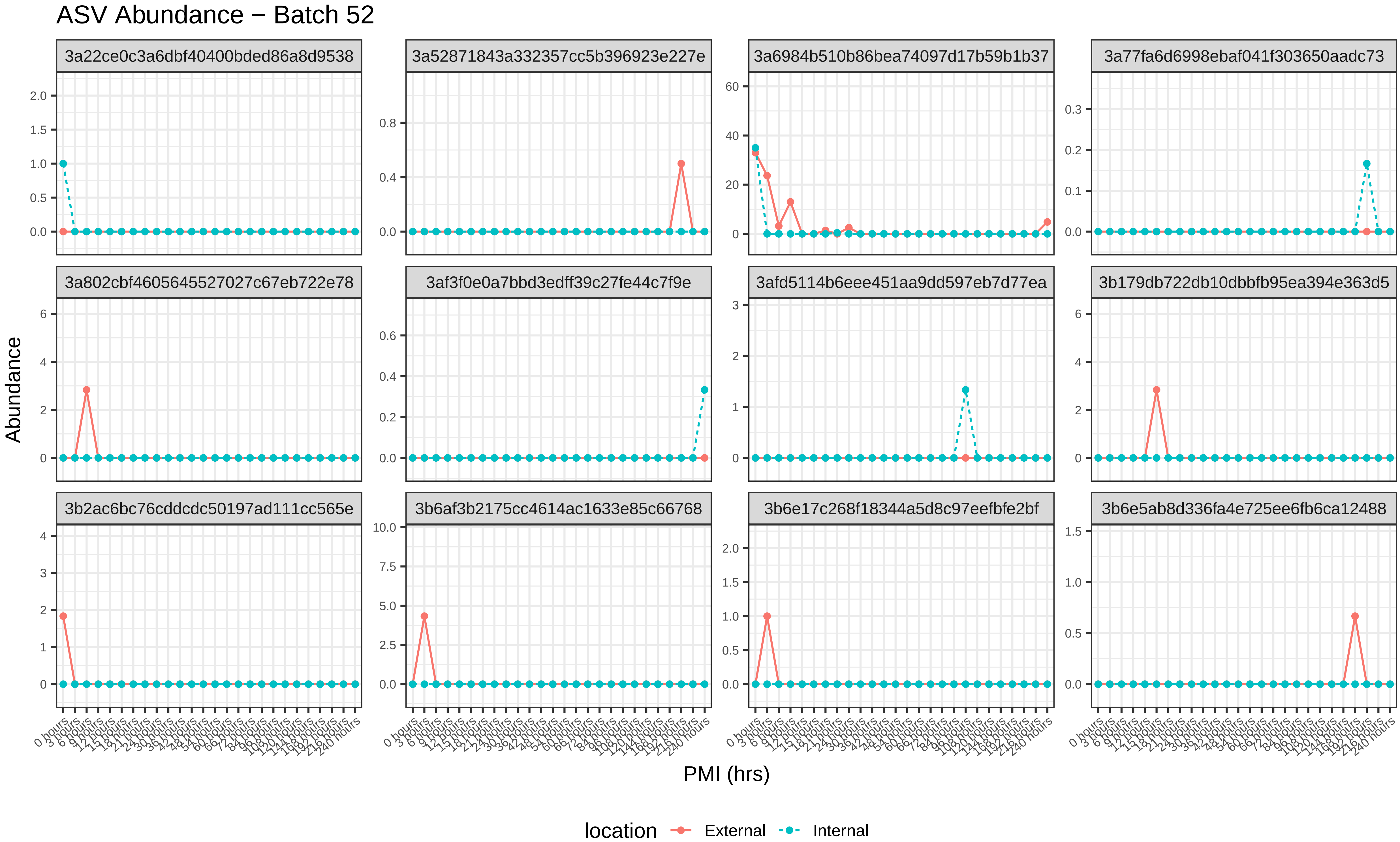

ASV Abundance − Batch 52
3a22ce0c3a6dbf40400bded86a8d9538
3a52871843a332357cc5b396923e227e
3a6984b510b86bea74097d17b59b1b37
3a77fa6d6998ebaf041f303650aadc73
3a802cbf4605645527027c67eb722e78
3af3f0e0a7bbd3edff39c27fe44c7f9e
3afd5114b6eee451aa9dd597eb7d77ea
3b179db722db10dbbfb95ea394e363d5
3b2ac6bc76cddcdc50197ad111cc565e
3b6af3b2175cc4614ac1633e85c66768
3b6e17c268f18344a5d8c97eefbfe2bf
3b6e5ab8d336fa4e725ee6fb6ca12488
Abundance
PMI (hrs)
location
External
Internal

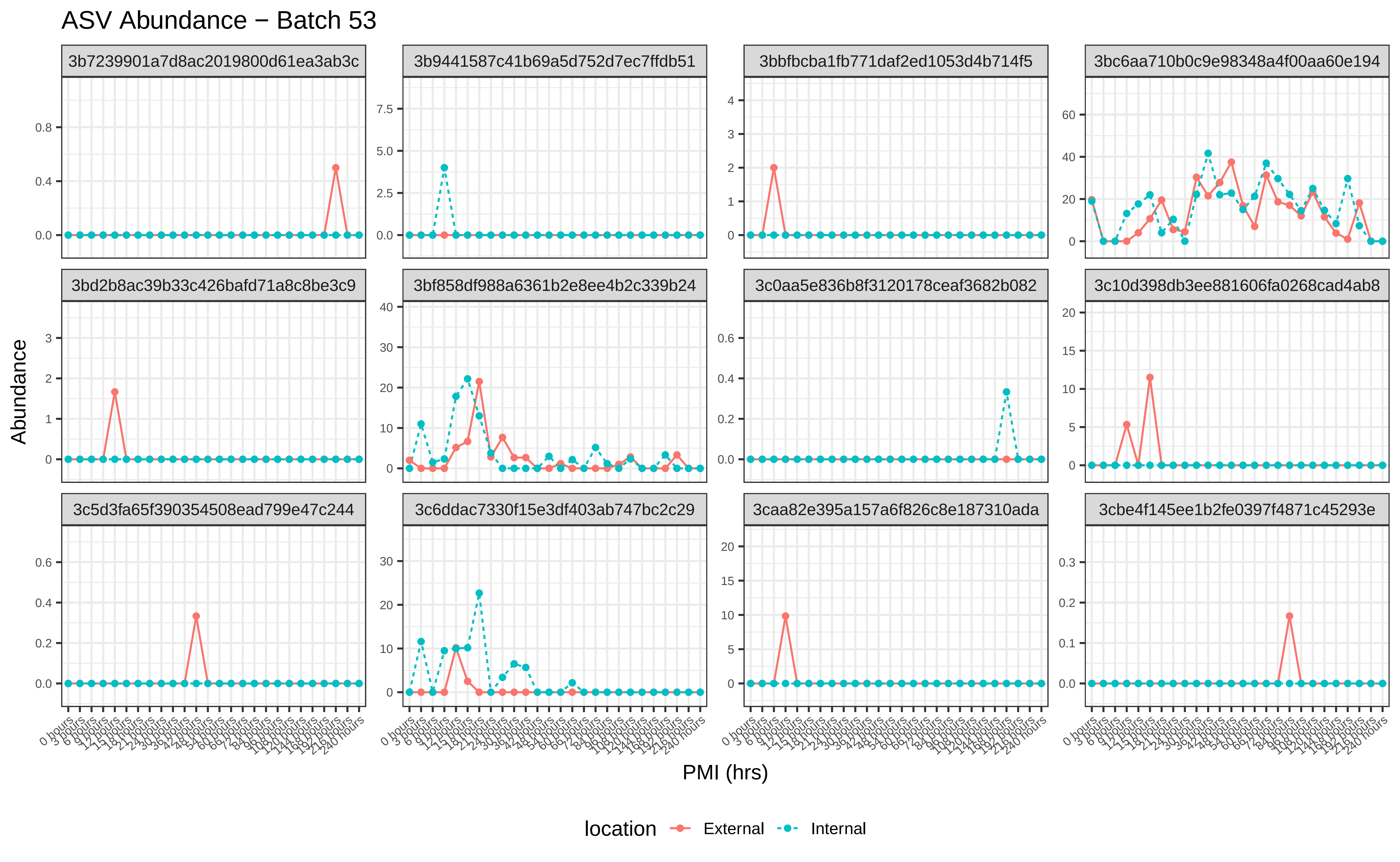
ASV Abundance − Batch 53
3b7239901a7d8ac2019800d61ea3ab3c
3b9441587c41b69a5d752d7ec7ffdb51
3bbfbcba1fb771daf2ed1053d4b714f5
3bc6aa710b0c9e98348a4f00aa60e194
3bd2b8ac39b33c426bafd71a8c8be3c9
3bf858df988a6361b2e8ee4b2c339b24
3c0aa5e836b8f3120178ceaf3682b082
3c10d398db3ee881606fa0268cad4ab8
3c5d3fa65f390354508ead799e47c244
3c6ddac7330f15e3df403ab747bc2c29
3caa82e395a157a6f826c8e187310ada
3cbe4f145ee1b2fe0397f4871c45293e
Abundance
PMI (hrs)
location
External
Internal

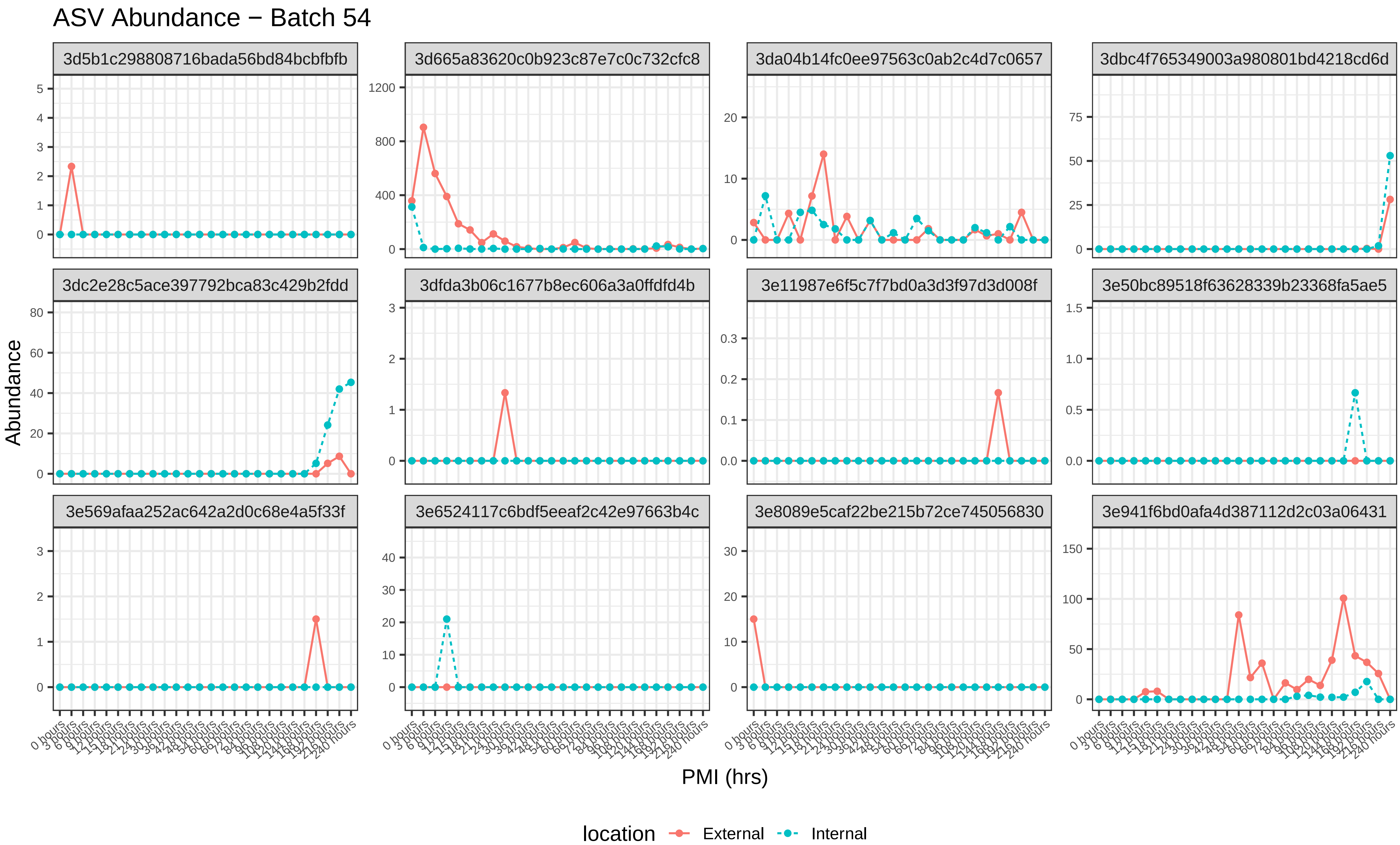
ASV Abundance − Batch 54
3d5b1c298808716bada56bd84bcbfbfb
3d665a83620c0b923c87e7c0c732cfc8
3da04b14fc0ee97563c0ab2c4d7c0657
3dbc4f765349003a980801bd4218cd6d
3dc2e28c5ace397792bca83c429b2fdd
3dfda3b06c1677b8ec606a3a0ffdfd4b
3e11987e6f5c7f7bd0a3d3f97d3d008f
3e50bc89518f63628339b23368fa5ae5
3e569afaa252ac642a2d0c68e4a5f33f
3e6524117c6bdf5eeaf2c42e97663b4c
3e8089e5caf22be215b72ce745056830
3e941f6bd0afa4d387112d2c03a06431
Abundance
PMI (hrs)
location
External
Internal

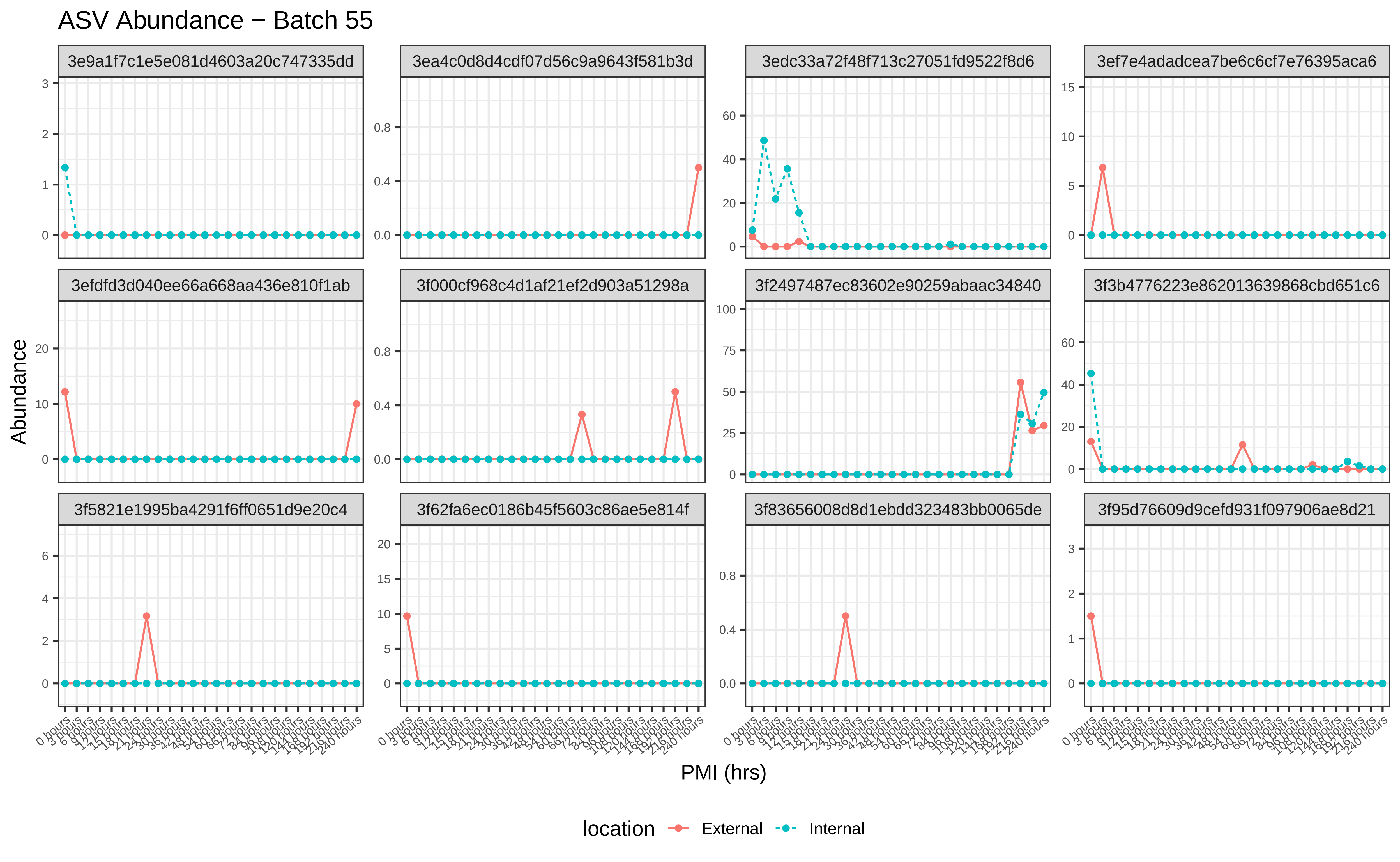
ASV Abundance − Batch 55
3e9a1f7c1e5e081d4603a20c747335dd
3ea4c0d8d4cdf07d56c9a9643f581b3d
3edc33a72f48f713c27051fd9522f8d6
3ef7e4adadcea7be6c6cf7e76395aca6
3efdfd3d040ee66a668aa436e810f1ab
3f000cf968c4d1af21ef2d903a51298a
3f2497487ec83602e90259abaac34840
3f3b4776223e862013639868cbd651c6
3f5821e1995ba4291f6ff0651d9e20c4
3f62fa6ec0186b45f5603c86ae5e814f
3f83656008d8d1ebdd323483bb0065de
3f95d76609d9cefd931f097906ae8d21
Abundance
PMI (hrs)
location
External
Internal

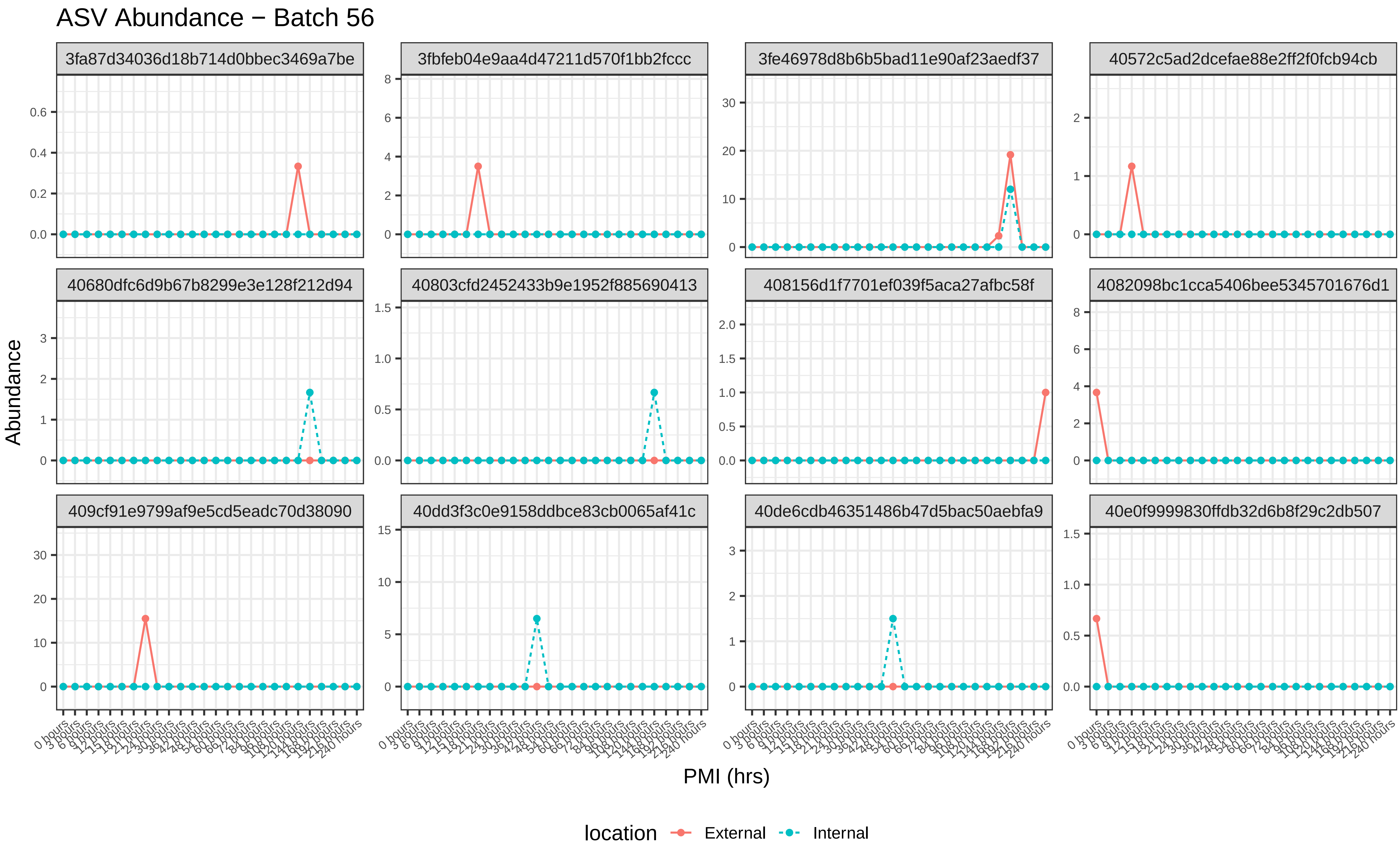
ASV Abundance − Batch 56
3fa87d34036d18b714d0bbec3469a7be
3fbfeb04e9aa4d47211d570f1bb2fccc
3fe46978d8b6b5bad11e90af23aedf37
40572c5ad2dcefae88e2ff2f0fcb94cb
40680dfc6d9b67b8299e3e128f212d94
40803cfd2452433b9e1952f885690413
408156d1f7701ef039f5aca27afbc58f
4082098bc1cca5406bee5345701676d1
409cf91e9799af9e5cd5eadc70d38090
40dd3f3c0e9158ddbce83cb0065af41c
40de6cdb46351486b47d5bac50aebfa9
40e0f9999830ffdb32d6b8f29c2db507
Abundance
PMI (hrs)
location
External
Internal

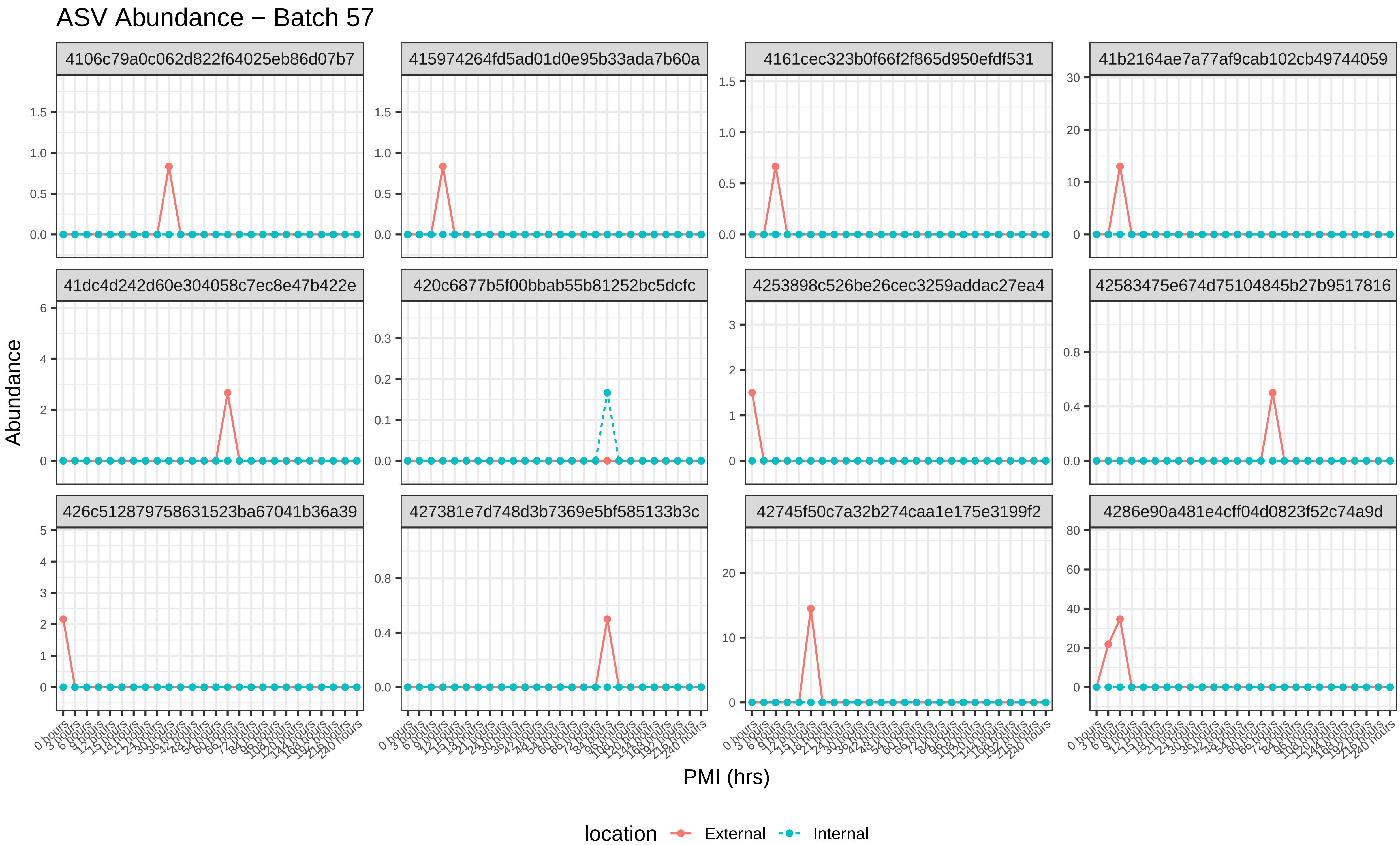
ASV Abundance − Batch 57
4106c79a0c062d822f64025eb86d07b7
415974264fd5ad01d0e95b33ada7b60a
4161cec323b0f66f2f865d950efdf531
41b2164ae7a77af9cab102cb49744059
41dc4d242d60e304058c7ec8e47b422e
420c6877b5f00bbab55b81252bc5dcfc
4253898c526be26cec3259addac27ea4
42583475e674d75104845b27b9517816
426c512879758631523ba67041b36a39
427381e7d748d3b7369e5bf585133b3c
42745f50c7a32b274caa1e175e3199f2
4286e90a481e4cff04d0823f52c74a9d
Abundance
PMI (hrs)
location
External
Internal

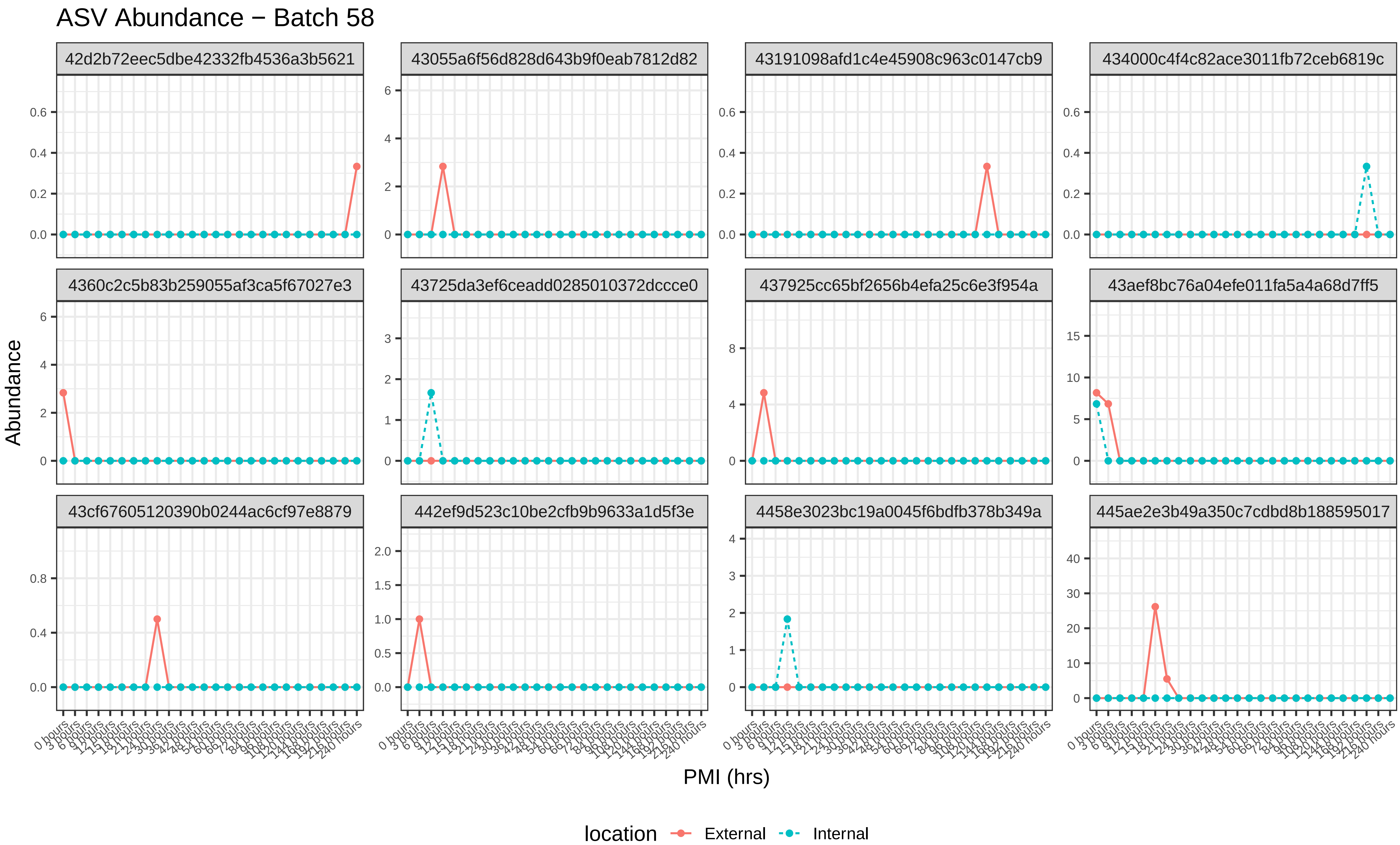
ASV Abundance − Batch 58
42d2b72eec5dbe42332fb4536a3b5621
43055a6f56d828d643b9f0eab7812d82
43191098afd1c4e45908c963c0147cb9
434000c4f4c82ace3011fb72ceb6819c
4360c2c5b83b259055af3ca5f67027e3
43725da3ef6ceadd0285010372dccce0
437925cc65bf2656b4efa25c6e3f954a
43aef8bc76a04efe011fa5a4a68d7ff5
43cf67605120390b0244ac6cf97e8879
442ef9d523c10be2cfb9b9633a1d5f3e
4458e3023bc19a0045f6bdfb378b349a
445ae2e3b49a350c7cdbd8b188595017
Abundance
PMI (hrs)
location
External
Internal

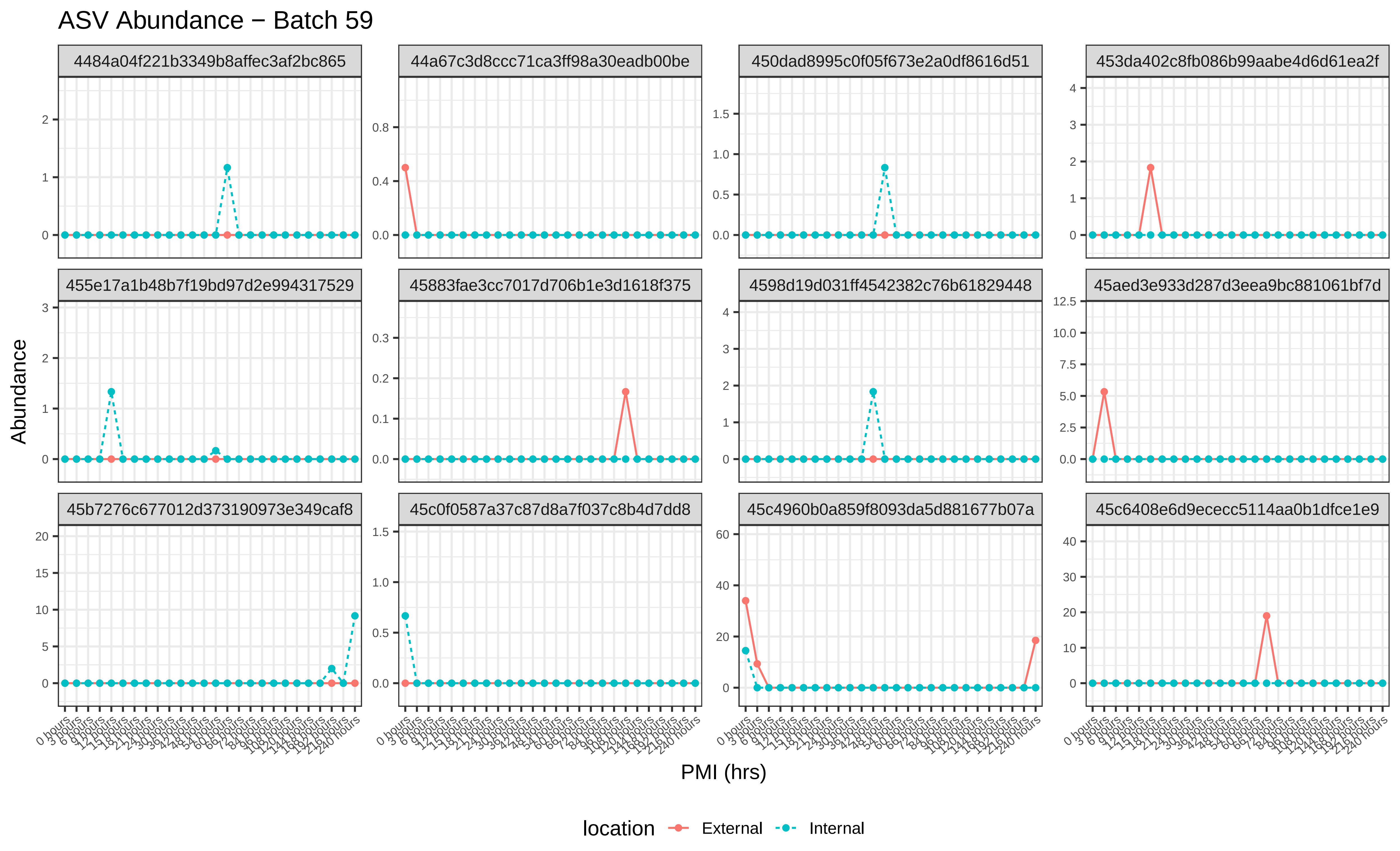
ASV Abundance − Batch 59
4484a04f221b3349b8affec3af2bc865
44a67c3d8ccc71ca3ff98a30eadb00be
450dad8995c0f05f673e2a0df8616d51
453da402c8fb086b99aabe4d6d61ea2f
455e17a1b48b7f19bd97d2e994317529
45883fae3cc7017d706b1e3d1618f375
4598d19d031ff4542382c76b61829448
45aed3e933d287d3eea9bc881061bf7d
45b7276c677012d373190973e349caf8
45c0f0587a37c87d8a7f037c8b4d7dd8
45c4960b0a859f8093da5d881677b07a
45c6408e6d9ececc5114aa0b1dfce1e9
Abundance
PMI (hrs)
location
External
Internal

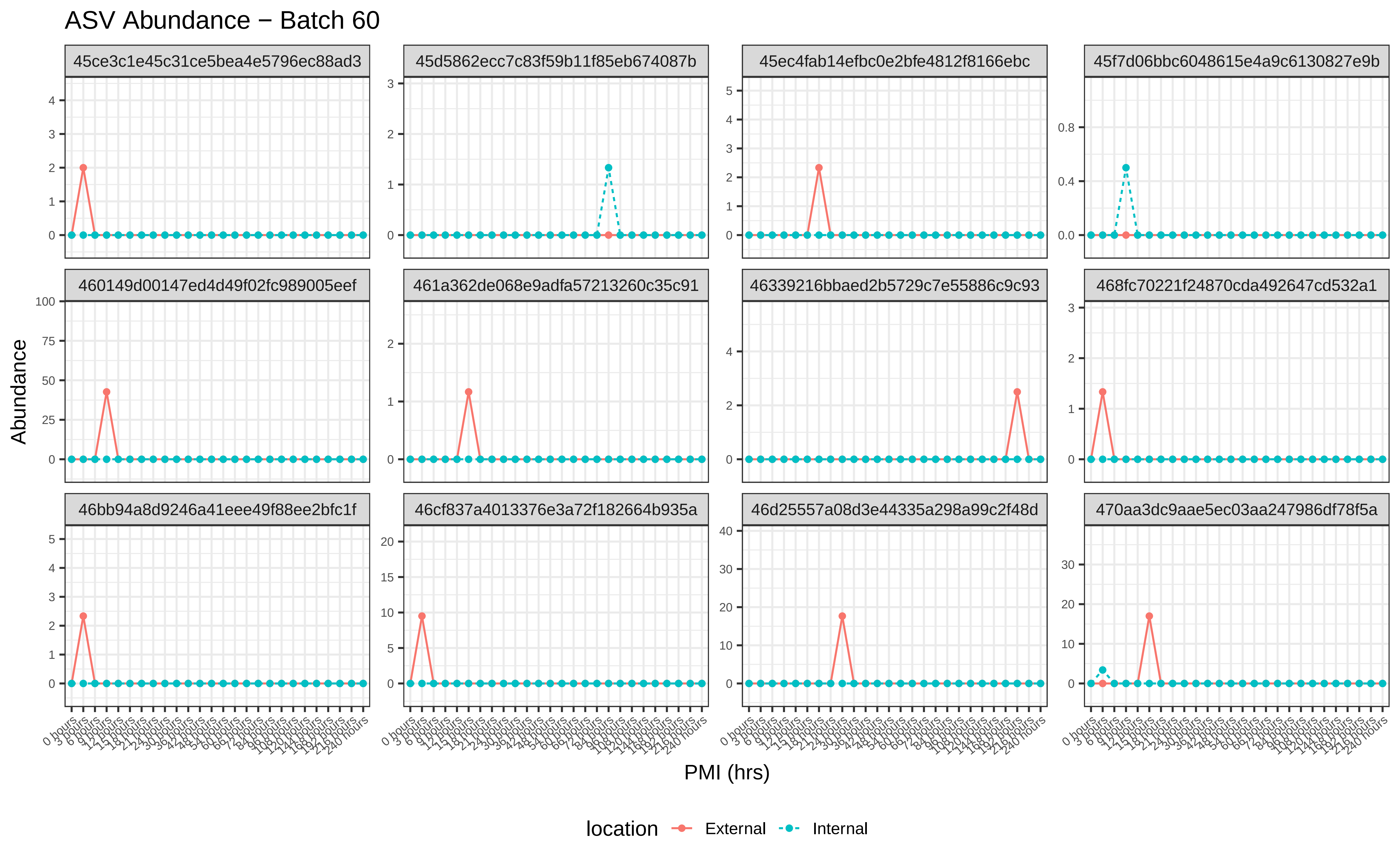

ASV Abundance − Batch 60
45ce3c1e45c31ce5bea4e5796ec88ad3
45d5862ecc7c83f59b11f85eb674087b
45ec4fab14efbc0e2bfe4812f8166ebc
45f7d06bbc6048615e4a9c6130827e9b
460149d00147ed4d49f02fc989005eef
461a362de068e9adfa57213260c35c91
46339216bbaed2b5729c7e55886c9c93
468fc70221f24870cda492647cd532a1
46bb94a8d9246a41eee49f88ee2bfc1f
46cf837a4013376e3a72f182664b935a
46d25557a08d3e44335a298a99c2f48d
470aa3dc9aae5ec03aa247986df78f5a
Abundance
PMI (hrs)
location
External
Internal

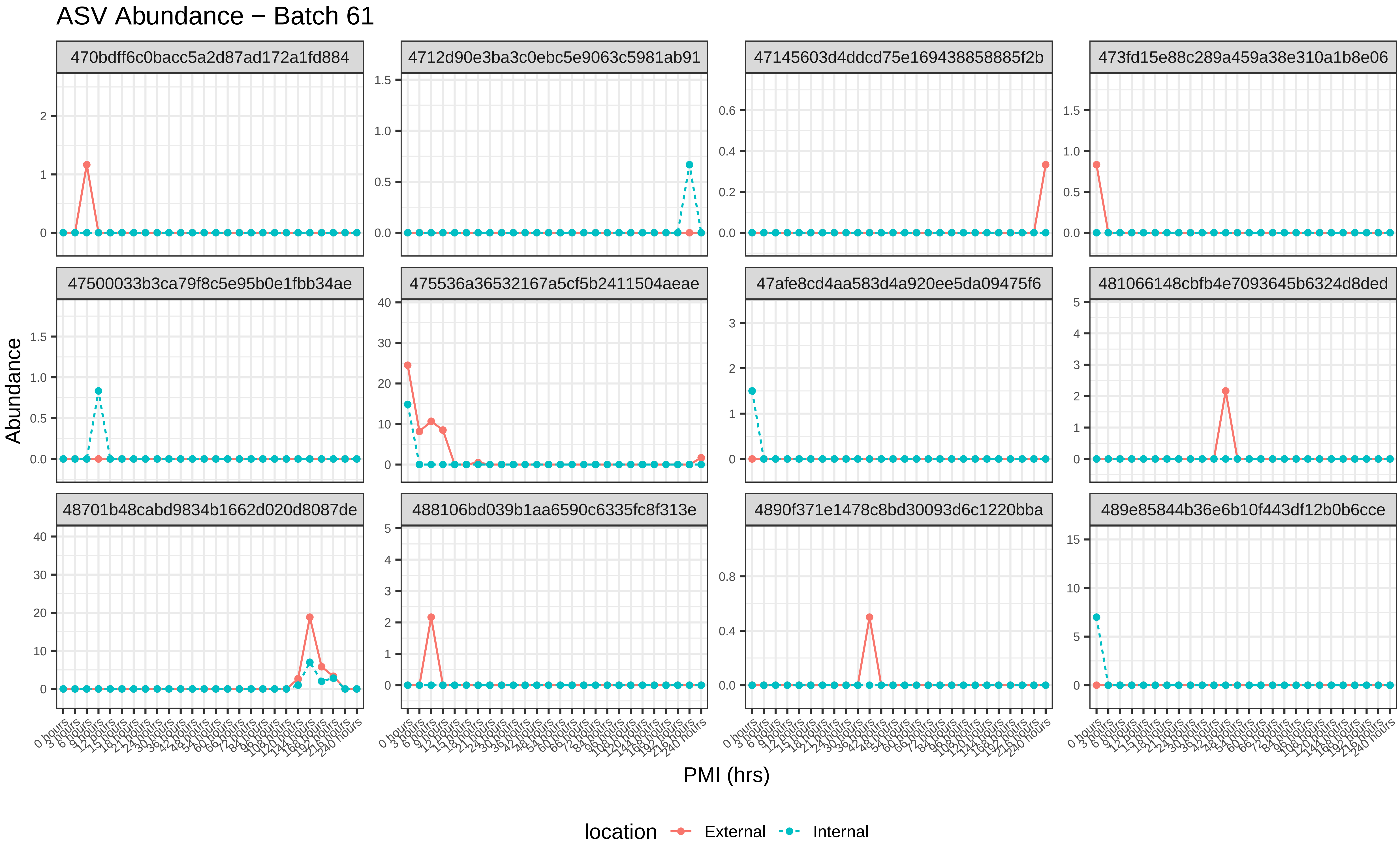
ASV Abundance − Batch 61
470bdff6c0bacc5a2d87ad172a1fd884
4712d90e3ba3c0ebc5e9063c5981ab91
47145603d4ddcd75e169438858885f2b
473fd15e88c289a459a38e310a1b8e06
47500033b3ca79f8c5e95b0e1fbb34ae
475536a36532167a5cf5b2411504aeae
47afe8cd4aa583d4a920ee5da09475f6
481066148cbfb4e7093645b6324d8ded
48701b48cabd9834b1662d020d8087de
488106bd039b1aa6590c6335fc8f313e
4890f371e1478c8bd30093d6c1220bba
489e85844b36e6b10f443df12b0b6cce
Abundance
PMI (hrs)
location
External
Internal

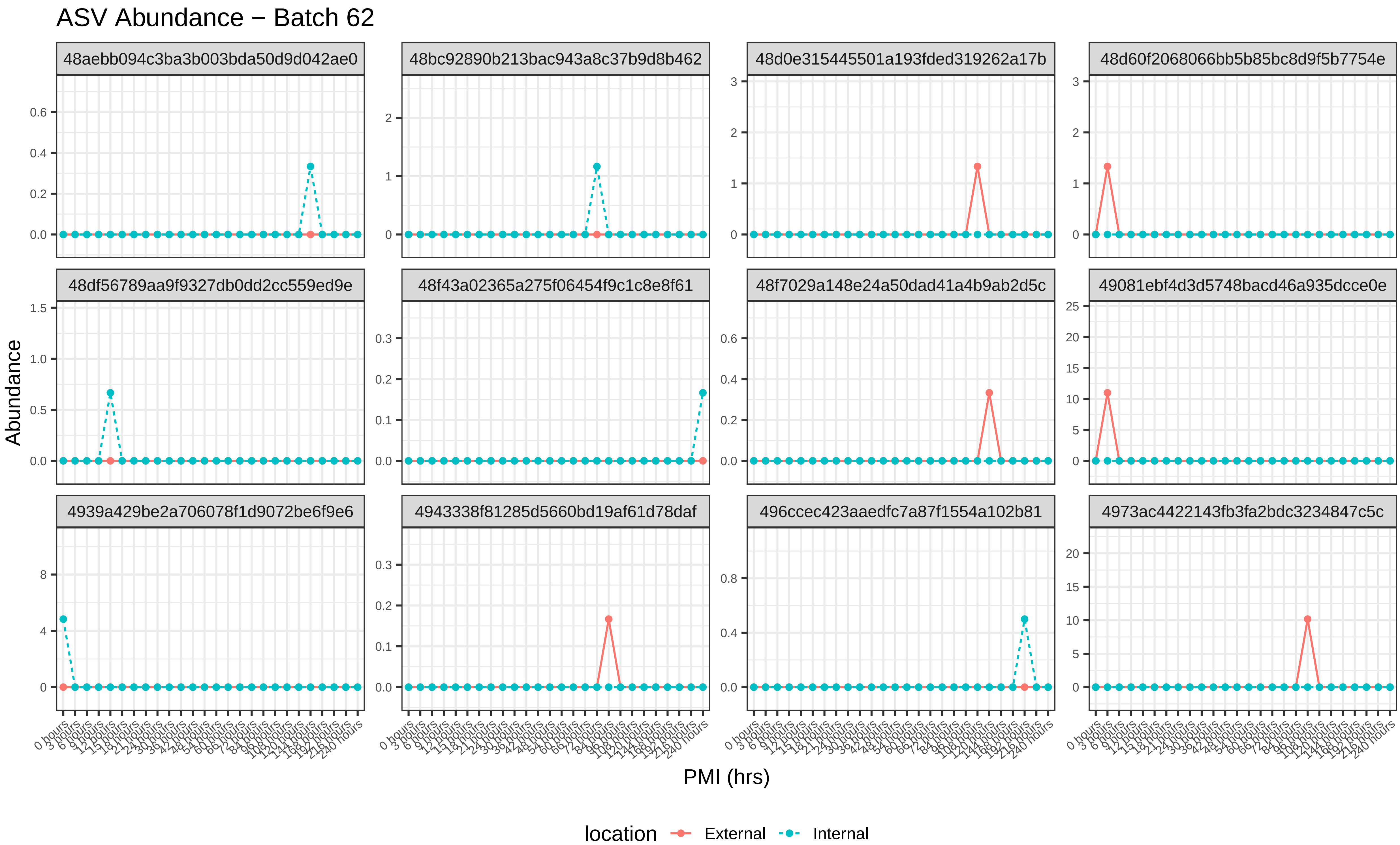

ASV Abundance − Batch 62
48aebb094c3ba3b003bda50d9d042ae0
48bc92890b213bac943a8c37b9d8b462
48d0e315445501a193fded319262a17b
48d60f2068066bb5b85bc8d9f5b7754e
48df56789aa9f9327db0dd2cc559ed9e
48f43a02365a275f06454f9c1c8e8f61
48f7029a148e24a50dad41a4b9ab2d5c
49081ebf4d3d5748bacd46a935dcce0e
4939a429be2a706078f1d9072be6f9e6
4943338f81285d5660bd19af61d78daf
496ccec423aaedfc7a87f1554a102b81
4973ac4422143fb3fa2bdc3234847c5c
Abundance
PMI (hrs)
location
External
Internal

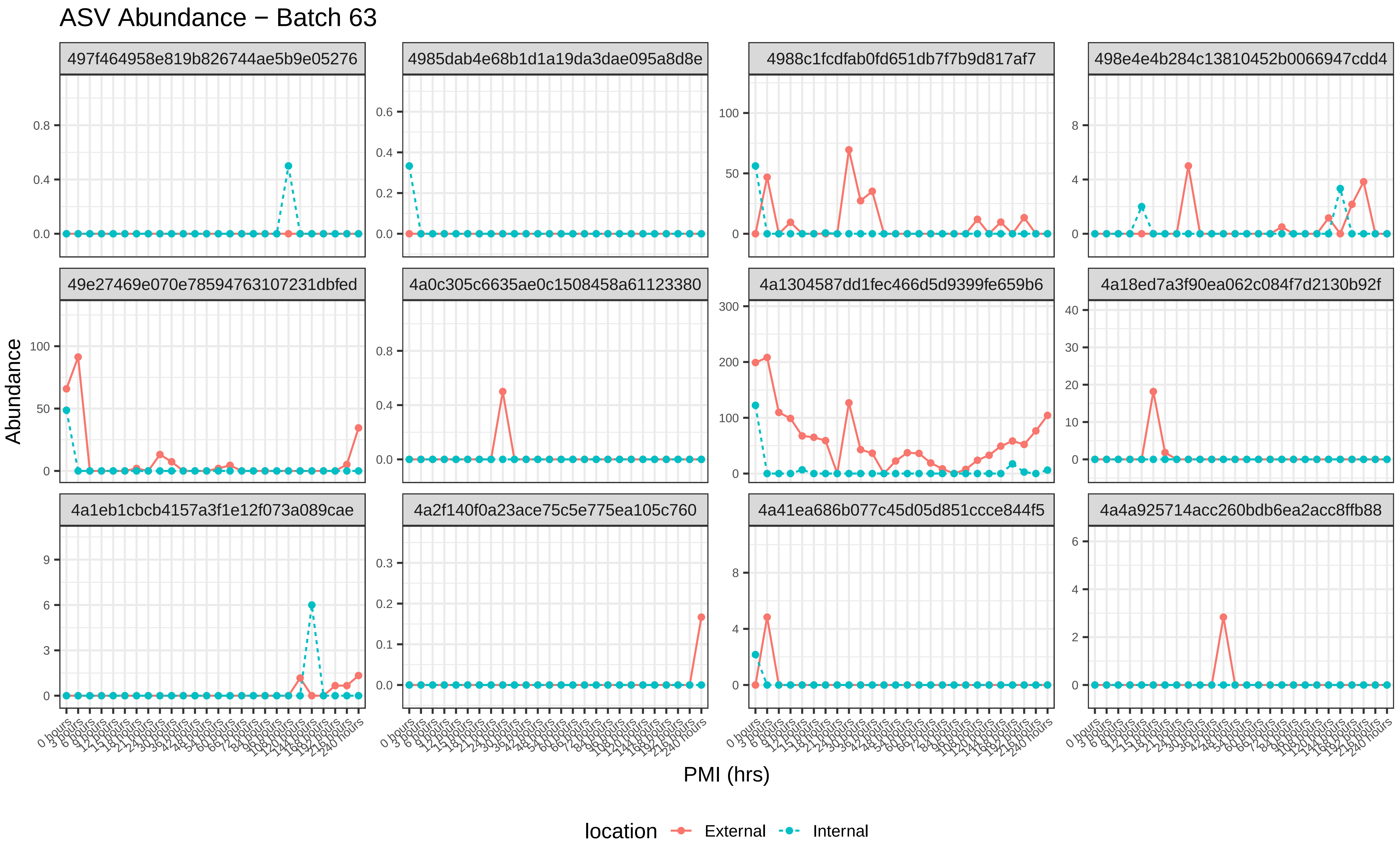
ASV Abundance − Batch 63
497f464958e819b826744ae5b9e05276
4985dab4e68b1d1a19da3dae095a8d8e
4988c1fcdfab0fd651db7f7b9d817af7
498e4e4b284c13810452b0066947cdd4
49e27469e070e78594763107231dbfed
4a0c305c6635ae0c1508458a61123380
4a1304587dd1fec466d5d9399fe659b6
4a18ed7a3f90ea062c084f7d2130b92f
4a1eb1cbcb4157a3f1e12f073a089cae
4a2f140f0a23ace75c5e775ea105c760
4a41ea686b077c45d05d851ccce844f5
4a4a925714acc260bdb6ea2acc8ffb88
Abundance
PMI (hrs)
location
External
Internal

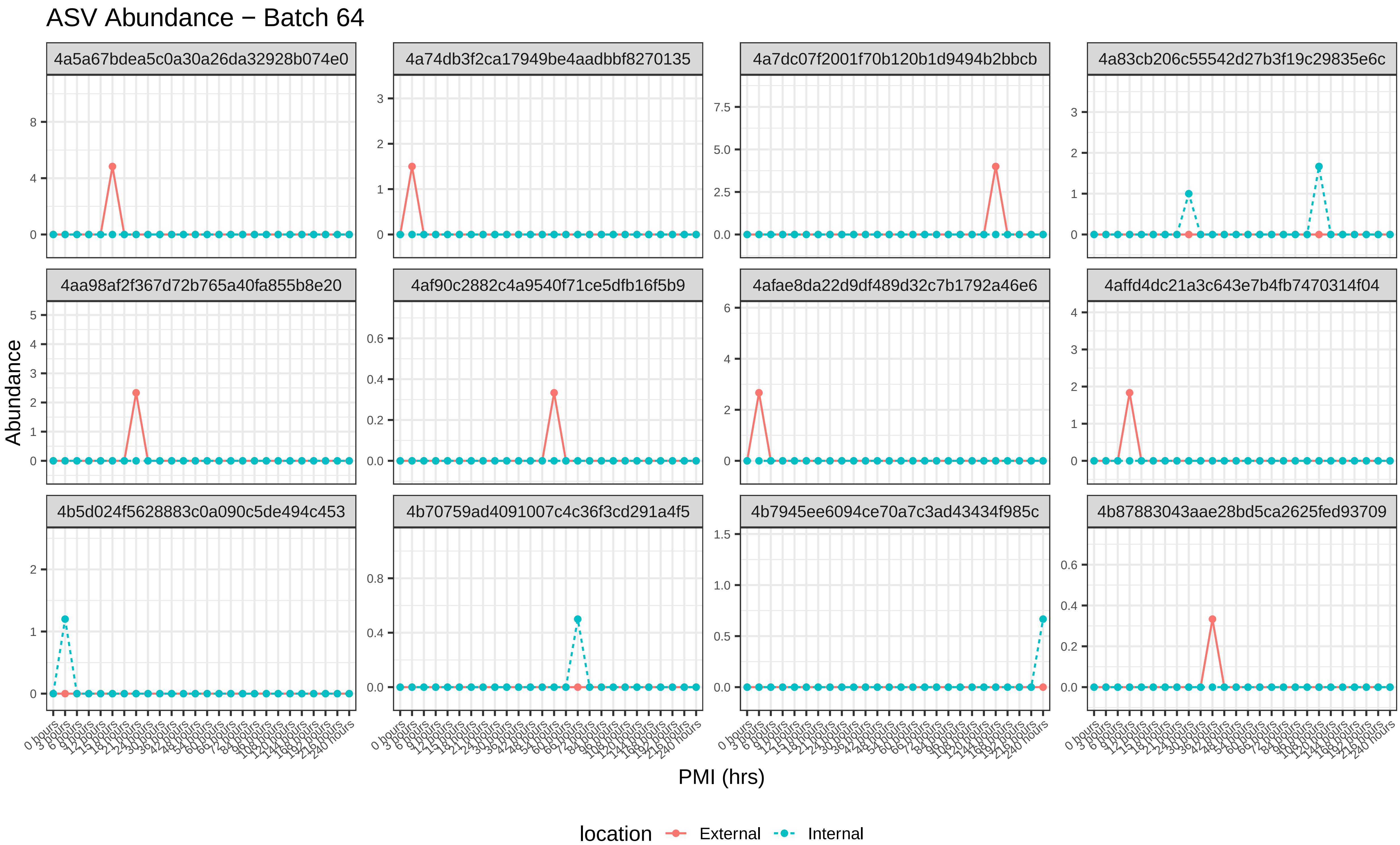

ASV Abundance − Batch 64
4a5a67bdea5c0a30a26da32928b074e0
4a74db3f2ca17949be4aadbbf8270135
4a7dc07f2001f70b120b1d9494b2bbcb
4a83cb206c55542d27b3f19c29835e6c
4aa98af2f367d72b765a40fa855b8e20
4af90c2882c4a9540f71ce5dfb16f5b9
4afae8da22d9df489d32c7b1792a46e6
4affd4dc21a3c643e7b4fb7470314f04
4b5d024f5628883c0a090c5de494c453
4b70759ad4091007c4c36f3cd291a4f5
4b7945ee6094ce70a7c3ad43434f985c
4b87883043aae28bd5ca2625fed93709
Abundance
PMI (hrs)
location
External
Internal

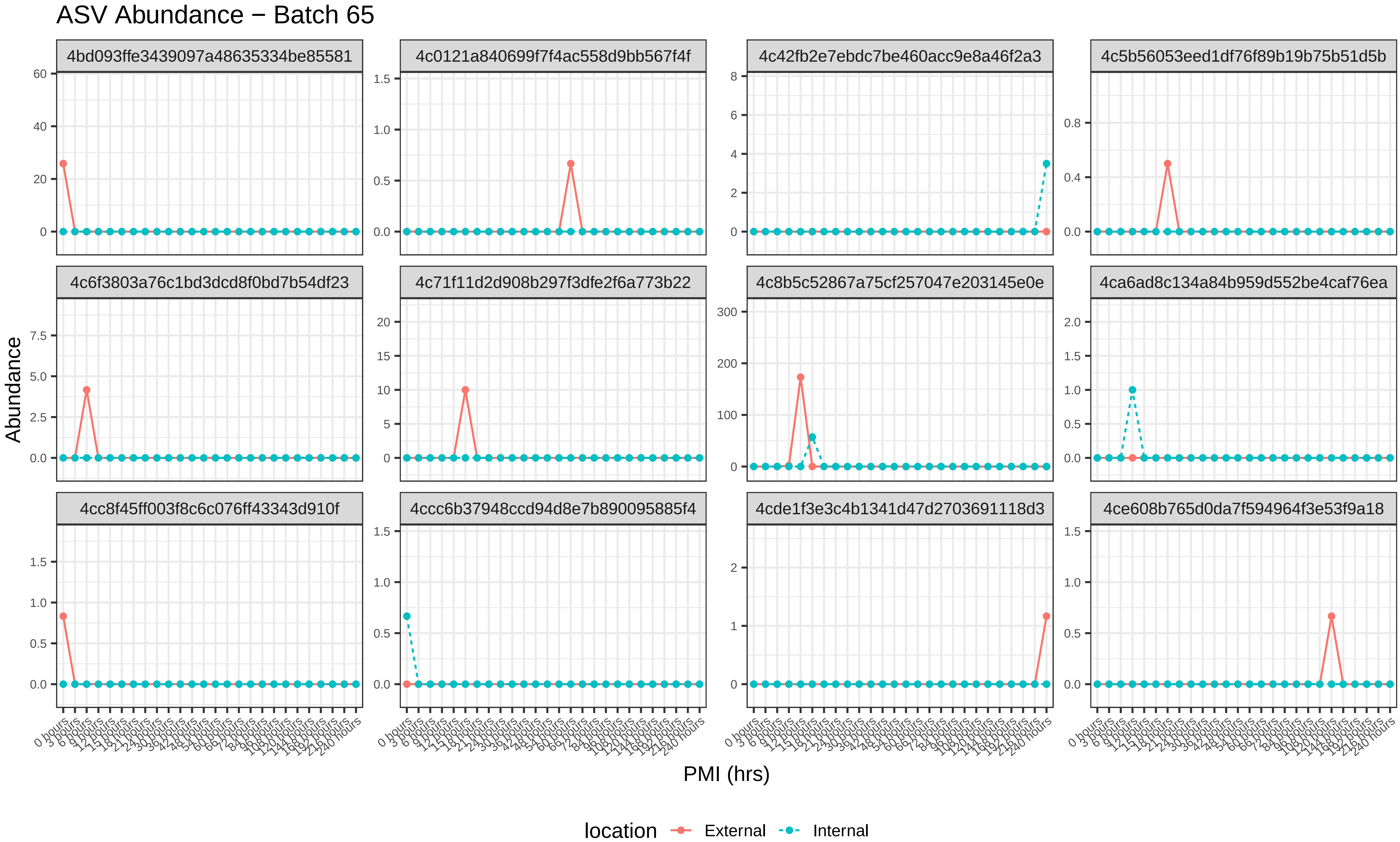
ASV Abundance − Batch 65
4bd093ffe3439097a48635334be85581
4c0121a840699f7f4ac558d9bb567f4f
4c42fb2e7ebdc7be460acc9e8a46f2a3
4c5b56053eed1df76f89b19b75b51d5b
4c6f3803a76c1bd3dcd8f0bd7b54df23
4c71f11d2d908b297f3dfe2f6a773b22
4c8b5c52867a75cf257047e203145e0e
4ca6ad8c134a84b959d552be4caf76ea
4cc8f45ff003f8c6c076ff43343d910f
4ccc6b37948ccd94d8e7b890095885f4
4cde1f3e3c4b1341d47d2703691118d3
4ce608b765d0da7f594964f3e53f9a18
Abundance
PMI (hrs)
location
External
Internal

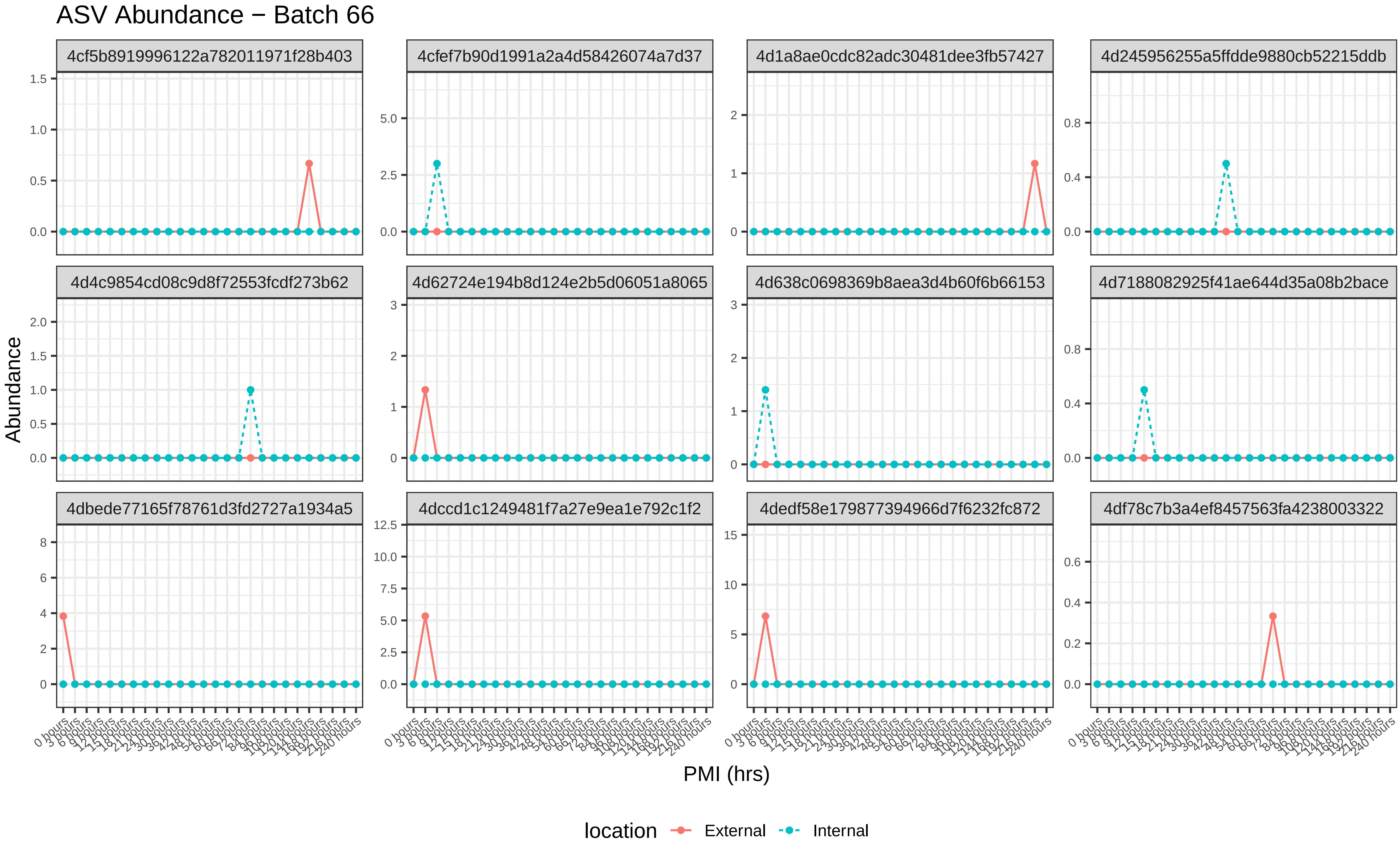
ASV Abundance − Batch 66
4cf5b8919996122a782011971f28b403
4cfef7b90d1991a2a4d58426074a7d37
4d1a8ae0cdc82adc30481dee3fb57427
4d245956255a5ffdde9880cb52215ddb
4d4c9854cd08c9d8f72553fcdf273b62
4d62724e194b8d124e2b5d06051a8065
4d638c0698369b8aea3d4b60f6b66153
4d7188082925f41ae644d35a08b2bace
4dbede77165f78761d3fd2727a1934a5
4dccd1c1249481f7a27e9ea1e792c1f2
4dedf58e179877394966d7f6232fc872
4df78c7b3a4ef8457563fa4238003322
Abundance
PMI (hrs)
location
External
Internal

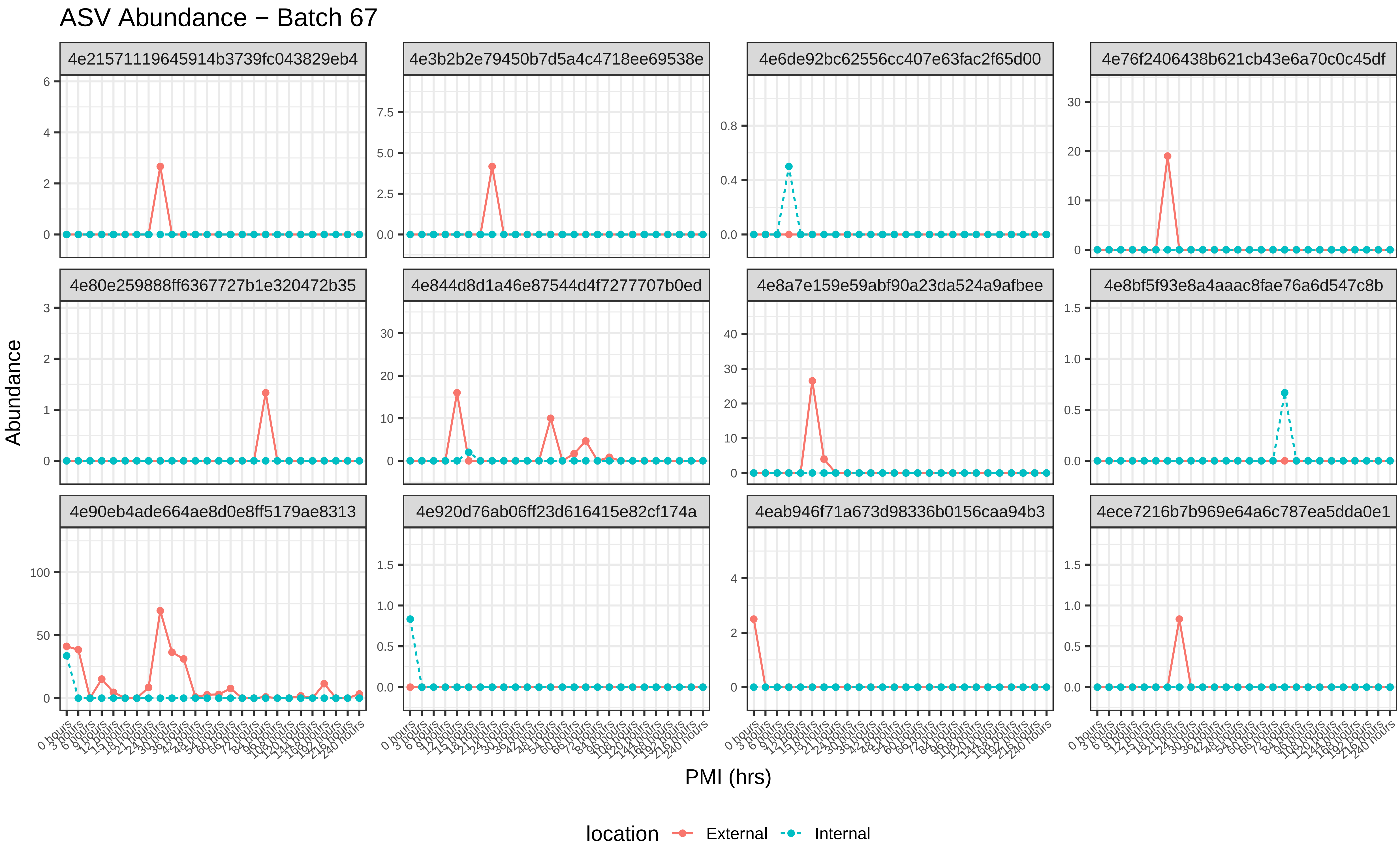
ASV Abundance − Batch 67
4e21571119645914b3739fc043829eb4
4e3b2b2e79450b7d5a4c4718ee69538e
4e6de92bc62556cc407e63fac2f65d00
4e76f2406438b621cb43e6a70c0c45df
4e80e259888ff6367727b1e320472b35
4e844d8d1a46e87544d4f7277707b0ed
4e8a7e159e59abf90a23da524a9afbee
4e8bf5f93e8a4aaac8fae76a6d547c8b
4e90eb4ade664ae8d0e8ff5179ae8313
4e920d76ab06ff23d616415e82cf174a
4eab946f71a673d98336b0156caa94b3
4ece7216b7b969e64a6c787ea5dda0e1
Abundance
PMI (hrs)
location
External
Internal

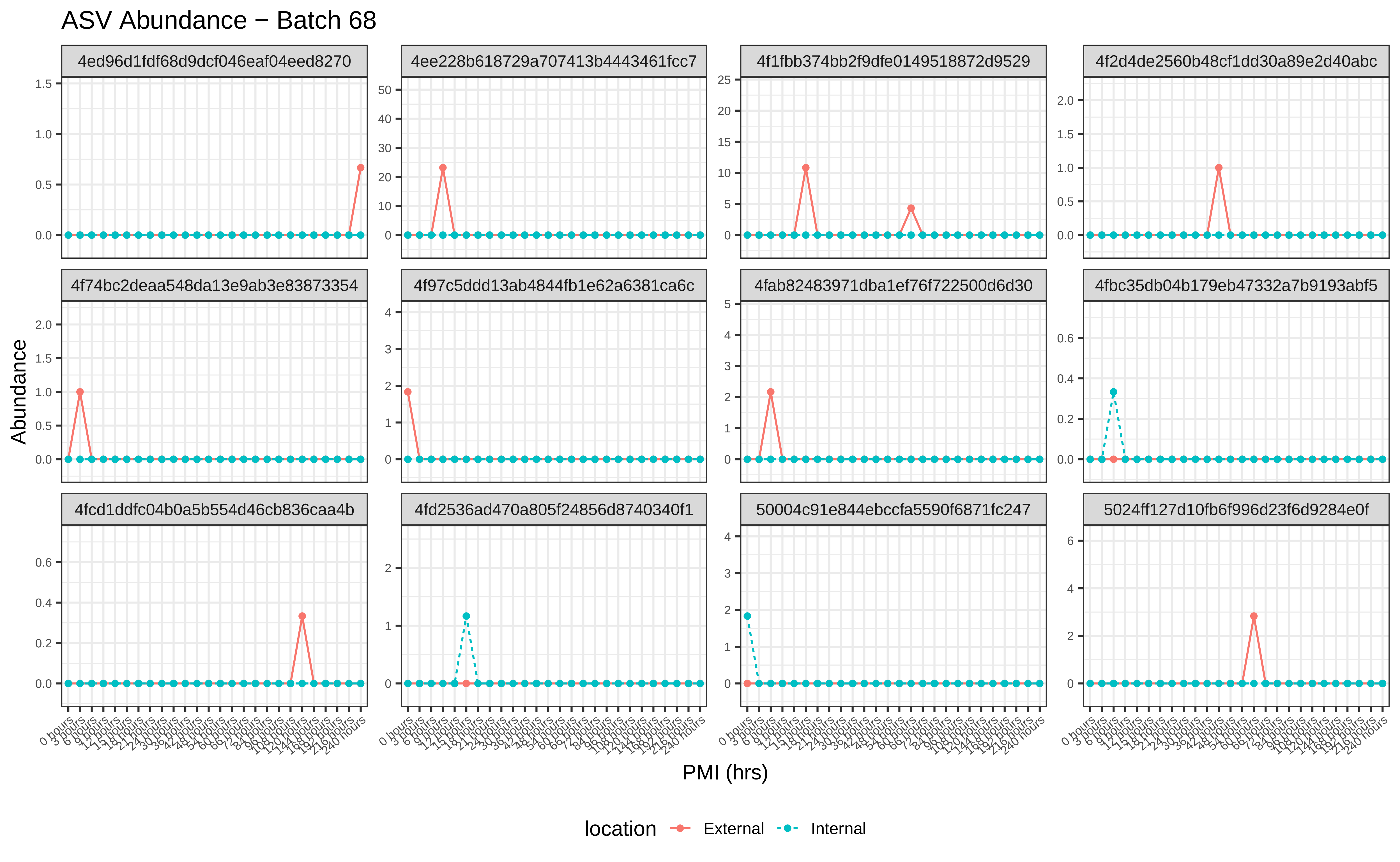
ASV Abundance − Batch 68
4ed96d1fdf68d9dcf046eaf04eed8270
4ee228b618729a707413b4443461fcc7
4f1fbb374bb2f9dfe0149518872d9529
4f2d4de2560b48cf1dd30a89e2d40abc
4f74bc2deaa548da13e9ab3e83873354
4f97c5ddd13ab4844fb1e62a6381ca6c
4fab82483971dba1ef76f722500d6d30
4fbc35db04b179eb47332a7b9193abf5
4fcd1ddfc04b0a5b554d46cb836caa4b
4fd2536ad470a805f24856d8740340f1
50004c91e844ebccfa5590f6871fc247
5024ff127d10fb6f996d23f6d9284e0f
Abundance
PMI (hrs)
location
External
Internal

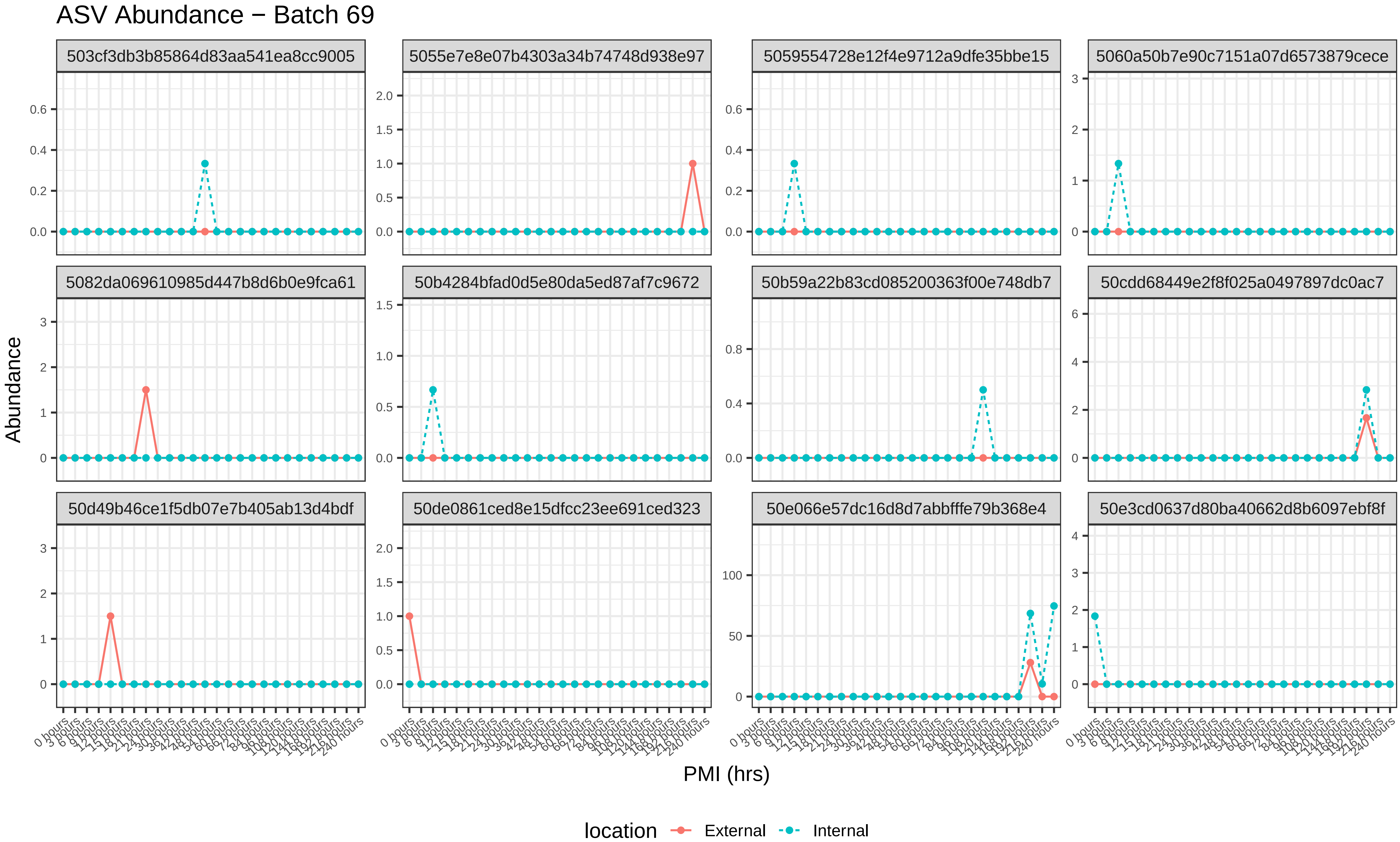
ASV Abundance − Batch 69
503cf3db3b85864d83aa541ea8cc9005
5055e7e8e07b4303a34b74748d938e97
5059554728e12f4e9712a9dfe35bbe15
5060a50b7e90c7151a07d6573879cece
5082da069610985d447b8d6b0e9fca61
50b4284bfad0d5e80da5ed87af7c9672
50b59a22b83cd085200363f00e748db7
50cdd68449e2f8f025a0497897dc0ac7
50d49b46ce1f5db07e7b405ab13d4bdf
50de0861ced8e15dfcc23ee691ced323
50e066e57dc16d8d7abbfffe79b368e4
50e3cd0637d80ba40662d8b6097ebf8f
Abundance
PMI (hrs)
location
External
Internal

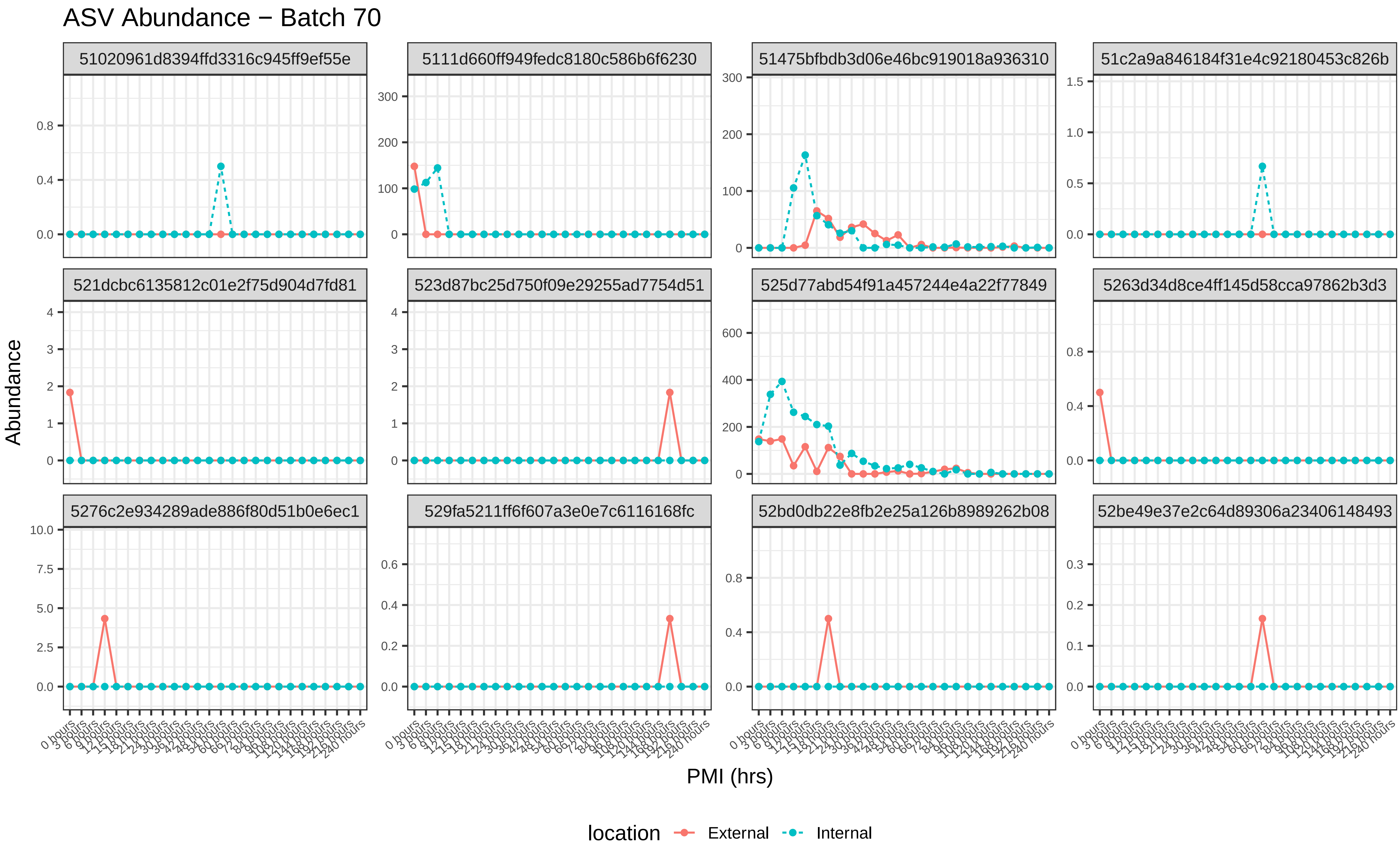
ASV Abundance − Batch 70
51020961d8394ffd3316c945ff9ef55e
5111d660ff949fedc8180c586b6f6230
51475bfbdb3d06e46bc919018a936310
51c2a9a846184f31e4c92180453c826b
521dcbc6135812c01e2f75d904d7fd81
523d87bc25d750f09e29255ad7754d51
525d77abd54f91a457244e4a22f77849
5263d34d8ce4ff145d58cca97862b3d3
5276c2e934289ade886f80d51b0e6ec1
529fa5211ff6f607a3e0e7c6116168fc
52bd0db22e8fb2e25a126b8989262b08
52be49e37e2c64d89306a23406148493
Abundance
PMI (hrs)
location
External
Internal

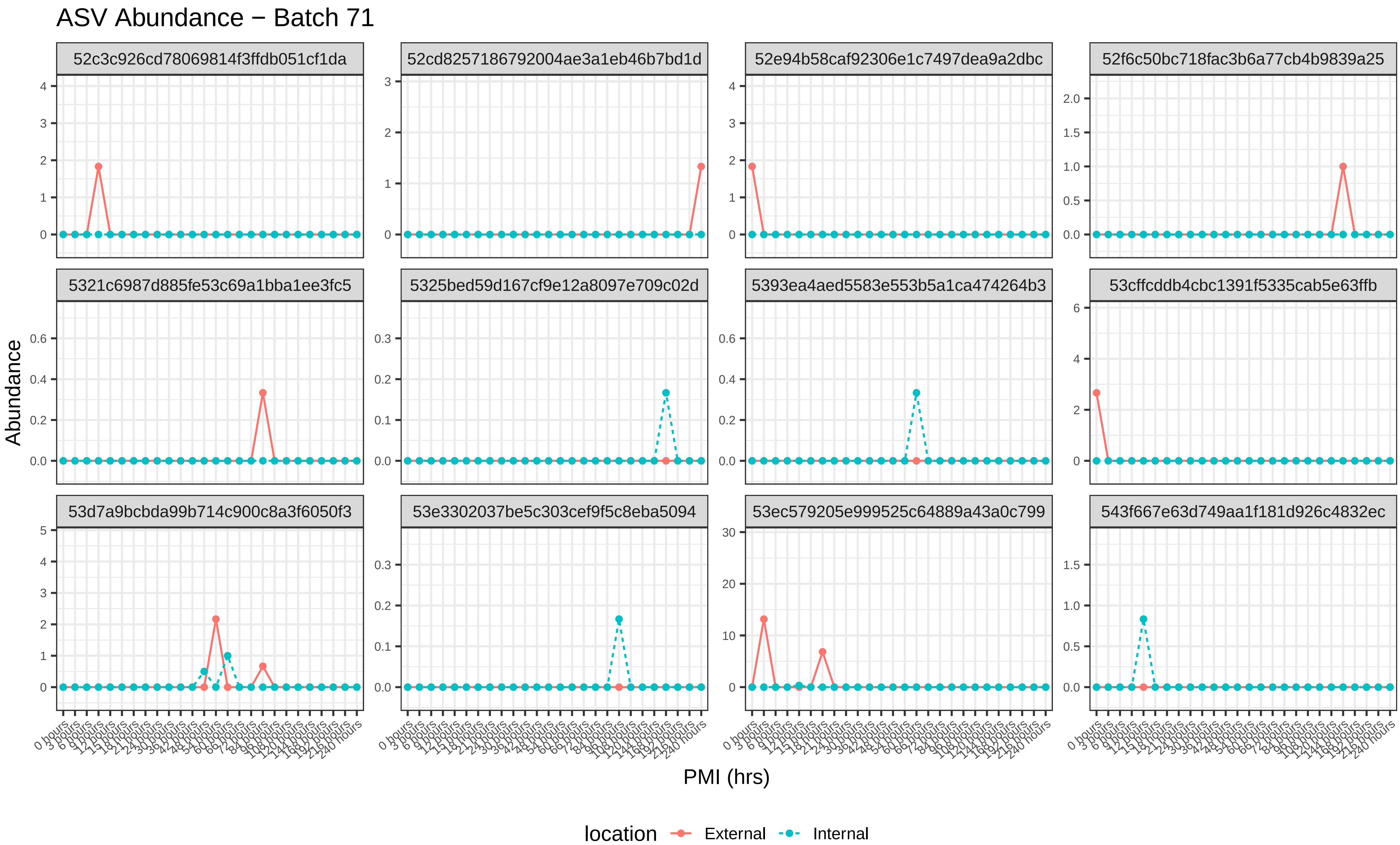

ASV Abundance − Batch 71
52c3c926cd78069814f3ffdb051cf1da
52cd8257186792004ae3a1eb46b7bd1d
52e94b58caf92306e1c7497dea9a2dbc
52f6c50bc718fac3b6a77cb4b9839a25
5321c6987d885fe53c69a1bba1ee3fc5
5325bed59d167cf9e12a8097e709c02d
5393ea4aed5583e553b5a1ca474264b3
53cffcddb4cbc1391f5335cab5e63ffb
53d7a9bcbda99b714c900c8a3f6050f3
53e3302037be5c303cef9f5c8eba5094
53ec579205e999525c64889a43a0c799
543f667e63d749aa1f181d926c4832ec
Abundance
PMI (hrs)
location
External
Internal

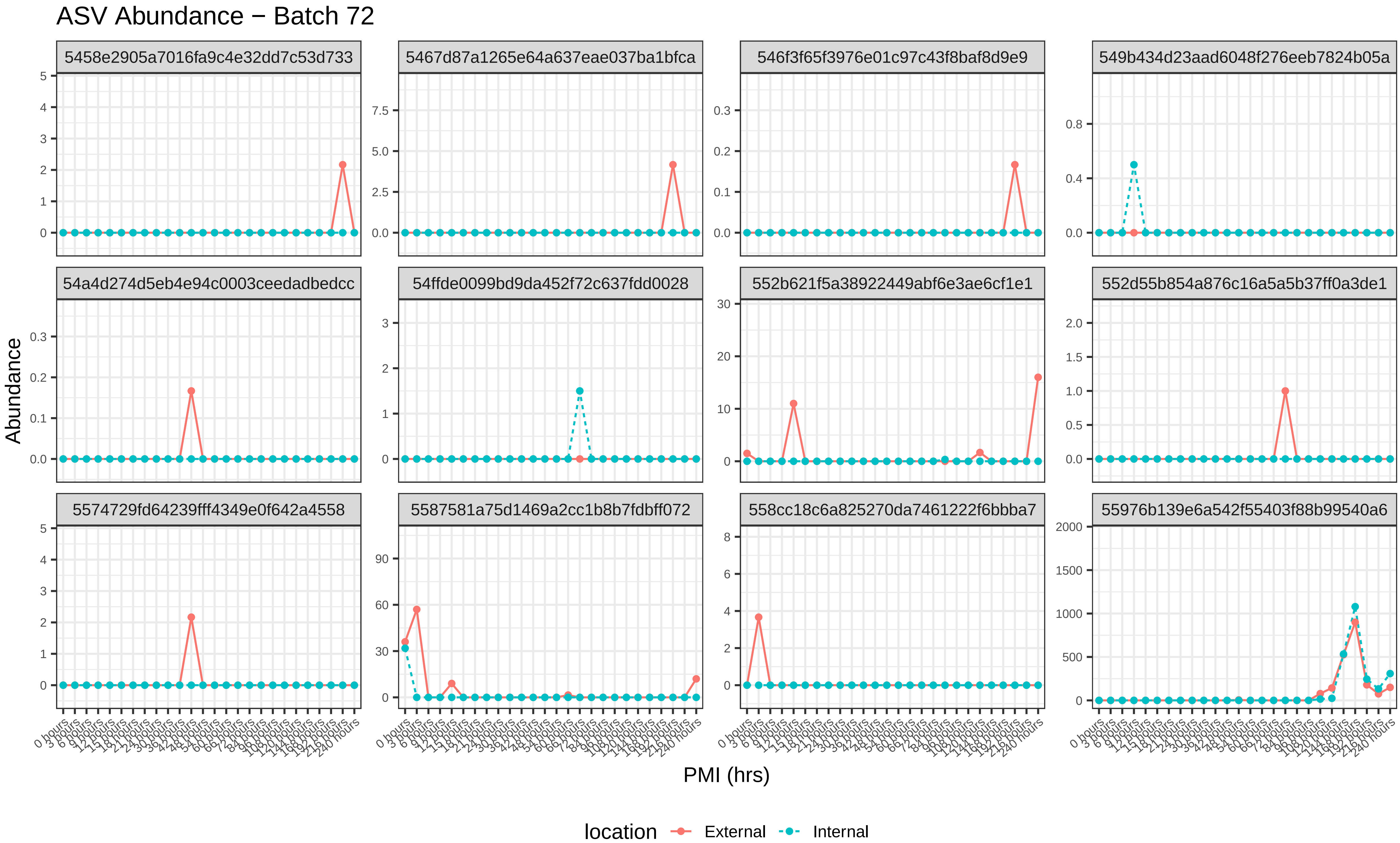
ASV Abundance − Batch 72
5458e2905a7016fa9c4e32dd7c53d733
5467d87a1265e64a637eae037ba1bfca
546f3f65f3976e01c97c43f8baf8d9e9
549b434d23aad6048f276eeb7824b05a
54a4d274d5eb4e94c0003ceedadbedcc
54ffde0099bd9da452f72c637fdd0028
552b621f5a38922449abf6e3ae6cf1e1
552d55b854a876c16a5a5b37ff0a3de1
5574729fd64239fff4349e0f642a4558
5587581a75d1469a2cc1b8b7fdbff072
558cc18c6a825270da7461222f6bbba7
55976b139e6a542f55403f88b99540a6
Abundance
PMI (hrs)
location
External
Internal

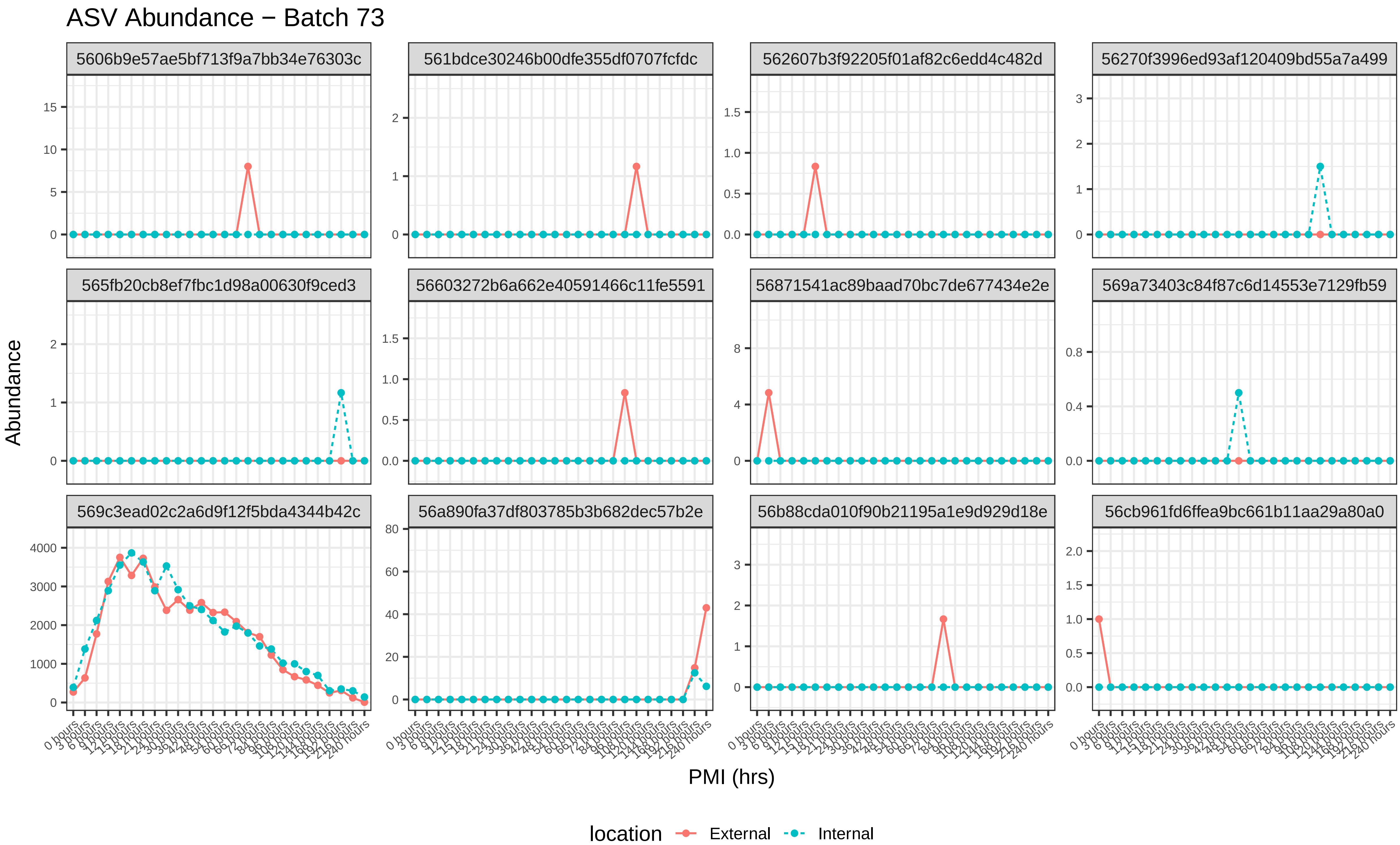

ASV Abundance − Batch 73
5606b9e57ae5bf713f9a7bb34e76303c
561bdce30246b00dfe355df0707fcfdc
562607b3f92205f01af82c6edd4c482d
56270f3996ed93af120409bd55a7a499
565fb20cb8ef7fbc1d98a00630f9ced3
56603272b6a662e40591466c11fe5591
56871541ac89baad70bc7de677434e2e
569a73403c84f87c6d14553e7129fb59
569c3ead02c2a6d9f12f5bda4344b42c
56a890fa37df803785b3b682dec57b2e
56b88cda010f90b21195a1e9d929d18e
56cb961fd6ffea9bc661b11aa29a80a0
Abundance
PMI (hrs)
location
External
Internal

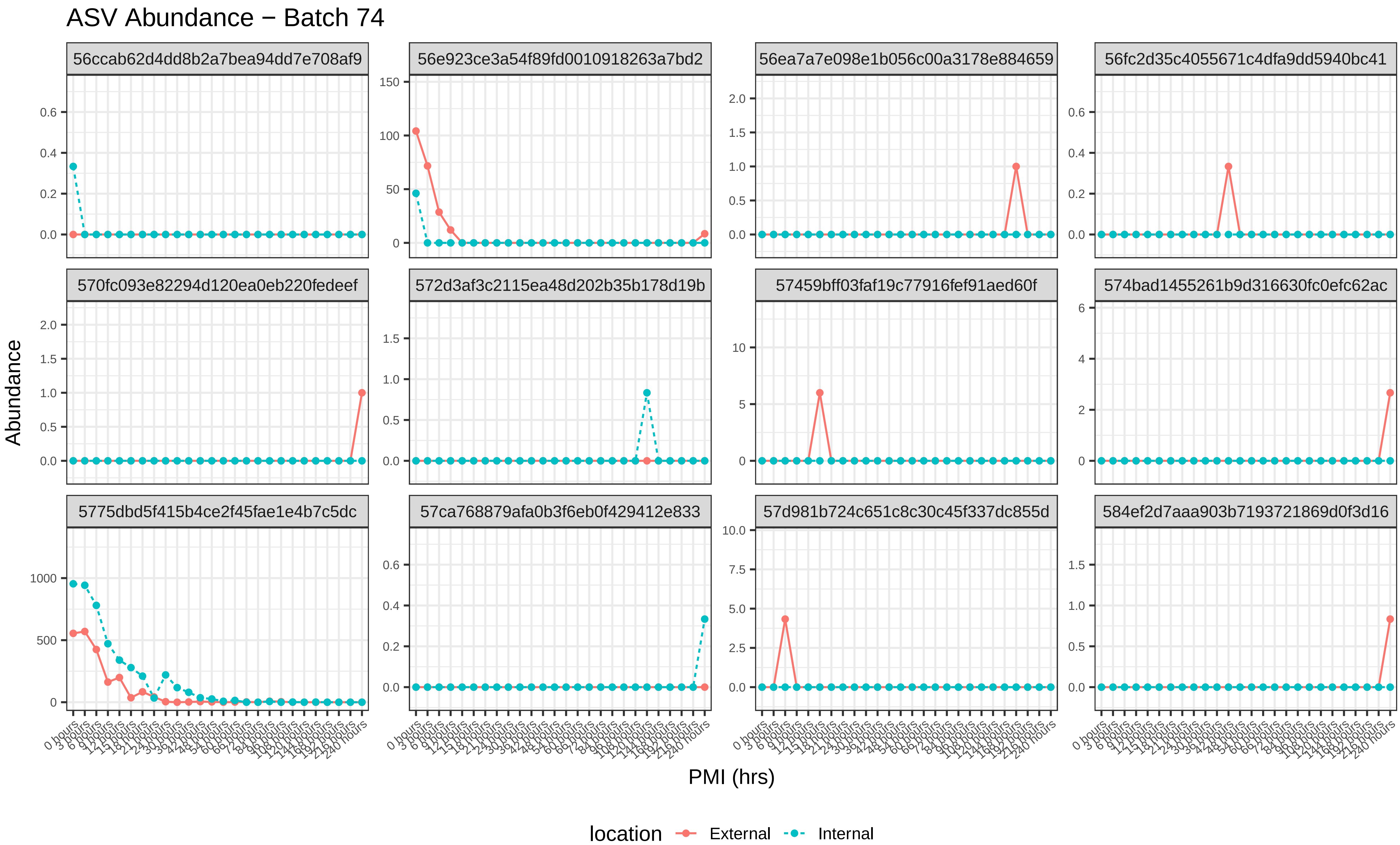
ASV Abundance − Batch 74
56ccab62d4dd8b2a7bea94dd7e708af9
56e923ce3a54f89fd0010918263a7bd2
56ea7a7e098e1b056c00a3178e884659
56fc2d35c4055671c4dfa9dd5940bc41
570fc093e82294d120ea0eb220fedeef
572d3af3c2115ea48d202b35b178d19b
57459bff03faf19c77916fef91aed60f
574bad1455261b9d316630fc0efc62ac
5775dbd5f415b4ce2f45fae1e4b7c5dc
57ca768879afa0b3f6eb0f429412e833
57d981b724c651c8c30c45f337dc855d
584ef2d7aaa903b7193721869d0f3d16
Abundance
PMI (hrs)
location
External
Internal

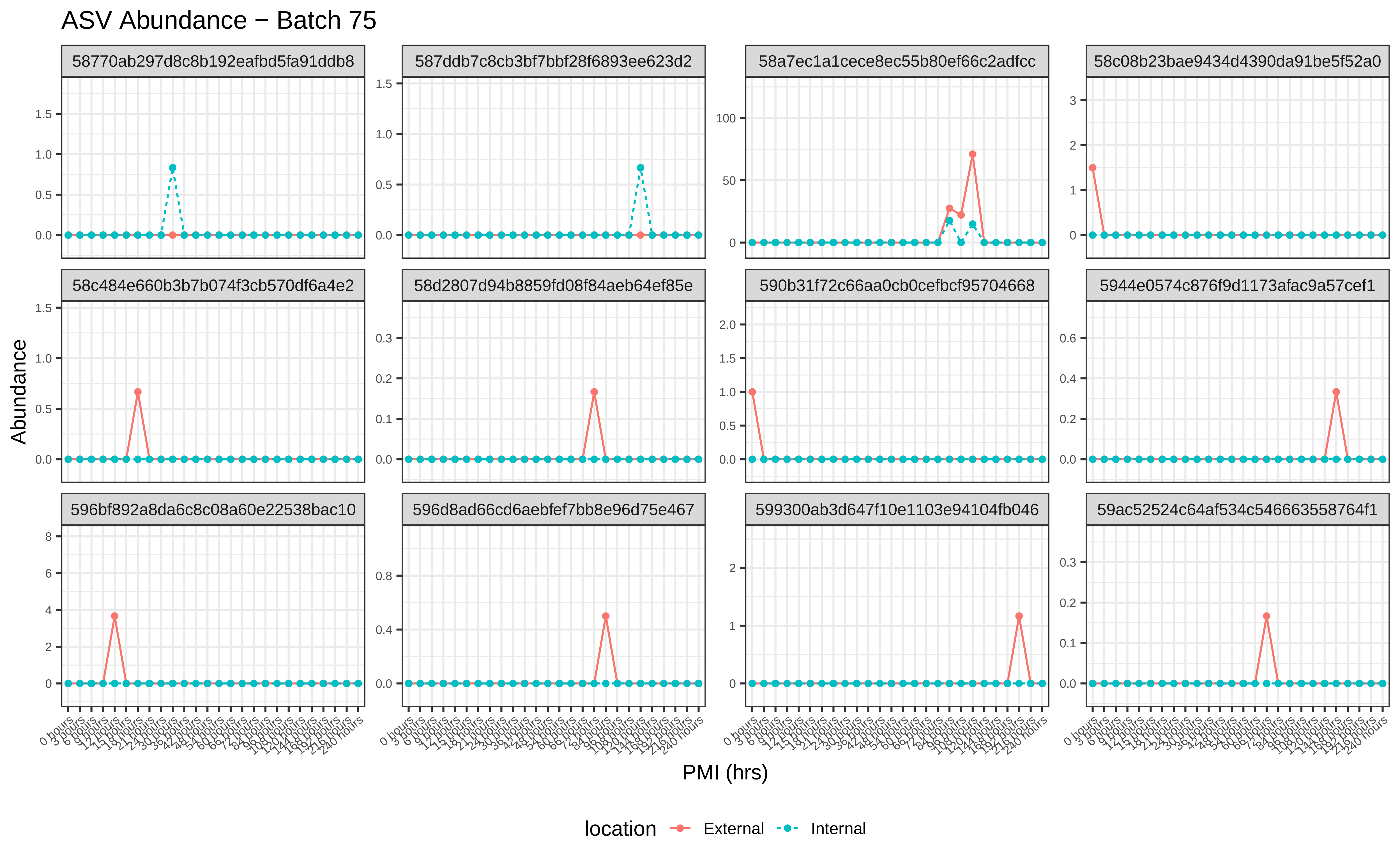

ASV Abundance − Batch 75
58770ab297d8c8b192eafbd5fa91ddb8
587ddb7c8cb3bf7bbf28f6893ee623d2
58a7ec1a1cece8ec55b80ef66c2adfcc
58c08b23bae9434d4390da91be5f52a0
58c484e660b3b7b074f3cb570df6a4e2
58d2807d94b8859fd08f84aeb64ef85e
590b31f72c66aa0cb0cefbcf95704668
5944e0574c876f9d1173afac9a57cef1
596bf892a8da6c8c08a60e22538bac10
596d8ad66cd6aebfef7bb8e96d75e467
599300ab3d647f10e1103e94104fb046
59ac52524c64af534c546663558764f1
Abundance
PMI (hrs)
location
External
Internal

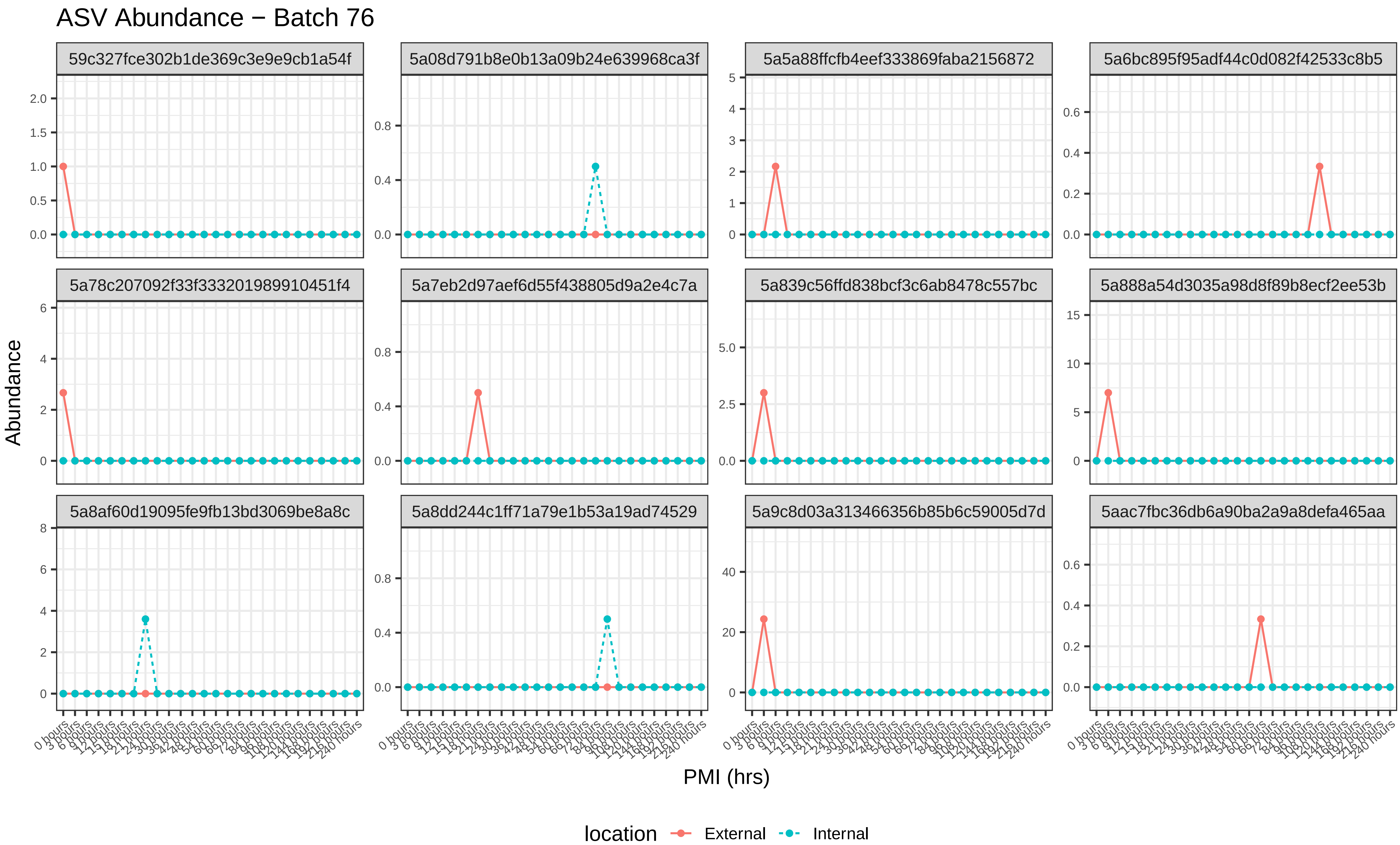
ASV Abundance − Batch 76
59c327fce302b1de369c3e9e9cb1a54f
5a08d791b8e0b13a09b24e639968ca3f
5a5a88ffcfb4eef333869faba2156872
5a6bc895f95adf44c0d082f42533c8b5
5a78c207092f33f333201989910451f4
5a7eb2d97aef6d55f438805d9a2e4c7a
5a839c56ffd838bcf3c6ab8478c557bc
5a888a54d3035a98d8f89b8ecf2ee53b
5a8af60d19095fe9fb13bd3069be8a8c
5a8dd244c1ff71a79e1b53a19ad74529
5a9c8d03a313466356b85b6c59005d7d
5aac7fbc36db6a90ba2a9a8defa465aa
Abundance
PMI (hrs)
location
External
Internal

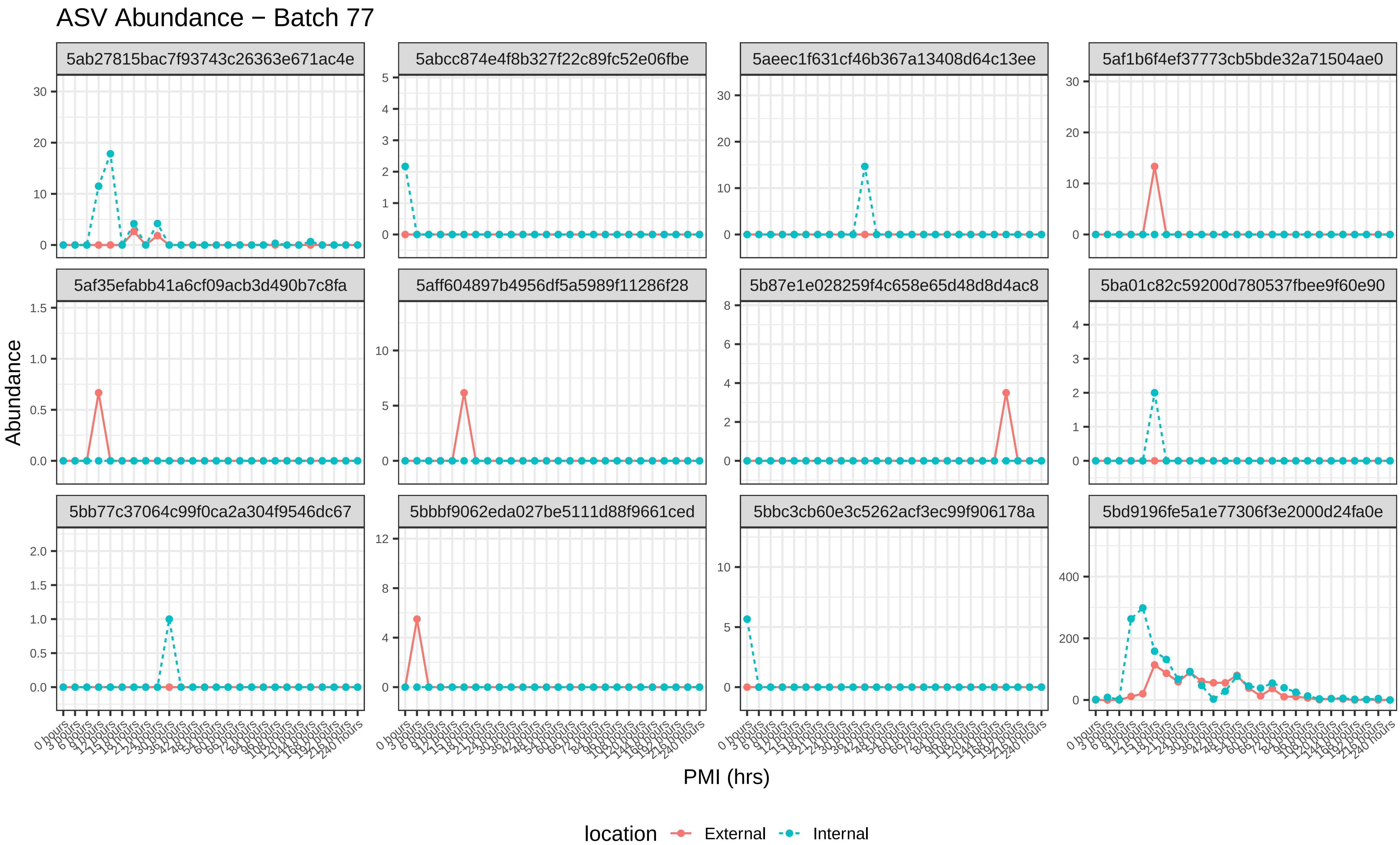

ASV Abundance − Batch 77
5ab27815bac7f93743c26363e671ac4e
5abcc874e4f8b327f22c89fc52e06fbe
5aeec1f631cf46b367a13408d64c13ee
5af1b6f4ef37773cb5bde32a71504ae0
5af35efabb41a6cf09acb3d490b7c8fa
5aff604897b4956df5a5989f11286f28
5b87e1e028259f4c658e65d48d8d4ac8
5ba01c82c59200d780537fbee9f60e90
5bb77c37064c99f0ca2a304f9546dc67
5bbbf9062eda027be5111d88f9661ced
5bbc3cb60e3c5262acf3ec99f906178a
5bd9196fe5a1e77306f3e2000d24fa0e
Abundance
PMI (hrs)
location
External
Internal

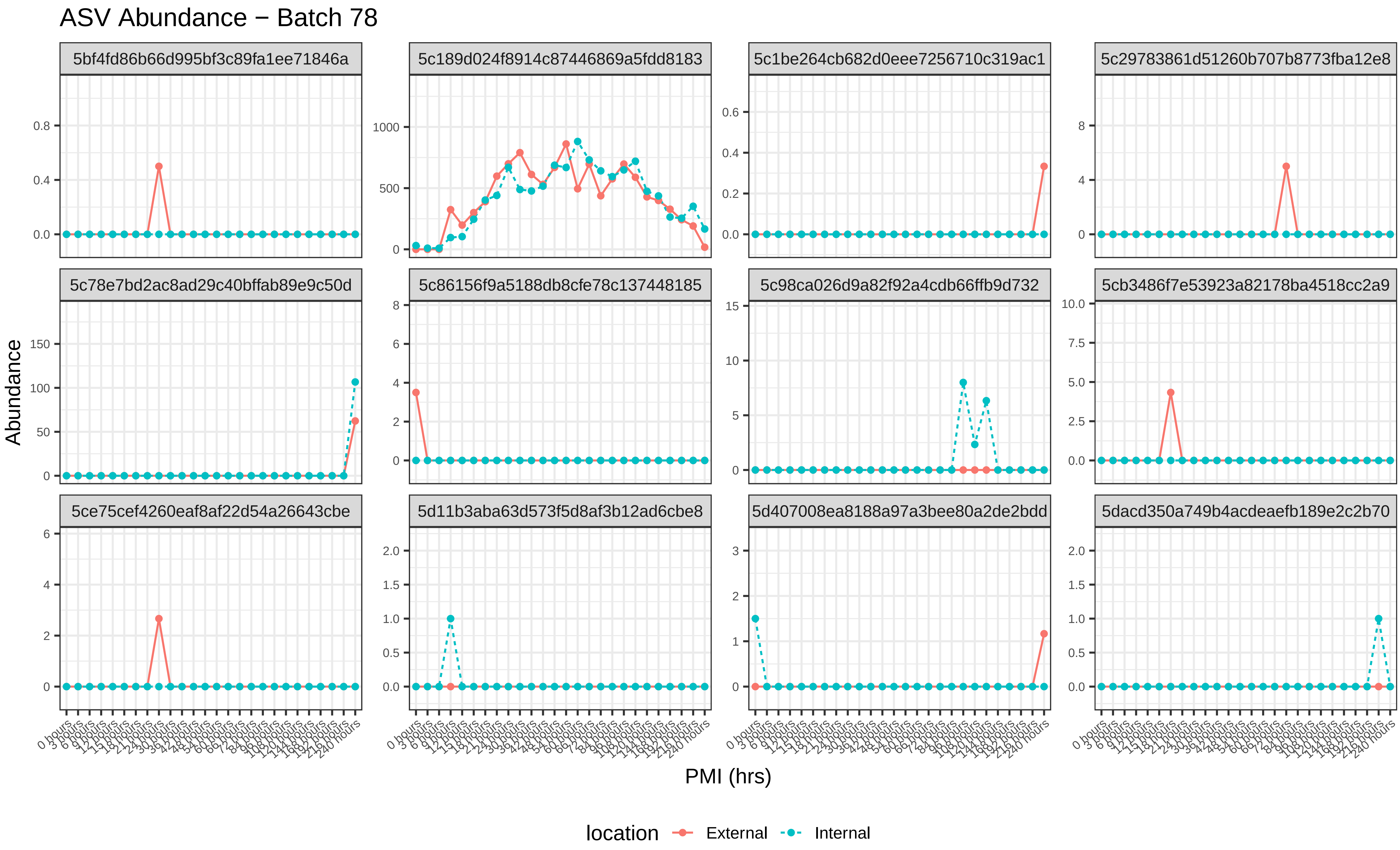
ASV Abundance − Batch 78
5bf4fd86b66d995bf3c89fa1ee71846a
5c189d024f8914c87446869a5fdd8183
5c1be264cb682d0eee7256710c319ac1
5c29783861d51260b707b8773fba12e8
5c78e7bd2ac8ad29c40bffab89e9c50d
5c86156f9a5188db8cfe78c137448185
5c98ca026d9a82f92a4cdb66ffb9d732
5cb3486f7e53923a82178ba4518cc2a9
5ce75cef4260eaf8af22d54a26643cbe
5d11b3aba63d573f5d8af3b12ad6cbe8
5d407008ea8188a97a3bee80a2de2bdd
5dacd350a749b4acdeaefb189e2c2b70
Abundance
PMI (hrs)
location
External
Internal

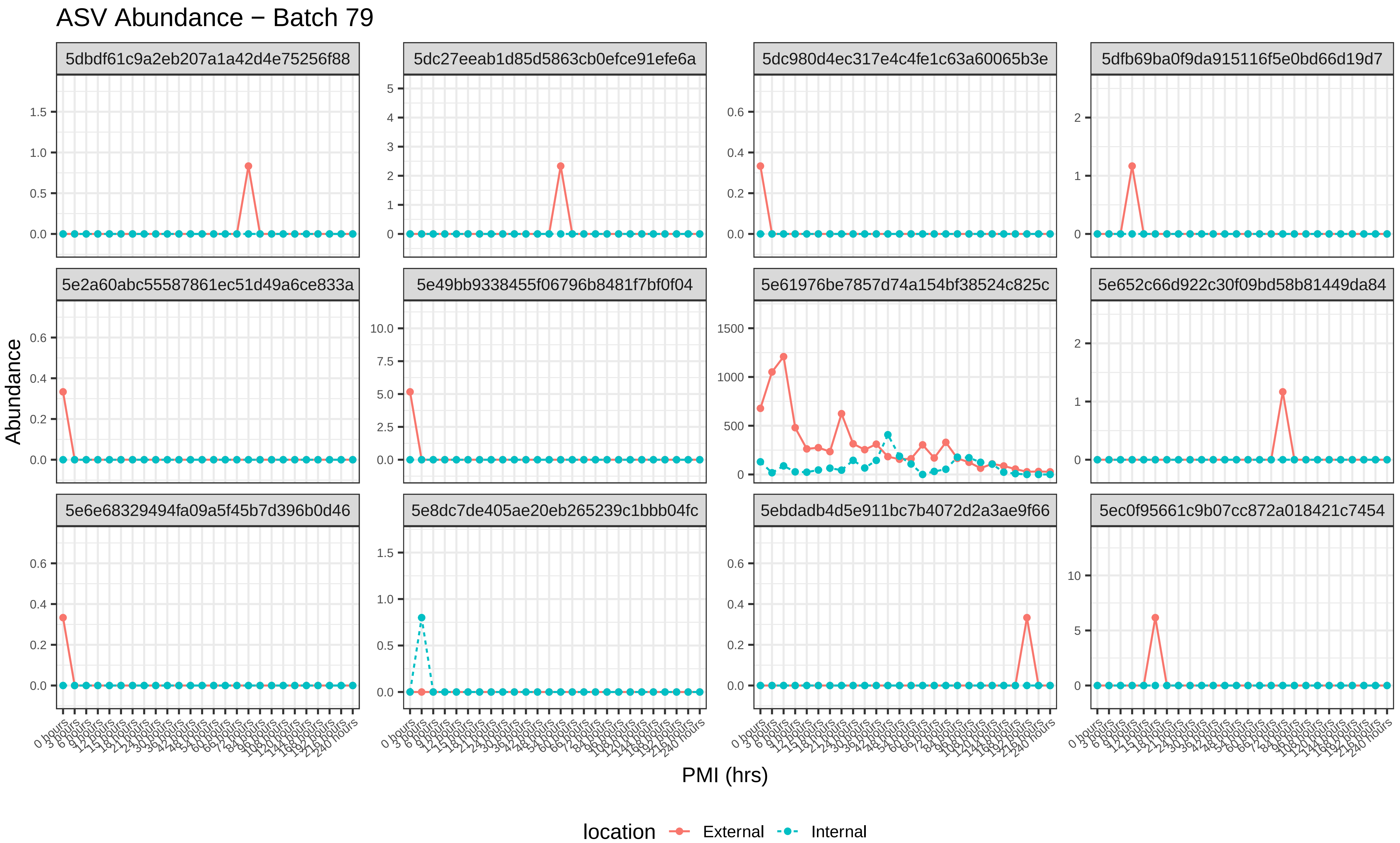
ASV Abundance − Batch 79
5dbdf61c9a2eb207a1a42d4e75256f88
5dc27eeab1d85d5863cb0efce91efe6a
5dc980d4ec317e4c4fe1c63a60065b3e
5dfb69ba0f9da915116f5e0bd66d19d7
5e2a60abc55587861ec51d49a6ce833a
5e49bb9338455f06796b8481f7bf0f04
5e61976be7857d74a154bf38524c825c
5e652c66d922c30f09bd58b81449da84
5e6e68329494fa09a5f45b7d396b0d46
5e8dc7de405ae20eb265239c1bbb04fc
5ebdadb4d5e911bc7b4072d2a3ae9f66
5ec0f95661c9b07cc872a018421c7454
Abundance
PMI (hrs)
location
External
Internal

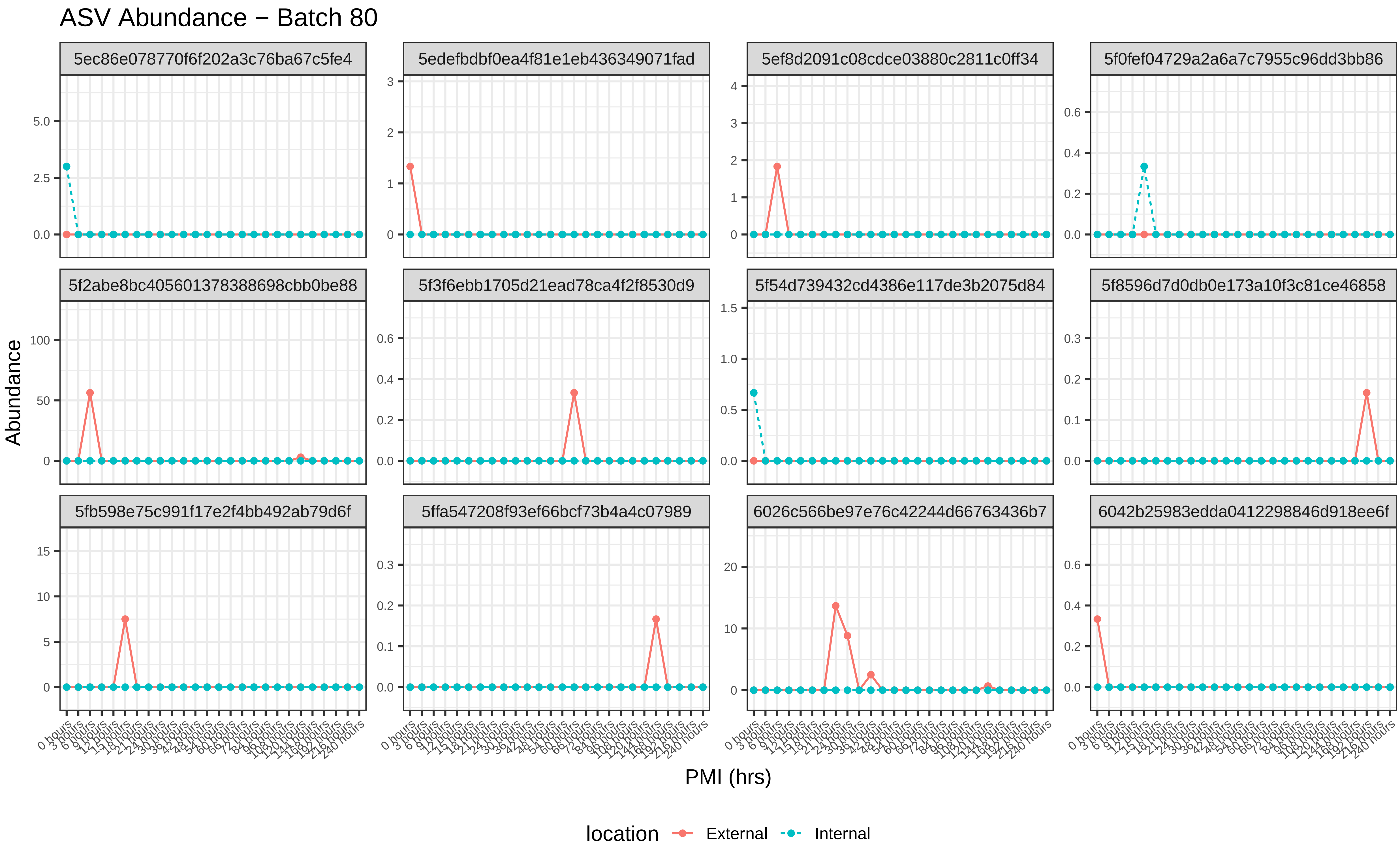

ASV Abundance − Batch 80
5ec86e078770f6f202a3c76ba67c5fe4
5edefbdbf0ea4f81e1eb436349071fad
5ef8d2091c08cdce03880c2811c0ff34
5f0fef04729a2a6a7c7955c96dd3bb86
5f2abe8bc405601378388698cbb0be88
5f3f6ebb1705d21ead78ca4f2f8530d9
5f54d739432cd4386e117de3b2075d84
5f8596d7d0db0e173a10f3c81ce46858
5fb598e75c991f17e2f4bb492ab79d6f
5ffa547208f93ef66bcf73b4a4c07989
6026c566be97e76c42244d66763436b7
6042b25983edda0412298846d918ee6f
Abundance
PMI (hrs)
0 hours
3 hours
6 hours
9 hours
12 hours
15 hours
18 hours
21 hours
24 hours
30 hours
36 hours
42 hours
48 hours
54 hours
60 hours
66 hours
72 hours
84 hours
96 hours
108 hours
120 hours
144 hours
168 hours
192 hours
216 hours
240 hours
location
External
Internal

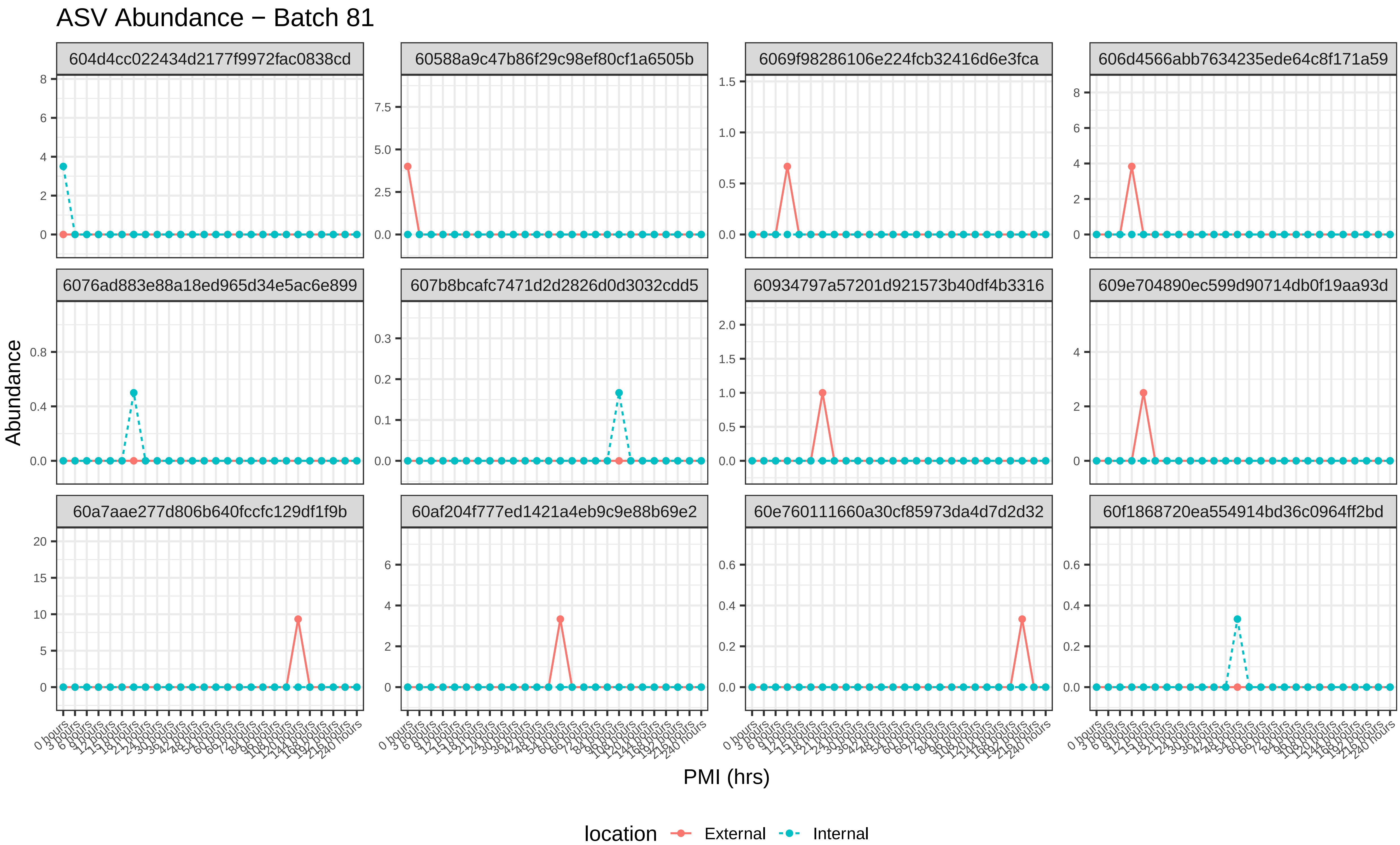

ASV Abundance − Batch 81
604d4cc022434d2177f9972fac0838cd
60588a9c47b86f29c98ef80cf1a6505b
6069f98286106e224fcb32416d6e3fca
606d4566abb7634235ede64c8f171a59
6076ad883e88a18ed965d34e5ac6e899
607b8bcafc7471d2d2826d0d3032cdd5
60934797a57201d921573b40df4b3316
609e704890ec599d90714db0f19aa93d
60a7aae277d806b640fccfc129df1f9b
60af204f777ed1421a4eb9c9e88b69e2
60e760111660a30cf85973da4d7d2d32
60f1868720ea554914bd36c0964ff2bd
Abundance
PMI (hrs)
location
External
Internal

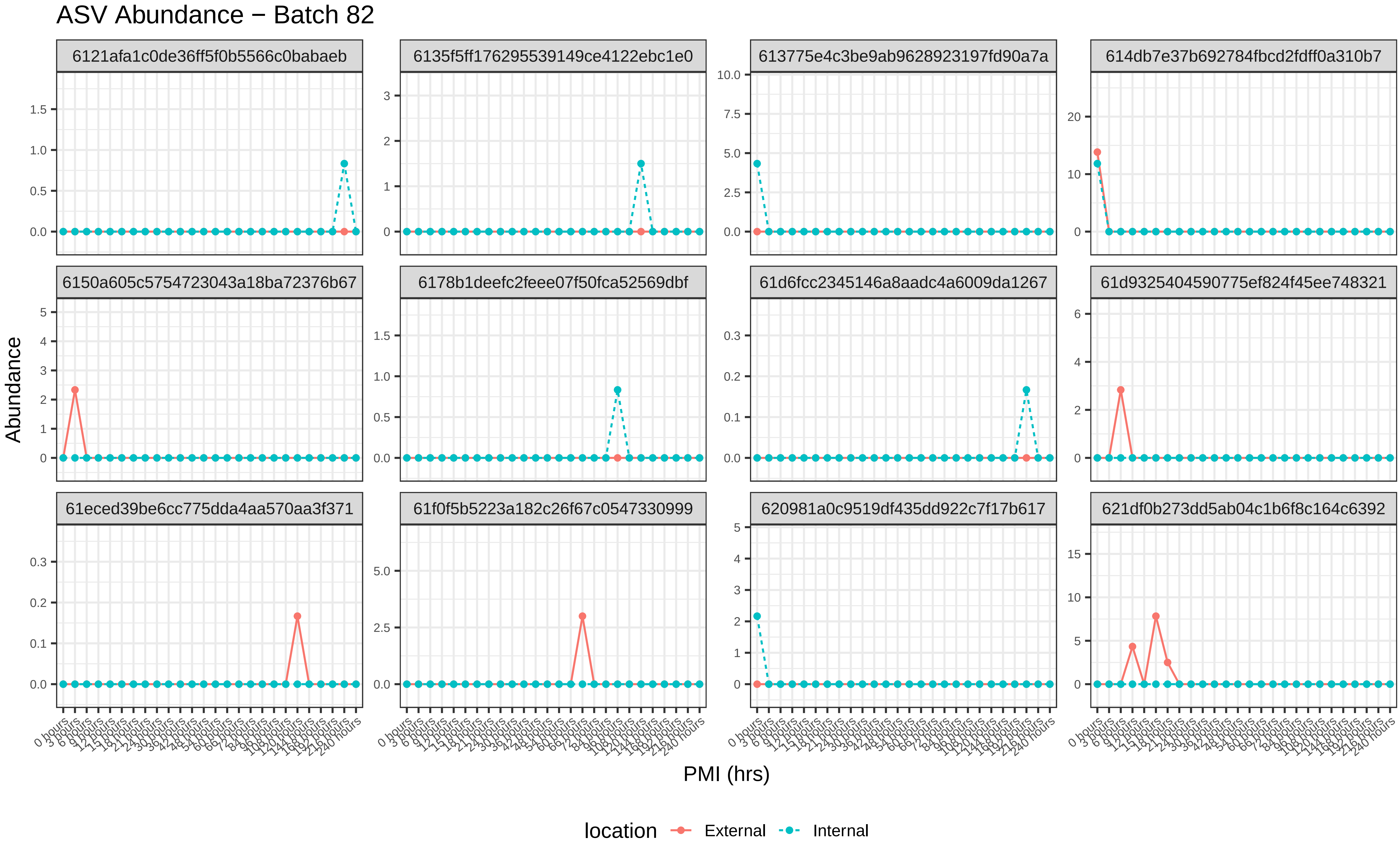

ASV Abundance − Batch 82
6121afa1c0de36ff5f0b5566c0babaeb
6135f5ff176295539149ce4122ebc1e0
613775e4c3be9ab9628923197fd90a7a
614db7e37b692784fbcd2fdff0a310b7
6150a605c5754723043a18ba72376b67
6178b1deefc2feee07f50fca52569dbf
61d6fcc2345146a8aadc4a6009da1267
61d9325404590775ef824f45ee748321
61eced39be6cc775dda4aa570aa3f371
61f0f5b5223a182c26f67c0547330999
620981a0c9519df435dd922c7f17b617
621df0b273dd5ab04c1b6f8c164c6392
Abundance
PMI (hrs)
location
External
Internal

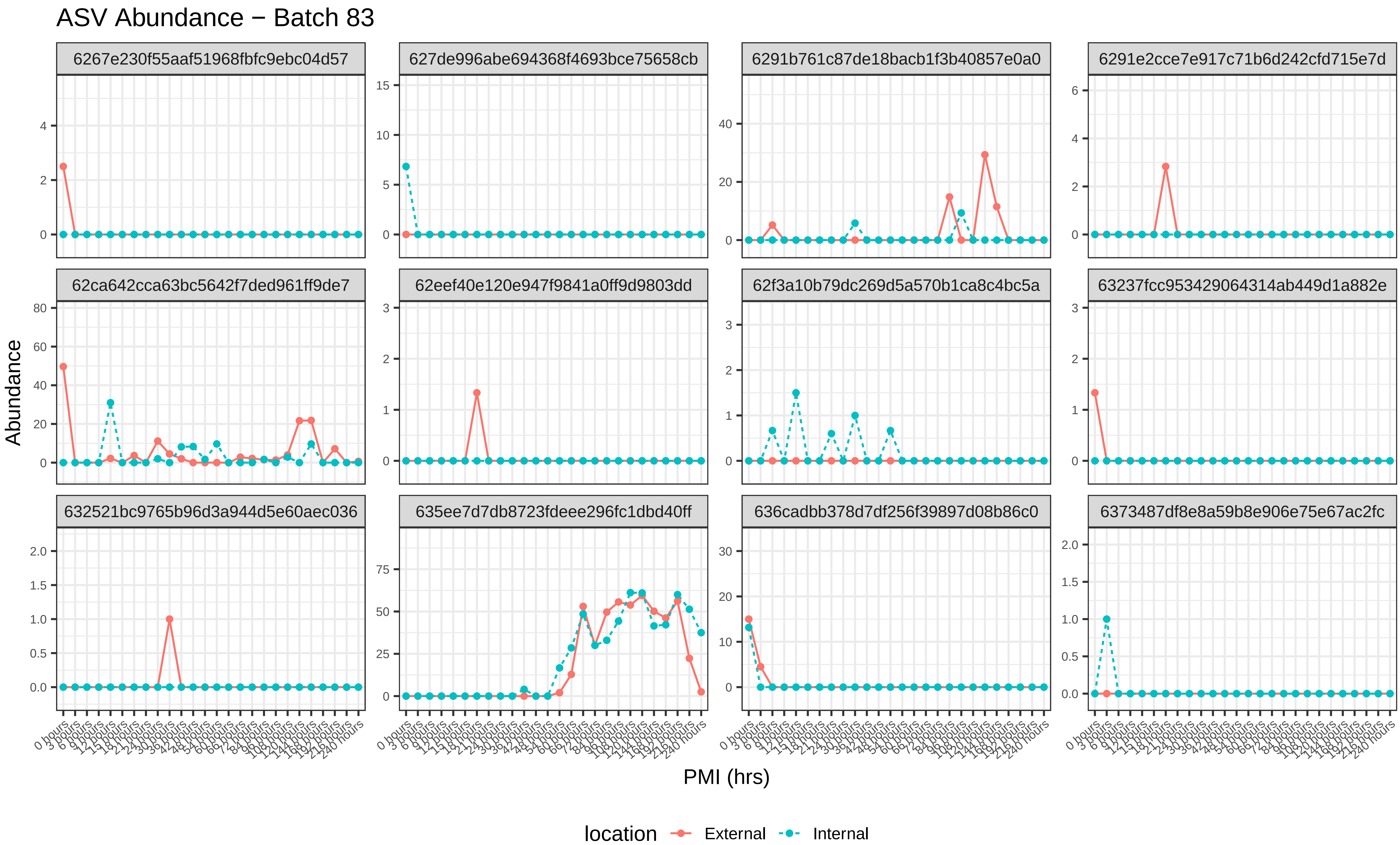
ASV Abundance − Batch 83
6267e230f55aaf51968fbfc9ebc04d57
627de996abe694368f4693bce75658cb
6291b761c87de18bacb1f3b40857e0a0
6291e2cce7e917c71b6d242cfd715e7d
62ca642cca63bc5642f7ded961ff9de7
62eef40e120e947f9841a0ff9d9803dd
62f3a10b79dc269d5a570b1ca8c4bc5a
63237fcc953429064314ab449d1a882e
632521bc9765b96d3a944d5e60aec036
635ee7d7db8723fdeee296fc1dbd40ff
636cadbb378d7df256f39897d08b86c0
6373487df8e8a59b8e906e75e67ac2fc
Abundance
PMI (hrs)
location
External
Internal

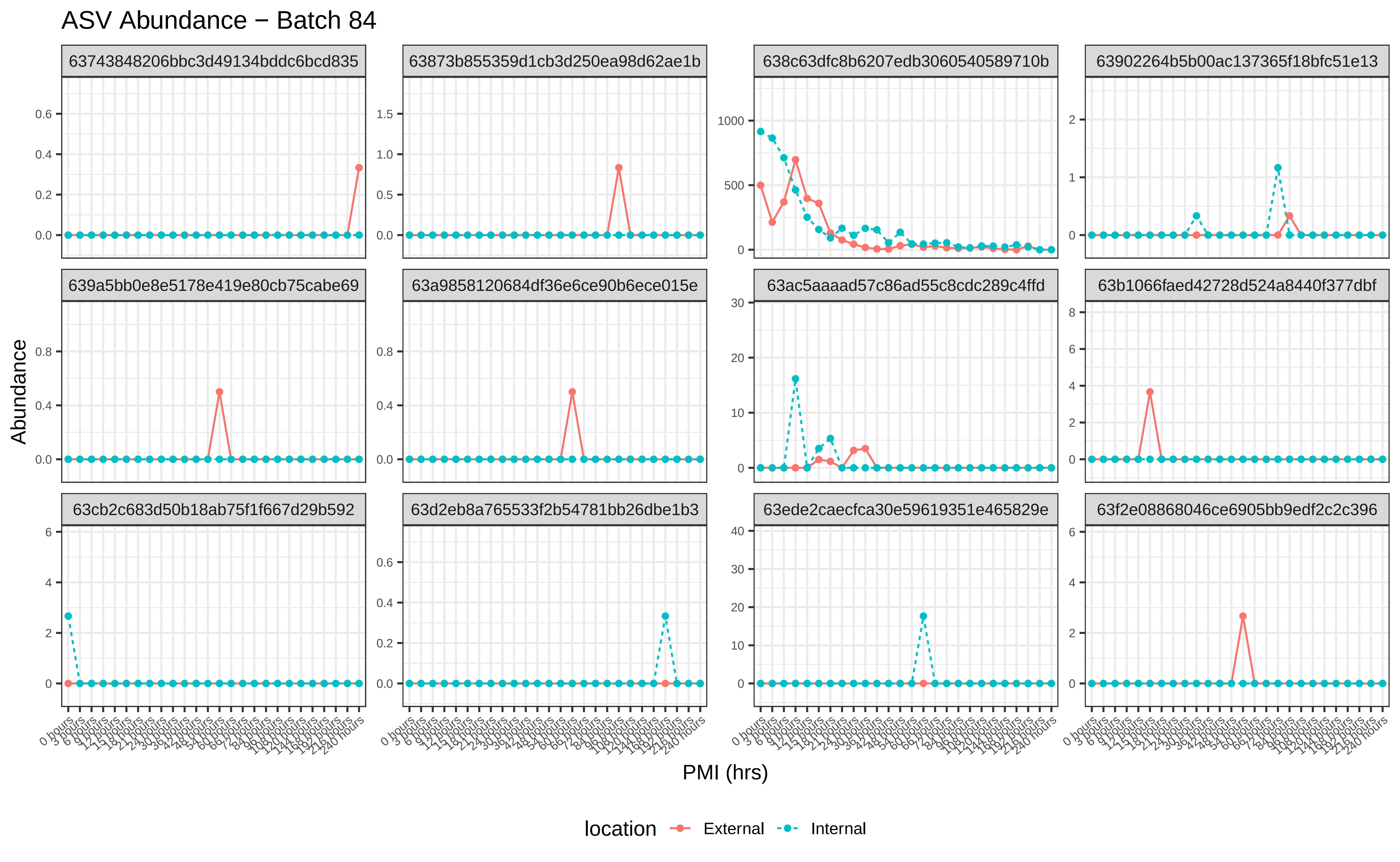

ASV Abundance − Batch 84
63743848206bbc3d49134bddc6bcd835
63873b855359d1cb3d250ea98d62ae1b
638c63dfc8b6207edb3060540589710b
63902264b5b00ac137365f18bfc51e13
639a5bb0e8e5178e419e80cb75cabe69
63a9858120684df36e6ce90b6ece015e
63ac5aaaad57c86ad55c8cdc289c4ffd
63b1066faed42728d524a8440f377dbf
63cb2c683d50b18ab75f1f667d29b592
63d2eb8a765533f2b54781bb26dbe1b3
63ede2caecfca30e59619351e465829e
63f2e08868046ce6905bb9edf2c2c396
Abundance
PMI (hrs)
location
External
Internal

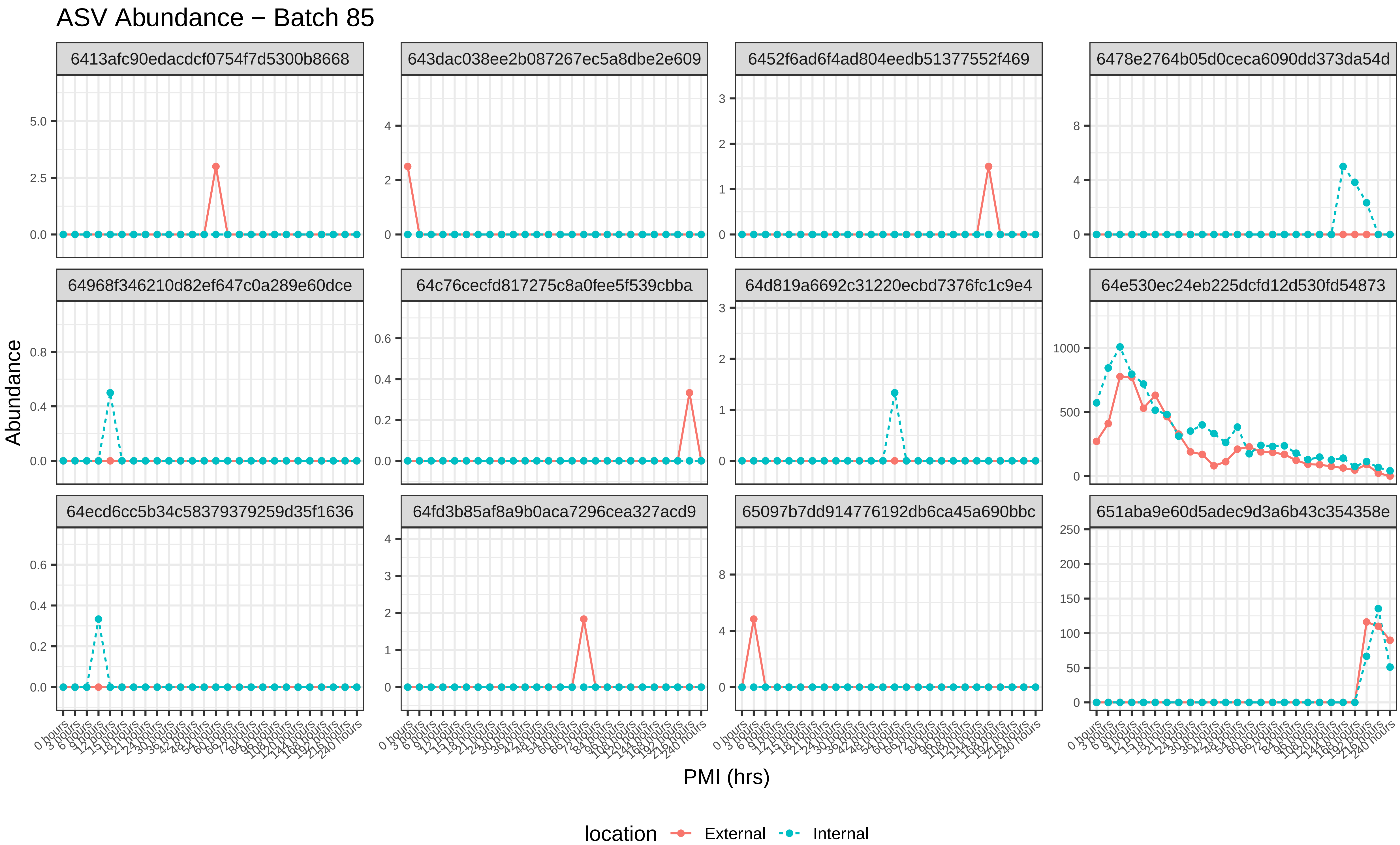

ASV Abundance − Batch 85
6413afc90edacdcf0754f7d5300b8668
643dac038ee2b087267ec5a8dbe2e609
6452f6ad6f4ad804eedb51377552f469
6478e2764b05d0ceca6090dd373da54d
64968f346210d82ef647c0a289e60dce
64c76cecfd817275c8a0fee5f539cbba
64d819a6692c31220ecbd7376fc1c9e4
64e530ec24eb225dcfd12d530fd54873
64ecd6cc5b34c58379379259d35f1636
64fd3b85af8a9b0aca7296cea327acd9
65097b7dd914776192db6ca45a690bbc
651aba9e60d5adec9d3a6b43c354358e
Abundance
PMI (hrs)
location
External
Internal

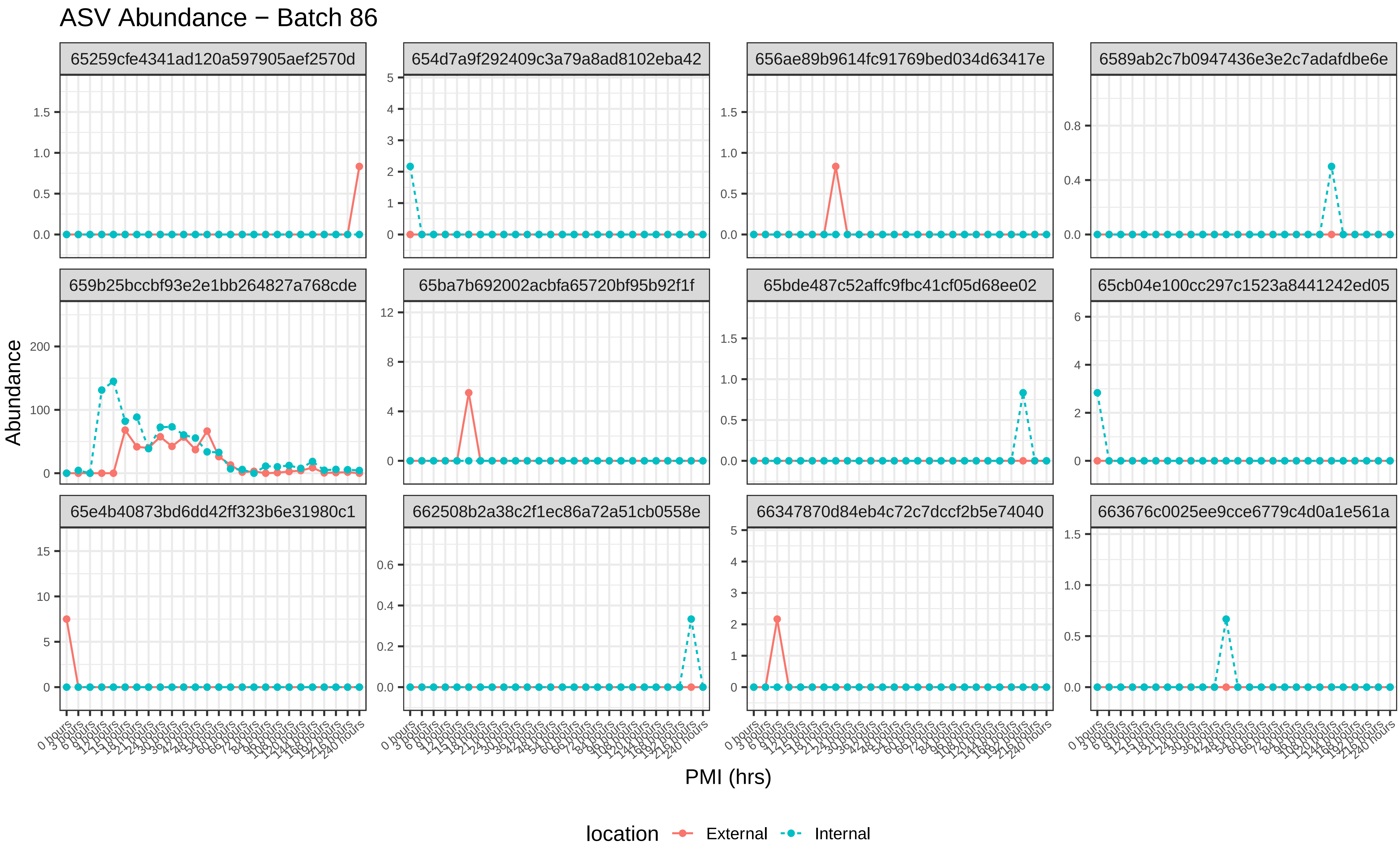

ASV Abundance − Batch 86
65259cfe4341ad120a597905aef2570d
654d7a9f292409c3a79a8ad8102eba42
656ae89b9614fc91769bed034d63417e
6589ab2c7b0947436e3e2c7adafdbe6e
659b25bccbf93e2e1bb264827a768cde
65ba7b692002acbfa65720bf95b92f1f
65bde487c52affc9fbc41cf05d68ee02
65cb04e100cc297c1523a8441242ed05
65e4b40873bd6dd42ff323b6e31980c1
662508b2a38c2f1ec86a72a51cb0558e
66347870d84eb4c72c7dccf2b5e74040
663676c0025ee9cce6779c4d0a1e561a
Abundance
PMI (hrs)
location
External
Internal

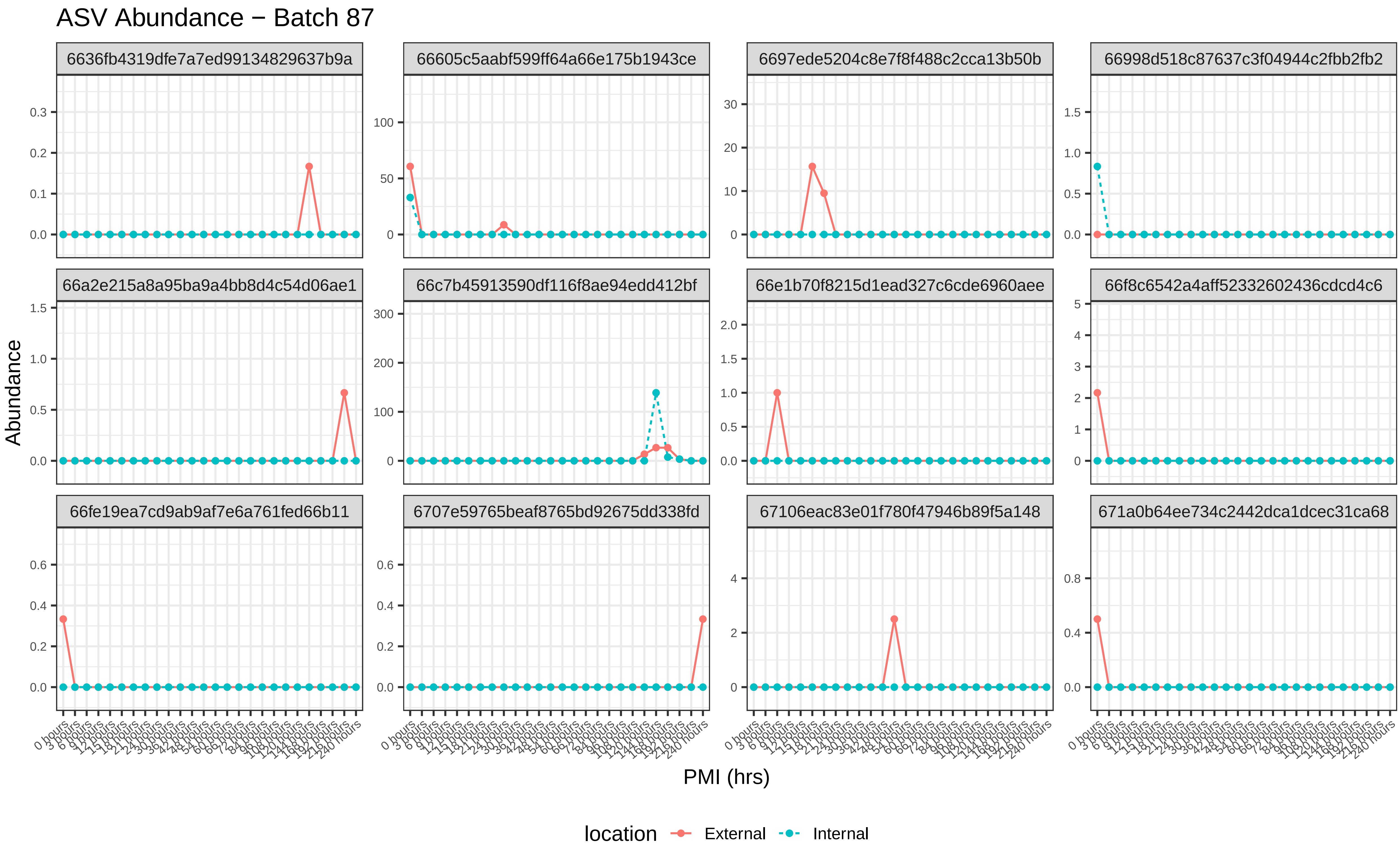
ASV Abundance − Batch 87
6636fb4319dfe7a7ed99134829637b9a
66605c5aabf599ff64a66e175b1943ce
6697ede5204c8e7f8f488c2cca13b50b
66998d518c87637c3f04944c2fbb2fb2
66a2e215a8a95ba9a4bb8d4c54d06ae1
66c7b45913590df116f8ae94edd412bf
66e1b70f8215d1ead327c6cde6960aee
66f8c6542a4aff52332602436cdcd4c6
66fe19ea7cd9ab9af7e6a761fed66b11
6707e59765beaf8765bd92675dd338fd
67106eac83e01f780f47946b89f5a148
671a0b64ee734c2442dca1dcec31ca68
Abundance
PMI (hrs)
location
External
Internal

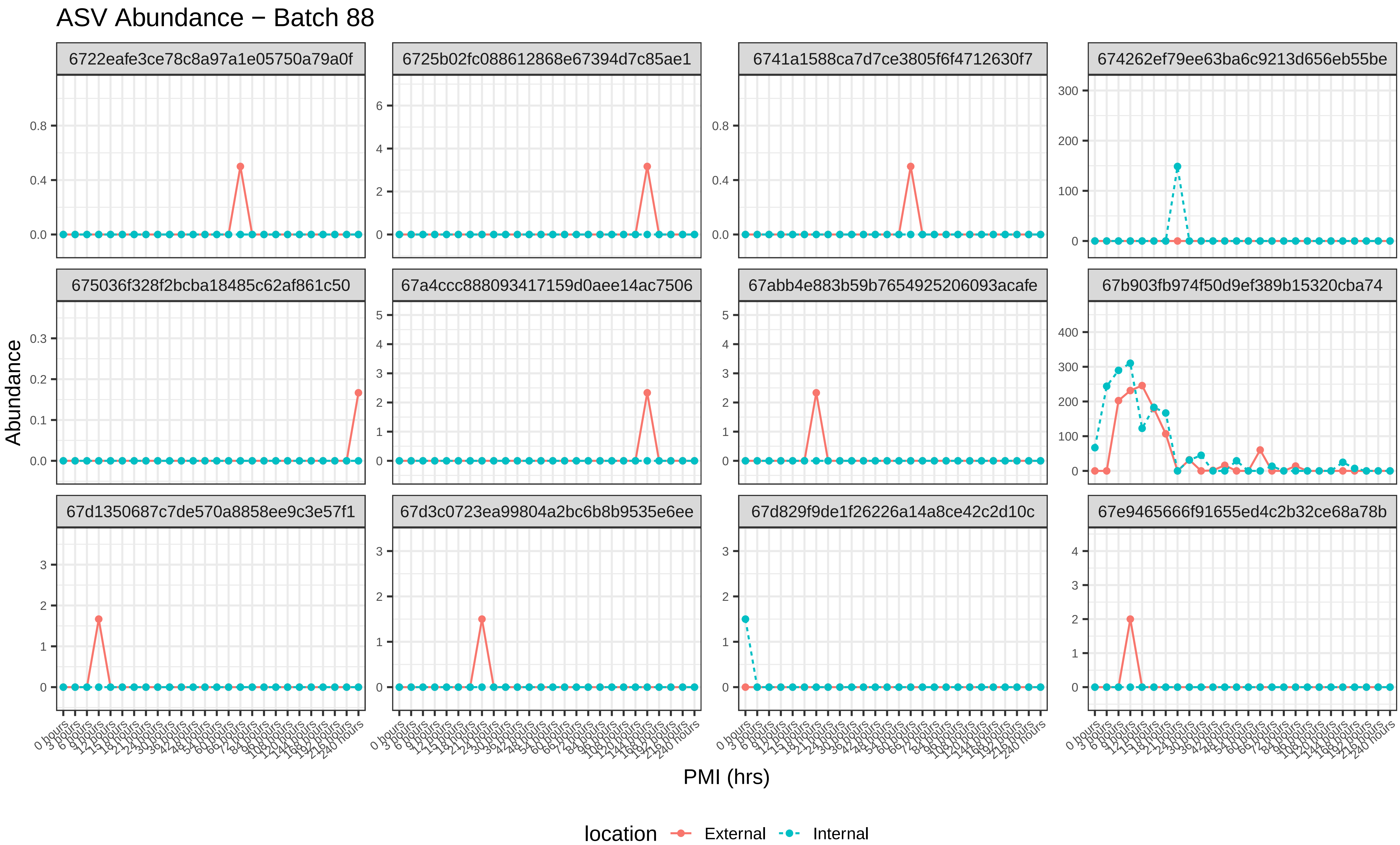

ASV Abundance − Batch 88
6722eafe3ce78c8a97a1e05750a79a0f
6725b02fc088612868e67394d7c85ae1
6741a1588ca7d7ce3805f6f4712630f7
674262ef79ee63ba6c9213d656eb55be
675036f328f2bcba18485c62af861c50
67a4ccc888093417159d0aee14ac7506
67abb4e883b59b7654925206093acafe
67b903fb974f50d9ef389b15320cba74
67d1350687c7de570a8858ee9c3e57f1
67d3c0723ea99804a2bc6b8b9535e6ee
67d829f9de1f26226a14a8ce42c2d10c
67e9465666f91655ed4c2b32ce68a78b
Abundance
PMI (hrs)
location
External
Internal

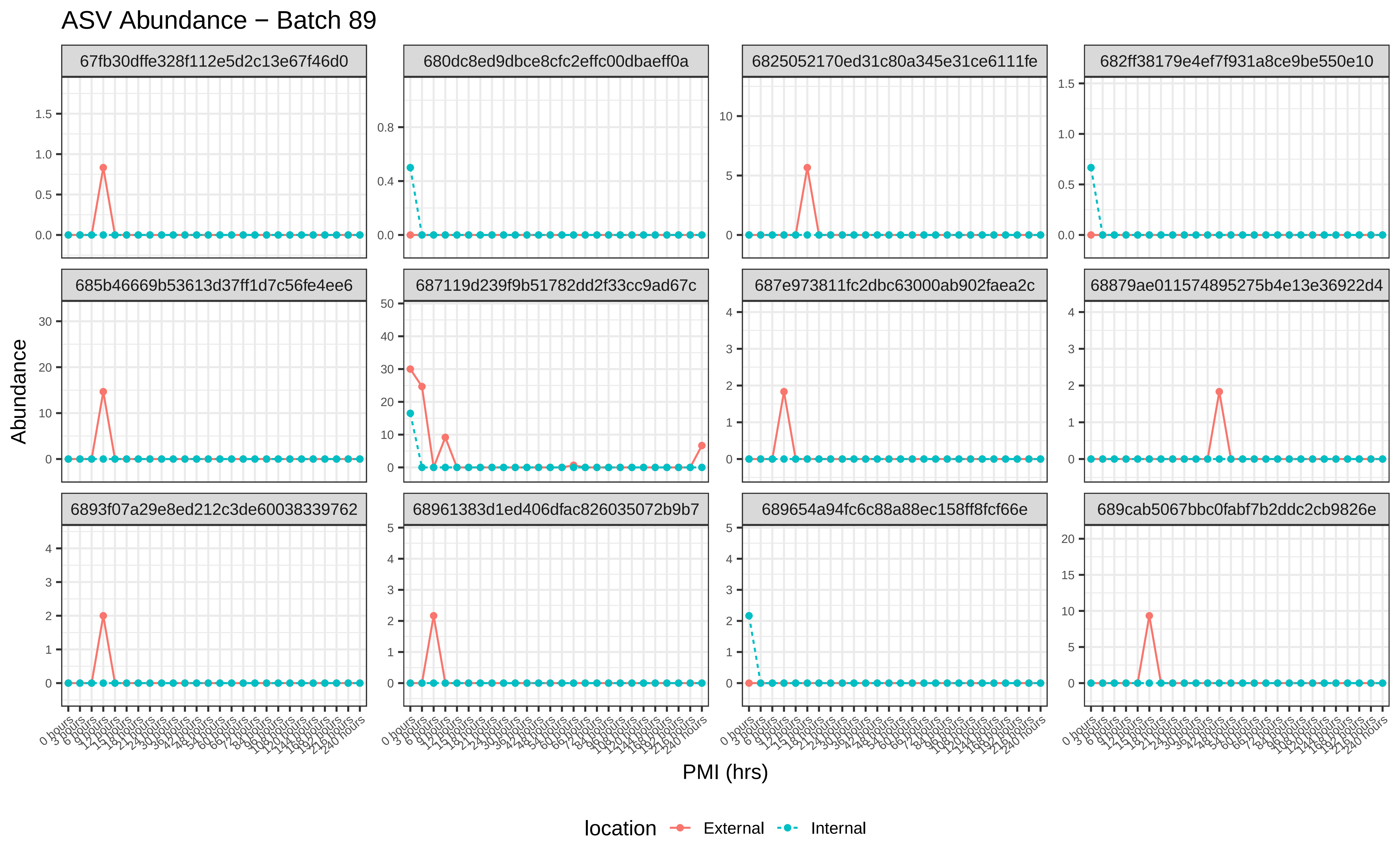
ASV Abundance − Batch 89
67fb30dffe328f112e5d2c13e67f46d0
680dc8ed9dbce8cfc2effc00dbaeff0a
6825052170ed31c80a345e31ce6111fe
682ff38179e4ef7f931a8ce9be550e10
685b46669b53613d37ff1d7c56fe4ee6
687119d239f9b51782dd2f33cc9ad67c
687e973811fc2dbc63000ab902faea2c
68879ae011574895275b4e13e36922d4
6893f07a29e8ed212c3de60038339762
68961383d1ed406dfac826035072b9b7
689654a94fc6c88a88ec158ff8fcf66e
689cab5067bbc0fabf7b2ddc2cb9826e
Abundance
PMI (hrs)
location
External
Internal

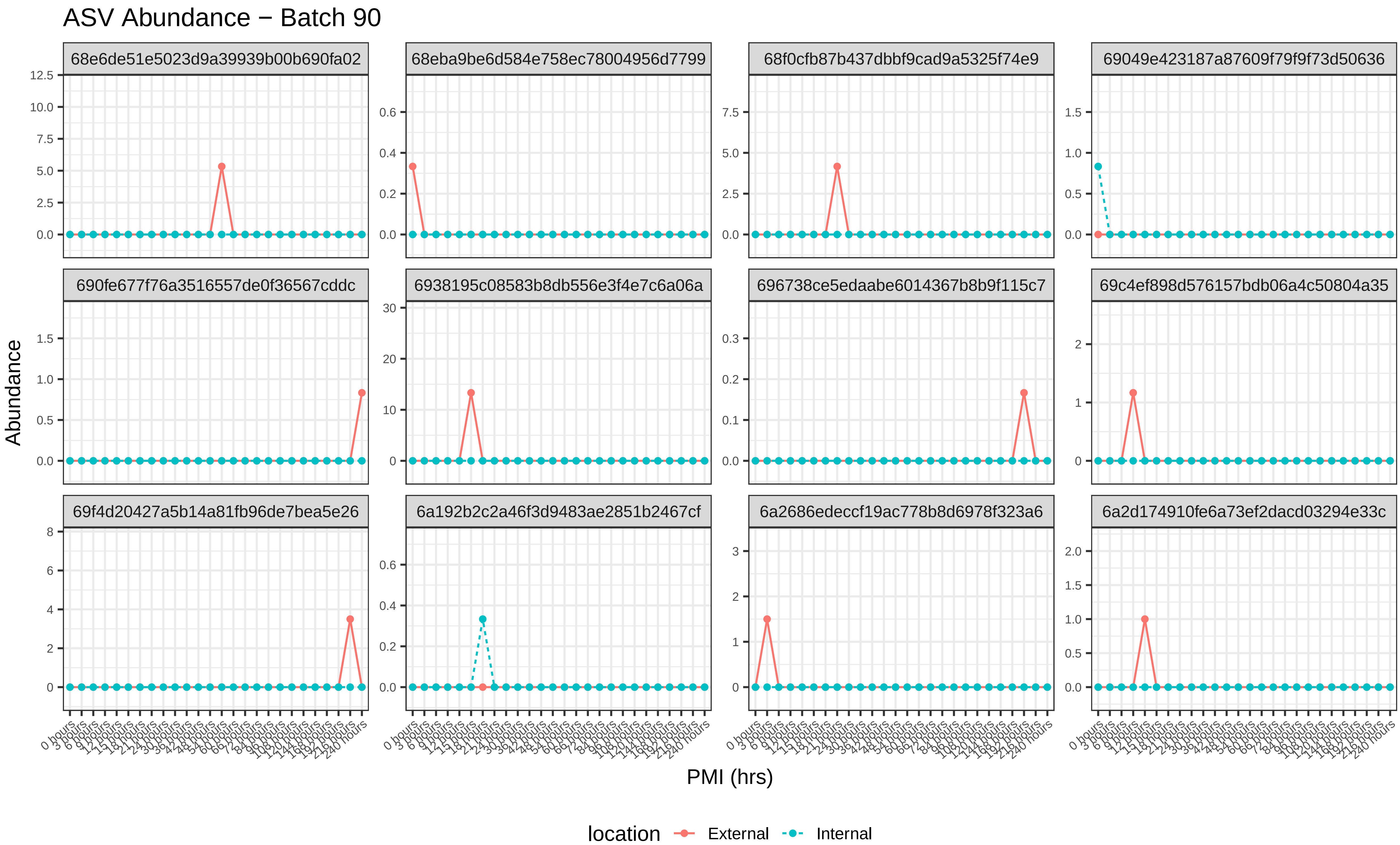
ASV Abundance − Batch 90
68e6de51e5023d9a39939b00b690fa02
68eba9be6d584e758ec78004956d7799
68f0cfb87b437dbbf9cad9a5325f74e9
69049e423187a87609f79f9f73d50636
690fe677f76a3516557de0f36567cddc
6938195c08583b8db556e3f4e7c6a06a
696738ce5edaabe6014367b8b9f115c7
69c4ef898d576157bdb06a4c50804a35
69f4d20427a5b14a81fb96de7bea5e26
6a192b2c2a46f3d9483ae2851b2467cf
6a2686edeccf19ac778b8d6978f323a6
6a2d174910fe6a73ef2dacd03294e33c
Abundance
PMI (hrs)
location
External
Internal

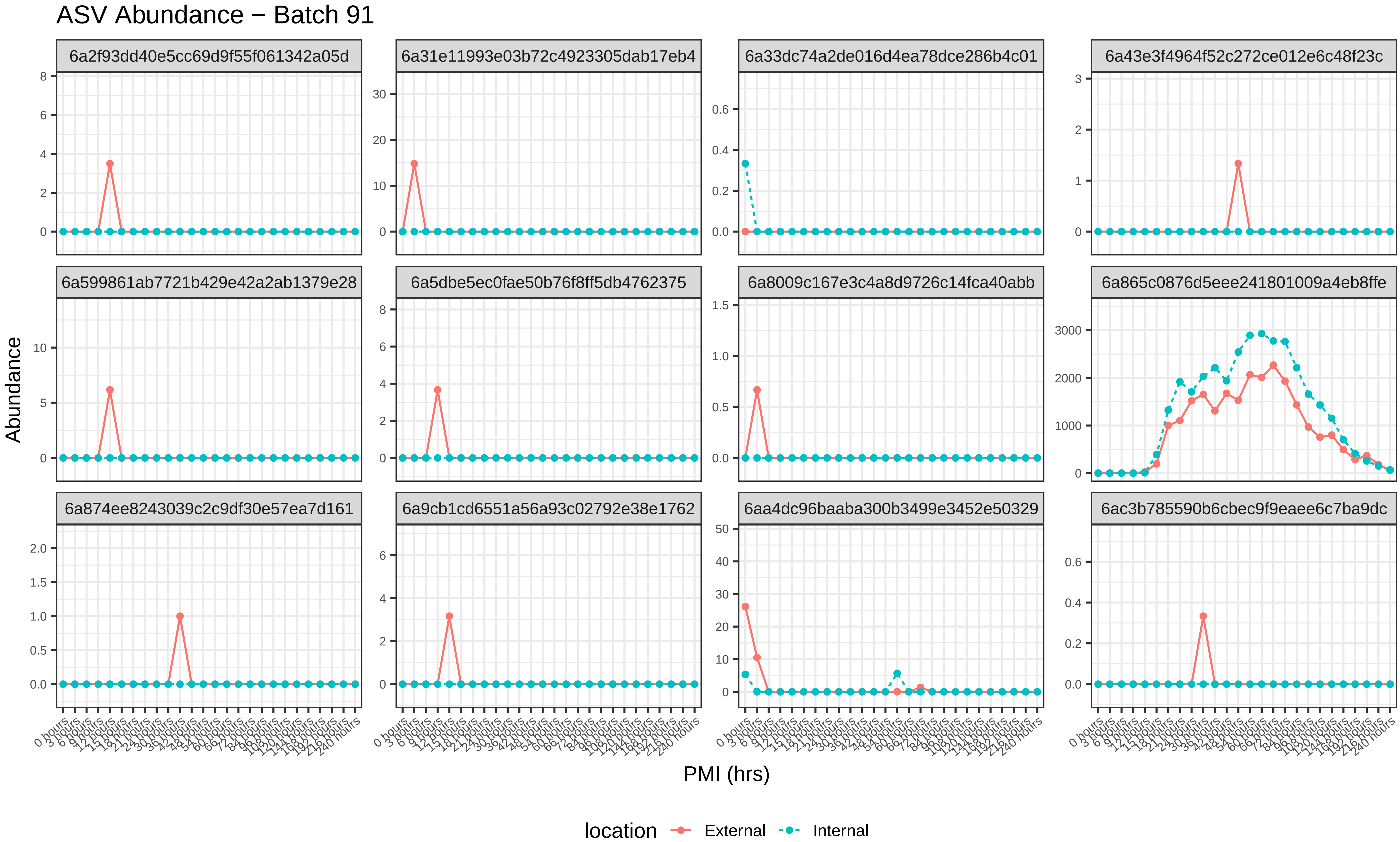

ASV Abundance − Batch 91
6a2f93dd40e5cc69d9f55f061342a05d
6a31e11993e03b72c4923305dab17eb4
6a33dc74a2de016d4ea78dce286b4c01
6a43e3f4964f52c272ce012e6c48f23c
6a599861ab7721b429e42a2ab1379e28
6a5dbe5ec0fae50b76f8ff5db4762375
6a8009c167e3c4a8d9726c14fca40abb
6a865c0876d5eee241801009a4eb8ffe
6a874ee8243039c2c9df30e57ea7d161
6a9cb1cd6551a56a93c02792e38e1762
6aa4dc96baaba300b3499e3452e50329
6ac3b785590b6cbec9f9eaee6c7ba9dc
Abundance
PMI (hrs)
location
External
Internal

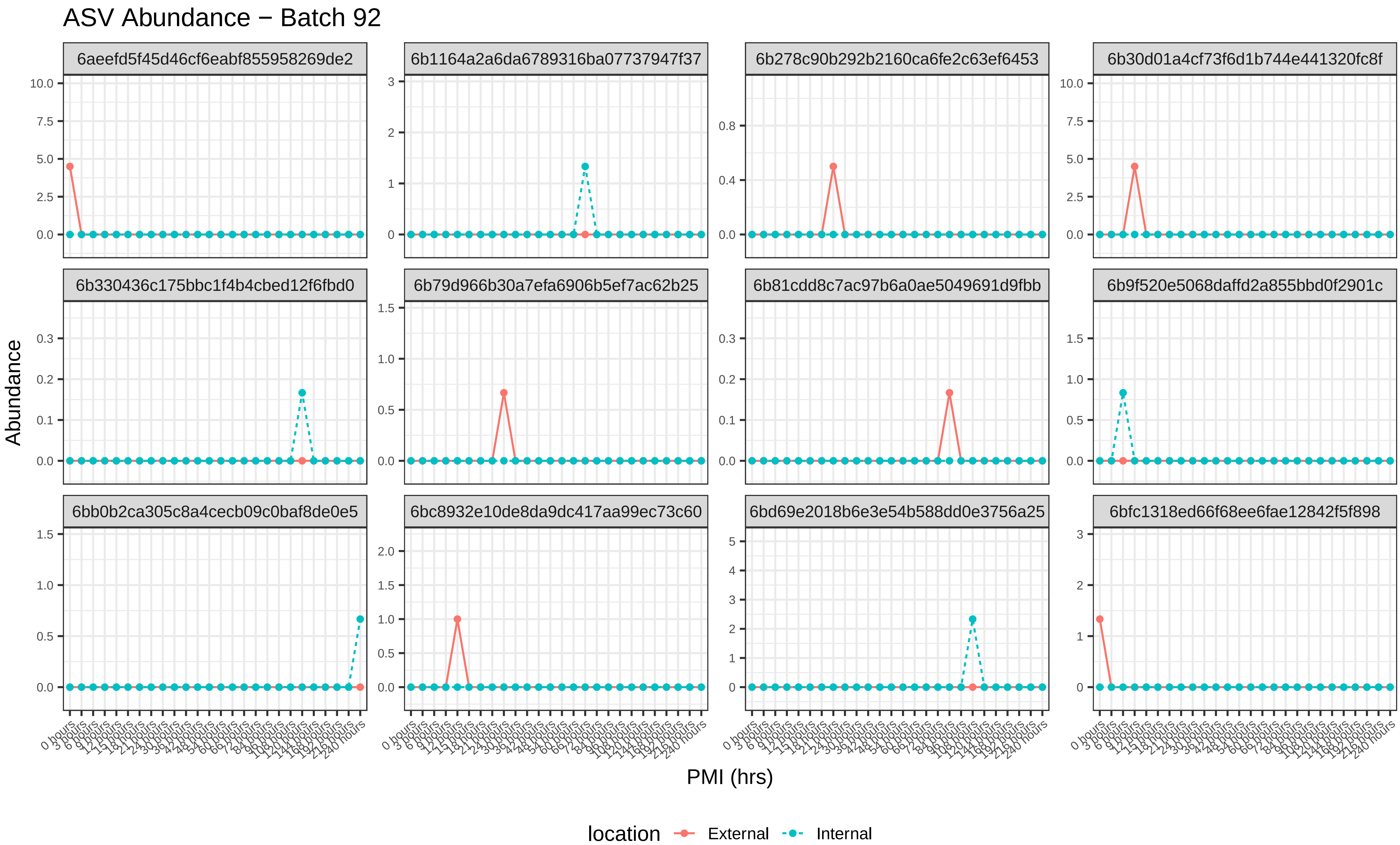

ASV Abundance − Batch 92
6aeefd5f45d46cf6eabf855958269de2
6b1164a2a6da6789316ba07737947f37
6b278c90b292b2160ca6fe2c63ef6453
6b30d01a4cf73f6d1b744e441320fc8f
6b330436c175bbc1f4b4cbed12f6fbd0
6b79d966b30a7efa6906b5ef7ac62b25
6b81cdd8c7ac97b6a0ae5049691d9fbb
6b9f520e5068daffd2a855bbd0f2901c
6bb0b2ca305c8a4cecb09c0baf8de0e5
6bc8932e10de8da9dc417aa99ec73c60
6bd69e2018b6e3e54b588dd0e3756a25
6bfc1318ed66f68ee6fae12842f5f898
Abundance
PMI (hrs)
location
External
Internal

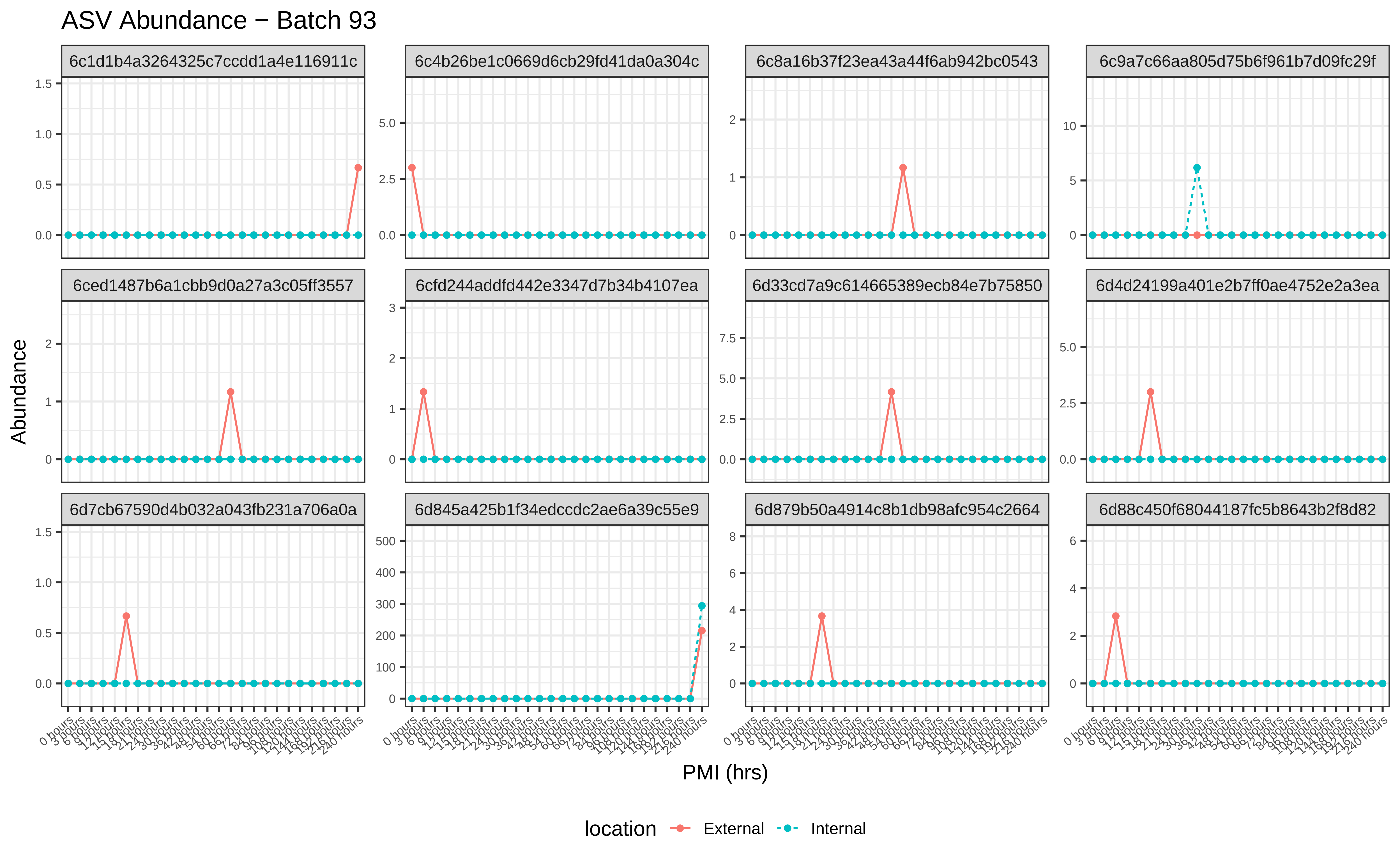

ASV Abundance − Batch 93
6c1d1b4a3264325c7ccdd1a4e116911c
6c4b26be1c0669d6cb29fd41da0a304c
6c8a16b37f23ea43a44f6ab942bc0543
6c9a7c66aa805d75b6f961b7d09fc29f
6ced1487b6a1cbb9d0a27a3c05ff3557
6cfd244addfd442e3347d7b34b4107ea
6d33cd7a9c614665389ecb84e7b75850
6d4d24199a401e2b7ff0ae4752e2a3ea
6d7cb67590d4b032a043fb231a706a0a
6d845a425b1f34edccdc2ae6a39c55e9
6d879b50a4914c8b1db98afc954c2664
6d88c450f68044187fc5b8643b2f8d82
Abundance
PMI (hrs)
location
External
Internal

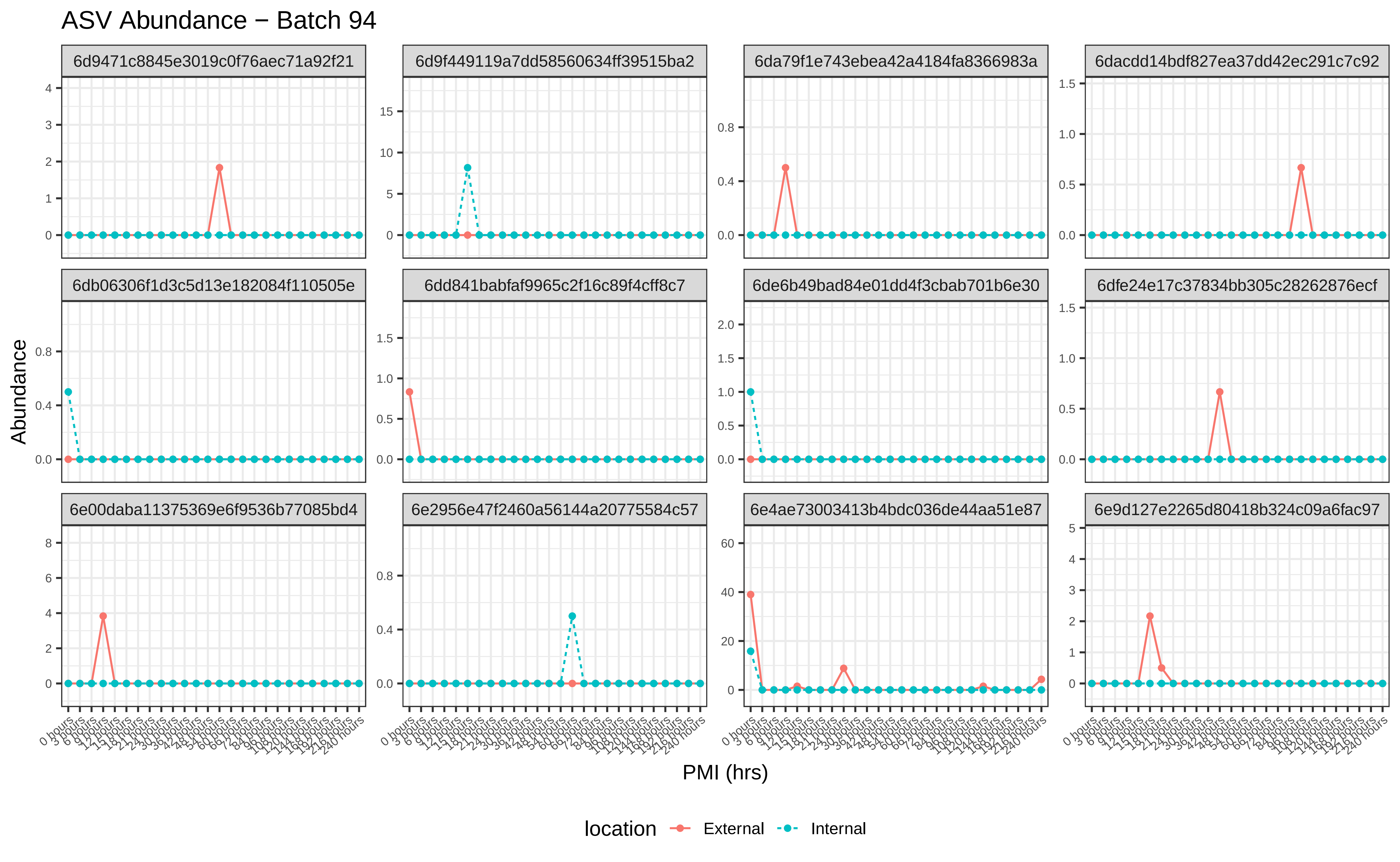
ASV Abundance − Batch 94
6d9471c8845e3019c0f76aec71a92f21
6d9f449119a7dd58560634ff39515ba2
6da79f1e743ebea42a4184fa8366983a
6dacdd14bdf827ea37dd42ec291c7c92
6db06306f1d3c5d13e182084f110505e
6dd841babfaf9965c2f16c89f4cff8c7
6de6b49bad84e01dd4f3cbab701b6e30
6dfe24e17c37834bb305c28262876ecf
6e00daba11375369e6f9536b77085bd4
6e2956e47f2460a56144a20775584c57
6e4ae73003413b4bdc036de44aa51e87
6e9d127e2265d80418b324c09a6fac97
Abundance
PMI (hrs)
location
External
Internal

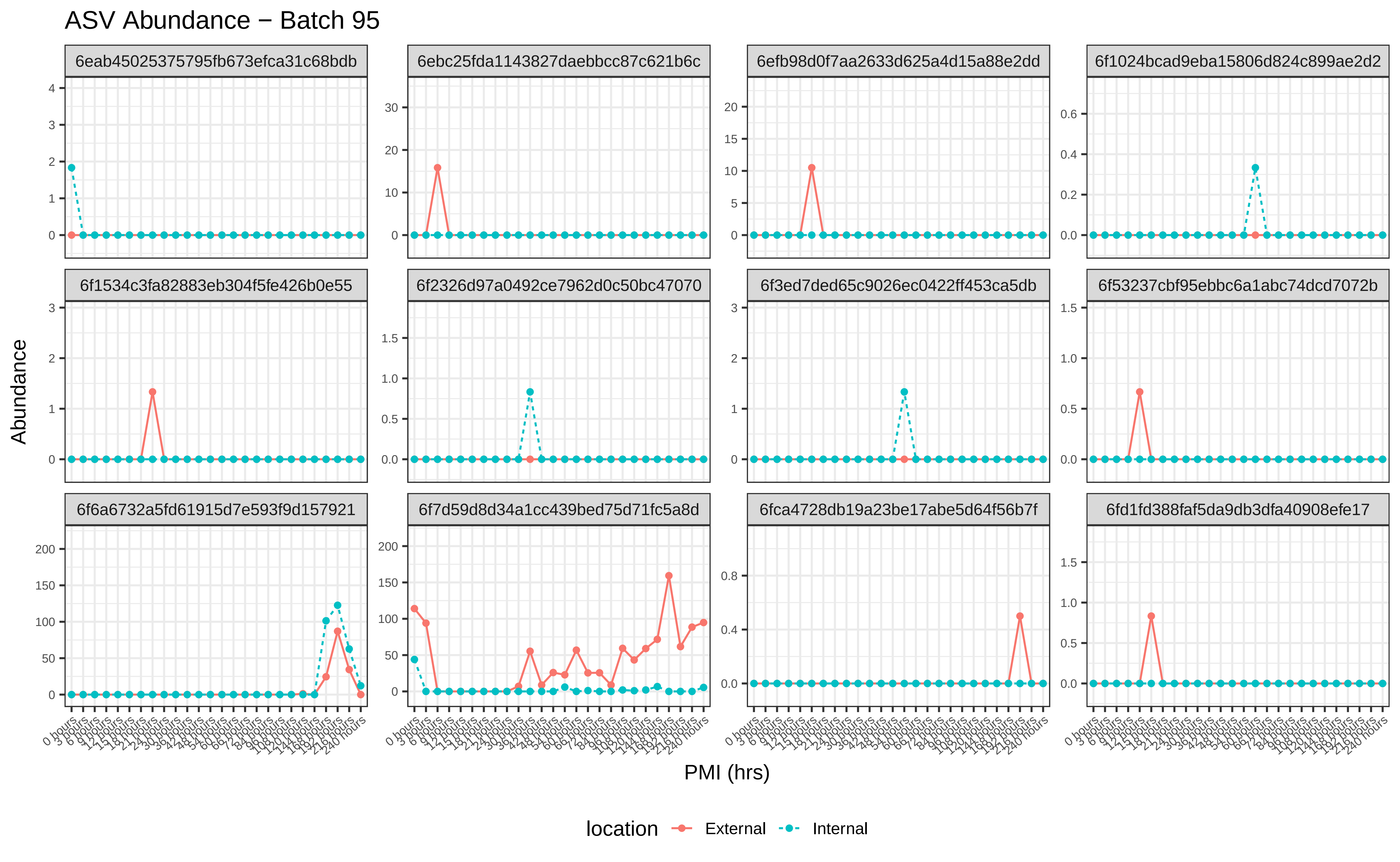
ASV Abundance − Batch 95
6eab45025375795fb673efca31c68bdb
6ebc25fda1143827daebbcc87c621b6c
6efb98d0f7aa2633d625a4d15a88e2dd
6f1024bcad9eba15806d824c899ae2d2
6f1534c3fa82883eb304f5fe426b0e55
6f2326d97a0492ce7962d0c50bc47070
6f3ed7ded65c9026ec0422ff453ca5db
6f53237cbf95ebbc6a1abc74dcd7072b
6f6a6732a5fd61915d7e593f9d157921
6f7d59d8d34a1cc439bed75d71fc5a8d
6fca4728db19a23be17abe5d64f56b7f
6fd1fd388faf5da9db3dfa40908efe17
Abundance
PMI (hrs)
location
External
Internal

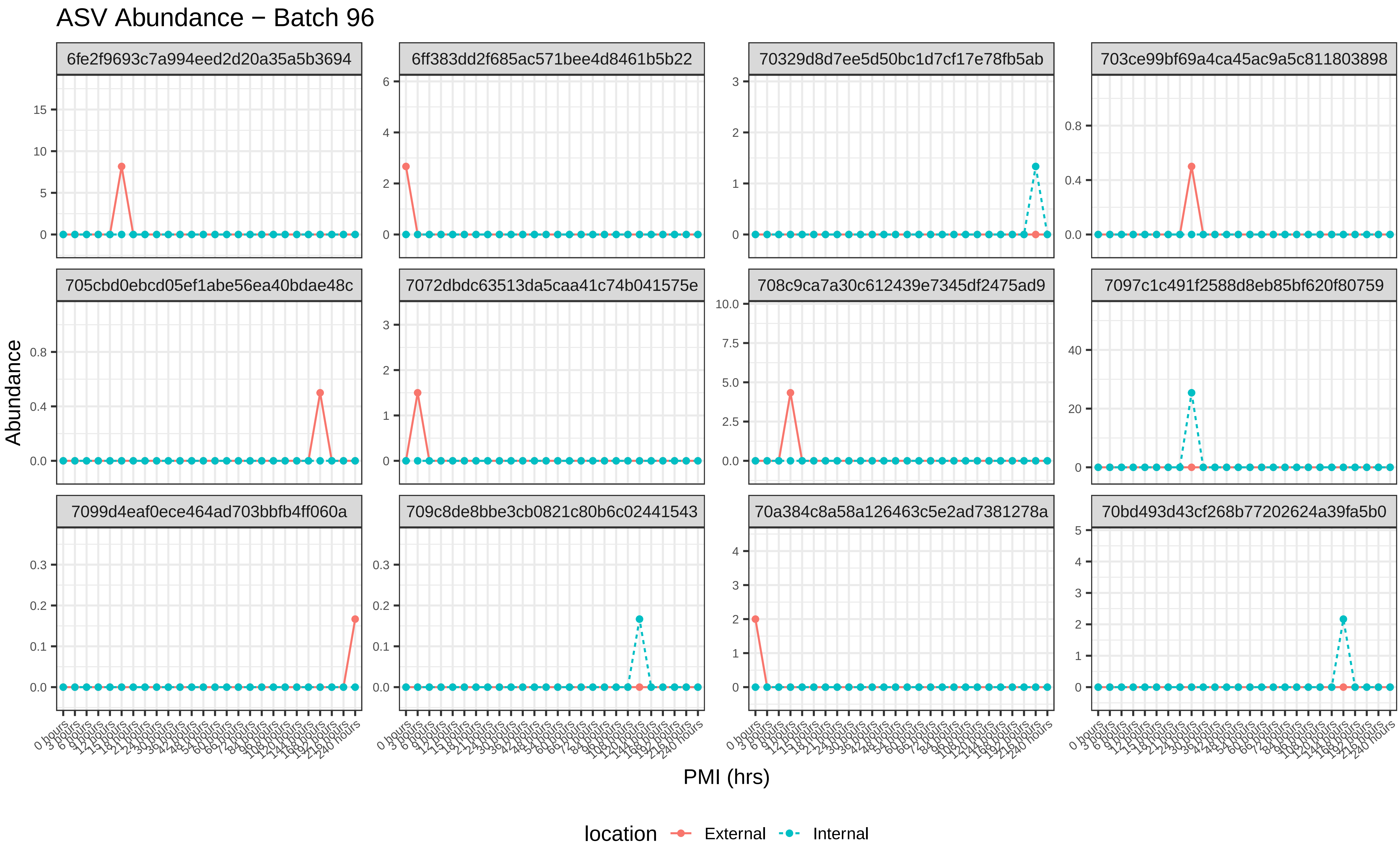

ASV Abundance − Batch 96
6fe2f9693c7a994eed2d20a35a5b3694
6ff383dd2f685ac571bee4d8461b5b22
70329d8d7ee5d50bc1d7cf17e78fb5ab
703ce99bf69a4ca45ac9a5c811803898
705cbd0ebcd05ef1abe56ea40bdae48c
7072dbdc63513da5caa41c74b041575e
708c9ca7a30c612439e7345df2475ad9
7097c1c491f2588d8eb85bf620f80759
7099d4eaf0ece464ad703bbfb4ff060a
709c8de8bbe3cb0821c80b6c02441543
70a384c8a58a126463c5e2ad7381278a
70bd493d43cf268b77202624a39fa5b0
Abundance
PMI (hrs)
0 hours
3 hours
6 hours
9 hours
12 hours
15 hours
18 hours
21 hours
24 hours
30 hours
36 hours
42 hours
48 hours
54 hours
60 hours
66 hours
72 hours
84 hours
96 hours
108 hours
120 hours
144 hours
168 hours
192 hours
216 hours
240 hours
location
External
Internal

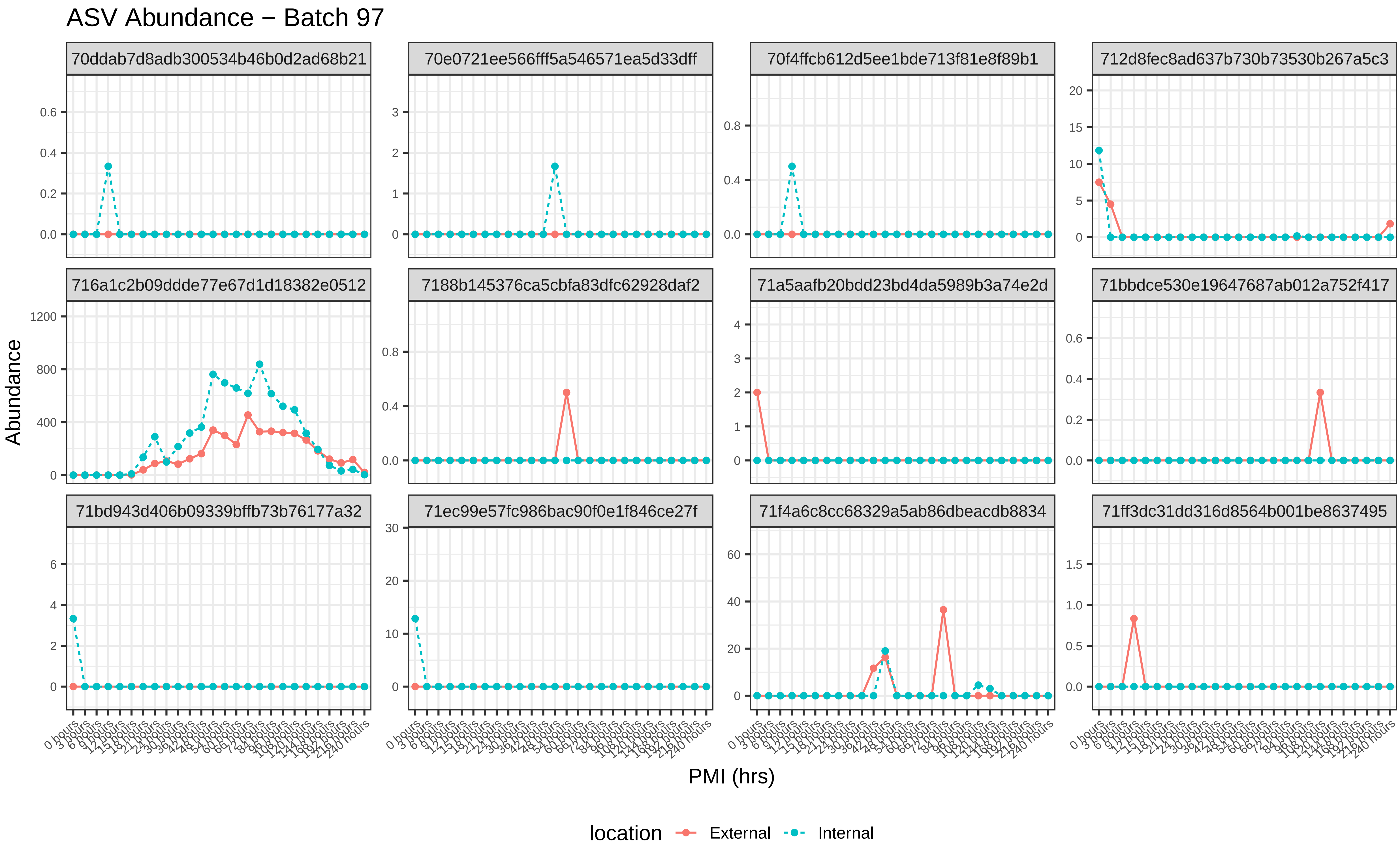

ASV Abundance − Batch 97
70ddab7d8adb300534b46b0d2ad68b21
70e0721ee566fff5a546571ea5d33dff
70f4ffcb612d5ee1bde713f81e8f89b1
712d8fec8ad637b730b73530b267a5c3
716a1c2b09ddde77e67d1d18382e0512
7188b145376ca5cbfa83dfc62928daf2
71a5aafb20bdd23bd4da5989b3a74e2d
71bbdce530e19647687ab012a752f417
71bd943d406b09339bffb73b76177a32
71ec99e57fc986bac90f0e1f846ce27f
71f4a6c8cc68329a5ab86dbeacdb8834
71ff3dc31dd316d8564b001be8637495
Abundance
PMI (hrs)
0 hours
3 hours
6 hours
9 hours
12 hours
15 hours
18 hours
21 hours
24 hours
30 hours
36 hours
42 hours
48 hours
54 hours
60 hours
66 hours
72 hours
84 hours
96 hours
108 hours
120 hours
144 hours
168 hours
192 hours
216 hours
240 hours
location
External
Internal

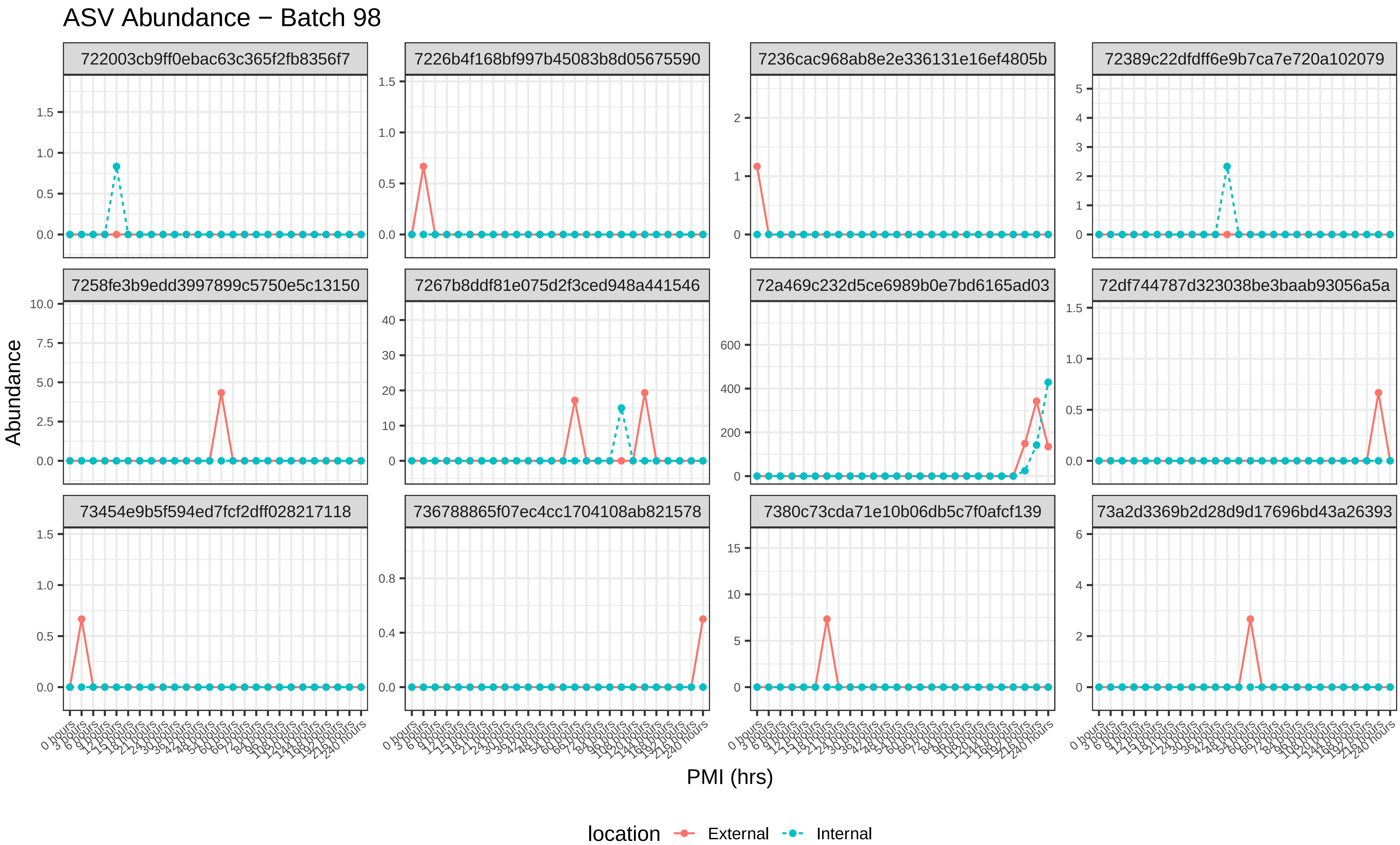

ASV Abundance − Batch 98
722003cb9ff0ebac63c365f2fb8356f7
7226b4f168bf997b45083b8d05675590
7236cac968ab8e2e336131e16ef4805b
72389c22dfdff6e9b7ca7e720a102079
7258fe3b9edd3997899c5750e5c13150
7267b8ddf81e075d2f3ced948a441546
72a469c232d5ce6989b0e7bd6165ad03
72df744787d323038be3baab93056a5a
73454e9b5f594ed7fcf2dff028217118
736788865f07ec4cc1704108ab821578
7380c73cda71e10b06db5c7f0afcf139
73a2d3369b2d28d9d17696bd43a26393
Abundance
PMI (hrs)
location
External
Internal

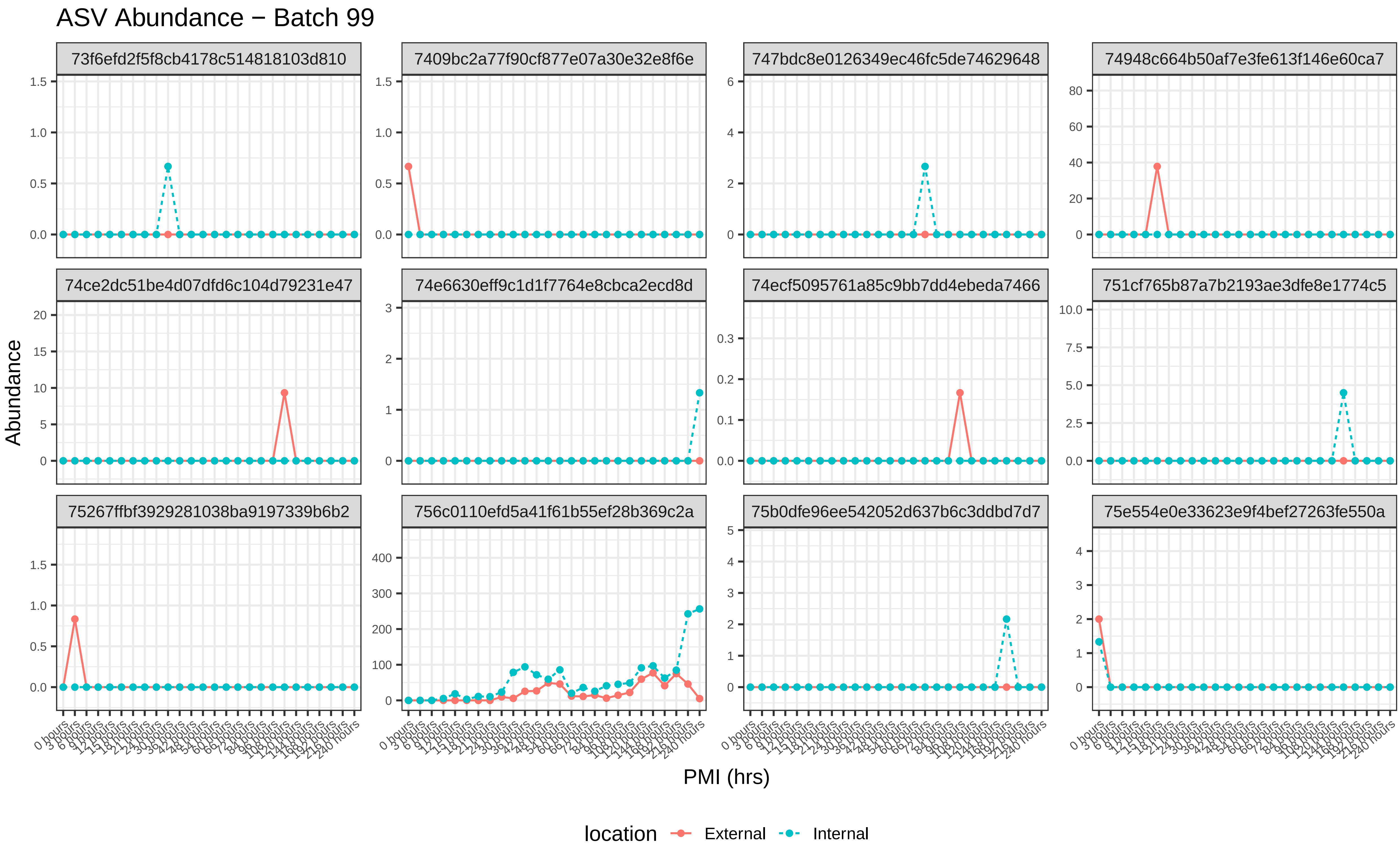

ASV Abundance − Batch 99
73f6efd2f5f8cb4178c514818103d810
7409bc2a77f90cf877e07a30e32e8f6e
747bdc8e0126349ec46fc5de74629648
74948c664b50af7e3fe613f146e60ca7
74ce2dc51be4d07dfd6c104d79231e47
74e6630eff9c1d1f7764e8cbca2ecd8d
74ecf5095761a85c9bb7dd4ebeda7466
751cf765b87a7b2193ae3dfe8e1774c5
75267ffbf3929281038ba9197339b6b2
756c0110efd5a41f61b55ef28b369c2a
75b0dfe96ee542052d637b6c3ddbd7d7
75e554e0e33623e9f4bef27263fe550a
Abundance
PMI (hrs)
location
External
Internal

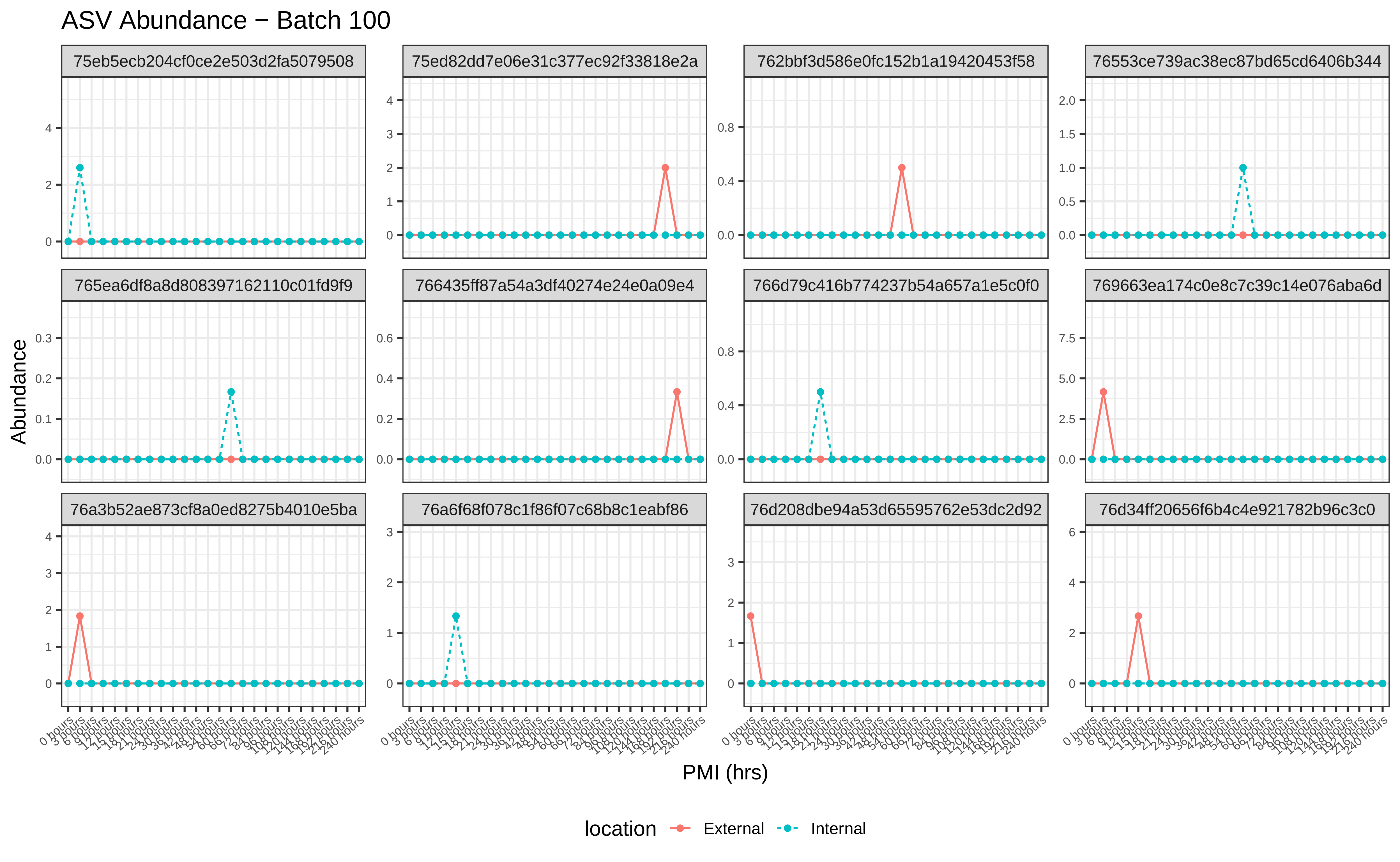

ASV Abundance − Batch 100
75eb5ecb204cf0ce2e503d2fa5079508
75ed82dd7e06e31c377ec92f33818e2a
762bbf3d586e0fc152b1a19420453f58
76553ce739ac38ec87bd65cd6406b344
765ea6df8a8d808397162110c01fd9f9
766435ff87a54a3df40274e24e0a09e4
766d79c416b774237b54a657a1e5c0f0
769663ea174c0e8c7c39c14e076aba6d
76a3b52ae873cf8a0ed8275b4010e5ba
76a6f68f078c1f86f07c68b8c1eabf86
76d208dbe94a53d65595762e53dc2d92
76d34ff20656f6b4c4e921782b96c3c0
Abundance
PMI (hrs)
location
External
Internal

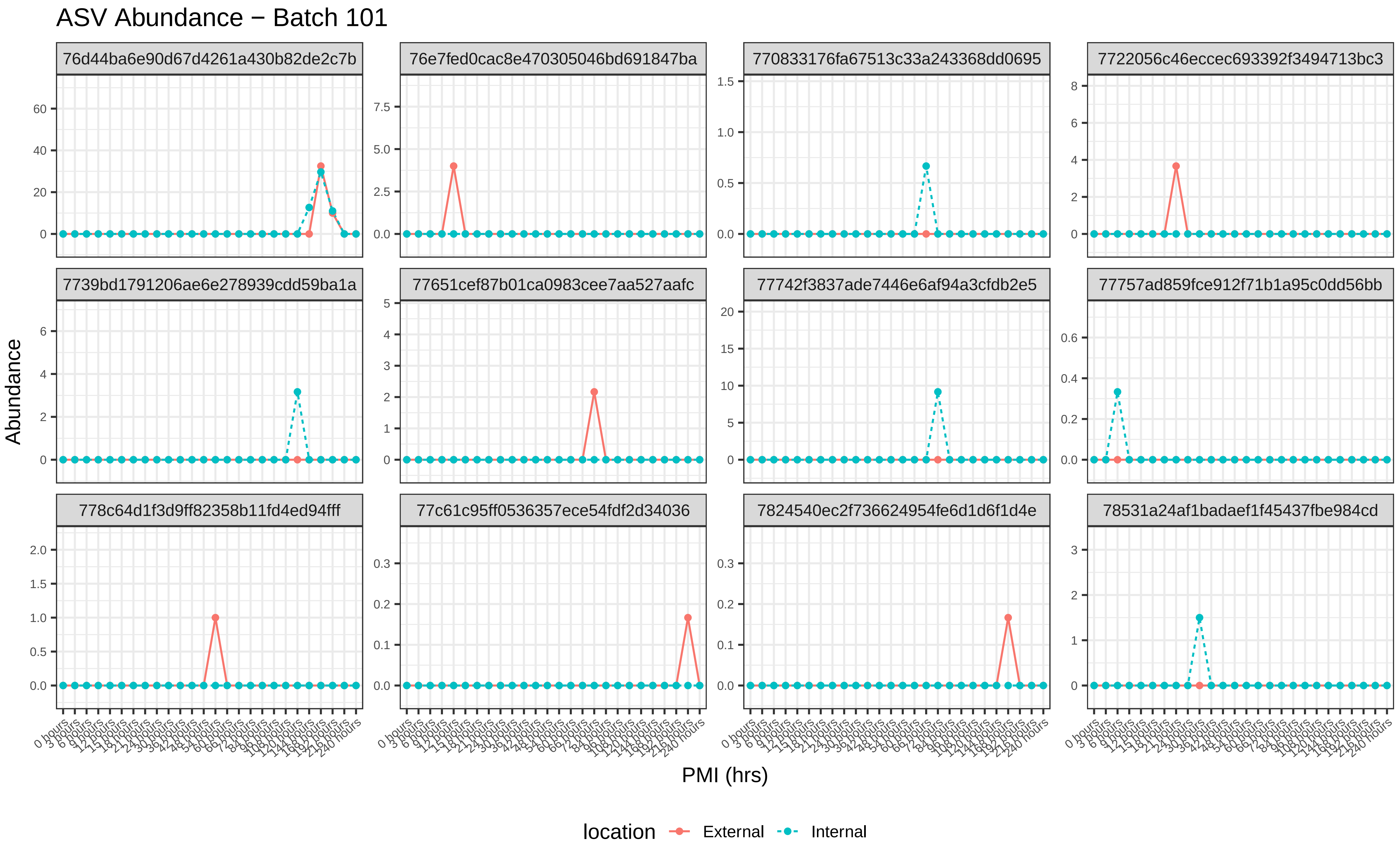
ASV Abundance − Batch 101
76d44ba6e90d67d4261a430b82de2c7b
76e7fed0cac8e470305046bd691847ba
770833176fa67513c33a243368dd0695
7722056c46eccec693392f3494713bc3
7739bd1791206ae6e278939cdd59ba1a
77651cef87b01ca0983cee7aa527aafc
77742f3837ade7446e6af94a3cfdb2e5
77757ad859fce912f71b1a95c0dd56bb
778c64d1f3d9ff82358b11fd4ed94fff
77c61c95ff0536357ece54fdf2d34036
7824540ec2f736624954fe6d1d6f1d4e
78531a24af1badaef1f45437fbe984cd
Abundance
PMI (hrs)
location
External
Internal

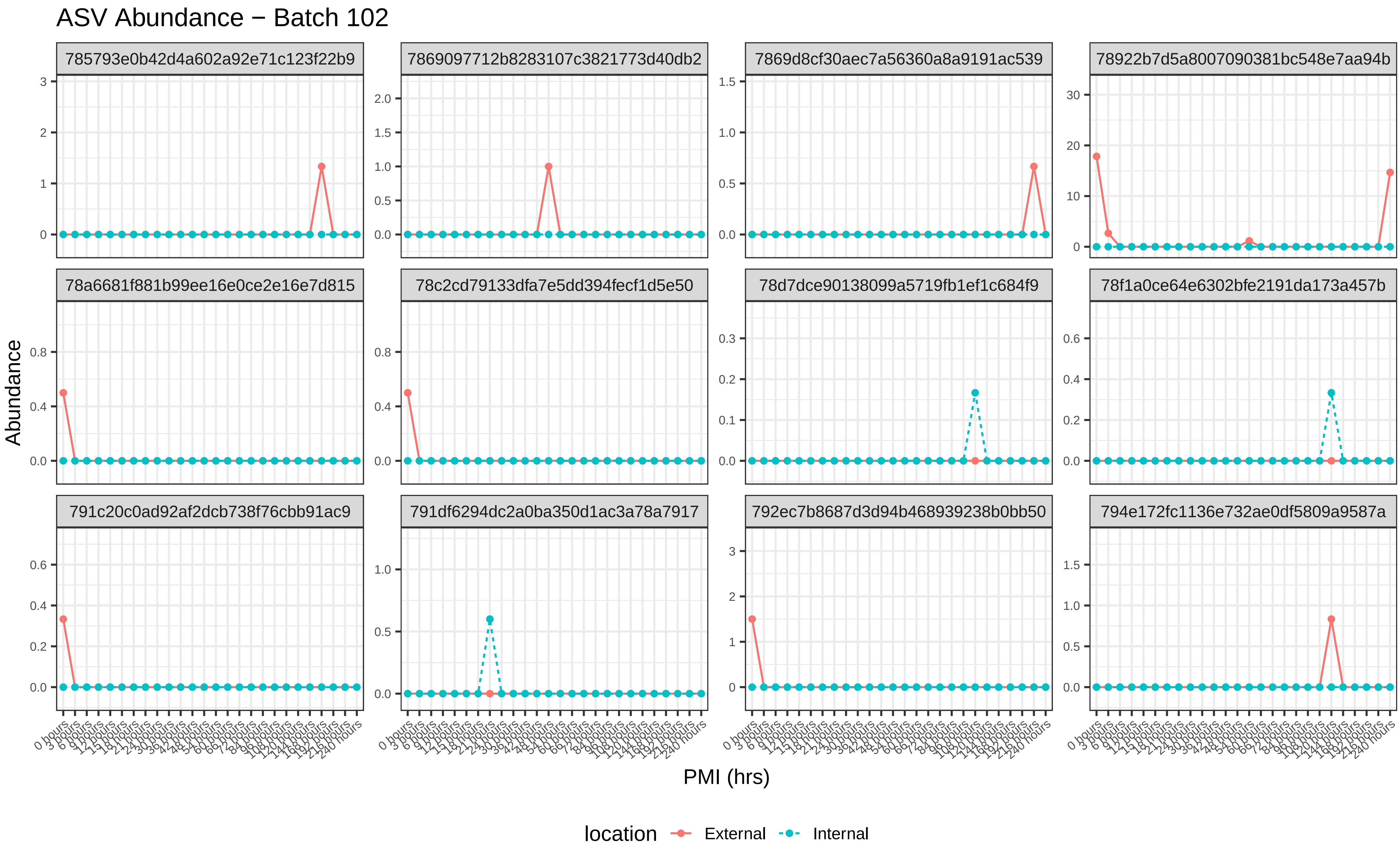

ASV Abundance − Batch 102
785793e0b42d4a602a92e71c123f22b9
7869097712b8283107c3821773d40db2
7869d8cf30aec7a56360a8a9191ac539
78922b7d5a8007090381bc548e7aa94b
78a6681f881b99ee16e0ce2e16e7d815
78c2cd79133dfa7e5dd394fecf1d5e50
78d7dce90138099a5719fb1ef1c684f9
78f1a0ce64e6302bfe2191da173a457b
791c20c0ad92af2dcb738f76cbb91ac9
791df6294dc2a0ba350d1ac3a78a7917
792ec7b8687d3d94b468939238b0bb50
794e172fc1136e732ae0df5809a9587a
Abundance
PMI (hrs)
location
External
Internal

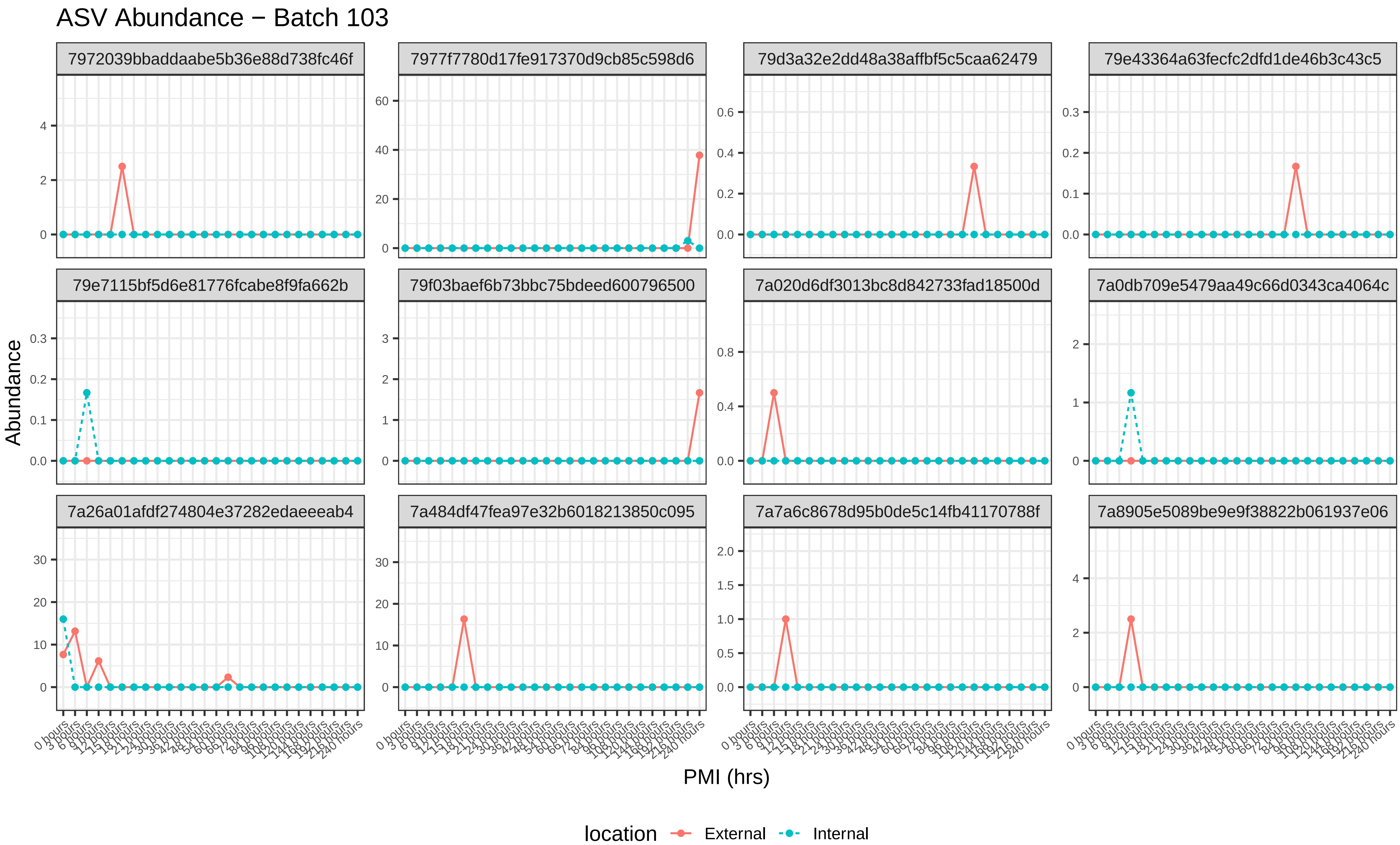
ASV Abundance − Batch 103
7972039bbaddaabe5b36e88d738fc46f
7977f7780d17fe917370d9cb85c598d6
79d3a32e2dd48a38affbf5c5caa62479
79e43364a63fecfc2dfd1de46b3c43c5
79e7115bf5d6e81776fcabe8f9fa662b
79f03baef6b73bbc75bdeed600796500
7a020d6df3013bc8d842733fad18500d
7a0db709e5479aa49c66d0343ca4064c
7a26a01afdf274804e37282edaeeeab4
7a484df47fea97e32b6018213850c095
7a7a6c8678d95b0de5c14fb41170788f
7a8905e5089be9e9f38822b061937e06
Abundance
PMI (hrs)
location
External
Internal

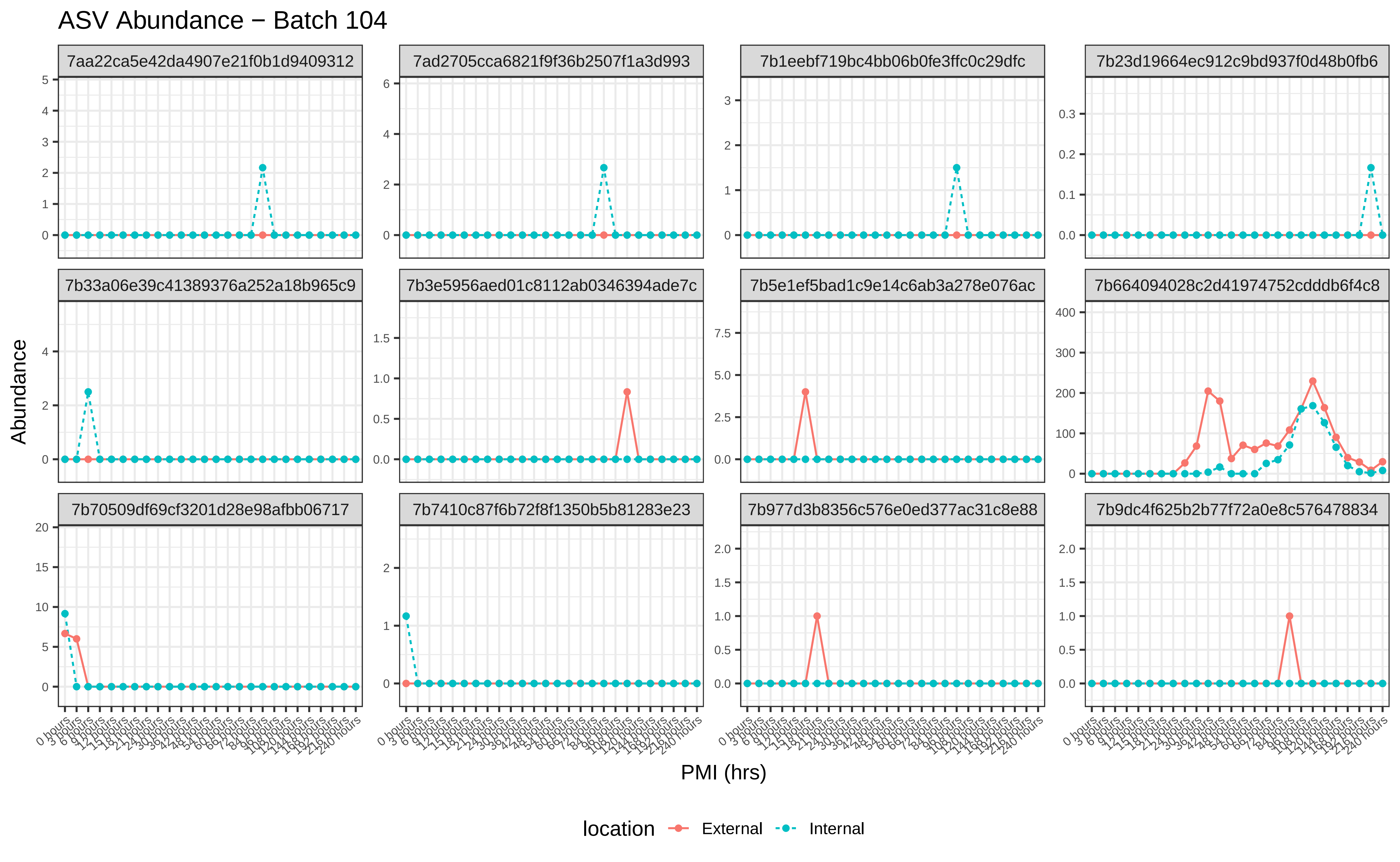
ASV Abundance − Batch 104
7aa22ca5e42da4907e21f0b1d9409312
7ad2705cca6821f9f36b2507f1a3d993
7b1eebf719bc4bb06b0fe3ffc0c29dfc
7b23d19664ec912c9bd937f0d48b0fb6
7b33a06e39c41389376a252a18b965c9
7b3e5956aed01c8112ab0346394ade7c
7b5e1ef5bad1c9e14c6ab3a278e076ac
7b664094028c2d41974752cdddb6f4c8
7b70509df69cf3201d28e98afbb06717
7b7410c87f6b72f8f1350b5b81283e23
7b977d3b8356c576e0ed377ac31c8e88
7b9dc4f625b2b77f72a0e8c576478834
Abundance
PMI (hrs)
location
External
Internal

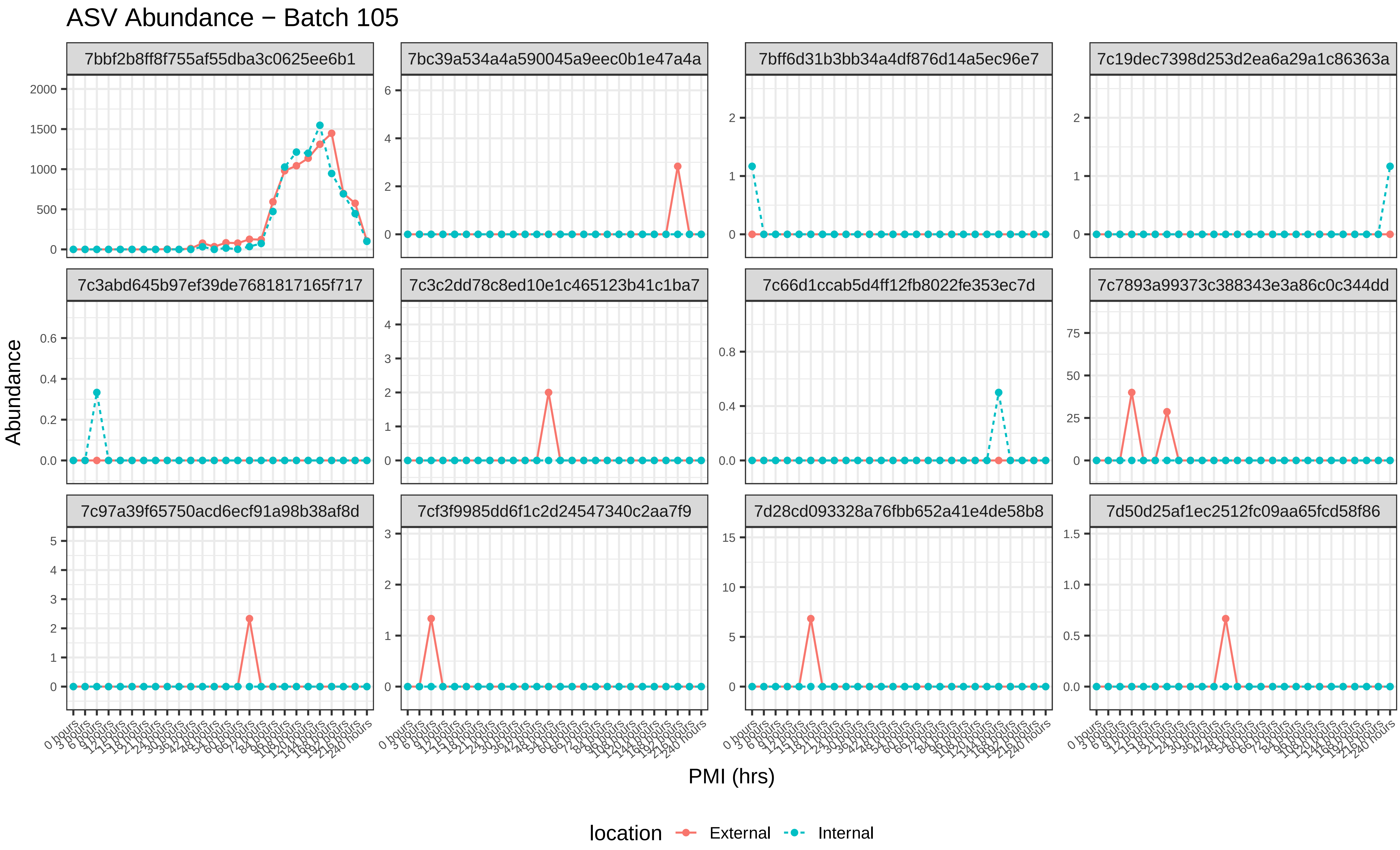
ASV Abundance − Batch 105
7bbf2b8ff8f755af55dba3c0625ee6b1
7bc39a534a4a590045a9eec0b1e47a4a
7bff6d31b3bb34a4df876d14a5ec96e7
7c19dec7398d253d2ea6a29a1c86363a
7c3abd645b97ef39de7681817165f717
7c3c2dd78c8ed10e1c465123b41c1ba7
7c66d1ccab5d4ff12fb8022fe353ec7d
7c7893a99373c388343e3a86c0c344dd
7c97a39f65750acd6ecf91a98b38af8d
7cf3f9985dd6f1c2d24547340c2aa7f9
7d28cd093328a76fbb652a41e4de58b8
7d50d25af1ec2512fc09aa65fcd58f86
Abundance
PMI (hrs)
location
External
Internal

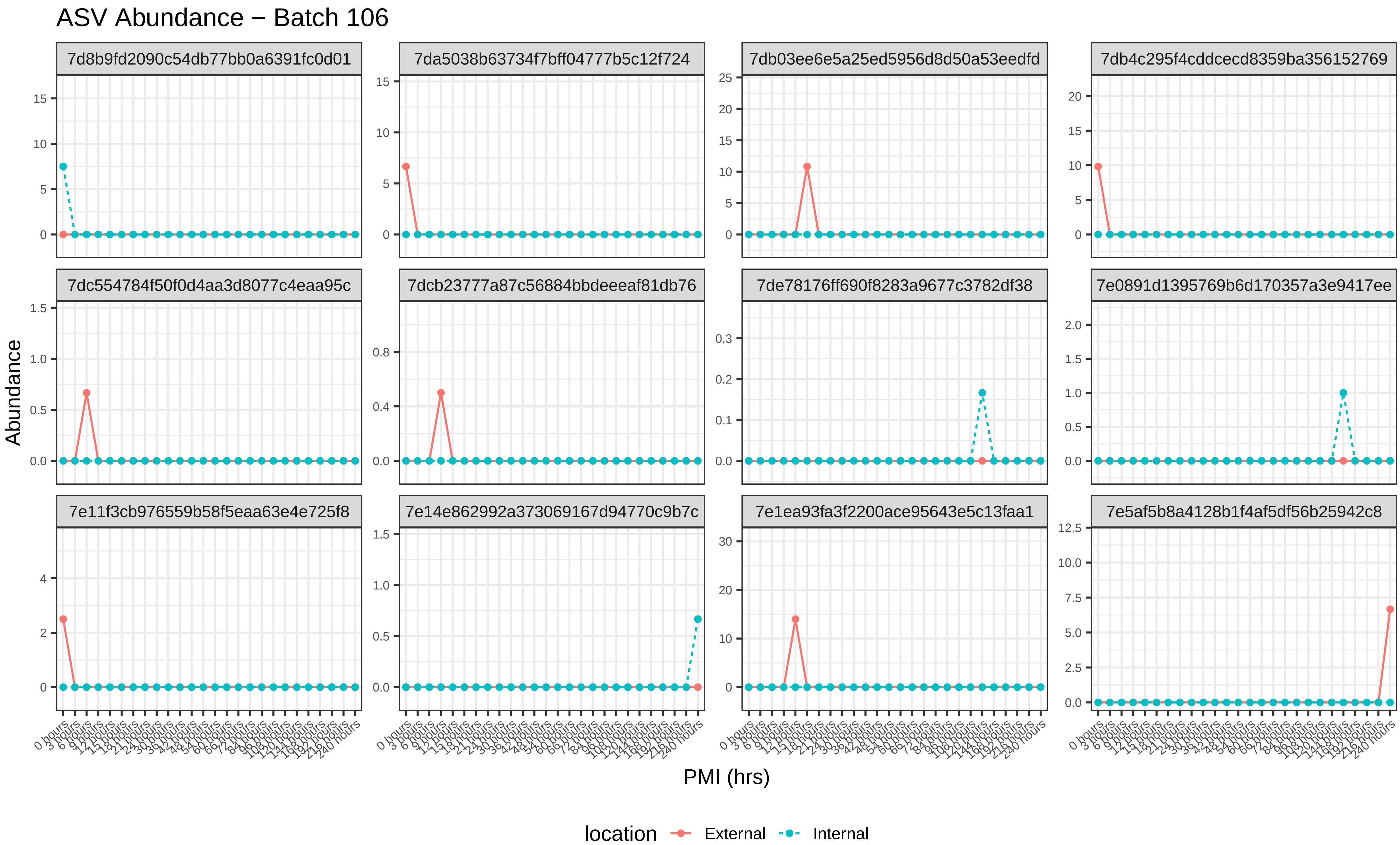
ASV Abundance − Batch 106
7d8b9fd2090c54db77bb0a6391fc0d01
7da5038b63734f7bff04777b5c12f724
7db03ee6e5a25ed5956d8d50a53eedfd
7db4c295f4cddcecd8359ba356152769
7dc554784f50f0d4aa3d8077c4eaa95c
7dcb23777a87c56884bbdeeeaf81db76
7de78176ff690f8283a9677c3782df38
7e0891d1395769b6d170357a3e9417ee
7e11f3cb976559b58f5eaa63e4e725f8
7e14e862992a373069167d94770c9b7c
7e1ea93fa3f2200ace95643e5c13faa1
7e5af5b8a4128b1f4af5df56b25942c8
Abundance
PMI (hrs)
location
External
Internal

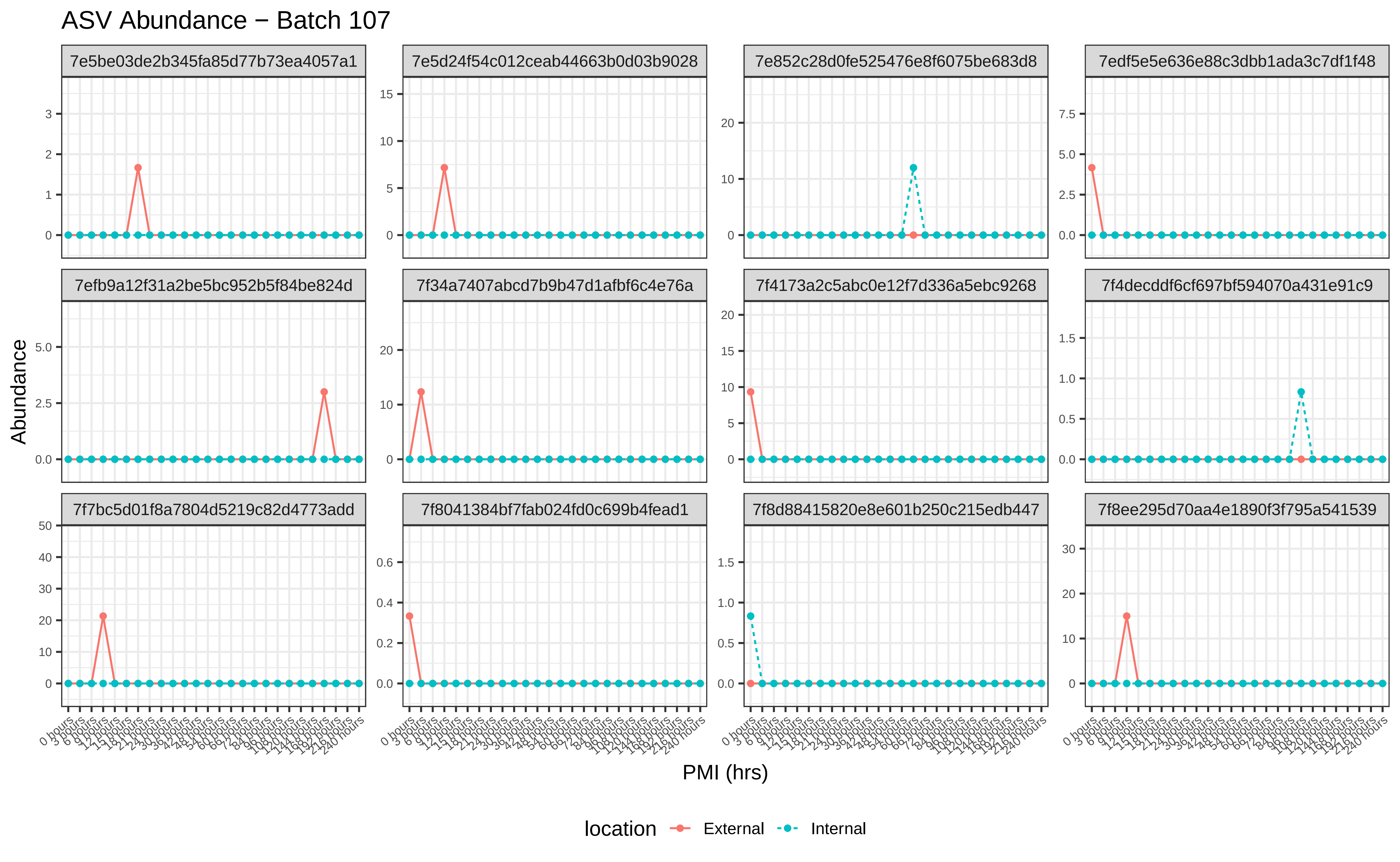

ASV Abundance − Batch 107
7e5be03de2b345fa85d77b73ea4057a1
7e5d24f54c012ceab44663b0d03b9028
7e852c28d0fe525476e8f6075be683d8
7edf5e5e636e88c3dbb1ada3c7df1f48
7efb9a12f31a2be5bc952b5f84be824d
7f34a7407abcd7b9b47d1afbf6c4e76a
7f4173a2c5abc0e12f7d336a5ebc9268
7f4decddf6cf697bf594070a431e91c9
7f7bc5d01f8a7804d5219c82d4773add
7f8041384bf7fab024fd0c699b4fead1
7f8d88415820e8e601b250c215edb447
7f8ee295d70aa4e1890f3f795a541539
Abundance
PMI (hrs)
location
External
Internal

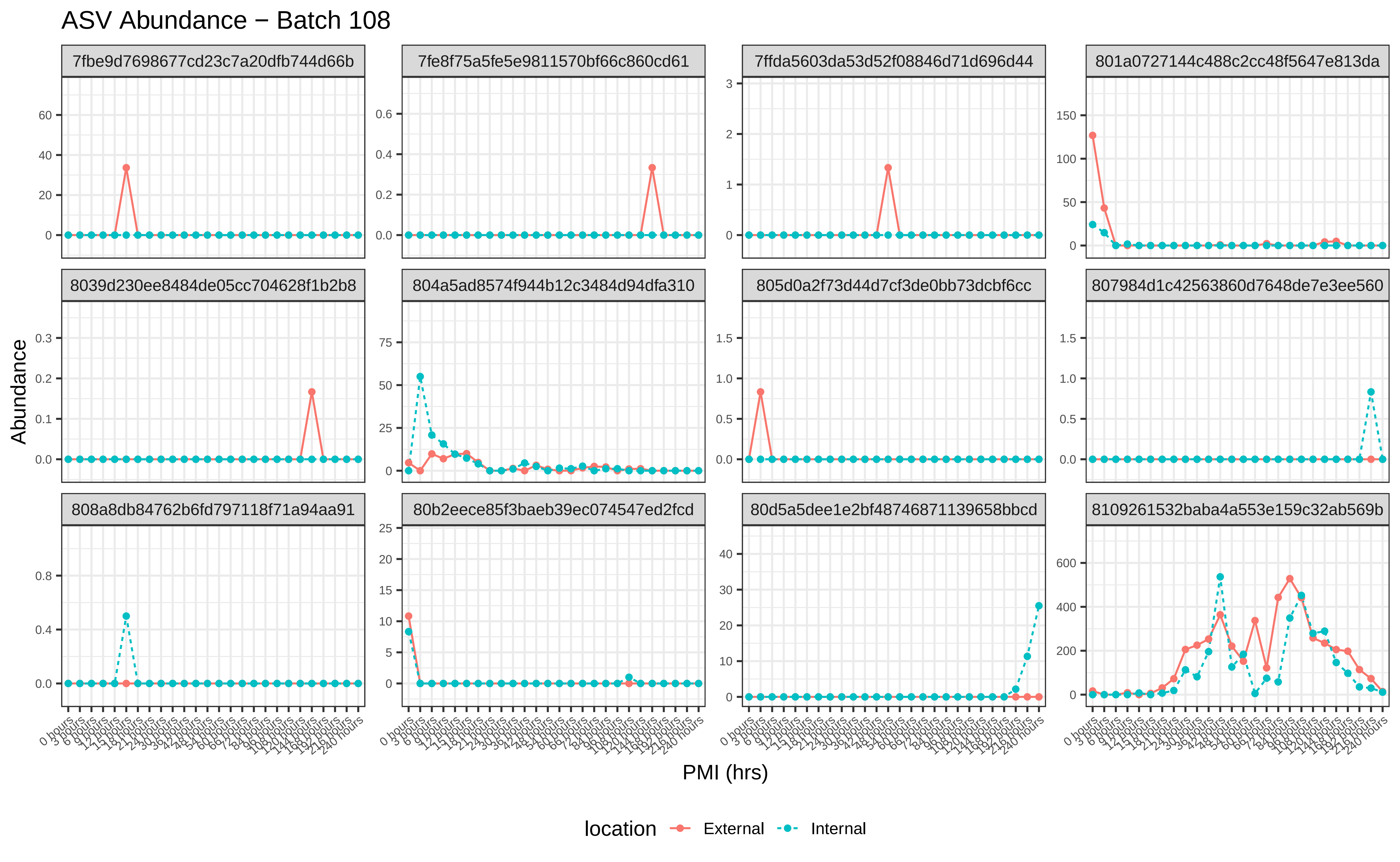

ASV Abundance − Batch 108
7fbe9d7698677cd23c7a20dfb744d66b
7fe8f75a5fe5e9811570bf66c860cd61
7ffda5603da53d52f08846d71d696d44
801a0727144c488c2cc48f5647e813da
8039d230ee8484de05cc704628f1b2b8
804a5ad8574f944b12c3484d94dfa310
805d0a2f73d44d7cf3de0bb73dcbf6cc
807984d1c42563860d7648de7e3ee560
808a8db84762b6fd797118f71a94aa91
80b2eece85f3baeb39ec074547ed2fcd
80d5a5dee1e2bf48746871139658bbcd
8109261532baba4a553e159c32ab569b
Abundance
PMI (hrs)
location
External
Internal

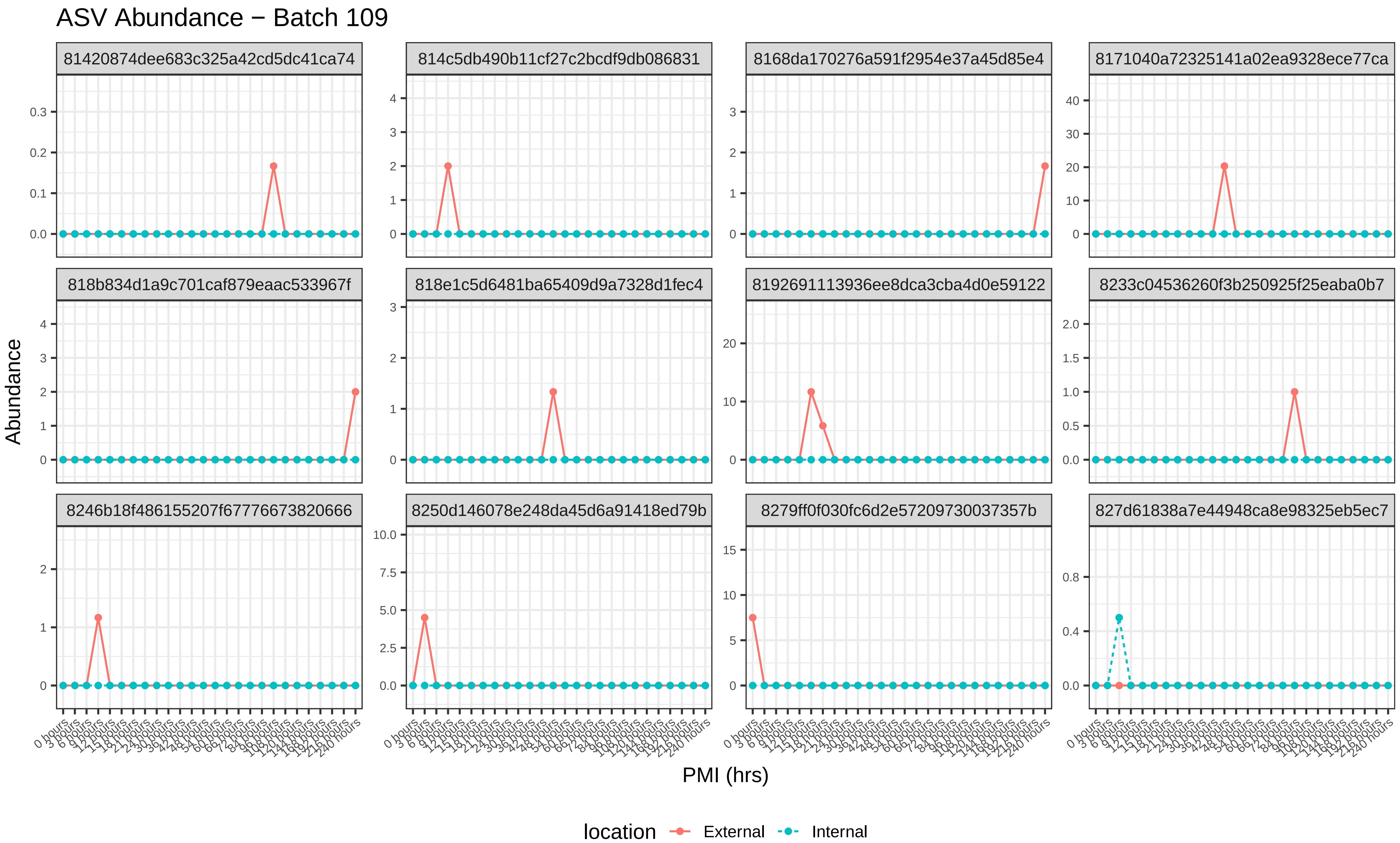
ASV Abundance − Batch 109
81420874dee683c325a42cd5dc41ca74
814c5db490b11cf27c2bcdf9db086831
8168da170276a591f2954e37a45d85e4
8171040a72325141a02ea9328ece77ca
818b834d1a9c701caf879eaac533967f
818e1c5d6481ba65409d9a7328d1fec4
8192691113936ee8dca3cba4d0e59122
8233c04536260f3b250925f25eaba0b7
8246b18f486155207f67776673820666
8250d146078e248da45d6a91418ed79b
8279ff0f030fc6d2e57209730037357b
827d61838a7e44948ca8e98325eb5ec7
Abundance
PMI (hrs)
location
External
Internal

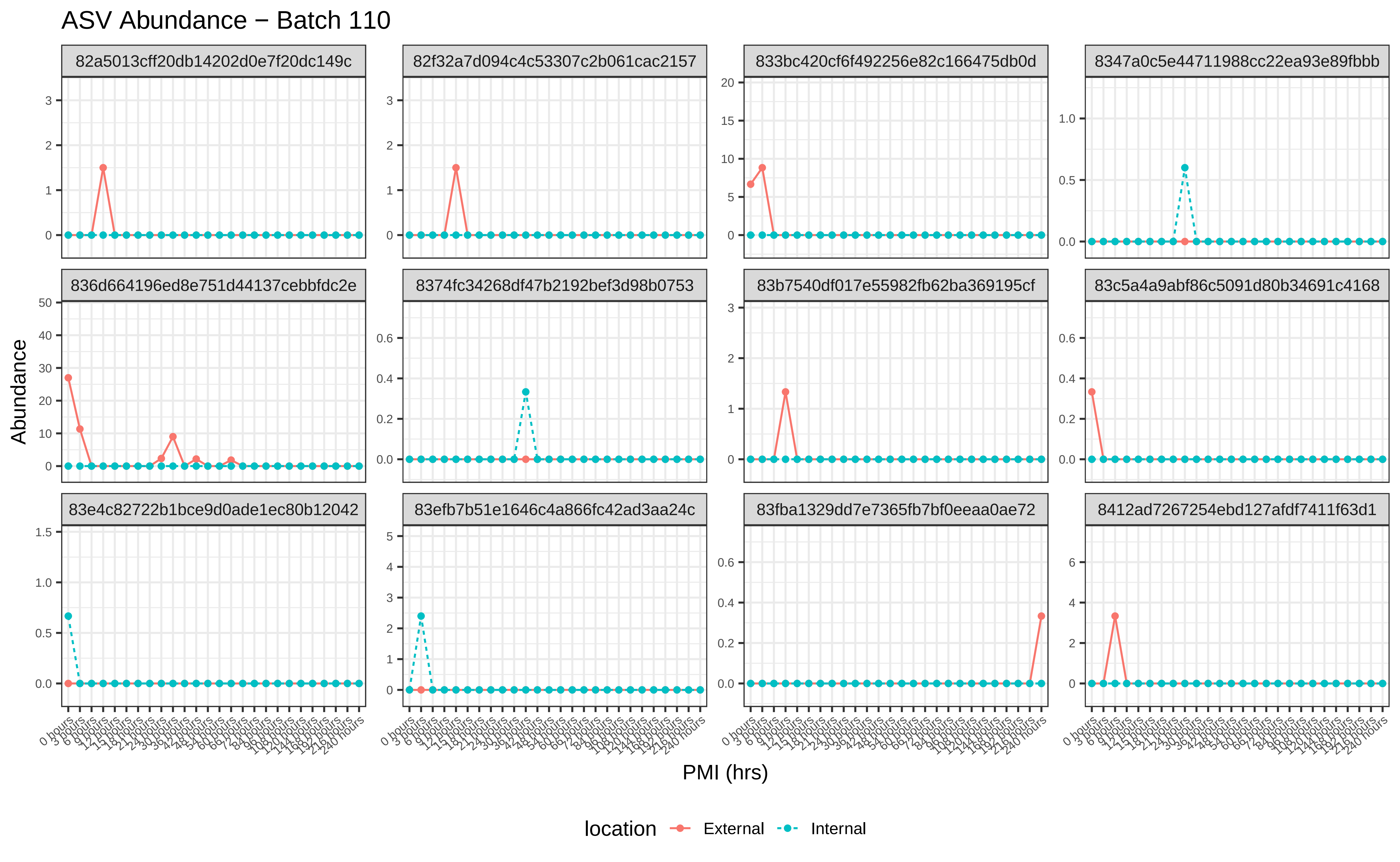
ASV Abundance − Batch 110
82a5013cff20db14202d0e7f20dc149c
82f32a7d094c4c53307c2b061cac2157
833bc420cf6f492256e82c166475db0d
8347a0c5e44711988cc22ea93e89fbbb
836d664196ed8e751d44137cebbfdc2e
8374fc34268df47b2192bef3d98b0753
83b7540df017e55982fb62ba369195cf
83c5a4a9abf86c5091d80b34691c4168
83e4c82722b1bce9d0ade1ec80b12042
83efb7b51e1646c4a866fc42ad3aa24c
83fba1329dd7e7365fb7bf0eeaa0ae72
8412ad7267254ebd127afdf7411f63d1
Abundance
PMI (hrs)
location
External
Internal

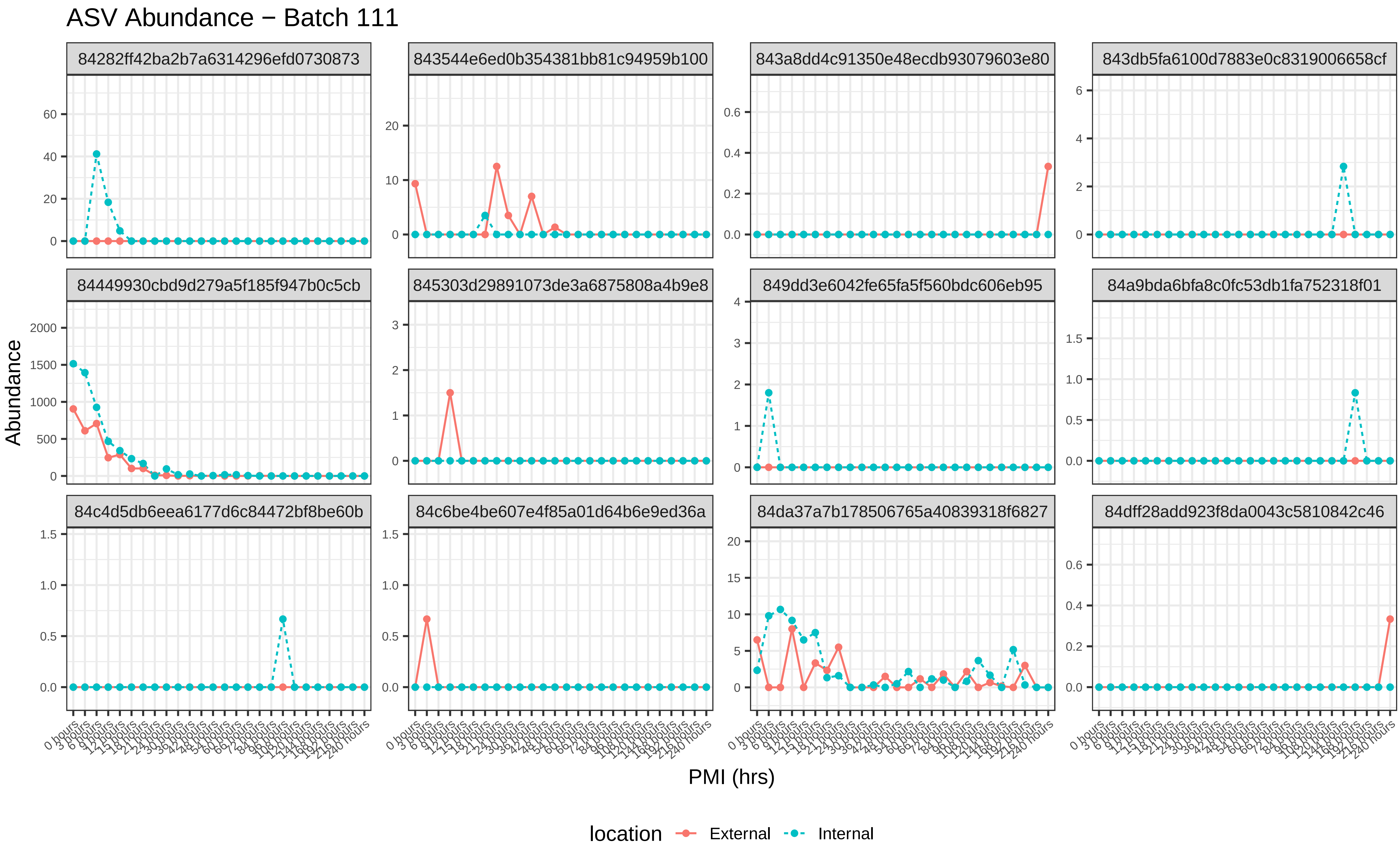
ASV Abundance − Batch 111
84282ff42ba2b7a6314296efd0730873
843544e6ed0b354381bb81c94959b100
843a8dd4c91350e48ecdb93079603e80
843db5fa6100d7883e0c8319006658cf
84449930cbd9d279a5f185f947b0c5cb
845303d29891073de3a6875808a4b9e8
849dd3e6042fe65fa5f560bdc606eb95
84a9bda6bfa8c0fc53db1fa752318f01
84c4d5db6eea6177d6c84472bf8be60b
84c6be4be607e4f85a01d64b6e9ed36a
84da37a7b178506765a40839318f6827
84dff28add923f8da0043c5810842c46
Abundance
PMI (hrs)
location
External
Internal

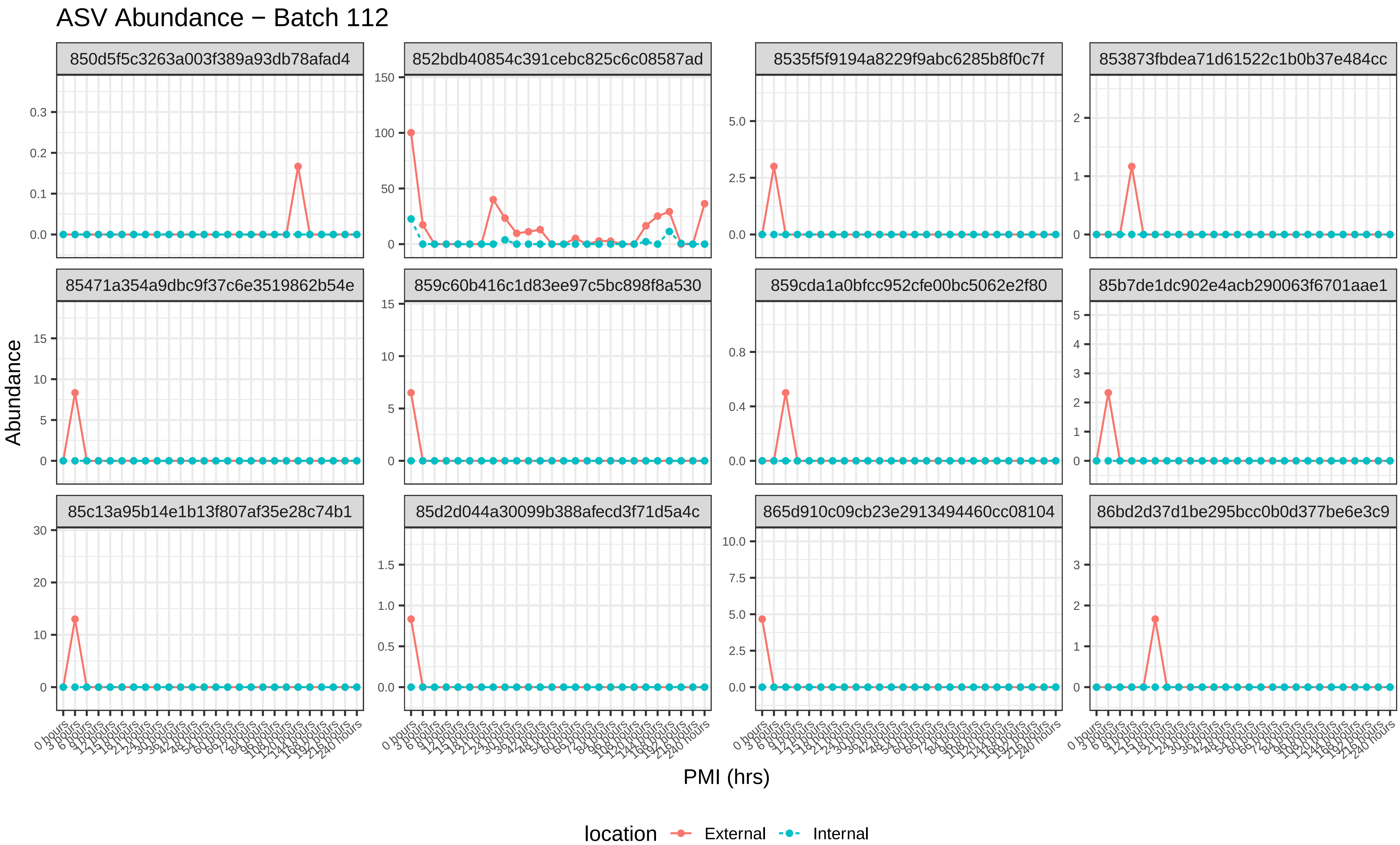
ASV Abundance − Batch 112
850d5f5c3263a003f389a93db78afad4
852bdb40854c391cebc825c6c08587ad
8535f5f9194a8229f9abc6285b8f0c7f
853873fbdea71d61522c1b0b37e484cc
85471a354a9dbc9f37c6e3519862b54e
859c60b416c1d83ee97c5bc898f8a530
859cda1a0bfcc952cfe00bc5062e2f80
85b7de1dc902e4acb290063f6701aae1
85c13a95b14e1b13f807af35e28c74b1
85d2d044a30099b388afecd3f71d5a4c
865d910c09cb23e2913494460cc08104
86bd2d37d1be295bcc0b0d377be6e3c9
Abundance
PMI (hrs)
location
External
Internal

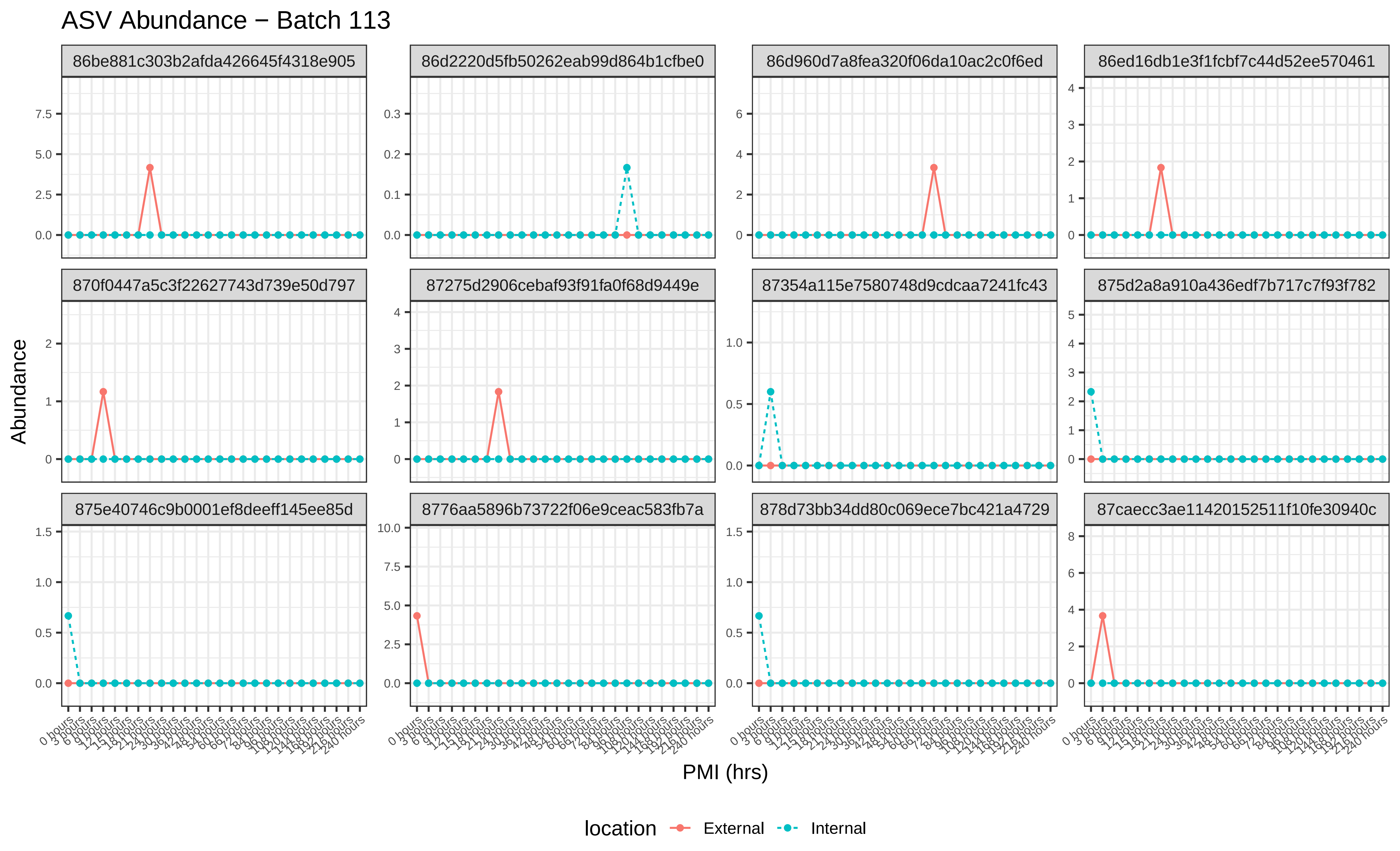

ASV Abundance − Batch 113
86be881c303b2afda426645f4318e905
86d2220d5fb50262eab99d864b1cfbe0
86d960d7a8fea320f06da10ac2c0f6ed
86ed16db1e3f1fcbf7c44d52ee570461
870f0447a5c3f22627743d739e50d797
87275d2906cebaf93f91fa0f68d9449e
87354a115e7580748d9cdcaa7241fc43
875d2a8a910a436edf7b717c7f93f782
875e40746c9b0001ef8deeff145ee85d
8776aa5896b73722f06e9ceac583fb7a
878d73bb34dd80c069ece7bc421a4729
87caecc3ae11420152511f10fe30940c
Abundance
PMI (hrs)
location
External
Internal

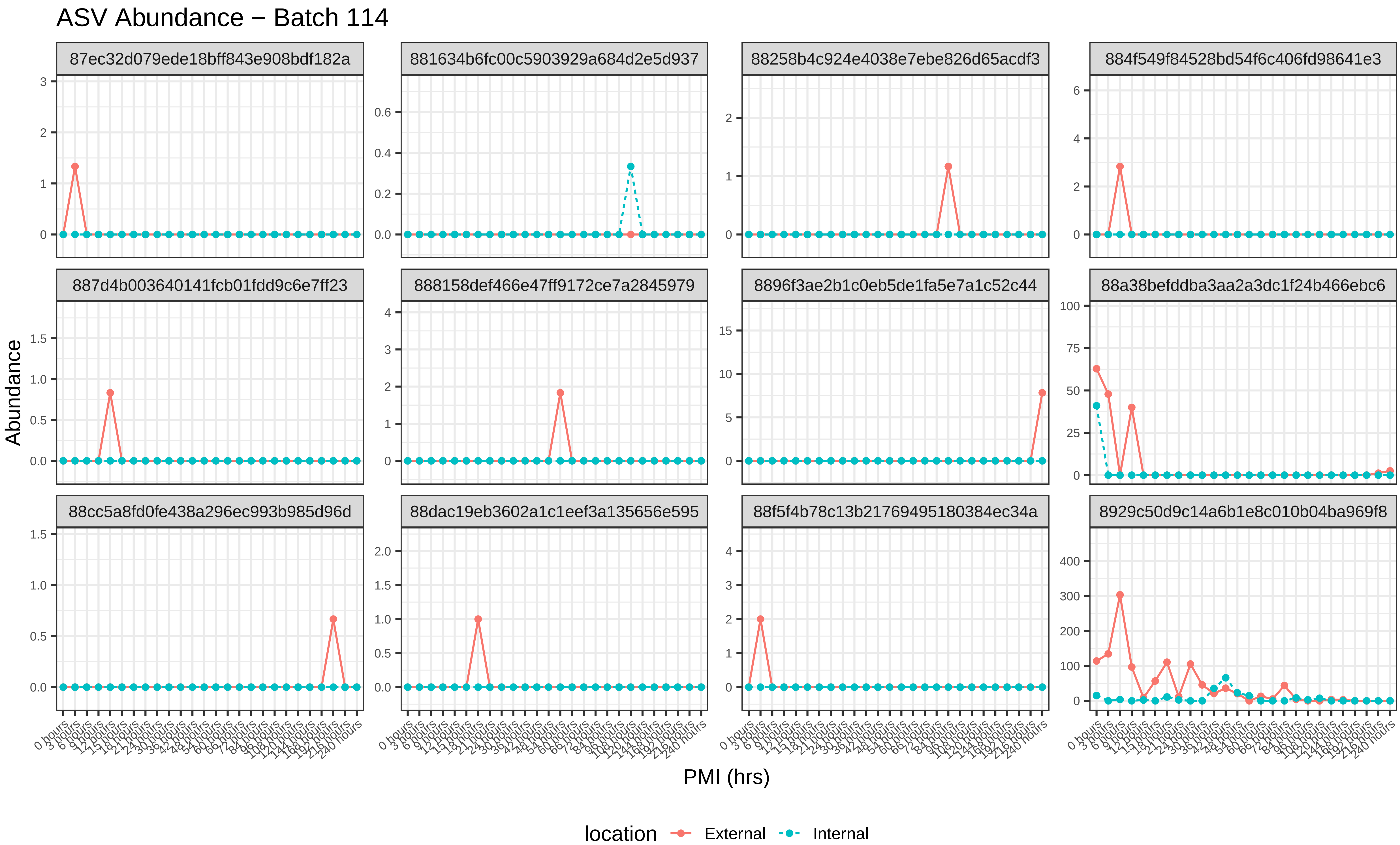
ASV Abundance − Batch 114
87ec32d079ede18bff843e908bdf182a
881634b6fc00c5903929a684d2e5d937
88258b4c924e4038e7ebe826d65acdf3
884f549f84528bd54f6c406fd98641e3
887d4b003640141fcb01fdd9c6e7ff23
888158def466e47ff9172ce7a2845979
8896f3ae2b1c0eb5de1fa5e7a1c52c44
88a38befddba3aa2a3dc1f24b466ebc6
88cc5a8fd0fe438a296ec993b985d96d
88dac19eb3602a1c1eef3a135656e595
88f5f4b78c13b21769495180384ec34a
8929c50d9c14a6b1e8c010b04ba969f8
Abundance
PMI (hrs)
location
External
Internal

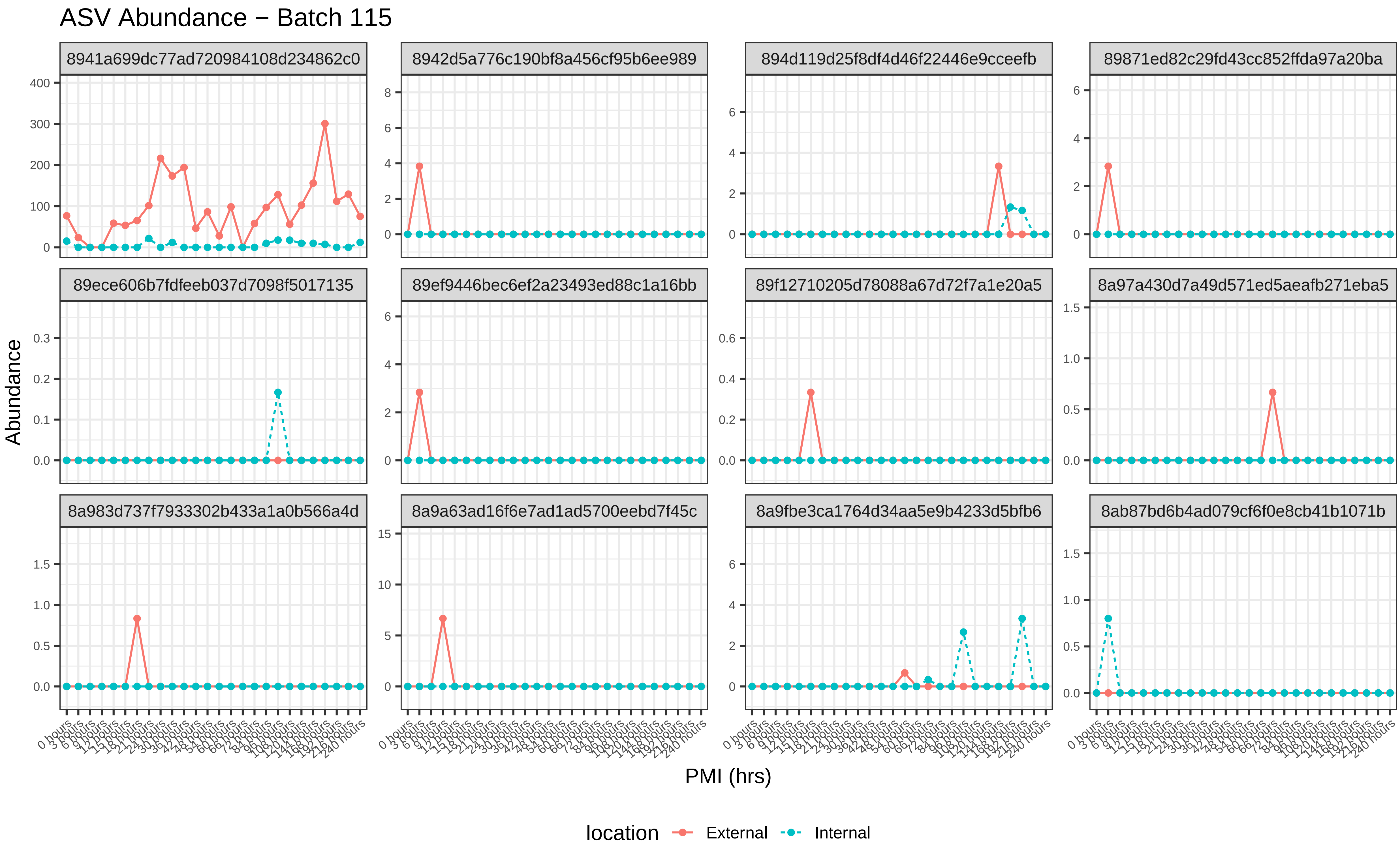
ASV Abundance − Batch 115
8941a699dc77ad720984108d234862c0
8942d5a776c190bf8a456cf95b6ee989
894d119d25f8df4d46f22446e9cceefb
89871ed82c29fd43cc852ffda97a20ba
89ece606b7fdfeeb037d7098f5017135
89ef9446bec6ef2a23493ed88c1a16bb
89f12710205d78088a67d72f7a1e20a5
8a97a430d7a49d571ed5aeafb271eba5
8a983d737f7933302b433a1a0b566a4d
8a9a63ad16f6e7ad1ad5700eebd7f45c
8a9fbe3ca1764d34aa5e9b4233d5bfb6
8ab87bd6b4ad079cf6f0e8cb41b1071b
Abundance
PMI (hrs)
location
External
Internal

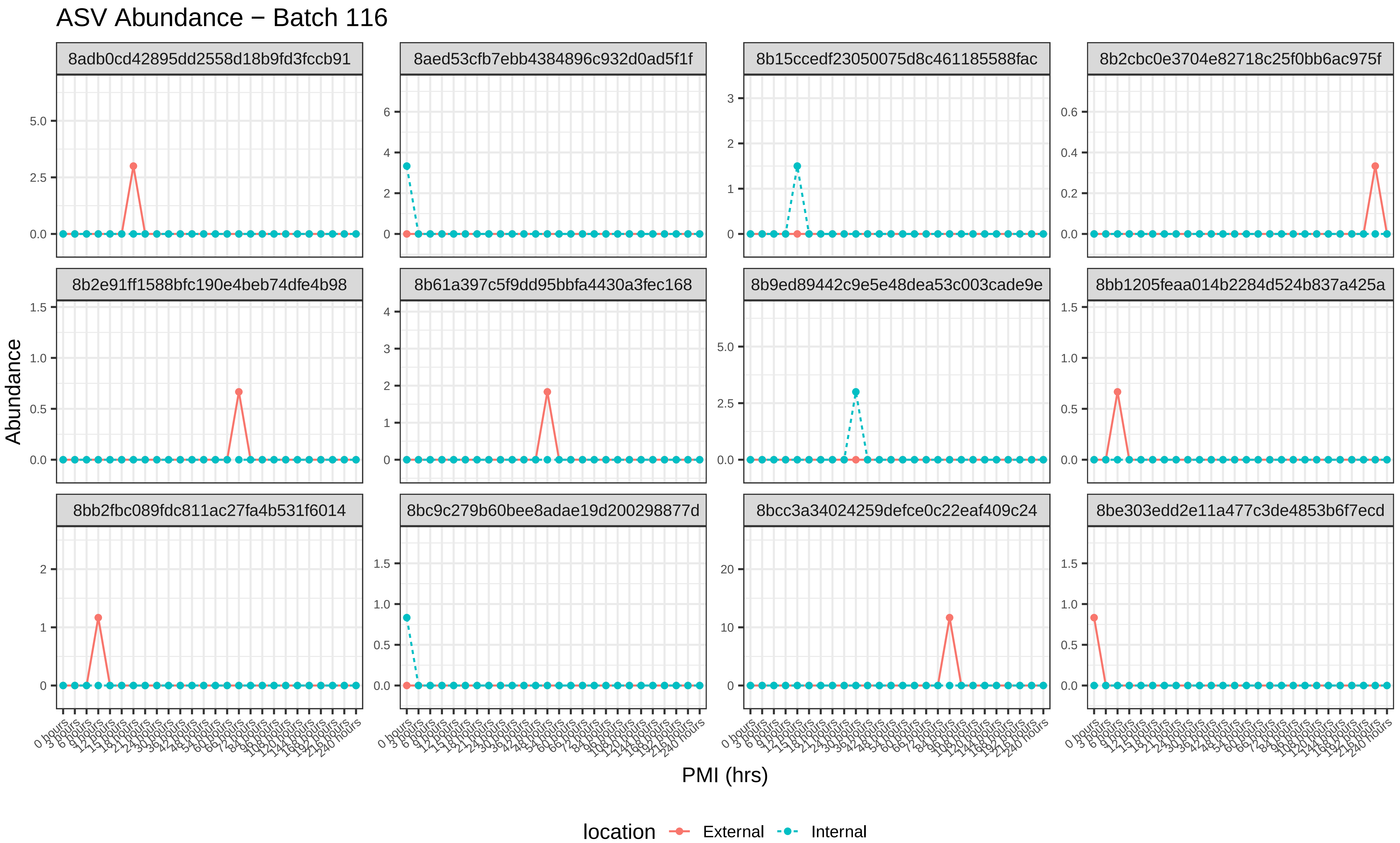
ASV Abundance − Batch 116
8adb0cd42895dd2558d18b9fd3fccb91
8aed53cfb7ebb4384896c932d0ad5f1f
8b15ccedf23050075d8c461185588fac
8b2cbc0e3704e82718c25f0bb6ac975f
8b2e91ff1588bfc190e4beb74dfe4b98
8b61a397c5f9dd95bbfa4430a3fec168
8b9ed89442c9e5e48dea53c003cade9e
8bb1205feaa014b2284d524b837a425a
8bb2fbc089fdc811ac27fa4b531f6014
8bc9c279b60bee8adae19d200298877d
8bcc3a34024259defce0c22eaf409c24
8be303edd2e11a477c3de4853b6f7ecd
Abundance
PMI (hrs)
0 hours
3 hours
6 hours
9 hours
12 hours
15 hours
18 hours
21 hours
24 hours
30 hours
36 hours
42 hours
48 hours
54 hours
60 hours
66 hours
72 hours
84 hours
96 hours
108 hours
120 hours
144 hours
168 hours
192 hours
216 hours
240 hours
location
External
Internal

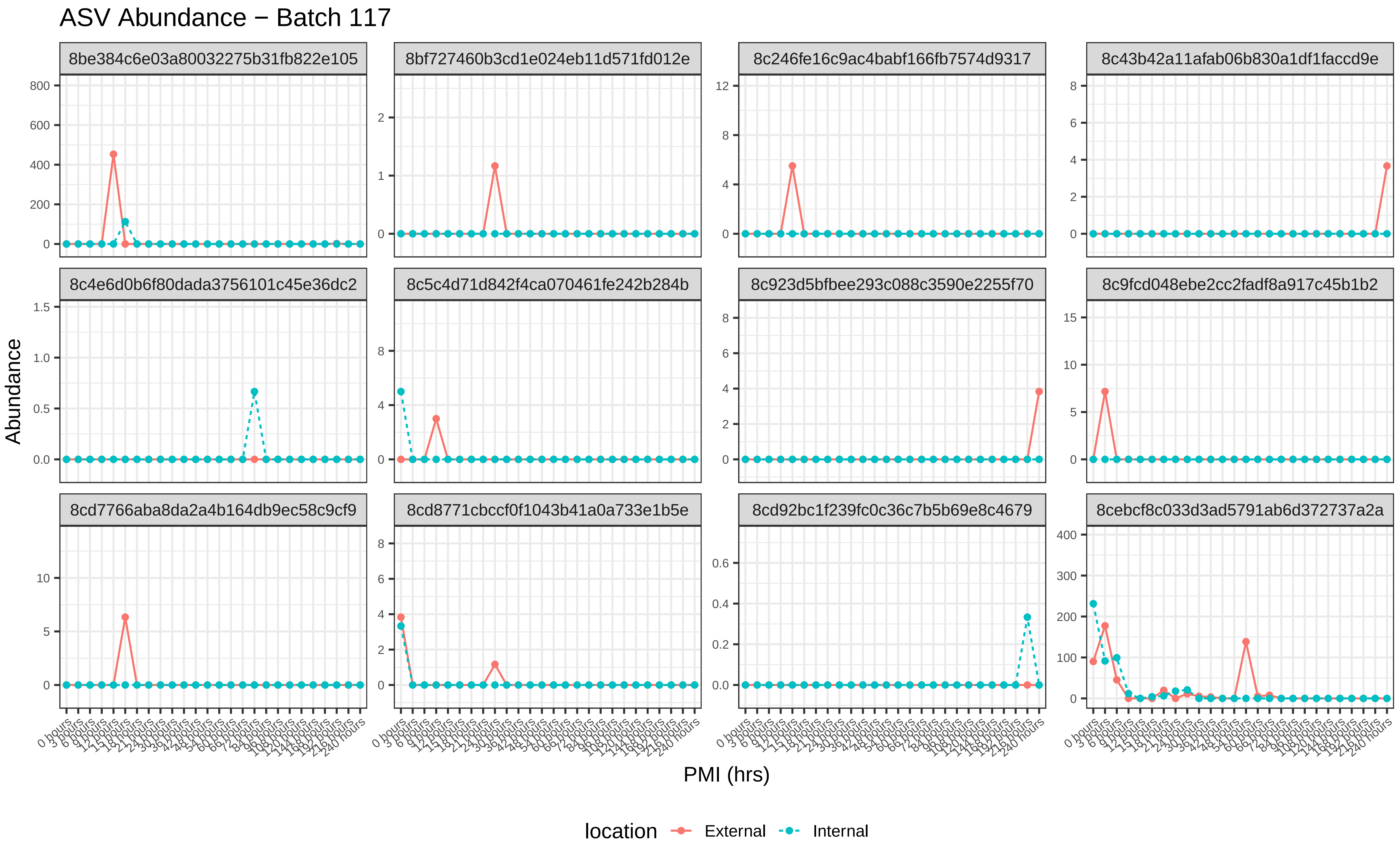

ASV Abundance − Batch 117
8be384c6e03a80032275b31fb822e105
8bf727460b3cd1e024eb11d571fd012e
8c246fe16c9ac4babf166fb7574d9317
8c43b42a11afab06b830a1df1faccd9e
8c4e6d0b6f80dada3756101c45e36dc2
8c5c4d71d842f4ca070461fe242b284b
8c923d5bfbee293c088c3590e2255f70
8c9fcd048ebe2cc2fadf8a917c45b1b2
8cd7766aba8da2a4b164db9ec58c9cf9
8cd8771cbccf0f1043b41a0a733e1b5e
8cd92bc1f239fc0c36c7b5b69e8c4679
8cebcf8c033d3ad5791ab6d372737a2a
Abundance
PMI (hrs)
location
External
Internal

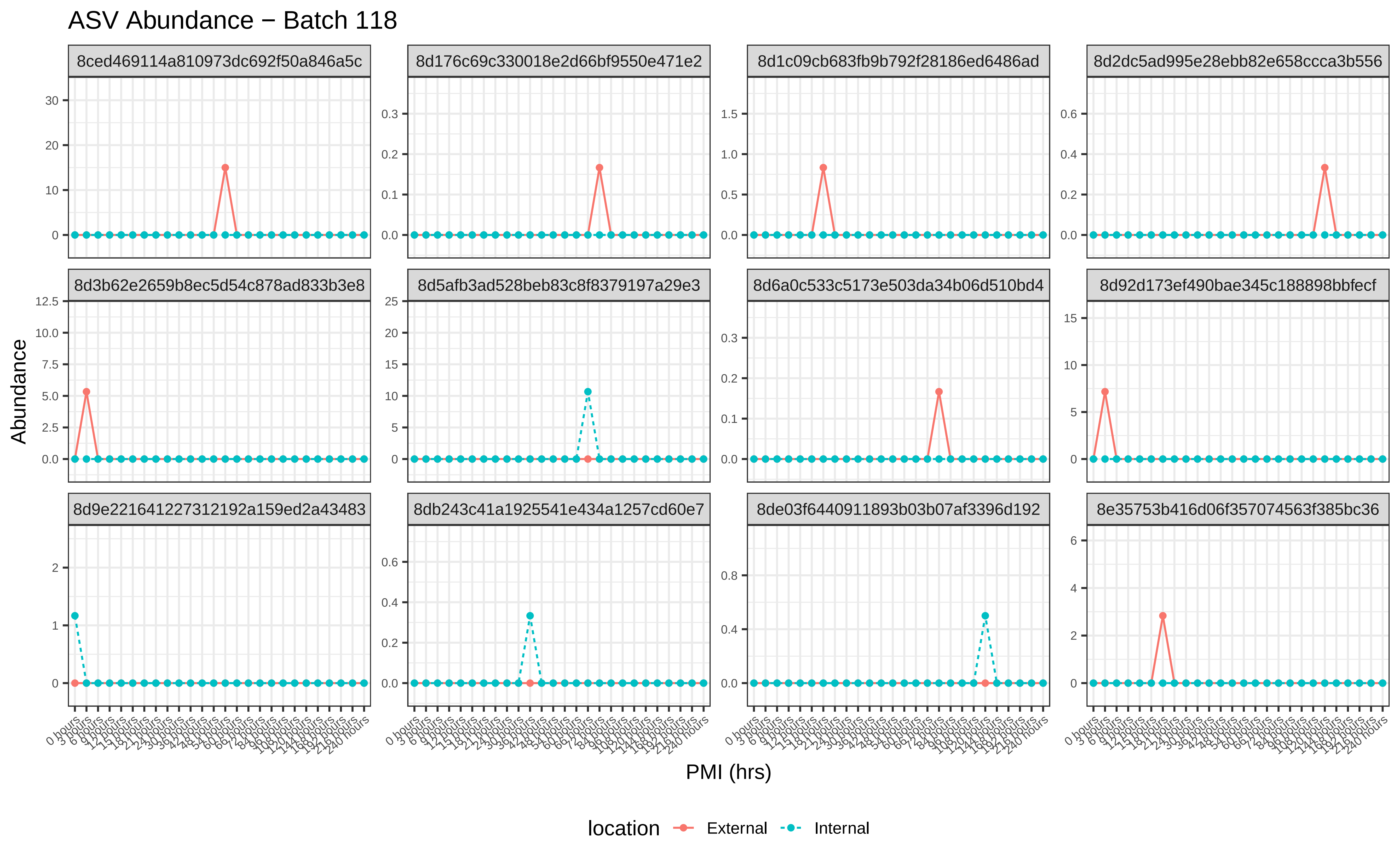
ASV Abundance − Batch 118
8ced469114a810973dc692f50a846a5c
8d176c69c330018e2d66bf9550e471e2
8d1c09cb683fb9b792f28186ed6486ad
8d2dc5ad995e28ebb82e658ccca3b556
8d3b62e2659b8ec5d54c878ad833b3e8
8d5afb3ad528beb83c8f8379197a29e3
8d6a0c533c5173e503da34b06d510bd4
8d92d173ef490bae345c188898bbfecf
8d9e221641227312192a159ed2a43483
8db243c41a1925541e434a1257cd60e7
8de03f6440911893b03b07af3396d192
8e35753b416d06f357074563f385bc36
Abundance
PMI (hrs)
location
External
Internal

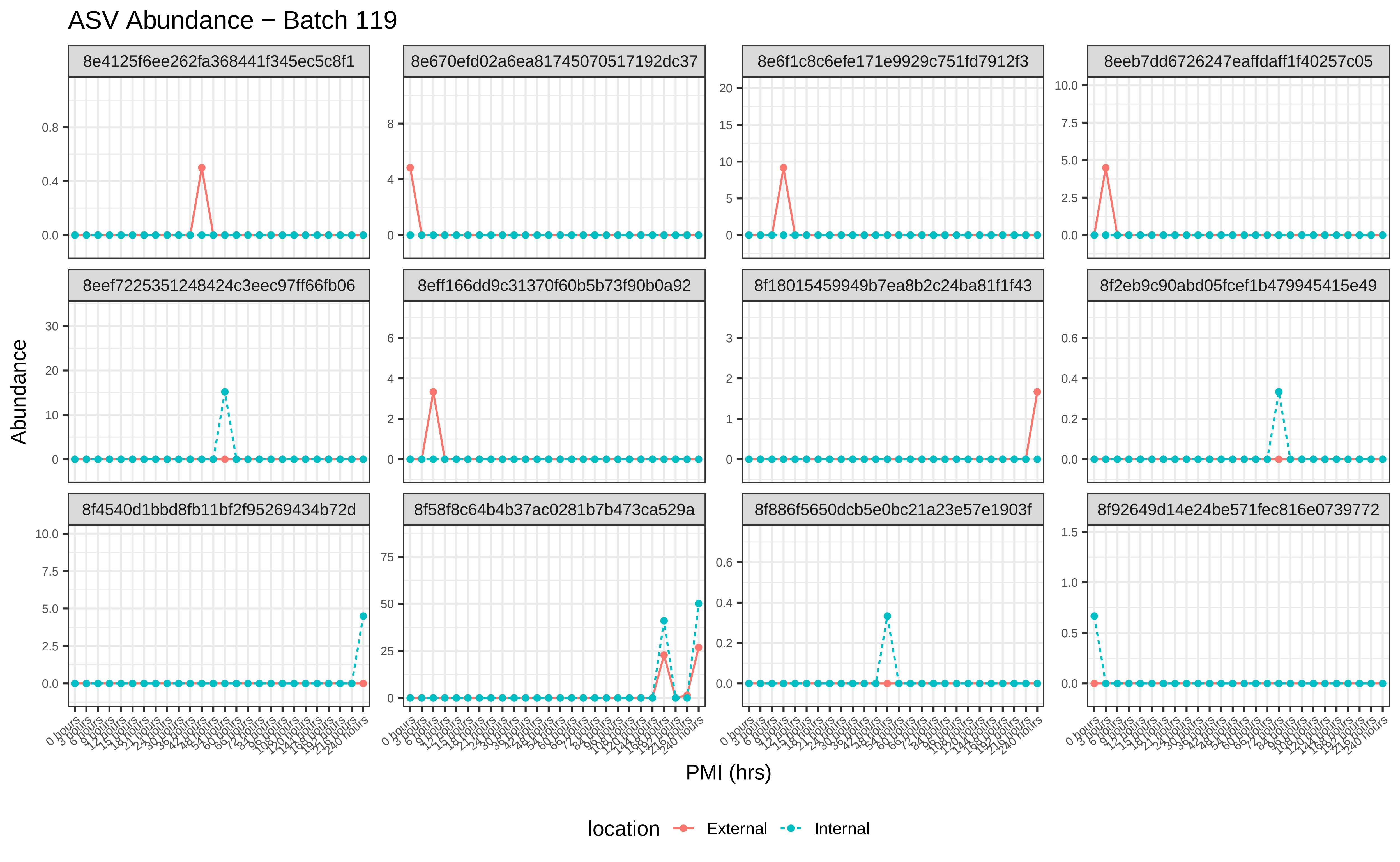
ASV Abundance − Batch 119
8e4125f6ee262fa368441f345ec5c8f1
8e670efd02a6ea81745070517192dc37
8e6f1c8c6efe171e9929c751fd7912f3
8eeb7dd6726247eaffdaff1f40257c05
8eef7225351248424c3eec97ff66fb06
8eff166dd9c31370f60b5b73f90b0a92
8f18015459949b7ea8b2c24ba81f1f43
8f2eb9c90abd05fcef1b479945415e49
8f4540d1bbd8fb11bf2f95269434b72d
8f58f8c64b4b37ac0281b7b473ca529a
8f886f5650dcb5e0bc21a23e57e1903f
8f92649d14e24be571fec816e0739772
Abundance
PMI (hrs)
location
External
Internal

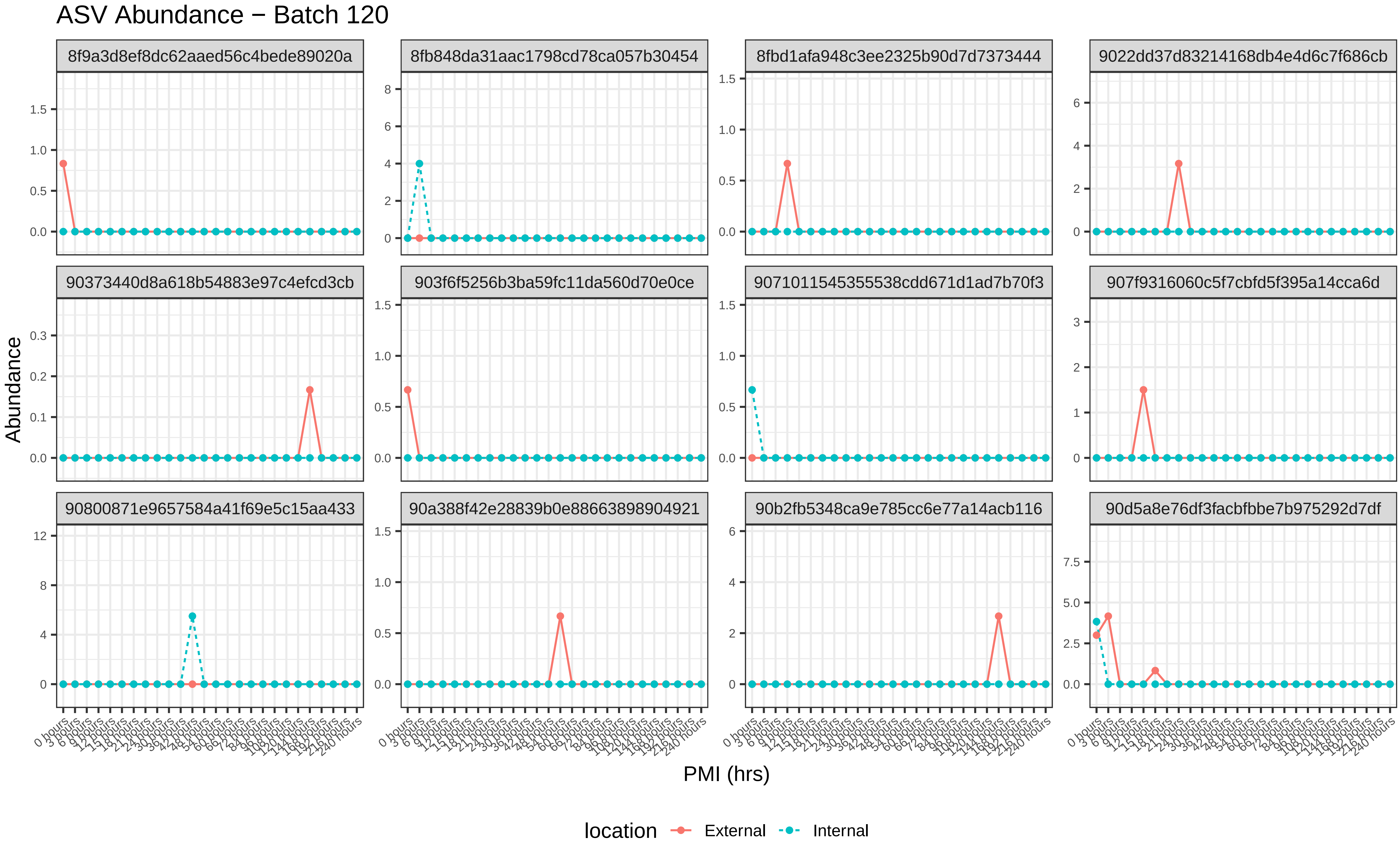
ASV Abundance − Batch 120
8f9a3d8ef8dc62aaed56c4bede89020a
8fb848da31aac1798cd78ca057b30454
8fbd1afa948c3ee2325b90d7d7373444
9022dd37d83214168db4e4d6c7f686cb
90373440d8a618b54883e97c4efcd3cb
903f6f5256b3ba59fc11da560d70e0ce
9071011545355538cdd671d1ad7b70f3
907f9316060c5f7cbfd5f395a14cca6d
90800871e9657584a41f69e5c15aa433
90a388f42e28839b0e88663898904921
90b2fb5348ca9e785cc6e77a14acb116
90d5a8e76df3facbfbbe7b975292d7df
Abundance
PMI (hrs)
location
External
Internal

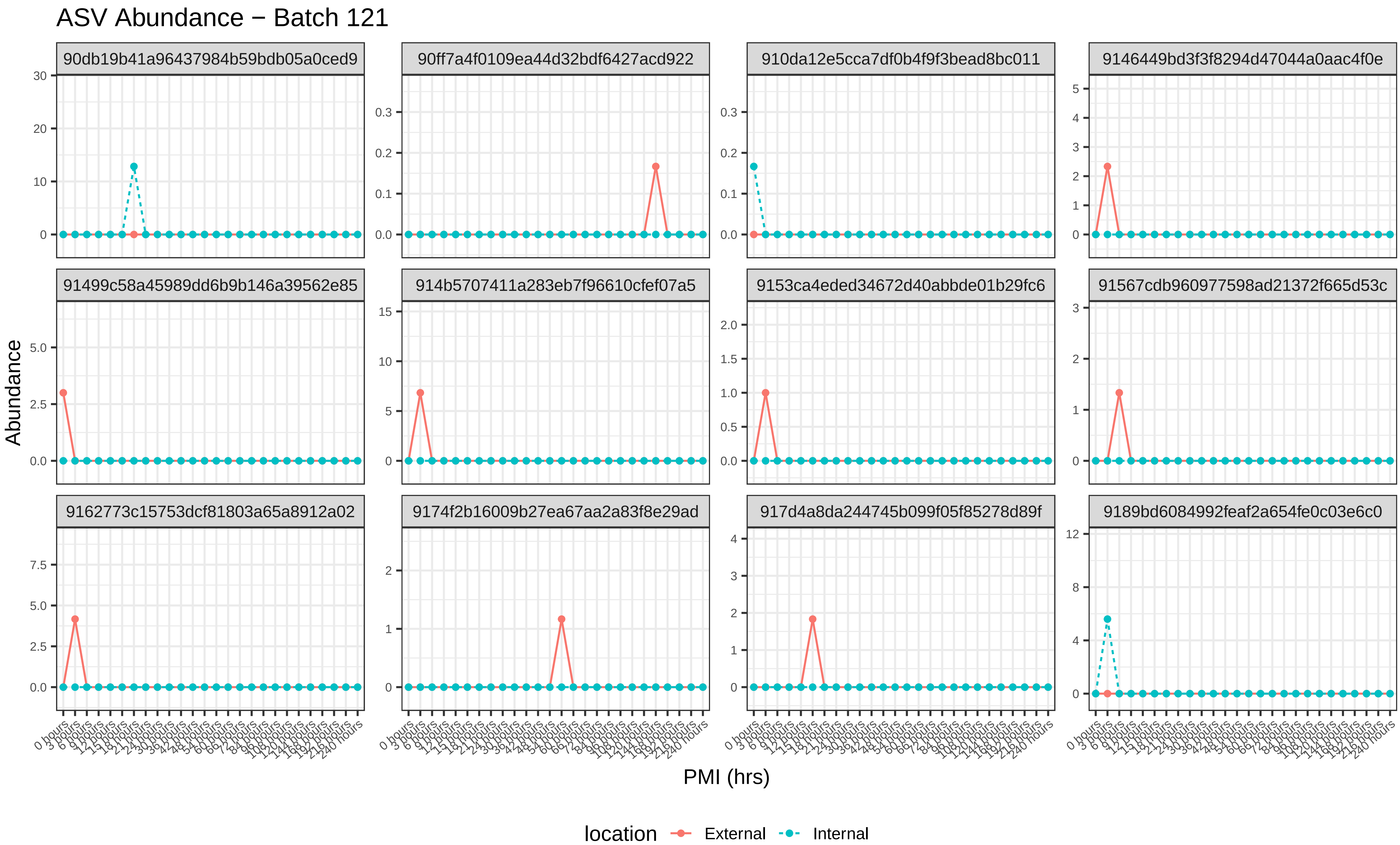

ASV Abundance − Batch 121
90db19b41a96437984b59bdb05a0ced9
90ff7a4f0109ea44d32bdf6427acd922
910da12e5cca7df0b4f9f3bead8bc011
9146449bd3f3f8294d47044a0aac4f0e
91499c58a45989dd6b9b146a39562e85
914b5707411a283eb7f96610cfef07a5
9153ca4eded34672d40abbde01b29fc6
91567cdb960977598ad21372f665d53c
9162773c15753dcf81803a65a8912a02
9174f2b16009b27ea67aa2a83f8e29ad
917d4a8da244745b099f05f85278d89f
9189bd6084992feaf2a654fe0c03e6c0
Abundance
PMI (hrs)
location
External
Internal

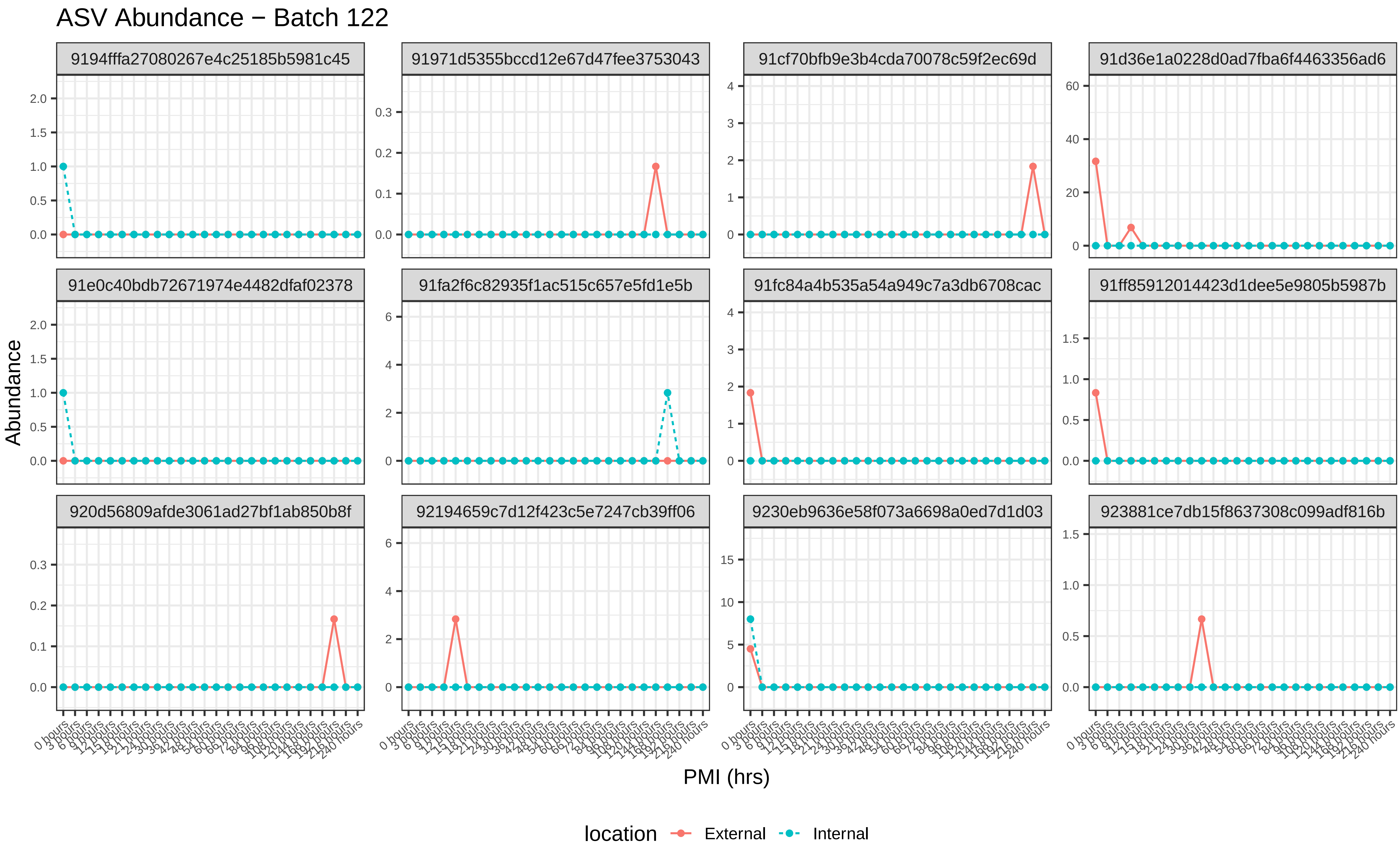
ASV Abundance − Batch 122
9194fffa27080267e4c25185b5981c45
91971d5355bccd12e67d47fee3753043
91cf70bfb9e3b4cda70078c59f2ec69d
91d36e1a0228d0ad7fba6f4463356ad6
91e0c40bdb72671974e4482dfaf02378
91fa2f6c82935f1ac515c657e5fd1e5b
91fc84a4b535a54a949c7a3db6708cac
91ff85912014423d1dee5e9805b5987b
920d56809afde3061ad27bf1ab850b8f
92194659c7d12f423c5e7247cb39ff06
9230eb9636e58f073a6698a0ed7d1d03
923881ce7db15f8637308c099adf816b
Abundance
PMI (hrs)
location
External
Internal

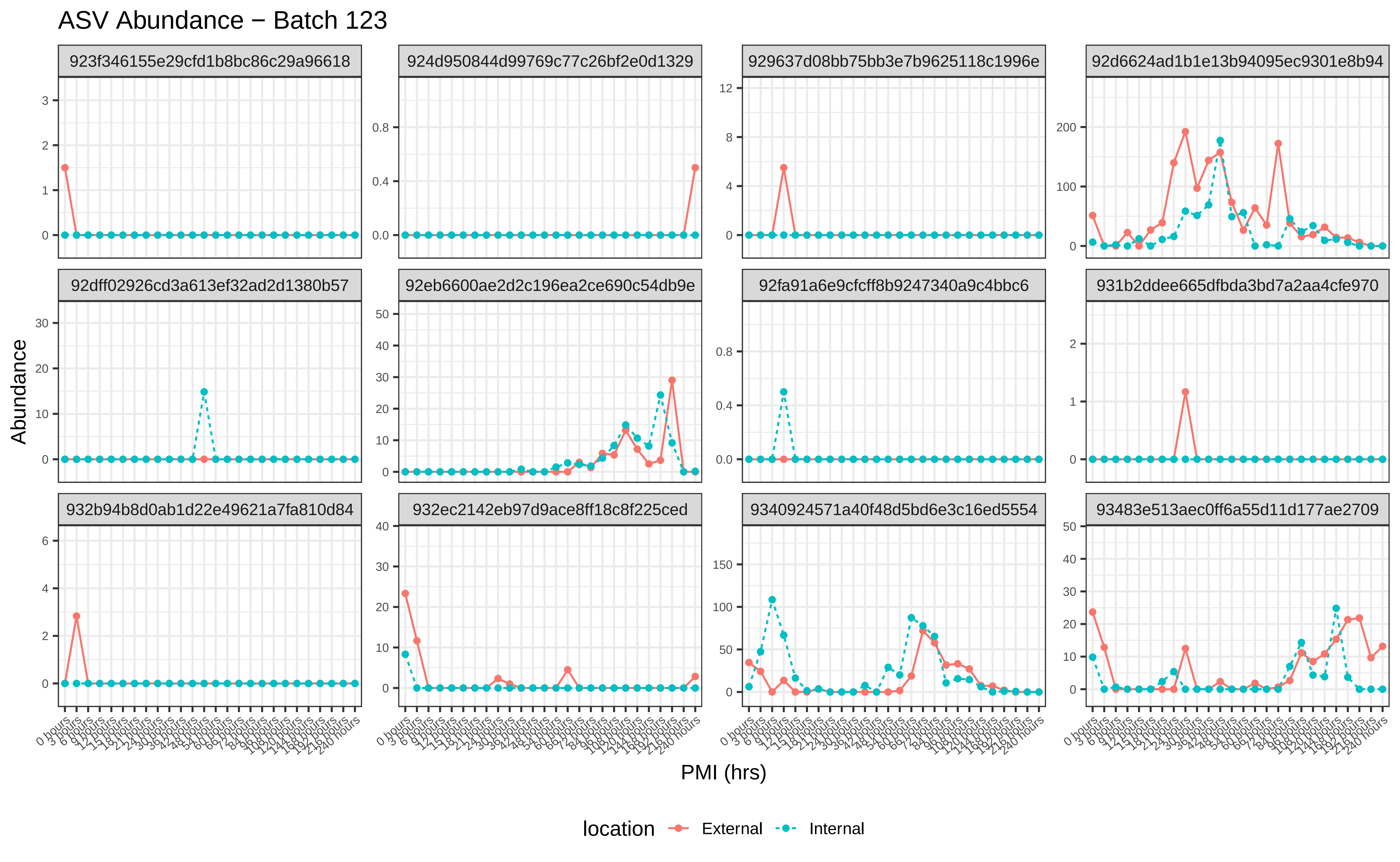
ASV Abundance − Batch 123
923f346155e29cfd1b8bc86c29a96618
924d950844d99769c77c26bf2e0d1329
929637d08bb75bb3e7b9625118c1996e
92d6624ad1b1e13b94095ec9301e8b94
92dff02926cd3a613ef32ad2d1380b57
92eb6600ae2d2c196ea2ce690c54db9e
92fa91a6e9cfcff8b9247340a9c4bbc6
931b2ddee665dfbda3bd7a2aa4cfe970
932b94b8d0ab1d22e49621a7fa810d84
932ec2142eb97d9ace8ff18c8f225ced
9340924571a40f48d5bd6e3c16ed5554
93483e513aec0ff6a55d11d177ae2709
Abundance
PMI (hrs)
location
External
Internal

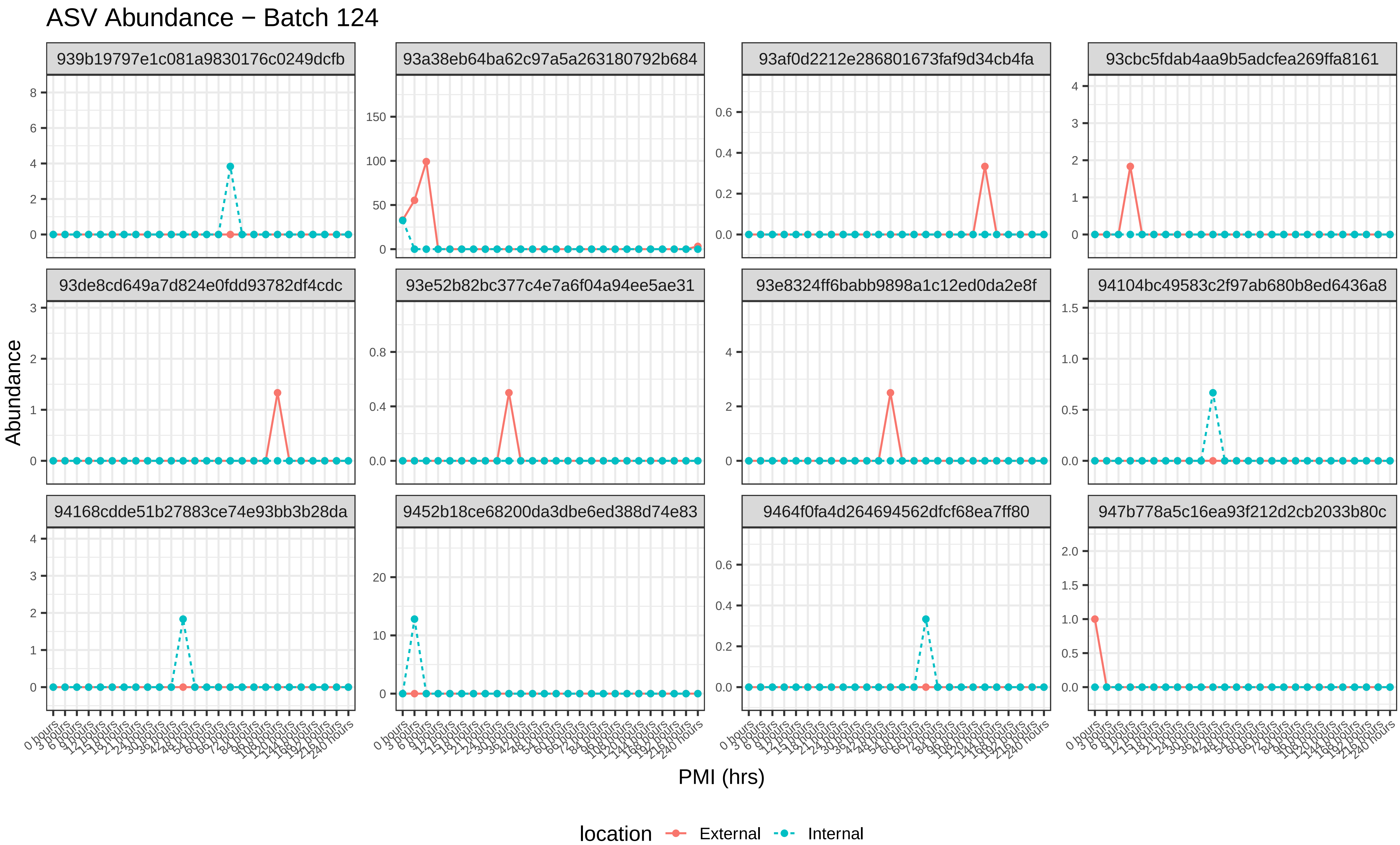

ASV Abundance − Batch 124
939b19797e1c081a9830176c0249dcfb
93a38eb64ba62c97a5a263180792b684
93af0d2212e286801673faf9d34cb4fa
93cbc5fdab4aa9b5adcfea269ffa8161
93de8cd649a7d824e0fdd93782df4cdc
93e52b82bc377c4e7a6f04a94ee5ae31
93e8324ff6babb9898a1c12ed0da2e8f
94104bc49583c2f97ab680b8ed6436a8
94168cdde51b27883ce74e93bb3b28da
9452b18ce68200da3dbe6ed388d74e83
9464f0fa4d264694562dfcf68ea7ff80
947b778a5c16ea93f212d2cb2033b80c
Abundance
PMI (hrs)
location
External
Internal

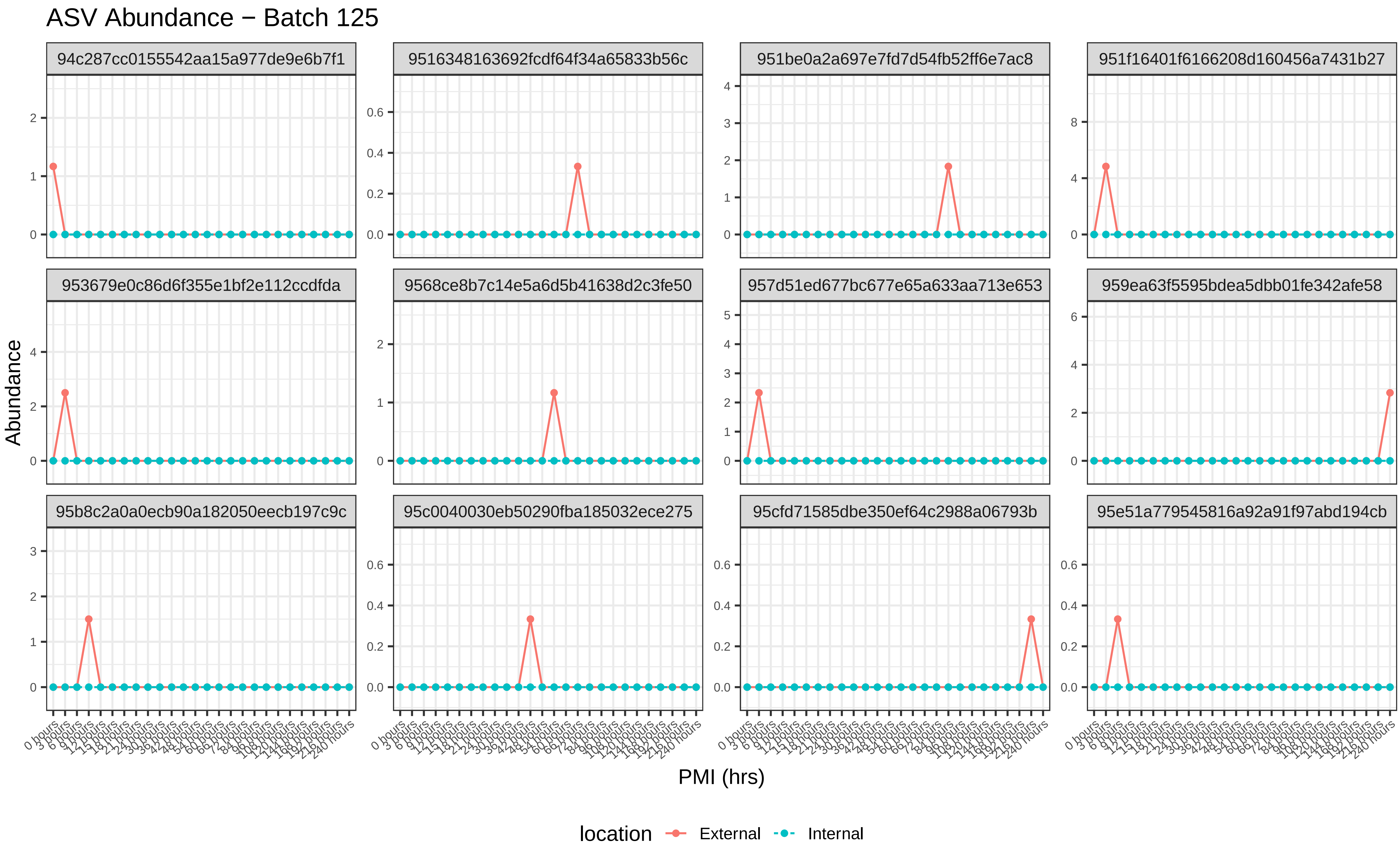
ASV Abundance − Batch 125
94c287cc0155542aa15a977de9e6b7f1
9516348163692fcdf64f34a65833b56c
951be0a2a697e7fd7d54fb52ff6e7ac8
951f16401f6166208d160456a7431b27
953679e0c86d6f355e1bf2e112ccdfda
9568ce8b7c14e5a6d5b41638d2c3fe50
957d51ed677bc677e65a633aa713e653
959ea63f5595bdea5dbb01fe342afe58
95b8c2a0a0ecb90a182050eecb197c9c
95c0040030eb50290fba185032ece275
95cfd71585dbe350ef64c2988a06793b
95e51a779545816a92a91f97abd194cb
Abundance
PMI (hrs)
location
External
Internal

ASV Abundance − Batch 126

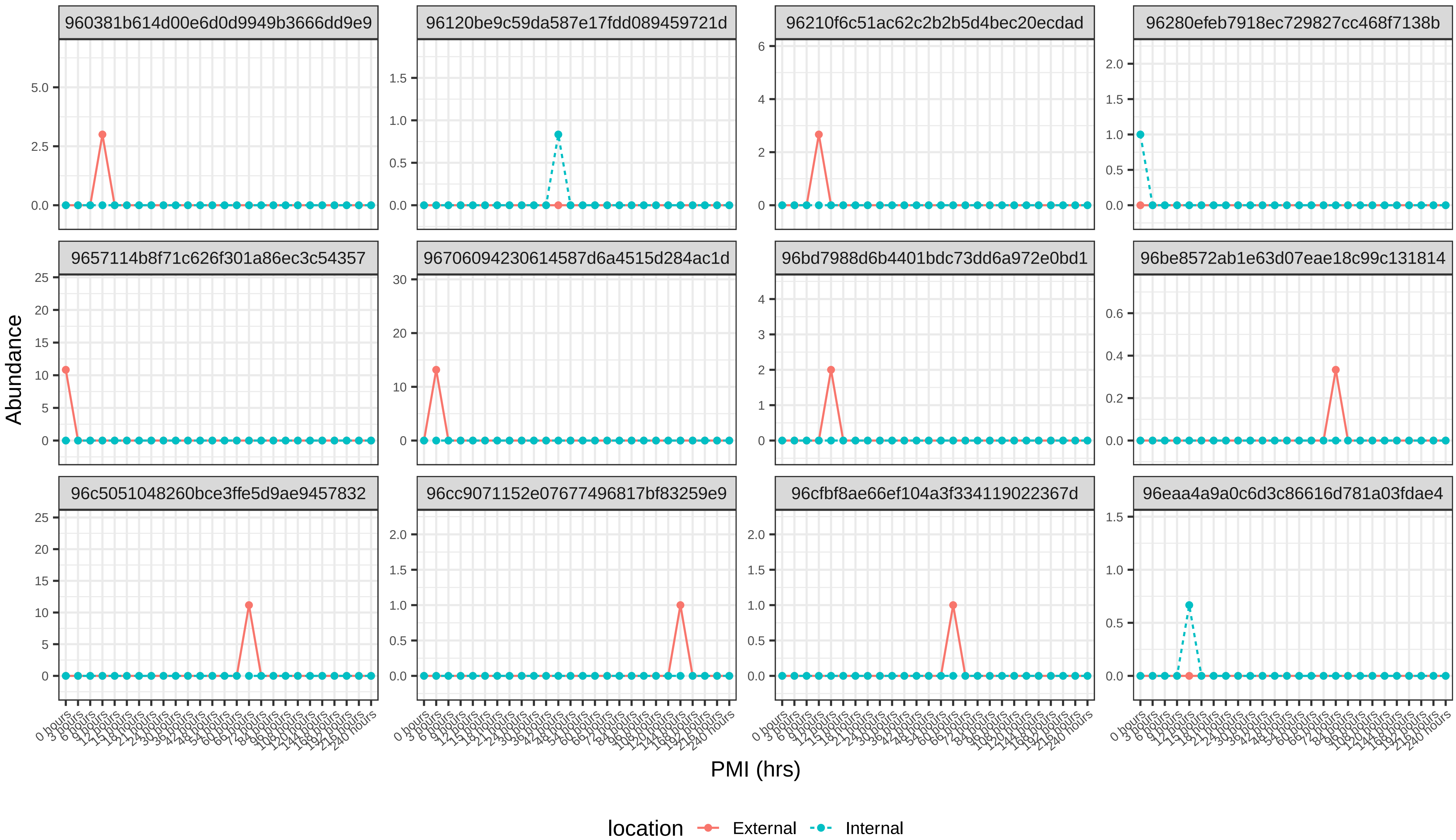

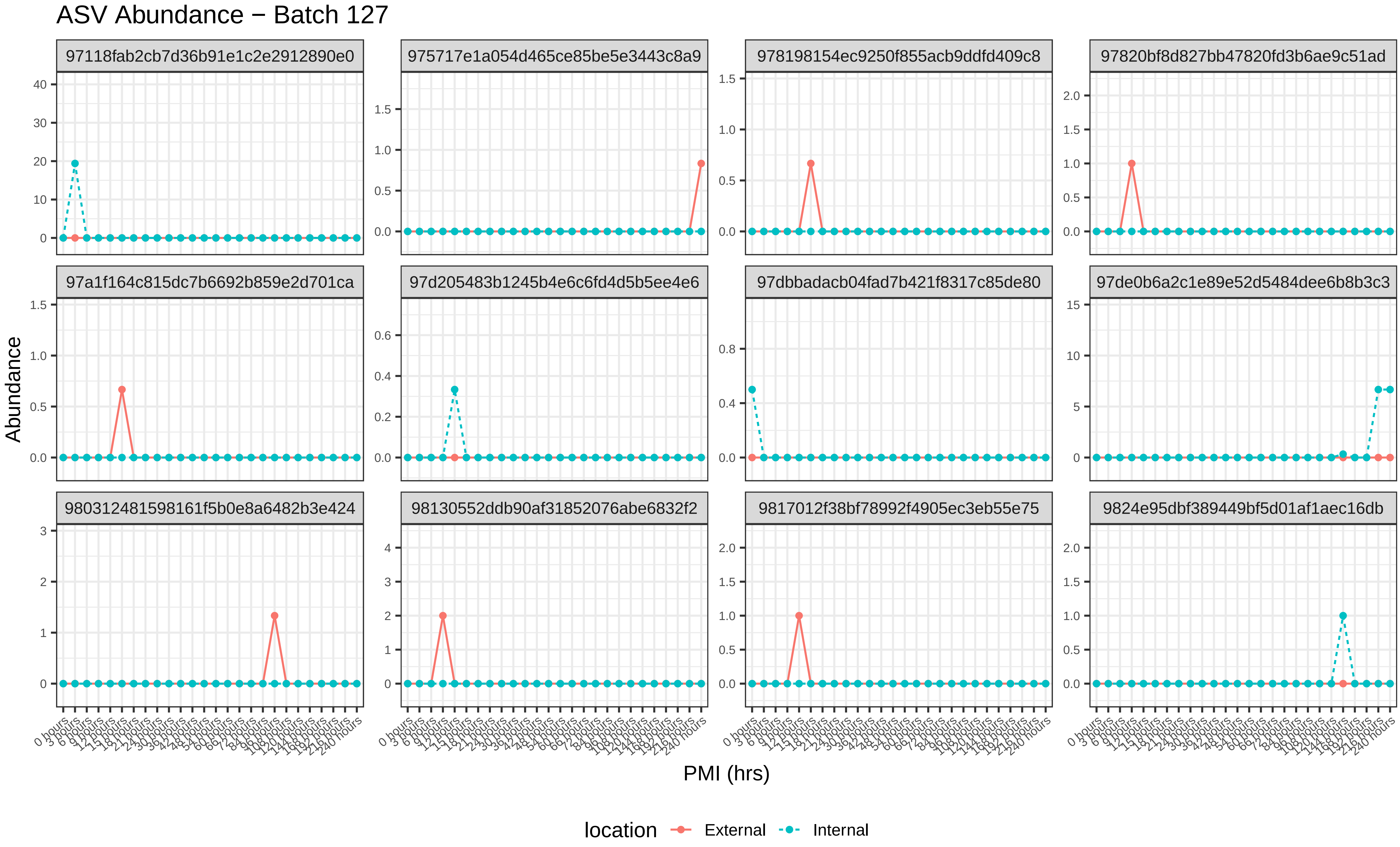

ASV Abundance − Batch 127
97118fab2cb7d36b91e1c2e2912890e0
975717e1a054d465ce85be5e3443c8a9
978198154ec9250f855acb9ddfd409c8
97820bf8d827bb47820fd3b6ae9c51ad
97a1f164c815dc7b6692b859e2d701ca
97d205483b1245b4e6c6fd4d5b5ee4e6
97dbbadacb04fad7b421f8317c85de80
97de0b6a2c1e89e52d5484dee6b8b3c3
980312481598161f5b0e8a6482b3e424
98130552ddb90af31852076abe6832f2
9817012f38bf78992f4905ec3eb55e75
9824e95dbf389449bf5d01af1aec16db
Abundance
PMI (hrs)
location
External
Internal

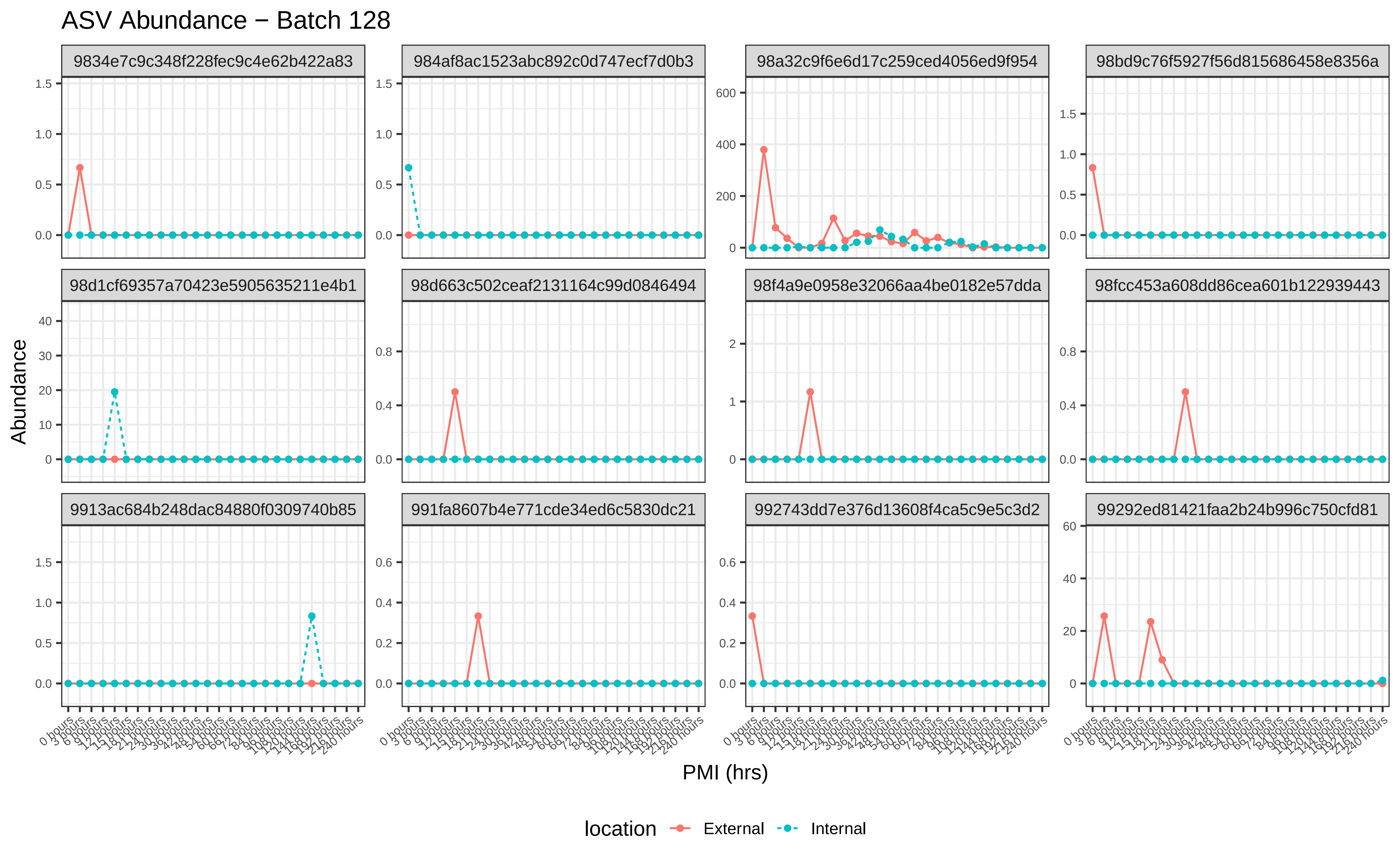

ASV Abundance − Batch 128
9834e7c9c348f228fec9c4e62b422a83
984af8ac1523abc892c0d747ecf7d0b3
98a32c9f6e6d17c259ced4056ed9f954
98bd9c76f5927f56d815686458e8356a
98d1cf69357a70423e5905635211e4b1
98d663c502ceaf2131164c99d0846494
98f4a9e0958e32066aa4be0182e57dda
98fcc453a608dd86cea601b122939443
9913ac684b248dac84880f0309740b85
991fa8607b4e771cde34ed6c5830dc21
992743dd7e376d13608f4ca5c9e5c3d2
99292ed81421faa2b24b996c750cfd81
Abundance
PMI (hrs)
location
External
Internal

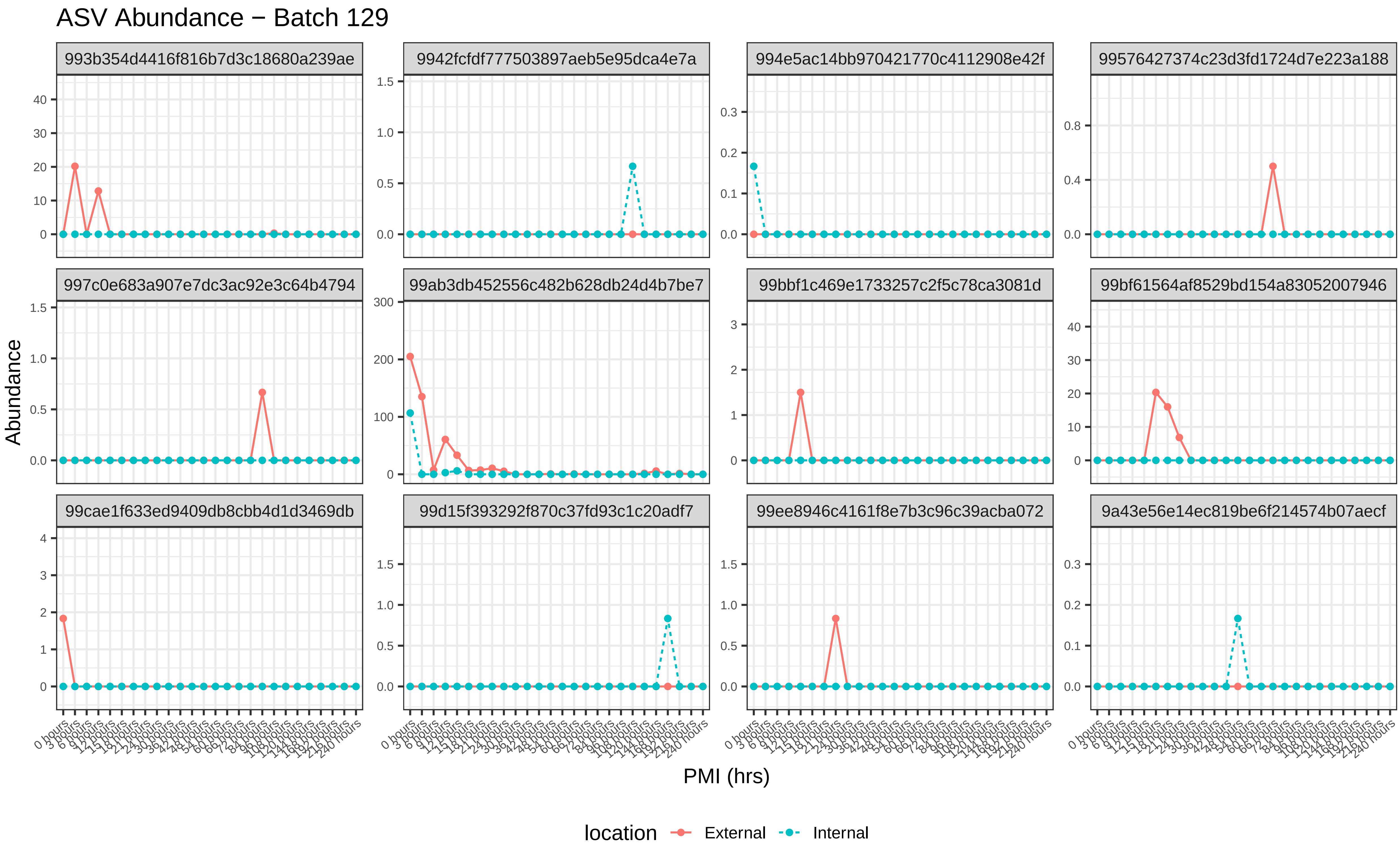

ASV Abundance − Batch 129
993b354d4416f816b7d3c18680a239ae
9942fcfdf777503897aeb5e95dca4e7a
994e5ac14bb970421770c4112908e42f
99576427374c23d3fd1724d7e223a188
997c0e683a907e7dc3ac92e3c64b4794
99ab3db452556c482b628db24d4b7be7
99bbf1c469e1733257c2f5c78ca3081d
99bf61564af8529bd154a83052007946
99cae1f633ed9409db8cbb4d1d3469db
99d15f393292f870c37fd93c1c20adf7
99ee8946c4161f8e7b3c96c39acba072
9a43e56e14ec819be6f214574b07aecf
Abundance
PMI (hrs)
location
External
Internal

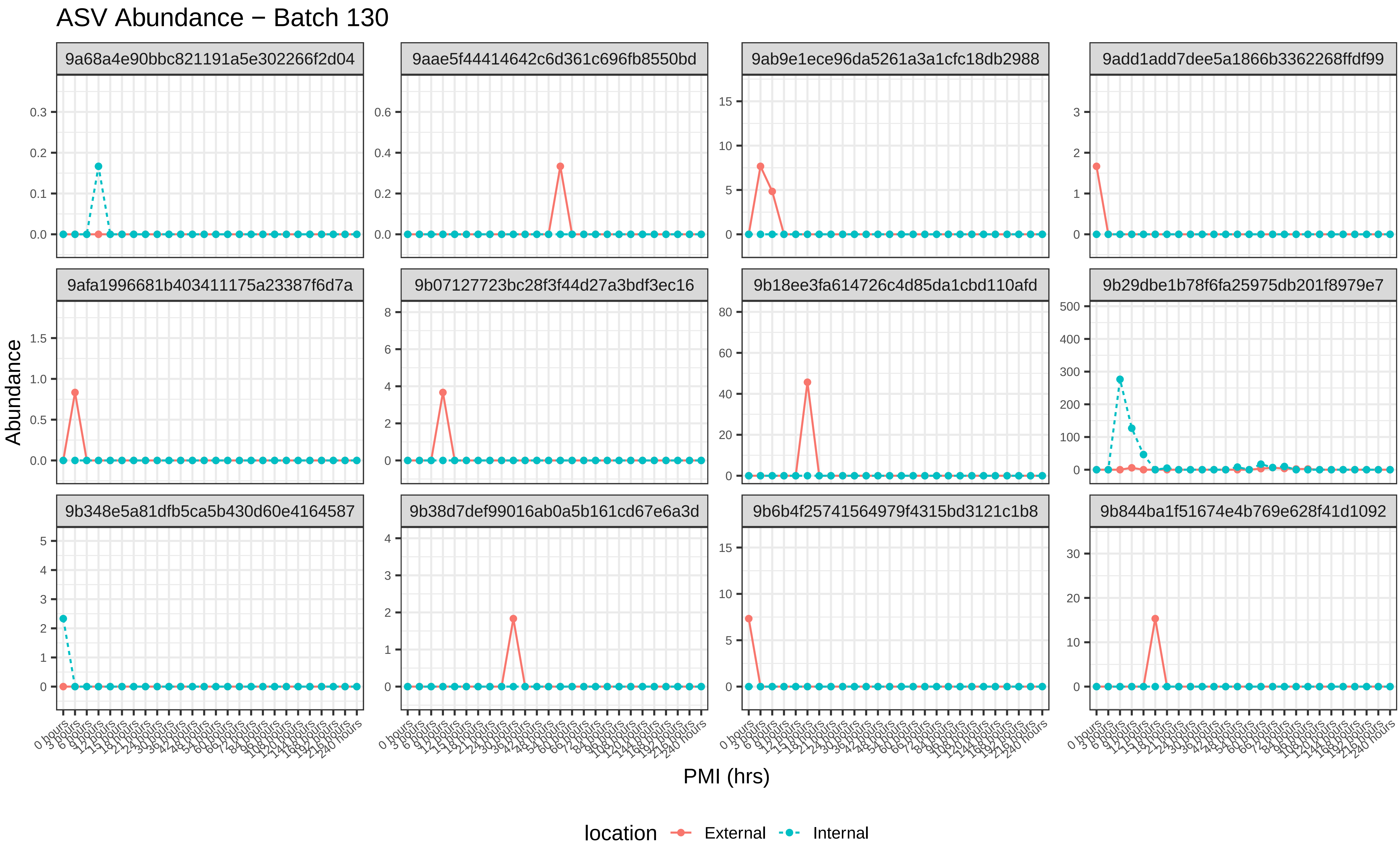
ASV Abundance − Batch 130
9a68a4e90bbc821191a5e302266f2d04
9aae5f44414642c6d361c696fb8550bd
9ab9e1ece96da5261a3a1cfc18db2988
9add1add7dee5a1866b3362268ffdf99
9afa1996681b403411175a23387f6d7a
9b07127723bc28f3f44d27a3bdf3ec16
9b18ee3fa614726c4d85da1cbd110afd
9b29dbe1b78f6fa25975db201f8979e7
9b348e5a81dfb5ca5b430d60e4164587
9b38d7def99016ab0a5b161cd67e6a3d
9b6b4f25741564979f4315bd3121c1b8
9b844ba1f51674e4b769e628f41d1092
Abundance
PMI (hrs)
location
External
Internal

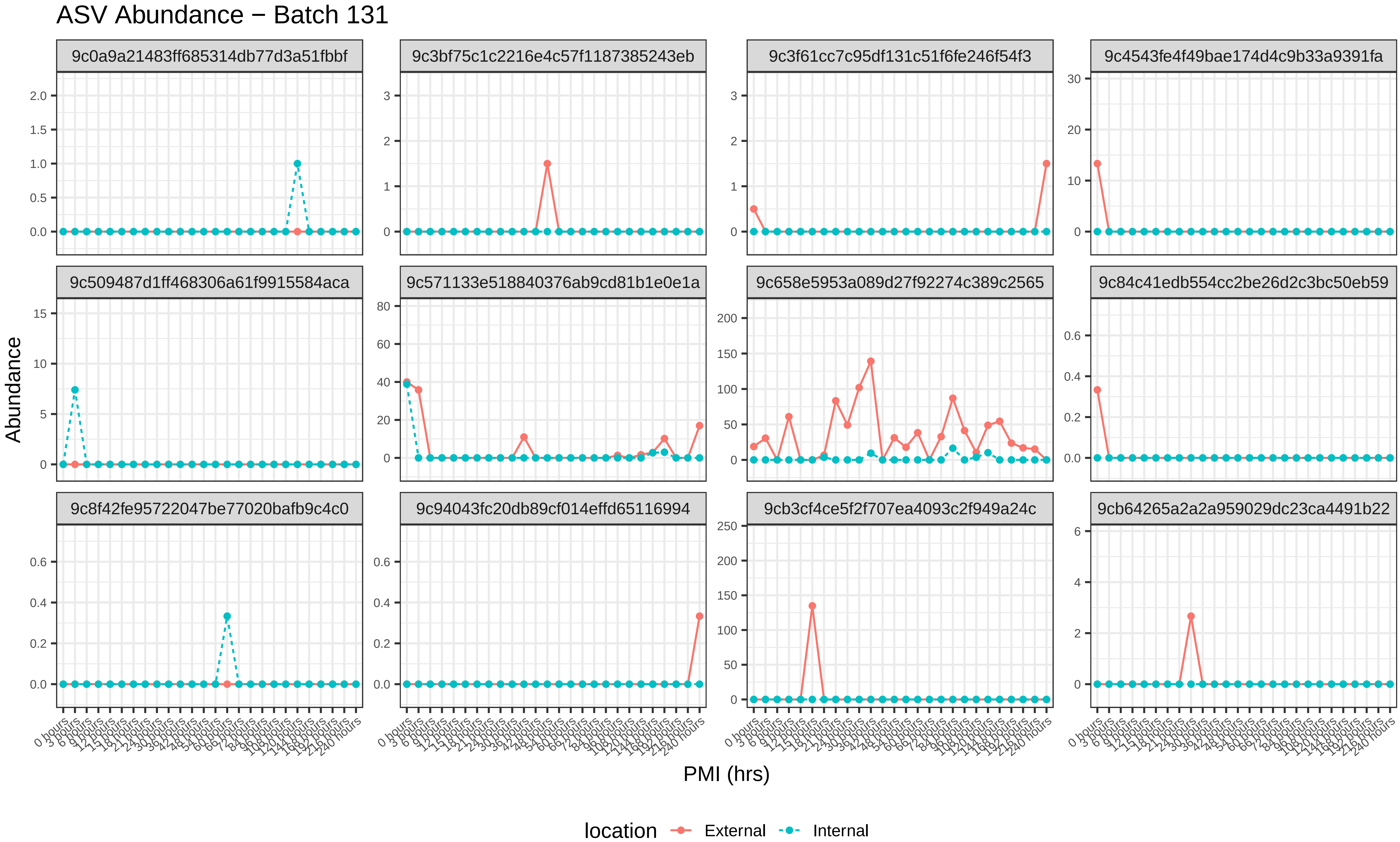
ASV Abundance − Batch 131
9c0a9a21483ff685314db77d3a51fbbf
9c3bf75c1c2216e4c57f1187385243eb
9c3f61cc7c95df131c51f6fe246f54f3
9c4543fe4f49bae174d4c9b33a9391fa
9c509487d1ff468306a61f9915584aca
9c571133e518840376ab9cd81b1e0e1a
9c658e5953a089d27f92274c389c2565
9c84c41edb554cc2be26d2c3bc50eb59
9c8f42fe95722047be77020bafb9c4c0
9c94043fc20db89cf014effd65116994
9cb3cf4ce5f2f707ea4093c2f949a24c
9cb64265a2a2a959029dc23ca4491b22
Abundance
PMI (hrs)
0 hours
3 hours
6 hours
9 hours
12 hours
15 hours
18 hours
21 hours
24 hours
30 hours
36 hours
42 hours
48 hours
54 hours
60 hours
66 hours
72 hours
84 hours
96 hours
108 hours
120 hours
144 hours
168 hours
192 hours
216 hours
240 hours
location
External
Internal

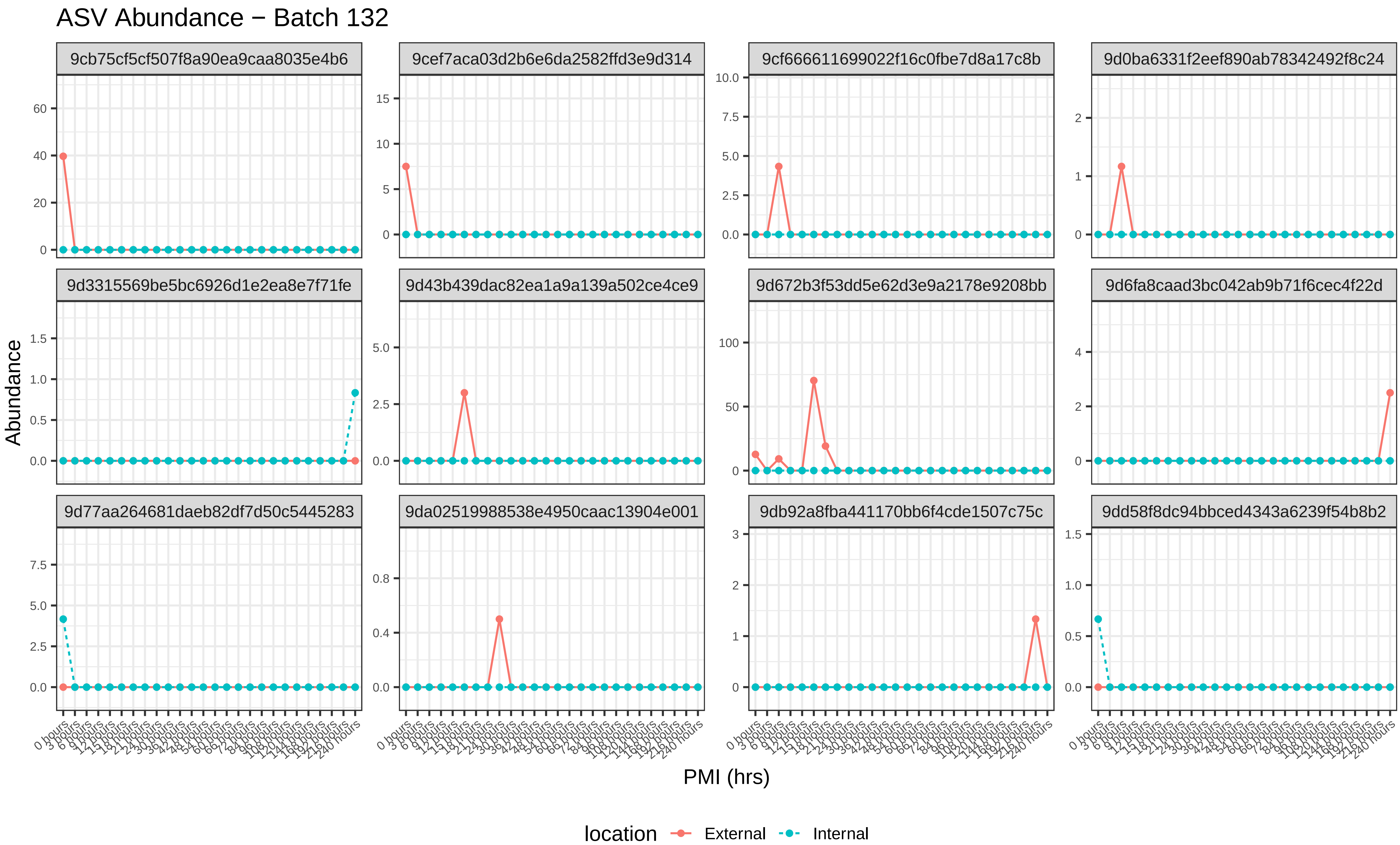
ASV Abundance − Batch 132
9cb75cf5cf507f8a90ea9caa8035e4b6
9cef7aca03d2b6e6da2582ffd3e9d314
9cf666611699022f16c0fbe7d8a17c8b
9d0ba6331f2eef890ab78342492f8c24
9d3315569be5bc6926d1e2ea8e7f71fe
9d43b439dac82ea1a9a139a502ce4ce9
9d672b3f53dd5e62d3e9a2178e9208bb
9d6fa8caad3bc042ab9b71f6cec4f22d
9d77aa264681daeb82df7d50c5445283
9da02519988538e4950caac13904e001
9db92a8fba441170bb6f4cde1507c75c
9dd58f8dc94bbced4343a6239f54b8b2
Abundance
PMI (hrs)
location
External
Internal

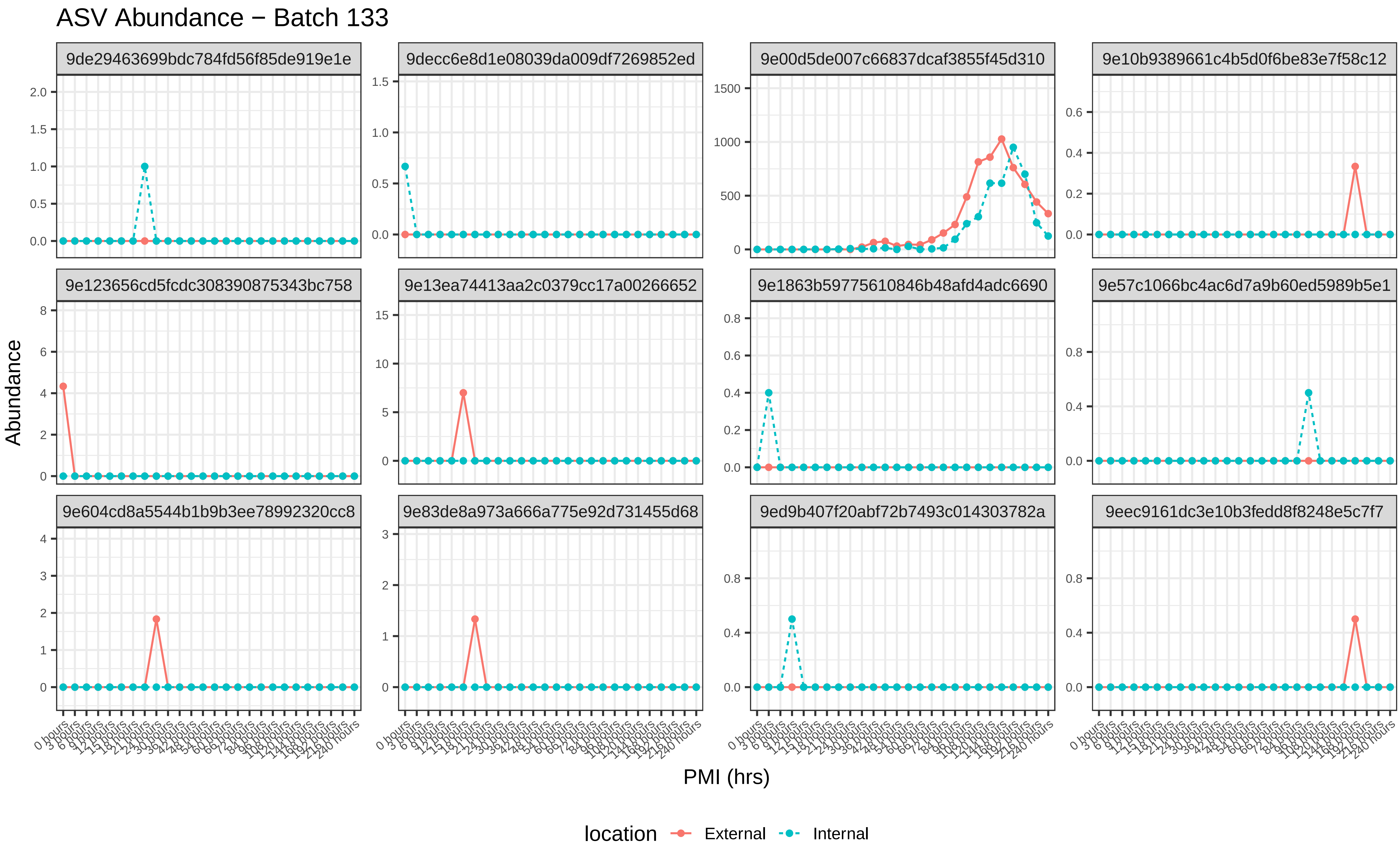

ASV Abundance − Batch 133
9de29463699bdc784fd56f85de919e1e
9decc6e8d1e08039da009df7269852ed
9e00d5de007c66837dcaf3855f45d310
9e10b9389661c4b5d0f6be83e7f58c12
9e123656cd5fcdc308390875343bc758
9e13ea74413aa2c0379cc17a00266652
9e1863b59775610846b48afd4adc6690
9e57c1066bc4ac6d7a9b60ed5989b5e1
9e604cd8a5544b1b9b3ee78992320cc8
9e83de8a973a666a775e92d731455d68
9ed9b407f20abf72b7493c014303782a
9eec9161dc3e10b3fedd8f8248e5c7f7
Abundance
PMI (hrs)
location
External
Internal

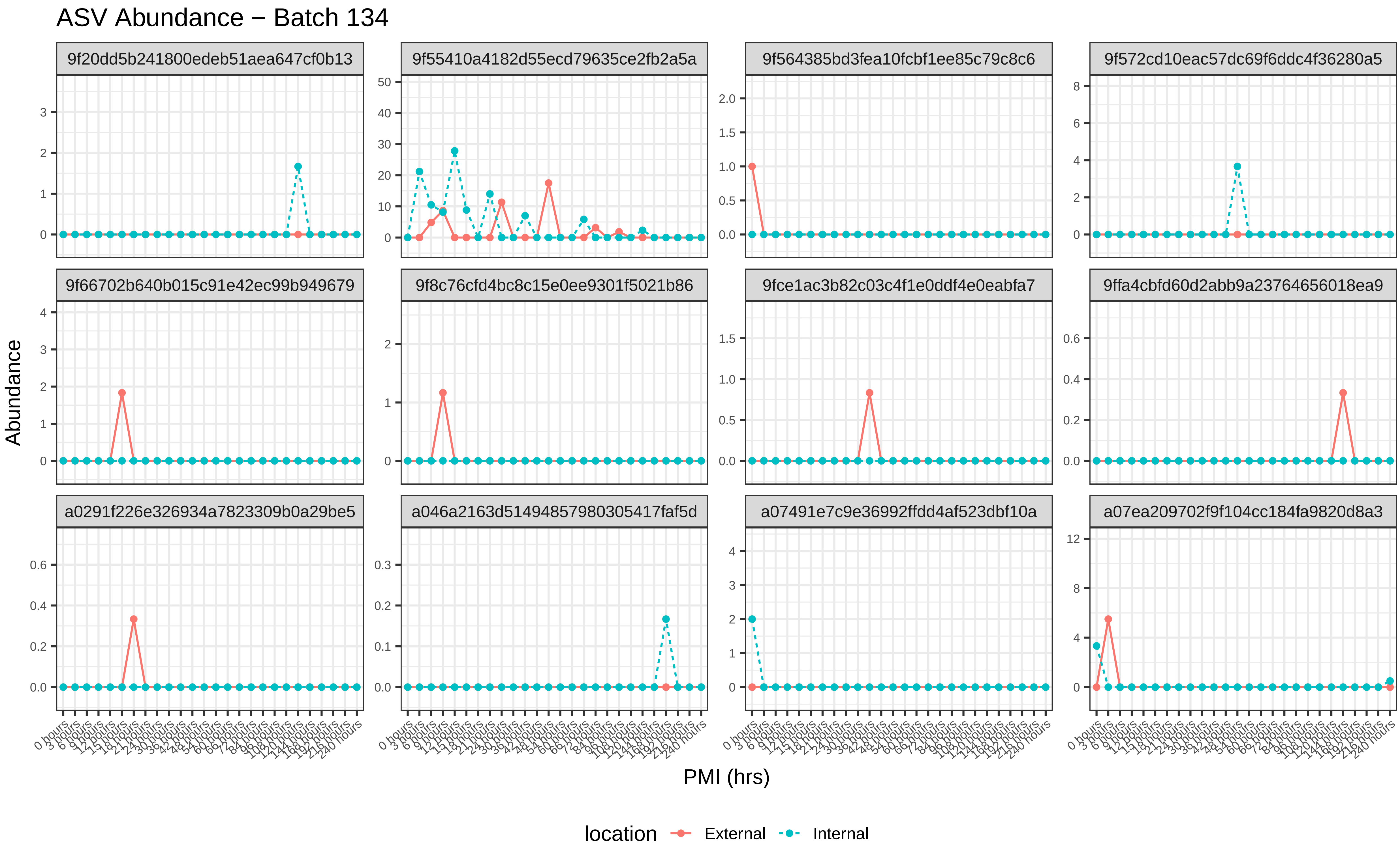
ASV Abundance − Batch 134
9f20dd5b241800edeb51aea647cf0b13
9f55410a4182d55ecd79635ce2fb2a5a
9f564385bd3fea10fcbf1ee85c79c8c6
9f572cd10eac57dc69f6ddc4f36280a5
9f66702b640b015c91e42ec99b949679
9f8c76cfd4bc8c15e0ee9301f5021b86
9fce1ac3b82c03c4f1e0ddf4e0eabfa7
9ffa4cbfd60d2abb9a23764656018ea9
a0291f226e326934a7823309b0a29be5
a046a2163d51494857980305417faf5d
a07491e7c9e36992ffdd4af523dbf10a
a07ea209702f9f104cc184fa9820d8a3
Abundance
PMI (hrs)
location
External
Internal

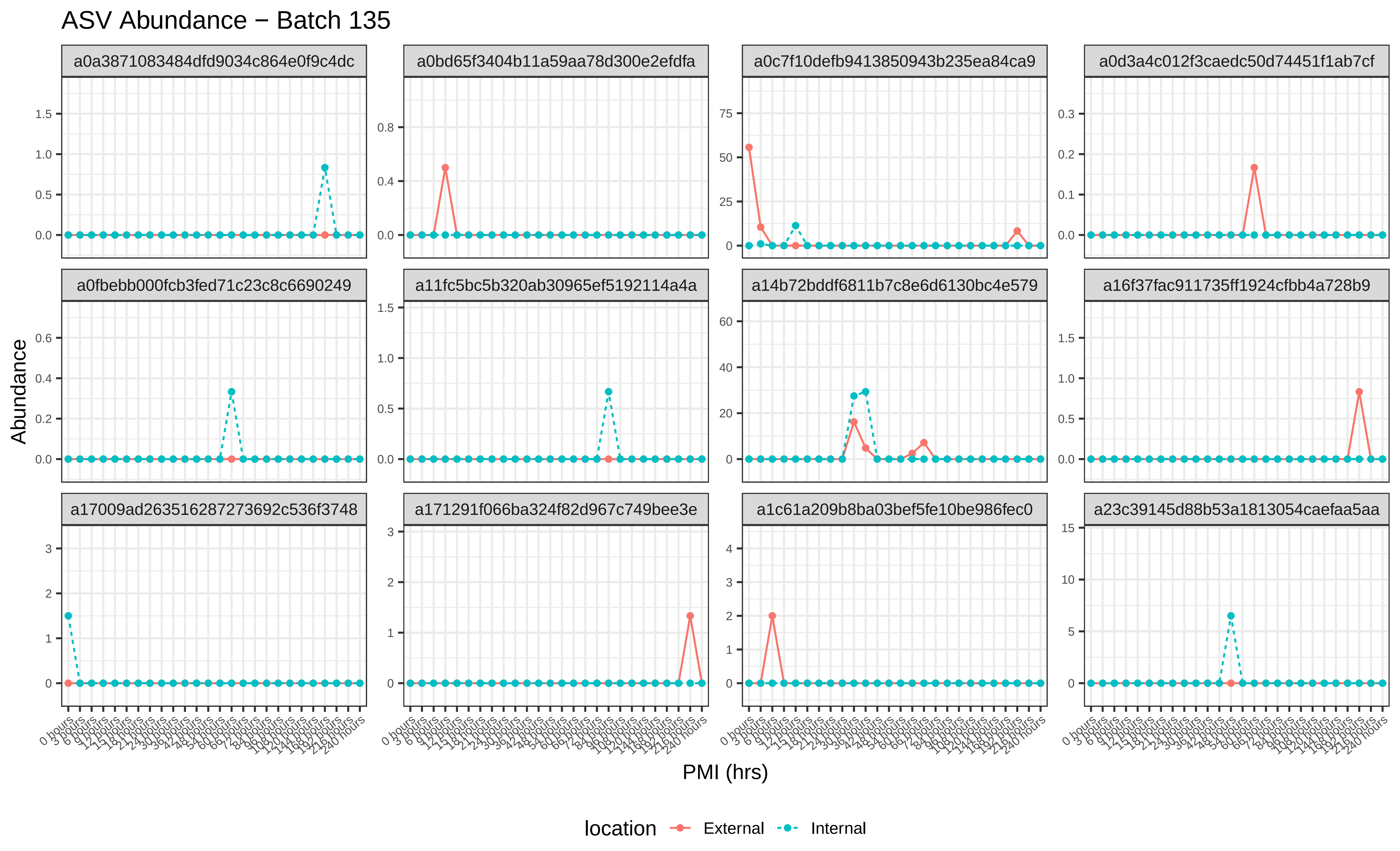
ASV Abundance − Batch 135
a0a3871083484dfd9034c864e0f9c4dc
a0bd65f3404b11a59aa78d300e2efdfa
a0c7f10defb9413850943b235ea84ca9
a0d3a4c012f3caedc50d74451f1ab7cf
a0fbebb000fcb3fed71c23c8c6690249
a11fc5bc5b320ab30965ef5192114a4a
a14b72bddf6811b7c8e6d6130bc4e579
a16f37fac911735ff1924cfbb4a728b9
a17009ad263516287273692c536f3748
a171291f066ba324f82d967c749bee3e
a1c61a209b8ba03bef5fe10be986fec0
a23c39145d88b53a1813054caefaa5aa
Abundance
PMI (hrs)
location
External
Internal

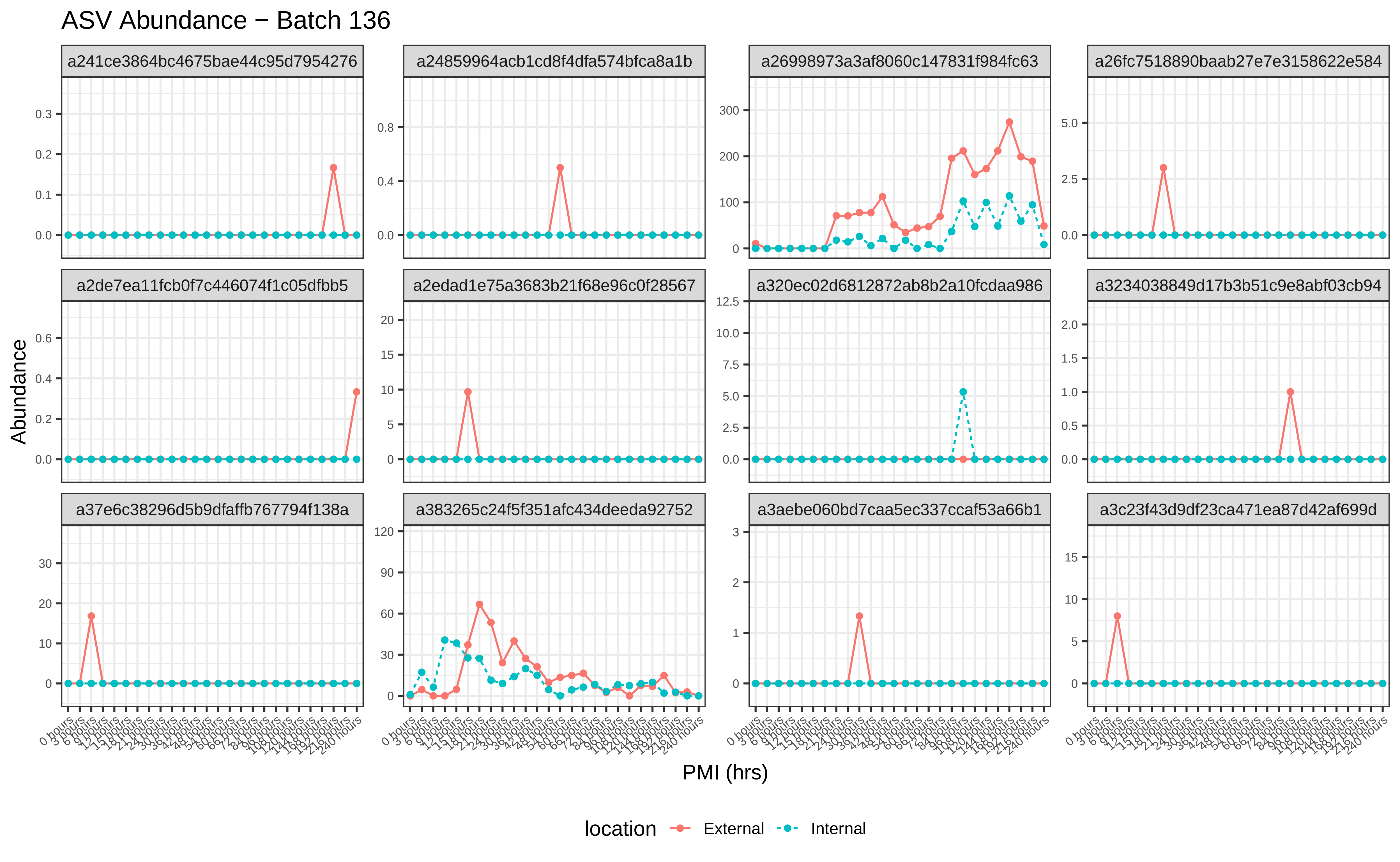
ASV Abundance − Batch 136
a241ce3864bc4675bae44c95d7954276
a24859964acb1cd8f4dfa574bfca8a1b
a26998973a3af8060c147831f984fc63
a26fc7518890baab27e7e3158622e584
a2de7ea11fcb0f7c446074f1c05dfbb5
a2edad1e75a3683b21f68e96c0f28567
a320ec02d6812872ab8b2a10fcdaa986
a3234038849d17b3b51c9e8abf03cb94
a37e6c38296d5b9dfaffb767794f138a
a383265c24f5f351afc434deeda92752
a3aebe060bd7caa5ec337ccaf53a66b1
a3c23f43d9df23ca471ea87d42af699d
Abundance
PMI (hrs)
location
External
Internal

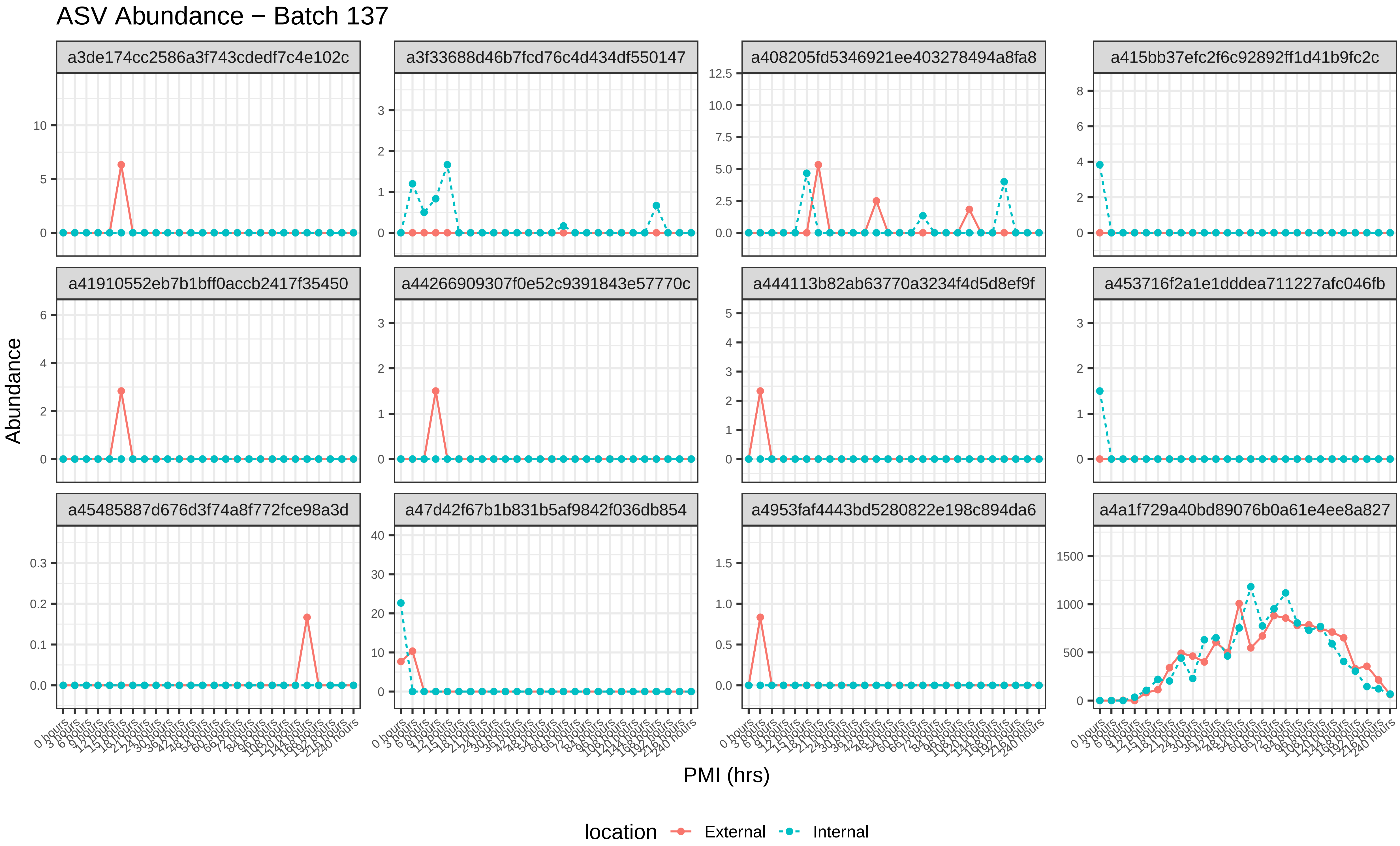
ASV Abundance − Batch 137
a3de174cc2586a3f743cdedf7c4e102c
a3f33688d46b7fcd76c4d434df550147
a408205fd5346921ee403278494a8fa8
a415bb37efc2f6c92892ff1d41b9fc2c
a41910552eb7b1bff0accb2417f35450
a44266909307f0e52c9391843e57770c
a444113b82ab63770a3234f4d5d8ef9f
a453716f2a1e1dddea711227afc046fb
a45485887d676d3f74a8f772fce98a3d
a47d42f67b1b831b5af9842f036db854
a4953faf4443bd5280822e198c894da6
a4a1f729a40bd89076b0a61e4ee8a827
Abundance
PMI (hrs)
location
External
Internal

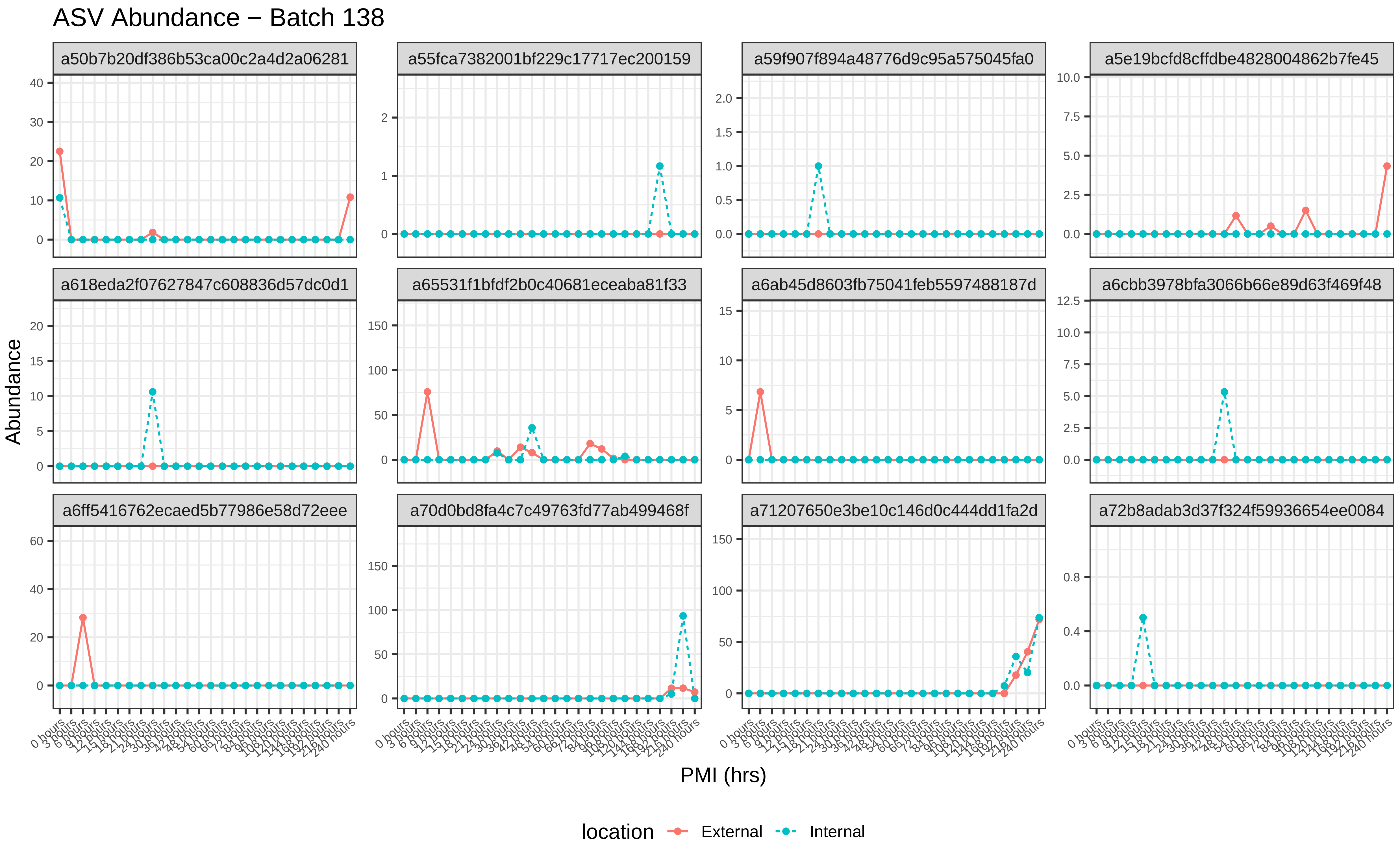

ASV Abundance − Batch 138
a50b7b20df386b53ca00c2a4d2a06281
a55fca7382001bf229c17717ec200159
a59f907f894a48776d9c95a575045fa0
a5e19bcfd8cffdbe4828004862b7fe45
a618eda2f07627847c608836d57dc0d1
a65531f1bfdf2b0c40681eceaba81f33
a6ab45d8603fb75041feb5597488187d
a6cbb3978bfa3066b66e89d63f469f48
a6ff5416762ecaed5b77986e58d72eee
a70d0bd8fa4c7c49763fd77ab499468f
a71207650e3be10c146d0c444dd1fa2d
a72b8adab3d37f324f59936654ee0084
Abundance
PMI (hrs)
location
External
Internal

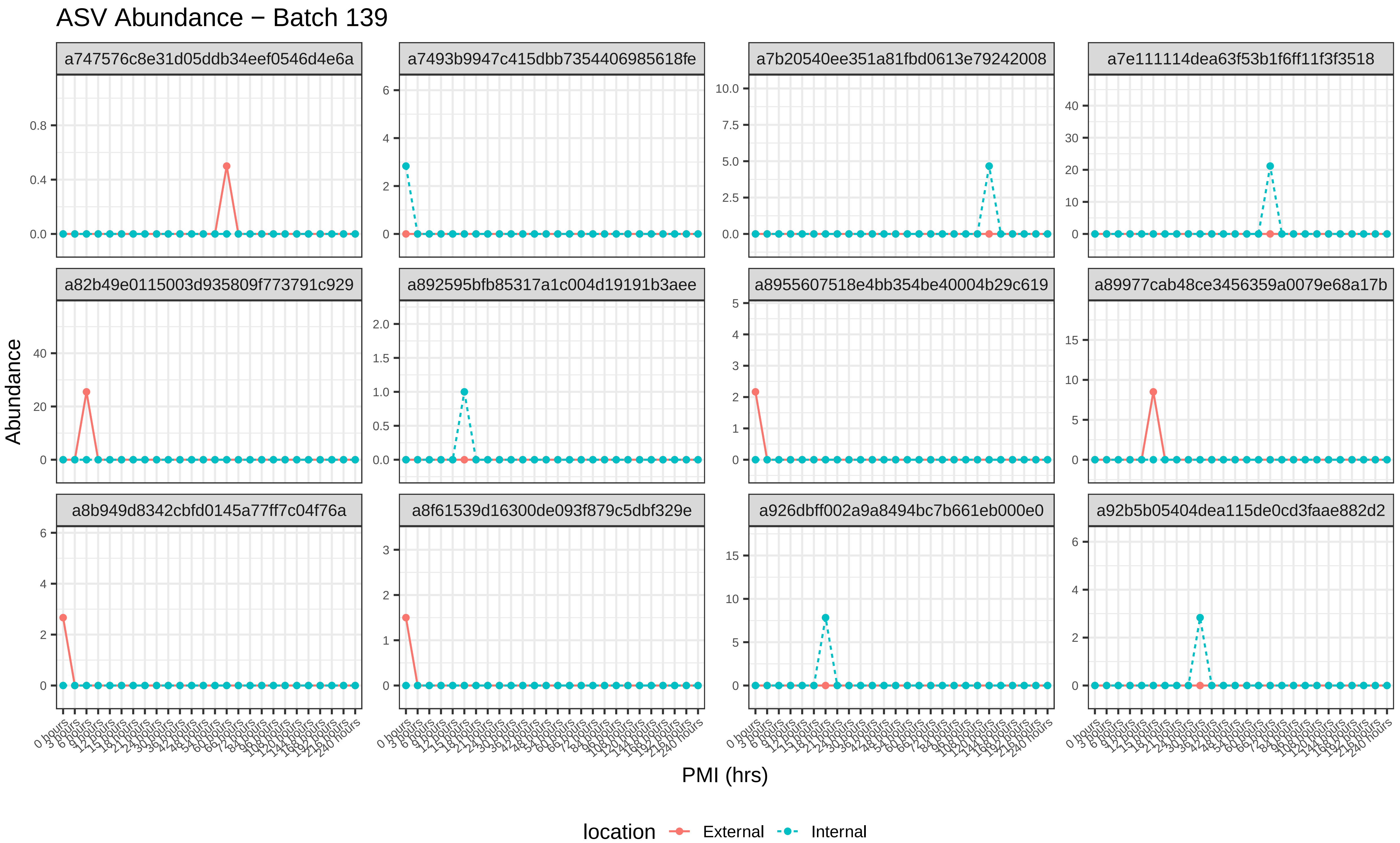

ASV Abundance − Batch 139
a747576c8e31d05ddb34eef0546d4e6a
a7493b9947c415dbb7354406985618fe
a7b20540ee351a81fbd0613e79242008
a7e111114dea63f53b1f6ff11f3f3518
a82b49e0115003d935809f773791c929
a892595bfb85317a1c004d19191b3aee
a8955607518e4bb354be40004b29c619
a89977cab48ce3456359a0079e68a17b
a8b949d8342cbfd0145a77ff7c04f76a
a8f61539d16300de093f879c5dbf329e
a926dbff002a9a8494bc7b661eb000e0
a92b5b05404dea115de0cd3faae882d2
Abundance
PMI (hrs)
location
External
Internal

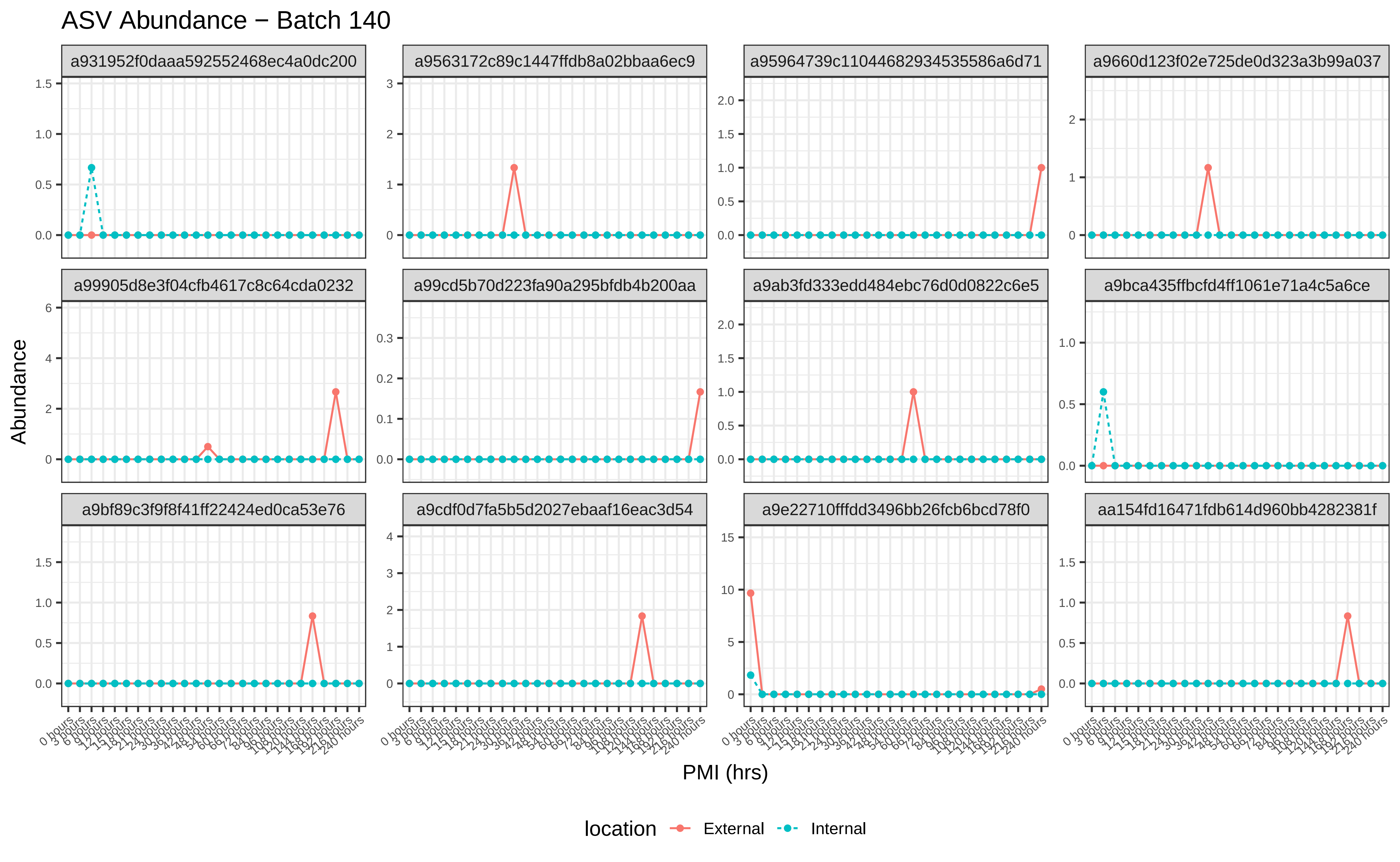
ASV Abundance − Batch 140
a931952f0daaa592552468ec4a0dc200
a9563172c89c1447ffdb8a02bbaa6ec9
a95964739c1104468293453558 6a6d71
a9660d123f02e725de0d323a3b99a037
a99905d8e3f04cfb4617c8c64cda0232
a99cd5b70d223fa90a295bfdb4b200aa
a9ab3fd333edd484ebc76d0d0822c6e5
a9bca435ffbcfd4ff1061e71a4c5a6ce
a9bf89c3f9f8f41ff22424ed0ca53e76
a9cdf0d7fa5b5d2027ebaaf16eac3d54
a9e22710fffdd3496bb26fcb6bcd78f0
aa154fd16471fdb614d960bb4282381f
Abundance
PMI (hrs)
location
External
Internal

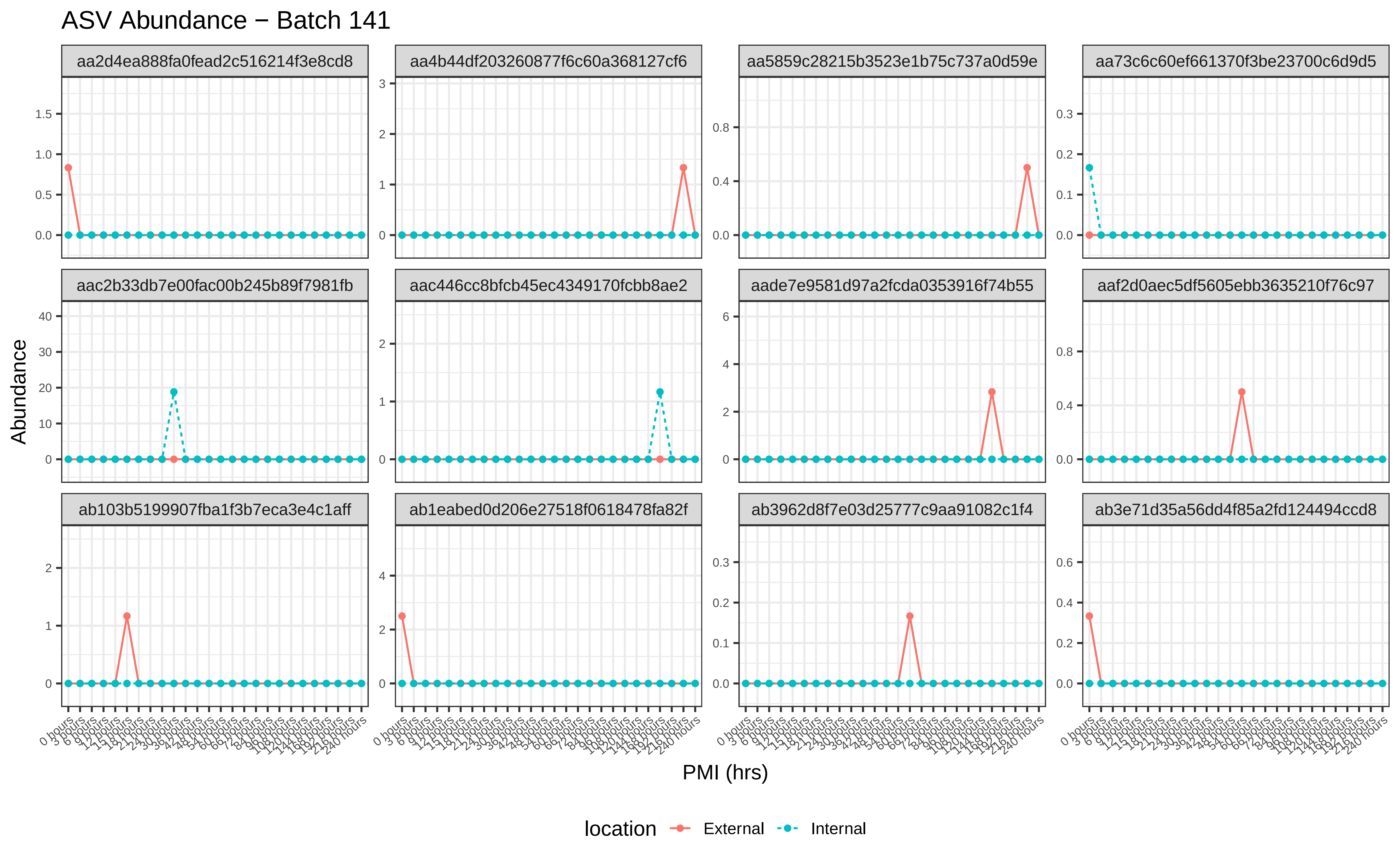
ASV Abundance − Batch 141
aa2d4ea888fa0fead2c516214f3e8cd8
aa4b44df203260877f6c60a368127cf6
aa5859c28215b3523e1b75c737a0d59e
aa73c6c60ef661370f3be23700c6d9d5
aac2b33db7e00fac00b245b89f7981fb
aac446cc8bfcb45ec4349170fcbb8ae2
aade7e9581d97a2fcda0353916f74b55
aaf2d0aec5df5605ebb3635210f76c97
ab103b5199907fba1f3b7eca3e4c1aff
ab1eabed0d206e27518f0618478fa82f
ab3962d8f7e03d25777c9aa91082c1f4
ab3e71d35a56dd4f85a2fd124494ccd8
Abundance
PMI (hrs)
location
External
Internal

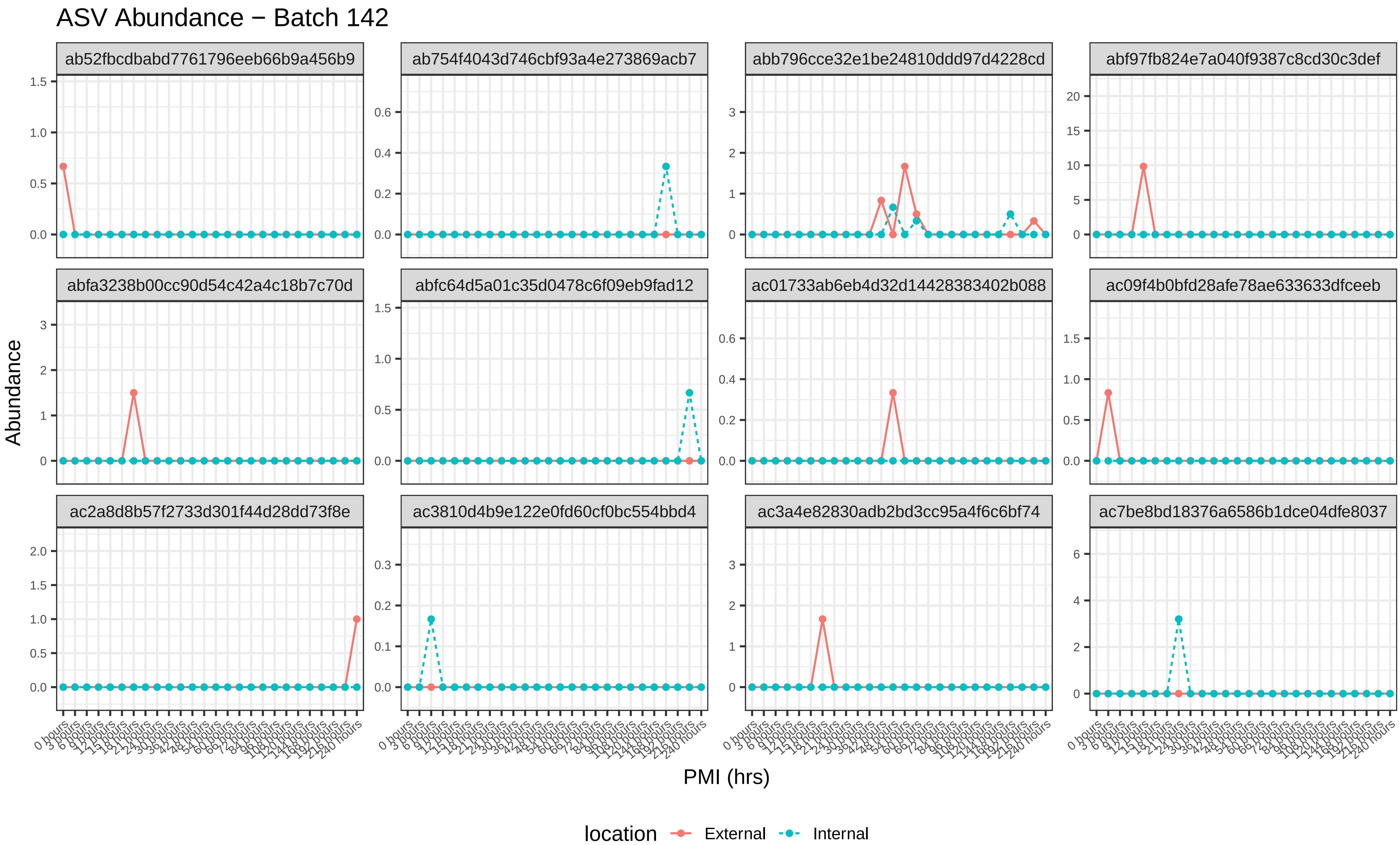
ASV Abundance − Batch 142
ab52fbcdbabd7761796eeb66b9a456b9
ab754f4043d746cbf93a4e273869acb7
abb796cce32e1be24810ddd97d4228cd
abf97fb824e7a040f9387c8cd30c3def
abfa3238b00cc90d54c42a4c18b7c70d
abfc64d5a01c35d0478c6f09eb9fad12
ac01733ab6eb4d32d14428383402b088
ac09f4b0bfd28afe78ae633633dfceeb
ac2a8d8b57f2733d301f44d28dd73f8e
ac3810d4b9e122e0fd60cf0bc554bbd4
ac3a4e82830adb2bd3cc95a4f6c6bf74
ac7be8bd18376a6586b1dce04dfe8037
Abundance
PMI (hrs)
location
External
Internal

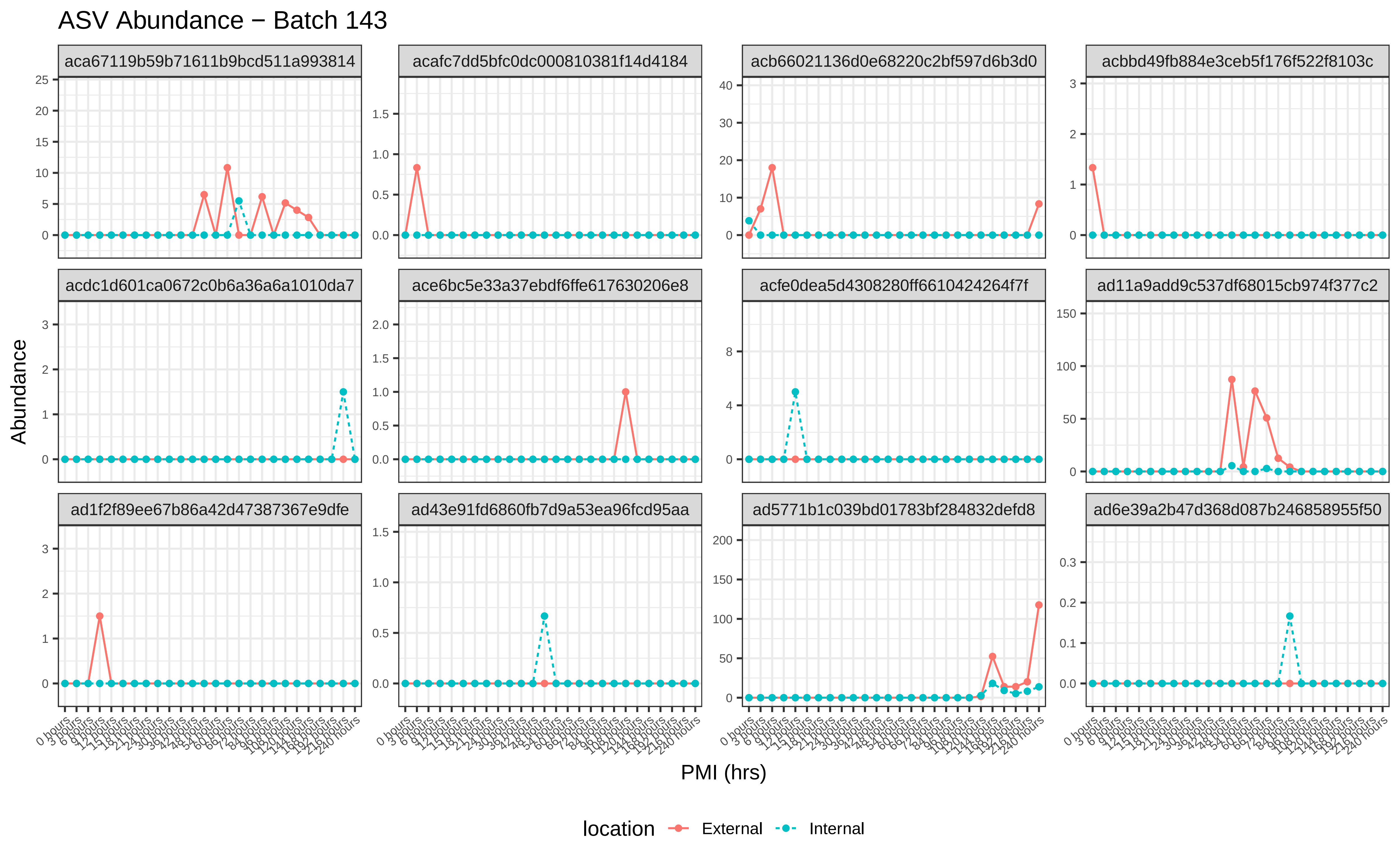

ASV Abundance − Batch 143
aca67119b59b71611b9bcd511a993814
acafc7dd5bfc0dc000810381f14d4184
acb66021136d0e68220c2bf597d6b3d0
acbbd49fb884e3ceb5f176f522f8103c
acdc1d601ca0672c0b6a36a6a1010da7
ace6bc5e33a37ebdf6ffe617630206e8
acfe0dea5d4308280ff6610424264f7f
ad11a9add9c537df68015cb974f377c2
ad1f2f89ee67b86a42d47387367e9dfe
ad43e91fd6860fb7d9a53ea96fcd95aa
ad5771b1c039bd01783bf284832defd8
ad6e39a2b47d368d087b246858955f50
Abundance
PMI (hrs)
location
External
Internal

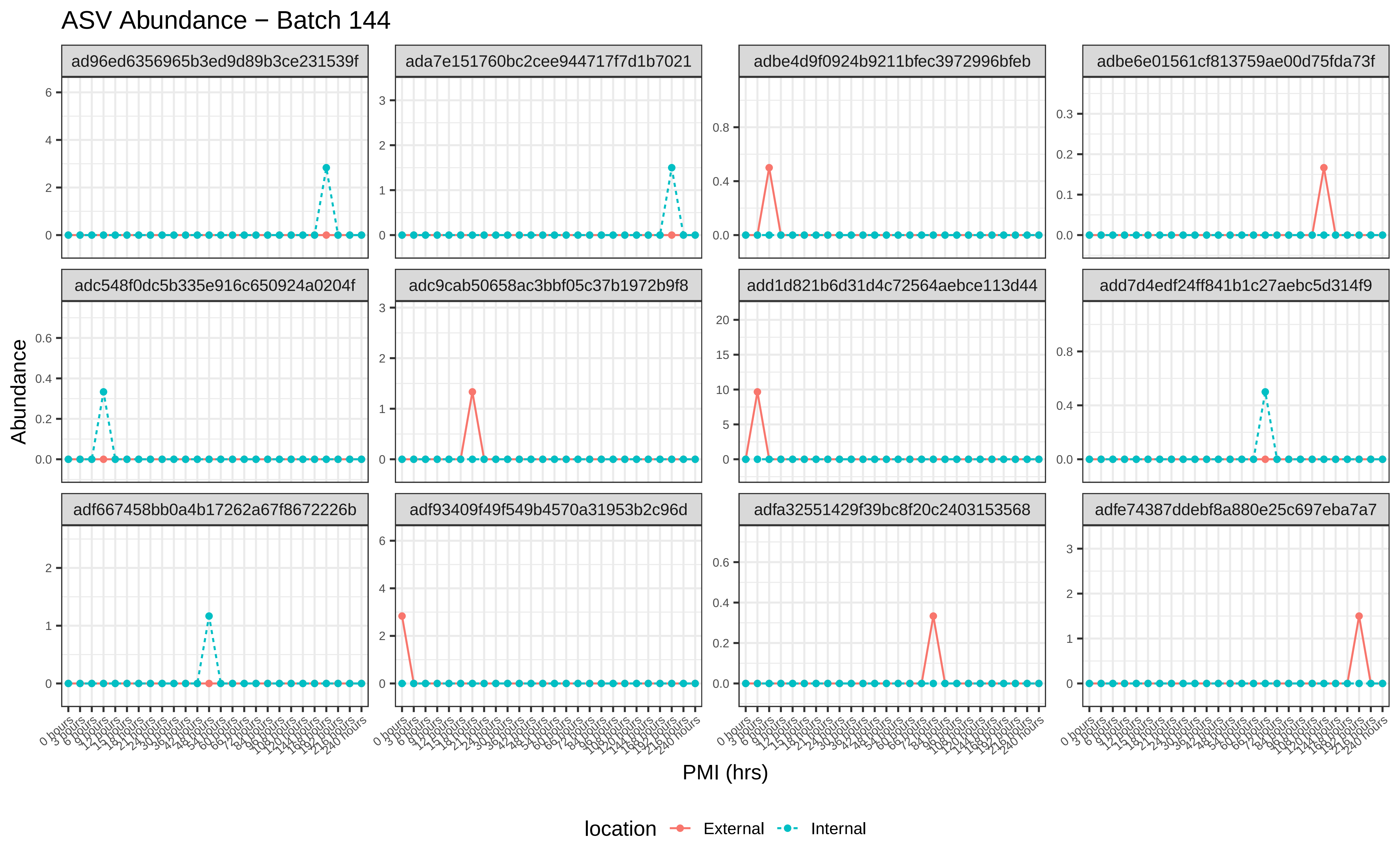

ASV Abundance − Batch 144
ad96ed6356965b3ed9d89b3ce231539f
ada7e151760bc2cee944717f7d1b7021
adbe4d9f0924b9211bfec3972996bfeb
adbe6e01561cf813759ae00d75fda73f
adc548f0dc5b335e916c650924a0204f
adc9cab50658ac3bbf05c37b1972b9f8
add1d821b6d31d4c72564aebce113d44
add7d4edf24ff841b1c27aebc5d314f9
adf667458bb0a4b17262a67f8672226b
adf93409f49f549b4570a31953b2c96d
adfa32551429f39bc8f20c2403153568
adfe74387ddebf8a880e25c697eba7a7
Abundance
PMI (hrs)
location
External
Internal

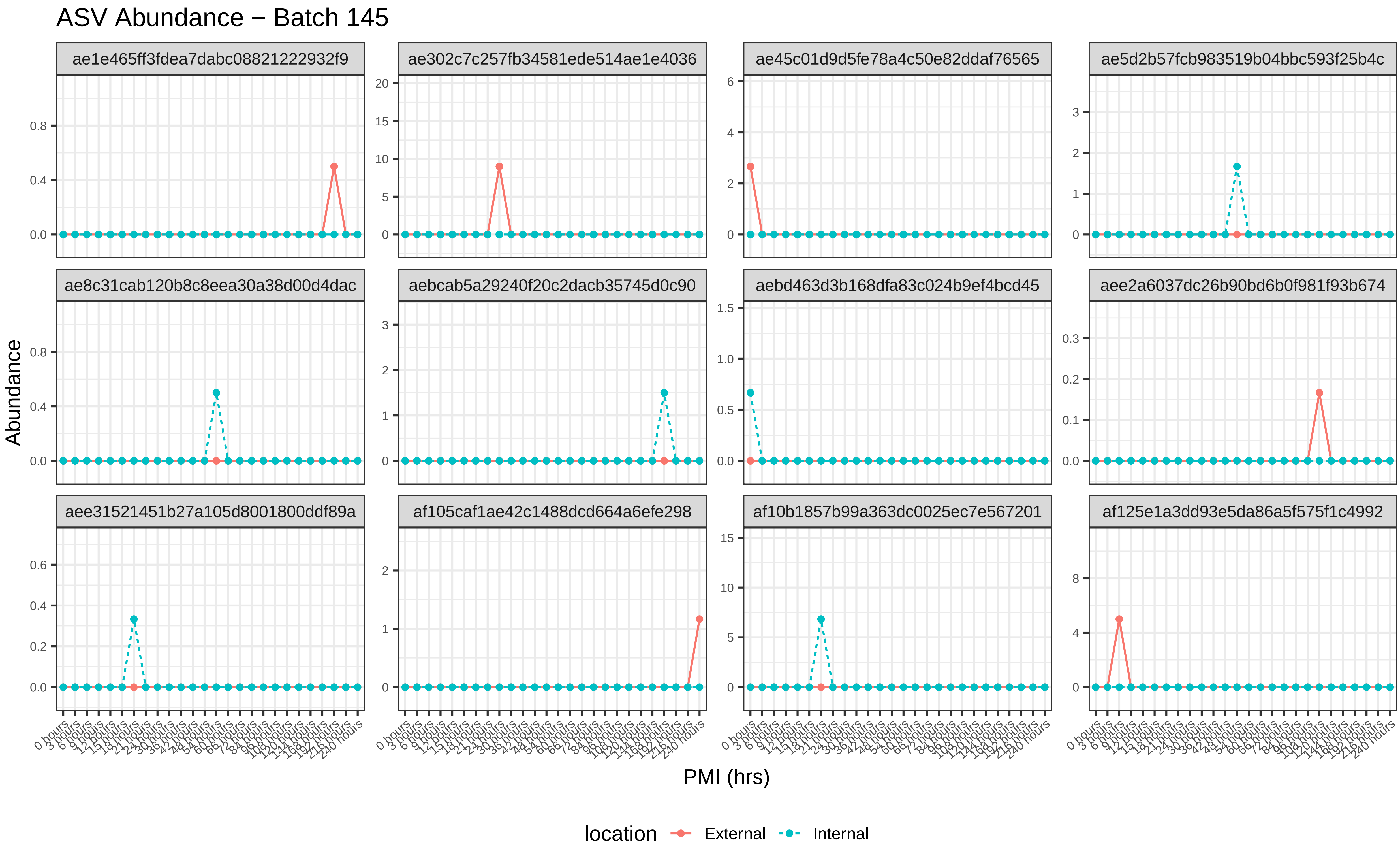

ASV Abundance − Batch 145
ae1e465ff3fdea7dabc08821222932f9
ae302c7c257fb34581ede514ae1e4036
ae45c01d9d5fe78a4c50e82ddaf76565
ae5d2b57fcb983519b04bbc593f25b4c
ae8c31cab120b8c8eea30a38d00d4dac
aebcab5a29240f20c2dacb35745d0c90
aebd463d3b168dfa83c024b9ef4bcd45
aee2a6037dc26b90bd6b0f981f93b674
aee31521451b27a105d8001800ddf89a
af105caf1ae42c1488dcd664a6efe298
af10b1857b99a363dc0025ec7e567201
af125e1a3dd93e5da86a5f575f1c4992
Abundance
PMI (hrs)
location
External
Internal

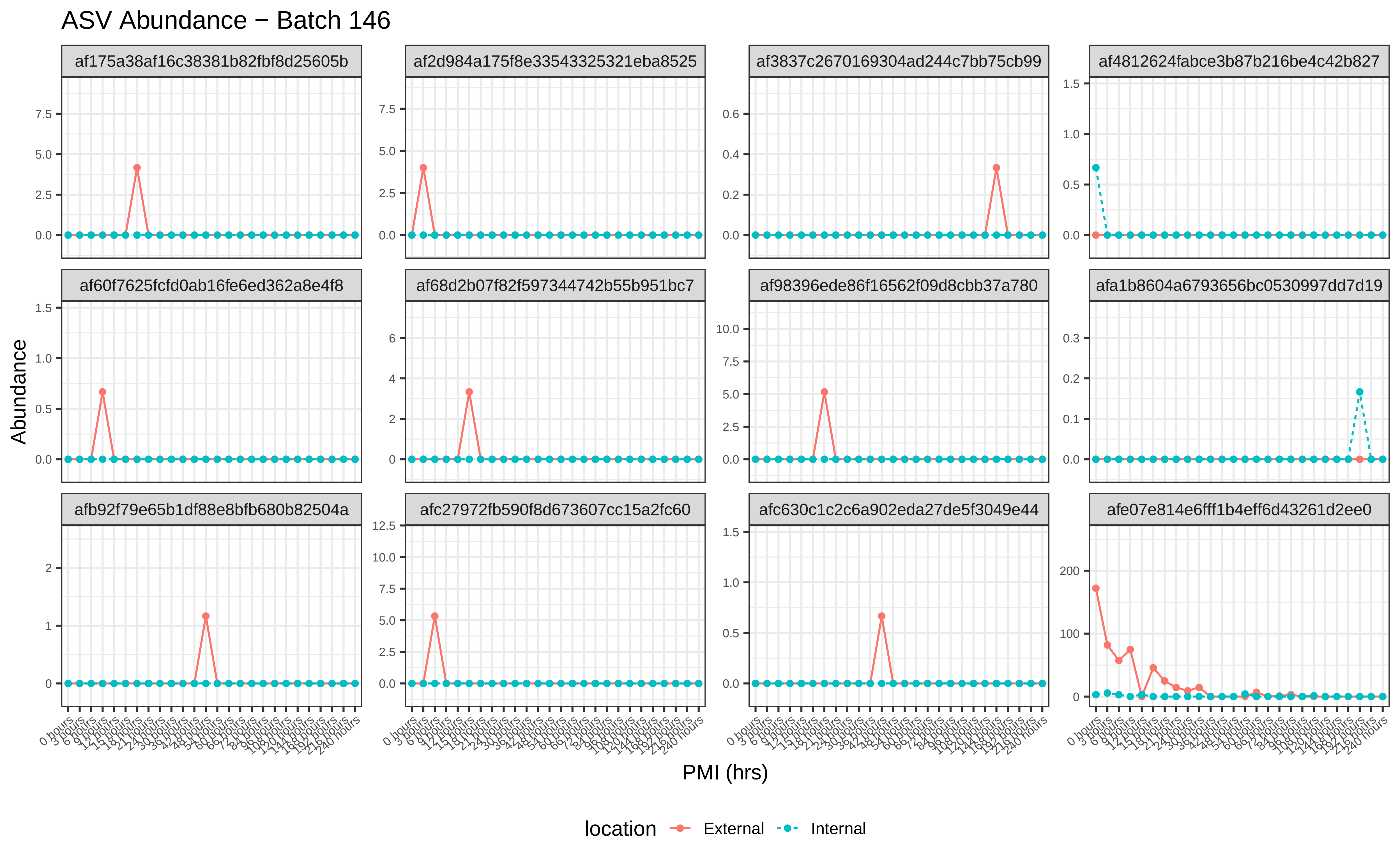
ASV Abundance − Batch 146
af175a38af16c38381b82fbf8d25605b
af2d984a175f8e33543325321eba8525
af3837c2670169304ad244c7bb75cb99
af4812624fabce3b87b216be4c42b827
af60f7625fcfd0ab16fe6ed362a8e4f8
af68d2b07f82f597344742b55b951bc7
af98396ede86f16562f09d8cbb37a780
afa1b8604a6793656bc0530997dd7d19
afb92f79e65b1df88e8bfb680b82504a
afc27972fb590f8d673607cc15a2fc60
afc630c1c2c6a902eda27de5f3049e44
afe07e814e6fff1b4eff6d43261d2ee0
Abundance
PMI (hrs)
location
External
Internal

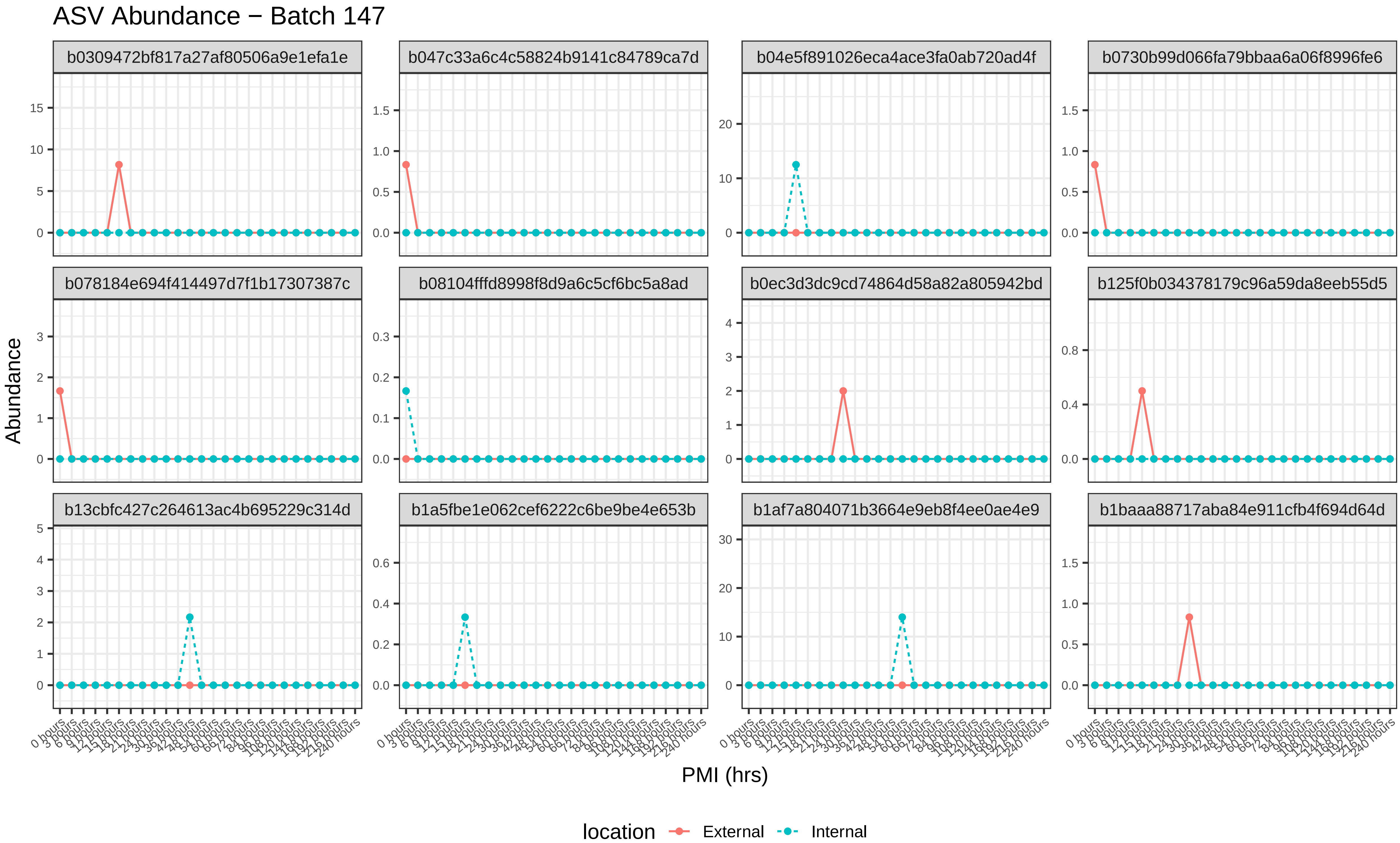
ASV Abundance − Batch 147
b0309472bf817a27af80506a9e1efa1e
b047c33a6c4c58824b9141c84789ca7d
b04e5f891026eca4ace3fa0ab720ad4f
b0730b99d066fa79bbaa6a06f8996fe6
b078184e694f414497d7f1b17307387c
b08104fffd8998f8d9a6c5cf6bc5a8ad
b0ec3d3dc9cd74864d58a82a805942bd
b125f0b034378179c96a59da8eeb55d5
b13cbfc427c264613ac4b695229c314d
b1a5fbe1e062cef6222c6be9be4e653b
b1af7a804071b3664e9eb8f4ee0ae4e9
b1baaa88717aba84e911cfb4f694d64d
Abundance
PMI (hrs)
location
External
Internal

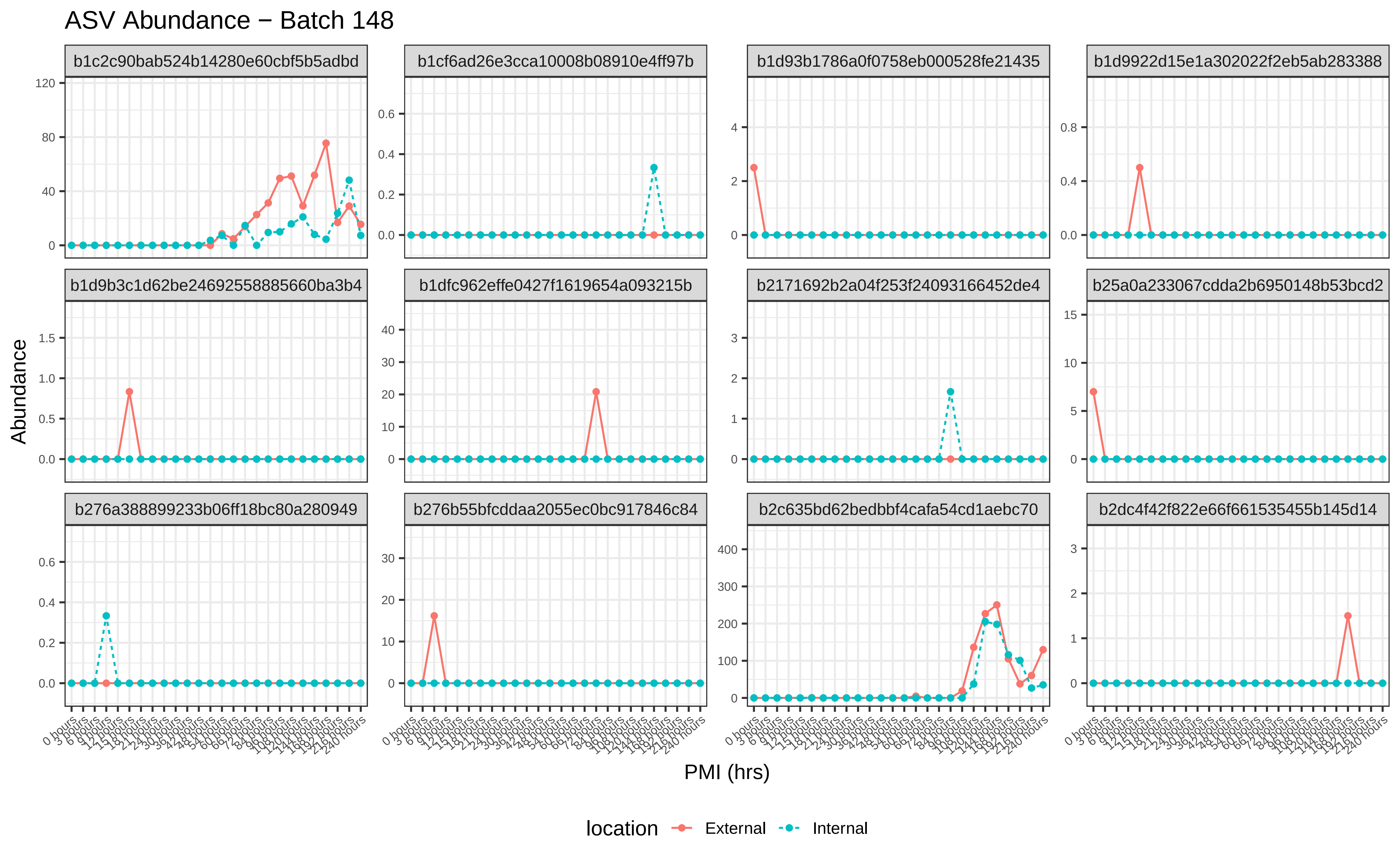

ASV Abundance − Batch 148
b1c2c90bab524b14280e60cbf5b5adbd
b1cf6ad26e3cca10008b08910e4ff97b
b1d93b1786a0f0758eb000528fe21435
b1d9922d15e1a302022f2eb5ab283388
b1d9b3c1d62be24692558885660ba3b4
b1dfc962effe0427f1619654a093215b
b2171692b2a04f253f24093166452de4
b25a0a233067cdda2b6950148b53bcd2
b276a388899233b06ff18bc80a280949
b276b55bfcddaa2055ec0bc917846c84
b2c635bd62bedbbf4cafa54cd1aebc70
b2dc4f42f822e66f661535455b145d14
Abundance
PMI (hrs)
location
External
Internal

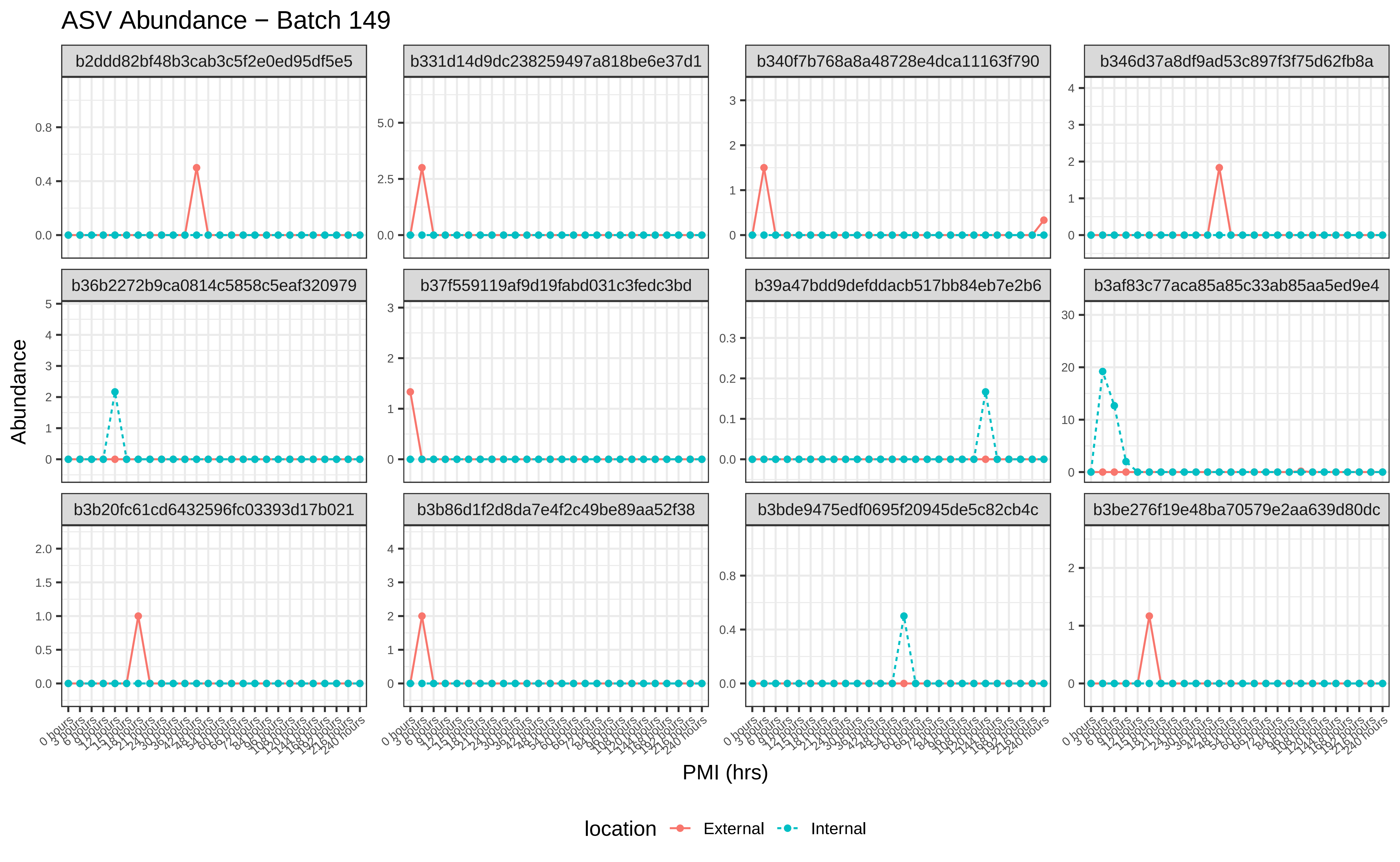
ASV Abundance − Batch 149
b2ddd82bf48b3cab3c5f2e0ed95df5e5
b331d14d9dc238259497a818be6e37d1
b340f7b768a8a48728e4dca11163f790
b346d37a8df9ad53c897f3f75d62fb8a
b36b2272b9ca0814c5858c5eaf320979
b37f559119af9d19fabd031c3fedc3bd
b39a47bdd9defddacb517bb84eb7e2b6
b3af83c77aca85a85c33ab85aa5ed9e4
b3b20fc61cd6432596fc03393d17b021
b3b86d1f2d8da7e4f2c49be89aa52f38
b3bde9475edf0695f20945de5c82cb4c
b3be276f19e48ba70579e2aa639d80dc
Abundance
PMI (hrs)
location
External
Internal

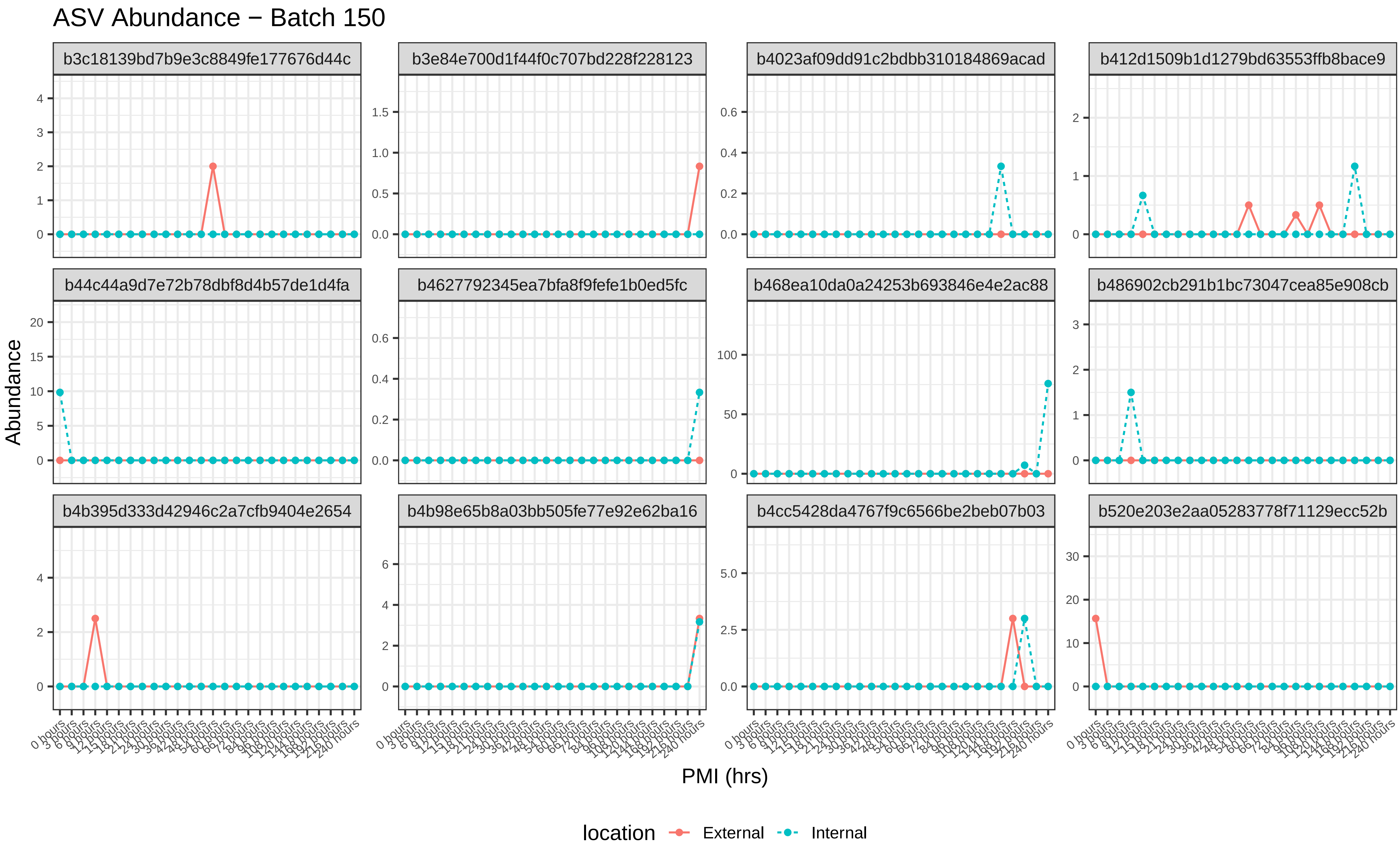
ASV Abundance − Batch 150
b3c18139bd7b9e3c8849fe177676d44c
b3e84e700d1f44f0c707bd228f228123
b4023af09dd91c2bdbb310184869acad
b412d1509b1d1279bd63553ffb8bace9
b44c44a9d7e72b78dbf8d4b57de1d4fa
b4627792345ea7bfa8f9fefe1b0ed5fc
b468ea10da0a24253b693846e4e2ac88
b486902cb291b1bc73047cea85e908cb
b4b395d333d42946c2a7cfb9404e2654
b4b98e65b8a03bb505fe77e92e62ba16
b4cc5428da4767f9c6566be2beb07b03
b520e203e2aa05283778f71129ecc52b
Abundance
PMI (hrs)
location
External
Internal

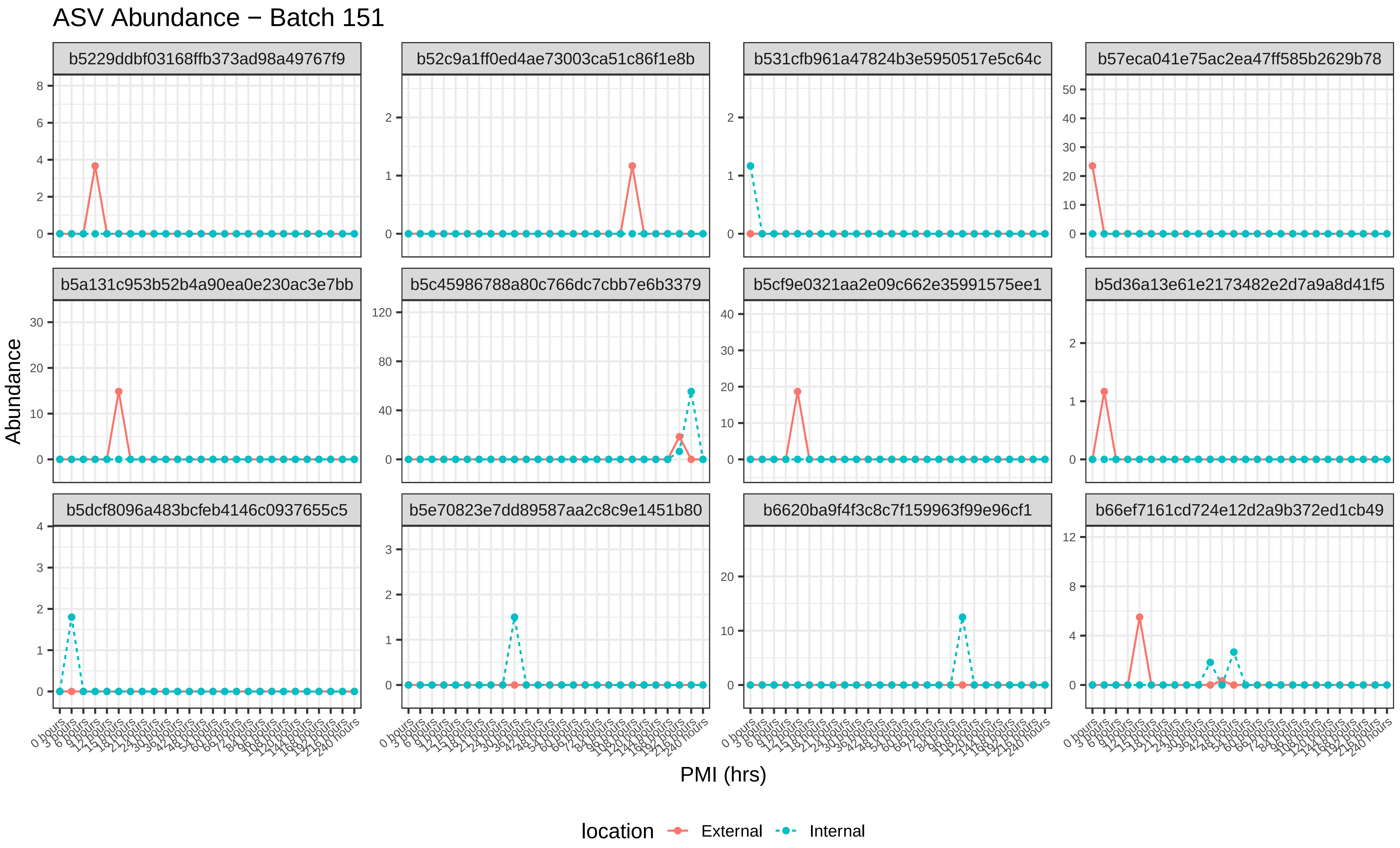
ASV Abundance − Batch 151
b5229ddbf03168ffb373ad98a49767f9
b52c9a1ff0ed4ae73003ca51c86f1e8b
b531cfb961a47824b3e5950517e5c64c
b57eca041e75ac2ea47ff585b2629b78
b5a131c953b52b4a90ea0e230ac3e7bb
b5c45986788a80c766dc7cbb7e6b3379
b5cf9e0321aa2e09c662e35991575ee1
b5d36a13e61e2173482e2d7a9a8d41f5
b5dcf8096a483bcfeb4146c0937655c5
b5e70823e7dd89587aa2c8c9e1451b80
b6620ba9f4f3c8c7f159963f99e96cf1
b66ef7161cd724e12d2a9b372ed1cb49
Abundance
PMI (hrs)
location
External
Internal

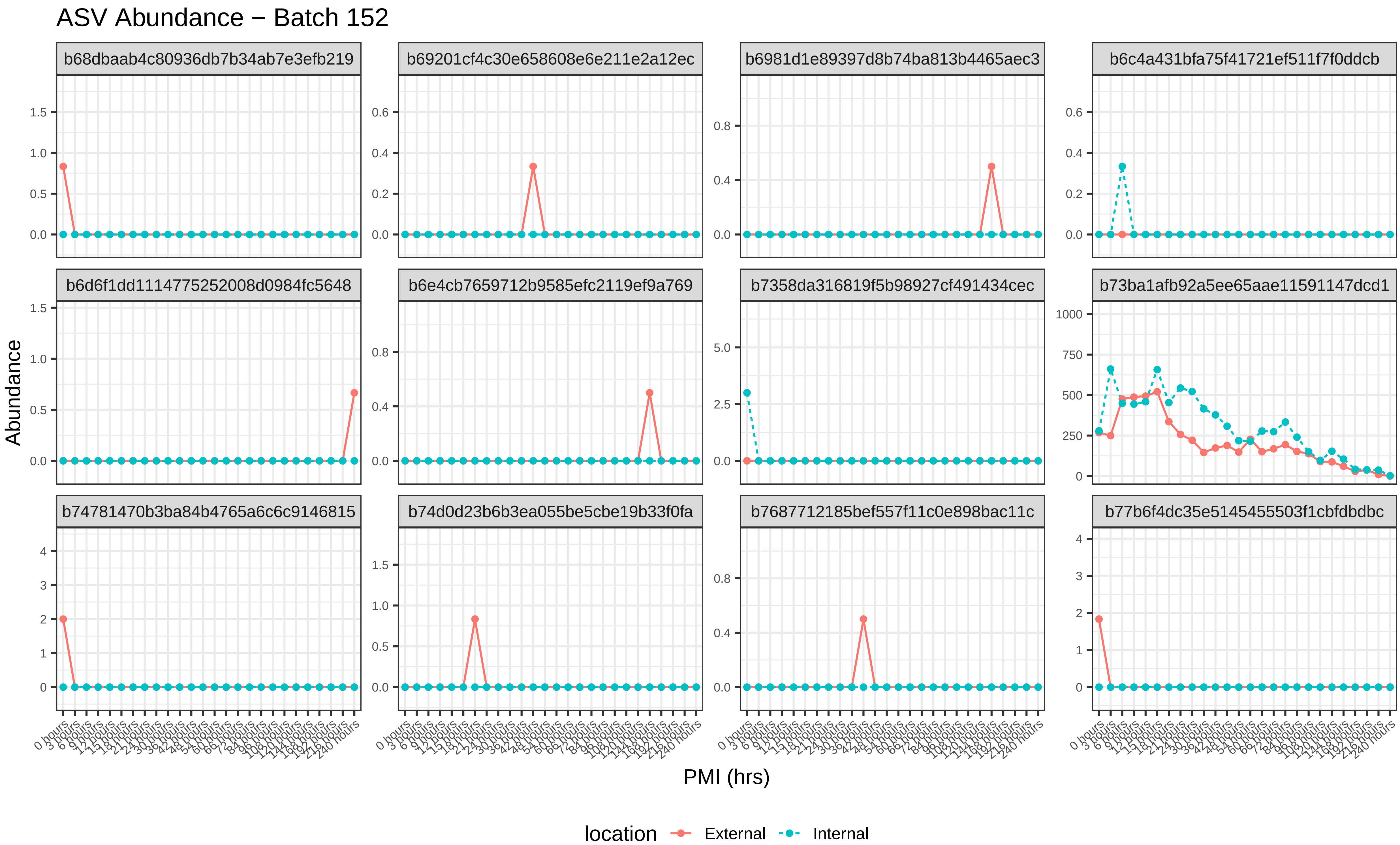
ASV Abundance − Batch 152
b68dbaab4c80936db7b34ab7e3efb219
b69201cf4c30e658608e6e211e2a12ec
b6981d1e89397d8b74ba813b4465aec3
b6c4a431bfa75f41721ef511f7f0ddcb
b6d6f1dd1114775252008d0984fc5648
b6e4cb7659712b9585efc2119ef9a769
b7358da316819f5b98927cf491434cec
b73ba1afb92a5ee65aae11591147dcd1
b74781470b3ba84b4765a6c6c9146815
b74d0d23b6b3ea055be5cbe19b33f0fa
b7687712185bef557f11c0e898bac11c
b77b6f4dc35e5145455503f1cbfdbdbc
Abundance
PMI (hrs)
location
External
Internal

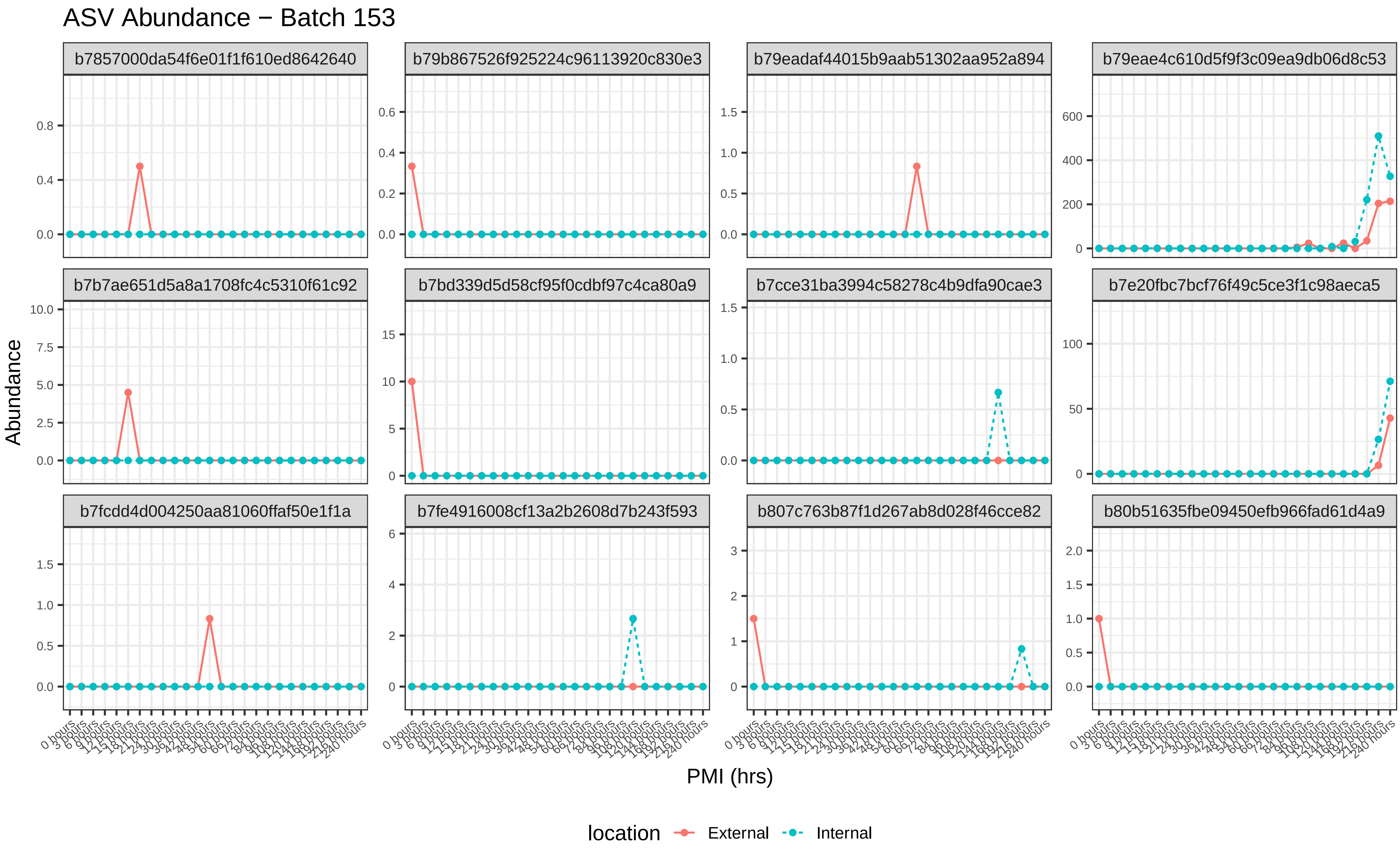
ASV Abundance − Batch 153
b7857000da54f6e01f1f610ed8642640
b79b867526f925224c96113920c830e3
b79eadaf44015b9aab51302aa952a894
b79eae4c610d5f9f3c09ea9db06d8c53
b7b7ae651d5a8a1708fc4c5310f61c92
b7bd339d5d58cf95f0cdbf97c4ca80a9
b7cce31ba3994c58278c4b9dfa90cae3
b7e20fbc7bcf76f49c5ce3f1c98aeca5
b7fcdd4d004250aa81060ffaf50e1f1a
b7fe4916008cf13a2b2608d7b243f593
b807c763b87f1d267ab8d028f46cce82
b80b51635fbe09450efb966fad61d4a9
Abundance
PMI (hrs)
location
External
Internal

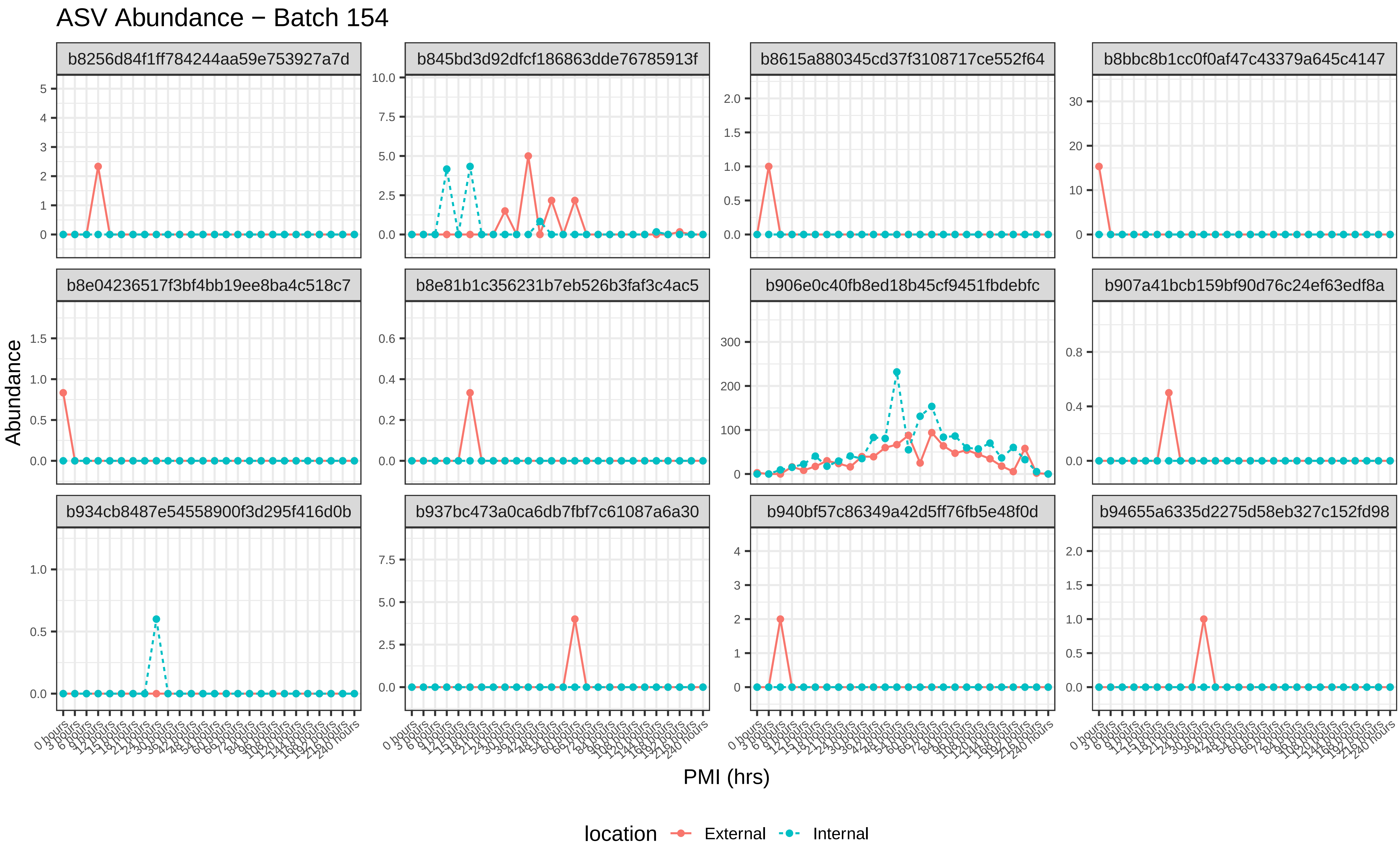

ASV Abundance − Batch 154
b8256d84f1ff784244aa59e753927a7d
b845bd3d92dfcf186863dde76785913f
b8615a880345cd37f3108717ce552f64
b8bbc8b1cc0f0af47c43379a645c4147
b8e04236517f3bf4bb19ee8ba4c518c7
b8e81b1c356231b7eb526b3faf3c4ac5
b906e0c40fb8ed18b45cf9451fbdebfc
b907a41bcb159bf90d76c24ef63edf8a
b934cb8487e54558900f3d295f416d0b
b937bc473a0ca6db7fbf7c61087a6a30
b940bf57c86349a42d5ff76fb5e48f0d
b94655a6335d2275d58eb327c152fd98
Abundance
PMI (hrs)
location
External
Internal

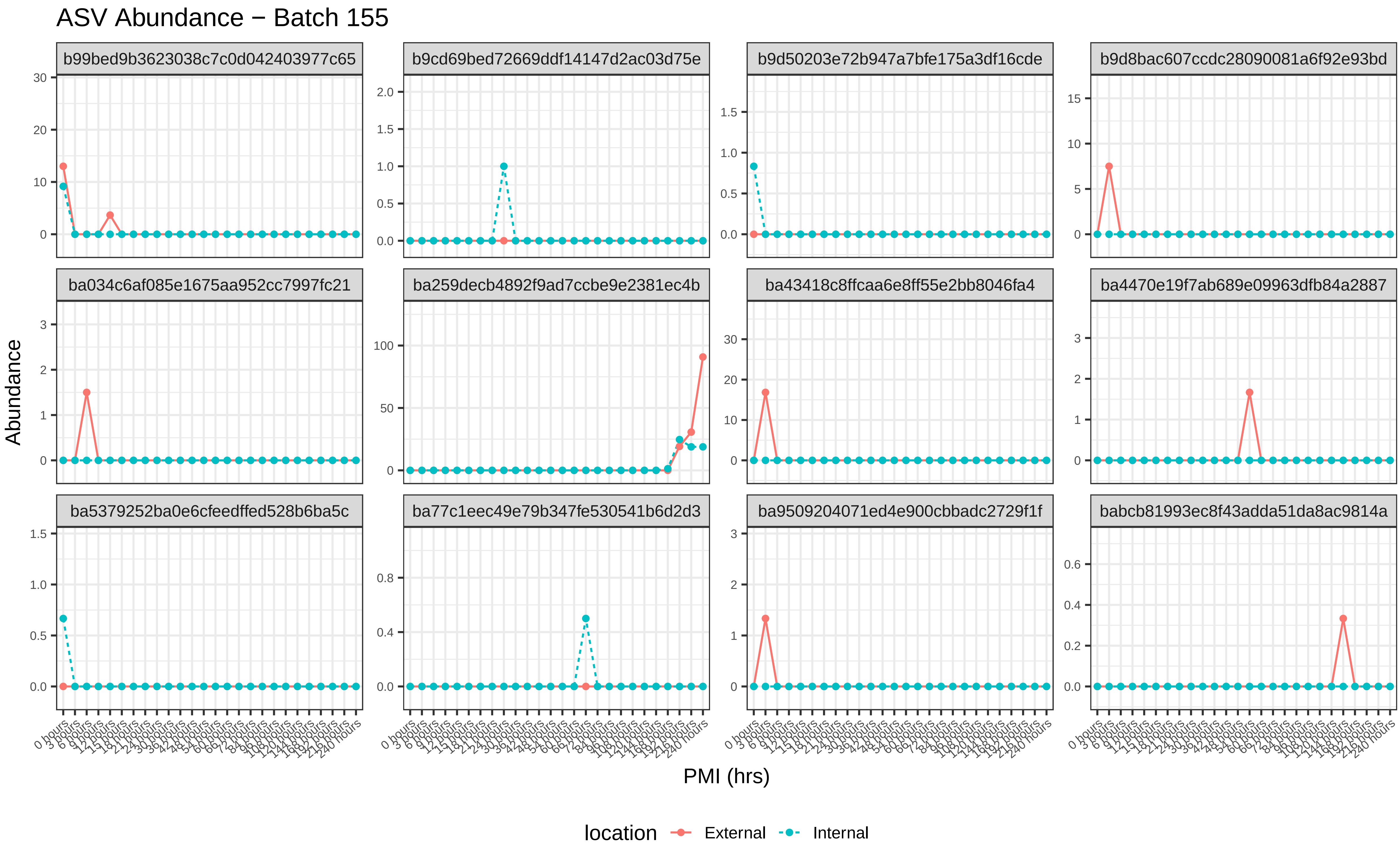

ASV Abundance − Batch 155
b99bed9b3623038c7c0d042403977c65
b9cd69bed72669ddf14147d2ac03d75e
b9d50203e72b947a7bfe175a3df16cde
b9d8bac607ccdc28090081a6f92e93bd
ba034c6af085e1675aa952cc7997fc21
ba259decb4892f9ad7ccbe9e2381ec4b
ba43418c8ffcaa6e8ff55e2bb8046fa4
ba4470e19f7ab689e09963dfb84a2887
ba5379252ba0e6cfeedffed528b6ba5c
ba77c1eec49e79b347fe530541b6d2d3
ba9509204071ed4e900cbbadc2729f1f
babcb81993ec8f43adda51da8ac9814a
Abundance
PMI (hrs)
location
External
Internal

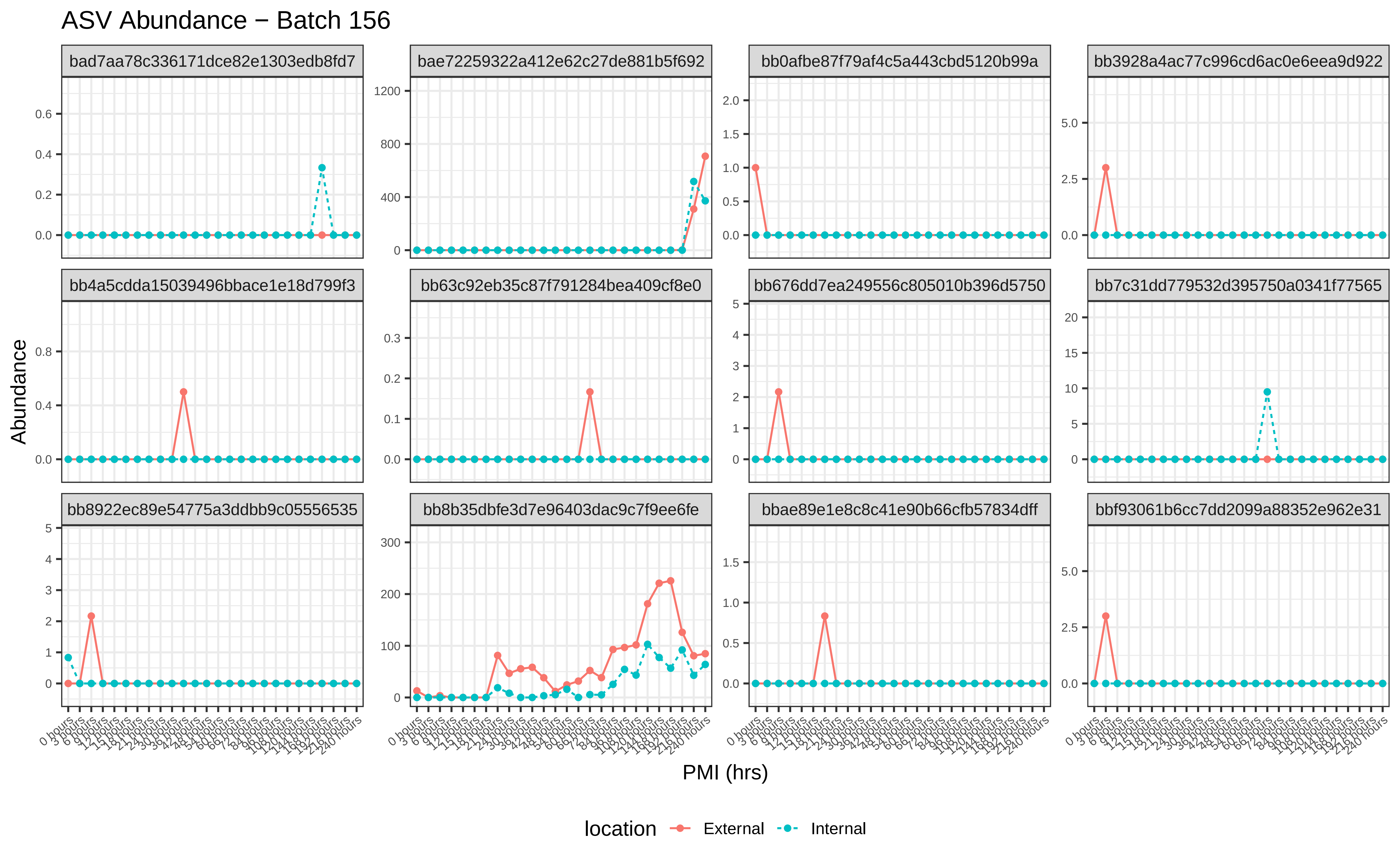
ASV Abundance − Batch 156
bad7aa78c336171dce82e1303edb8fd7
bae72259322a412e62c27de881b5f692
bb0afbe87f79af4c5a443cbd5120b99a
bb3928a4ac77c996cd6ac0e6eea9d922
bb4a5cdda15039496bbace1e18d799f3
bb63c92eb35c87f791284bea409cf8e0
bb676dd7ea249556c805010b396d5750
bb7c31dd779532d395750a0341f77565
bb8922ec89e54775a3ddbb9c05556535
bb8b35dbfe3d7e96403dac9c7f9ee6fe
bbae89e1e8c8c41e90b66cfb57834dff
bbf93061b6cc7dd2099a88352e962e31
Abundance
PMI (hrs)
location
External
Internal

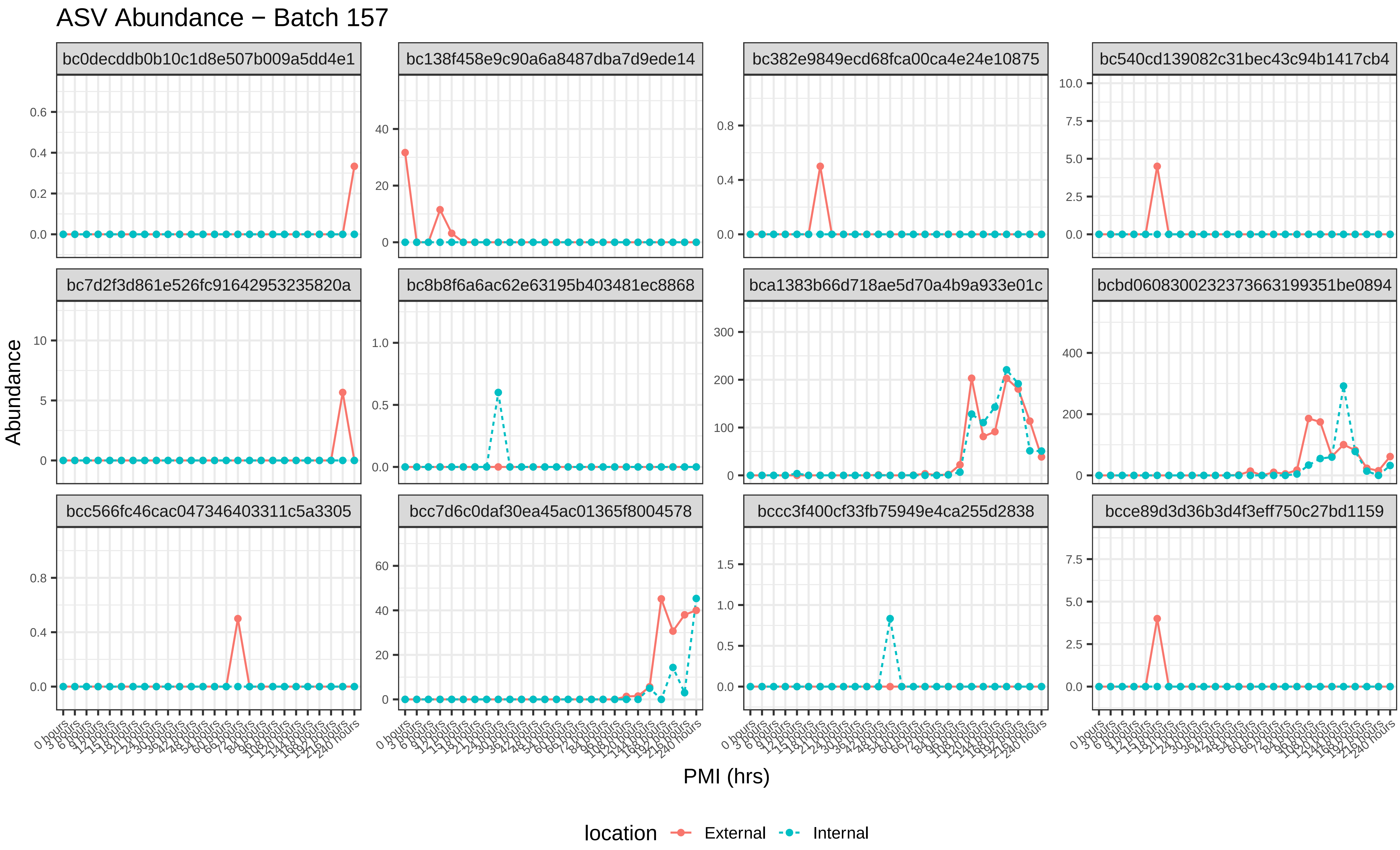
ASV Abundance − Batch 157
bc0decddb0b10c1d8e507b009a5dd4e1
bc138f458e9c90a6a8487dba7d9ede14
bc382e9849ecd68fca00ca4e24e10875
bc540cd139082c31bec43c94b1417cb4
bc7d2f3d861e526fc91642953235820a
bc8b8f6a6ac62e63195b403481ec8868
bca1383b66d718ae5d70a4b9a933e01c
bcbd0608300232373663199351be0894
bcc566fc46cac047346403311c5a3305
bcc7d6c0daf30ea45ac01365f8004578
bccc3f400cf33fb75949e4ca255d2838
bcce89d3d36b3d4f3eff750c27bd1159
Abundance
PMI (hrs)
location
External
Internal

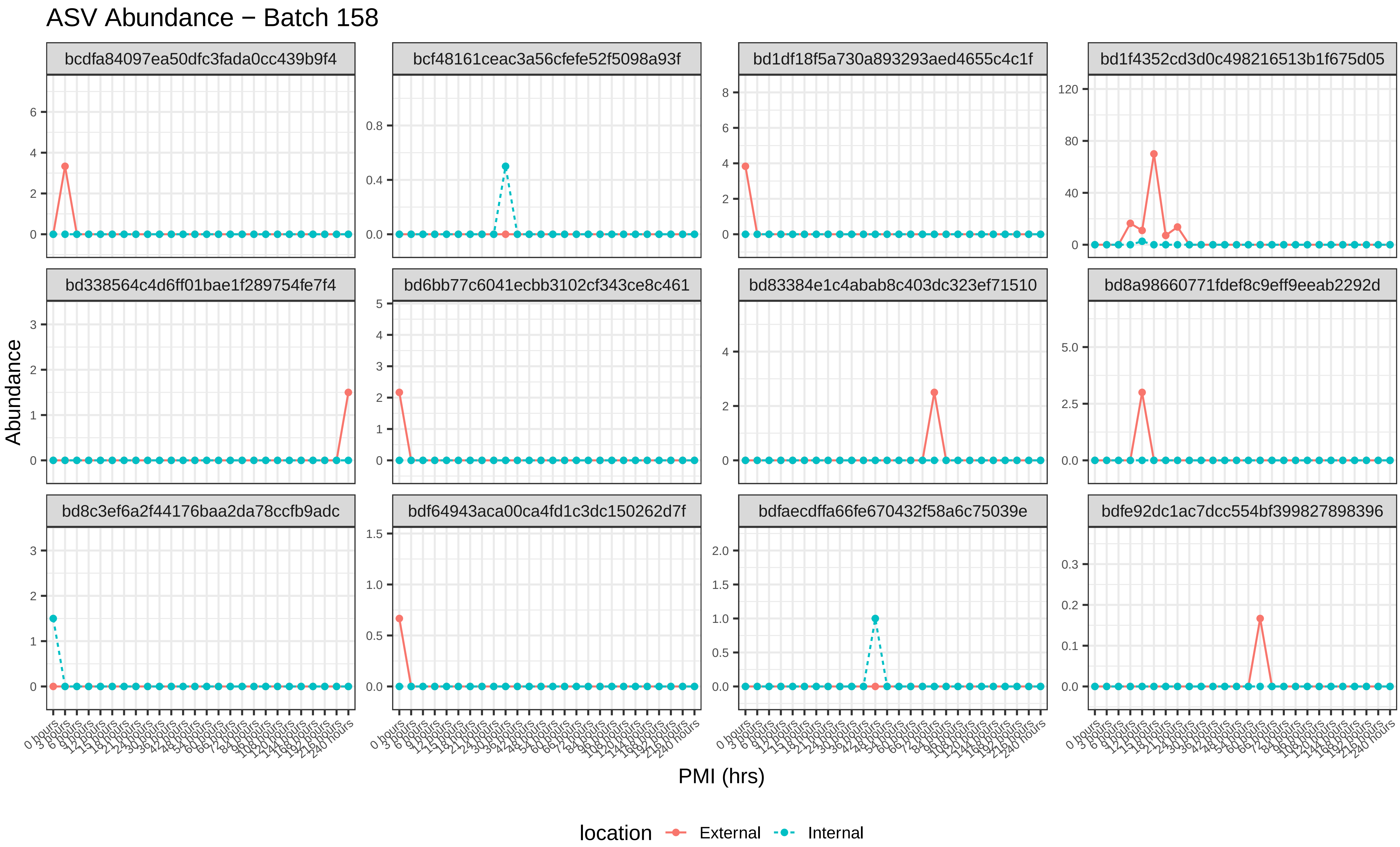
ASV Abundance − Batch 158
bcdfa84097ea50dfc3fada0cc439b9f4
bcf48161ceac3a56cfefe52f5098a93f
bd1df18f5a730a893293aed4655c4c1f
bd1f4352cd3d0c498216513b1f675d05
bd338564c4d6ff01bae1f289754fe7f4
bd6bb77c6041ecbb3102cf343ce8c461
bd83384e1c4abab8c403dc323ef71510
bd8a98660771fdef8c9eff9eeab2292d
bd8c3ef6a2f44176baa2da78ccfb9adc
bdf64943aca00ca4fd1c3dc150262d7f
bdfaecdffa66fe670432f58a6c75039e
bdfe92dc1ac7dcc554bf399827898396
Abundance
PMI (hrs)
location
External
Internal

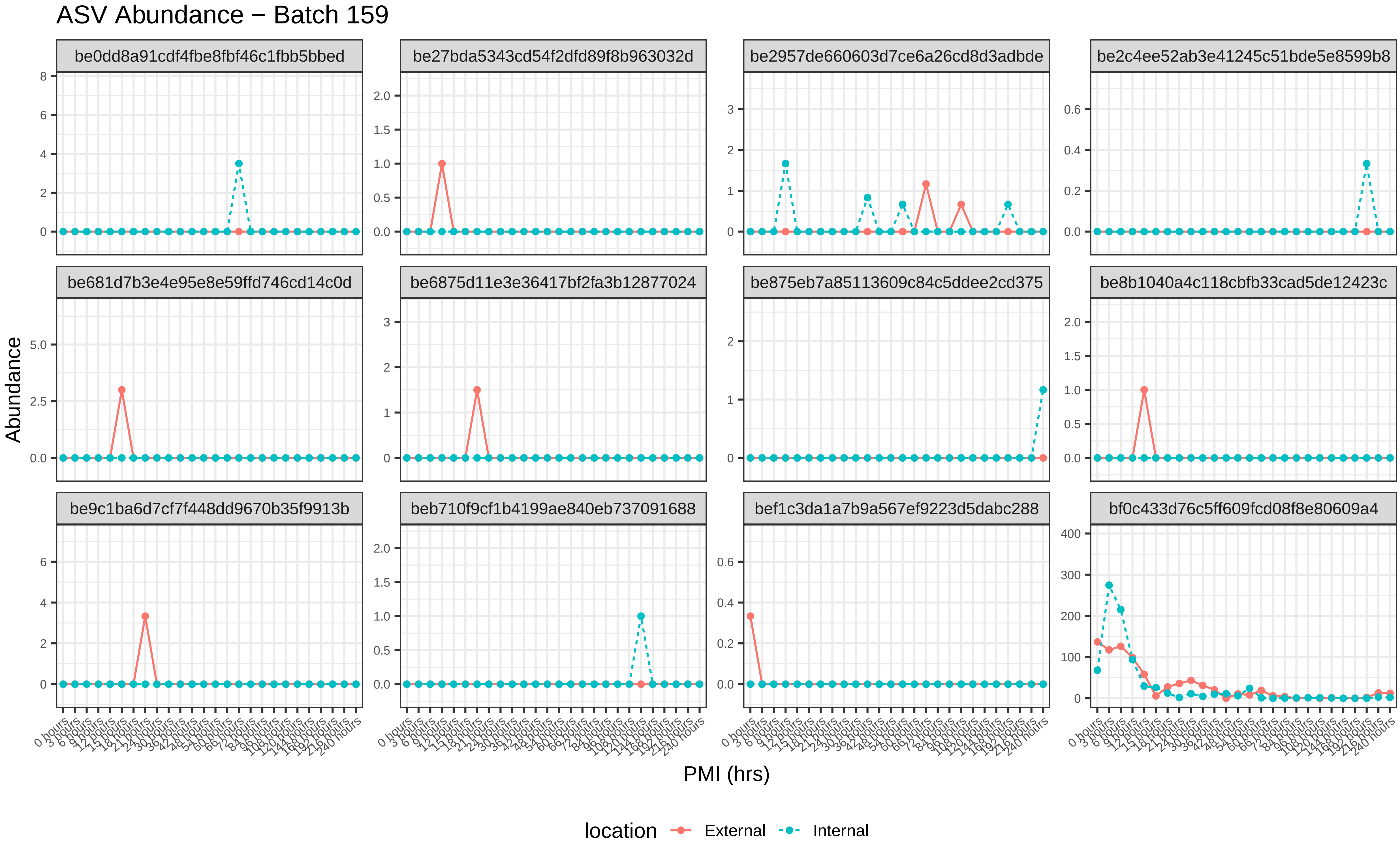
ASV Abundance − Batch 159
be0dd8a91cdf4fbe8fbf46c1fbb5bbed
be27bda5343cd54f2dfd89f8b963032d
be2957de660603d7ce6a26cd8d3adbde
be2c4ee52ab3e41245c51bde5e8599b8
be681d7b3e4e95e8e59ffd746cd14c0d
be6875d11e3e36417bf2fa3b12877024
be875eb7a85113609c84c5ddee2cd375
be8b1040a4c118cbfb33cad5de12423c
be9c1ba6d7cf7f448dd9670b35f9913b
beb710f9cf1b4199ae840eb737091688
bef1c3da1a7b9a567ef9223d5dabc288
bf0c433d76c5ff609fcd08f8e80609a4
Abundance
PMI (hrs)
location
External
Internal

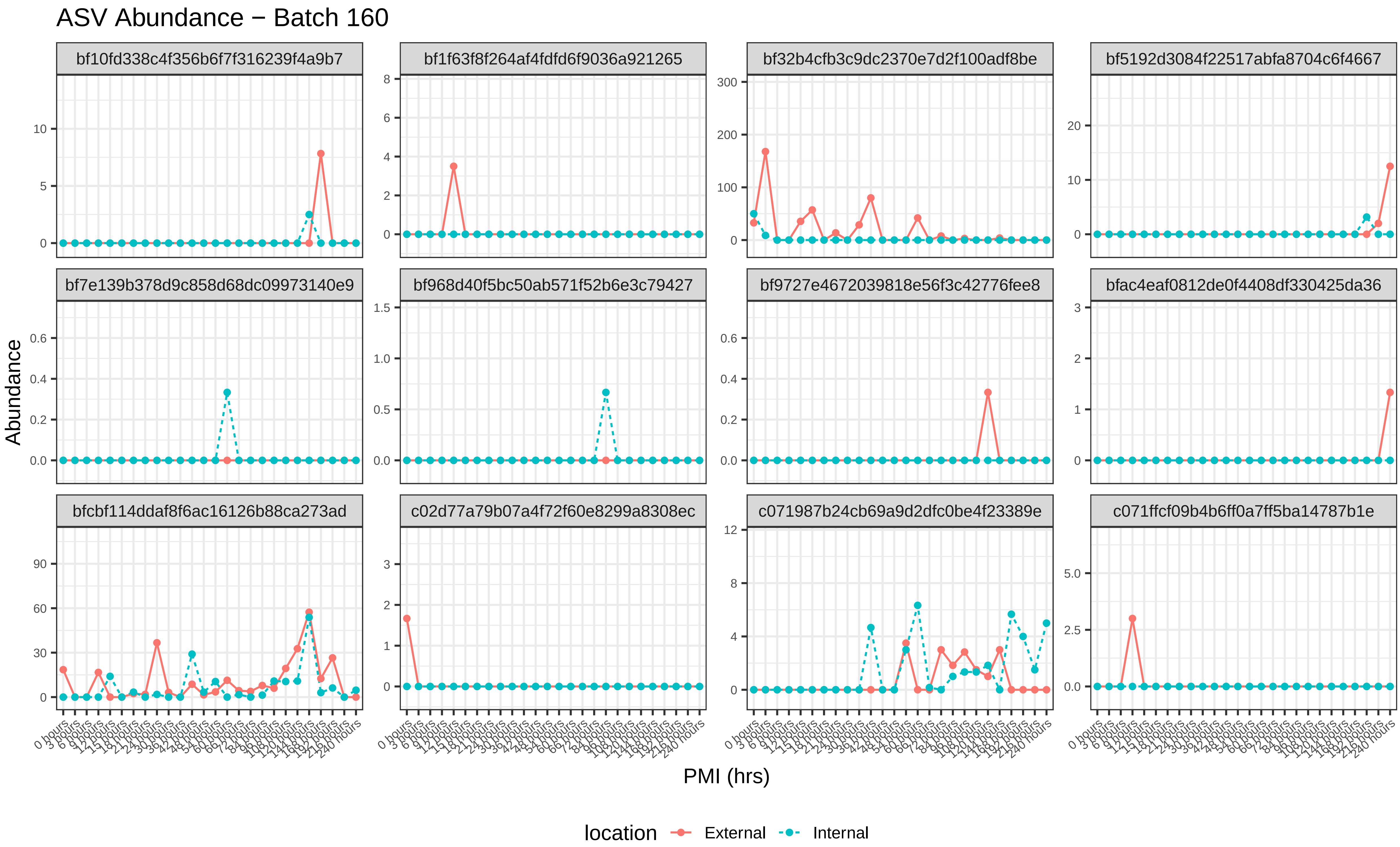

ASV Abundance − Batch 160
bf10fd338c4f356b6f7f316239f4a9b7
bf1f63f8f264af4fdfd6f9036a921265
bf32b4cfb3c9dc2370e7d2f100adf8be
bf5192d3084f22517abfa8704c6f4667
bf7e139b378d9c858d68dc09973140e9
bf968d40f5bc50ab571f52b6e3c79427
bf9727e4672039818e56f3c42776fee8
bfac4eaf0812de0f4408df330425da36
bfcbf114ddaf8f6ac16126b88ca273ad
c02d77a79b07a4f72f60e8299a8308ec
c071987b24cb69a9d2dfc0be4f23389e
c071ffcf09b4b6ff0a7ff5ba14787b1e
Abundance
PMI (hrs)
location
External
Internal

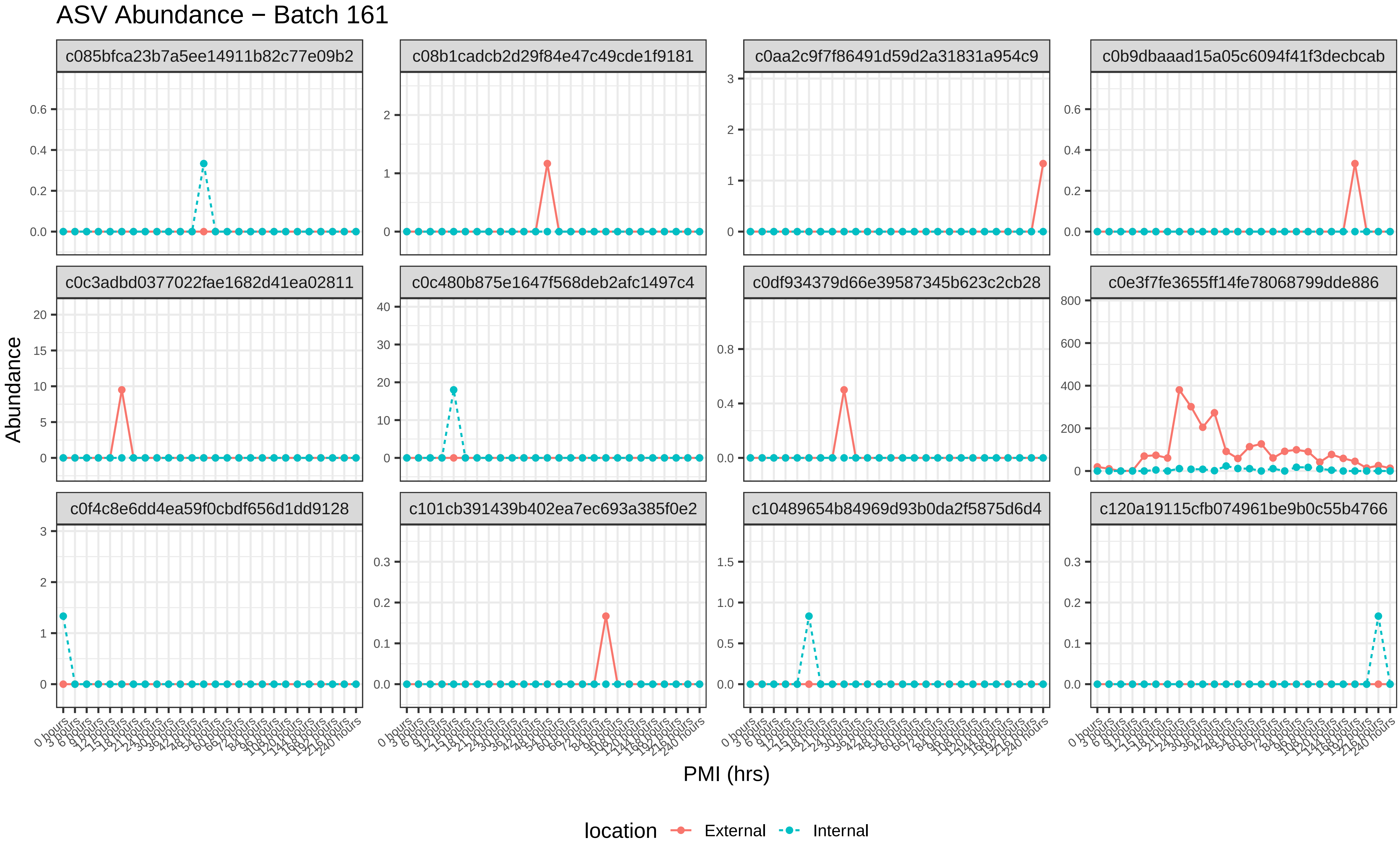

ASV Abundance − Batch 161
c085bfca23b7a5ee14911b82c77e09b2
c08b1cadcb2d29f84e47c49cde1f9181
c0aa2c9f7f86491d59d2a31831a954c9
c0b9dbaaad15a05c6094f41f3decbcab
c0c3adbd0377022fae1682d41ea02811
c0c480b875e1647f568deb2afc1497c4
c0df934379d66e39587345b623c2cb28
c0e3f7fe3655ff14fe78068799dde886
c0f4c8e6dd4ea59f0cbdf656d1dd9128
c101cb391439b402ea7ec693a385f0e2
c10489654b84969d93b0da2f5875d6d4
c120a19115cfb074961be9b0c55b4766
Abundance
PMI (hrs)
location
External
Internal

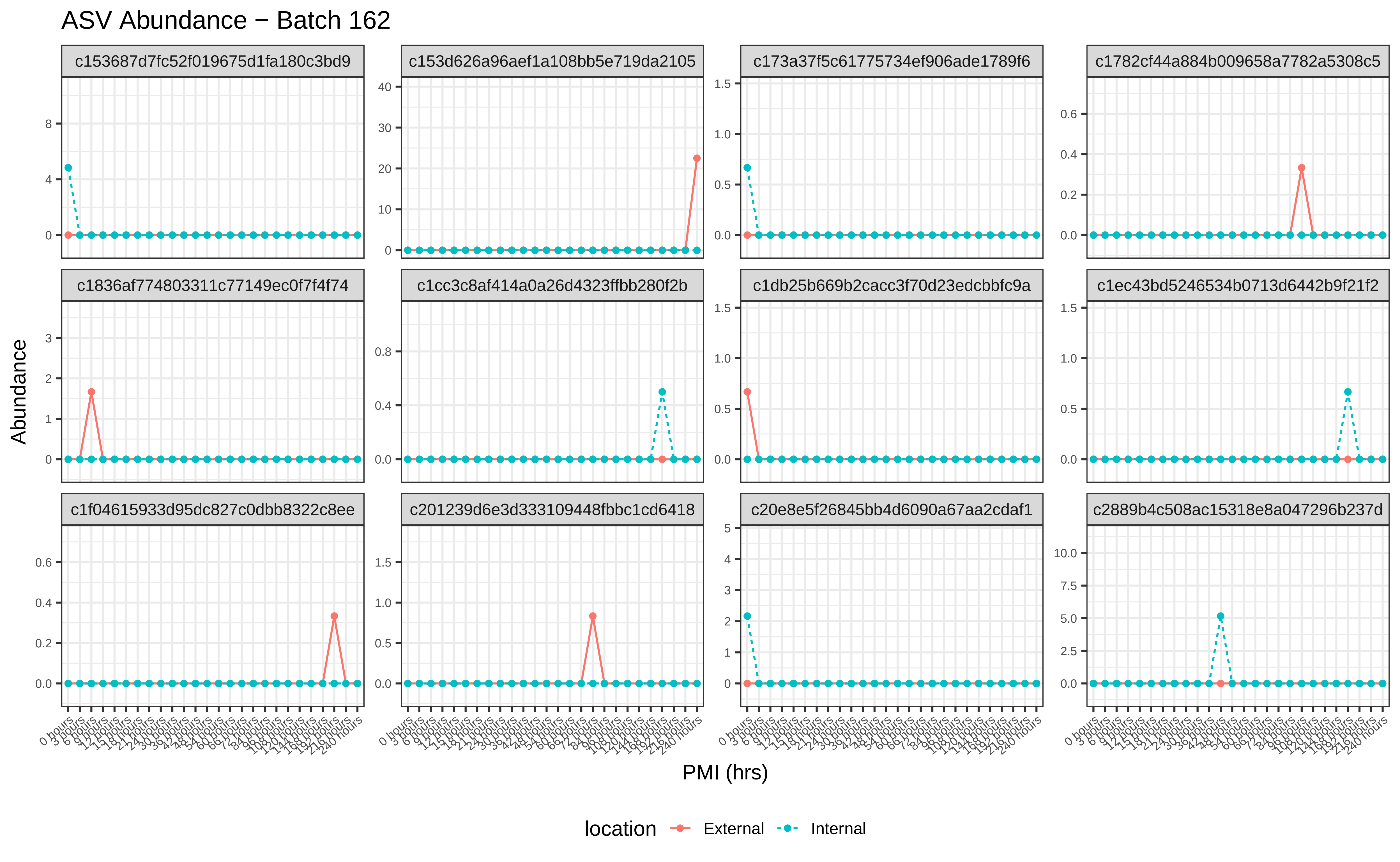
ASV Abundance − Batch 162
c153687d7fc52f019675d1fa180c3bd9
c153d626a96aef1a108bb5e719da2105
c173a37f5c61775734ef906ade1789f6
c1782cf44a884b009658a7782a5308c5
c1836af774803311c77149ec0f7f4f74
c1cc3c8af414a0a26d4323ffbb280f2b
c1db25b669b2cacc3f70d23edcbbfc9a
c1ec43bd5246534b0713d6442b9f21f2
c1f04615933d95dc827c0dbb8322c8ee
c201239d6e3d333109448fbbc1cd6418
c20e8e5f26845bb4d6090a67aa2cdaf1
c2889b4c508ac15318e8a047296b237d
Abundance
PMI (hrs)
location
External
Internal

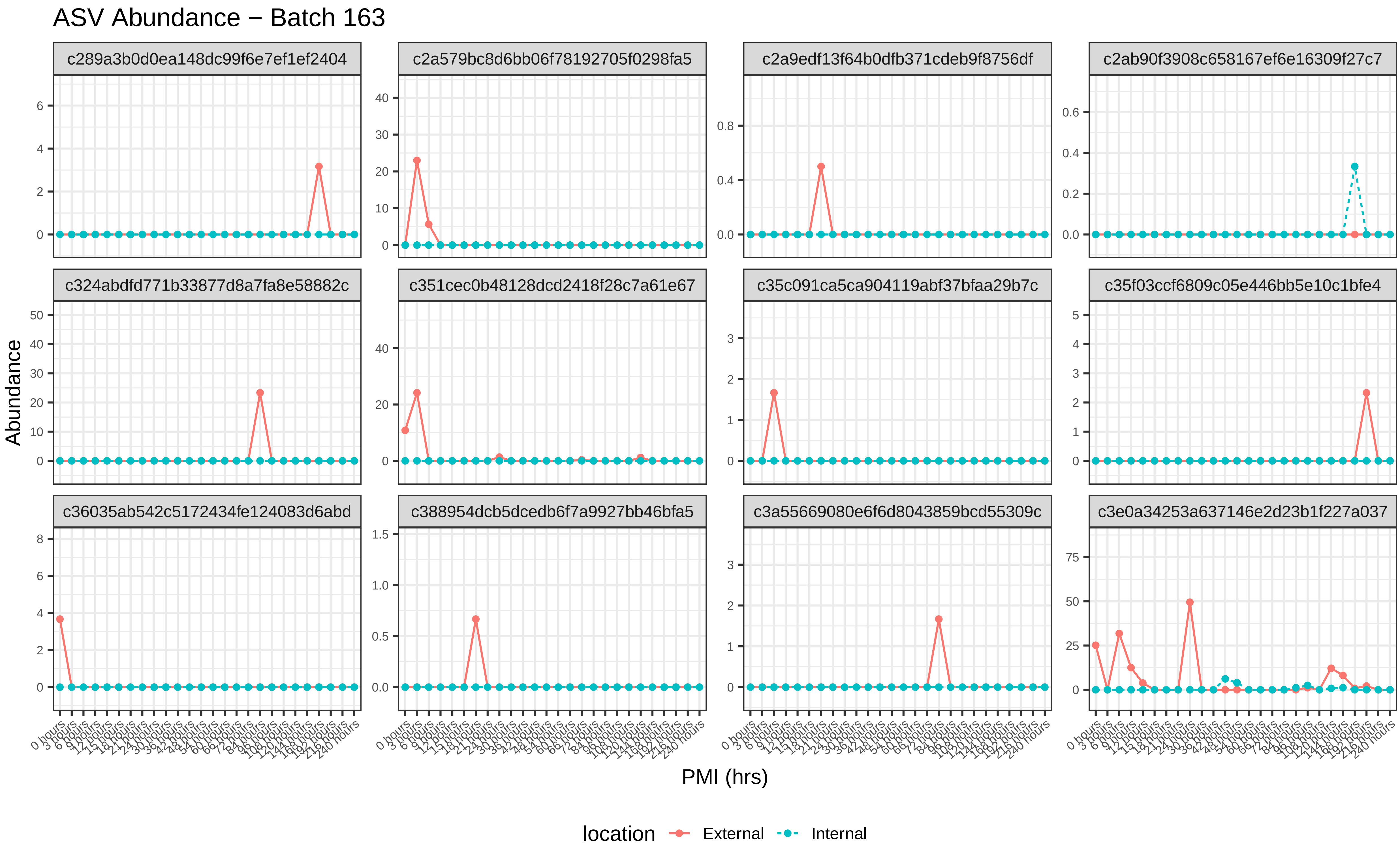

ASV Abundance − Batch 163
c289a3b0d0ea148dc99f6e7ef1ef2404
c2a579bc8d6bb06f78192705f0298fa5
c2a9edf13f64b0dfb371cdeb9f8756df
c2ab90f3908c658167ef6e16309f27c7
c324abdfd771b33877d8a7fa8e58882c
c351cec0b48128dcd2418f28c7a61e67
c35c091ca5ca904119abf37bfaa29b7c
c35f03ccf6809c05e446bb5e10c1bfe4
c36035ab542c5172434fe124083d6abd
c388954dcb5dcedb6f7a9927bb46bfa5
c3a55669080e6f6d8043859bcd55309c
c3e0a34253a637146e2d23b1f227a037
Abundance
PMI (hrs)
location
External
Internal

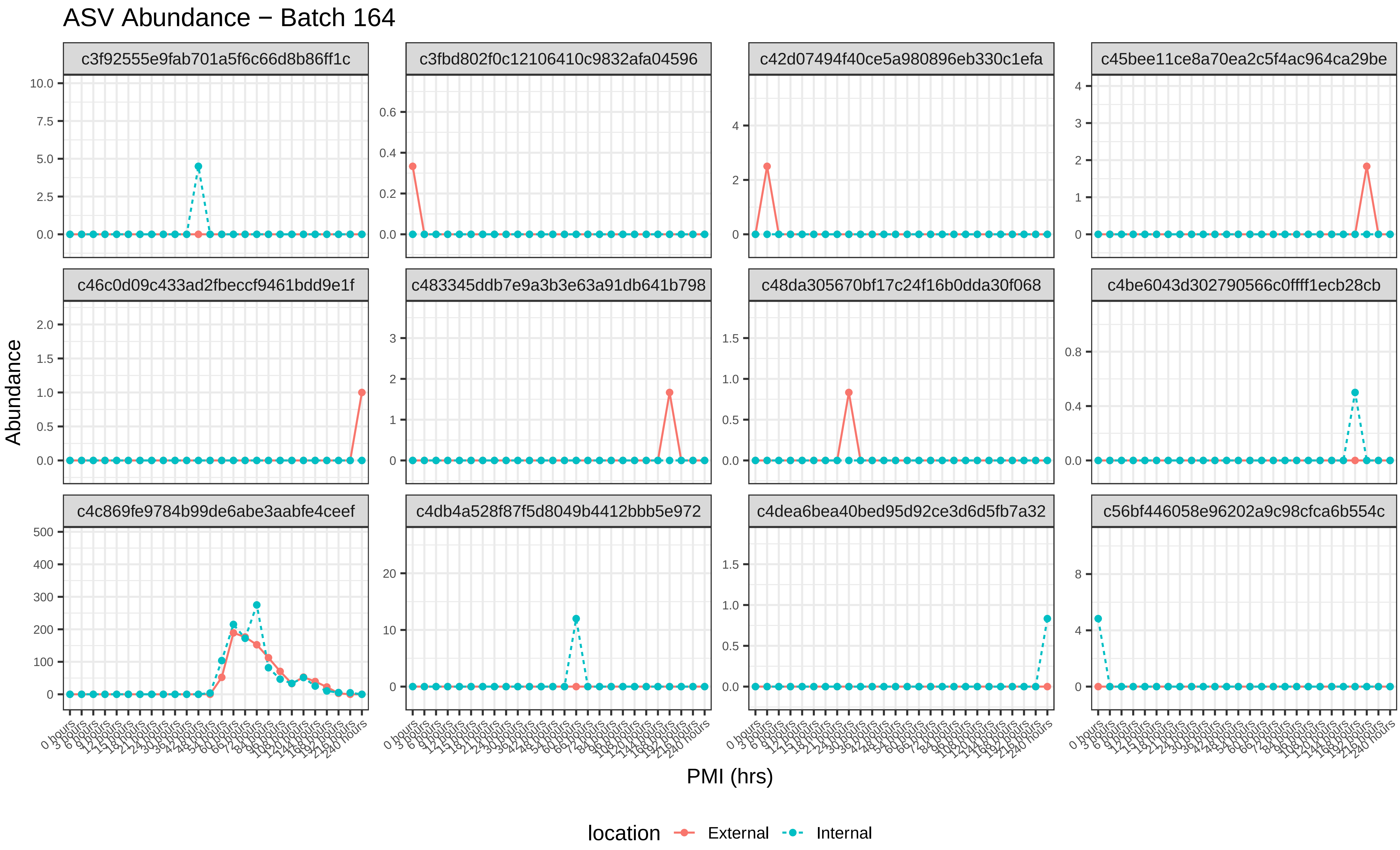
ASV Abundance − Batch 164
c3f92555e9fab701a5f6c66d8b86ff1c
c3fbd802f0c12106410c9832afa04596
c42d07494f40ce5a980896eb330c1efa
c45bee11ce8a70ea2c5f4ac964ca29be
c46c0d09c433ad2fbeccf9461bdd9e1f
c483345ddb7e9a3b3e63a91db641b798
c48da305670bf17c24f16b0dda30f068
c4be6043d302790566c0ffff1ecb28cb
c4c869fe9784b99de6abe3aabfe4ceef
c4db4a528f87f5d8049b4412bbb5e972
c4dea6bea40bed95d92ce3d6d5fb7a32
c56bf446058e96202a9c98cfca6b554c
Abundance
PMI (hrs)
location
External
Internal

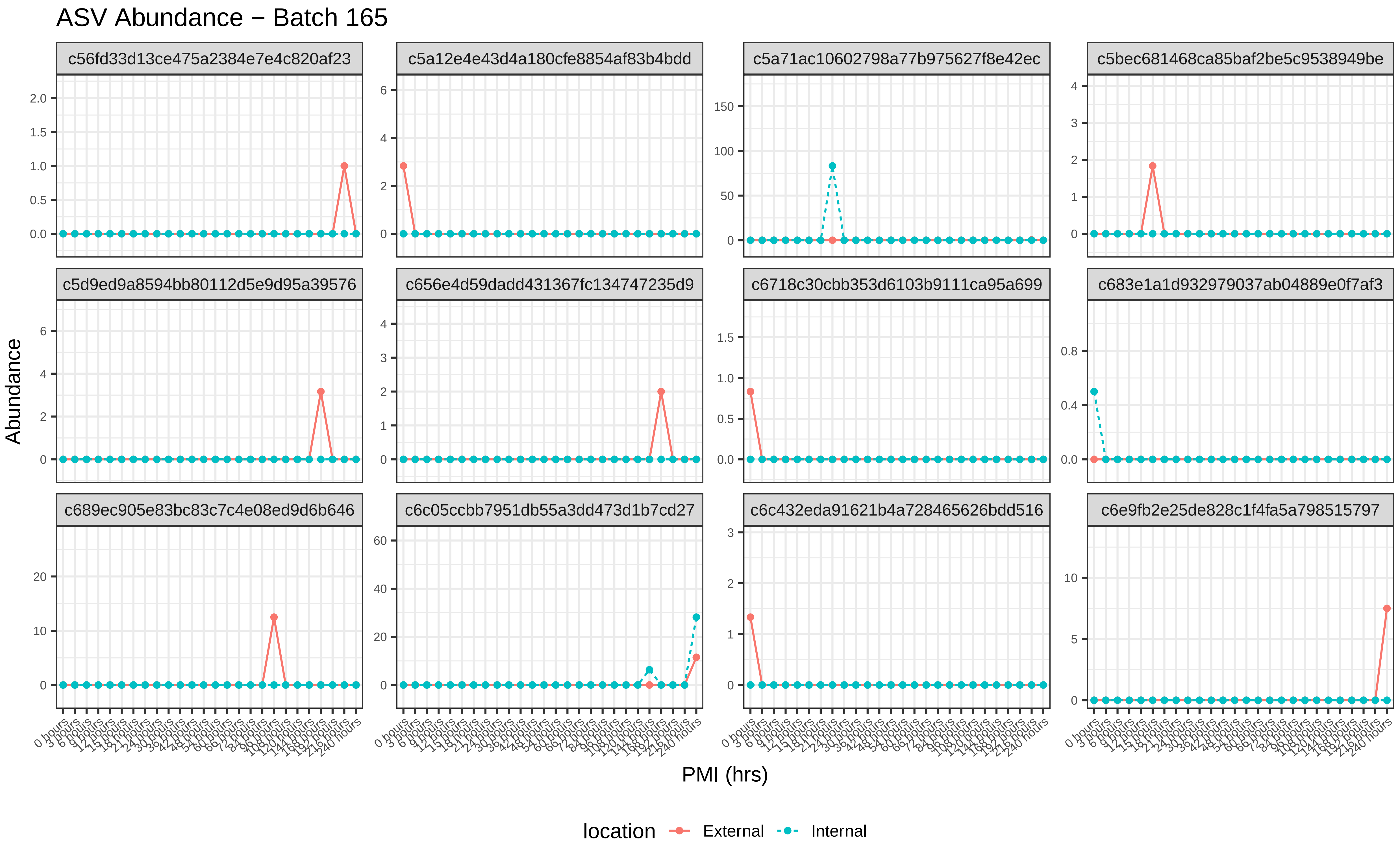
ASV Abundance − Batch 165
c56fd33d13ce475a2384e7e4c820af23
c5a12e4e43d4a180cfe8854af83b4bdd
c5a71ac10602798a77b975627f8e42ec
c5bec681468ca85baf2be5c9538949be
c5d9ed9a8594bb80112d5e9d95a39576
c656e4d59dadd431367fc134747235d9
c6718c30cbb353d6103b9111ca95a699
c683e1a1d932979037ab04889e0f7af3
c689ec905e83bc83c7c4e08ed9d6b646
c6c05ccbb7951db55a3dd473d1b7cd27
c6c432eda91621b4a728465626bdd516
c6e9fb2e25de828c1f4fa5a798515797
Abundance
PMI (hrs)
location
External
Internal

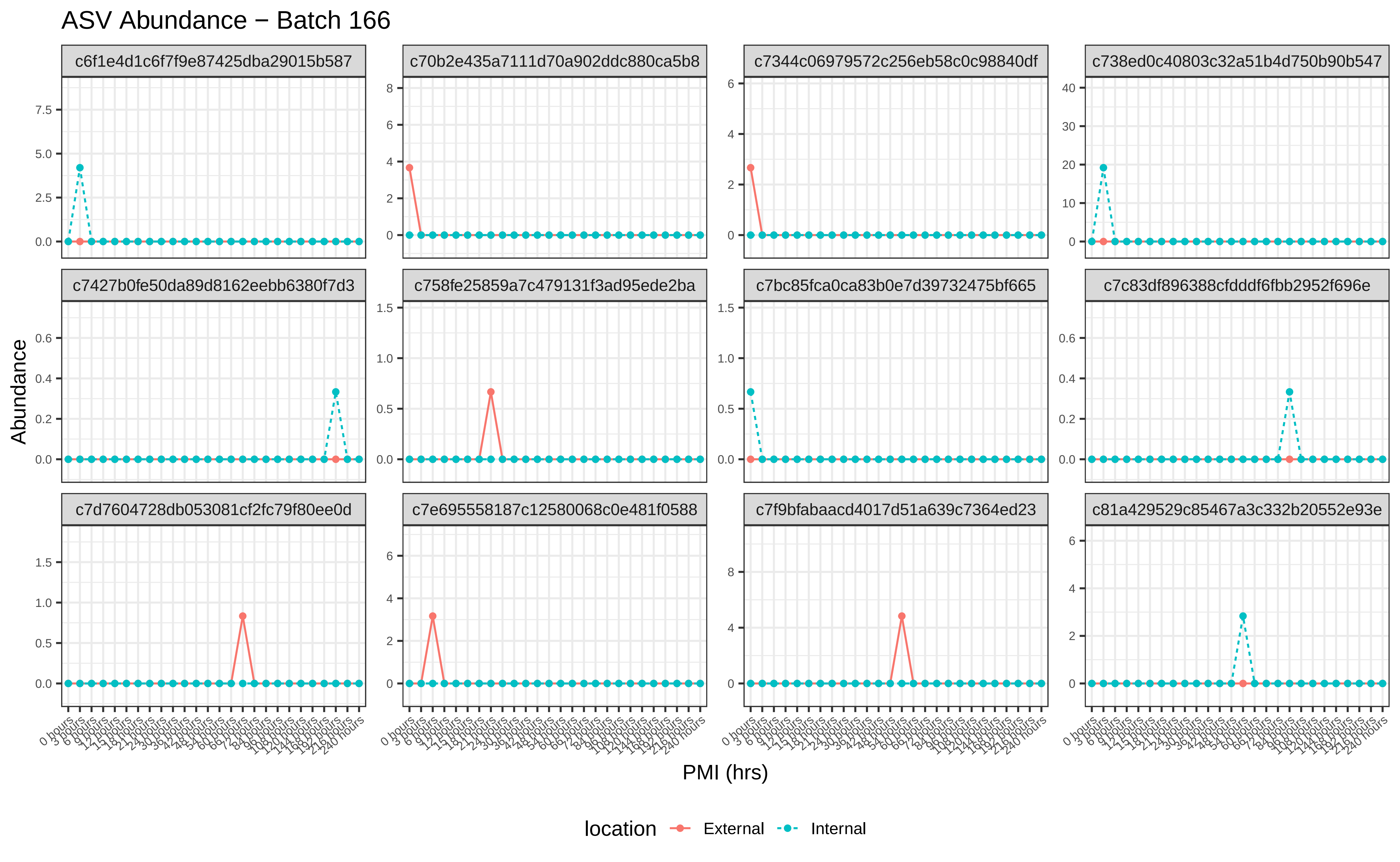

ASV Abundance − Batch 166
c6f1e4d1c6f7f9e87425dba29015b587
c70b2e435a7111d70a902ddc880ca5b8
c7344c06979572c256eb58c0c98840df
c738ed0c40803c32a51b4d750b90b547
c7427b0fe50da89d8162eebb6380f7d3
c758fe25859a7c479131f3ad95ede2ba
c7bc85fca0ca83b0e7d39732475bf665
c7c83df896388cfdddf6fbb2952f696e
c7d7604728db053081cf2fc79f80ee0d
c7e695558187c12580068c0e481f0588
c7f9bfabaacd4017d51a639c7364ed23
c81a429529c85467a3c332b20552e93e
Abundance
PMI (hrs)
location
External
Internal

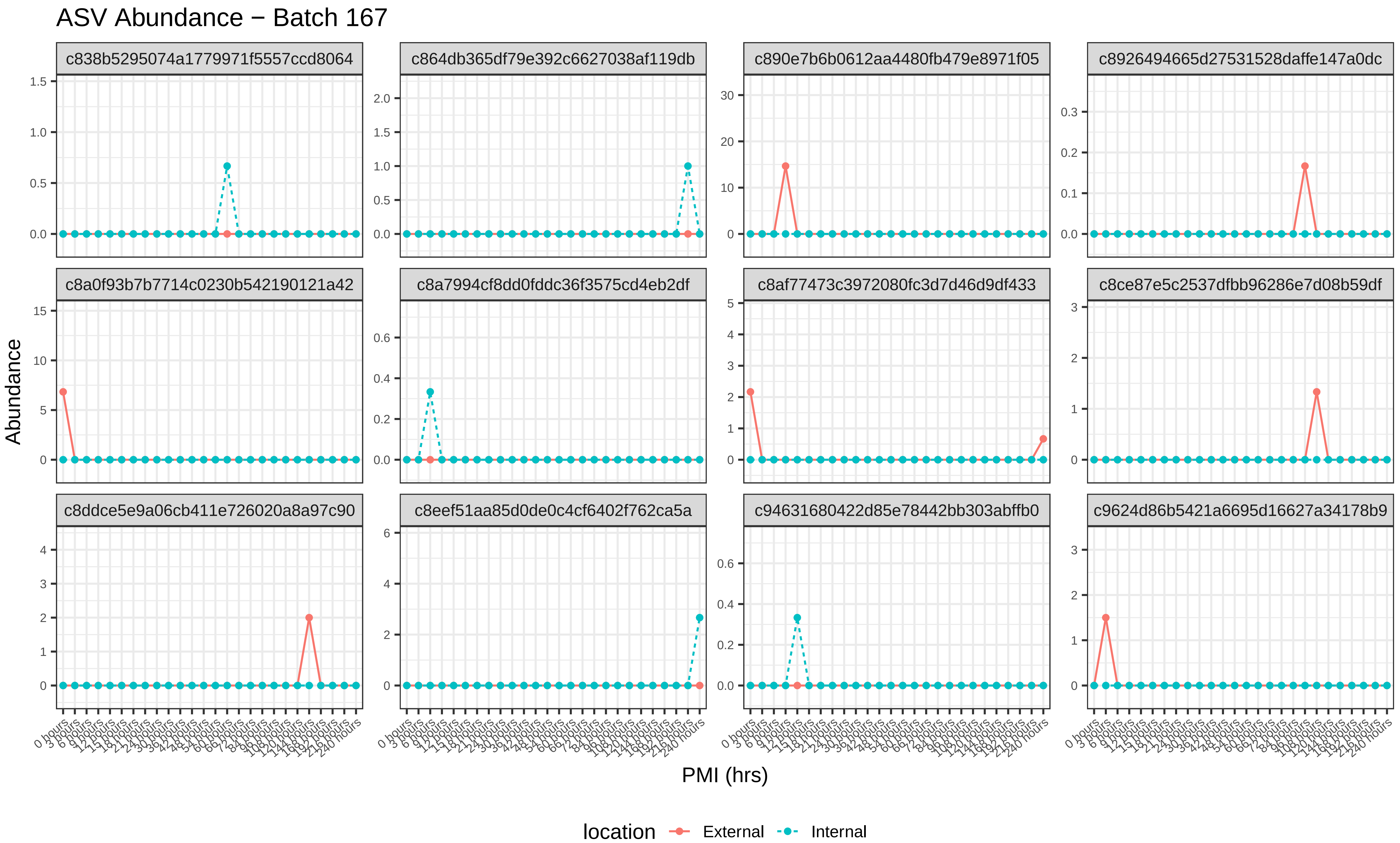
ASV Abundance − Batch 167
c838b5295074a1779971f5557ccd8064
c864db365df79e392c6627038af119db
c890e7b6b0612aa4480fb479e8971f05
c8926494665d27531528daffe147a0dc
c8a0f93b7b7714c0230b542190121a42
c8a7994cf8dd0fddc36f3575cd4eb2df
c8af77473c3972080fc3d7d46d9df433
c8ce87e5c2537dfbb96286e7d08b59df
c8ddce5e9a06cb411e726020a8a97c90
c8eef51aa85d0de0c4cf6402f762ca5a
c94631680422d85e78442bb303abffb0
c9624d86b5421a6695d16627a34178b9
Abundance
PMI (hrs)
location
External
Internal

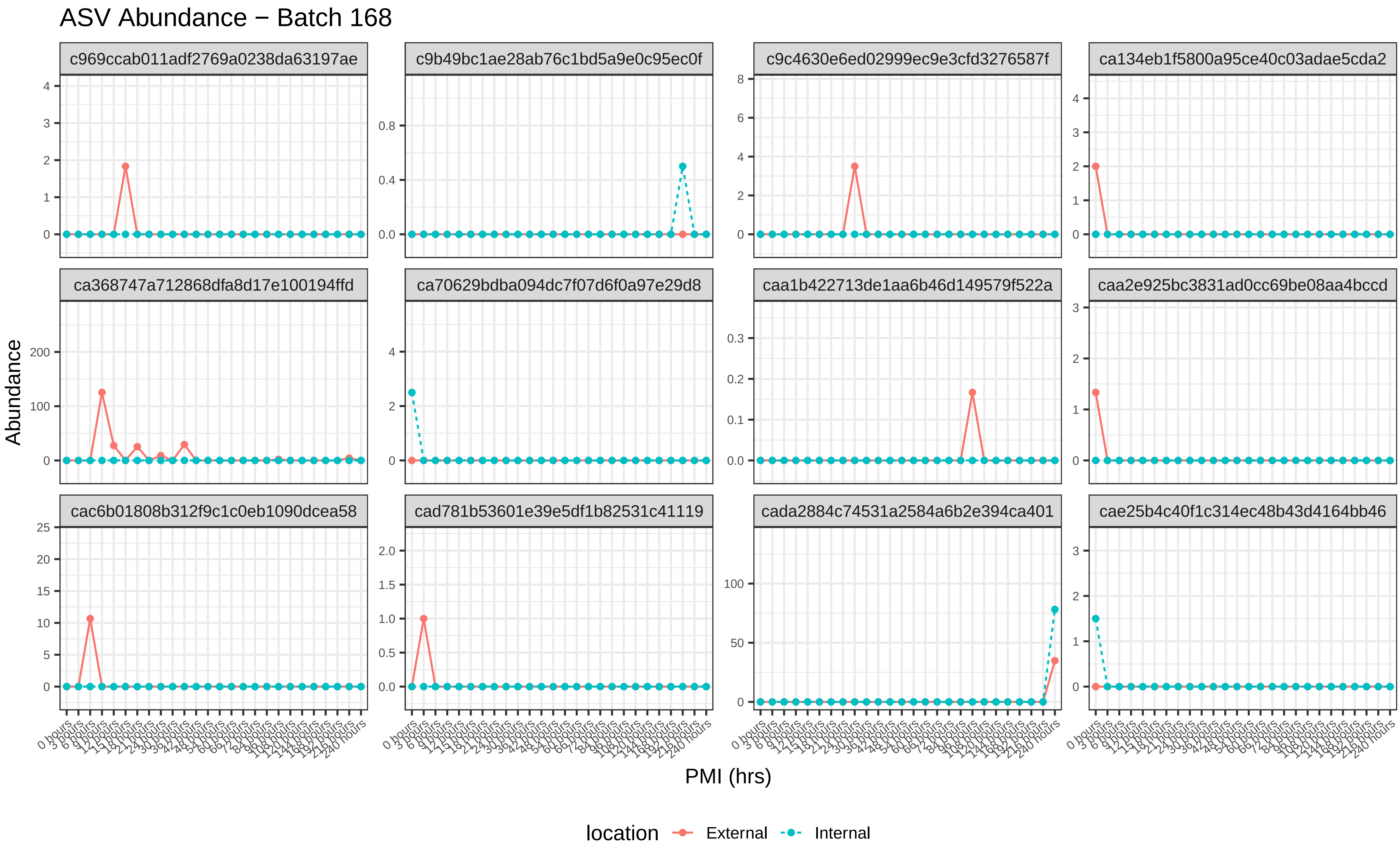
ASV Abundance − Batch 168
c969ccab011adf2769a0238da63197ae
c9b49bc1ae28ab76c1bd5a9e0c95ec0f
c9c4630e6ed02999ec9e3cfd3276587f
ca134eb1f5800a95ce40c03adae5cda2
ca368747a712868dfa8d17e100194ffd
ca70629bdba094dc7f07d6f0a97e29d8
caa1b422713de1aa6b46d149579f522a
caa2e925bc3831ad0cc69be08aa4bccd
cac6b01808b312f9c1c0eb1090dcea58
cad781b53601e39e5df1b82531c41119
cada2884c74531a2584a6b2e394ca401
cae25b4c40f1c314ec48b43d4164bb46
Abundance
PMI (hrs)
location
External
Internal

ASV Abundance − Batch 169

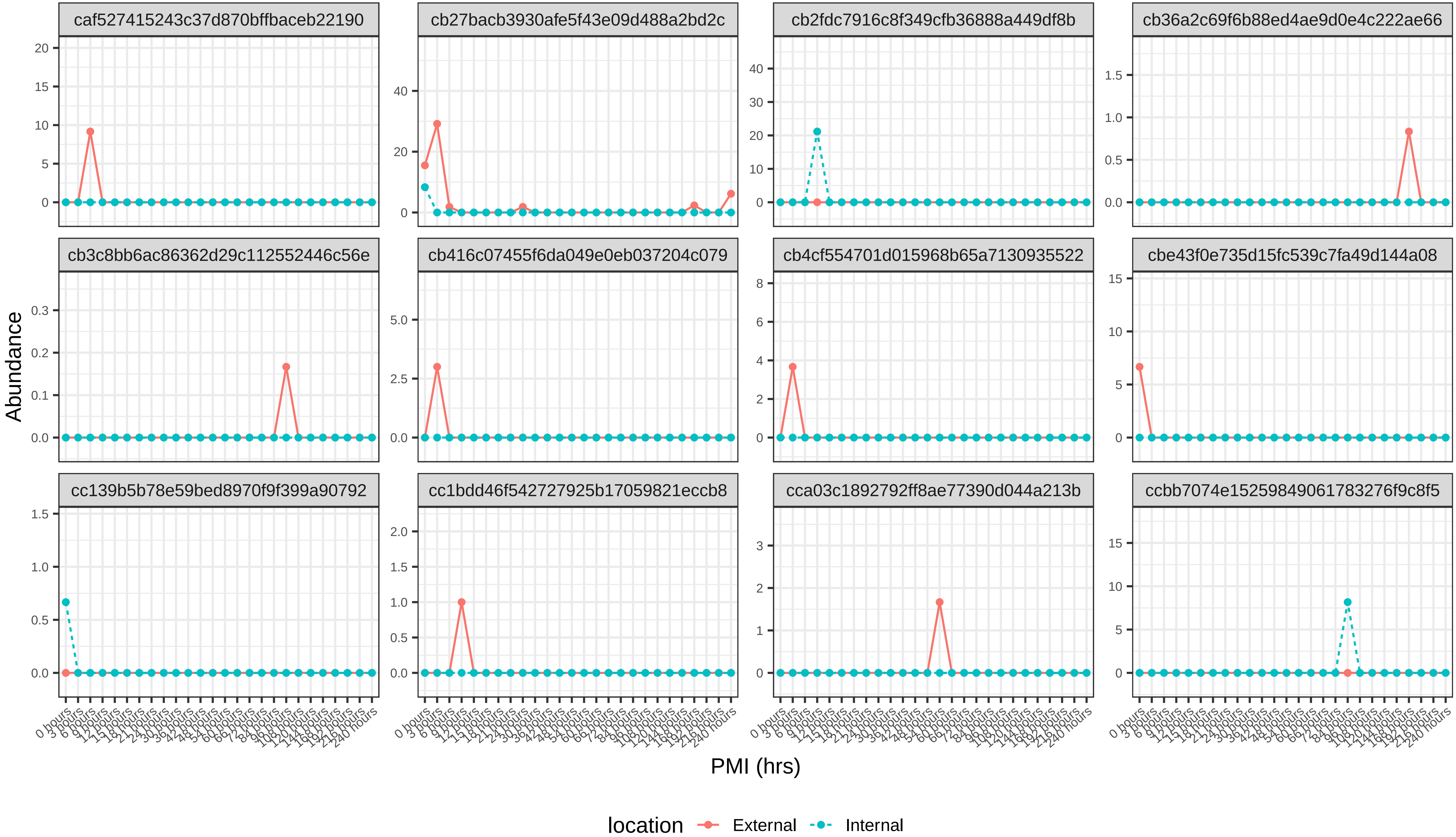

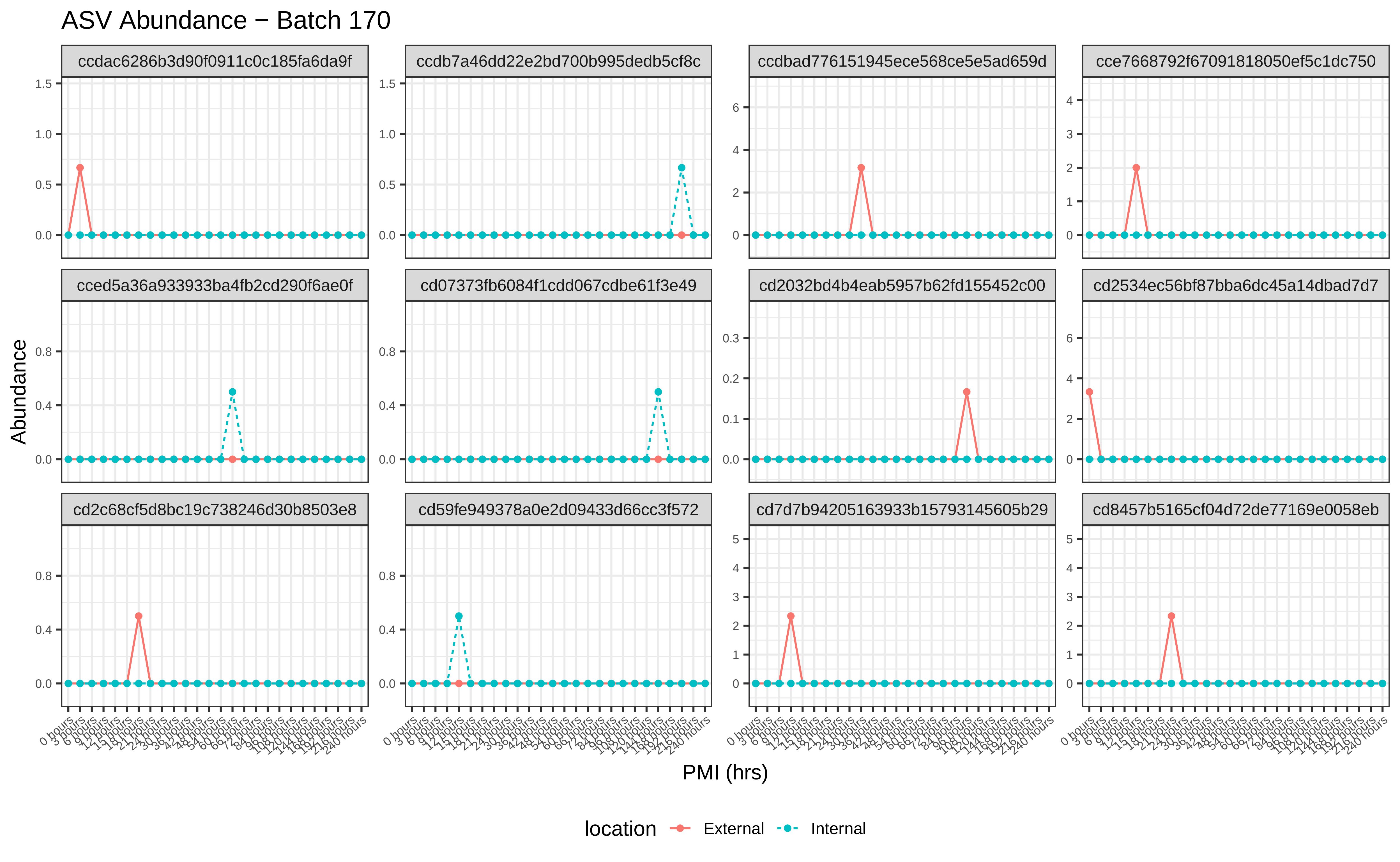

ASV Abundance − Batch 170
ccdac6286b3d90f0911c0c185fa6da9f
ccdb7a46dd22e2bd700b995dedb5cf8c
ccdbad776151945ece568ce5e5ad659d
cce7668792f67091818050ef5c1dc750
cced5a36a933933ba4fb2cd290f6ae0f
cd07373fb6084f1cdd067cdbe61f3e49
cd2032bd4b4eab5957b62fd155452c00
cd2534ec56bf87bba6dc45a14dbad7d7
cd2c68cf5d8bc19c738246d30b8503e8
cd59fe949378a0e2d09433d66cc3f572
cd7d7b94205163933b15793145605b29
cd8457b5165cf04d72de77169e0058eb
Abundance
PMI (hrs)
location
External
Internal

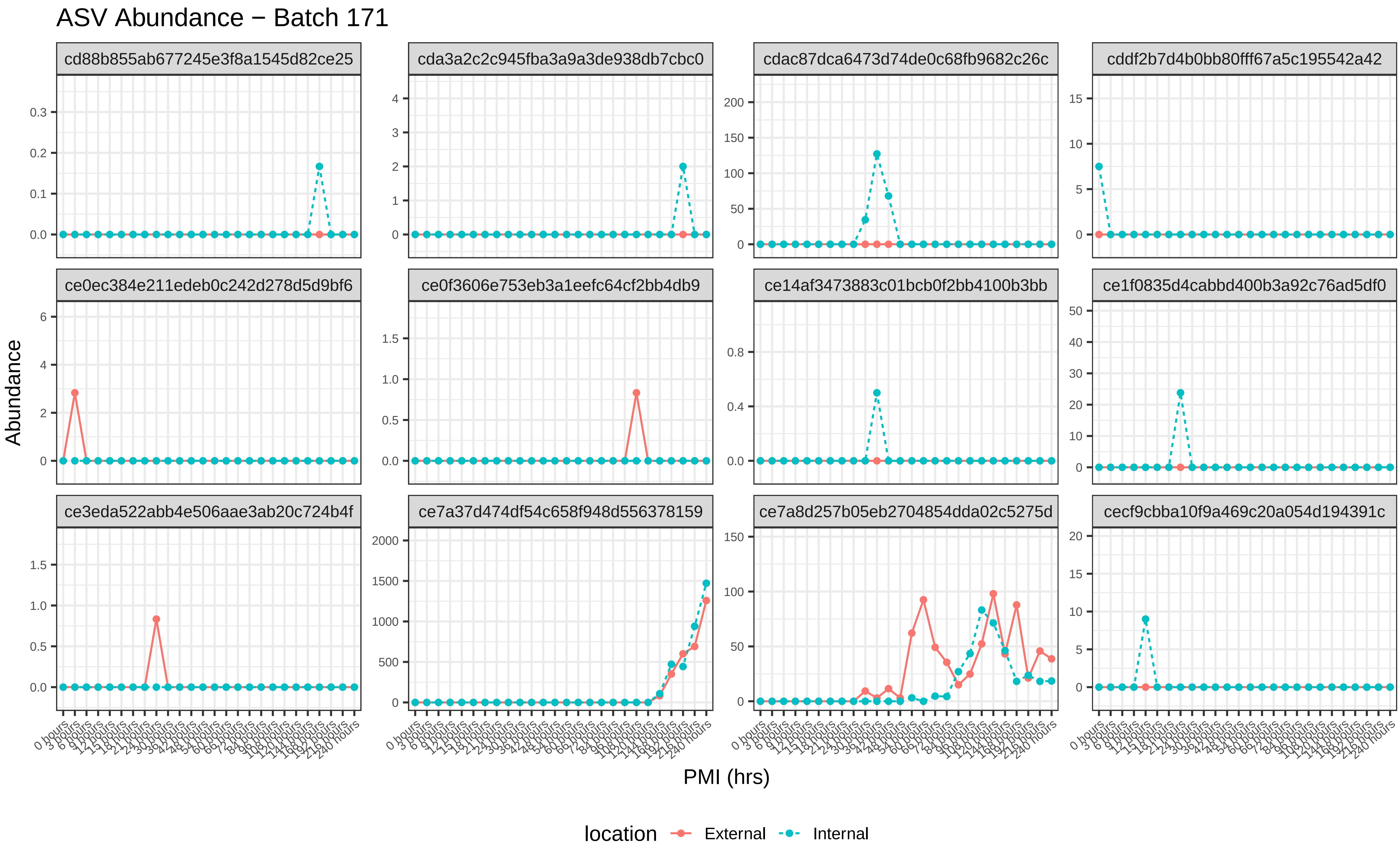
ASV Abundance − Batch 171
cd88b855ab677245e3f8a1545d82ce25
cda3a2c2c945fba3a9a3de938db7cbc0
cdac87dca6473d74de0c68fb9682c26c
cddf2b7d4b0bb80fff67a5c195542a42
ce0ec384e211edeb0c242d278d5d9bf6
ce0f3606e753eb3a1eefc64cf2bb4db9
ce14af3473883c01bcb0f2bb4100b3bb
ce1f0835d4cabbd400b3a92c76ad5df0
ce3eda522abb4e506aae3ab20c724b4f
ce7a37d474df54c658f948d556378159
ce7a8d257b05eb2704854dda02c5275d
cecf9cbba10f9a469c20a054d194391c
Abundance
PMI (hrs)
location
External
Internal

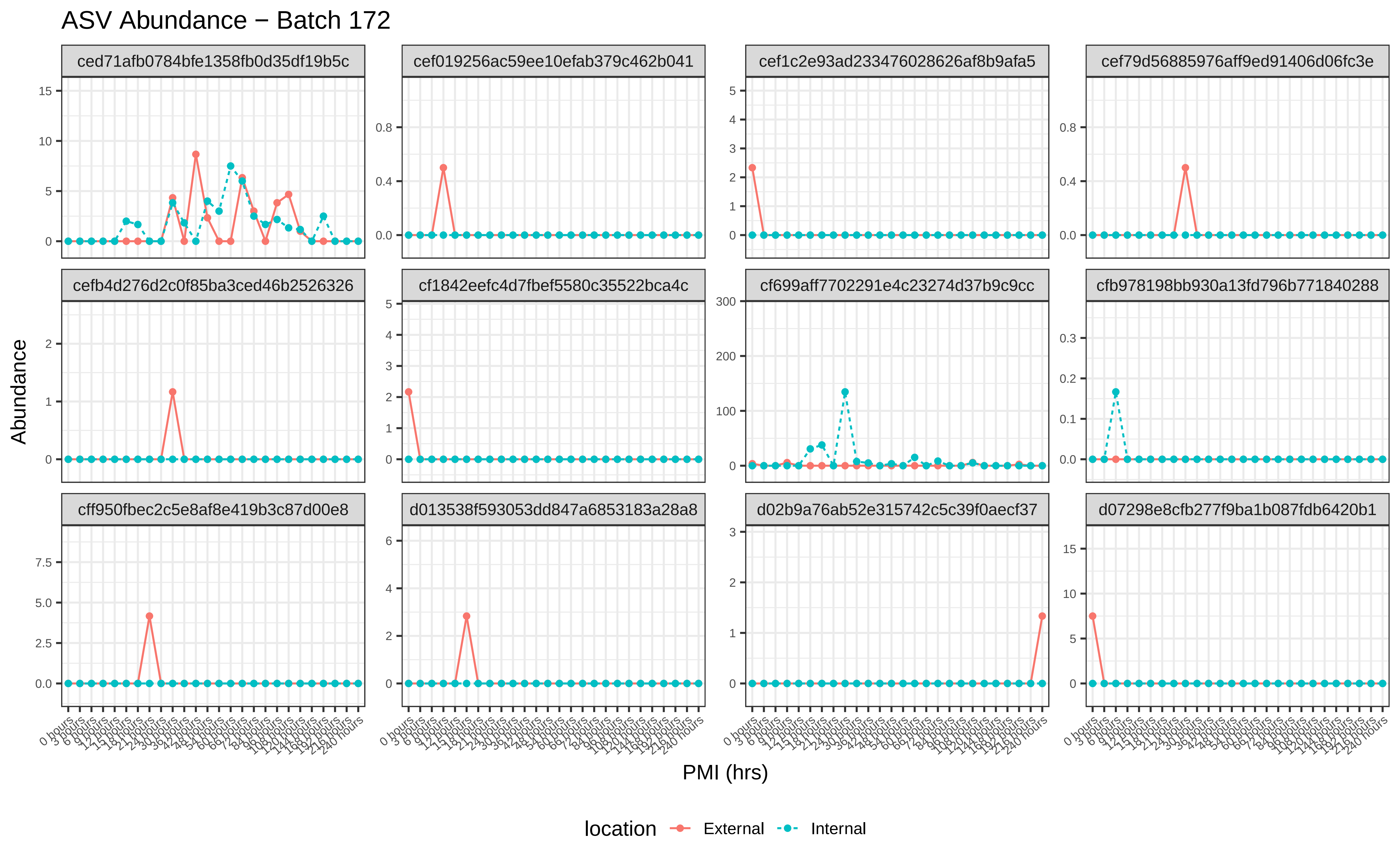
ASV Abundance − Batch 172
ced71afb0784bfe1358fb0d35df19b5c
cef019256ac59ee10efab379c462b041
cef1c2e93ad233476028626af8b9afa5
cef79d56885976aff9ed91406d06fc3e
cefb4d276d2c0f85ba3ced46b2526326
cf1842eefc4d7fbef5580c35522bca4c
cf699aff7702291e4c23274d37b9c9cc
cfb978198bb930a13fd796b771840288
cff950fbec2c5e8af8e419b3c87d00e8
d013538f593053dd847a6853183a28a8
d02b9a76ab52e315742c5c39f0aecf37
d07298e8cfb277f9ba1b087fdb6420b1
Abundance
PMI (hrs)
location
External
Internal

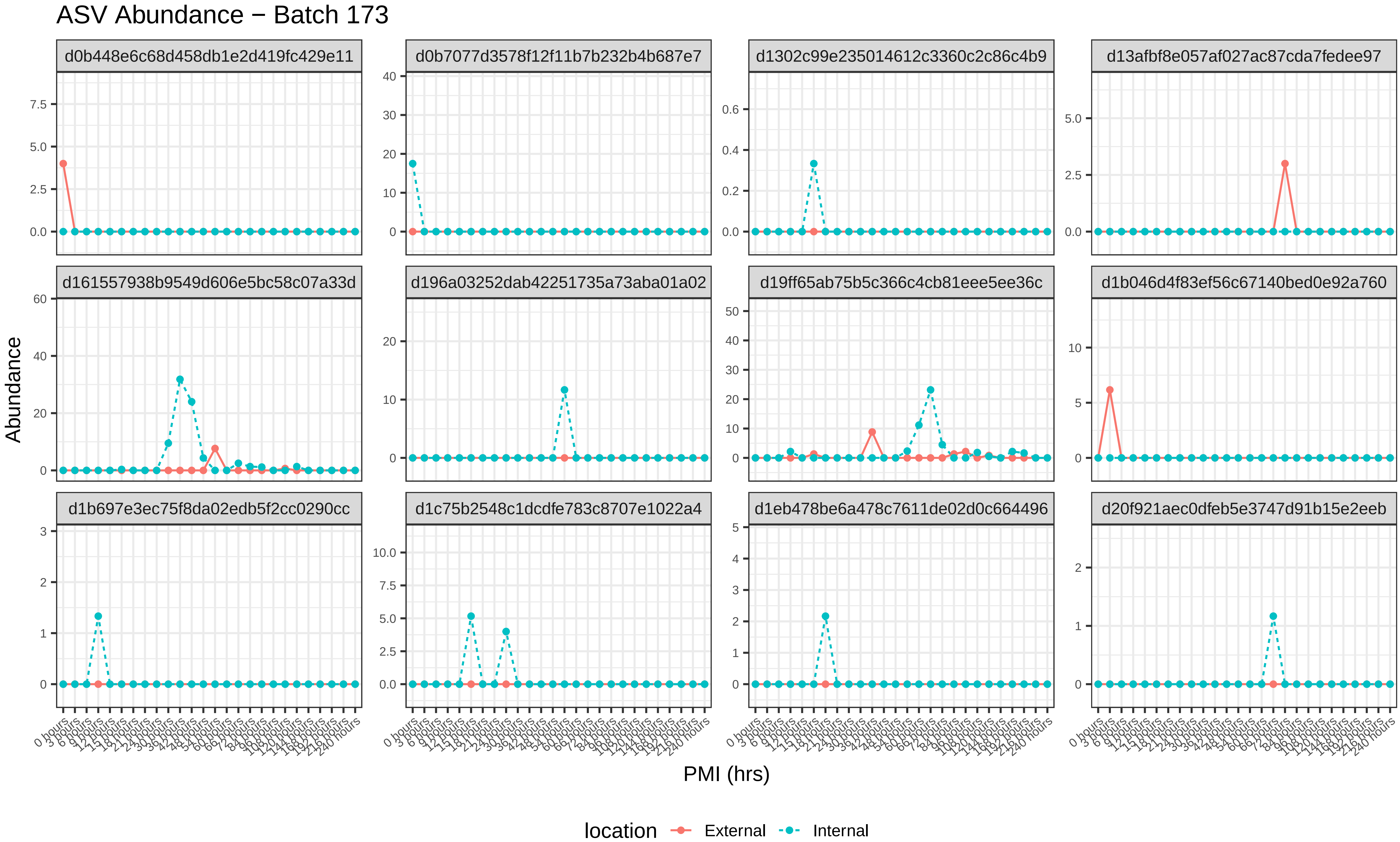
ASV Abundance − Batch 173
d0b448e6c68d458db1e2d419fc429e11
d0b7077d3578f12f11b7b232b4b687e7
d1302c99e235014612c3360c2c86c4b9
d13afbf8e057af027ac87cda7fedee97
d161557938b9549d606e5bc58c07a33d
d196a03252dab42251735a73aba01a02
d19ff65ab75b5c366c4cb81eee5ee36c
d1b046d4f83ef56c67140bed0e92a760
d1b697e3ec75f8da02edb5f2cc0290cc
d1c75b2548c1dcdfe783c8707e1022a4
d1eb478be6a478c7611de02d0c664496
d20f921aec0dfeb5e3747d91b15e2eeb
Abundance
PMI (hrs)
location
External
Internal

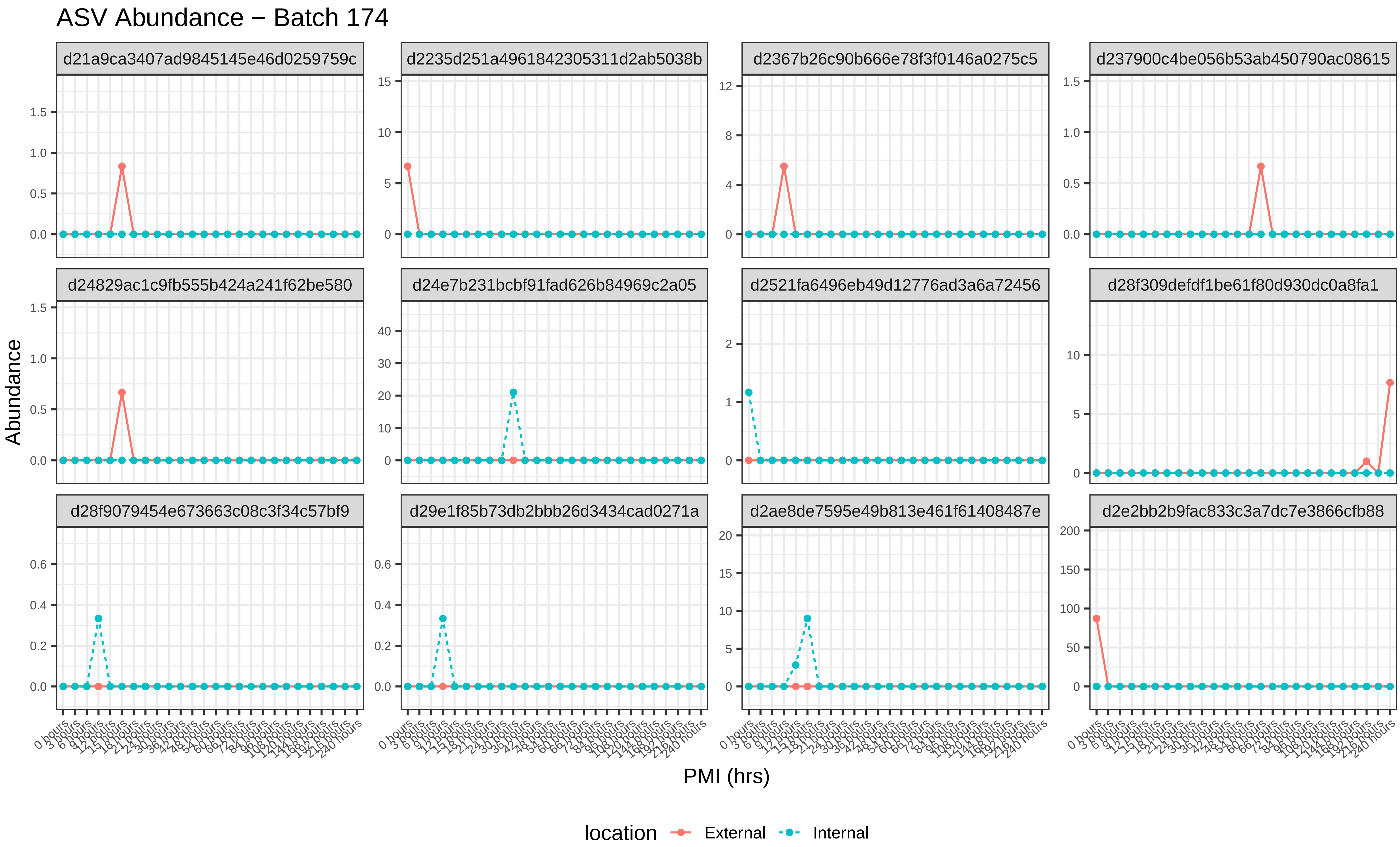

ASV Abundance − Batch 174
d21a9ca3407ad9845145e46d0259759c
d2235d251a4961842305311d2ab5038b
d2367b26c90b666e78f3f0146a0275c5
d237900c4be056b53ab450790ac08615
d24829ac1c9fb555b424a241f62be580
d24e7b231bcbf91fad626b84969c2a05
d2521fa6496eb49d12776ad3a6a72456
d28f309defdf1be61f80d930dc0a8fa1
d28f9079454e673663c08c3f34c57bf9
d29e1f85b73db2bbb26d3434cad0271a
d2ae8de7595e49b813e461f61408487e
d2e2bb2b9fac833c3a7dc7e3866cfb88
Abundance
PMI (hrs)
location
External
Internal

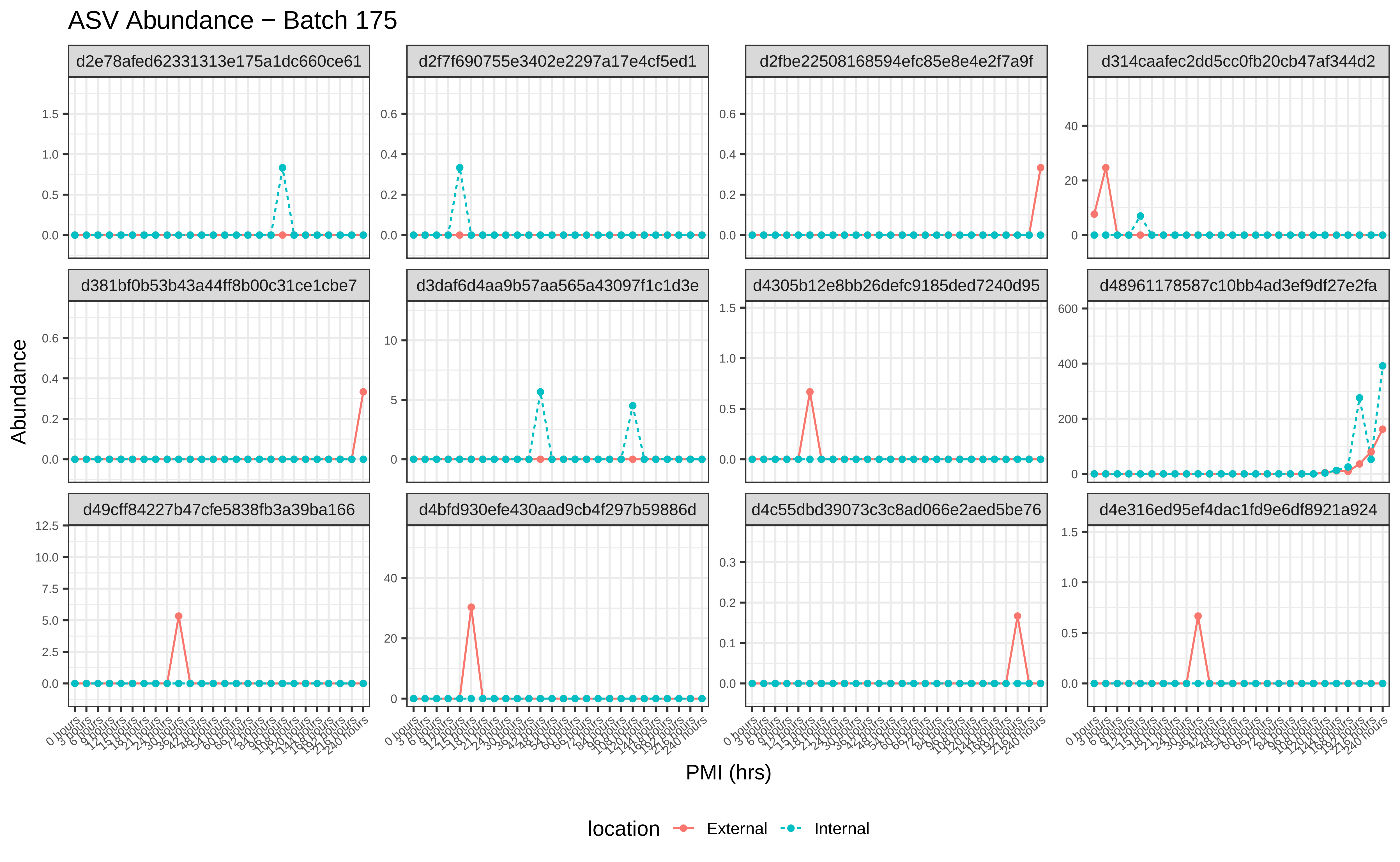
ASV Abundance − Batch 175
d2e78afed62331313e175a1dc660ce61
d2f7f690755e3402e2297a17e4cf5ed1
d2fbe22508168594efc85e8e4e2f7a9f
d314caafec2dd5cc0fb20cb47af344d2
d381bf0b53b43a44ff8b00c31ce1cbe7
d3daf6d4aa9b57aa565a43097f1c1d3e
d4305b12e8bb26defc9185ded7240d95
d48961178587c10bb4ad3ef9df27e2fa
d49cff84227b47cfe5838fb3a39ba166
d4bfd930efe430aad9cb4f297b59886d
d4c55dbd39073c3c8ad066e2aed5be76
d4e316ed95ef4dac1fd9e6df8921a924
Abundance
PMI (hrs)
location
External
Internal

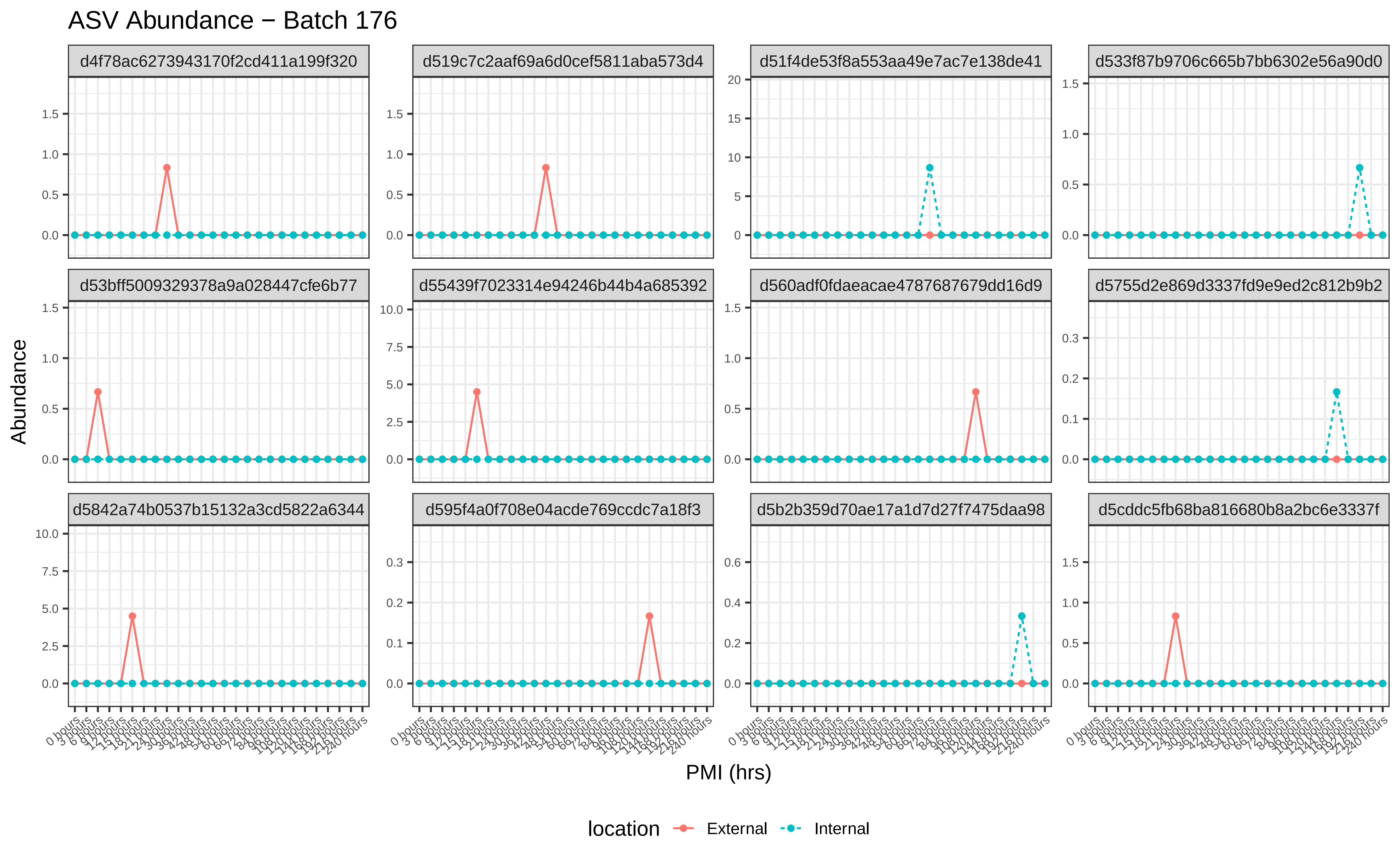
ASV Abundance − Batch 176
d4f78ac6273943170f2cd411a199f320
d519c7c2aaf69a6d0cef5811aba573d4
d51f4de53f8a553aa49e7ac7e138de41
d533f87b9706c665b7bb6302e56a90d0
d53bff5009329378a9a028447cfe6b77
d55439f7023314e94246b44b4a685392
d560adf0fdaeacae4787687679dd16d9
d5755d2e869d3337fd9e9ed2c812b9b2
d5842a74b0537b15132a3cd5822a6344
d595f4a0f708e04acde769ccdc7a18f3
d5b2b359d70ae17a1d7d27f7475daa98
d5cddc5fb68ba816680b8a2bc6e3337f
Abundance
PMI (hrs)
location
External
Internal

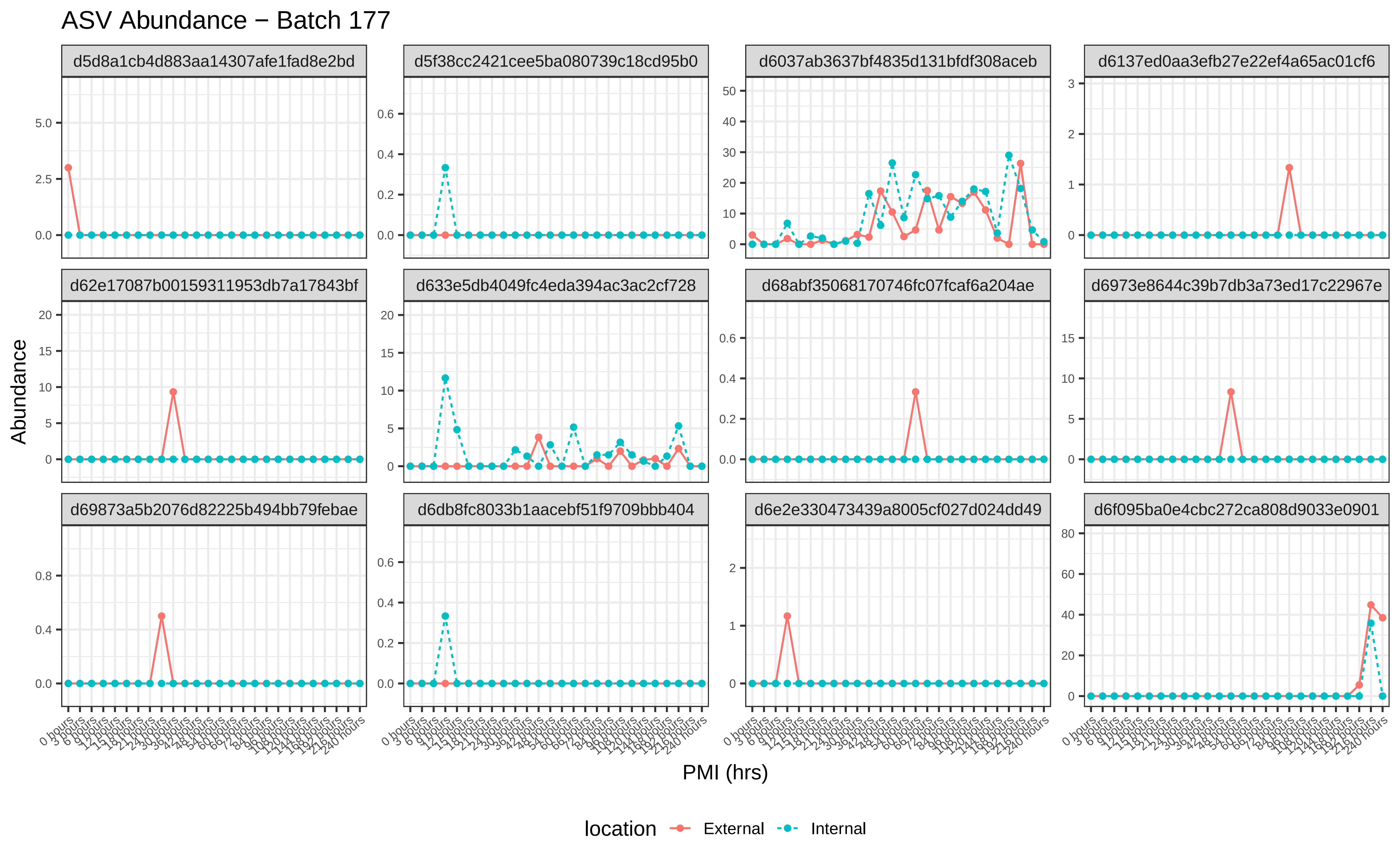
ASV Abundance − Batch 177
d5d8a1cb4d883aa14307afe1fad8e2bd
d5f38cc2421cee5ba080739c18cd95b0
d6037ab3637bf4835d131bfdf308aceb
d6137ed0aa3efb27e22ef4a65ac01cf6
d62e17087b00159311953db7a17843bf
d633e5db4049fc4eda394ac3ac2cf728
d68abf35068170746fc07fcaf6a204ae
d6973e8644c39b7db3a73ed17c22967e
d69873a5b2076d82225b494bb79febae
d6db8fc8033b1aacebf51f9709bbb404
d6e2e330473439a8005cf027d024dd49
d6f095ba0e4cbc272ca808d9033e0901
Abundance
PMI (hrs)
location
External
Internal

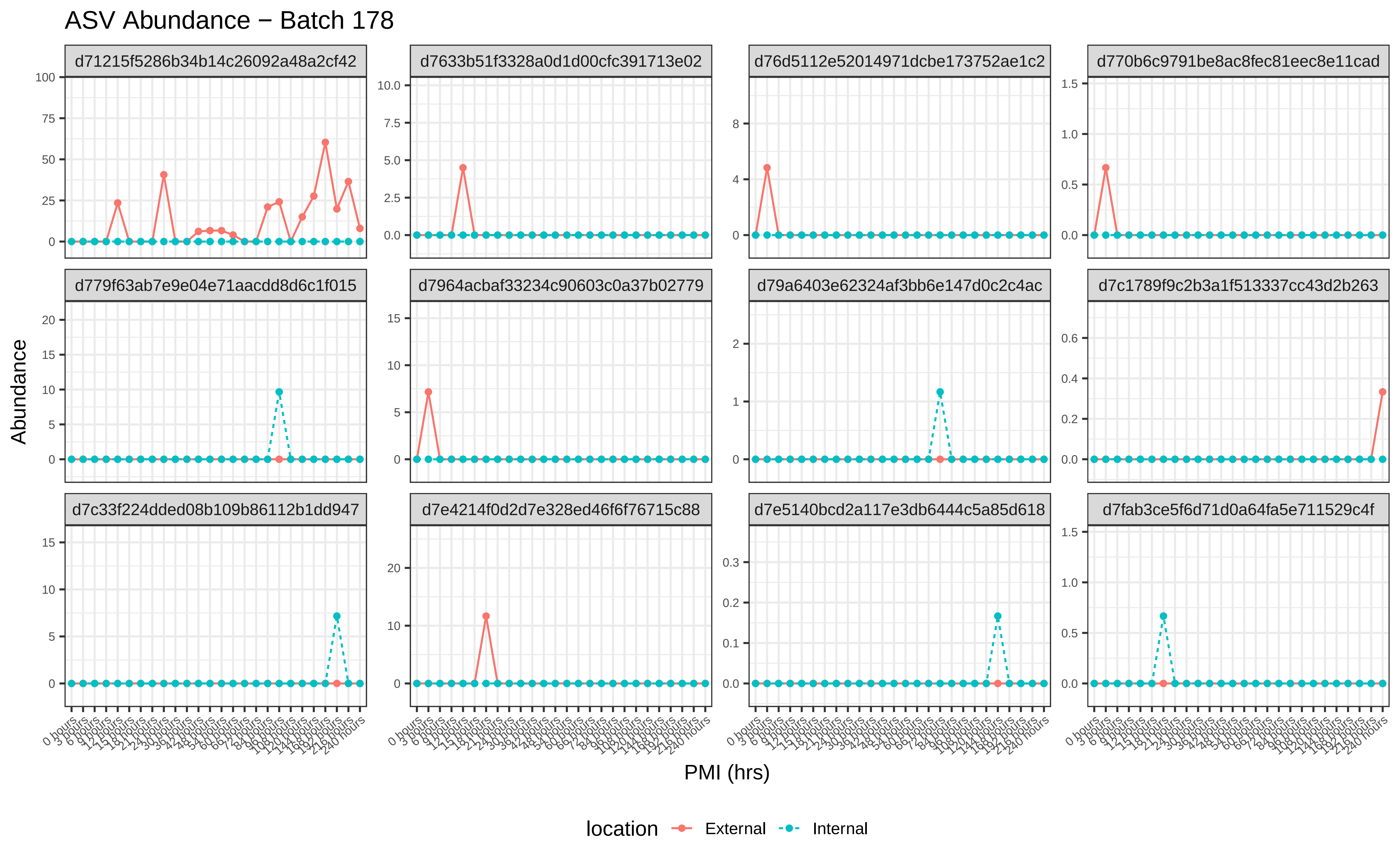
ASV Abundance − Batch 178
d71215f5286b34b14c26092a48a2cf42
d7633b51f3328a0d1d00cfc391713e02
d76d5112e52014971dcbe173752ae1c2
d770b6c9791be8ac8fec81eec8e11cad
d779f63ab7e9e04e71aacdd8d6c1f015
d7964acbaf33234c90603c0a37b02779
d79a6403e62324af3bb6e147d0c2c4ac
d7c1789f9c2b3a1f513337cc43d2b263
d7c33f224dded08b109b86112b1dd947
d7e4214f0d2d7e328ed46f6f76715c88
d7e5140bcd2a117e3db6444c5a85d618
d7fab3ce5f6d71d0a64fa5e711529c4f
Abundance
PMI (hrs)
location
External
Internal

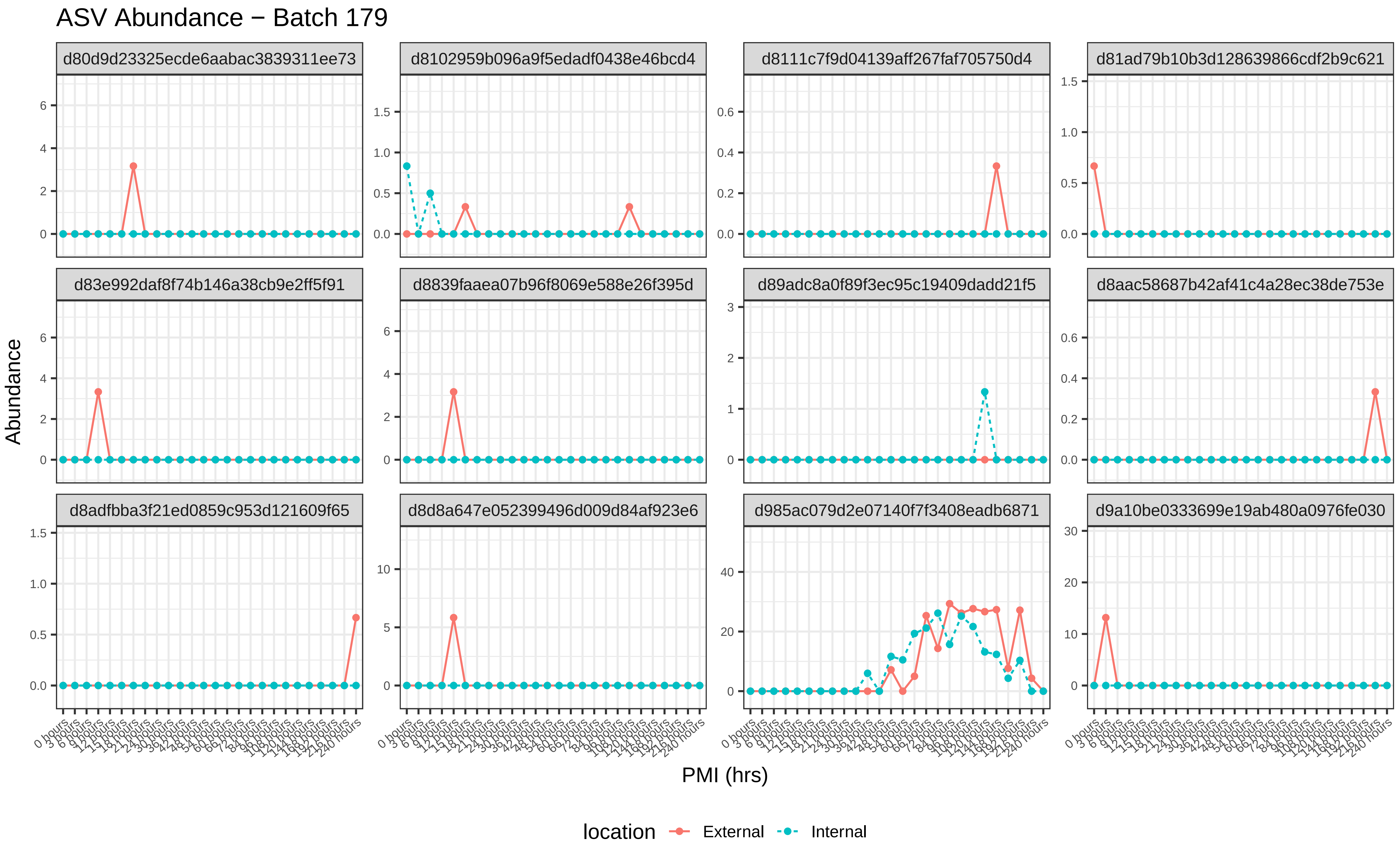
ASV Abundance − Batch 179
d80d9d23325ecde6aabac3839311ee73
d8102959b096a9f5edadf0438e46bcd4
d8111c7f9d04139aff267faf705750d4
d81ad79b10b3d128639866cdf2b9c621
d83e992daf8f74b146a38cb9e2ff5f91
d8839faaea07b96f8069e588e26f395d
d89adc8a0f89f3ec95c19409dadd21f5
d8aac58687b42af41c4a28ec38de753e
d8adfbba3f21ed0859c953d121609f65
d8d8a647e052399496d009d84af923e6
d985ac079d2e07140f7f3408eadb6871
d9a10be0333699e19ab480a0976fe030
Abundance
PMI (hrs)
location
External
Internal

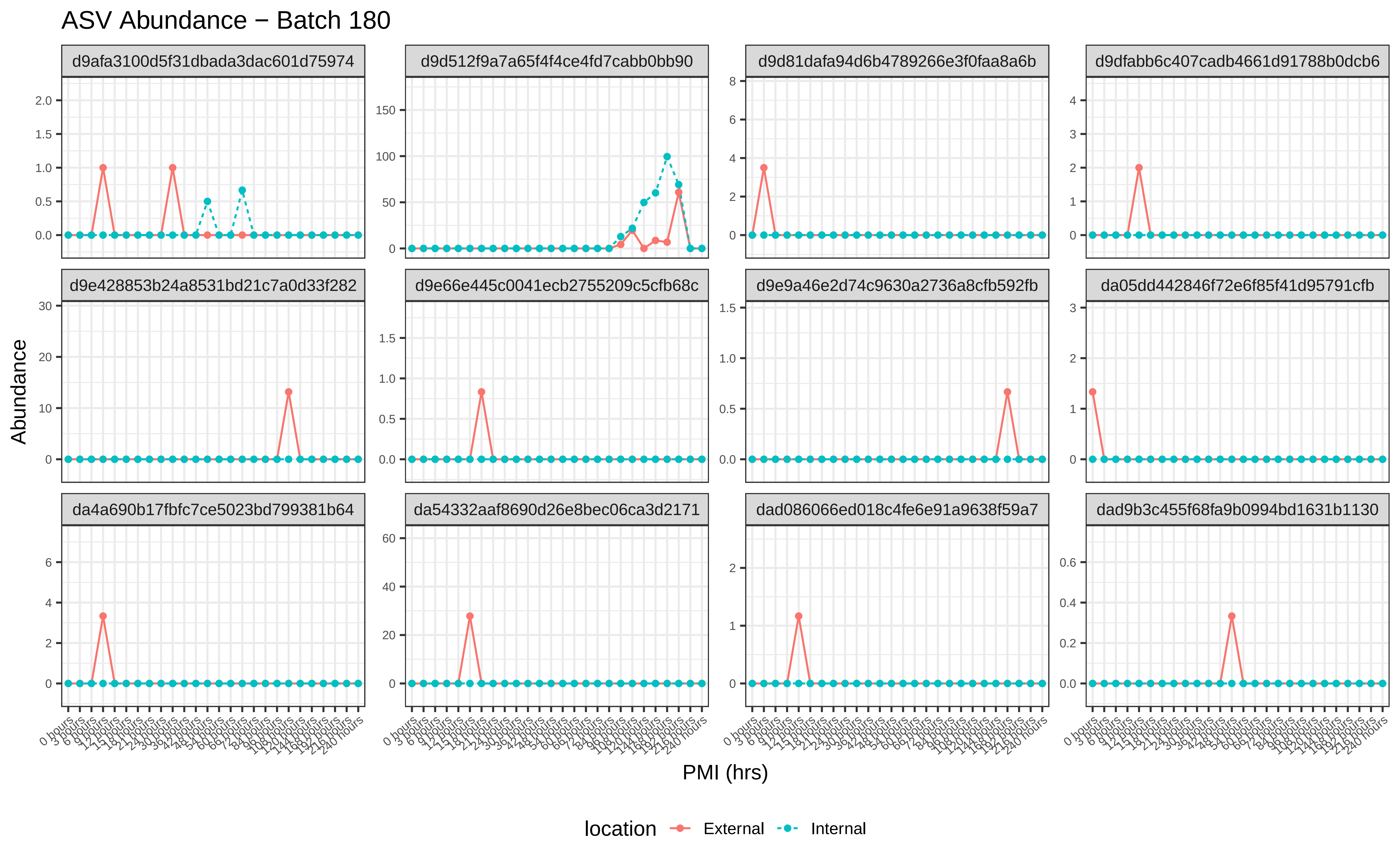
ASV Abundance − Batch 180
d9afa3100d5f31dbada3dac601d75974
d9d512f9a7a65f4f4ce4fd7cabb0bb90
d9d81dafa94d6b4789266e3f0faa8a6b
d9dfabb6c407cadb4661d91788b0dcb6
d9e428853b24a8531bd21c7a0d33f282
d9e66e445c0041ecb2755209c5cfb68c
d9e9a46e2d74c9630a2736a8cfb592fb
da05dd442846f72e6f85f41d95791cfb
da4a690b17fbfc7ce5023bd799381b64
da54332aaf8690d26e8bec06ca3d2171
dad086066ed018c4fe6e91a9638f59a7
dad9b3c455f68fa9b0994bd1631b1130
Abundance
PMI (hrs)
location
External
Internal

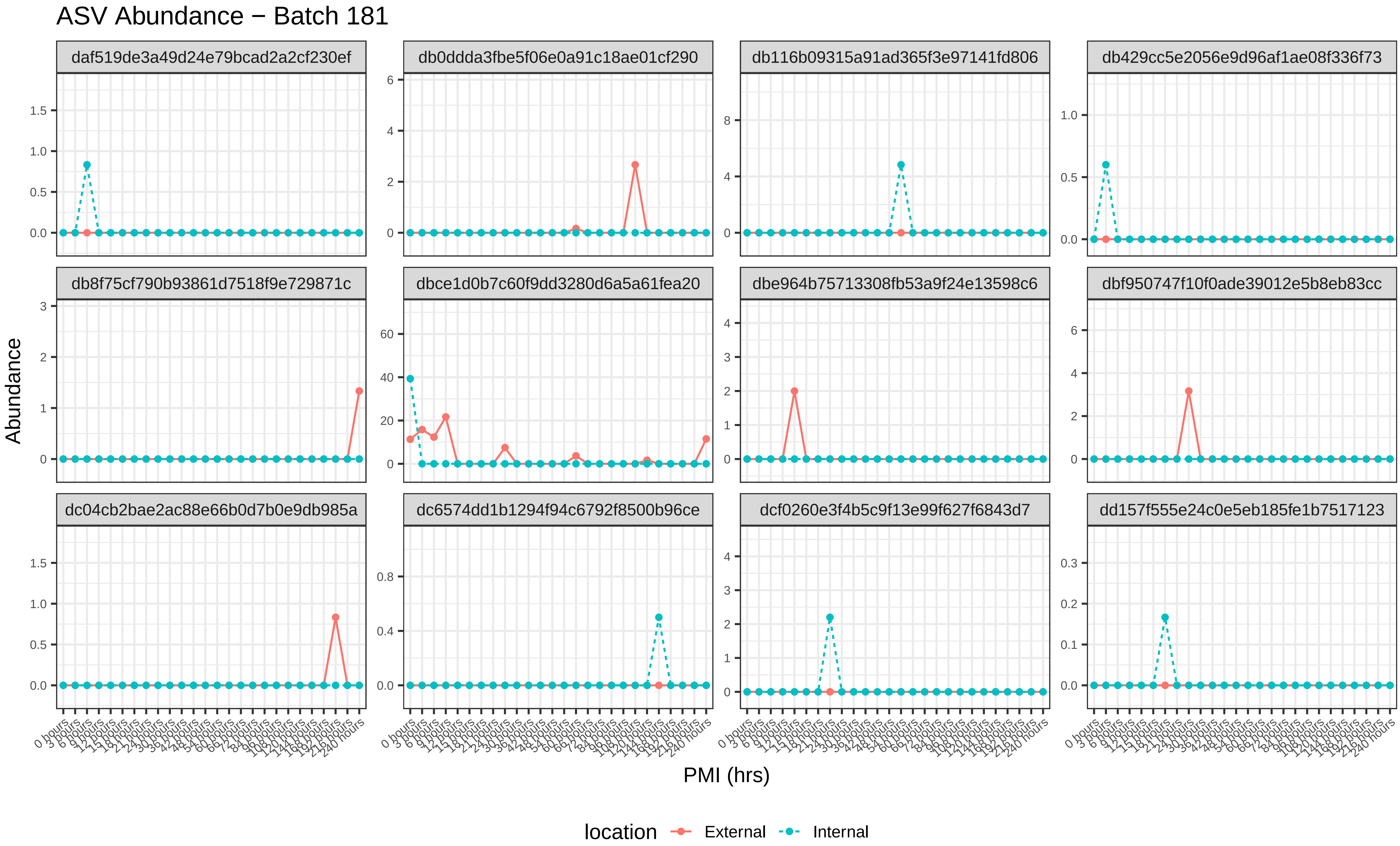
ASV Abundance − Batch 181
daf519de3a49d24e79bcad2a2cf230ef
db0ddda3fbe5f06e0a91c18ae01cf290
db116b09315a91ad365f3e97141fd806
db429cc5e2056e9d96af1ae08f336f73
db8f75cf790b93861d7518f9e729871c
dbce1d0b7c60f9dd3280d6a5a61fea20
dbe964b75713308fb53a9f24e13598c6
dbf950747f10f0ade39012e5b8eb83cc
dc04cb2bae2ac88e66b0d7b0e9db985a
dc6574dd1b1294f94c6792f8500b96ce
dcf0260e3f4b5c9f13e99f627f6843d7
dd157f555e24c0e5eb185fe1b7517123
Abundance
0 hours
3 hours
6 hours
9 hours
12 hours
15 hours
18 hours
21 hours
24 hours
30 hours
36 hours
42 hours
48 hours
54 hours
60 hours
66 hours
72 hours
84 hours
96 hours
108 hours
120 hours
144 hours
168 hours
192 hours
216 hours
240 hours
PMI (hrs)
location
External
Internal

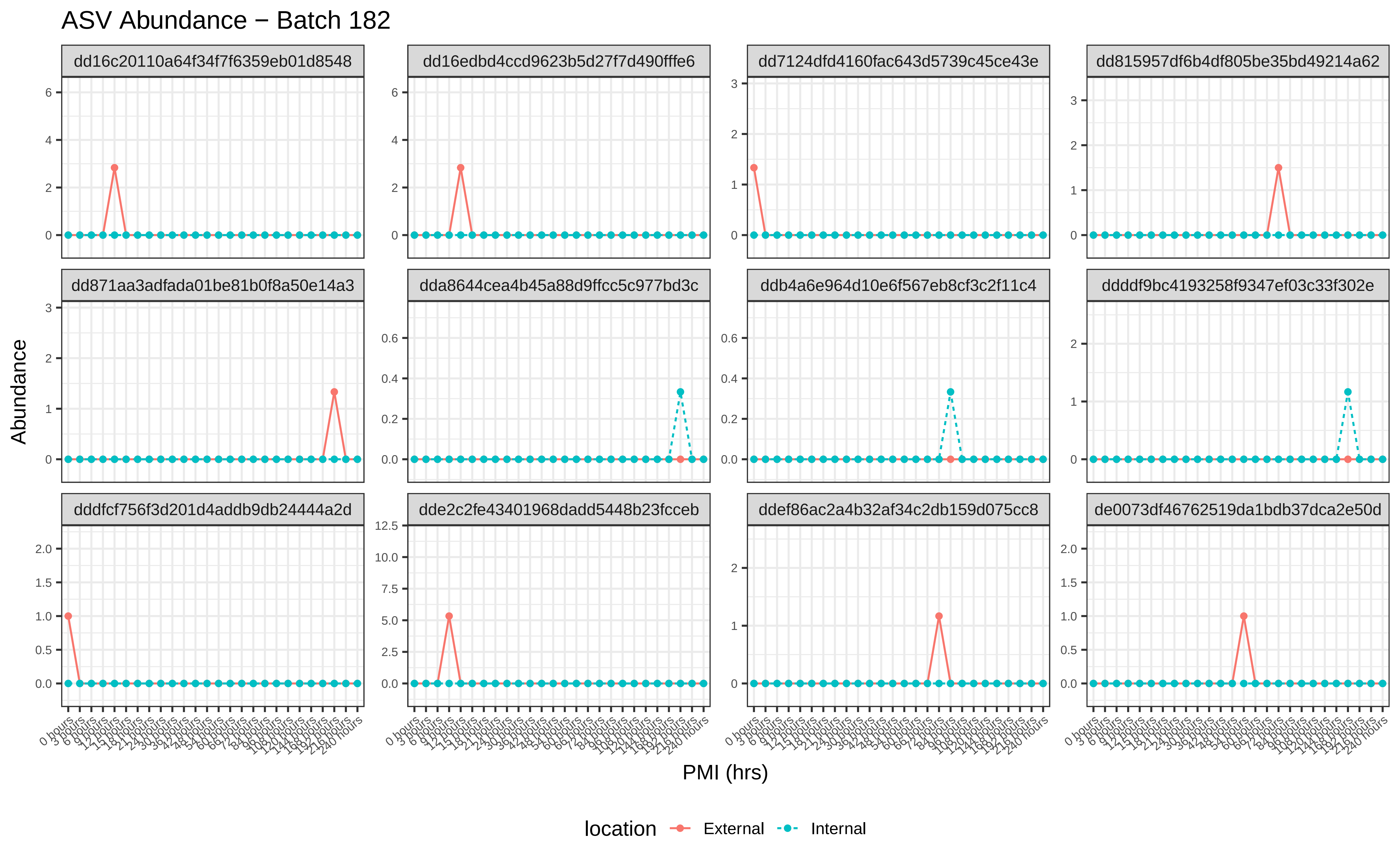

ASV Abundance − Batch 182
dd16c20110a64f34f7f6359eb01d8548
dd16edbd4ccd9623b5d27f7d490fffe6
dd7124dfd4160fac643d5739c45ce43e
dd815957df6b4df805be35bd49214a62
dd871aa3adfada01be81b0f8a50e14a3
dda8644cea4b45a88d9ffcc5c977bd3c
ddb4a6e964d10e6f567eb8cf3c2f11c4
ddddf9bc4193258f9347ef03c33f302e
dddfcf756f3d201d4addb9db24444a2d
dde2c2fe43401968dadd5448b23fcceb
ddef86ac2a4b32af34c2db159d075cc8
de0073df46762519da1bdb37dca2e50d
Abundance
PMI (hrs)
location
External
Internal

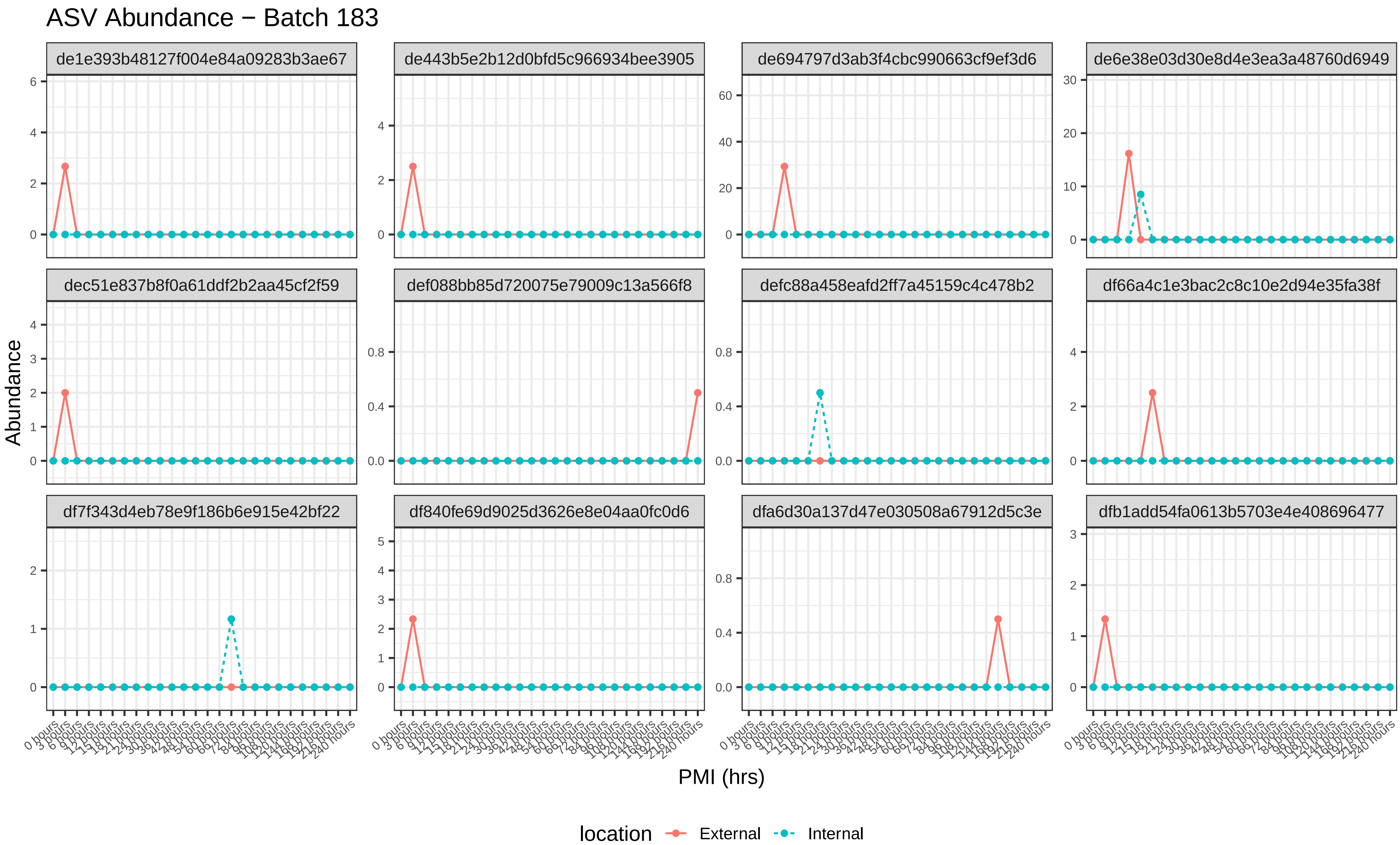
ASV Abundance − Batch 183
de1e393b48127f004e84a09283b3ae67
de443b5e2b12d0bfd5c966934bee3905
de694797d3ab3f4cbc990663cf9ef3d6
de6e38e03d30e8d4e3ea3a48760d6949
dec51e837b8f0a61ddf2b2aa45cf2f59
def088bb85d720075e79009c13a566f8
defc88a458eafd2ff7a45159c4c478b2
df66a4c1e3bac2c8c10e2d94e35fa38f
df7f343d4eb78e9f186b6e915e42bf22
df840fe69d9025d3626e8e04aa0fc0d6
dfa6d30a137d47e030508a67912d5c3e
dfb1add54fa0613b5703e4e408696477
Abundance
PMI (hrs)
location
External
Internal

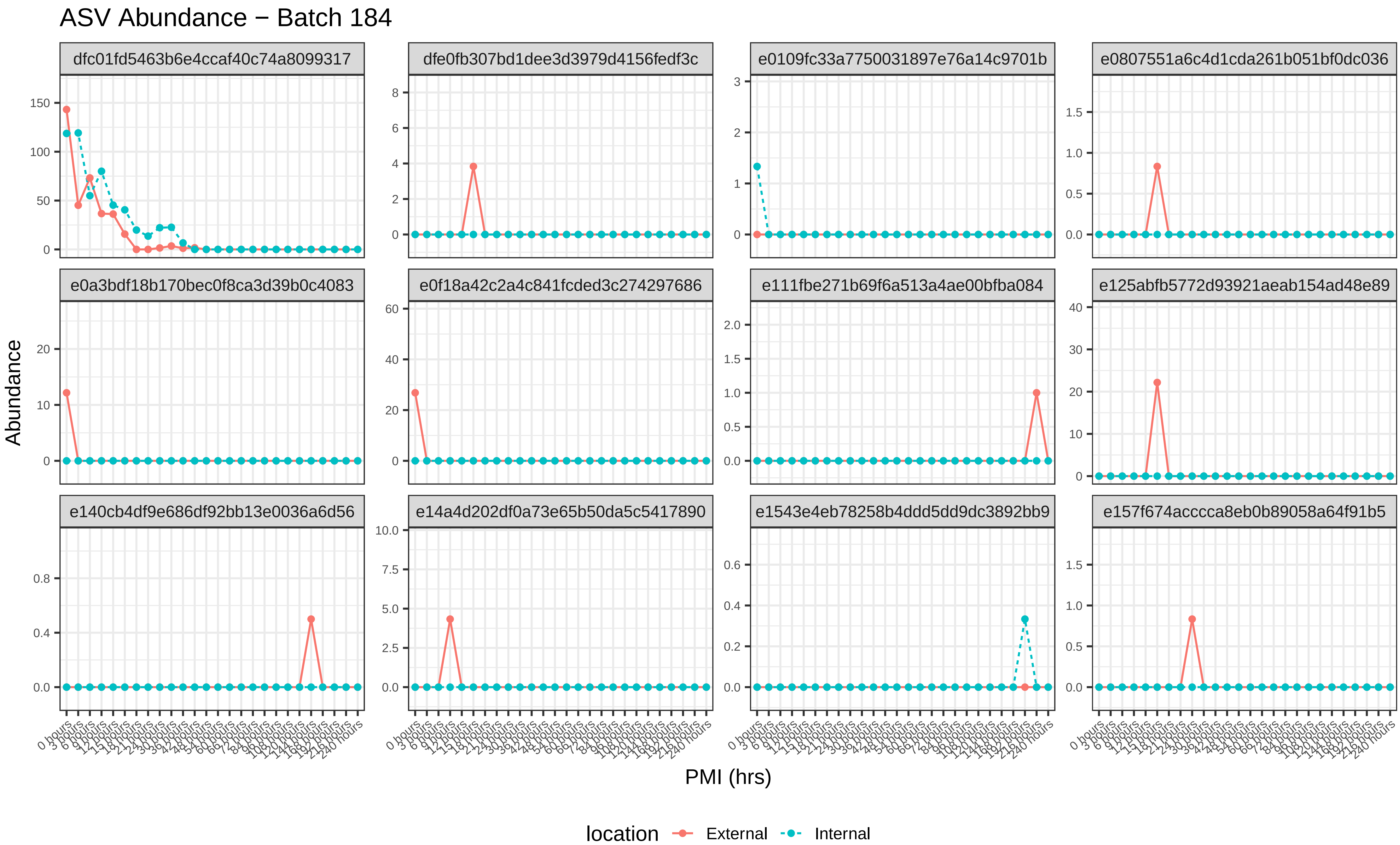
ASV Abundance − Batch 184
dfc01fd5463b6e4ccaf40c74a8099317
dfe0fb307bd1dee3d3979d4156fedf3c
e0109fc33a7750031897e76a14c9701b
e0807551a6c4d1cda261b051bf0dc036
e0a3bdf18b170bec0f8ca3d39b0c4083
e0f18a42c2a4c841fcded3c274297686
e111fbe271b69f6a513a4ae00bfba084
e125abfb5772d93921aeab154ad48e89
e140cb4df9e686df92bb13e0036a6d56
e14a4d202df0a73e65b50da5c5417890
e1543e4eb78258b4ddd5dd9dc3892bb9
e157f674accca8eb0b89058a64f91b5
Abundance
PMI (hrs)
location
External
Internal

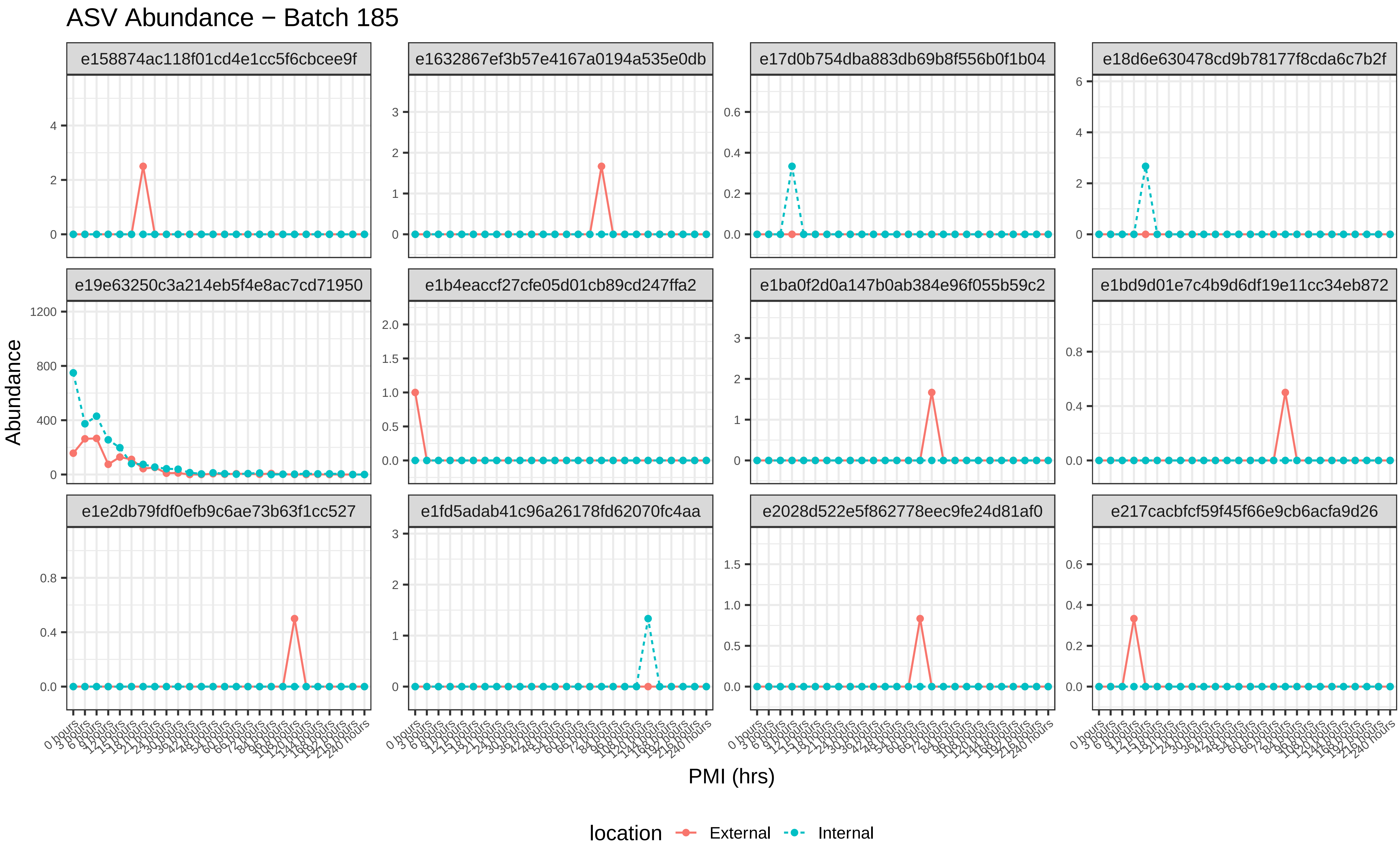
ASV Abundance − Batch 185
e158874ac118f01cd4e1cc5f6cbcee9f
e1632867ef3b57e4167a0194a535e0db
e17d0b754dba883db69b8f556b0f1b04
e18d6e630478cd9b78177f8cda6c7b2f
e19e63250c3a214eb5f4e8ac7cd71950
e1b4eaccf27cfe05d01cb89cd247ffa2
e1ba0f2d0a147b0ab384e96f055b59c2
e1bd9d01e7c4b9d6df19e11cc34eb872
e1e2db79fdf0efb9c6ae73b63f1cc527
e1fd5adab41c96a26178fd62070fc4aa
e2028d522e5f862778eec9fe24d81af0
e217cacbfcf59f45f66e9cb6acfa9d26
Abundance
PMI (hrs)
location
External
Internal

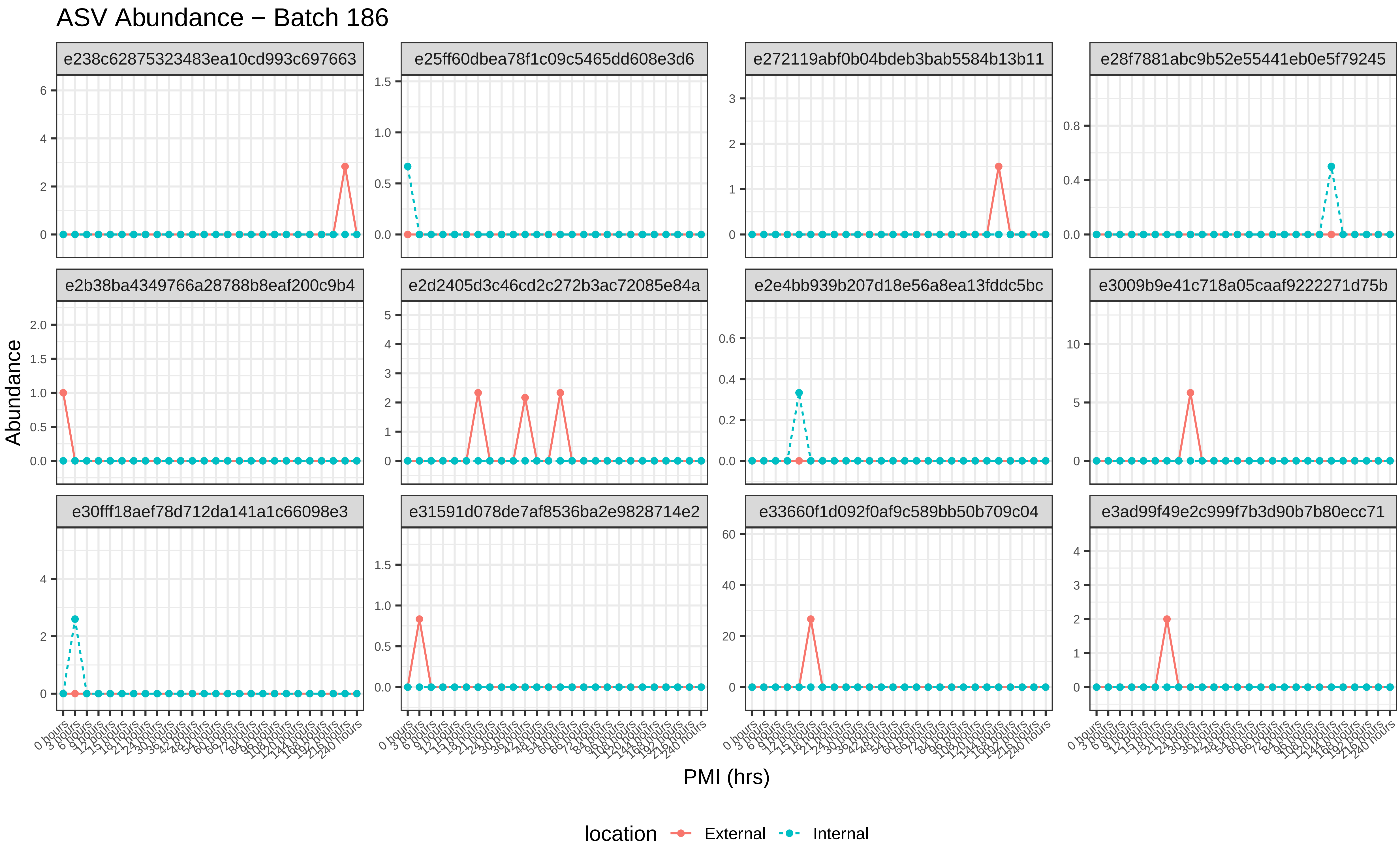
ASV Abundance − Batch 186
e238c62875323483ea10cd993c697663
e25ff60dbea78f1c09c5465dd608e3d6
e272119abf0b04bdeb3bab5584b13b11
e28f7881abc9b52e55441eb0e5f79245
e2b38ba4349766a28788b8eaf200c9b4
e2d2405d3c46cd2c272b3ac72085e84a
e2e4bb939b207d18e56a8ea13fddc5bc
e3009b9e41c718a05caaf9222271d75b
e30fff18aef78d712da141a1c66098e3
e31591d078de7af8536ba2e9828714e2
e33660f1d092f0af9c589bb50b709c04
e3ad99f49e2c999f7b3d90b7b80ecc71
Abundance
PMI (hrs)
location
External
Internal

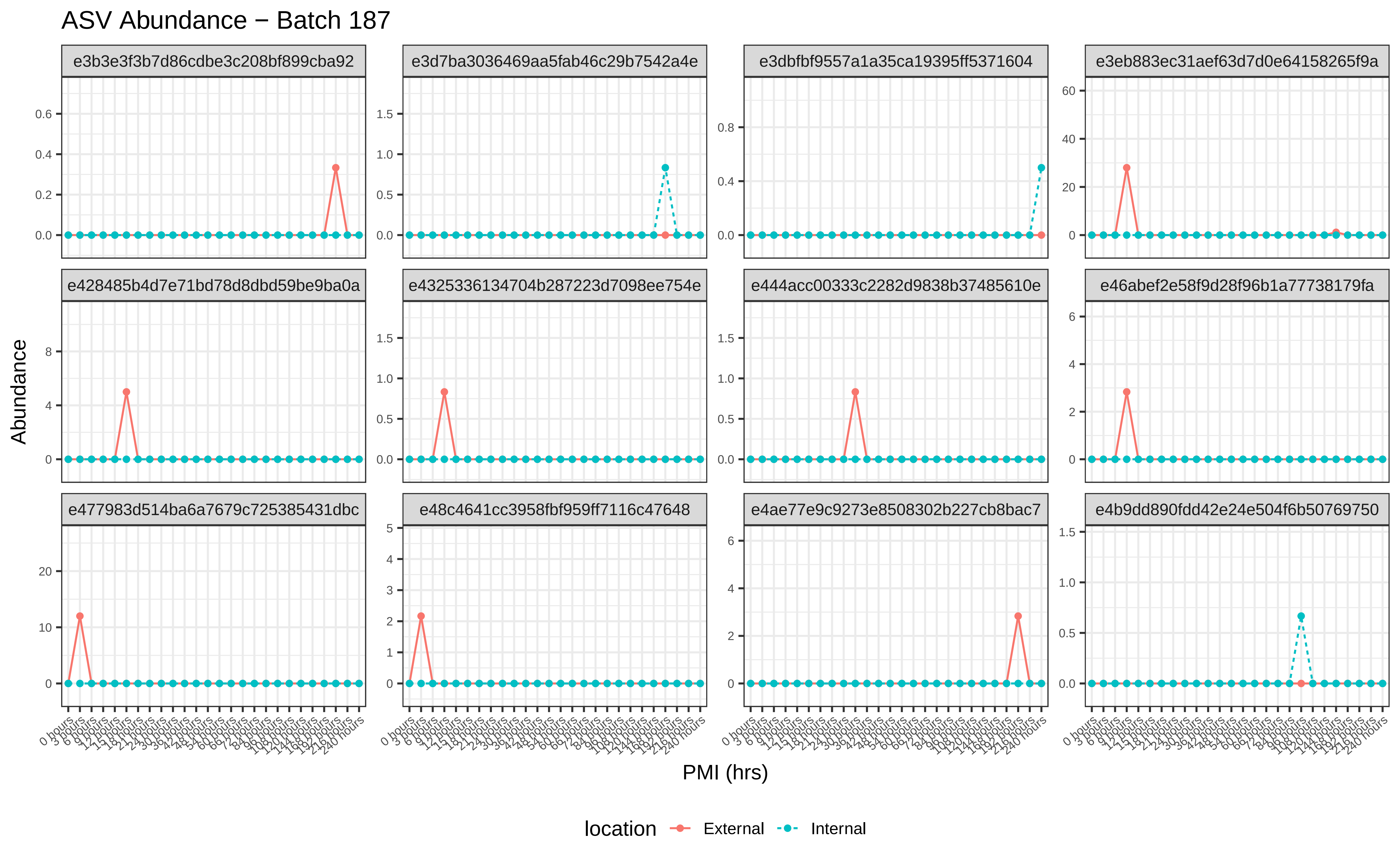
ASV Abundance − Batch 187
e3b3e3f3b7d86cdbe3c208bf899cba92
e3d7ba3036469aa5fab46c29b7542a4e
e3dbfbf9557a1a35ca19395ff5371604
e3eb883ec31aef63d7d0e64158265f9a
e428485b4d7e71bd78d8dbd59be9ba0a
e4325336134704b287223d7098ee754e
e444acc00333c2282d9838b37485610e
e46abef2e58f9d28f96b1a77738179fa
e477983d514ba6a7679c725385431dbc
e48c4641cc3958fbf959ff7116c47648
e4ae77e9c9273e8508302b227cb8bac7
e4b9dd890fdd42e24e504f6b50769750
Abundance
PMI (hrs)
location
External
Internal

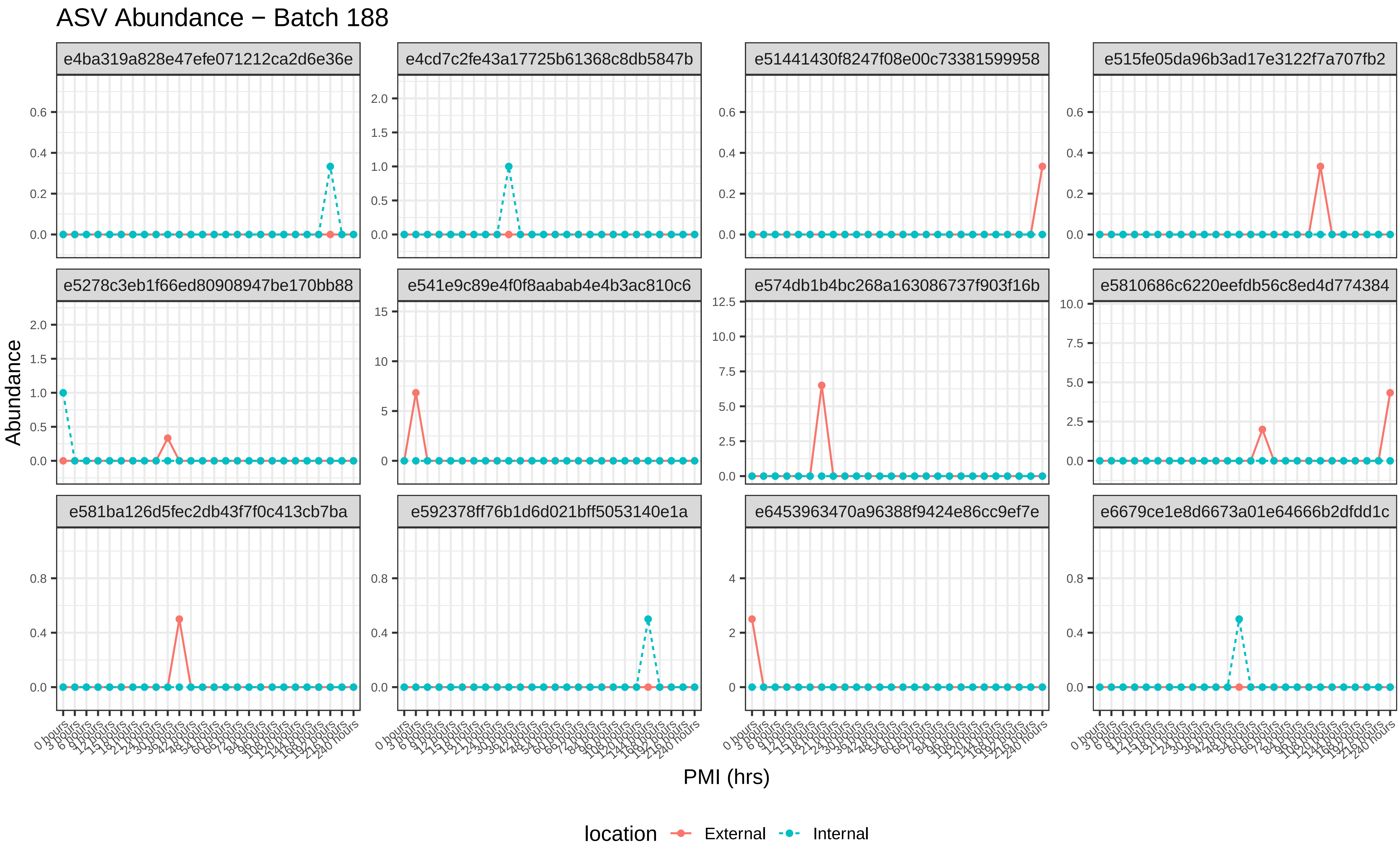

ASV Abundance − Batch 188
e4ba319a828e47efe071212ca2d6e36e
e4cd7c2fe43a17725b61368c8db5847b
e51441430f8247f08e00c73381599958
e515fe05da96b3ad17e3122f7a707fb2
e5278c3eb1f66ed80908947be170bb88
e541e9c89e4f0f8aabab4e4b3ac810c6
e574db1b4bc268a163086737f903f16b
e5810686c6220eefdb56c8ed4d774384
e581ba126d5fec2db43f7f0c413cb7ba
e592378ff76b1d6d021bff5053140e1a
e6453963470a96388f9424e86cc9ef7e
e6679ce1e8d6673a01e64666b2dfdd1c
Abundance
PMI (hrs)
location
External
Internal

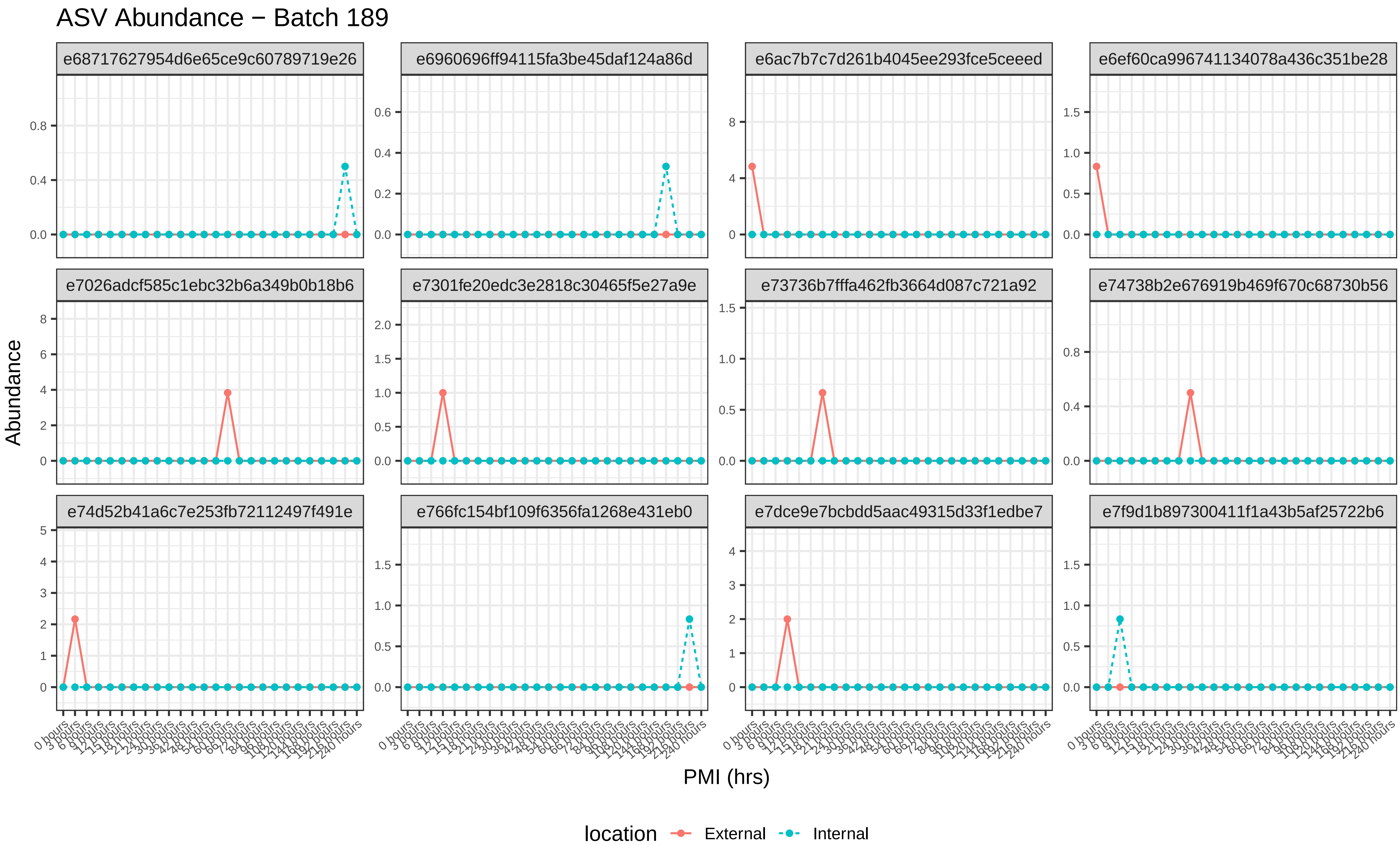

ASV Abundance − Batch 189
e68717627954d6e65ce9c60789719e26
e6960696ff94115fa3be45daf124a86d
e6ac7b7c7d261b4045ee293fce5ceeed
e6ef60ca996741134078a436c351be28
e7026adcf585c1ebc32b6a349b0b18b6
e7301fe20edc3e2818c30465f5e27a9e
e73736b7fffa462fb3664d087c721a92
e74738b2e676919b469f670c68730b56
e74d52b41a6c7e253fb72112497f491e
e766fc154bf109f6356fa1268e431eb0
e7dce9e7bcbdd5aac49315d33f1edbe7
e7f9d1b897300411f1a43b5af25722b6
Abundance
PMI (hrs)
location
External
Internal

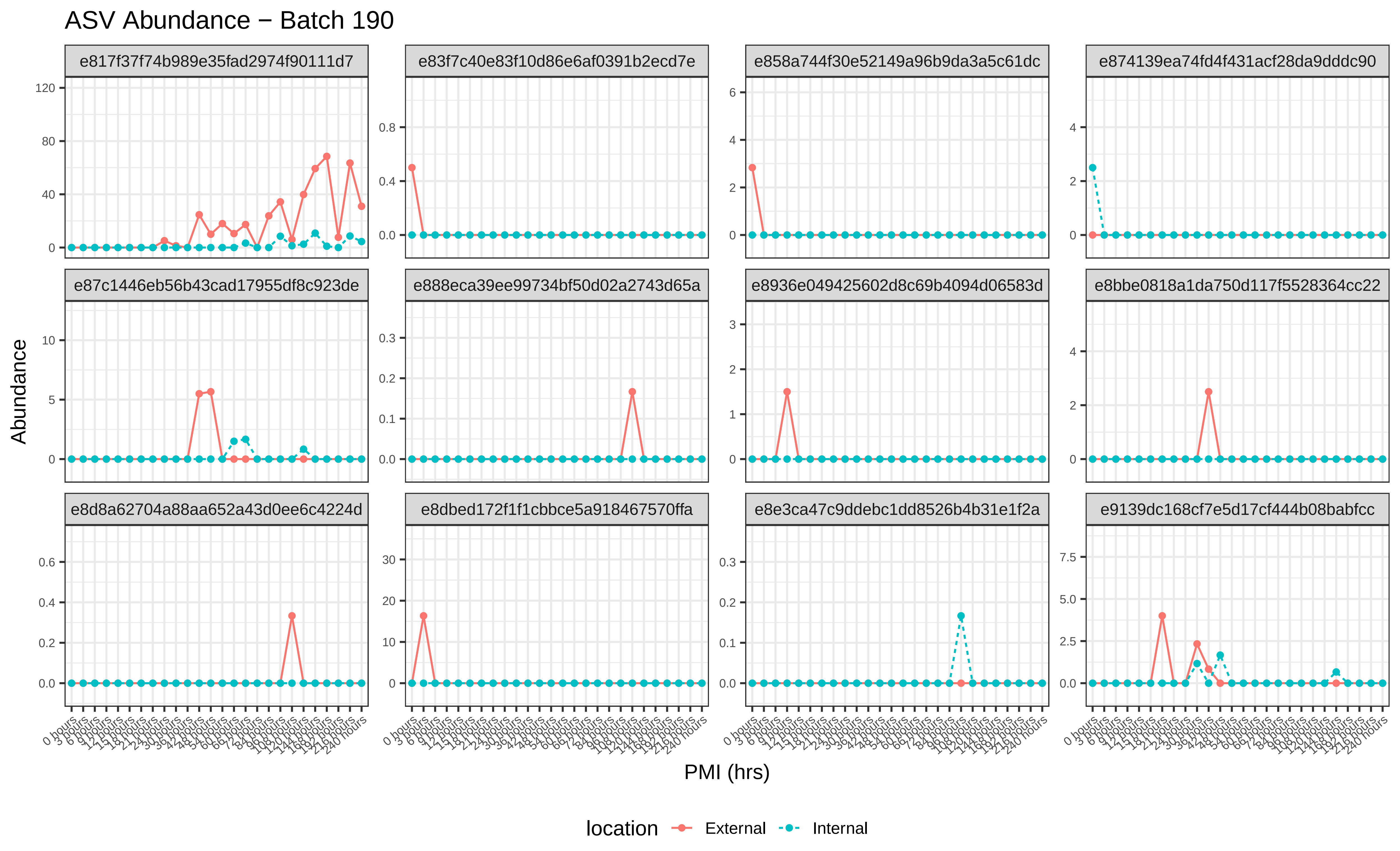

ASV Abundance − Batch 190
e817f37f74b989e35fad2974f90111d7
e83f7c40e83f10d86e6af0391b2ecd7e
e858a744f30e52149a96b9da3a5c61dc
e874139ea74fd4f431acf28da9dddc90
e87c1446eb56b43cad17955df8c923de
e888eca39ee99734bf50d02a2743d65a
e8936e049425602d8c69b4094d06583d
e8bbe0818a1da750d117f5528364cc22
e8d8a62704a88aa652a43d0ee6c4224d
e8dbed172f1f1cbbce5a918467570ffa
e8e3ca47c9ddebc1dd8526b4b31e1f2a
e9139dc168cf7e5d17cf444b08babfcc
Abundance
PMI (hrs)
location
External
Internal

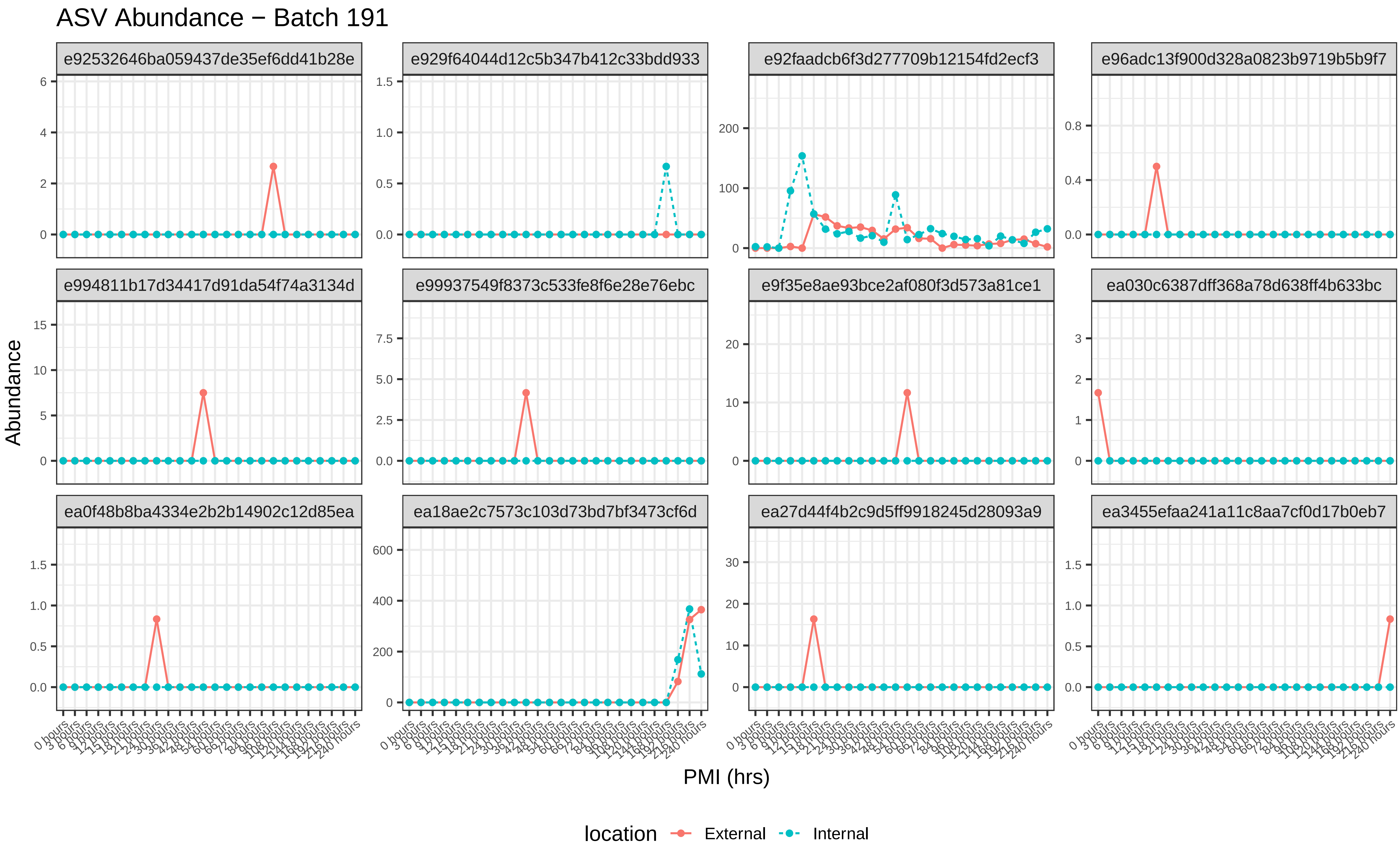

ASV Abundance − Batch 191
e92532646ba059437de35ef6dd41b28e
e929f64044d12c5b347b412c33bdd933
e92faadcb6f3d277709b12154fd2ecf3
e96adc13f900d328a0823b9719b5b9f7
e994811b17d34417d91da54f74a3134d
e99937549f8373c533fe8f6e28e76ebc
e9f35e8ae93bce2af080f3d573a81ce1
ea030c6387dff368a78d638ff4b633bc
ea0f48b8ba4334e2b2b14902c12d85ea
ea18ae2c7573c103d73bd7bf3473cf6d
ea27d44f4b2c9d5ff9918245d28093a9
ea3455efaa241a11c8aa7cf0d17b0eb7
Abundance
PMI (hrs)
location
External
Internal

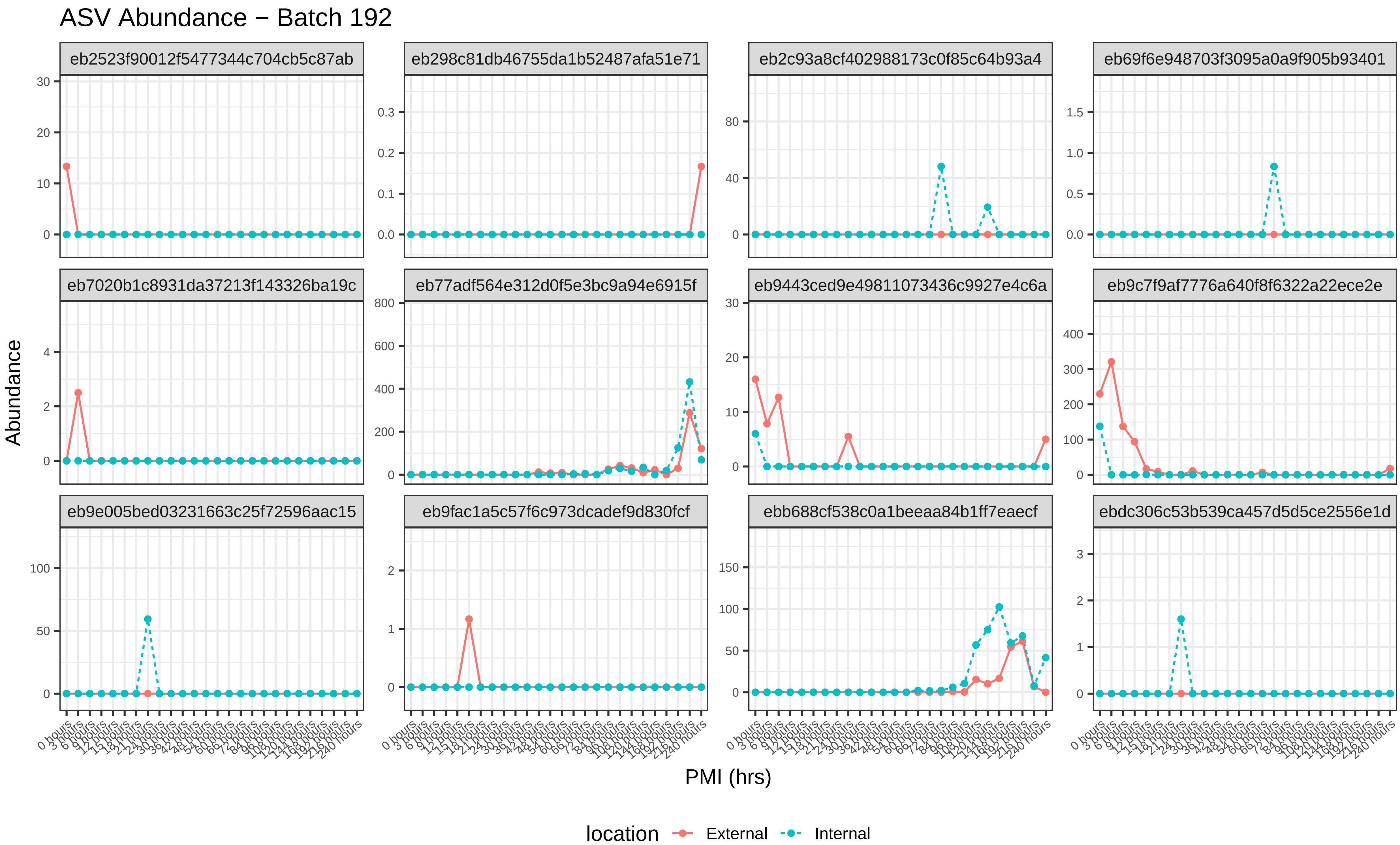

ASV Abundance − Batch 192
eb2523f90012f5477344c704cb5c87ab
eb298c81db46755da1b52487afa51e71
eb2c93a8cf402988173c0f85c64b93a4
eb69f6e948703f3095a0a9f905b93401
eb7020b1c8931da37213f143326ba19c
eb77adf564e312d0f5e3bc9a94e6915f
eb9443ced9e49811073436c9927e4c6a
eb9c7f9af7776a640f8f6322a22ece2e
eb9e005bed03231663c25f72596aac15
eb9fac1a5c57f6c973dcadef9d830fcf
ebb688cf538c0a1beeaa84b1ff7eaecf
ebdc306c53b539ca457d5d5ce2556e1d
Abundance
PMI (hrs)
location
External
Internal

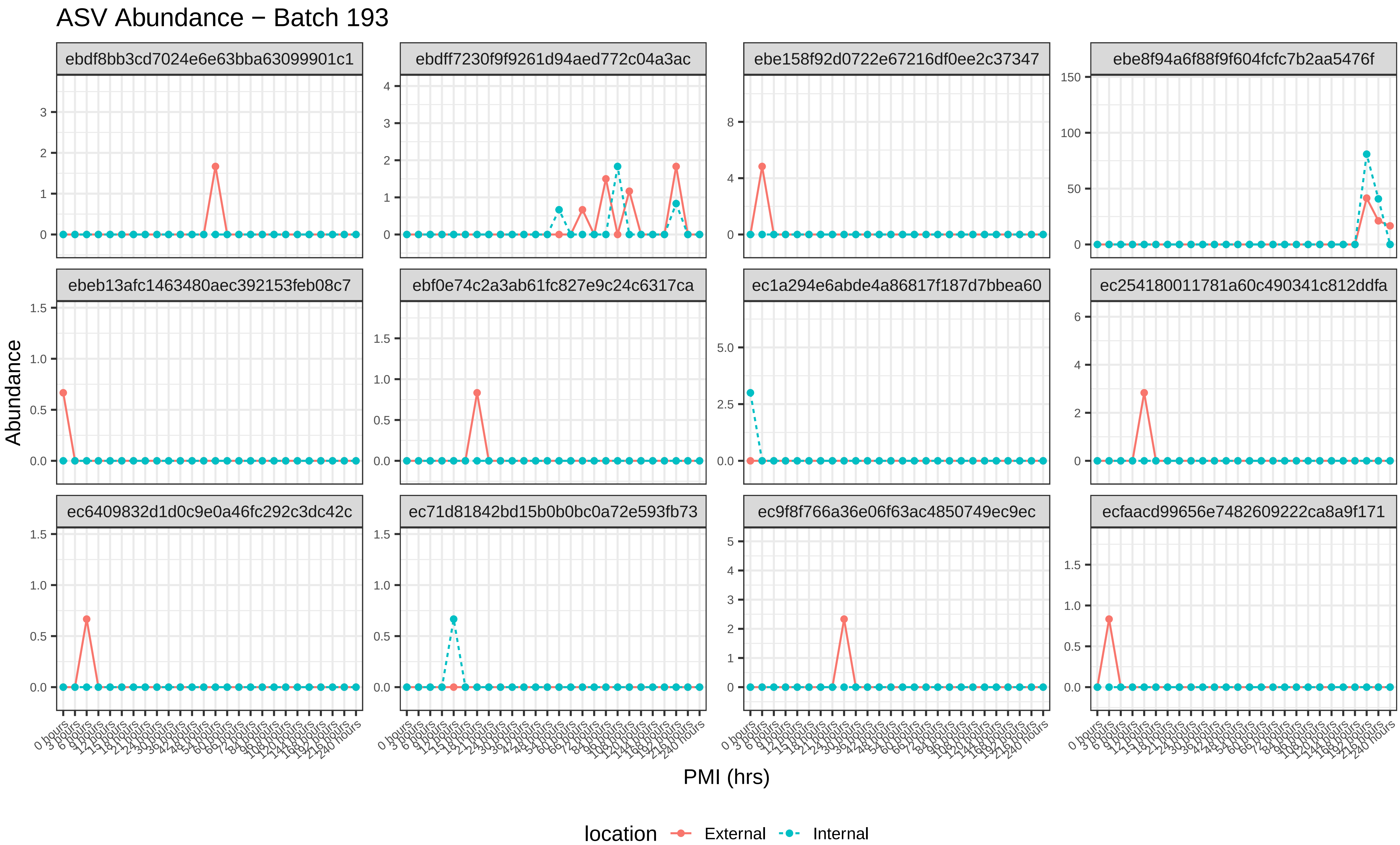

ASV Abundance − Batch 193
ebdf8bb3cd7024e6e63bba63099901c1
ebdff7230f9f9261d94aed772c04a3ac
ebe158f92d0722e67216df0ee2c37347
ebe8f94a6f88f9f604fcfc7b2aa5476f
ebeb13afc1463480aec392153feb08c7
ebf0e74c2a3ab61fc827e9c24c6317ca
ec1a294e6abde4a86817f187d7bbea60
ec254180011781a60c490341c812ddfa
ec6409832d1d0c9e0a46fc292c3dc42c
ec71d81842bd15b0b0bc0a72e593fb73
ec9f8f766a36e06f63ac4850749ec9ec
ecfaacd99656e7482609222ca8a9f171
Abundance
PMI (hrs)
location
External
Internal

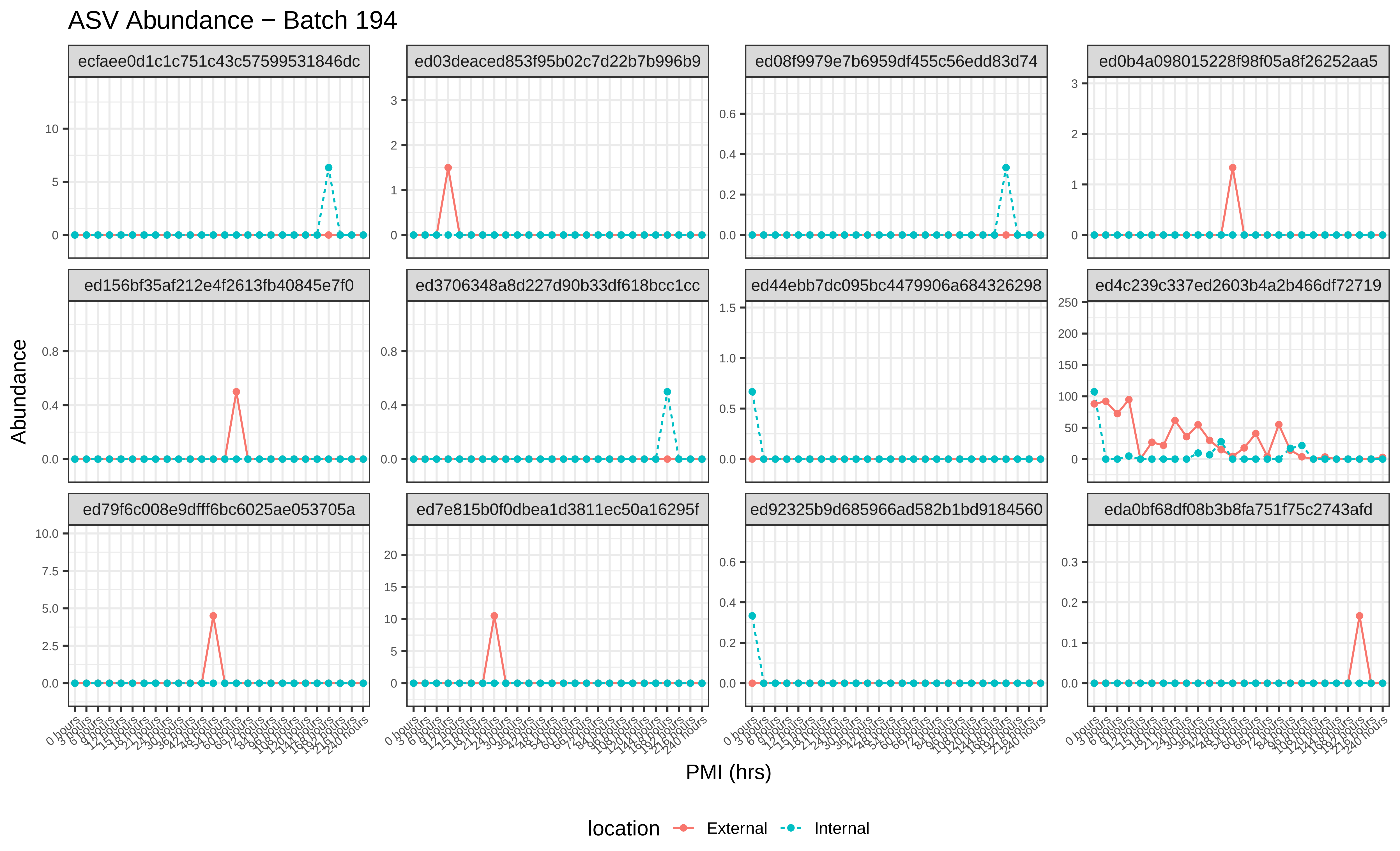

ASV Abundance − Batch 194
ecfaee0d1c1c751c43c57599531846dc
ed03deaced853f95b02c7d22b7b996b9
ed08f9979e7b6959df455c56edd83d74
ed0b4a098015228f98f05a8f26252aa5
ed156bf35af212e4f2613fb40845e7f0
ed3706348a8d227d90b33df618bcc1cc
ed44ebb7dc095bc4479906a684326298
ed4c239c337ed2603b4a2b466df72719
ed79f6c008e9dfff6bc6025ae053705a
ed7e815b0f0dbea1d3811ec50a16295f
ed92325b9d685966ad582b1bd9184560
eda0bf68df08b3b8fa751f75c2743afd
Abundance
PMI (hrs)
location
External
Internal

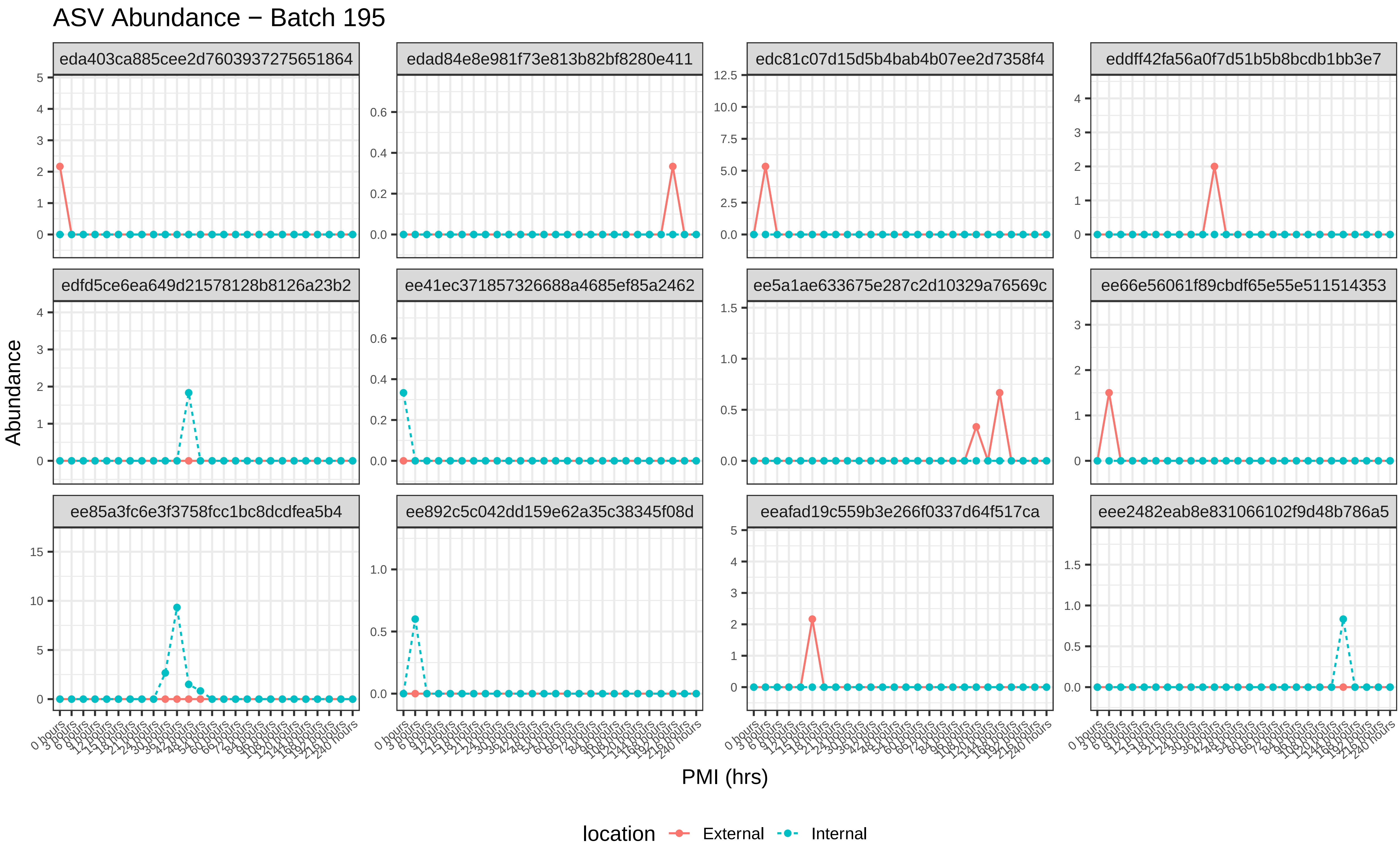

ASV Abundance − Batch 195
eda403ca885cee2d7603937275651864
edad84e8e981f73e813b82bf8280e411
edc81c07d15d5b4bab4b07ee2d7358f4
eddff42fa56a0f7d51b5b8bcdb1bb3e7
edfd5ce6ea649d21578128b8126a23b2
ee41ec371857326688a4685ef85a2462
ee5a1ae633675e287c2d10329a76569c
ee66e56061f89cbdf65e55e511514353
ee85a3fc6e3f3758fcc1bc8dcdfea5b4
ee892c5c042dd159e62a35c38345f08d
eeafad19c559b3e266f0337d64f517ca
eee2482eab8e831066102f9d48b786a5
Abundance
PMI (hrs)
location
External
Internal

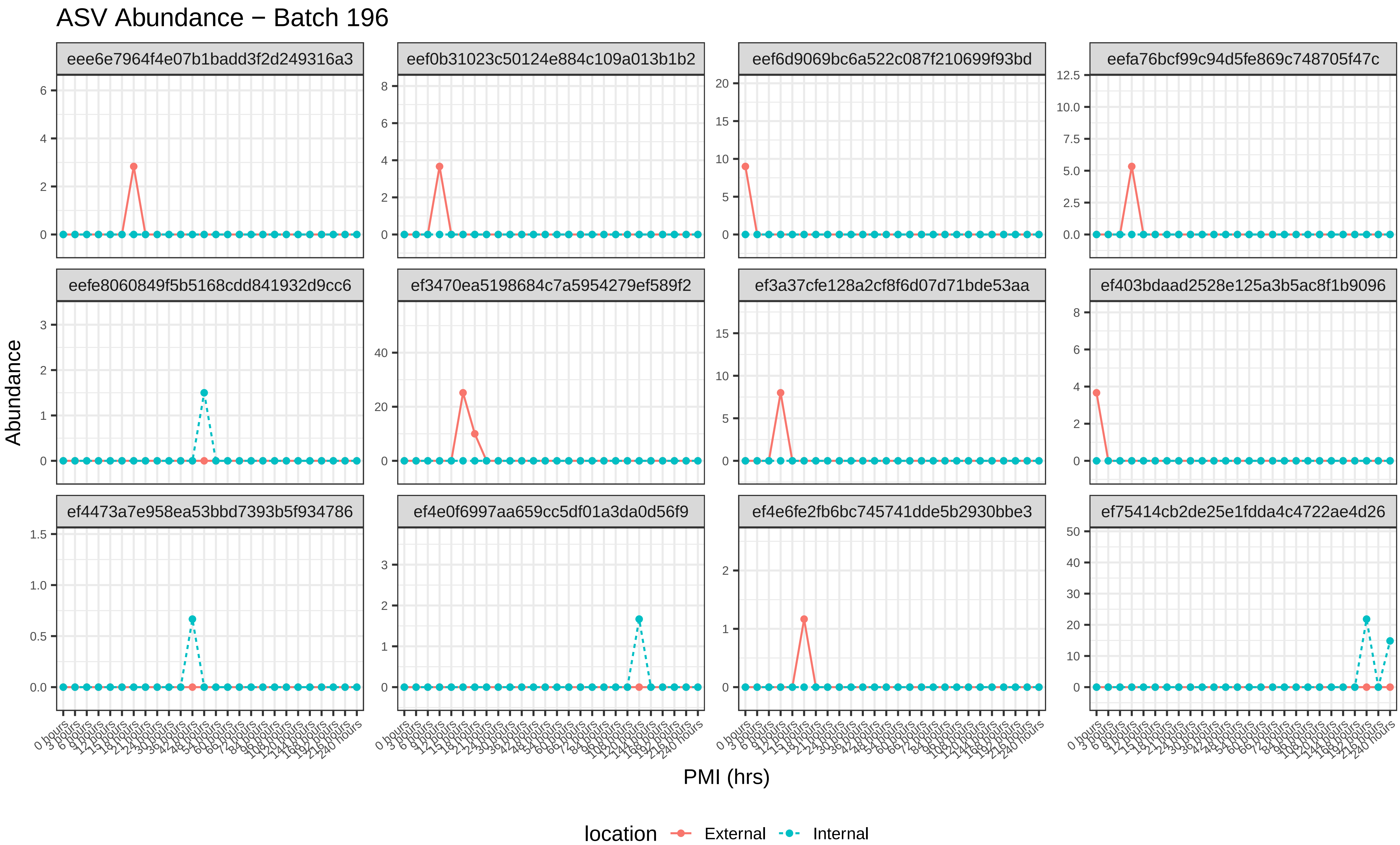

ASV Abundance − Batch 196
eee6e7964f4e07b1badd3f2d249316a3
eef0b31023c50124e884c109a013b1b2
eef6d9069bc6a522c087f210699f93bd
eefa76bcf99c94d5fe869c748705f47c
eefe8060849f5b5168cdd841932d9cc6
ef3470ea5198684c7a5954279ef589f2
ef3a37cfe128a2cf8f6d07d71bde53aa
ef403bdaad2528e125a3b5ac8f1b9096
ef4473a7e958ea53bbd7393b5f934786
ef4e0f6997aa659cc5df01a3da0d56f9
ef4e6fe2fb6bc745741dde5b2930bbe3
ef75414cb2de25e1fdda4c4722ae4d26
Abundance
PMI (hrs)
location
External
Internal

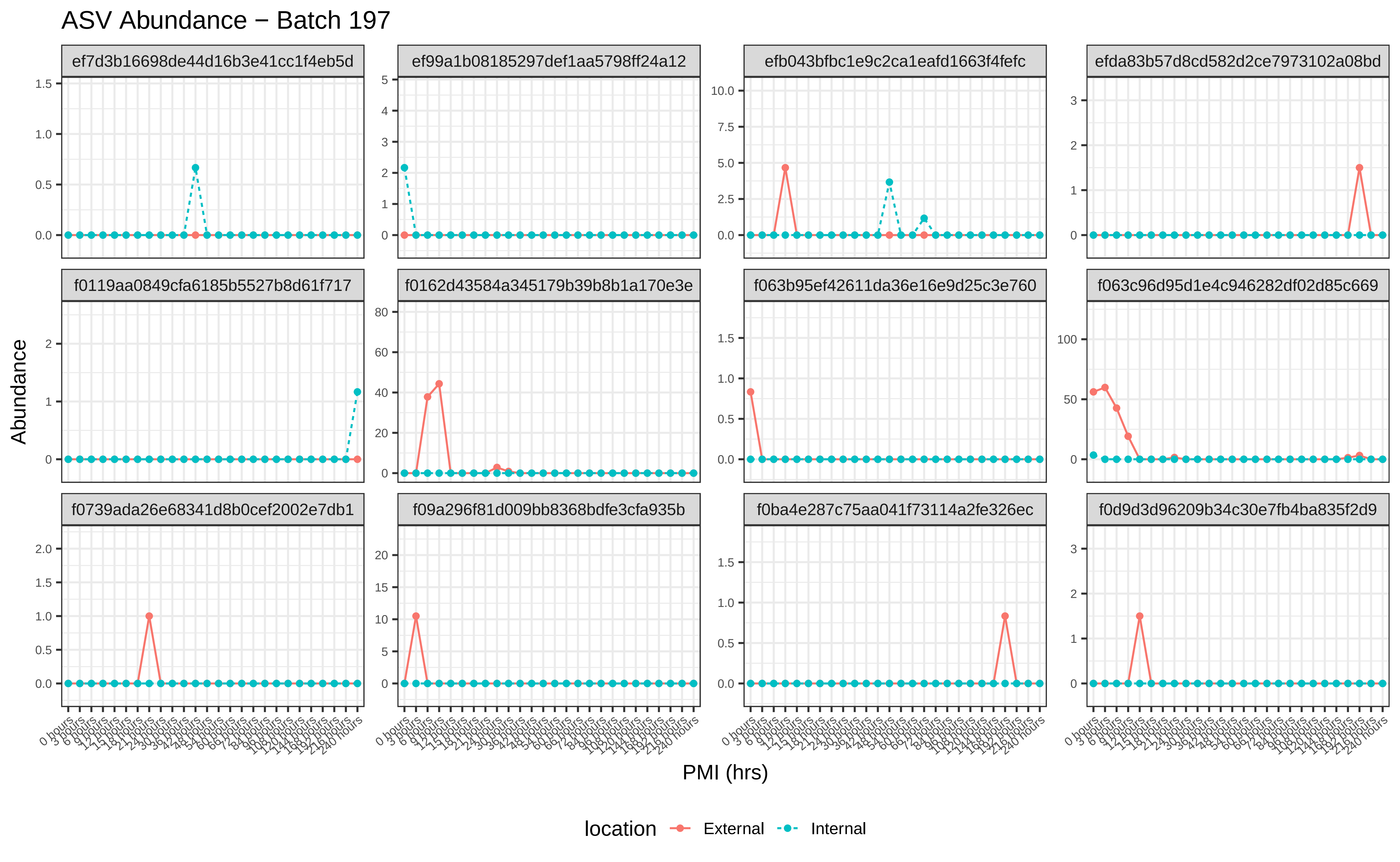
ASV Abundance − Batch 197
ef7d3b16698de44d16b3e41cc1f4eb5d
ef99a1b08185297def1aa5798ff24a12
efb043bfbc1e9c2ca1eafd1663f4fefc
efda83b57d8cd582d2ce7973102a08bd
f0119aa0849cfa6185b5527b8d61f717
f0162d43584a345179b39b8b1a170e3e
f063b95ef42611da36e16e9d25c3e760
f063c96d95d1e4c946282df02d85c669
f0739ada26e68341d8b0cef2002e7db1
f09a296f81d009bb8368bdfe3cfa935b
f0ba4e287c75aa041f73114a2fe326ec
f0d9d3d96209b34c30e7fb4ba835f2d9
Abundance
PMI (hrs)
location
External
Internal

ASV Abundance − Batch 198

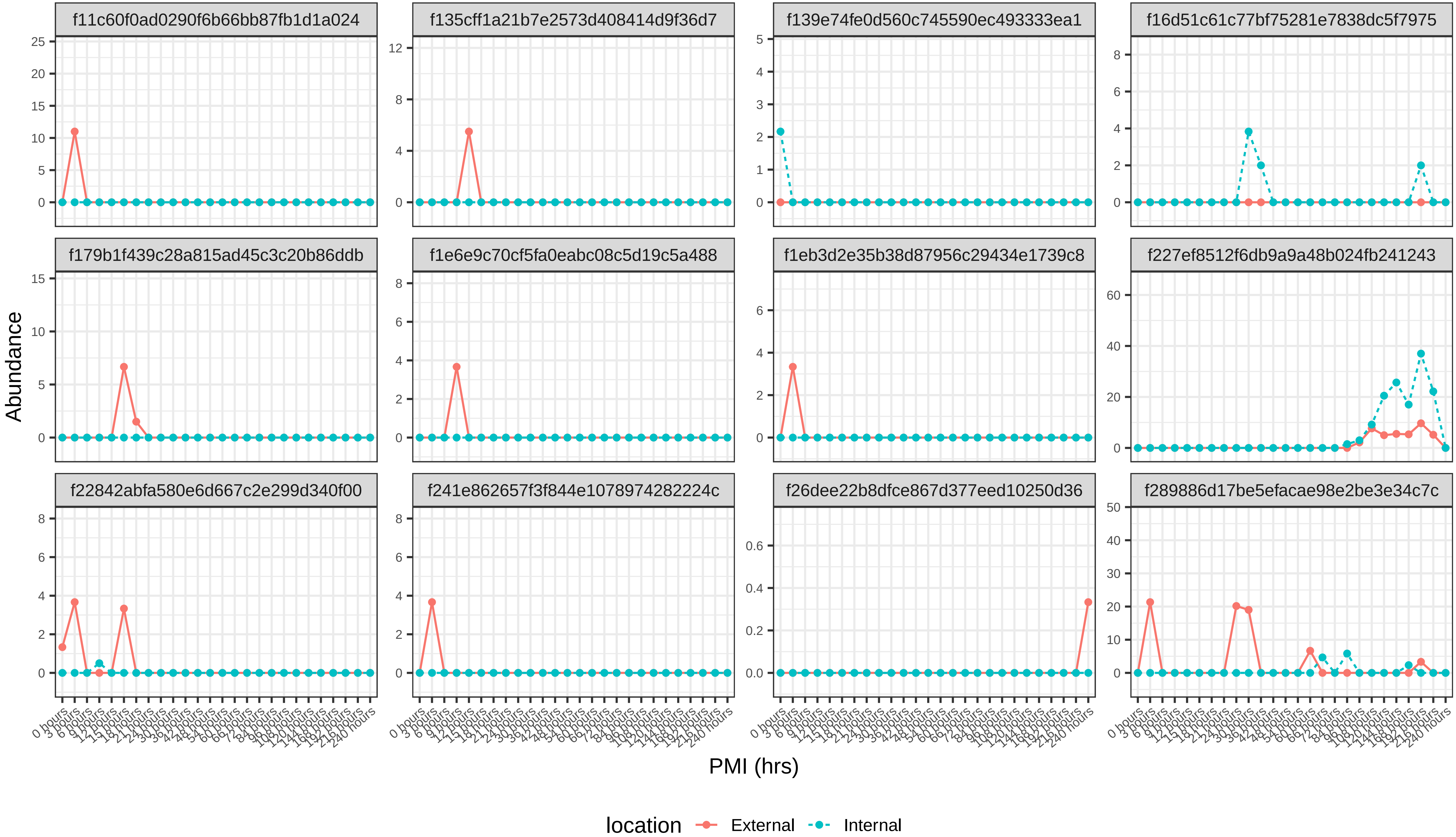

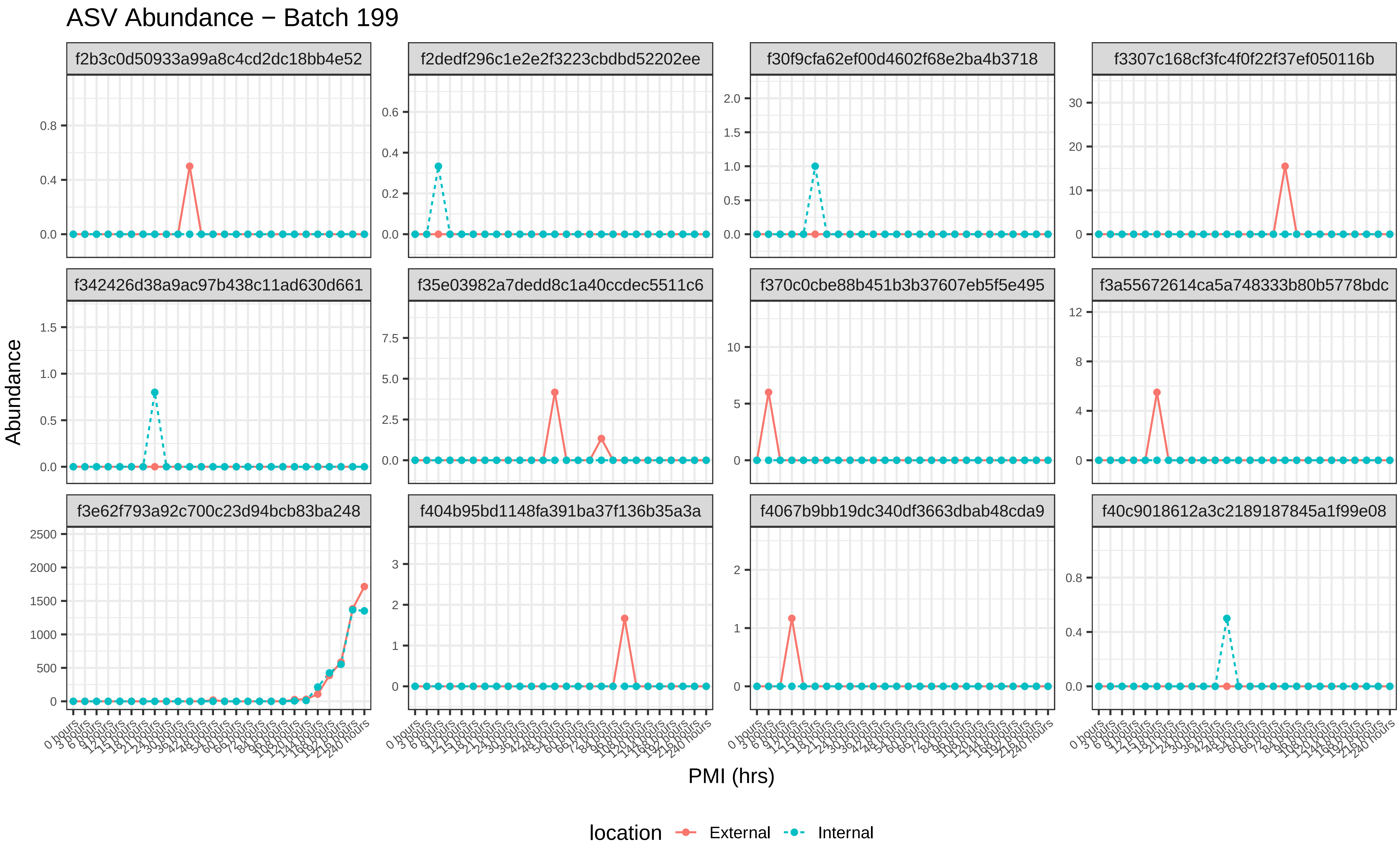
ASV Abundance − Batch 199
f2b3c0d50933a99a8c4cd2dc18bb4e52
f2dedf296c1e2e2f3223cbdbd52202ee
f30f9cfa62ef00d4602f68e2ba4b3718
f3307c168cf3fc4f0f22f37ef050116b
f342426d38a9ac97b438c11ad630d661
f35e03982a7dedd8c1a40ccdec5511c6
f370c0cbe88b451b3b37607eb5f5e495
f3a55672614ca5a748333b80b5778bdc
f3e62f793a92c700c23d94bcb83ba248
f404b95bd1148fa391ba37f136b35a3a
f4067b9bb19dc340df3663dbab48cda9
f40c9018612a3c2189187845a1f99e08
Abundance
PMI (hrs)
location
External
Internal

ASV Abundance − Batch 200

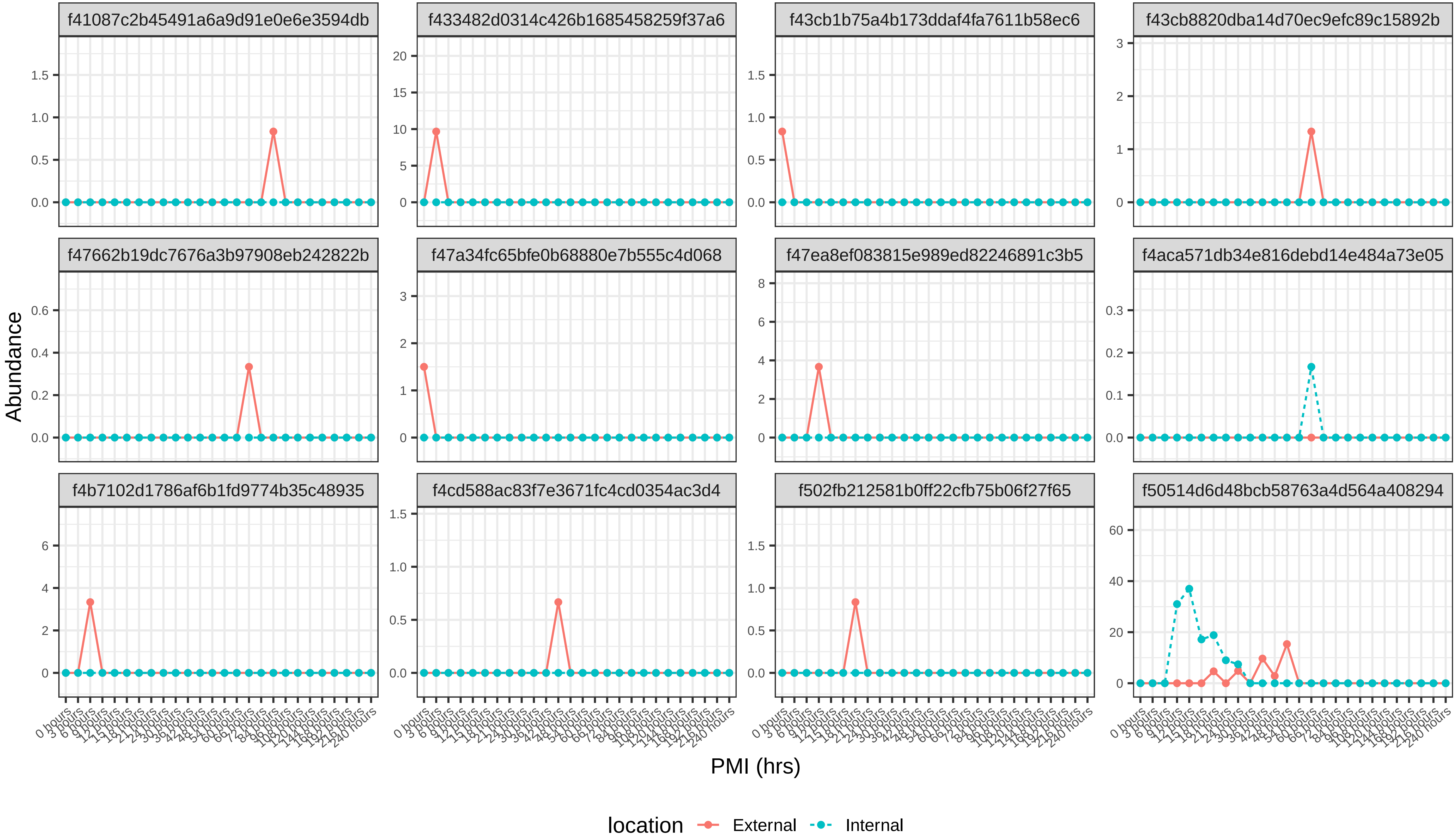

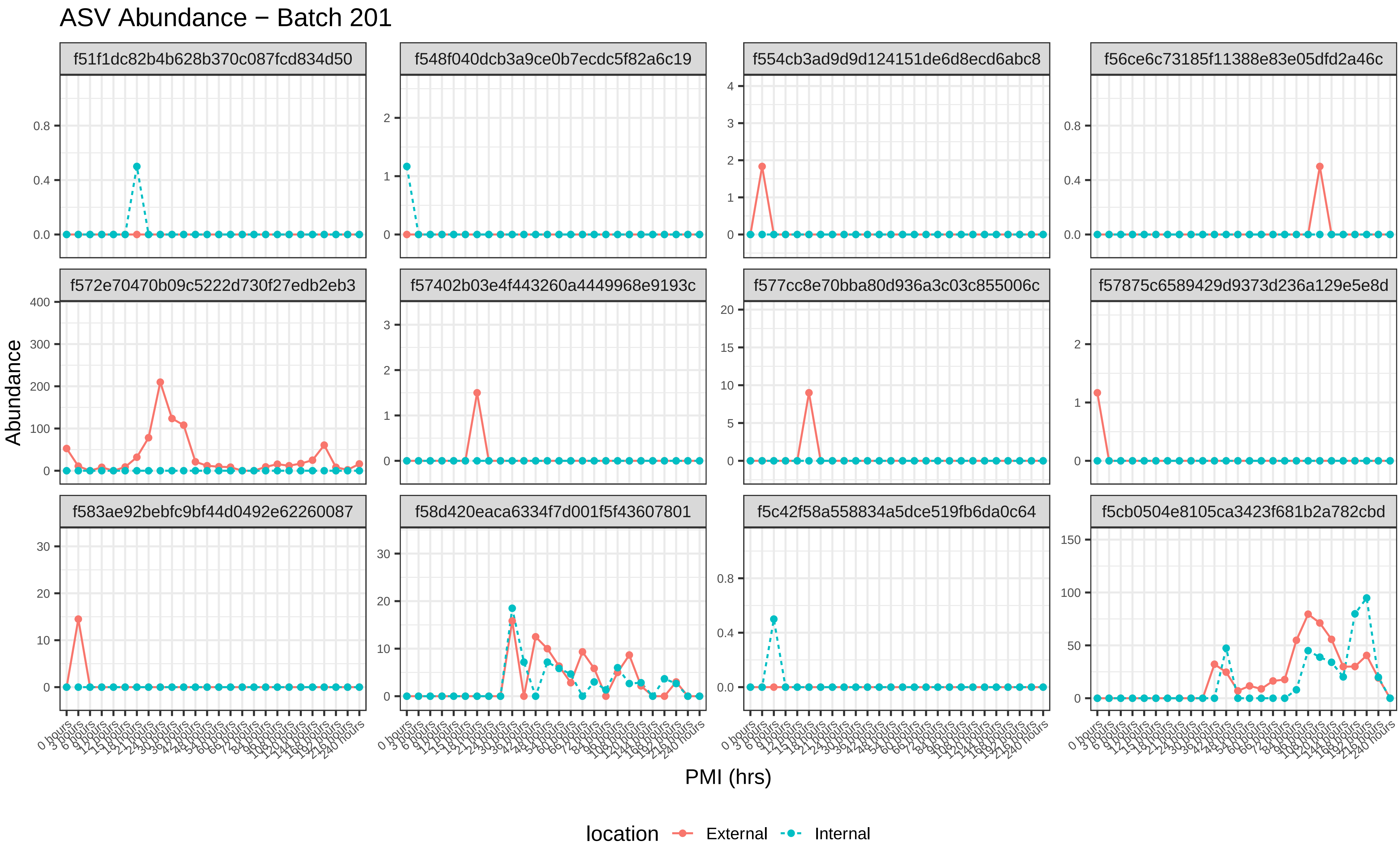
ASV Abundance − Batch 201
f51f1dc82b4b628b370c087fcd834d50
f548f040dcb3a9ce0b7ecdc5f82a6c19
f554cb3ad9d9d124151de6d8ecd6abc8
f56ce6c73185f11388e83e05dfd2a46c
f572e70470b09c5222d730f27edb2eb3
f57402b03e4f443260a4449968e9193c
f577cc8e70bba80d936a3c03c855006c
f57875c6589429d9373d236a129e5e8d
f583ae92bebfc9bf44d0492e62260087
f58d420eaca6334f7d001f5f43607801
f5c42f58a558834a5dce519fb6da0c64
f5cb0504e8105ca3423f681b2a782cbd
Abundance
PMI (hrs)
location
External
Internal

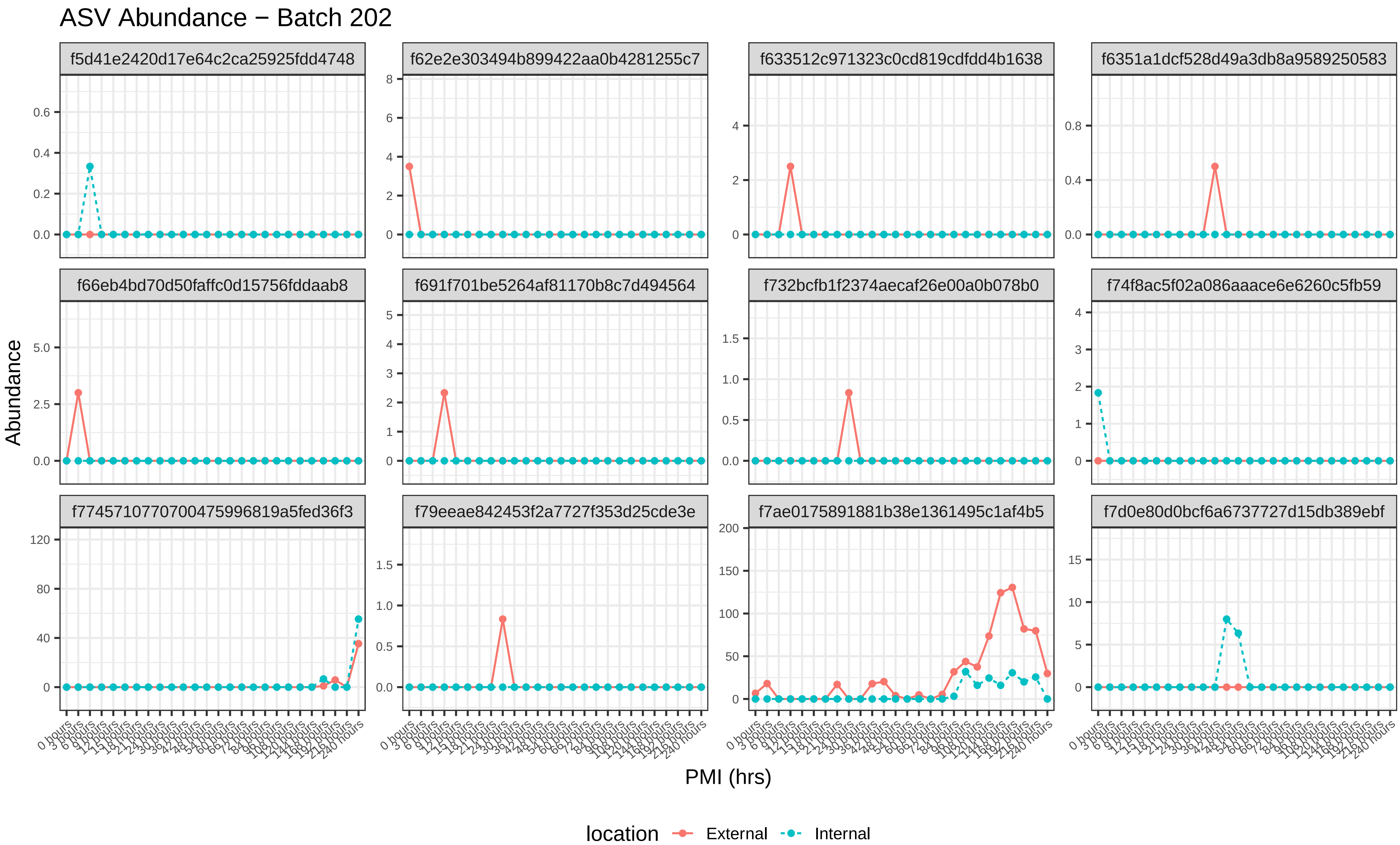
ASV Abundance − Batch 202
f5d41e2420d17e64c2ca25925fdd4748
f62e2e303494b899422aa0b4281255c7
f633512c971323c0cd819cdfdd4b1638
f6351a1dcf528d49a3db8a9589250583
f66eb4bd70d50faffc0d15756fddaab8
f691f701be5264af81170b8c7d494564
f732bcfb1f2374aecaf26e00a0b078b0
f74f8ac5f02a086aaace6e6260c5fb59
f774571077070047599681 9a5fed36f3
f79eeae842453f2a7727f353d25cde3e
f7ae0175891881b38e1361495c1af4b5
f7d0e80d0bcf6a6737727d15db389ebf
Abundance
PMI (hrs)
location
External
Internal

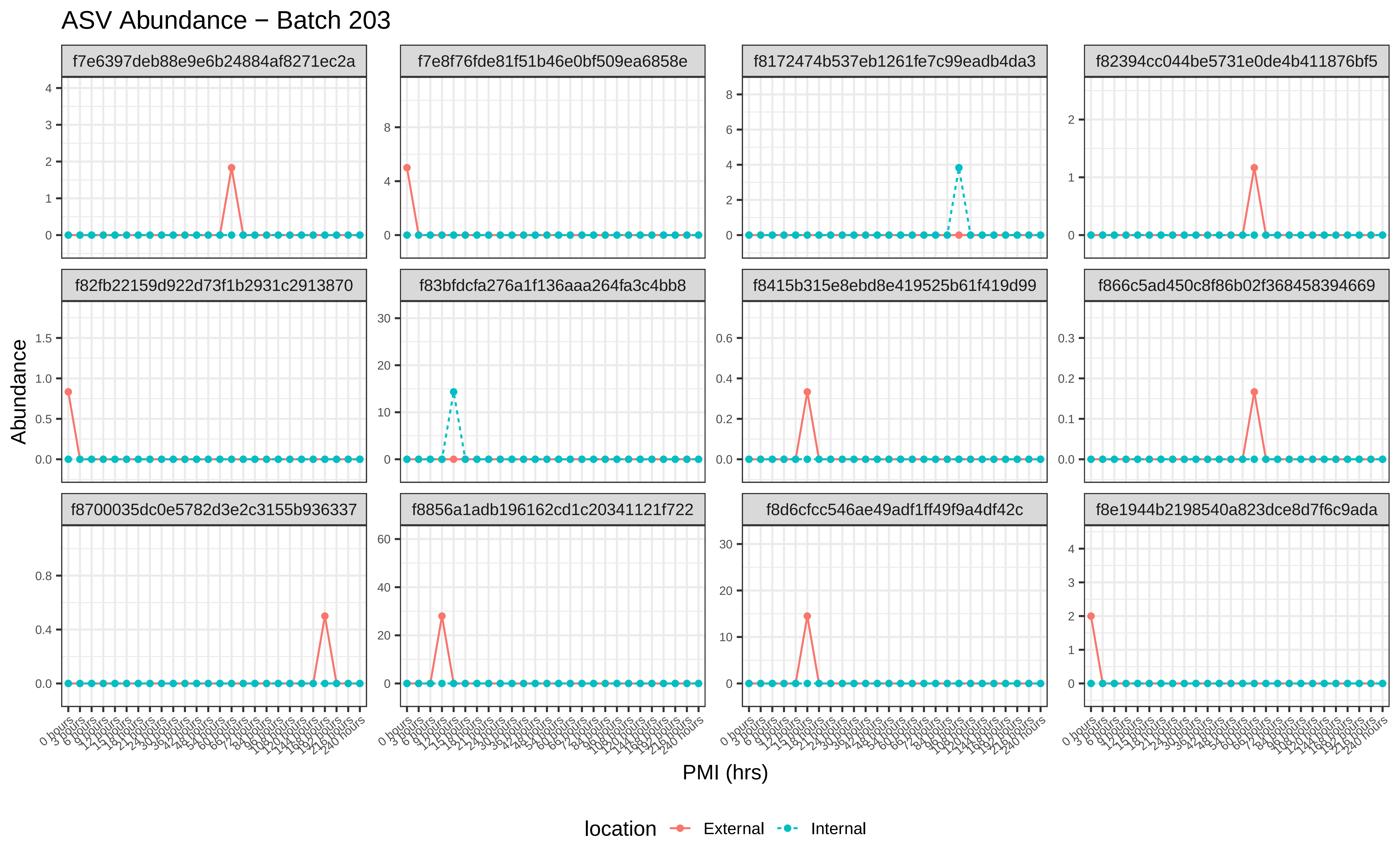
ASV Abundance − Batch 203
f7e6397deb88e9e6b24884af8271ec2a
f7e8f76fde81f51b46e0bf509ea6858e
f8172474b537eb1261fe7c99eadb4da3
f82394cc044be5731e0de4b411876bf5
f82fb22159d922d73f1b2931c2913870
f83bfdcfa276a1f136aaa264fa3c4bb8
f8415b315e8ebd8e419525b61f419d99
f866c5ad450c8f86b02f368458394669
f8700035dc0e5782d3e2c3155b936337
f8856a1adb196162cd1c20341121f722
f8d6cfcc546ae49adf1ff49f9a4df42c
f8e1944b2198540a823dce8d7f6c9ada
Abundance
PMI (hrs)
location
External
Internal

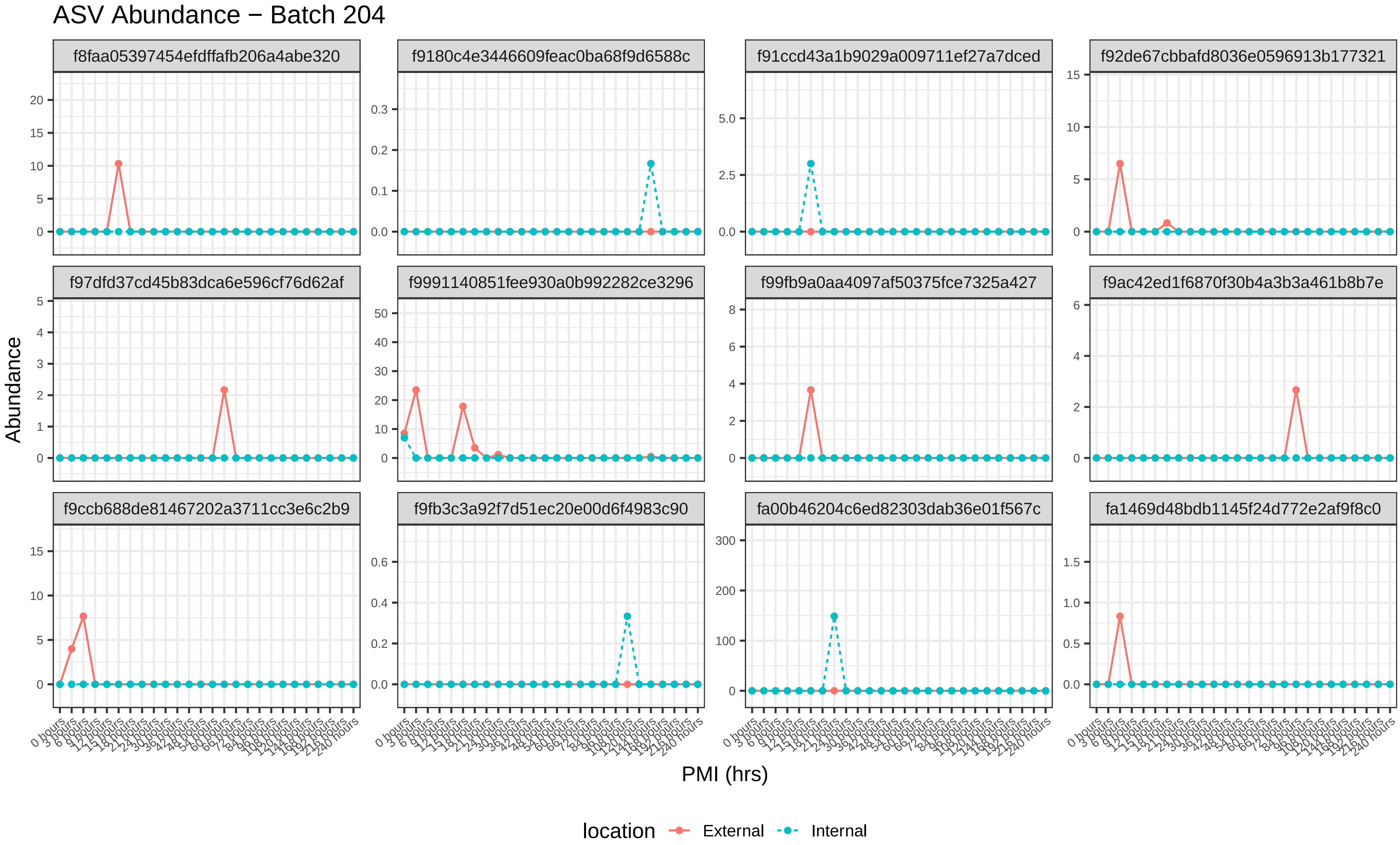
ASV Abundance − Batch 204
f8faa05397454efdffafb206a4abe320
f9180c4e3446609feac0ba68f9d6588c
f91ccd43a1b9029a009711ef27a7dced
f92de67cbbafd8036e0596913b177321
f97dfd37cd45b83dca6e596cf76d62af
f9991140851fee930a0b992282ce3296
f99fb9a0aa4097af50375fce7325a427
f9ac42ed1f6870f30b4a3b3a461b8b7e
f9ccb688de81467202a3711cc3e6c2b9
f9fb3c3a92f7d51ec20e00d6f4983c90
fa00b46204c6ed82303dab36e01f567c
fa1469d48bdb1145f24d772e2af9f8c0
Abundance
PMI (hrs)
location
External
Internal

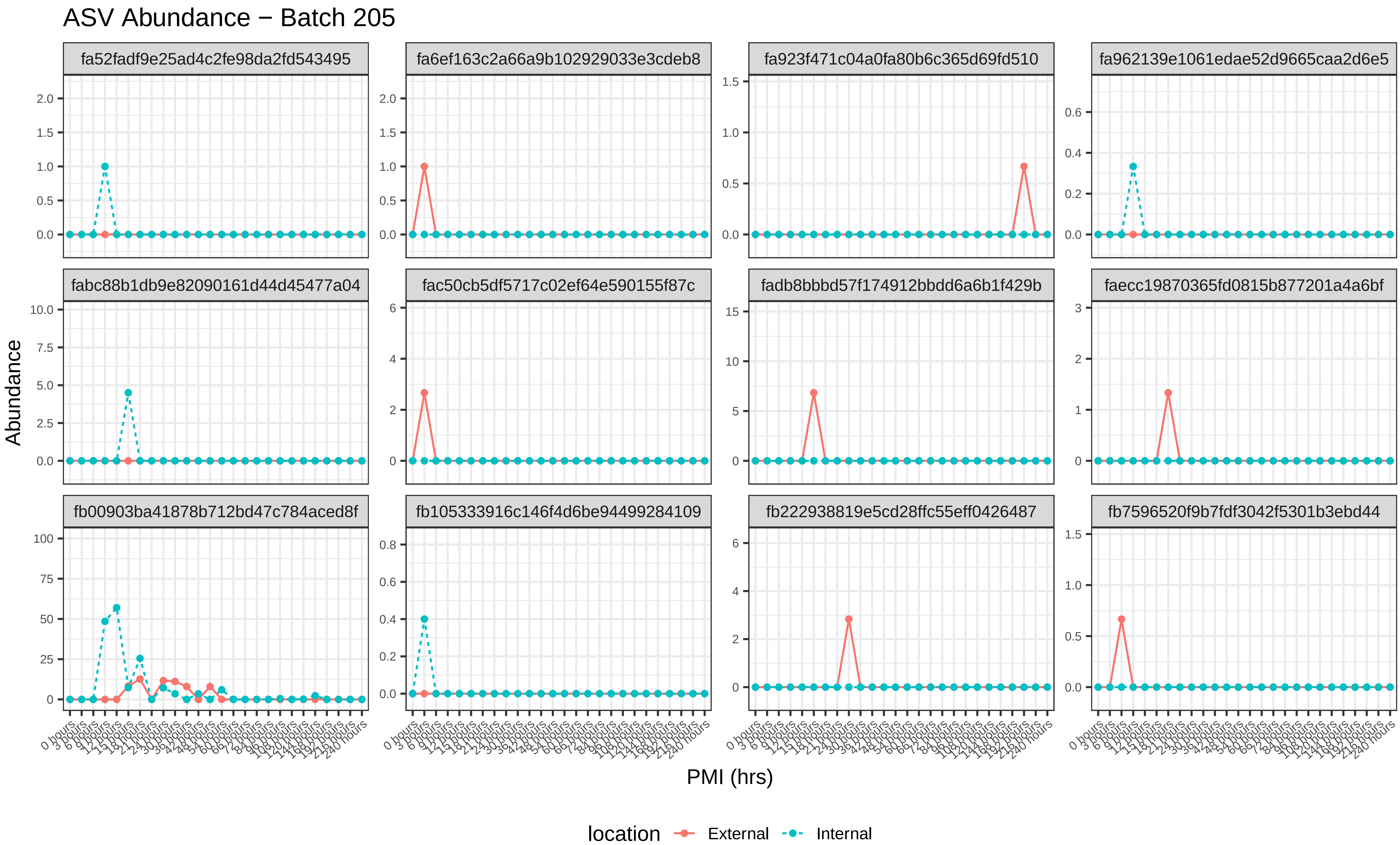
ASV Abundance − Batch 205
fa52fadf9e25ad4c2fe98da2fd543495
fa6ef163c2a66a9b102929033e3cdeb8
fa923f471c04a0fa80b6c365d69fd510
fa962139e1061edae52d9665caa2d6e5
fabc88b1db9e82090161d44d45477a04
fac50cb5df5717c02ef64e590155f87c
fadb8bbbd57f174912bbdd6a6b1f429b
faecc19870365fd0815b877201a4a6bf
fb00903ba41878b712bd47c784aced8f
fb105333916c146f4d6be94499284109
fb222938819e5cd28ffc55eff0426487
fb7596520f9b7fdf3042f5301b3ebd44
Abundance
PMI (hrs)
location
External
Internal

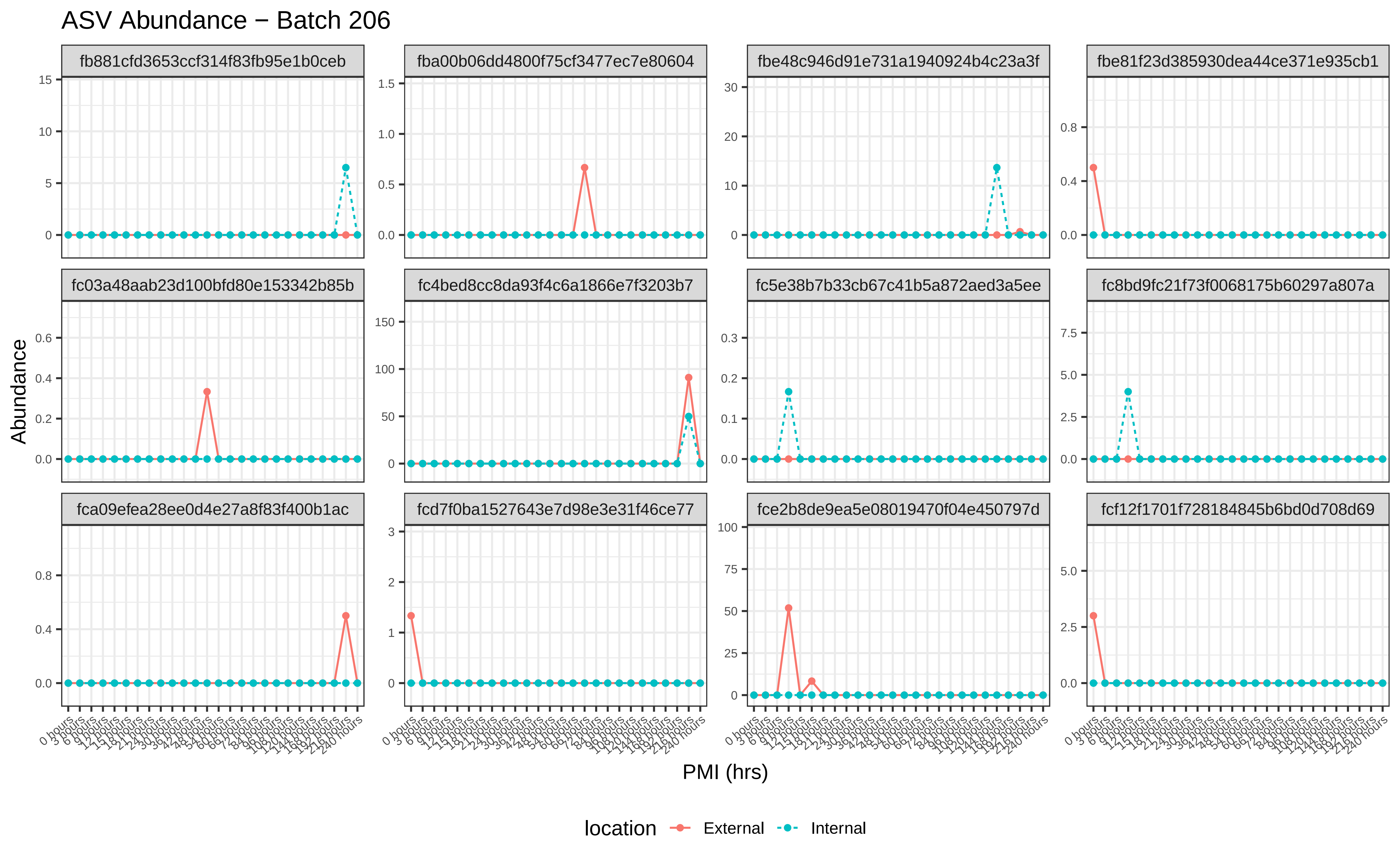

ASV Abundance − Batch 206
fb881cfd3653ccf314f83fb95e1b0ceb
fba00b06dd4800f75cf3477ec7e80604
fbe48c946d91e731a1940924b4c23a3f
fbe81f23d385930dea44ce371e935cb1
fc03a48aab23d100bfd80e153342b85b
fc4bed8cc8da93f4c6a1866e7f3203b7
fc5e38b7b33cb67c41b5a872aed3a5ee
fc8bd9fc21f73f0068175b60297a807a
fca09efea28ee0d4e27a8f83f400b1ac
fcd7f0ba1527643e7d98e3e31f46ce77
fce2b8de9ea5e08019470f04e450797d
fcf12f1701f728184845b6bd0d708d69
Abundance
PMI (hrs)
location
External
Internal

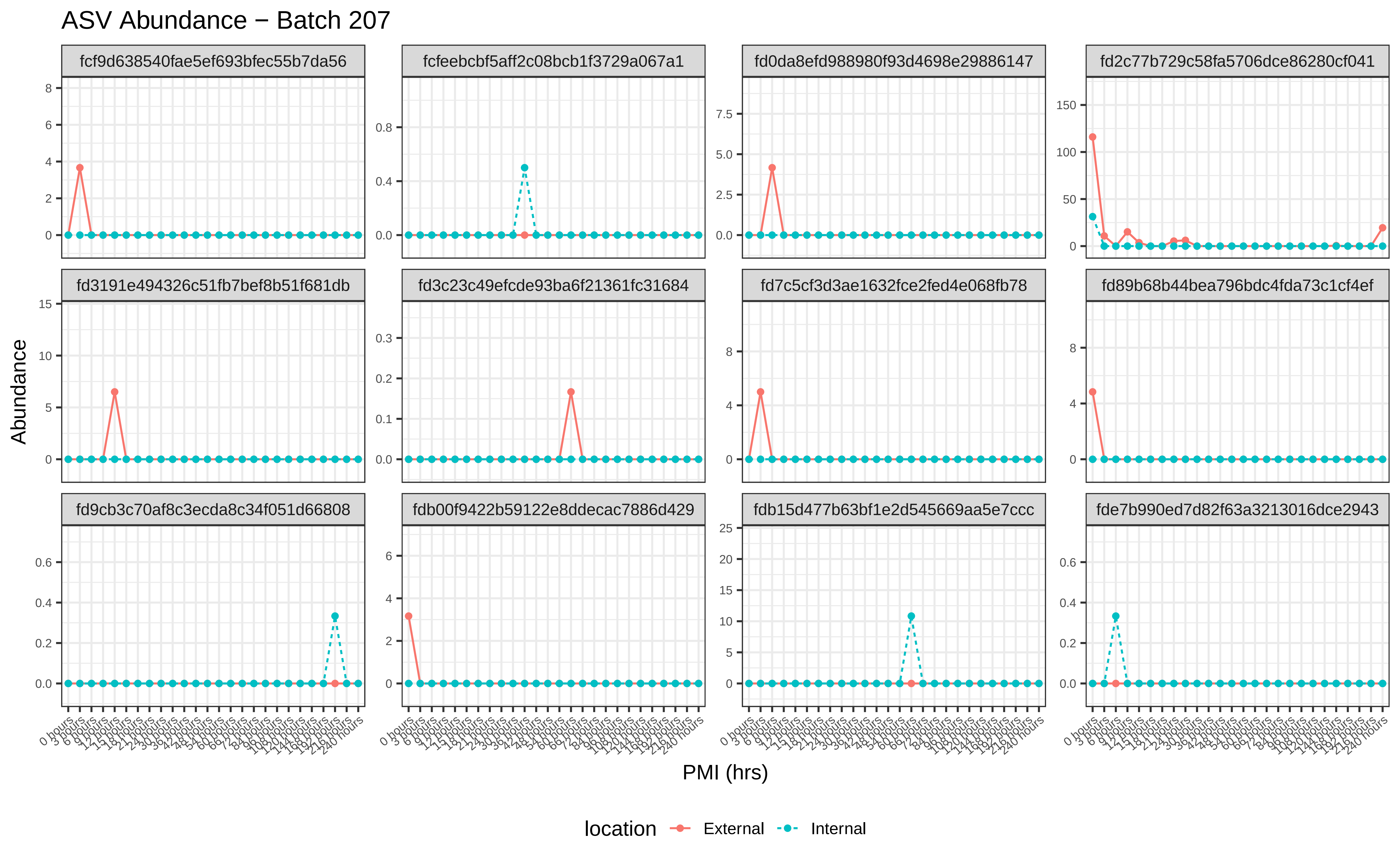

ASV Abundance − Batch 207
fcf9d638540fae5ef693bfec55b7da56
fcfeebcbf5aff2c08bcb1f3729a067a1
fd0da8efd988980f93d4698e29886147
fd2c77b729c58fa5706dce86280cf041
fd3191e494326c51fb7bef8b51f681db
fd3c23c49efcde93ba6f21361fc31684
fd7c5cf3d3ae1632fce2fed4e068fb78
fd89b68b44bea796bdc4fda73c1cf4ef
fd9cb3c70af8c3ecda8c34f051d66808
fdb00f9422b59122e8ddecac7886d429
fdb15d477b63bf1e2d545669aa5e7ccc
fde7b990ed7d82f63a3213016dce2943
Abundance
PMI (hrs)
location
External
Internal

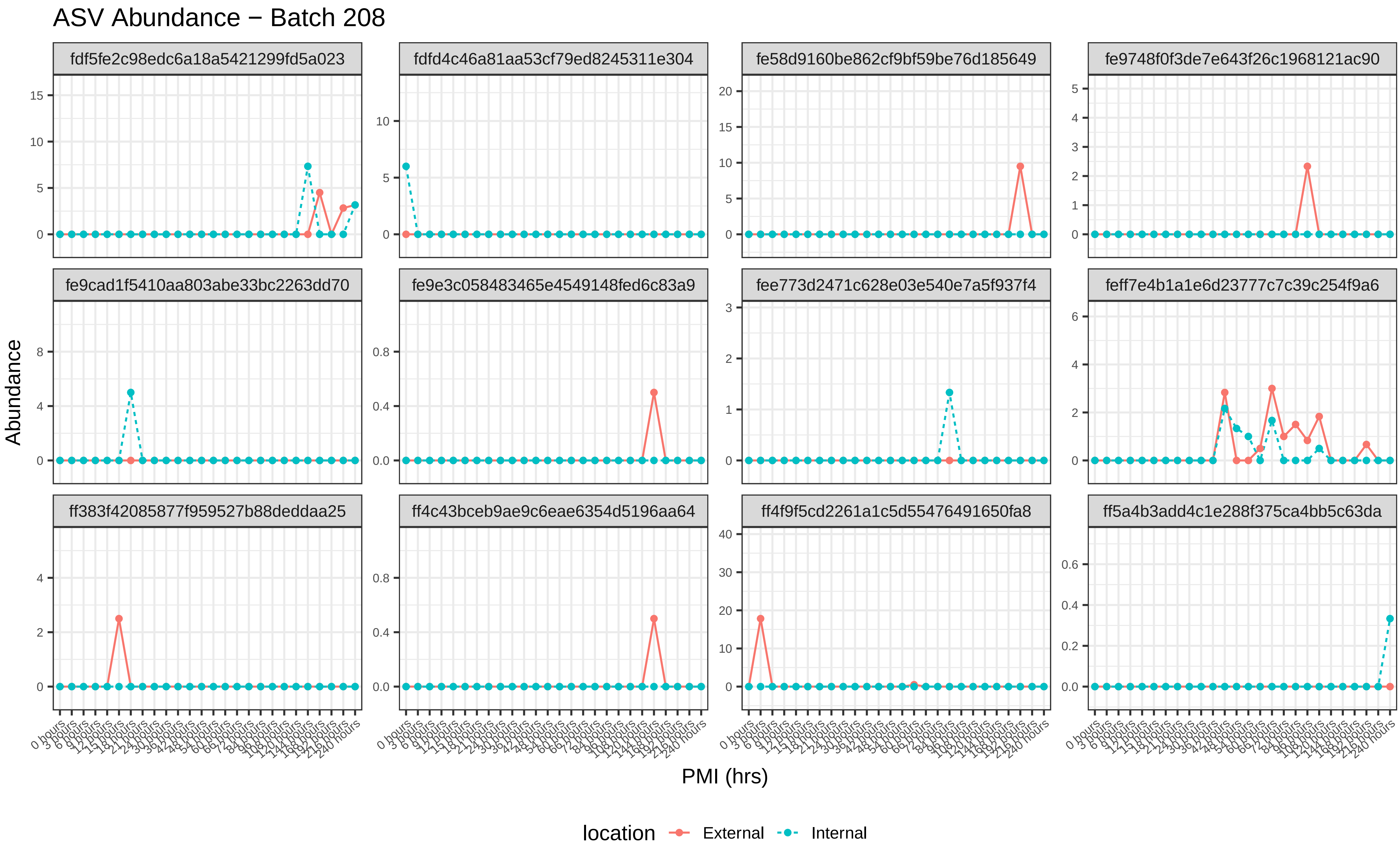

ASV Abundance − Batch 208
fdf5fe2c98edc6a18a5421299fd5a023
fdfd4c46a81aa53cf79ed8245311e304
fe58d9160be862cf9bf59be76d185649
fe9748f0f3de7e643f26c1968121ac90
fe9cad1f5410aa803abe33bc2263dd70
fe9e3c058483465e4549148fed6c83a9
fee773d2471c628e03e540e7a5f937f4
feff7e4b1a1e6d23777c7c39c254f9a6
ff383f42085877f959527b88deddaa25
ff4c43bceb9ae9c6eae6354d5196aa64
ff4f9f5cd2261a1c5d55476491650fa8
ff5a4b3add4c1e288f375ca4bb5c63da
Abundance
PMI (hrs)
location
External
Internal

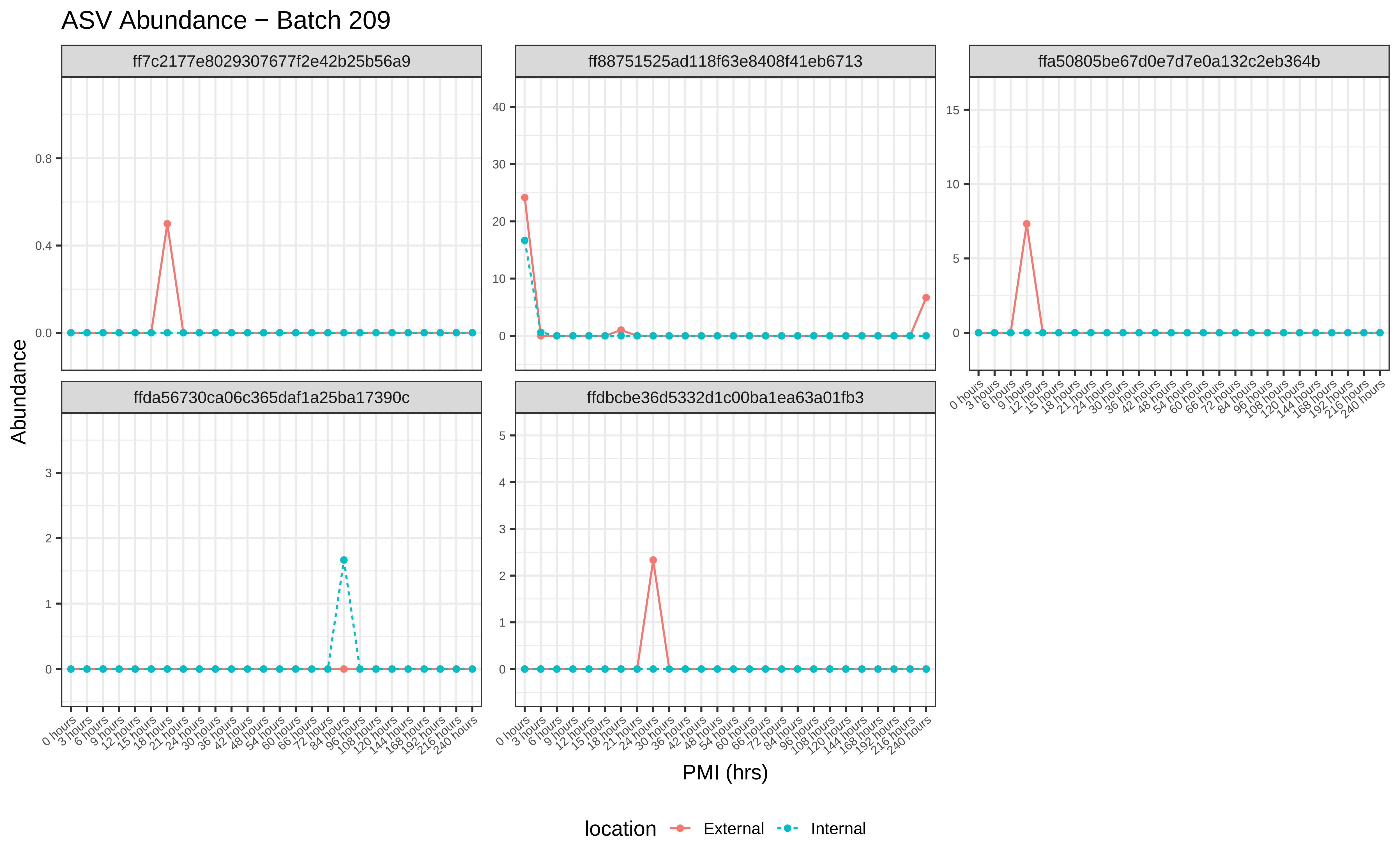

ASV Abundance − Batch 209
ff7c2177e8029307677f2e42b25b56a9
ff88751525ad118f63e8408f41eb6713
ffa50805be67d0e7d7e0a132c2eb364b
ffda56730ca06c365daf1a25ba17390c
ffdbcbe36d5332d1c00ba1ea63a01fb3
Abundance
PMI (hrs)
0 hours
3 hours
6 hours
9 hours
12 hours
15 hours
18 hours
21 hours
24 hours
30 hours
36 hours
42 hours
48 hours
54 hours
60 hours
66 hours
72 hours
84 hours
96 hours
108 hours
120 hours
144 hours
168 hours
192 hours
216 hours
240 hours
location
External
Internal
